# The traditional Chinese medicine Qiliqiangxin in heart failure with reduced ejection fraction: a randomized, double-blind, placebo-controlled trial

**DOI:** 10.1038/s41591-024-03169-2

**Published:** 2024-08-02

**Authors:** Iokfai Cheang, Wenming Yao, Yanli Zhou, Xu Zhu, Gehui Ni, Xinyi Lu, Shengen Liao, Rongrong Gao, Fang Zhou, Jiangang Shen, Alice Yeuk Lan Leung, Meng Jiang, Hong Kong, Ling Bai, Ailiman Mahemuti, Haitao Yuan, Yu-Gang Dong, Chun-Ka Wong, Qinghua Xu, Gaoxing Zhang, Jianhua Wu, Qi Lu, Junhai Zhang, Chunxi Cha, Qian Ren, Lu Fu, Bing Wang, Yongshun Xu, Houxiang Hu, Jing Dong, Zhuo Shang, Chaoping Yu, Songsen Li, Chen Yao, Lingling Gao, Haifeng Zhang, Anthony Rosenzweig, Zhenhua Jia, Xinli Li, QUEST Trial Committees and Investigators

**Affiliations:** 1 State Key Laboratory for Innovation and Transformation of Luobing Theory, Department of Cardiology, the First Affiliated Hospital of Nanjing Medical University, Jiangsu Province Hospital, Nanjing, China; 2 School of Chinese Medicine, The University of Hong Kong, Hong Kong SAR, China; 3 Renji Hospital, School of Medicine, Shanghai Jiaotong University, Shanghai, China; 4 Sichuan Provincial People’s Hospital, Chengdu, China; 5 The First Affiliated Hospital of Xi’an Jiaotong University, Xi’an, China; 6 First Affiliated Hospital of Xinjiang Medical University, Urumqi, China; 7 Shandong Provincial Hospital, Jinan, China; 8 The First Affiliated Hospital, Sun Yat-sen University, Guangzhou, China; 9 Queen Mary Hospital, LKS Faculty of Medicine, The University of Hong Kong, Hong Kong SAR, China; 10 Liaocheng People’s Hospital, Liaocheng, China; 11 Jiangmen Central Hospital, Jiangmen, China; 12 Xiuyan Manchu Autonomous County Central Peoples Hospital, Anshan, China; 13 Affiliated Hospital of Nantong University, Nantong, China; 14 Weixian Hospital of Traditional Chinese Medicine, Weixian, China; 15 Xinjiang Production and Construction Corps First Division Hospital, Aksu, China; 16 Sunsimiao Hospital of BUCM, Tongchuan, China; 17 The First Hospital of Harbin Medical University, Harbin, China; 18 Zouping People’s Hospital, Zouping, China; 19 Workers’ Hospital of Handan Iron and Steel Group Co., Handan, China; 20 Affiliated Hospital of North Sichuan Medical College, Nanchong, China; 21 The Second Affiliated Hospital of Shaanxi Medical University of Chinese Medicine, Xianyang, China; 22 The Second People’s Hospital of Bengbu, Bengbu, China; 23 Pidu District People’s Hospital, Chengdu, China; 24 Luoyang Central Hospital, Luoyang, China; 25 Peking University Clinical Research Institute, Beijing, China; 26 Institute for Heart and Brain Health, University of Michigan Medical Center, Ann Arbor, MI USA; 27 State Key Laboratory for Innovation and Transformation of Luobing Theory, Hebei Clinical Research Center of Cardiovascular Disease of Traditional Chinese Medicine, Shijiazhuang, China

**Keywords:** Heart failure, Outcomes research

## Abstract

Previous findings have indicated the potential benefits of the Chinese traditional medicine Qiliqiangxin (QLQX) in heart failure. Here we performed a double-blind, randomized controlled trial to evaluate the efficacy and safety of QLQX in patients with heart failure and reduced ejection fraction (HFrEF). This multicenter trial, conducted in 133 hospitals in China, enrolled 3,110 patients with HFrEF with NT-proBNP levels of ≥450 pg ml^−1^ and left ventricular ejection fraction of ≤40%. Participants were randomized to receive either QLQX capsules or placebo (four capsules three times daily) alongside standard heart failure therapy. The trial met its primary outcome, which was a composite of hospitalization for heart failure and cardiovascular death: over a median follow-up of 18.3 months, the primary outcome occurred in 389 patients (25.02%) in the QLQX group and 467 patients (30.03%) in the placebo group (hazard ratio (HR), 0.78; 95% confidence interval (CI), 0.68−0.90; *P* < 0.001). In an analysis of secondary outcomes, the QLQX group showed reductions in both hospitalization for heart failure (15.63% versus 19.16%; HR, 0.76; 95% CI, 0.64−0.90; *P* = 0.002) and cardiovascular death (13.31% versus 15.95%; HR, 0.83; 95% CI, 0.68−0.996; *P* = 0.045) compared to the placebo group. All-cause mortality did not differ significantly between the two groups (HR, 0.84; 95% CI, 0.70−1.01; *P* = 0.058) and adverse events were also comparable between the groups. The results of this trial indicate that QLQX may improve clinical outcomes in patients with HFrEF when added to conventional therapy. ChiCTR registration: ChiCTR1900021929.

## Abstract (web summary)

In a randomized controlled trial involving patients with heart failure with reduced ejection fraction, treatment with Qiliqiangxin, a traditional Chinese medicine derived from 11 types of plants, decreased the incidence of cardiac events, as compared to placebo.

## Main

Heart failure is a prevalent medical condition affecting approximately 64.3 million individuals worldwide, with over 6 million cases reported in the USA and 13 million in China^1,2^. Despite the availability of lifesaving guideline-directed medical therapy (GDMT) for heart failure with reduced ejection fraction (HFrEF), patients have a poor 5-year survival rate with an elevated risk of cardiovascular and heart failure admission^3,4^. Existing drugs may not effectively prevent disease progression in all patients. Ongoing research and development efforts aim to address the unmet needs in heart failure treatment and improve patient outcomes.

Traditional Chinese medicine (TCM), a major subdiscipline of complementary medicine, has increased in popularity in both Asian and Western countries in the past few decades and has the potential to supplement current therapies for the management of chronic heart failure^5,6^. The incomplete relief of symptoms, adverse effects of medication and individual variability in response all highlight the need for complementary medicine, including TCM. By addressing the gaps in conventional therapy, TCM may provide a holistic approach to heart failure management, potentially improving patient outcomes and quality of life. However, randomized controlled trials and rigorous scientific evidence have yet to demonstrate the ability of TCM to improve clinical outcomes in patients with heart failure beyond established therapies.

In our pilot study, Qiliqiangxin (QLQX) capsules, a TCM formula, demonstrated promising results when added to established heart failure treatment for patients with HFrEF (ChiCTR registration: ChiCTR1900021929). Administering QLQX capsules resulted in a reduction in the level of N-terminal pro-b-type natriuretic peptide (NT-proBNP) and improvement in heart failure symptoms, New York Heart Association (NYHA) functional class and 6-minute walking distance, as well as an improvement in quality of life. The study was conducted over a 12-week assessment period^7^. Although the QLQX group experienced lower rates of mortality and readmission than the placebo group, the sample size was too small to draw definitive conclusions. Preclinical studies have also indicated that QLQX has beneficial effects on myocardial metabolism, fibrosis and cardiac remodeling^8–11^. However, further research is needed to determine whether QLQX treatment improves clinical outcomes in patients with heart failure.

To address this question, the Qiliqiangxin in Heart Failure: Assessment of Reduction in Mortality (QUEST) study was developed with support from the National Key Research and Development program of China. This randomized placebo-controlled trial aimed to evaluate the efficacy and safety of QLQX for the treatment of major heart failure outcomes in patients with HFrEF.

## Results

### Patient disposition

**Fig. 1:**
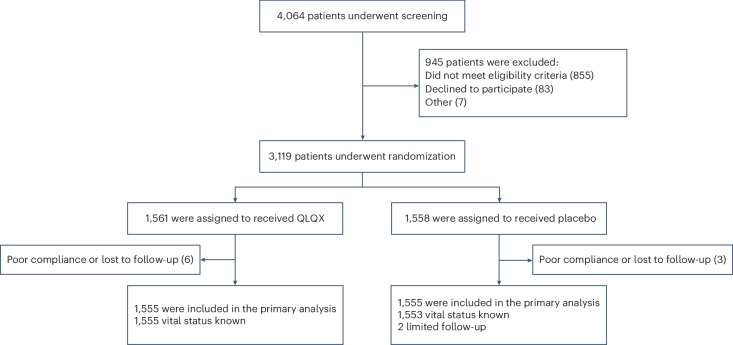
Patient enrollment and follow-up. Flowchart illustrating the screening, randomization and follow-up of patients in the study. Out of 4,064 patients screened, 3,119 were randomized to receive either Qiliqiangxin (1,561 patients) or placebo (1,558 patients). A total of 12 patients were lost to follow-up or showed poor compliance. Ultimately, 1,555 patients in each group were included in the primary analysis.

From 24 May 2019 through 24 May 2021, a total of 4,064 patients were screened and 3,119 were enrolled at 133 sites. Patients were randomly assigned to receive either QLQX or matched placebo capsules. Six patients in the QLQX group and three patients in the placebo group had no after-baseline efficacy and safety data and were therefore not included in the analysis. Additionally, two patients in the placebo group had limited follow-up (Fig. 1).

The characteristics of the patients and therapies for heart failure were well balanced between the trial groups at baseline (Table 1). The mean age of the enrolled patients was 63 years and 72% were male. At randomization, 52.6% were classified as having NYHA class II heart failure and the mean ejection fraction was 32.18%. The median NT-proBNP level was 1,730.8 pg ml^−1^. The use of concomitant GDMT was well balanced (all *P* > 0.05) in the two groups: 941 (60.5%) in the QLQX group and 962 (61.9%) in the placebo group received triple therapy (a β-blocker plus a mineralocorticoid receptor antagonist (MRA) plus an angiotensin-converting enzyme inhibitor (ACEi), angiotensin receptor blocker (ARB) or angiotensin receptor neprilysin inhibitor (ARNi)). Notably, 8.5% received a sodium glucose cotransporter 2 inhibitor (SGLT2i) and 56.9% received an ARNi.

**Table 1:** Baseline characteristics

Characteristics	QLQX *(n* = 1,555)	Placebo (*n* = 1,555)
Age (years)	62.56 ± 12.18	62.52 ± 12.54
Male sex (*n* (%))	1,095 (70.42)	1,148 (73.83)
Han ethnicity (*n* (%))	1,467 (94.34)	1,465 (94.21)
Height (cm)	166.4 ± 7.7	166.6 ± 7.9
Weight (kg)	67.0 ± 12.4	67.0 ± 12.8
Body mass index^a^ (kg m^−2^)	24.1 ± 3.6	24.0 ± 3.7
Smoking (*n* (%))	282 (18.14)	277 (17.81)
Principal cause of heart failure (*n* (%))		
Ischemic	1,156 (74.34)	1,165 (74.92)
Nonischemic	389 (25.02)	381 (24.50)
Unknown	10 (0.64)	9 (0.58)
Time from initial diagnosis of heart failure (*n* (%)) (median (IQR); months)	29 (10–63)	29 (10–72)
>3 years	577	588
≤3 years	978	967
Medical history (*n* (%))		
Hypertension	708 (45.53)	741 (47.65)
Diabetes mellitus	434 (27.91)	443 (28.49)
Atrial fibrillation	325 (20.90)	333 (21.41)
Myocardial infarction	261 (16.78)	262 (16.85)
Stroke	147 (9.45)	166 (10.68)
NYHA functional class (*n* (%))		
II	819 (52.67)	817 (52.54)
III	633 (40.71)	627 (40.32)
IV	103 (6.62)	111 (7.14)
Systolic blood pressure (mm Hg)	120.85 ± 17.55	120.79 ± 16.70
Resting heart rate (beats per minute)	78.07 ± 14.62	78.11 ± 15.49
Left ventricular ejection fraction (%)	32.20 ± 6.02	32.16 ± 6.08
NT-proBNP (median (IQR); pg ml^−1^)	1,692 (870.90−3,854.50)	1,763 (861.80−3,857.45)
Creatinine (µmol l^−1^)	85.72 ± 27.14	87.67 ± 31.91
eGFR^b^ (ml min^−1^ 1.73 m^−2^)	96.56 ± 17.13	96.03 ± 17.32
eGFR^b^ of <60 ml min^−1^ 1.73 m^−2^ (*n* (%))	33 (2.1)	31 (2.0)
Pacemaker and implantable cardioverter−defibrillator (*n* (%))	25 (1.6)	16 (1.0)
Left bundle branch blockage (*n* (%))	30 (1.9)	28 (1.8)
Heart failure medication (*n* (%))		
Diuretic	1,393 (89.58)	1,406 (90.42)
ACEi/ARB/ARNi	1,299 (83.53)	1,303 (83.79)
β-blocker	1,347 (86.62)	1,338 (86.05)
MRA	1,280 (82.3)	1,306 (84.0)
Triple therapy (ACEi/ARB/ARNi + β-blocker + MRA)	941 (60.5)	962 (61.9)
Double therapy (2 of ACEi/ARB/ARNi, β-blocker and MRA)	477 (30.7)	459 (29.5)
Single therapy (ACEi/ARB/ARNi or β-blocker or MRA)	129 (8.3)	127 (8.2)
Intolerant or contraindicated	8 (0.5)	7 (0.5)
Statin	921 (59.23)	938 (60.32)
Digitalis	379 (24.4)	398 (25.6)
ANRi	870 (55.9)	900 (57.9)
SGLT2i	144 (9.3)	119 (7.7)

Data are shown as mean ± s.d. unless indicated otherwise. Percentages may not total 100 because of rounding. Data were missing for the following characteristics: heart rate (1 patient in the QLQX group), systolic blood pressure (1 patient in the QLQX group), specific month of the initial diagnosis of heart failure (17 patients in the QLQX group and 11 patients in the placebo group), creatinine and eGFR (32 patients in the QLQX group and 34 patients in the placebo group) and the principal diagnosis of HFrEF to randomization (10 patients in the QLQX group and 9 patients in the placebo group). eGFR, estimated glomerular filtration rate; IQR, interquartile range.

^a^The body mass index was calculated as weight (kg) divided by the square of height (m).

^b^eGFR was calculated using the CKD-EPI formula.

### Primary outcomes

**Fig. 2:**
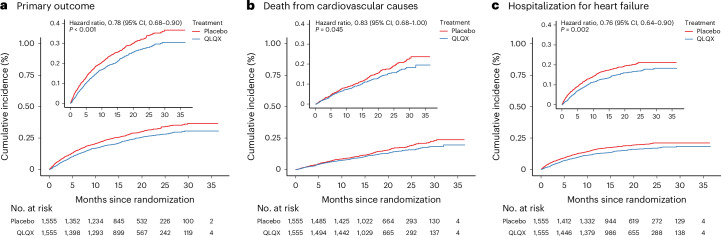
Kaplan–Meier curves for key study outcomes according to study group. **a−c**, Kaplan**−**Meier curves showing the cumulative incidences over time for the primary outcome (a composite of death from cardiovascular causes and first HHF) (**a**), death from cardiovascular causes (**b**) and first HHF (**c**). The insets show the same data with an expanded *y* axis.

The primary composite outcome of a first hospitalization for heart failure (HHF) or cardiovascular death occurred in 389 patients (25.02%) in the QLQX group and 467 patients (30.03%) in the placebo group (hazard ratio (HR), 0.78; 95% confidence interval (CI), 0.68−0.90; *P* < 0.001) (Table 2 and Fig. 2a).

**Table 2:** Primary and secondary outcomes

Outcome	QLQX (*n* = 1,555)	Placebo (*n* = 1,555)	HR (95% CI)	*P* value
**Primary composite outcome and components**				
Cardiovascular death or hospitalization due to heart failure	389 (25.02)	467 (30.03)	0.78 (0.68–0.90)	<0.001
Cardiovascular death	207 (13.31)	248 (15.95)	0.83 (0.68–1.00)	0.045
Hospitalization for heart failure	243 (15.63)	298 (19.16)	0.76 (0.64–0.90)	0.002
Primary endpoint in patients with principal ischemic etiology (1,156 versus 1,165)	295/1,156	367/1,165	0.76 (0.65–0.88)	<0.001
Cardiovascular death (cause-specific hazard model)^a^	–	–	0.80 (0.66–0.96)	0.02
Cardiovascular death (Fine–Gray regression model)^a^	–	–	0.80 (0.66–0.97)	0.02
**Secondary outcomes**				
Death from any cause	221 (14.21)	262 (16.85)	0.84 (0.70–1.01)	0.058
Secondary composite endpoint^b^	26 (1.67)	44 (2.83)	0.58 (0.35–0.94)	0.027
Treatment terminated because of worsening heart failure	0	3	–	0.083^c^
Successful resuscitation after cardiac arrest	2	1	–	0.563^c^
Malignant arrhythmia	12 (0.77)	24 (1.54)	0.46 (0.23–0.93)	0.029
Nonfatal stroke	12 (0.77)	17 (1.09)	0.73 (0.35–1.55)	0.419

Data are shown as *n* (%) unless indicated otherwise. HRs (QLQX versus placebo) and CIs were calculated using Cox proportional-hazards models. *P* values are two-sided.

^a^With noncardiovascular mortality as competing event.

^b^Data shown are from the primary analysis cutoff date (24 May 2022). For patients with multiple events, only the first event that contributed to the composite outcome was included.

^c^Calculated using the stratified log-rank test without adjustment for multiple comparisons.

The event rates for each component of the composite outcome favored QLQX. A total of 207 deaths (13.31%) in the QLQX group and 248 deaths (15.59%) in the placebo group were due to cardiovascular causes (HR, 0.83; 95% CI, 0.68–0.996; *P* = 0.045) (Table 2 and Fig. 2b). Of the patients receiving QLQX, 243 (15.63%) were hospitalized for heart failure compared to 298 patients (19.16%) receiving placebo (HR, 0.76; 95% CI, 0.64−0.90; *P* = 0.002) (Table 2 and Fig. 2c).

**Fig. 3:**
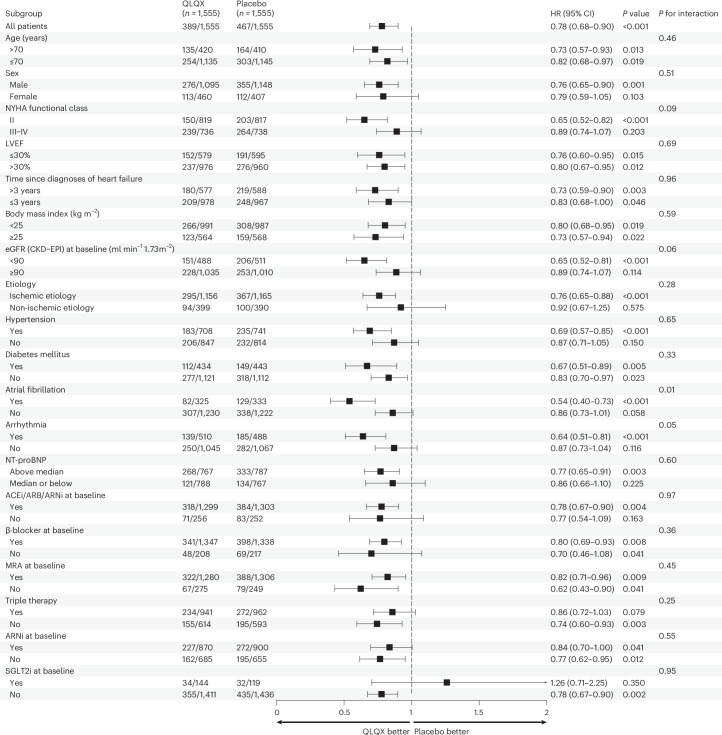
Primary composite outcome, according to subgroup. HRs plotted with 95% CIs were obtained via a Cox proportional-hazards model for the primary composite endpoint (death from cardiovascular causes or first HHF). The middle line represents an HR of 1.0. Error bars indicate 95% CIs. For eGFR, data were missing for 32 patients in the QLQX group and 34 patients in the placebo group. LVEF, left ventricular ejection fraction.

Using a cause-specific hazard model, the QLQX group had a significantly lower risk of cardiovascular death (HR, 0.80; 95% CI, 0.66−0.96; *P* = 0.02) compared to the placebo group. With the Fine−Gray competing risk regression model, the treatment also demonstrated a significant effect with a subdistribution HR of 0.80 (95% CI, 0.66−0.97; *P* = 0.02). The effect of QLQX on the primary outcome was generally consistent across prespecified subgroups, including in patients with or without ARNi and in patients with ischemic etiology, except in the subgroup defined according to SGLT2i, which showed wide CIs due to small numbers of participants (Fig. 3).

### Secondary outcomes

The incidence of the secondary composite outcome of abandoning treatment due to heart failure exacerbation, cardiac arrest resuscitation, malignant arrhythmia or nonfatal stroke was lower in the QLQX group than in the placebo group (HR, 0.58; 95% CI, 0.35−0.94; *P* = 0.027; Table 2). A total of 221 patients (14.21%) in the QLQX group and 262 patients (16.85%) in the placebo group died from any cause (HR, 0.84; 95% CI, 0.70−1.01; *P* = 0.058).

There was a greater decrease in serum NT-proBNP levels in the QLQX group (−444.00 (−1,401.00, 85.00) pg ml^−1^) than in the placebo group (−363.00 (−1,280.00, 183.00) pg ml^−1^) between baseline and patient follow-up at 3 months (*P* = 0.047). A total of 56.54% of patients in the QLQX group had reductions in NT-proBNP levels of at least 30% compared to 49.86% of patients in the placebo group (*P* = 0.002; Table 3).

**Table 3:** Changes in plasma NT-proBNP levels from baseline to follow-up at 3 months

Parameter	QLQX (*n* = 1,109)	*P* (intragroup)	Placebo (*n* = 1,109)	*P* (intragroup)	*P* (intergroup)
Difference in NT-proBNP^a^ (median (IQR); pg ml^−1^)	−444.00 (−1401.00, 85.00)	<0.001	−363.00 (−1,280.00, 183.00)	<0.001	0.047
Percent reduction in NT-proBNP^b^ (median (IQR); %)	38.84 (−6.83, 69.94)	–	29.67 (−16.42, 66.48)	–	0.001
Patients with reduction in NT-proBNP of >30% (*n* (%))	627 (56.54%)	–	553 (49.86%)	–	0.002

Intergroup significance was tested using Student's *t*-test for continuous variables and the chi-square or Wilcoxon test for categorical variables, with the Wilcoxon paired signed-rank test used for intragroup comparisons. *P* values are two-sided.

^a^Difference in NT-proBNP = baseline level − 3-month level.

^b^Percent reduction in NT-proBNP = (baseline level − 3-month level)/baseline level × 100.

### Safety outcome

The frequent serious adverse events and adverse events leading to discontinuation of the study drug are summarized in Extended Data Table 1.

Adherence to the trial drug was greater than 80% in 97.59% of patients. Adverse events related to the study drug occurred in 21 patients (1.35%) in the QLQX group and 14 patients (0.9%) in the placebo group (*P* = 0.308). QLQX treatments was stopped in 16 patients and placebo was stopped in 9 patients for reasons other than death (1.3% versus 0.58%; *P* = 0.228). Gastrointestinal symptoms (abdominal distention, diarrhea, constipation, belching, nausea, indigestion) occurred in 15.69% of patients receiving QLQX and in 14.53% of patients receiving placebo (*P* = 0.395). Adverse events rarely led to treatment discontinuation.

There was no notable excess of any event in the QLQX group other than insomnia (3.67% versus 1.86%*;* *P* = 0.003). A total of 59 (3.79%) patients in the QLQX group and 70 (4.50%) patients in the placebo group had worsening renal function (*P* = 0.369); 40 (2.57%) patients in the QLQX group and 48 (3.09%) patients in the placebo group had increased liver enzyme levels (*P* = 0.449); and 51 (3.28%) patients in the QLQX group and 53 (3.41%) patients in the placebo group had hypertriglyceridemia (*P* = 0.921).

### Sensitivity analysis

**Extended Data Fig. 1:**
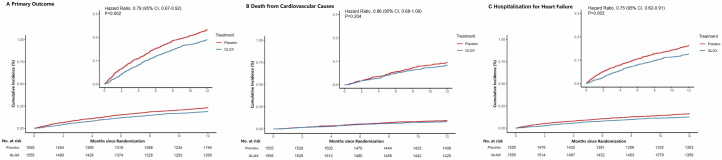
Sensitivity analysis for the key study outcome, According to 12-months follow-up. (**A-C**). Kaplan-Meier curves showing the cumulative incidences over time for the primary outcome (a composite of death from cardiovascular causes or first hospitalization for heart failure) (**A**), death from cardiovascular causes (**B**) and first hospitalization for heart failure (**C**). Insets show the same data with an expanded y axis. QLQX denotes Qiliqiangxin capsule, HR: hazard ratio, CI: confident interval.

When censoring at follow-up at 12-months, the analysis results were overall concordant with those of the primary analyses (Extended Data Fig. 1). The use of QLQX was associated with a lower risk of first HHF (HR, 0.75; 95% CI, 0.62−0.91; *P* = 0.003) and the primary outcome of death from cardiovascular causes or first HHF (HR, 0.79; 95% CI, 0.67−0.92; *P* = 0.002) compared to placebo administration. In the per protocol set (PPS), the primary and secondary outcomes also demonstrated consistent results (Extended Data Tables 2 and 3). Based on the intention-to-treat principle, complete case analysis (including the nine cases excluded after randomization due to missing follow-up data) also showed overall consistent results (major adverse cardiovascular events: 389 (24.92%) with QLQX versus 468 (30.04%) with placebo; HR, 0.76; 95% CI, 0.68−0.90; *P* < 0.001, Extended Data Table 4). Of note, these sensitivity analyses were not prespecified.

## Discussion

TCM has been established through careful clinical observation, but only a few traditional medicines have been subjected to randomized controlled trials to examine their efficacy as supplements to established clinical care. In this study, the effect of QLQX on clinical outcomes was evaluated in patients with HFrEF who were already receiving treatment according to established guidelines. The results showed that QLQX reduced the primary endpoint of death from cardiovascular causes and/or HHF by 22% compared to placebo, and secondary outcome results generally favored QLQX, with reductions observed in both components of the composite outcome in the QLQX group.

Benefits with respect to cardiovascular mortality and HHF were generally consistent in the subgroups, even in the subgroups defined by the novel guideline-recommended medication of ARNi for heart failure. Although there was no discernable difference between the rates of cardiovascular death at 12 months, as demonstrated in the subgroup analysis, it is noteworthy that this might be primarily driven by factors of NYHA class II, ischemic cardiomyopathy and patients without ARNI at baseline. Additionally, the point estimate in patients receiving SGLT2i raises the possibility of an adverse interaction, but relatively few patients received an SGLT2i; thus, the CI was too large to infer either a positive or negative incremental role of QLQX in this population. Therefore, our results should be interpreted within the context of the specific patient population. Although both ARNi and SGLT2i have become established treatments for certain subgroups of patients with heart failure, further studies, including larger-scale clinical trials and comparative studies, would provide a more comprehensive understanding of the efficacy, safety and potential synergistic effects of these treatment approaches.

It is noteworthy that the overall rate of death from any cause was numerically lower for patients who received QLQX than for those who received placebo, although this trend was not statistically significant (14.21% versus 16.85%). Both groups also demonstrated reduced serum NT-proBNP levels at the 3-month follow-up, which corroborates the findings of our previous trial^7^. This result could be attributed to regular monitoring and engagement with health-care professionals due to participation in the trials leading to improved cardiac health. This further supports the beneficial effects of QLQX in addition to standard medical management for HFrEF.

Distinct from previous trials, most of the patients included here were already receiving recommended treatment, highlighting a population with a much higher risk for first HHF and death from cardiovascular causes, indicating the need for medical attention. The adverse events were balanced between the groups, and only a few patients experienced drug-related adverse events (1.35% in the QLQX group and 0.90% in the placebo group), indicating that QLQX capsules were safe and well tolerated with no increase in renal and hepatic adverse events compared to standard care. Neither of these major adverse events was common (with each occurring in <6.2% of patients, with no between-group differences). Gastrointestinal-related symptoms were the most frequently reported adverse events with no statistical differences between the treatment and control groups. Overall, few patients stopped taking QLQX or placebo because of any adverse effect (<1.5% of patients in each treatment group). Of note, insomnia was rare, generally mild and resolved over time; however, its incidence was significantly higher in the QLQX group.

Although the current study supports the efficacy of QLQX in reducing heart failure morbidity and mortality, potentially extending a therapeutic role for complementary therapy with QLQX in the management of chronic heart failure, the use of a ‘pre-mix’ of QLQX might be in direct conflict with the conventional reductionist approach of modern medicine^12,13^. Although interpretation of QLQX's efficacy from TCM physiology has certain similarities to the cardiovascular continuum^14^, the underlying mechanisms by which QLQX improves prognosis heart failure remain uncertain.

Numerous studies have been performed on QLQX to determine its effects on acute and chronic cardiac injury and stress. These studies have focused on nutrient surplus and deprivation signaling, autophagy, and PPARγ coactivator-1ɑ (PGC-1ɑ) and its downstream transcription factors. The QLQX formula and its components offer protection against myocardial hypertrophy, fibrosis and cardiac remodeling^10,15–19^, increasing water excretion^20^ and promoting microangiogenesis^21,22^. QLQX has been found to improve cardiac hypertrophy by upregulating PPARγ and PGC-1α, reduce cardiomyocyte apoptosis caused by high-glucose environments, and prevent and treat heart failure through various mechanisms, including upregulation of PPARγ and downregulation of miRNA-22. Among the components in QLQX, studies have demonstrated that citri reticulatae pericarpium and its active monomer nobiletin might contribute significantly to such protective effects by attenuating adverse cardiac remodeling^23–25^. These results indirectly support the potential of QLQX as a complementary therapy for the treatment of heart failure. Further studies are warranted to explore the effective molecule(s) and synergistic interactions in QLQX and its corresponding individual components.

Traditional medicines in TCM have been demonstrated to contain isoprene oligomers with a diterpenoid or triterpenoid structure, which are known to exert cardiovascular effects by signaling through nutrient surplus and nutrient deprivation pathways. QLQX, a diterpene- and triterpene-replete combination of herbs, might be a focused pharmacological probe. QLQX ameliorates oxidative stress, maladaptive hypertrophy, apoptosis, and proinflammatory and profibrotic pathways in prolonged cardiac injury by improving mitochondrial health and mitophagy and promoting healthy glucose and fatty acid metabolism and ATP production. The nutrient deprivation pathways involved in the mechanism of action of QLQX are also involved in the mechanism of action of SGLT2i, which are also derived from a plant source^26^. The potential interaction between QLQX and SGLT2i requires further experimental work and clinical trials.

Although there is considerable experience with QLQX in patients with heart failure, most trials have been small and of short duration. In previous meta-analyses of studies^27,28^, it was shown that the combination of QLQX capsules with standard heart failure treatment can lead to improvement in clinical symptoms and a better quality of life for patients with chronic heart failure. However, it is important to note that these studies had a high risk of bias and the long-term prognosis of patients with HFrEF under the influence of QLQX has not been extensively studied. Therefore, randomized controlled studies using evidence-based methods are urgently needed to verify these findings. Distinct from previous studies, the current study provides a rigorous example of how traditional medicine can be effectively translated into scientific research. We hope that our study will encourage further exploration and research into the potential efficacy of TCM in treating other medical conditions.

Several limitations of this study are worth noting. First, generalization of the results may be limited due to potential differences in effectiveness across populations that include predominantly Chinese Han patients, variations in background treatments, the acceptability of the treatments in other settings and whether similar adherence is expected in other populations. Second, although the study did not require baseline ARNi and SGLT2i use, it is worth noting that there were no significant differences in drug use between the two groups. Because the QUEST study started before approval of SGLT2i for the treatment of heart failure in China, the baseline use of SGLT2i was relatively low. Studies of the effects of QLQX in addition to current GDMT, including SGLT2i and device therapy, are warranted. Third, it is important to acknowledge that cardiac resynchronization therapy was excluded because of the low implantation rates in China^29^, as this could limit the generalizability of the study results to other settings or populations. Fourth, despite enrolling patients using a certain diagnostic cutoff value for NT-proBNP to characterize the heart failure phenotype, the mean value was similar to that of the study populations in other relevant trials and patients in the community. Finally, the study was conducted during the COVID-19 pandemic, which limited onsite follow-up. To minimize the impact of the pandemic on the study outcomes, we conducted a portion of the follow-up visits virtually while adhering to social distancing guidelines, and the investigational products were delivered to the participants.

In conclusion, we conducted a multicenter clinical trial evaluating the efficacy and safety of QLQX capsules in patients with HFrEF. At a median follow-up of 18.3 months, the incidence of the primary endpoint of first HHF and cardiovascular death was significantly lower in the QLQX group than in the placebo group. These findings provide compelling evidence supporting the use of QLQX in patients with chronic heart failure undergoing standard treatment. Additionally, these findings highlight the potential additive value of harmonizing traditional Chinese and modern medical approaches.

## Methods

### Study design and setting

The executive committee designed and oversaw the conduct and analysis of the trial in collaboration with the sponsor, Shijiazhuang Yiling Pharmaceutical. The safety of patients in the trial was overseen by an independent data safety monitoring and clinical event adjudication committee (for a complete list of the committee members see Supplementary Information, ‘QUEST committees and investigators’).

This was a randomized, double-blind, placebo-controlled, parallel-group, event-driven, multicenter clinical study. The trial design was conducted and is reported in accordance with the protocol and statistical analysis plan (SAP), which are available with the full text in the supplementary Information (Supplementary Information, ‘Protocol and statistical analysis plan’)^30,31^. The study adhered to the CONSORT guidelines. The protocol was reviewed and approved by the independent ethics committee of the First Affiliated Hospital of Nanjing Medical University (approved no. of ethics committee: 2018-SR-275) and the ethics committee of each participating study center. The trial was registered at http://www.chictr.org.cn, registration no. ChiCTR1900021929 (registration date: 16 March 2019).

Data were collected using an electronic data capture application and Epidata v.3.1 software and managed by the independent statistics committee in strict accordance with a predefined SAP. The analyses were conducted by independent statisticians from the Peking University Clinical Research Institute. The first draft of the manuscript was prepared by the first author, who had unrestricted access to the data, and was reviewed and edited by all authors. All authors made the decision to submit the manuscript for publication and assume responsibility for the accuracy and completeness of the analysis.

### Participants

The enrollment period was from 24 May 2019 to 24 May 2021. The eligibility requirements at screening included an age of at least 18 years, left ventricular ejection fraction (LVEF) of 40% or less, NYHA functional grading of II to III and stable clinical symptoms. Patients diagnosed as grade IV within 2 weeks before enrollment were also included in the study. Patients were required to have a plasma NT-proBNP level of ≥450 pg ml^−1^.

All patients (in both the QXQL and placebo groups) were required to receive standard heart failure drug therapy following the guidelines for the diagnosis and treatment of heart failure in China^32^, including an ACEi, ARB or ARNi, a β-blocker and an MCA. The optimal therapeutic dose of these drugs was required, except in the case of contraindication or intolerance.

The detailed inclusion and exclusion criteria were as follows. Inclusion criteria: (1) signed informed consent; (2) age of ≥18 years at the time of consent; (3) established documented diagnosis of heart failure for at least 3 months according to the Chinese heart failure diagnosis and treatment guidelines issued by the Chinese Medical Association Cardiovascular Branch; (4) LVEF of ≤ 40% (by echocardiogram, radionuclide imaging, ventriculogram, contrast angiography or cardiac magnetic resonance imaging); (5) NYHA cardiac functional grading of II to III, with stable clinical symptoms, or diagnosis as grade IV within 2 weeks before enrollment; (6) serum NT-proBNP level of ≥450 pg ml^−1^; (7) receipt of a standardized baseline treatment regimen without dose adjustment given intravenously for at least 2 weeks before enrollment; and (8) no use of another TCM medicineor herbs having the same contents as QLQX, such as Danshen and Tongxinluo capsules. Exclusion criteria: (1) heart failure caused by valvular disease, congenital heart disease, pericardial disease, arrhythmia or noncardiaogenic disease or caused by vital organ failure (such as renal failure, hepatic failure, etc.), right-sided heart failure caused by pulmonary or other definite causes or acute heart failure; (2) plans to undergo coronary revascularization (percutaneous coronary intervention or coronary artery bypass grafting) or cardiac synchronization therapy after randomization or receipt of cardiac resynchronization therapy before enrollment; (3) any condition other than a cardiovascular disease, including but not limited to, malignant tumor, severe mental illness, hematopoietic diseases, neuroendocrine system disease, liver transaminase and alkaline phosphatase levels more than three times the upper limit of normal, abnormal renal function, a serum creatinine level of >2 mg dl^−1^ (176.82 μmol l^−1^) and a potassium level of >5.5 mmol l^−1^; (4) left ventricular outflow tract obstruction, myocarditis, aortic aneurysm, aortic dissection or obvious hemodynamic changes caused by an unrepaired valve; (5) cardiogenic shock, uncontrollable malignant arrhythmia, sinus or atrioventricular block at second degree, type II or above, without pacemaker treatment, progressive unstable angina pectoris or acute myocardial infarction; (6) uncontrolled hypertension, defined as a systolic blood pressure of ≥180 mmHg and/or a diastolic blood pressure of ≥110 mmHg, or a systolic blood pressure of <90 mmHg and/or a diastolic blood pressure of <60 mmHg; (7) participation in another clinical study with an investigative product during the month before enrollment; (8) women of child-bearing potential (that is, those who were not chemically or surgically sterilized or who were not postmenopausal) who were not willing to use a medically accepted method of contraception that was considered reliable in the judgment of the investigator, from the time of signing the informed consent to the end of the study and four weeks thereafter, women who had a positive pregnancy test at enrollment or randomization, or women who were breast-feeding; (9) an allergic constitution (known to be allergic to the research drug); and (10) inability of the patient, in the opinion of the investigator, to understand and/or comply with study medications or procedures or any conditions that might render the patient unable to complete the study.

### Investigational products

QLQX has 11 components. The proportions of these when preparing 1,000 capsules are as follows: astragali radix, 450 g; ginseng radix et rhizoma, 225 g; aconiti lateralis radix praeparata, 112.5 g; *Salvia* miltiorrhiza radix et rhizoma, 225 g; Descurainiae semen, 150 g; alismatis rhizoma, 225 g; polygonati odorati rhizoma, 75 g; cinnamomi ramulus, 90 g; carthami flos, 90 g; Periplocae cortex, 180 g; citri reticulatae pericarpium, 75 g.

**Extended Data Fig. 2:**
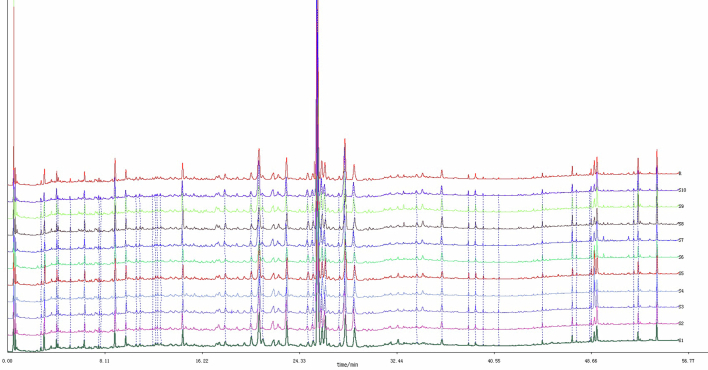
Ultra Performance Liquid Chromatography (UPLC) fingerprint for ten different batches of Qiliqiangxin capsules (QLQX). Ten batches of QLQX were analyzed by UPLC to establish reference fingerprints representing the typical patterns, demonstrating uniformity across batches. The x-axis represents time (minutes). R denotes Reference, and S1-10 denote Sample 1 through Sample 10.

UPLC fingerprint analysis combined with chemometric methods was applied to evaluate the differences and similarities in the chemical constituents of QLQX capsules from the ten batches, which showed good consistency in preparation (Extended Data Fig. 2 and Extended Data Table 5).

To mimic the experimental drug, scorch-fried medicated leaven and caramel coloring were used in the placebo. The matching placebo capsules had identical color, weight, size, smell, specification, property of contents, labels and packaging as the QLQX capsules to ensure blinding during the study. Shijiazhuang Yiling Pharmaceutical (Shijazhuang, People’s Republic of China) provided the investigative products (QLQX capsule and the matching placebo capsules) for this research. An independent third party conducted blind coding of the experimental drug and placebo, rendering them virtually indistinguishable to participants and investigators.

The investigative product was manufactured consistently to ensure the quality and safety of the drug and strictly adhered to the good manufacturing practices of Chinese national drug production. Details of the manufacturing process, product controls and composition are presented in chemistry manufacturing and control (CMC) reports (Supplementary Information, ‘CMC report’).

### Randomization and masking

Participants were randomly assigned (1:1) to receive QLQX or placebo in addition to the established heart failure medication regimen. This study adopted a block randomization method, and the participants were randomly grouped and managed through the randomization and trial supply management (RTSM) system. Randomization and drug numbers were generated by an independent statistician from the Peking University Clinical Institute using SAS software and were integrated into the RTSM system. The allocation list was stored in the RTSM system and was not available to any member of the research team. At each participating hospital, patients who provided written informed consent and met the study criteria were randomized by investigators who obtained the assigned treatment and code number from the RTSM system. Each package was labeled with a unique code number that was used to assign treatment to the participants but did not indicate treatment allocation to the investigator. After the database had been locked and the statistical analysis plan had been finalized, unblinding was conducted via the RTSM system.

### Study procedures

All patients provided written informed consent and entered a 14-day screening period, during which the trial inclusion and exclusion criteria were checked and baseline information was gathered. After screening, patients were randomly assigned to receive either QLQX (0.3-g capsules, four capsules three times daily) or matching placebo (0.3-g capsules, four capsules three times daily). A web-based response system was used to determine treatment assignment. After randomization, eligible patients were evaluated at 1 and 3 months and every other 3 months after that. Patients were free to discontinue treatment for any reason, and the reasons for withdrawal were recorded in the case report form. Dose reduction (to 2−3 capsules three times daily of QLQX or placebo) or temporary discontinuation was permitted in cases of adverse complications, with a subsequent increase in dose or resumption of treatment, if possible.

The recruitment period lasted for 24 months. The shortest follow-up period was 12 months, and the median duration of follow-up duration was 18.3 months (IQR, 14.3 to 23.5 months).

### Study outcomes

The primary outcome was the time to the first major adverse cardiovascular event, which was defined as either cardiovascular death or first HHF occurring within the follow-up period. An episode of worsening heart failure was either an unplanned hospitalization with objective evidence of an exacerbation of heart failure (as determined through clinical examination and/or laboratory evidence) or an urgent visit resulting in intravenous therapy for heart failure.

Secondary outcome measures included all-cause mortality, secondary endpoint events (treatment termination due to worsening heart failure, successful resuscitation after cardiac arrest, malignant arrhythmia, nonfatal stroke), cardiovascular death and first HHF in patients with ischemic etiology within the follow-up period and serum NT-proBNP levels at 3 months. All outcomes were adjudicated by the Clinical Event Adjudication Committee, which was unaware of trial group assignment, according to prespecified criteria. The definitions of endpoints and adverse events, as well as the procedures applicable to the Clinical Event Adjudication Committee, are described in the supplementary protocol (Supplementary Information, ‘Protocol’).

The prespecified safety analyses included serious adverse events, adverse events associated with discontinuation of a trial drug, adverse events of interest and laboratory findings of note. Data on other adverse events were routinely collected at each follow-up examination or unexpected visit.

### Statistical analysis

The sample size was determined to provide adequate power to assess the outcomes of cardiovascular death and first HHF. We estimated that the annual rate of the composite endpoint would be 25% in patients receiving placebo and 20% in the QLQX group within the 12−36 months of follow-up.

The error was controlled at an overall two-sided *α* level of 0.05 and *β* level of 0.20 with two scheduled internal interim efficacy analyses, and the statistical stopping guideline for compelling benefit required a one-sided nominal *P* value <0.0001 at the first analysis and <0.00605 at the second analysis in favor of QLQX as the primary endpoint. On the basis of these calculations, it was estimated that approximately 3,080 patients would need to be included to provide the required number of 620 expected composite primary end point events, with an anticipated recruitment period of 24 months.

Only one interim analysis was performed when two-thirds of the primary endpoint events had occurred due to the pandemic. Termination for futility was set to be triggered if the conditional power was below 20%, whereas termination for efficacy would be triggered if the interim analysis yielded a *P* value of <0.00605. The significance of the two-sided *α* level for the multiple comparisons across the primary outcomes was 0.04628, with one interim efficacy analysis taken into account (with consumed two-sided *α*_1_ = 0.012096).

Full analysis set (FAS) refers to the dataset that includes all patients who underwent randomization and received at least one dose of the study drug, with minimal and reasonable exclusion of individuals adhering to the intention-to-treat principle. The PPS was a subset of the FAS in which individuals had optimal adherence to the protocol with drug administration of at least 80% and no significant protocol violations. Protocol violation refers to (1) important violations of inclusion criteria; (2) individuals who did not receive the study drug; and (3) lack of post-randomization observation data. The safety set (SS) included all randomized participants who received at least one treatment and underwent safety evaluations.

Baseline characteristics are summarized as the mean + s.d., median and IQR or percentages. The baseline covariates were subject to a small amount of missing data that were assumed to be randomly missing. The comparability of characteristics between study groups was assessed using Student's *t*-test for continuous variables and the Wilcoxon test for categorical variables. Intergroup comparisons were made using the chi-square test for categorical variables and Student's *t*-test or the Wilcoxon test for continuous variables. Intragroup comparisons were conducted using the Wilcoxon signed-rank test for categorical variables. Time-to-event data were evaluated using Kaplan–Meier estimates and Cox proportional-hazards models, with the trial site as a random effect, to estimate the HR and 95% CI for the primary and secondary outcomes. Cumulative primary endpoint curves were constructed using Kaplan−Meier methods, and differences between curves were tested using the log-rank method.

We employed post hoc analysis using the Fine−Gray and cause-specific hazards competing risk-adjusted model, thereby enhancing the robustness and reliability of our findings. The model accounted for the competing risk of noncardiovascular mortality. We also assessed the consistency of the treatment effect among the prespecified subgroup variables of interest, which was verified using the Instrument for assessing the Credibility of Effect Modification Analyses (ICEMAN)^33^. Sensitivity analyses were not prespecified and were conducted to assess the robustness of the primary findings by (1) censoring at follow-up of 12-months; (2) complete case analysis; and (3) using the PPS outcomes.

Statistical analyses were conducted using SAS v.9.4 software. Two-sided *P* < 0.05 was considered statistically significant.

### Reporting summary

Further information on research design is available in the Nature Portfolio Reporting Summary linked to this article.

## Online content

Any methods, additional references, Nature Portfolio reporting summaries, source data, extended data, supplementary information, acknowledgements, peer review information; details of author contributions and competing interests; and statements of data and code availability are available at 10.1038/s41591-024-03169-2.

### Supplementary information

Supplementary Information Appendix for the Committee Information, Protocol and SAP and the QLQX CMC report.

Reporting Summary

## Figures and Tables

**Extended Data Table 1:**
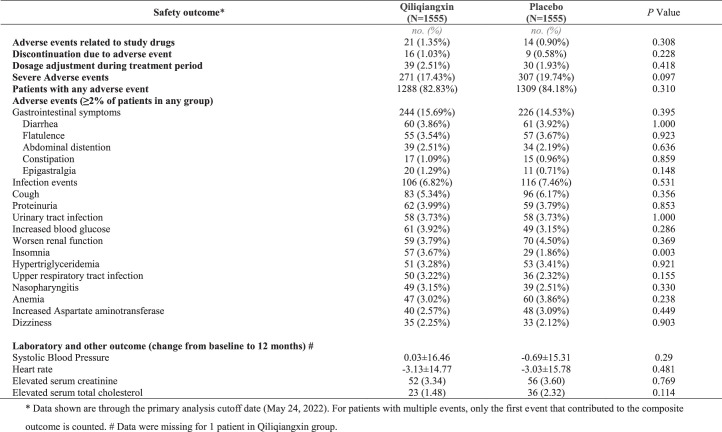
Safety Outcome

**Extended Data Table 2:**
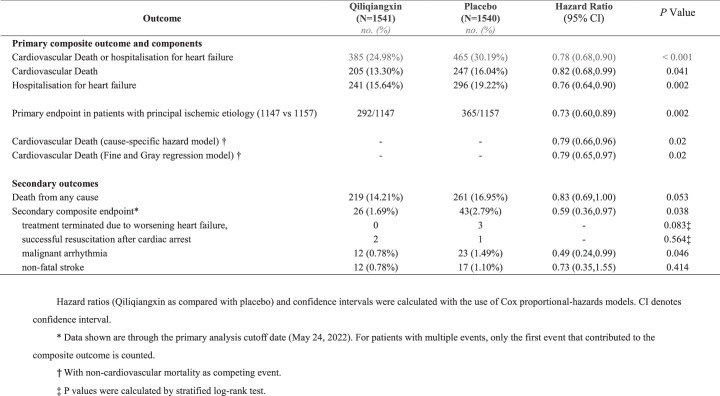
Primary and Secondary Outcomes in Per Protocol Set

**Extended Data Table 3:**
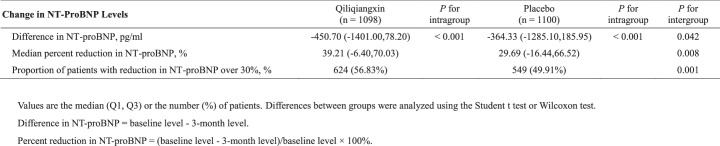
Change in Plasma NT-ProBNP Levels From Baseline to After 3-Month of Follow-Up in Per Protocol Set

**Extended Data Table 4:**
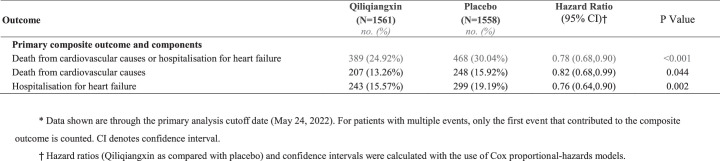
Primary and Secondary Outcomes in Complete Cases after Randomization

**Extended Data Table 5:**
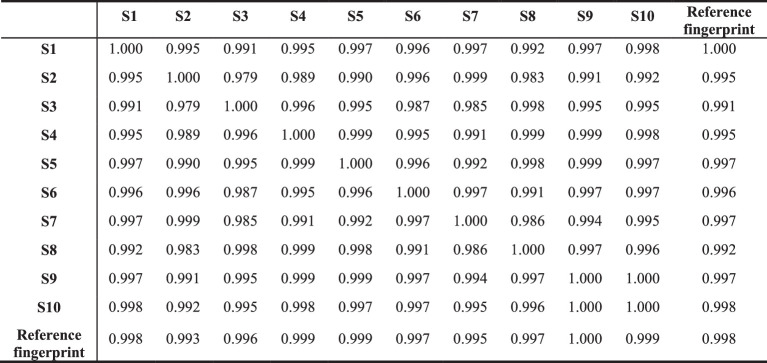
The similarity of UPLC fingerprints from the 10 batches Qiliqiangxin capsules and Samples

## Data Availability

The data that support the findings of this study are not publicly available due to restrictions on patient privacy, but are available upon reasonable request for access to the patient-level data from this study. Requests can be submitted via email to the corresponding authors (X. Li (xinli3267@yeah.net) and Z.J. (jzhjiazhenhua@163.com)) with detailed proposals for use of the data. Responses to such requests can be expected within one month. Depending on the data requested, the data request will be reviewed and, if agreed, a signed agreement with the sponsor is required before the data can be accessed.
