# Supplementary material for: The traditional Chinese medicine Qiliqiangxin in heart failure with reduced ejection fraction: a randomized, double-blind, placebo-controlled trial
Source: Nat Med. 2024 Aug 2;30(8):2295–302. doi: 10.1038/s41591-024-03169-2 (PMC11333273; doi:10.1038/s41591-024-03169-2)
Supplement: Supplementary file 1 — Appendix for the Committee Information, Protocol and SAP and the QLQX CMC report. [file 41591_2024_3169_MOESM1_ESM.pdf]

nature medicine

Supplementary information https://doi.org/10.1038/s41591-024-03169-2

The traditional Chinese medicine Qiliqiangxin in heart failure with reduced ejection fraction: a randomized, double-blind, placebo-controlled trial

In the format provided by the authors and unedited

04a_committee & appendix 2

04b_P+SAP_0227 11

04c_QLQX CMC report 451

Complete list of the QUEST Committees and Investigators

- **Executive Committee**

Professor Xinli Li (Chairman, PI of QUEST study), The First Affiliated Hospital With Nanjing Medical University, Nanjing, China.

Professor Zhenhua Jia (Co-chair, PI of Cardiovascular continuum and TCM collateral disease theory research program), State Key Laboratory for Innovation and Transformation of Luobing Theory, Hebei clinical research center of cardiovascular disease of traditional Chinese Medicine, Shijiazhuang, Hebei, China.

Professor Haifeng Zhang (Co-chair), The First Affiliated Hospital With Nanjing Medical University, Nanjing, China.

Professor Jiangang Shen (Co-chair, PI of QUEST TCM syndrome study), School of Chinese Medicine, The University of Hong Kong, Hong Kong SAR, China.

- **Academic Counselling Committee**

Academician Runlin Gao, Fuwai Hospital, Chinese Academy of Medical Sciences and Peking Union Medical College, Beijing, China.

Academician Boli Zhang, Tianjin University of Traditional Chinese Medicine, Tianjin, China.

Academician Yun Zhang, Shandong University, Jinan, Shandong, China.

Academician Junbo Ge, Zhongshan Hospital, Fudan University, Shanghai, China

Academician Yaling Han, General Hospital of Northern Theater Command, Shenyang, China.

Professor Congxin Huang, Renmin Hospital of Wuhan University, Wuhan, China.

Professor Anthony Rosenzweig, Institute for Heart and Brain Health, University of Michigan Medical Center, Ann Arbor, USA.

- **Data Safety Monitoring Committee (DSMC)**

Professor Feng Chen, School of Public Health, Nanjing Medical University, Nanjing, China.

Professor Jun Zhu, Fuwai Hospital, Chinese Academy of Medical Sciences and Peking Union Medical College, Beijing, China.

Professor Qizhu Tang, Renmin Hospital of Wuhan University, Wuhan, China.

Professor Changsheng Ma, Beijing Anzhen Hospital, Capital Medical University, Beijing, China.

Professor Naisheng Cai, Zhongshan Hospital, Fudan University, Shanghai, China.

- **Clinical Event Adjudication Committee**

Professor Xinchun Yang, Beijing ChaoYang Hospital, Capital Medical University, Beijing, China.
Professor Lei Song, Fuwai Hospital, Chinese Academy of Medical Sciences and Peking Union Medical College, Beijing, China.
Professor Naisheng Cai, Zhongshan Hospital, Fudan University, Shanghai, China
Professor Haiming Shi, Huashan Hospital, Fudan University, Shanghai, China.
Professor Wenling Zhu, Peking Union Medical College Hospital, Peking Union Medical College and Chinese Academy of Medical Sciences, Beijing, China.

Appendix: DSMC Statistical Decision Rules

This study conducts one interim efficacy analysis and utilizes predefined alpha spending functions to calculate the nominal significance levels for both the interim and final analyses. It evaluates efficacy based on the observed treatment effects and assesses whether to stop the study early for futility or efficacy.

(1) Early Stopping for Futility:

If the futility analysis shows that the conditional power (assuming the observed treatment effect size at the interim analysis is the true effect size) is less than 20%, and other efficacy indicators similarly suggest minimal effect, then the study will be terminated early for futility due to the low probability of trial success and insufficient clinical significance of the treatment effect.

(2) Early Stopping for Efficacy:

To control the overall Type I error rate, the Lan-DeMets and O'Brien-Fleming alpha spending function method is used, with a nominal one-sided alpha level of $\alpha 1 = 0.006048$ for this interim analysis. If the interim analysis yields $P1 < 0.00605$, the null hypothesis may be rejected early for efficacy. Otherwise, the trial will continue. If the trial proceeds to completion, the final analysis will use a nominal one-sided alpha level of $\alpha 2 = 0.02314$. If $P2 < 0.02314$ at the final analysis, the difference between the two groups can be considered statistically significant.

List of participating center and investigators

No.	Participating Center	Participating Center (Chinese)	Principle Investigator	Principle Investigator (Chinese)
1	The First Affiliated Hospital With Nanjing Medical University	南京医科大学第一附属医院	Xinli Li; Haifeng Zhang; Iokfai Cheang	李新立；张海锋；郑旭辉
2	Hebei Yiling Hospital	河北以岭医院	Guoqiang Yuan	袁国强
3	Binzhou People's Hospital	滨州市人民医院	Jiayu Cui	崔家玉
4	Binzhou Central Hospital	滨州市中心医院	Lijun Meng	孟立军
5	Xinxiang Central Hospital	新乡市中心医院	ZhiFang Wang	王志方
6	Baoji Second People's Hospital	宝鸡市第二人民医院	Haifeng Ding	丁海峰
7	Medicine Second Afiliated Hospital Guizhou University of Traditional Chinese Medicine	贵州中医药大学第二附属医院	Tao Xu	许滔
8	Liaocheng People's Hospital	聊城市人民医院	Qinghua Xu; Peng Sun	胥庆华；孙鹏
9	First Affiliated Hospital of Xinjiang Medical University	新疆医科大学第一附属医院	Ailiman Mahemuti	艾力曼·马合木提
10	Weihai Municipal Hospital	威海市立医院	Zhitao Teng	滕志涛
11	Shandong Provincial Qianfoshan Hospital	山东省千佛山医院	Enkui Hao	郝恩魁
12	Jiangmen Central Hospital	江门市中心医院	Gaoxing Zhang	张高星
13	Luoyang Central Hospital	洛阳市中心医院	Songsen Li	李松森
14	Yunnan University Affiliated Hospital, The Second Peoples Hospital of Yunnan	云南省第二人民医院	Minghua Han	韩明华
15	Yan'An Hospital of Kunming City	昆明市延安医院	Qi Yin	尹淇
16	Jiangxi Provincial Hospital	江西省人民医院	Guotai Shen	盛国太
17	The First People's Hospital of HeFei	合肥市第一人民医院	Xiao-hong Zhang	张晓红
18	Beijing Anzhen Hospital,Capital Medical University	首都医科大学附属北京安贞医院	Jianzeng Dong	董建增
19	The First Hospital of Harbin Medical University	哈尔滨医科大学附属第一医院	Lu Fu	富路
20	Qingdao Municipal Hospital	青岛市立医院	Yibing Shao	邵一兵
21	Affiliated Hospital of Gansu University of Chinese Medicine	甘肃中医药大学附属医院	Yingdong Li	李应东
22	The First Affiliated Hospital of Wannan Medical College	皖南医学院弋矶山医院	Yueyun Chen	陈月云
23	The First Affiliated Hospital of Chongqing Medical University	重庆医科大学附属第一医院	Suxin Luo	罗素新

24	Peking University People's Hospital	北京大学人民医院	Hong Chen	陈红
25	Shandong Provincial Hospital	山东省立医院	Haitao Yuan	苑海涛
26	Wu'an First People's Hospital	武安市第一人民医院	Xiaofang Wang	王晓芳
27	Beijing Daxing District Hospital of Integrated Chinese and Western Medicine	北京市大兴区中西医结合医院	Wei Song	宋炜
28	The First Affiliated Hospital, Sun Yat-sen University	中山大学附属第一医院	Yu-Gang Dong	董吁钢
29	The Second People's Hospital of Liaocheng	聊城市第二人民医院	Yun Zhang	张云
30	Qiqihar Traditional Chinese Medicine Hospital	齐齐哈尔市中医医院	Junyi Li	李俊毅
31	CR& WISCO General Hospital	华润武钢总医院	Xuexiang Lv	吕学祥
32	Daming People's Hospital	大名县人民医院	Tao Cheng	成涛
33	The First Affiliated Hospital of Medical College of Shihezi University	石河子大学医学院第一附属医院	Li Wang	王丽
34	Queen Mary Hospital	香港大学玛丽医院	Chung Wah David Siu; Chun-Ka Wong	萧颂华；黄浚嘉
35	Weixian Hospital of Traditional Chinese Medicine	魏县中医院	Junhai Zhang	张军海
36	Sichuan Provincial People's Hospital	四川省人民医院	Hong Kong	孔洪
37	Taonan People's Hospital	洮南市人民医院	Chunli Gao	高春丽
38	Affiliated Renhe Hospital of China Three Gorges University	三峡大学附属仁和医院	Jianfeng Lv	吕建峰
39	Shijiazhuang Third Hospital, Hebei Medical University Affiliated Shijianzhuang Third Hospital	石家庄市第三医院	Lijun Wang	王立君
40	The First Affiliated Hospital of Bengbu Medical College	蚌埠医学院第一附属医院	Ningru Zhang	张宁汝
41	The Fourth Hospital of Daqing	大庆市第四医院	Guo-tao Wang	王国涛
42	Affiliated Hospital of Nantong University	南通大学附属医院	Qi Lu	陆齐
43	Laiwu Iron and Steel Group Laikuang Hospital	莱矿医院	Fangchen Lv	吕方晨
44	The Second People's Hospital of Xintai	新泰市第二人民医院	Guoqing Wu	武国庆
45	The Third Affiliated Hospital, Sun Yat-sen University	中山大学附属第三医院	Xiao-Xian Qian	钱孝贤
46	The First Affiliated Hospital of Guangzhou University of Chinese Medicine	广州中医药大学第一附属医院	Rong Li	李荣

47	Baicheng Central Hospital	白城市中心医院	Tiancai Liu	刘天才
48	Nanjing Hospital of T.C.M	南京市中医院	Ning Gu	顾宁
49	Sinopharm Tongmei Genrral Hospital	国药同煤总医院	Chengbao Lei	雷成宝
50	Shiyan Renmin Hospital	十堰市人民医院	Jianing Wang	王家宁
51	Xiuyan Manchu Autonomous County Central Peoples Hospital	岫岩满族自治县中心人民医院	Jianhua Wu	吴建华
52	Handan Central Hospital	邯郸市中心医院	Hongsen Tian	田洪森
53	Zhuzhou 331 Hospital	株洲市三三一医院	Xingze Xie	谢兴泽
54	Beidahuang Group General Hospital	北大荒集团总医院	Liyan Wang	王丽艳
55	Xinjiang Production and Construction Corps First Division Hospital	新疆建设兵团第一师医院	Chunxi Cha	茶春喜
56	Guangyuan Central Hospital	广元市中心医院	Yuanjun He	何元军
57	The First Bethune Hospital of Jilin University	吉林大学第一医院	Jian Sun	孙健
58	Songyuan Central Hospital	松原市中心医院	Lijuan Yuan	苑丽娟
59	The People's Hospital of Xin Tai City	新泰市人民医院	Hongyan Zhang	张鸿雁
60	Union Hospital Tongji Medical College of Huazhong University of Science and Technology	华中科技大学附属协和医院	Jing Yuan	袁璟
61	The First Hospital of Hunan University of Chinese Medicine	湖南中医药大学第一附属医院	Jianhe Liu	刘建和
62	People's Hospital of Bortala Mongolian Autonomous Prefecture	博尔塔拉蒙古自治州人民医院	Bayin Bate	巴音巴特
63	Zibo Municipal Hospital	临淄区人民医院	Guangzhong Zheng	郑贯中
64	Zhuzhou Kind Cardiovascular Disease Hospital	株洲恺德心血管病医院	Chengqian Ren	任承潜
65	Ankang Central Hospital	安康市中心医院	Hui Ren	任晖
66	The Second Affiliated Hospital of Nnachang University	南昌大学第二附属医院	Yanqing Wu	吴延庆
67	The First Affiliated Hospital of Ustc AnHui Provincial Hospital	安徽省立医院	Ji Yan	严激
68	The Second Hospital of Hebei Medical University	河北医科大学第二医院	Xiugong Zu	祖秀光
69	The People's Hospital of Hebi	鹤壁市人民医院	Yunfu Li	李云富
70	Luoyang Traditional Chinese Medicine Hospital	洛阳市中医院	Yanling Sun	孙艳玲
71	Jining No.1 People's Hospital	济宁市第一人民医院	Jundong Miao	苗俊东
72	Shijiazhuang People's Hospital	石家庄市人民医院	Hao Chen	陈浩

73	The Affiliated Hospital of XuZhou Medical University	徐州医科大学附属医院	Zhirong Wang	王志荣
74	The Second People's Hospital of Bengbu	蚌埠市第二人民医院	Zhuo Shang	商卓
75	Yantai Hospital of Traditional Chinese Medicine	烟台市中医医院	Jun Ma	马俊
76	Beijing Pinggu Hospital	北京市平谷区医院	Xiaohonh Gao	高筱红
77	Kunming Municipal Hospital of Traditional Chinese Medicine	昆明市中医医院	Yanrong Jin	金艳蓉
78	Yushu People‘s Hospital	榆树市人民医院	Yongge Sun	孙永阁
79	China-Japan Friendship Hospital	中日友好医院	Xianlun Li	李宪伦
80	Dunhua Municipal Hospital in Jilin Province	敦化市医院	Fanju Meng	孟繁菊
81	Guangdong Provincial Hospital of Traditional Chinese Medicine	广东省中医院	Weihui Lv	吕渭辉
82	Sichuan Mianyang 404 Hospital	绵阳 404 医院	Decai Li	李德才
83	Zhenjiang First People's Hospital	镇江市第一人民医院	Guohui Zhang	张国辉
84	The Second Affiliated Hospital of Shandong First Medical University	山东第一医科大学第二附属医院	Bin Ge	葛斌
85	Zouping People's Hospital	邹平市人民医院	Bing Wang	王冰
86	Dongying Shengli Hospital	东营胜利医院	Qingrui Kong	孔庆瑞
87	Guangdong Hospital of Integrated Traditional Chinese and Western Medicine	广东省中西医结合医院	Peisheng Dai	戴培胜
88	Workers' Hospital of Handan Iron and Steel Group Co.	邯郸钢铁集团有限责任公司职工医院	Yongshun Xu	许永顺
89	The Second Affiliated Hospital of Shaanxi Medical University of Chinese Medicine	陕西中医药大学第二附属医院	Jing Dong	董静
90	Chengdu First People's Hospital	成都市第一人民医院	Jun Zhang	张俊
91	Xuzhou Central Hospital	徐州市中心医院	Bing Han	韩冰
92	The Third Affiliated Hospital of Sun Yat-Sen University, Yuedong Hospital	中山大学附属第三医院粤东医院	Xinghong Xie	谢幸洪
93	Weifang Hospital of Traditional Chinese Medicine	潍坊市中医院	Kegang Ji	嵇克刚
94	Weihai Central Hospital	威海市中心医院	JianHua Zhou	周建华
95	Chenzhou Third people's Hospital	郴州市第三人民医院	Yijun Luo	罗怡军
96	Affiliated Hospital of North Sichuan Medical College	川北医学院附属医院	Houxiang Hu	胡厚祥

97	Fuwai Central China Cardiovascular Hospital	阜外华中心血管病医院	Chuanyu Gao	高传玉
98	Kaifeng Central Hospital	开封市中心医院	Lei Qin	秦雷
99	The First Affiliated Hospital of Hebei North University	河北北方学院附属第一医院	Fangjiang Li	李方江
100	Affiliated Nanhua Hospital, University of South China	南华大学附属南华医院	Xiaofeng Ma	马小峰
101	Pidu District People's Hospital, Chengdu	成都市郫都区人民医院	Chaoping Yu	余朝萍
102	Sunsimiao Hospital of BUCM	北京中医药大学孙思邈医院	Qian Ren	任骞
103	Ankang Hospital of Traditional Chinese Medicine	安康市中医医院	Chuang Lou	娄闯
104	Yanjin County People's Hospital	延津县人民医院	Shifeng Ren	任世峰
105	Renji Hospital, School of Medicine, Shanghai Jiaotong University	上海交通大学医学院附属仁济医院	Meng Jiang	姜萌
106	Shanxian Central Hospital	单县中心医院	Peitang Wu	吴培堂
107	Xianyang Hospital of Yan'an University	延安大学咸阳医院	Xianli Wang	王显利
108	Tongji Hospital Tongji Medical College of Huazhong University of Science and Technology	华中科技大学同济医学院附属同济医院	Chunxia Zhao	赵春霞
109	Hengyang First People's Hospital	衡阳市第一人民医院	Yong He	何勇
110	Beijing Huaxin Hospital	北京华信医院	Zhiqin Guo	郭志琴
111	Muping Zhongyi Yiyuan	烟台市牟平区中医医院	Aiqing Lin	林爱庆
112	Renmin Hospital of Wuhan University	武汉大学人民医院	Difei Shen	沈涤非
113	Dongying People’s Hospital	东营市人民医院	Xichang Dong	董希昌
114	The First Hospital of China Medical University	中国医科大学附属第一医院	Zixin Zhang	张子新
115	Anshan Hospital, The First Affiliated Hospital of China Medical University	中国医科大学鞍山医院	Bin Liu	刘斌
116	Affiliated Hospital of Weifang Medical University	潍坊医学院附属医院	Jianli Liu	刘建立
117	Xiangtan Central Hospital	湘潭市中心医院	Lei Wang	王磊
118	People's Hospital of Xiajin	夏津县人民医院	Yong Li	李勇
119	Affiliated Hospital of YouJiang Medical University for Nationality	右江民族医学院附属医院	Xingshou Pan	潘兴寿

120	Affiliated Hospital of Shaanxi University of Chinese Medicine	陕西中医药大学附属医院	Mingjun Zhao	赵明君
121	Yantai Yeda Hospital	烟台业达医院	Jinpeng Yao	姚金鹏
122	Linyi Traditional Chinese Medicine Hospital	临沂市中医医院	Xinyu Liu	刘新宇
123	Peking University Third Hospital Yanqing Hospital	北京市延庆区医院	Lixin Wang	王立新
124	General Hospital of Fushun Mining Bureau of Liaoning Health Industry Group	辽宁省健康产业集团抚矿总医院	Fengjun Qi	戚凤君
125	The People's Hospital of Pingyi County	平邑县人民医院	Kaiyan Zhang	张开颜
126	The First Affiliated Hospital of Xi'an Jiaotong University	西安交通大学医学院第一附属医院	Ling Bai	白玲
127	Qiqihar Jianhua Hospital	齐齐哈尔建华医院	Bo Jiang	姜波
128	The First Affiliated Hospital of Soochow University	苏州大学附属第一医院	Tan Chen; Yafeng Zhou	陈弹；周亚峰
129	Huludao Central Hospital	葫芦岛市中心医院	Lijuan Chang	常丽娟
130	Meizhou Hospital of TCM	梅州市中医院	Zhihua Huang	黄志华
131	Benxi Central Hospital	本溪市中心医院	Aijun Liu	刘爱军
132	The First Affiliated Hospital of Dalian Medical University	大连医科大学附属第一医院	Ying Liu	刘莹
133	Xinqu Distict People's Hospital of Chengdu	成都市新都区人民医院	Shiwu Deng	邓诗武

Table of Contents:

Page 2 —— English protocol version V1.3 (in English, Corresponding to the amended protocol version V1.3), translation date 18-Jan-2023.
Page 37 —— Amended protocol version V1.3 (in Chinese), dated 2-Nov-2018.
Page 71 —— English protocol version V1.2 (Corresponding to the amended protocol version V1.2), translation date 9-Oct-2018.
Page 109 —— Amended protocol version V1.2 (in Chinese), dated 10-Sept-2018
Page 143 —— Original protocol version V1.0 (in Chinese), dated 10-July-2018.
Page 179—— English QUEST statistical analysis plan V1.1, dated 15-Jan-2023.
Page 251 —— QUEST statistical analysis plan V1.1, dated 10-Jan-2023.
Page 318 —— English QUEST statistical analysis plan V1.0, dated 30-Nov-2022.
Page 382 —— QUEST statistical analysis plan V1.0, dated 21-Nov-2022.

English translation version of protocol version 1.3

1. Corresponding to the amended protocol version V1.3 (in Chinese), translation date 18-Jan-2023.
2. "Standard heart failure treatment" has been updated according to the "2018 Chinese Diagnosis and Treatment Guidelines for Heart Failure" for eligible patients.
3. The clinical laboratory indicators observed in the "7.2. Safety Indicators" section have been revised and included "blood routine (hemoglobin, red blood cells, white blood cells, platelets), urine routine (urinary protein, white blood cells, red blood cells), and serum biochemistry (urea nitrogen, creatinine, alanine aminotransferase, fasting blood glucose, potassium, sodium, chloride, total cholesterol, triglycerides)."
4. The description of the severity grading for adverse events in "10.2. Adverse Event Intensity Determination Criteria" has been revised.
5. The criteria for determining the relationship between adverse events and the investigational drug in "10.3. Criteria for Determining the Relationship Between Adverse Events and Investigational Drug" has been revised.
6. "Appendix 2: Echocardiography Detection Indicators and Standard Operation Procedures" has been added to standardize the process and parameter measurements for cardiac ultrasound examinations.
7. The original "Appendix 2: NT-proBNP Examination Blood Sampling, Preservation, and Transportation Procedures" has been deleted, and the monitoring of NT-proBNP will be conducted at each individual center. Before starting the study at a center, investigators must ensure that the center has a standardized process for NT-proBNP detection.
8. Contents were also amened with the actual date of the trials progress.
9. Language and syntax errors revision form the last translation version.
10. This English version protocol was translated and submitted for peer review.
11. Contents are on the basis of and translated from Chinese. If the English and Chinese contents are inconsistent, Chinese version shall prevail.

The content of this study protocol is confidential and is intended for use by clinical researchers. The material is the property of the sponsor or its sub-units and may not be copied or distributed to persons unrelated to the study.

Contents are on the basis of and translated from Chinese. If the English and Chinese contents are inconsistent, Chinese version shall prevail.

Qiliqiangxin in Heart FailUre: AssESsment of Reduction in MorTality

(QUEST STUDY)

Study Protocol

Protocol No.: SP-YFC-05-QUEST

Version No.: V1.3

Version date: Jan 18, 2023

ChiCTR No.: ChiCTR1900021929

The First Affiliated Hospital of Nanjing Medical University

Principal investigator: Professor LI Xinli

Duration of study: May 2019 to May 2023

RESPONSIBLE PARTIES

Principal Investigator of the protocol

Research unit	Principal investigator
The First Affiliated Hospital of Nanjing Medical University	LI Xinli Signature:________________ Date:________________

Statistical Analysis

Unit	Contact
Peking University Clinical Research Institute	YAO Chen

Drug Manufacturer

Unit	Contact	Tel	Email
Shijiazhuang Yiling Pharmaceutical Co., Ltd	HAN Shuolong	13582167153	hanshuolong@yiling.cn

Academic Counselling Committee

China: Academician GAO Runlin, Academician ZHANG Boli, Academician ZHANG Yun, Academician GE Junbo, Academician HAN Yaling, Professor HUANG Congxin

USA: Professor Anthony Rosenzweig

Data Safety Monitoring Committee

Chair: Professor CHEN Feng

Professor ZHU Jun, Professor TANG Qizhu, Professor MA Changsheng, Professor CAI Naisheng

Clinical Event Adjudication Committee

Chair: Professor YANG Xinchun

Professor SONG Lei, Professor CAI Naisheng, Professor SHI Haiming, Professor ZHU Wenling

Abbreviations

ACEI	Angiotensin converting enzyme inhibitor
ADR	Suspected adverse drug reaction
AEs	Adverse events
AHA	American Heart Association
AMI	Acute myocardial infarction
ARB	Angiotensin receptor blocker
ARNI	Angiotensin receptor neprilysin inhibitor
BNP	B-type natriuretic peptide
CEA	Clinical Event Adjudication
CGRP	Calcitonin gene-related peptide
CHF	Chronic heart failure
CRA	Clinical research auditor
CRC	Clinical research coordinator
CRF	Case report form
CV	Cardiovascular
DSMC	Data and Safety Monitoring Committee
DVP	Data verification plan
ECG	Electrocardiogram
EF	Ejection fraction
EOS	Final visit
ESC	European Society of Cardiology
ET	Endothelin
FAS	Full analysis set
HF	Heart failure
IP	Investigational Product
LVOT	Left ventricular outflow
MedDRA	ICH International Medical Dictionary
MI	Myocardial infarction
NO	Nitric oxide
NT-proBNP	N-terminal pro brain natriuretic peptide
NYHA	New York Heart Association
PPS	Per protocol set
RAAS	Renin-angiotensin-aldosterone system
SAE	Serious adverse event
SNS	Sympathetic nervous system
SAS	Safety analysis set
TVI	Time velocity integral
UNS	Unplanned visit

PROTOCOL SUMMARY

**Objectives**	Using evidence-based medicine research methods, with cardiovascular mortality and hospital readmission rate for worsening heart failure as the main research endpoints, further elucidating the clinical efficacy and safety of long-term use of Qiliqiangxin capsules (QLQX), clarifying the characteristics of efficacy and the suitable population, to provide high-quality clinical evidence for optimizing HF therapy.
**Study design**	Randomized, double-blind, placebo-controlled, multicenter clinical trial
**Inclusion and Exclusion Criteria**	**Inclusion criteria:**<br>1) Signed informed consent;<br>2) Aged ≥18 years at the time of consent;<br>3) Established documented diagnosis of heart failure for at least three months ago according to "Chinese Heart Failure Diagnosis and Treatment Guideline" issued by the Chinese Medical Association Cardiovascular Branch;<br>4) Left ventricular ejection fraction (LVEF) ≤40% (echocardiogram, radionuclide, ventriculogram, contrast angiography or cardiac MRI);<br>5) NYHA cardiac functional grading II to III, with stable clinical symptoms; or those diagnosed as grade IV within 2 weeks before enrollment;<br>6) Serum NT-proBNP ≥ 450pg/ml;<br>7) Those who have received standardized baseline treatment regimens without doses adjusted and given intravenously for at least two weeks prior to enrollment;<br>Standardized drug treatment includes angiotensin-converting enzyme inhibitor (ACEI) or angiotensin receptor blocker (ARB) or angiotensin receptor neprilysin inhibitor (ARNI), beta blocker, and aldosterone receptor antagonist (the optimal therapeutic dose should be achieved, except for contraindications or intolerance).<br>**Exclusion criteria:**<br>1) Patients should not enter the study if any of the following exclusion criteria are fulfilled<br>2) Heart failure caused by valvular disease, congenital heart disease, pericardial disease, arrhythmia or non-cardiaogenic disease, or caused by vital organ failure (such as renal, hepatic failure, etc.); and right heart failure caused by pulmonary or other definite causes; and acute heart failure;<br>3) Coronary revascularization (percutaneous coronary intervention [PCI] or coronary artery bypass grafting [CABG]) or cardiac synchronization therapy planned to undergo after randomization, or had received cardiac resynchronization therapy prior to enrolment;<br>4) Any condition outside the CV diseases such as but not limited to malignant tumor, severe mental illness, hematopoietic diseases, neuroendocrine system disease, liver transaminase and alkaline phosphatase ≥ 3 x upper limit of normal (ULN), abnormal renal function serum creatinine > 2 mg/dl (176.82 umol/L), potassium >5.5mmol/L;<br>5) Patient with left ventricular outflow tract obstruction, myocarditis, aortic aneurysm, aortic dissection, or obvious hemodynamic changes caused by unrepaired valve;<br>6) Cardiogenic shock, uncontrollable malignant arrhythmia, sinus or atrioventricular block at second degree type II or above without pacemaker treatment, progressive unstable angina pectoris or acute myocardial infarction;<br>7) Uncontrolled hypertension systolic blood pressure (SBP) ≥ 180mmHg and/or diastolic blood pressure (DBP) ≥ 110mmHg; or SBP < 90mmHg and/or DBP <60mmHg;

<table>
<tr><td></td><td colspan="2">8) Participation in another clinical study with an IP during the last month prior to enrolment;
9) Women of child-bearing potential (i.e., those who are not chemically or surgically sterilized or who are not post-menopausal) who are not willing to use a medically accepted method of contraception that is considered reliable in the judgment of the investigator, from the time of signing the informed consent throughout the study and 4 weeks thereafter, OR women who have a positive pregnancy test at enrolment or randomisation OR women who are breast-feeding;
10) Allergic constitution; known to be allergic to research drug;
11) Inability of the patient, in the opinion of the investigator, to understand and/or comply with study medications, procedures or any conditions may render the patient unable to complete the study.</td></tr>
<tr><td rowspan="2">Outcome Measures</td><td>Primary endpoints</td><td>The major adverse composite endpoint events included cardiovascular death and hospitalization for heart failure</td></tr>
<tr><td>Secondary endpoints</td><td>1. All-cause mortality
2. Secondary endpoint events (given up treatment due to worsening heart failure, successful resuscitation after cardiac arrest, malignant arrhythmia, non-fatal stroke)
3. The incidence of cardiovascular death and hospitalization for heart failure in patients with ischemic heart disease
4. Serum NT-proBNP decrease rate</td></tr>
<tr><td>Safety Outcome Measures</td><td colspan="2">To evaluate the safety and tolerability of QLQX in this patient population: changes in clinical parameters (complete blood count/ biochemistr, ECG, physical examination), and any forms of adverse events (AEs)</td></tr>
<tr><td>Sample size and Statistical methods</td><td colspan="2">The random allocation ratio between the QLQX group and control group is 1:1. The sample size is calculated based on the expected composite endpoint events of 620 composite endpoint events. Estimated incidence rate of composite endpoint events in the control group within the 36-month follow-up period is 25%. The trial lasts for approximately 36 months with an estimated recruitment period of 24 months, it is estimated that 3080 participants (1540 per group) need to be enrolled to obtain 620 endpoint events.</td></tr>
<tr><td>Dosage and mode of administration</td><td colspan="2">Study group: Standardized treatment of chronic heart failure + Qiliqiangxin capsules, 4 capsules, 3 times/day, administered orally
Control group: Standardized treatment of chronic heart failure + Placebo capsules, 4 capsules, 3 times/day, administered orally</td></tr>
<tr><td>Duration of treatment</td><td colspan="2">The patients were selected based on the criteria. The planned enrollment period was 2 years, and they took medicine for at least 1 year.</td></tr>
<tr><td>Statistical unit</td><td colspan="2">Peking University Clinical Research Institute</td></tr>
<tr><td>Expected progress</td><td colspan="2">May 2019 to May 2023</td></tr>
</table>

Contents

1. Study Title ... 8
2. Study Objective ... 8
3. Study background and rationale ... 8
4. Study Design ... 11
4.1. Randomization and Enrollment (Day 0 – 24th month) ... 11
4.2. Control group and Sample size ... 11
5. Rationale for Study Population ... 12
5.1. Inclusion Criteria ... 12
5.2. Exclusion Criteria ... 12
5.3. Discontinuation of investigational product (IP) ... 13
5.4. Withdrawal ... 13
5.5. Discontinuation of the study ... 14
6. Treatment ... 14
6.1 Investigational products (IP) ... 14
6.1.1. Products information ... 14
6.1.2. Package and label ... 14
6.1.3. Storage ... 15
6.1.4. Distribution and Recollection ... 15
6.1.5. Accountability ... 15
6.2. Study and Follow-up Plan ... 15
6.2.1. Study Procedure ... 15
6.2.2. Standardized heart failure treatment ... 16
6.2.3. Concomitant medications and other treatments ... 16
6.2.4. Treatment period ... 17
6.3. Evaluation on compliance ... 17
6.4 Adverse drug reaction ... 17
7. Outcome Measures for Analyses ... 17
7.1 Clinical observation endpoints ... 17
7.1.1. Primary outcome measure (efficacy) ... 17
7.1.2. Secondary outcome measures ... 17
7.2. Safety outcome measure: ... 17
8. Course of Study ... 17
9. Efficacy Assessments and Clinical Event Adjudication (CEA) Committee ... 19
9.1. Definitions of endpoint events ... 19
9.2. Endpoint reporting overview ... 20
10. Safety Assessment ... 21
10.1. Definition of Adverse Event： ... 21
10.2. Grading of Adverse events: ... 21
10.3. IP related adverse events of interest ... 21
10.4. Adjudication of severe adverse events ... 22
10.4.1. Definition of severe adverse events ... 22
10.4.2. Definition on specific severe adverse events ... 22
10.5. Recording of adverse events and follow-up ... 23

10.6. Adverse events based on examinations 23
11. Blinding and Unblinding 24
11.1. Random grouping of subjects 24
11.2. Unblinding and emergency unblinding protocol 24
11.3. Unblinding provisions 24
11.4. Screening number 25
12. Statistical Analysis 25
12.1. Definitions of analysis sets 25
12.2. Statistics methods 25
12.2.1. Enrollment and completion 26
12.2.2. Compliance analysis: 26
12.2.3. Efficacy analysis: 26
12.2.4. Safety analysis: 27
12.3. Interim analysis 27
13. Data Management 27
13.1. Database Design 27
13.2. Data entry 27
13.3. Data questioning management 27
13.4. Medical coding 28
13.5. Data audit 28
13.6. Database locking 28
14. Quality Control 28
15. Ethical conduct of the study 30
16. Study timetable and end of study 30
17. Data Archiving 30
18. Clinical Summary 30
References 31
1 Appendix 1: New York Heart Association (NYHA) Functional Classification 32
2 Appendix 2: Echocardiographic Testing Index and Standard Operating Procedures 32
3 Appendix 3: Endpoint event report process 34

1. Study Title

Qiliqiangxin in Heart FailUre：AssESsment of Reduction in MorTality (QUEST study)

2. Study Objective

Using evidence-based medicine research methods, with cardiovascular mortality and hospital readmission rate for worsening heart failure as the main research endpoints, further elucidating the clinical efficacy and safety of long-term use of Qiliqiangxin capsules (QLQX), clarifying the characteristics of efficacy and the suitable population, to provide high-quality clinical evidence for optimizing HF therapy.

3. Study background and rationale

Cardiovascular disease is one of the major causes of death globally, seriously threatening human life and health [1, 2]. Chronic heart failure (CHF) is a series of clinical syndromes caused by pump failure, reduced ejection fraction, circulatory congestion and/or a series of neurohormonal changes on the basis of structural and/or functional abnormality [3]. As a serious stage of various heart diseases, epidemiological studies showed that the number of global heart failure patients has reached 22.5 million, and the 5-year survival rate is similar to malignant tumors. With the changes of epidemiology and the development of social economy, the epidemiological characteristics of heart failure in developing countries are becoming similar with those in developed countries. For instance, coronary heart disease is becoming the main cause of heart failure in China [4, 5].

In recent years, study by the European Society of Cardiology (ESC) from 51 countries has found that there are estimated at least 15 million heart failure patients in 1 billion population. In 2007, the American Heart Association (AHA) reported that the number of heart failure patients has exceeded 5 million in the United States, and is increasing at a rate of 550,000/year [6]. The incidence of HF in Japan has similar geographical location and ethnic group characteristics as China, which demonstrating a similar epidemiology to Europe and the United States. Previous study in 2003 by GU Dongfeng *et al.* in China randomly surveyed 15,518 adults (aged 35-74 years old) in respective 5 provinces and cities in the south and north of China, and found that the estimated prevalence of heart failure in China was 0.9%, including 0.7% for men and 1.0% for women [7]. The overall incidence and prevalence rate are also gradually increasing worldwide.

Due to the economic improvement, diet change, and also the extending of human life, the incidence of heart failure increases with ageing [8]. Also, with the development of modern medicine, the mortality rate of patients with heart failure has gradually decreased, but remains in a relatively high level. The 1-year mortality rate of patients with heart failure over 70 years old is significantly higher than the counterpart (22% vs 13.7%) in the United States [9]. The 1-year and 3-year mortality rates of heart failure patients are 11.3% and 29.2% in Japan, respectively [10]. In Europe, the 4-year survival rate is 50%, and 40% of heart failure inpatients is admitted to hospital for treatment or dies within 1 year [11, 12]. Chronic heart failure is still a major problem that seriously threatens to human life and quality of life, which in need to be resolved.

In the past 20 years, the concept of medication treatment for heart failure has profoundly changed, from the perspective of improving hemodynamics to the point of biological adjustment. The treatment perspective of modern treatment is to improve and modulate the neuroendocrine balance of the Renin-Angiotensin-Aldosterone System (RAAS) and the sympathetic nervous system (SNS). Therefore, the goal of treatment is not only to improve symptoms and quality

of life, but also to delay the development of myocardial remodeling, terminate the vicious circle of progression, thereby to reduce the mortality and hospitalization rate of heart failure.

Myocardial remodeling is a major mechanism in development of heart failure, which include pathological cardiomyocyte hypertrophy with re-expression of embryonic genes, cardiomyocyte apoptosis and necrosis, and excessive deposition or degradation of myocardial extracellular matrix. Delaying or preventing myocardial remodeling is of great value in preventing and controlling the development of heart failure and improvement of cardiac function. Activation of two neuroendocrine systems, including SNS and RAAS, is closely associated with myocardial remodeling in heart failure. angiotensin-converting enzyme inhibitor (ACEI) or angiotensin receptor blocker (ARB), beta blockers, and aldosterone receptor antagonists are shown to be beneficial, indicating the effectiveness of inhibiting these two systems. However, the mechanisms of heart failure are not limited to the SNS and RAAS, further research are needed to explore the effective anti-heart failure pathways, and infer new targets for treating heart failure.

Numbers of clinical studies in the late 1980s also confirmed that activation of neuroendocrine system leads to myocardial remodeling, which is a key factor in the occurrence and development of heart failure. In 1987, CONSENSUS, a clinical trial of ACEI for heart failure, successfully reduced the total mortality of heart failure patients by 27%. Later, clinical trials such as SOLVED and V-HeFY further confirmed that ACEI can improve the prognosis of heart failure.

In the mid-to-late 1990s, CIBISII, MERIT-HF and COPERNICUS studies confirmed that beta blockers reduced mortality in heart failure patients by 34% to 35%. In addition, the RALES trial (1999) and EMPHASIS-HF (2011) studies had shown that aldosterone receptor antagonists can reduce mortality in heart failure patients by 24% to 30%.

Furthermore, novel medications that have demonstrated beneficial effects in patients with heart failure since 2010 are Angiotensin Receptor-Neprilysin inhibitor (ARNi) and sinus node inhibitor. In the PARADIGM-HF trial, 8442 heart failure patients with reduced ejection fraction (HFrEF) were randomized to receive ARNI and enalapril. The incidence of primary endpoints (cardiovascular death and hospitalization for heart failure) was 21.8% in ARNI group, significantly lower than the enalapril group (26.5%) [13]. The SHIFT study showed that the relative risk of hospitalization for cardiovascular death and worsening heart failure was reduced by 18% in ivabradine group compared with the standard treatment group, and left ventricular function and quality of life were significantly improved [14]. At the same time, clinical research has also found that diuretics can effectively relieve dyspnea in patients with heart failure. Rational use of diuretics is the key and foundation for other successful treatment of heart failure by eliminates fluid retention, improves heart function and exercise tolerance.

During this period, non-pharmacological treatment of heart failure has also made some important progress. In addition to the optimal drug treatment, cardiac resynchronization therapy can further improve the heart function and quality of life and reduce mortality in indicated heart failure patients.

Despite these progresses in the field of heart failure treatment in the past decades, the current prevalence and mortality of heart failure remain high. Novel treatment approaches is still in needs to improve in prognosis and outcomes of heart failure patients.

Traditional Chinese medicine (TCM) has accumulated practical experience in the prevention and treatment of heart failure. There have been various translation researches and reports on the treatment of heart failure with TCM. Among, the TCM treatment of heart failure, Qiliqiangxin Capsules (QLQX) has shown to be beneficial and effective in patients

with chronic HF. The research results were published on the *Journal of the American College of Cardiology* in 2013 [15] and received the attention and praise from worldwide scholars. All patients were treated with standard-optimized HF treatment, and the ratio of serum N-terminal pro-B-type natriuretic peptide (NT-proBNP) was significantly lower in the Qiliqiangxin capsule group compared with the placebo group. In specify, higher ratio of NT-proBNP decrease of >30% and lower incidence of adverse events in QLQX group were observed, indicating that QLQX is effective and safe in the treatment of CHF. This positive secondary endpoint has led QLQX, this proprietary Chinese medicine, to be included in the Guidelines for the Diagnosis and Treatment of Heart Failure in China 2014 and 2018.

Based on the TCM theory of collateral disease, Qiliqiangxin Capsules is the first TCM to explore the pathogenesis and treatment of chronic heart failure, which proposes insufficiency of the heart-qi as the basis of heart failure. Furthermore, obstruction of collaterals is the central link. The obstruction of the fluid flow leads to exude from veins, which causing blood stasis and water retention in the veins, then inducing edema. Prolong obstruction of collateral network will eventually escalate [16]. The theory is mirror with the modern medicine concept of the development of heart failure in recent years that the hemodynamic changes caused by early neuroendocrine activation can lead to ventricular remodeling.

Due to the post-marketing unique efficacy of QLQX in the clinical application, various of research towards the mechanism of QLQX have shown that it has cardiotonic and diuretic effect, improved the heart function and increased water discharge by reducing the renal expression of AQP2 in animal models [17]. It also can reduce the expression of AngII and periostin proteins in rats with heart failure after myocardial infarction and is dose-dependent [18]. These results suggested mechanism of the QLQX on improving cardiac function is related to the modulation of volume control and inhibition of myocardial remodeling [19]. Also, it might reduce the pro-inflammatory factors of cardiomyocytes and increasing the immunomodulatory effects of anti-inflammatory factors for improving heart function in AMI rats [20]. In the clinical real-world study of Qiliqiangxin Capsules in the treatment of patients with chronic congestive heart failure, it is indicated that Qiliqiangxin Capsules can improve cardiac function grading, heart failure (Lee's) score, systolic and diastolic function, ejection fraction (EF), TCM syndrome differentiation (中医证候), quality of life and other curative indicators and safety indicators, elevate blood nitric oxide (NO) and calcitonin gene-related peptide (CGRP) levels, and decrease endothelin (ET) levels; thereby significantly improving endothelial function in patients with heart failure.

In summary, Qiliqiangxin Capsules might have a potential in further optimizing the treatment of heart failure by modulating multiple pathways, multiple links and multiple targets. It reflects the advantages of compound Chinese medicine in treating heart failure from the holistic treatment. Based on the previous study showing significantly reduces NT-proBNP levels, QLQX might improve of the long-term prognosis in chronic heart failure. With the evidence-based medicine approach and incorporate the incidence of composite endpoint events consisting of cardiovascular death and hospitalization for heart failure as the study primary endpoints, current study is a large randomized, double-blind, multi-center clinical study to further elucidate the long-term efficacy and safety of QLQX in chronic heart failure.

4. Study Design

This study is a randomized, double-blind, placebo-controlled, parallel-group, multicenter clinical study.

The study will be event-driven, and all randomized patients will remain in the study (whether taking the study drug or not) until the number of primary endpoint events reaches the predicted value (620 cases), or the study terminates early when it meets the pre-defined efficacy or safety criteria of early termination.

Two mid-term efficacy analyses planned to be conducted after 1/2 and 2/3 primary endpoint events to assess whether an invalid or valid conclusion was reached so as to prematurely end the study.

The entire study will last approximately 36 months, and the recruitment period will be expected to be 24 months. The follow-up period after the last case of patient is included in the study is 12 months. The average follow-up time is predicted to be about 24 months.

Patients who show stable clinical symptoms, had received at least 2 weeks of standardized treatment and treatment of other concomitant diseases before enrollment are screened at the hospital. According to the local HF treatment guidelines, the drug type and dosage are fixed, unless it is contraindicated or intolerant. The patient who have not receive anti-HF drug intravenously for at least two weeks prior to enrollment, nor take oral administration of TCM or Chinese patent medicine having similar composition with Qiliqiangxin Capsule can directly enter the random grouping stage.

If patients fail to meet the above requirements, term standardized treatment to meet the above criteria before entering the random grouping stage are needed.

4.1. Randomization and Enrollment (Day 0 – 24th month)

Noted: Patients will have to receive at least 2 weeks of standardized heart failure treatment without any other TCM and/or herbal medicine before randomization. Regimen of the heart failure treatment will be documented. Any adjustment of the medication will be recorded in the case report form (CRF).

Patients will be randomized 1:1 into study group and placebo group to receive the investigation products (IP) in addition to the standard therapy.

Study group: Standardized treatment of chronic heart failure + Qiliqiangxin capsules, 4 capsules, 3 times/day, administered orally.

Control group: Standardized treatment of chronic heart failure + Placebo capsules, 4 capsules, 3 times/day, administered orally.

Treatment with any other TCM and herbal medicine should also be avoided after randomization.

After randomization, patients will be followed up at 1st, 3rd, 6th, 9th, 12th month, and every other 3 months for efficacy and safety assessment until the end of the trial. The randomization and enrollment period will last for 24 months.

4.2. Control group and Sample size

According to the PARADIGM-HF study, the composite event of cardiovascular death and/or hospitalization for heart failure in the median follow-up of 27 months was 21.8% in the LCZ696 group and 26.5% in the Enalapril group. Therefore, we estimated that the incidence of cardiovascular death and hospitalization for heart failure is 25% in patients with standardized treatment + placebo group within 36 months of follow-up and 20% in standardized treatment +

Qiliqiangxin capsule group.

The random distribution ratio is 1:1 between study group and control group. Considering the consumption of type I error in the interim analysis, α is adjusted to unilateral 0.02314. Based on the incidence of composite endpoint events is 25%, it is expected that 620 composite endpoint events need be observed to provide 80% power of test (β= 0.2), and 20% risk can be reduced in study group by log-rank test.

The entire study will last approximately 36 months to follow up, and the recruitment period are expected to be 24 months. The sample size is expected that 3,080 patients in over 100 centers (1540 patients per group) will be enrolled and be followed up at least for 12 months.

5. Rationale for Study Population

The enrolled patients should satisfy the following inclusion criteria, and not meet any exclusion criterion. In addition to following criteria, the patient should also be excluded if there is any contraindicated medical condition or use of incompatibility drug during basic treatment period.

5.1. Inclusion Criteria

1) Signed informed consent;
2) Aged ≥18 years at the time of consent;
3) Established documented diagnosis of heart failure for at least three months ago according to "Chinese Heart Failure Diagnosis and Treatment Guideline" issued by the Chinese Medical Association Cardiovascular Branch;
4) Left ventricular ejection fraction (LVEF) ≤40% (echocardiogram, radionuclide, ventriculogram, contrast angiography or cardiac MRI);
5) NYHA cardiac functional grading II to III, with stable clinical symptoms; or those diagnosed as grade IV within 2 weeks before enrollment;
6) Serum NT-proBNP ≥450pg/ml;
7) Those who have received standardized baseline treatment regimens without doses adjusted and given intravenously for at least two weeks prior to enrollment;
Standardized drug treatment includes angiotensin-converting enzyme inhibitor (ACEI) or angiotensin receptor blocker (ARB) or angiotensin receptor neprilysin inhibitor (ARNI), beta blocker, and aldosterone receptor antagonist (the optimal therapeutic dose should be achieved, except for contraindications or intolerance).

5.2. Exclusion Criteria

1) Patients should not enter the study if any of the following exclusion criteria are fulfilled
2) Heart failure caused by valvular disease, congenital heart disease, pericardial disease, arrhythmia or non-cardiaogenic disease, or caused by vital organ failure (such as renal, hepatic failure, etc.); and right heart failure caused by pulmonary or other definite causes; and acute heart failure;
3) Coronary revascularization (percutaneous coronary intervention [PCI] or coronary artery bypass grafting [CABG]) or cardiac synchronization therapy planned to undergo after randomization, or had received cardiac resynchronization therapy prior to enrolment;
4) Any condition outside the CV diseases such as but not limited to malignant tumor, severe mental illness, hematopoietic diseases, neuroendocrine system disease, liver transaminase and alkaline phosphatase ≥ 3 x upper

limit of normal (ULN), abnormal renal function serum creatinine > 2 mg/dl (176.82 umol/L), potassium >5.5mmol/L;

5) Patient with left ventricular outflow tract obstruction, myocarditis, aortic aneurysm, aortic dissection, or obvious hemodynamic changes caused by unrepaired valve;

6) Cardiogenic shock, uncontrollable malignant arrhythmia, sinus or atrioventricular block at second degree type II or above without pacemaker treatment, progressive unstable angina pectoris or acute myocardial infarction;

7) Uncontrolled hypertension systolic blood pressure (SBP) ⩾ 180mmHg and/or diastolic blood pressure (DBP) ⩾ 110mmHg; or SBP < 90mmHg and/or DBP <60mmHg;

8) Participation in another clinical study with an IP during the last month prior to enrolment;

9) Women of child-bearing potential (i.e., those who are not chemically or surgically sterilized or who are not post-menopausal) who are not willing to use a medically accepted method of contraception that is considered reliable in the judgment of the investigator, from the time of signing the informed consent throughout the study and 4 weeks thereafter, OR women who have a positive pregnancy test at enrolment or randomisation OR women who are breast-feeding;

10) Allergic constitution; known to be allergic to research drug;

11) Inability of the patient, in the opinion of the investigator, to understand and/or comply with study medications, procedures or any conditions may render the patient unable to complete the study.

5.3. Discontinuation of investigational product (IP)

At any time after randomization, patients are free to discontinue for any reason. Discontinuation from IP is not the same as complete withdrawal from the study. According to the intention-to-treat principle, patients discontinuation from IP will be continue in follow-up and recorded in CRF. For patients could not participate in outpatient follow-up, virtual interview and follow-up according to plan will be proceeded for the assessment of the adverse events and/or endpoint events unless the patient refuses to follow up and withdraw from the study. Study drug treatment can be discontinued when:

1. The patient can stop treatment at any time;

2. The patient has allergic reactions that are clearly associated with the IP;

3. The patient has occurrence of symptoms, signs and/or abnormal examination results that are related to the IP, or the condition determined by the investigator to terminate the study;

4. Pregnancy during the study;

During the trial, the patient should take the standard dose of the IP as long as possible. The patients should resume taking the IP as soon as possible after the relevant causes are excluded and follow up as planned.

5.4. Withdrawal

The patient has the right to withdraw from the study at any time for any reason. The withdrawal of the trial will not intervein the regimen and management.

The researcher should retrieve the remaining IP when the patient withdraws from study. The reason for the withdrawal should be recorded in CRF by follow-up interview or telephone. Follow-up should be continued in order to ascertain whether any endpoints or safety events have occurred. Optimally, patients who discontinue from IP should

continue to attend all study visits according to plan until study finish as much as possible. Information should be recorded in CRF.

5.5. Discontinuation of the study

1) The overall study may be stopped due to the following reasons:

- Base on Data Safety Monitoring Committee (DSMC) interim analysis results;
- Researchers find serious safety problems;
- Major mistakes in the study protocol;
- The sponsors decide to suspend study due to management problems or lack of funding;
- The competent administrative department cancels the experiment, and half-stops all studies.

The discontinuation of the study can be temporary or permanent. During the suspension, all study records should be kept for inspection.

6. Treatment

6.1 Investigational products (IP)

6.1.1. Products information

Study drug: Qiliqiangxin Capsule (芪苈强心胶囊)

-Ingredients: Astragalus, ginseng, monkshood, Danshen, Pepperweed Seed, rhizoma alismatis, radix polygonati officinalis, cassia twig, red flower, cortex periplocae, tangerine peel

-Property: Capsule; the contents are brown to black brown granules; bitter in taste;

-Specification: 0.3g/ capsule

-Bach number: GYZZ Z20040141

-Manufacturer: Shijiazhuang Yiling Pharmaceutical Co., Ltd

Placebo: Qiliqiangxin Matching Placebo

-With identical color, specification, packaging, , property of contents and other features with Qiliqiangxin Capsule

6.1.2. Package and label

The overall packages of the IPs are identical. Each capsule weighted 0.3 gram with identical appearance and smell.

Small package: The appearance is shown below and will be labeled with "For QUEST study only". Each package contains 36 capsules sealed in aluminum-plastic plates.

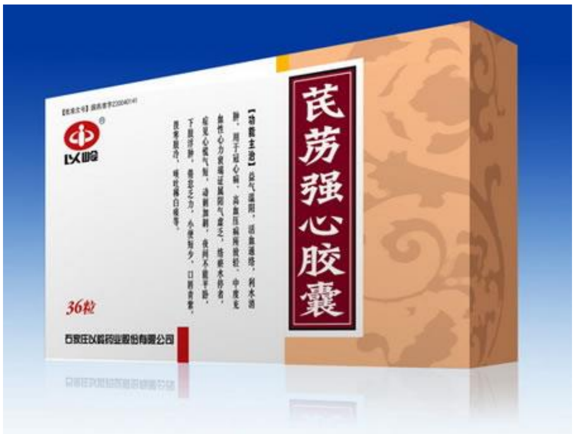

Big package: White paper box, with size of 29.5cm╳12cm╳22cm, each big package includes 33 small packages. Each is marked with following label:

**Qiliqiangxin in Heart FailUre: AssESsment of Reduction in MorTality (QUEST)**<br>**(For Clinical Study Only)**
**Package No.: XXXXX**<br>[Lot number] xxxx   [Expiration date] xxxx
[GYZZ] Z20040141<br>[Package] 33 small boxes, and 36 capsules in each small box (amount for 99-days)<br>[Directions] 3 times a day and 4 capsules each time<br>[Storage] Sealed in a cool dry place. Please store the product out of children's reach.
**Please be sure to follow doctor's orders and visit at the specified date to the hospital for follow-up evaluation. Thank you for your cooperation!**

6.1.3. Storage

All IP should be kept in a secure place under appropriate storage conditions. Each center must assign a committed staff to preserve and manage the study drug.

6.1.4. Distribution and Recollection

The IP provided for this study will be administrated only as directed in the study protocol. Patients will be asked to bring all unused study medication and empty packages to the study. During each follow-up, the investigator or delegate will collect and check the amount of returned capsule and fill in Drug Distribution Form, timely and accurately to account for all IP dispensed to and returned from the patient, in order to determine the compliance of subjects at each site visit. At the end of study, all remaining drugs should be returned. Undistributed IPs should be sealed when returned. Remaining drugs should be retained and uniformly destroyed after study.

6.1.5. Accountability

During each follow-up, the subjects should return all remaining drugs, and researchers must make an inventory of remaining drugs and keep record accordingly.

6.2. Study and Follow-up Plan

6.2.1. Study Procedure

Screening and Enrolment period (day -14 to day 0):

The investigators will review the inclusion and exclusion criteria. Patients who do not meet these criteria must not be randomized into the study. Set of assessments will be completed for these patients.

Randomization and Treatment period (day 0 to 12 months [with maximum of 36 months]):

-Study group: Standardized treatment of chronic heart failure + Qiliqiangxin Capsules (4 capsules/time, 3 times/day);

-Control group: Standardized treatment of chronic heart failure + Placebo Capsules (4 capsules/time, 3 times/day);

The dispensing date of the IPs is regarded as day 0. Patients will be randomized into the study or control group in a 1:1 ratio with the basis of current standardized treatments for chronic heart failure. The study drug is recommended to be taken about 30 minutes after meals, three times a day as follow. Patients will be asked to hold the morning dose on the morning of visit day if possible. If the patient miss to take the IP in a day, the accumulated dose for the next day should not exceed the daily dose. If the patient has an intolerable adverse event, which might relevant to study drug according to the judgment of researcher, the patient should terminate the following treatment with the study drug.

6.2.2. Standardized heart failure treatment

Treatment of the patients will be based on the local or regional heart failure guidelines. The treatment regimen from the 2018 China heart failure diagnosis and treatment guideline are summarized as followed:

1) Stable heart failure symptoms without the use of intravenous diuretics, inotropic, and vasodilator for 2 weeks.
2) Patients received standardized baseline treatment regimens without doses adjusted at least two weeks prior to randomization. Standardized treatment includes: angiotensin-converting enzyme inhibitor (ACEI) or angiotensin receptor blocker (ARB) or angiotensin receptor neprilysin inhibitor (ARNI), beta blocker, and aldosterone receptor antagonist (the optimal therapeutic dose should be achieved, except for contraindications or intolerance).
3) After entering the treatment period, Dose reduction or discontinuation of proven effective therapies should be avoided unless all other measures fail to improve the patient’s situation. Any adjustment of the treatment regimen should be recorded in the CRF. If the patient has experienced any potential endpoints, SAEs, DAEs and/or AEs of interest since the last visit, these should be recorded in the CRF.

6.2.3. Concomitant medications and other treatments

All patients should be treated according to regional standard of care for HF and other comorbidity(s). Also, cardiac and heart failure related procedures will be captured during the study. Background medication will not be provided by the Sponsor.

1) Detailed recording of medications related to HF, HTN as well as other relevant cardiovascular medications (e.g., statins, antihypertensive and antithrombotic agents) will be made throughout the study;
2) TCM and/or herbal medication have similar contents to the test drug should not be used during the entire treatment period after entering the randomization period.
3) Patients should receive dietary guidance for heart health, such as low-salt diet, moderate drinking, etc. Patients should also receive counseling for appropriate lifestyle improvements such as weight monitoring, physical exercise, smoking cessation, and alcohol withdrawal.
4) No drug has been found to be contraindicated with use of Qiliqiangxin Capsules.

6.2.4. Treatment period

12 months - 36 months

6.3. Evaluation on compliance

In order to determine the compliance of subjects, the administration (drug distribution and recovery) of all investigational products should be recorded in the appropriate sections of the eCRF. The actual dosage should be within 80%-120% of predefined dosage.

6.4 Adverse drug reaction

There is no significant adverse drug reaction of QLQX were found.

7. Outcome Measures for Analyses

7.1 Clinical observation endpoints

7.1.1. Primary outcome measure

- The composite endpoint events consisting of cardiovascular death and/or hospitalization for heart failure;

7.1.2. Secondary outcome measures

- All-cause mortality
- Secondary endpoint events (given up treatment due to worsening heart failure, successful resuscitation after cardiac arrest, malignant arrhythmia, non-fatal stroke)
- Components of the primary endpoints in patients with ischemic heart disease
- Level of Serum NT-proBNP

Note: All endpoint events should be determined and reviewed by Clinical Event Adjudication Committee.

7.2. Safety outcome measure:

- Adverse events (Serious Adverse Events [SAEs], Discontinuation of IP due to Adverse Events, etc.)
- Clinical laboratory indexes: complete blood count (hemoglobin, red blood cells, white blood cells, platelets), routine urine test (urinary protein, urinary white blood cells, urine red blood cells), serum biochemistry (urea nitrogen, creatinine, alanine aminotransferase, fasting blood glucose, potassium, sodium, chlorine, total cholesterol, triglycerides).
- 12-lead ECG
- Physical examination

8. Course of Study

All participants, including those discontinue study drug prior to completion of study, should continuously take all planned visits listed in the table until the end of study. If a visit is postponed or taken in advance, it should not affect the next visit. The next visit should be carried out in accordance with the original planned time.

Visit	1	2	3	4	5	6	7	8	9	10	11	12	13	14	UNS	EOS
**Day/Month**	Day 0	1M	3M	6M	9M	12M	15M	18M	21M	24M	27M	30M	33M	36M		
	-14	±3	±7	±7	±7	±7	±7	±7	±7	±7	±7	±7	±7	±7		≤2week
**Consents**	X															
**Inclusion/Exclusion**	X															
**Randomization**	X															
**Demongraphic**	X															
**Heart Failure History**	X															
**Physical Examination**	X	X[1]	X[1]	X[1]	X[1]	X	X[1]	X[1]	X[1]	X	X[1]	X[1]	X[1]	X	(X)	X
**Heart Failure Medication**	X	X	X	X	X	X	X	X	X	X	X	X	X	X	(X)	X
**CV Medication**	X	X	X	X	X	X	X	X	X	X	X	X	X	X	(X)	X
**Other Medication**	X	X	X	X	X	X	X	X	X	X	X	X	X	X	(X)	X
**Echocardiogram**	X*															
**CBC/ Urine test**	X	X				X				X				X	(X)	X
**Biochemistry**	X	X				X				X				X	(X)	X
**Pregnancy**	X					X				X				X	(X)	X
**12-lead EKG**	X*	X				X				X				X	(X)	X
**NT-proBNP**	X	X	X											X		
**Drug Dispen**	X		X	X	X	X	X	X	X	X	X	X	X	X		
**Drug Recollection**			X	X	X	X	X	X	X	X	X	X	X	X		X
**Endpoint**		X	X	X	X	X	X	X	X	X	X	X	X	X	(X)	X
**AE**		X	X	X	X	X	X	X	X	X	X	X	X	X	(X)	X
**SAE**		X	X	X	X	X	X	X	X	X	X	X	X	X	(X)	X

X[1]Simplified physical examination, X* Echocardiogram and 12-lead EKG within 6 months for screening;

- UNS (unplanned visit): (X) The marked item is optional and performed according to the judgment of researchers.
- EOS (final visit): Make arrangement according to the study end time (if there is a visit within one month before the end of study, it is regarded as a final visit, but needs to be supplemented with the items required completely)
- Pregnancy test is only applicable to women of childbearing age (if the urine pregnancy test is positive, it must be confirmed by serum pregnancy test).

9. Efficacy Assessments and Clinical Event Adjudication (CEA) Committee

9.1. Definitions of endpoint events

- **Hospitalization for heart failure:**

1. The patient was hospitalized for HF diagnosed preliminarily;

2. The patients who were admitted in hospital extended for at least 24 hours (or if the hospitalization time and discharge time were not available, it should indicate change of calendar date);

3. Record on the patient report that there are new symptoms or worsening symptoms due to HF, including at least one of the following:

a. Difficulty breathing (difficulty breathing on exertion, difficulty breathing on resting, orthopnea, paroxysmal breath with difficulty at night)

b. Reduced exercise tolerance

c. Fatigue

4. The patient had objective evidence of an acute exacerbation of HF, including at least two health examination results or a health examination result and at least one laboratory standard, including:

a. Determine the health examination results caused by HF, including new or deteriorated:

1) Peripheral edema; 2) Abdominal distension or increase of ascetic fluid (in the absence of primary liver disease); 3) Lung rales and/or crackles; 4) Increased jugular venous pressure and/or hepatojugular reflex (+); 5) S3 galloping; 6) Clinically significant or rapid weight gain, having relation with fluid retention.

b. Laboratory evidence of new or worsening HF obtained within 24 hours, including:

1) Increased concentration of B-type natriuretic peptide (BNP) / N-terminal B-type natriuretic peptide precursor (NT-proBNP) consistent with acute decompensated HF (eg.: BNP > 500 pg/ml or NT-proBNP > 2000 pg/ml); In patients with long-term elevation of natriuretic peptides, special attention should be paid to a significant increase beyond baseline. 2) Imaging evidence of pulmonary congestion; 3) Non-invasive diagnostic evidence of clinically significant increase in left or right ventricular filling pressure or decreased cardiac output; echocardiographic criteria includes: E/e´>15 or D leading pulmonary venous inflow pattern, congestive inferior vena cava with minimal inspiratory collapse, or reduction of small stroke distance (time velocity integral; TVI) at left ventricular outflow (LVOT). 4) Invasive diagnostic evidence: right heart catheterization showed pulmonary capillary wedge pressure (pulmonary artery wedge pressure) ≧18mmHg, central venous pressure ≧12mmHg, or cardiac output index <2.2L/min/m^2;

Note: If applicable, all results in the diagnostic test need to be recorded; even if the above criteria are not met, results might provide important information for the determination of the above events.

5. The patients receive an initial or intensive treatment for HF, including at least one of the following:

a. Enhance the treatment of oral diuretics;

b. Intravenous diuretics or vasoactive drugs (such as inotropics, vasopressors or vasodilators);

c. Mechanical or surgical intervention, including:

1) Mechanical circulation support (e.g.: Intra-aortic balloon pump, ventricular assist device, extracorporeal

membrane oxygenation, total artificial heart);

2) Mechanically assisted removal of body fluids (e.g.: ultrafiltration, hemofiltration, and dialysis);

- **Cardiovascular death:** including death caused by acute myocardial infarction (AMI), sudden cardiac death, acute decompensated heart failure, stroke, cardiovascular (CV) surgery, CV bleeding, and other CV inducing death;
- **All-cause mortality**
- **Given up treatment due to the worsening of HF:** Worsening of heart failure symptoms and signs, requiring intravenous drug or mechanical support treatment, and patients or family members voluntarily give up treatment or left hospital without cure; if the result of follow-up is death, it is included in heart failure death.
- **Successful resuscitation after cardiac arrest**
- **Malignant arrhythmia**： There is no uniform standard for the definition of malignant arrhythmia. It generally refers to arrhythmia that can cause severe hemodynamic disorder in a short period of time, causing syncope or even sudden death. According to this standard, malignant arrhythmia mainly has the following categories: (1) severe bradyarrhythmia, such as severe sick sinus syndrome, high or third degree atrioventricular block; (2) tachyarrhythmia, such as persistent ventricular tachycardia, ventricular flutter, ventricular fibrillation, atrial flutter/atrial fibrillation with rapid ventricular rates, atrioventricular reentry tachycardia, pre-excitation syndrome with atrial fibrillation, sinus tachycardia, etc.
- **Non-fatal stroke**

9.2. Endpoint reporting overview

When potential endpoint events have been identified, the researchers should collect all relevant support documents within 7 days and report to CEA committee. For each potential endpoint event, the investigator or delegate will record information in the CRF. Investigators will record the incident in endpoint report form and submit supporting data in a timely manner (admission and discharge records, medical records, death records, ECG, etc.). The potential endpoints event (All deaths, All HF events [hospitalizations for HF or urgent HF visits], cardiac ischemic events [MI and unstable angina], cerebrovascular events [stroke and TIA], etc.) will be reviewed for central CEA process.

CEA committee consists of a chairman and 5-6 members. Each case will be independently reviewed by two members of the committee. The conclusions will be submitted to the chairman of the committee.

10. Safety Assessment

10.1. Definition of Adverse Event:

- Adverse events (AE): AE refers to any adverse medical events occurring in this clinical experiment from the moment that the patient signs the informed consent and is chosen to participate in this study to the last follow-up, whether or not the events are caused by the use of the medicine described.
- Major adverse events: Any adverse events and/or laboratory abnormalities, other than SAEs, that lead to targeted medical interventions (e.g., discontinuation of medication, dose adjustment, and symptomatic treatment).

10.2. Grading of Adverse events:

All clinical adverse events that occur in this clinical study will be recorded on the CRF adverse event page. The severity of adverse events will be classified. For uniform standards, the severity of events is classified as follows:

Grade 1: Mild, no clinical symptoms or mild clinical symptoms; only clinical or laboratory abnormalities; no treatment required.

Grade 2: Moderate, requiring minimal, local, or non-invasive treatment; daily life activities using age-appropriate tools [a] are restricted, such as cooking, shopping, and making phone calls.

Grade 3: Severe illness or severe medical symptoms that are temporarily not life-threatening; resulting in hospitalization or prolonged hospital stay; resulting in disability; restricted in daily living activities [b]. Activities of daily living refer to bathing, dressing, undressing, eating, using the toilet, taking medication, and being non-bedridden.

Grade 4: Life-threatening, requiring emergency treatment.

Grade 5: Death due to adverse events.

a: Daily living activities with refer to cooking, buying daily necessities or clothes, using the phone, managing finances, etc.

b: Daily living activities refer to bathing, dressing/undressing, eating, grooming, taking medication, and not being bedridden.

10.3. IP related adverse events of interest

Criteria for assessing the relationship between adverse events and investigational drugs. All causal analyses of adverse events related to investigational drugs are assessed according to five levels: definite, possible, unlikely, definite unrelated, and uncertain. The first three are considered adverse drug reactions. There are five main considerations for causal analysis:

1) Definite: There is a reasonable temporal sequence between the occurrence of the AE and the use of the investigational drug. The AE is a known adverse reaction to the investigational drug. The AE is alleviated or disappears upon discontinuation of the investigational drug, and reoccurs upon repeated use. It cannot be explained by the subject's underlying disease.
2) Possible: There is a reasonable temporal sequence between the occurrence of the AE and the use of the

investigational drug. The AE is a known or suspected adverse reaction to the investigational drug, but there may be other factors that could cause the event, such as disease or concurrent medication. The AE is alleviated or disappears upon discontinuation of the investigational drug, or the effect of discontinuation on the event is unclear, or there is a lack of decisive information.

3) Unlikely: There is a reasonable temporal sequence between the occurrence of the AE and the use of the investigational drug, but it is not a known adverse reaction to the investigational drug type, and it is highly likely to be caused by the subject's illness or other treatment.
4) Definite unrelated: There is no reasonable temporal sequence between the AE and the use of the investigational drug, such as events that occurred before the use of the investigational drug, or events that are not known adverse reactions to the investigational drug, or events that are clearly caused by other factors such as the subject's underlying disease or concurrent medication.
5) Uncertain: There is no clear relationship between the timing of the AE and the medication, and the known type of adverse reaction is similar to the investigational drug. Other co-administered drugs may cause the same reaction, and there is not enough evidence to make a clear decision.

The "definite," "possible," and "uncertain" categories are combined to calculate the incidence of adverse reactions to the investigational drug.

10.4. Adjudication of severe adverse events

10.4.1. Definition of severe adverse events

Serious adverse events (SAE) refer to any clinical events that indicate significant harm, contraindications, adverse reactions, or the need for caution. Adverse events meet the criteria for SAE when they meet one or more of the following standards:

- Death
- Life-threatening (referring to the immediate risk of death for the patient at the time of the event; this does not include events that may lead to death if they become more serious)
- Result in hospitalization or prolonged hospital stay
- Result in persistent or significant work loss or disability
- Congenital malformations or defects

Other events that have not resulted in death, life-threatening situations, or the need for hospitalization but are considered to be harmful to patients or subjects, or require drug or surgical treatment to avoid the above situations upon appropriate medical judgment, should also be considered as SAE.

10.4.2. Definition on specific severe adverse events

In this study, the following events will not be reported as serious adverse events unless they are judged negative and the researcher believes that it is related to the study drug

◆ Cardiovascular death

- Hospitalization for heart failure
- Given up treatment due to worsening heart failure
- Successful resuscitation after cardiac arrest
- Malignant arrhythmia
- Non-fatal stroke

Other events leading to fatal outcomes should be reported as serious adverse events.

10.5. Recording of adverse events and follow-up

If any adverse events occurred, especially those associated with the study drug, should be followed up until the patients return to baseline or tend to stabilize. If the baseline status or stability cannot be restored after follow-up, it should be noted in the CRF. All SAEs must be reported within 24 hours, whether or not considered causally related to the investigational product, or to the study procedure(s). At the same time, researchers must complete a serious adverse event form, recording the time, severity, duration, measures taken, and outcome of serious adverse events.

10.6. Adverse events based on examinations

The results from protocol mandated laboratory tests and vital signs will be summarized and give possible interpretation. The abnormal laboratory results caused by reported adverse events should be recorded in the adverse event form. The abnormal results with clinical significance that meets one or more following conditions should be recorded as independent diagnosis on adverse event page of CRF (excluding abnormal laboratory result caused by reported adverse events):

- With associated clinical signs and symptoms
- Change in course of study drug's treatment dose
- Change in any of standard evidence-based medications and (or) other treatment measures need to be changed

11. Blinding and Unblinding

11.1. Random grouping of subjects

Statistical experts at Peking University Clinical Research Institute adopts SAS 9.4 statistical software package to generate random numbers using the block randomization method according to the ratio of 1:1 between study group and control group. The study drug (Qiliqiangxin or placebo capsules) was packaged according to this random number by the person unrelated to the study.

A randomization and trail supply management system (RTSM) is used in the study, and statistical professionals will provide a random numbered list to the RTSM. The patient is then assigned a random number by the RTSM.

After completing baseline assessment, random numbers are assigned by RTSM during baseline visits. After that, the drug number is obtained through the RTSM according to the interview plan, and the serial number of drug assigned each time is different, but the drugs are the same. Before randomization of patient, the researcher must first log into the RTSM and provide the according information (e.g. the subject's date of birth, gender).

11.2. Unblinding and emergency unblinding protocol

If an adverse event occurs, emergency unblinding can only be conducted in special circumstances where it is necessary to understand the use of the investigational drug in order to treat the patient. Once the decision to unblind has been made, the researcher must record the date, time, and reason for unblinding.

The researcher needs to log in to the RTSM system to complete the unblinding application, which will be reviewed by the principal investigator before being unblinded by the unblinding officer. Once unblinded, the case will be discontinued from the study and treated as a withdrawal.

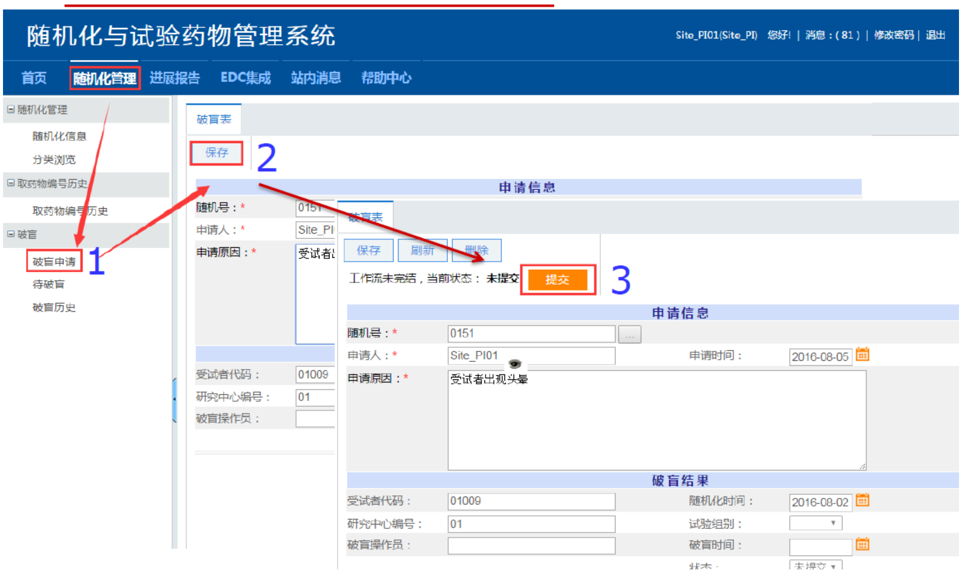

11.3. Unblinding provisions

All personnel involved with the analysis of the study will remain blinded until database lock and protocol violations have been identified and documented the study adopts two-step unblinding provision. After blind check, the data is locked, main researchers, medical statisticians, data administrators and sponsor representatives will do the first unblinding, and

the random number corresponding to the group will be marked as A or B, in order to make statistical analysis on all data. At the end of statistical analysis when summary report is completed, a secondary unblinding would be taken to reveal the group of A and B.

11.4. Screening number

The screening number was made according to the sequence of patients taking treatment at each hospital, and represented with center ID + three integers, such as the screening number is 01001, 01002 …... for center 01.

12. Statistical Analysis

After the research protocol is established, the statistical professionals, in collaboration with the primary researchers, will develop a statistical analysis plan. SAS®9.4 software or a higher version will be used for statistical analysis, and PASS13 was used for sample size calculations.

12.1. Definitions of analysis sets

The study population will be divided into the following categories:

- Full Analysis Set (FAS): This refers to the dataset that includes all randomized subjects who received the study drug at least once, with minimal and reasonable exclusion of subjects adhering to the intention-to-treat principle. Exclusions typically include: important inclusion criteria violations, subjects who did not receive the study drug treatment, and subjects with no post-randomization observation data. The primary efficacy endpoint is the time to composite endpoint event, analyzed using survival analysis methods. In selecting FAS for statistical analysis, missing data for the primary endpoint event will be handled by deletion.
- Per Protocol Set (PPS): This is a subset of FAS, in which the subjects have better adherence to the protocol. Subjects included in PPS typically have the following characteristics: (1) completed the minimum exposure to the study drug as predetermined in the protocol, with adherence to drug administration of at least 80%; (2) data for the primary endpoint event are available; and (3) no significant protocol violations.
- Safety Set (SS): This includes all randomized subjects who received at least one treatment and have safety evaluations. No imputations will be made for safety data.

Efficacy analysis will be based on FAS and PPS, while safety analysis will be based on SS. Baseline demographics will be analyzed based on FAS.

12.2. General Statistical methods

Efficacy analysis is taken on the basis of FAS and PPS. All baseline demographic data analysis will be performed on the basis of FAS and safety evaluation on SAS.

- All data are performed with two-sided test, and P value less than or equal to 0.05 (two-sided test) is considered with statistical difference (unless otherwise specified).
- Descriptive analysis: Classification data is described with number of cases and percentage. Quantitative data is described with mean, standard deviation, maximum value and minimum value, median, the first quartile (Q1) and third quartile (Q3).
- Comparison of general situation should be analyzed with appropriate method based on the type of index. Quantitative data should be analyzed with paired t test or Wilcoxon rank sum test; classification data with

chi-square test or precise probability method, and grade data with Wilcoxon rank-sum test or CMH test.

12.2.1. Enrollment and completion

- The number of subjects enrolled and completed at each center will be summarized, and a list of withdrawal will be provided. Comparisons of group sizes, distribution of cases at each center, dropout rates, and detailed termination reasons will be presented. The demographic characteristics of patients (age, height, vital signs, etc.), medical history, and medication history will be described. The comparability of the two groups in terms of age, height, weight, etc. will also be assessed.

12.2.2. Compliance analysis:

- Medication adherence will be compared between the two groups to evaluate whether the study drug was used on time and in the correct dosage, without using any prohibited drugs or food.
- Co-medication usage will be counted for each group and listed in detail.

12.2.3. Efficacy analysis:

- PP analysis and FAS analysis were performed simultaneously with efficacy evaluation;
- Main efficacy evaluation index is the time when a composite endpoint event (cardiovascular death and hospitalization for deterioration of heart failure) occurs. The lack of primary endpoint events is consodered as censored data.

The main research hypotheses are:

H0: $\lambda T / \lambda C \geq 1$

H1: $\lambda T / \lambda C < 1$

λT and λC are the risk of endpoint events in the study group and control group respectively. The Kaplan-Meier method was used to estimate the incidence of clinical endpoints, and a Log rank test was performed for analysis between the two groups. COX proportional hazard model was used and the center was served as a covariate to estimate the hazard ratio and the 95% confidence interval. The composite endpoints, cardiovascular death and rehospitalization for deterioration of heart failure, were analyzed separately.

- Secondary efficacy endpoints:

  All-cause Mortality: The Kaplan-Meier method will be used to estimate the all-cause mortality rates of the two groups, and the Log Rank test will be used for statistical comparison. The COX proportional hazard model will be utilized to estimate the hazard ratios (HRs) and their 95% confidence intervals (CIs) with center as a covariate.

  Composite endpoint (consisting of worsening heart failure leading to withdrawal of treatment, successful resuscitation after cardiac arrest, malignant arrhythmia, and non-fatal stroke): Analyzed using the same method as all-cause mortality.

  Cardiovascular death and hospitalization for heart failure in patients with coronary heart disease: Analyzed as all-cause mortality.

  Serum NT-proBNP level: Analyzed using measurement data, with statistical description and inter-group

comparison for the two groups' baseline and change from baseline.

12.2.4. Safety analysis:

Safety analysis is taken based on SS data set.

Data of adverse events (case number, times and incidence of various adverse events) are compared between the two groups. At the same time, detailed description of specific manifestation, extent of all adverse events and the relation with drugs would be further analyzed.

Crosstab is adopted to describe the change of laboratory index. Number of normal cases before treatment, number of abnormal cases after treatment and ratio of abnormal cases are analyzed in study group and control group. Indexes of vital signs are compared between before and after treatment.

12.3. Interim analysis

The study plans to perform two interim efficacy analyses after collecting 1/2 and 2/3 primary endpoints to assess whether a valid conclusion has been reached and then terminate the study early. According to Lan-DeMets α spending function and the O'Brien-Fleming method, the spending type I error was $\alpha = 0.0001$ (one side) in the first interim analysis period, and $\alpha = 0.00605$ (one side) in the second interim analysis period.

The specific requirements and operations related to the interim analysis will be specified in the DSMB in advance.

13. Data Management

This study used Epidata software to collect research data. Data management ensures the authenticity, integrality and accuracy of clinical data. The data management process needs to comply with the regulatory requirements of Clinical Trial Quality Management Regulations and Clinical Trial Data Management Work Technical Guidelines, in order to ensure traceability of study data. The main processes for data management are listed below.

13.1. Database Design

The data administrator adopts the Epidata software to design and release database according to the CRF after testing.

13.2. Data entry

Clinical research coordinator (CRC) is responsible for inputting the CRF data into the database. The data entry adopts secondary recording mode. Two CRC respectively input the data. Data administrator compares the two databases to generate the data inconsistency list. CRC modified the databases respectively according to the list and the CRF, and then made comparison again. The above steps are repeated until the two databases being identical.

13.3. Data questioning management

The data administrator wrote data verification SAS program according to the data verification plan (DVP) to verify and generated a data questioning list. The data questioning table would be generated after manual verification, and clinical research auditor (CRA) gives the data questioning table to the researcher for answer. After the researcher answering the question, CRA returned the data questioning table to data administrator and revised the database accordingly.

13.4. Medical coding

The medical coding of adverse events is done according to MedDRA 21.0 or advance version.

13.5. Data audit

After completion of database cleanup, the data administrator should write Data Verification Report for holding a data verification meeting.

The major recording contents of the audit report: number of enrolled cases, the condition of off cases and exclusion cases, the condition of deviation or violation from the program, compliance data, drug combination, adverse events, data related to the evaluation indicators, etc.

At the data audit meeting, the division of statistical population is discussed and determined according to the content of audit report.

13.6. Database locking

Complete the database locking list and lock the database according to the database locking program. Any issues discovered after locking the data can be corrected in the statistical analysis program once confirmed. If there is solid evidence showing that it is necessary to unlock the data, the researchers and related personnel must sign an unlocking document.

After the database is locked, the data administrator exports the data in SAS format and hands it over to the statistical personnel for statistical analysis.

14. Quality Control

1) Researchers should fulfill their respective responsibilities, strictly follow the clinical research plan, use standard operating procedures, verify all relevant observations and findings, and ensure the quality control and quality assurance system of clinical research implementation.
2) The allocation of subjects in clinical research must follow the randomized allocation plan determined by the research design. Each subject's processing group code should be saved by the statistical unit and the researcher respectively as a blind bottom.
3) Researchers must conduct necessary training for all personnel participating in clinical research, explain the relevant information, operating procedures and responsibilities, and ensure that the data is entered into the medical records and CRFs truthfully, accurately, completely, timely, and legally. The CRF must be guarded by a dedicated person.
4) Monitors should follow standard operating procedures, supervise the implementation of the research plan, confirm that all data records and reports are correct and complete, all CRFs are filled in correctly, and consistent with the original data.
5) Inspectors should systematically check clinical research-related activities and documents to evaluate whether the research is conducted in accordance with the plan, standard operating procedures, and relevant regulations.
6) All laboratory testing data in clinical research must be accurate and recorded or a copy of the original report pasted on the case report form.
7) Medical statisticians should include the research data completely and accurately in the report, and all steps involving

data management must be recorded for inspection of data quality and implementation process.

8) The statistical analysis process and results of clinical research data must use standardized statistical methods. Medical statisticians should participate in all stages of clinical research. The summary report of clinical research must match the statistical report.

9) The clinical research must be conducted strictly according to the approved plan, and any deviations from the plan must be recorded. Modifications to the research plan require a modification statement and approval from the ethics committee before being implemented.

10) Each research center has one research leader and several fixed members of the research group. The clinical research must be conducted strictly according to the research plan requirements. The leader's technical personnel should maintain close contact with each research center and inspect case observation records at different stages of the research, resolving possible problems in a timely manner.

15. Ethical conduct of the study

1. In the process of clinical research, the personal rights of the subjects must be fully protected to ensure the scientificity and reliability of the research. The rights, safety, and health of the subjects surpasses considerations of scientific and social benefits.
2. The research plan must be reviewed and approved by the ethics committee and signed off on, before being implemented. Any modifications to the research plan during the research process must be approved by the ethics committee. Serious adverse events that occur during the study must be reported to the ethics committee in a timely manner.
3. The researcher or their designated representative must explain the details of the clinical research to the subjects, and obtain informed consent after a full and detailed explanation of the research content.

16. Study timetable and end of study

July 2018	Completed the development of the trial plan and held preparatory meetings
December 2018	Modified the plan and passed ethics approval
January 2019	Study drug and data preparation
March 2019	Trial registration and sub-centers initiation
May 2019	Select and enroll the first case
May 2021	Complete randomized grouping of all cases
June 2022	Complete follow-up of all cases in each center
October 2022	Complete data entry and blind review
December 2022	Statistical analysis
February 2023	Complete the study summary report

17. Data Archiving

All study hospitals should keep these original data at least for five years after the termination of clinical study, including confirmation of all participants (effectively verify all records, such as CRF and hospital original record), informed consent, CRF form, and detailed records of drug distribution of all subjects.

18. Clinical Summary

After the end of statistical analysis, the main researchers are responsible for composing the clinical summary report and affixing the official seal of main research unit.

References

1) Lopez AD. Assessing the burden of mortaIity from cardiovascuIar diS-eases[J]. WorId Health Star Q, 1993, 46(2):91-96.

2) Cardiovascular Branch of Chinese Medical Association. Guidelines for the diagnosis and treatment of chronic heart failure [J]. Chinese Journal of Cardiovascular Diseases, 2007, 35(12):1076-1095.

3) Yuzhu CHEN. Practical Internal Medicine [M]. Beijing: People's Medical Publishing House, 2001.

4) World health organization. Cardiovascular disease:prevention and control[EB/OL]. http://www.who.int/dietphysicalactivity/publications/facts/cvd/en/. 2010-03-07.

5) Mendez GF, Cowie MR. The epidemiological features of heart failure in developing countries:a review of the literature[J]. Int J Cardiol, 2001, 80(2-3):213-219.

6) Rosamond W, Flegal K, Friday G, et al. Heart disease and stroke statistics - 2007 update - A report from the American Heart Association Statistics Committee and Stroke Statistics Subcommittee[J]. Circulation, 2007, 115(5):E69E171.

7) Dongfeng GU, Guangyong HUANG, Xigui WU, et al. Epidemiological investigation of heart failure in China and its prevalence rate [J]. Chinese Journal of Cardiovascular Diseases, 2003, 31(1):3-6.

8) Roger VL, Weston SA, Redfield MA, et al. Trends in heart failure incidence and survival in a community-based population[J]. JAMA, 2004, 292(3):344-350.

9) Pulignano G, Del S D, Tavazzi L, et al. Clinical features and outcomes of elderly outpatients with heart failure followed up in hospital cardiology units: data from a large nationwide cardiology database (IN-CHF Registry).[J]. American Heart Journal, 2002, 143(1):45-55.

10) Kawashiro N, Kasanuki H, Ogawa H, et al. Clinical characteristics and outcome of hospitalized patients with congestive heart failure: results of the HIJC-HF registry[J]. Circulation Journal Official Journal of the Japanese Circulation Society, 2008, 72(12):2015.

11) Stewart S, Macintyre K, Hole D J, et al. More 'malignant' than cancer? Five-year survival following a first admission for heart failure.[J]. European Journal of Heart Failure, 2014, 3(3):315-322.

12) Cowie M R, Wood D A, Coats A J, et al. Survival of patients with a new diagnosis of heart failure: a population based study.[J]. Heart, 2000, 83(5):505-10.

13) Mcmurray J J, Packer M, Desai A S, et al. Angiotensin-neprilysin inhibition versus enalapril in heart failure.[J]. New England Journal of Medicine, 2014, 371(11):993-1004.

14) Swedberg K, Komajda M, Böhm M, et al. Ivabradine and outcomes in chronic heart failure (SHIFT): a randomised placebo-controlled study.[J]. Lancet, 2010, 376(9758):2069-2069.

15) Li X, Zhang J, Huang J, et al. A multicenter, randomized, double-blind, parallel-group, placebo-controlled study of the effects of qili qiangxin capsules in patients with chronic heart failure[J]. Journal of the American College of Cardiology, 2013, 62(12):1065-1072.

16) Yiling WU. Meridian theory [M]. China Science and Technology Press, 2010.

17) Dingli XU. Effect of Qiliqiangxin Capsules on cardiac function and renal aquaporin-2 in rats with chronic heart failure. 2009 Xi'an National Heart Failure Academic Conference.

18) Jiazhen LI, Ping YANG. Study on intervention effect of Qiliqiangxin Capsules on periostin protein expression in rats with heart failure after myocardial infarction [J]. Chinese Journal of Laboratory Diagnosis, 2009, 13(2):170-172.

19) Ya LI, You SONG, Xiang CHENG, et al. Effects of Qiliqiangxin Capsules on myocardial remodeling and cardiac function after myocardial infarction in rats [J]. Chinese Journal of Molecular Cardiology, 2007, 7(4):201-204.

20) You SONG, Ya LI, Xiang CHENG, et al. Effect of Qiliqiangxin Capsules on regulation of TNF-α and IL-10 expression in myocardium of rats with acute myocardial infarction [J]. Chinese Journal of Immunology, 2007, 23(9):806-810.

1 Appendix 1: New York Heart Association (NYHA) Functional Classification

NYHA Class	Symptoms
I	No limitation of physical activity. Ordinary physical activity does not cause undue fatigue, palpitation, dyspnea (shortness of breath).
II	Slight limitation of physical activity. Comfortable at rest. Ordinary physical activity results in fatigue, palpitation, dyspnea (shortness of breath).
III	Marked limitation of physical activity. Comfortable at rest. Less than ordinary activity causes fatigue, palpitation, or dyspnea.
IV	Unable to carry on any physical activity without discomfort. Symptoms of heart failure at rest. If any physical activity is undertaken, discomfort increases.

2 Appendix 2: Echocardiographic Testing Index and Standard Operating Procedures

1. Personnel requirements

Ultrasound physicians participating in clinical trials must be familiar with the echocardiographic testing standard operating procedures (SOP).

2. Echocardiographic testing steps

The examination includes two-dimensional and Doppler echocardiography. Routine measurements are performed first, followed by dynamic imaging of five different planes including the left ventricular long axis view, left ventricular papillary muscle short axis view, apical four-chamber view, and apical two-chamber view. It is required to acquire 2-3 continuous cardiac cycles for each loop. Patients with atrial fibrillation are required to collect 5-10 cardiac cycles (3 consecutive cardiac cycles x 3 times).

1) Position: The patient should be in a supine or left lateral position for clear imaging.
2) Acoustic window location: Near the sternum, at the point of maximum heart apex pulsation, or at the 5-6th intercostal space on the midaxillary line.
3) Image adjustment: Adjusting the depth, gain, focus position, and pre/post processing properly. Applying techniques such as second harmonic and pseudo color improves image clarity and contrast, thereby clearly displaying the endocardium. The best possible image (requiring at least 80% of the endocardium) should be captured to facilitate tracing.
4) Image requirements: The left ventricular long axis view and M-mode curve should clearly display the interventricular septum, left ventricular cavity, and left ventricular posterior wall. The two-dimensional image of the apical four-chamber view should include all four heart chambers, with the apex at the top of the sector scan and the image centered. It is essential to fully expand all four chambers, especially the left ventricular cavity, and to clearly distinguish the left ventricular endocardial surface and the intersection of the atrial septum and the ventricular septum. The two-dimensional image of the apical two-chamber view

should include both the left and right ventricles, with the apex at the top of the sector scan and the image centered. The left ventricular cavity should be sufficiently expanded, and the left ventricular endocardial surface should be clearly distinguishable.

3. Parameter Measurement

1) Two-dimensional conventional measurement basic parameters: The left ventricular long axis view measures the end diastolic thickness of the interventricular septum and left ventricular posterior wall, the left ventricular end diastolic diameter and end systolic diameter, the end systolic left atrial anteroposterior diameter, and the interventricular septum and left ventricular posterior wall thickness.

2) Left ventricular systolic function evaluation using improved Simpson’s method:

- Apical four-chamber view: (1) left ventricular end diastolic volume (EDV4c); (2) left ventricular end systolic volume (ESV4c); (3) left ventricular ejection fraction (LVEF4c); (4) stroke volume (SV); and (5) cardiac output (CO).

- Apical two-chamber view: the measured indicators are the same as those in the apical four-chamber view.

3) The left ventricular end diastolic and end systolic inner membranes in the apical four-chamber view are delineated based on the QRS wave peak and T wave endpoint on the echocardiogram. The end diastolic volume of the left ventricle, end systolic volume, ejection fraction, stroke volume, and cardiac output are automatically calculated. These same values are calculated in the apical two-chamber view. Note: Papillary muscles should not be excluded from the delineation line.

The electrocardiogram is routinely connected, and the leads and gain are adjusted to the best display. Synchronous measurements of the subject's heart rate are taken. (If the instrument permits, the cardiac index (CI) is recorded based on parameters such as height and weight.)

4) Doppler examination records the forward flow velocity and regurgitation volume of each valve. If there is tricuspid regurgitation, accurately measure the peak velocity of the regurgitation and the pulmonary artery systolic pressure to avoid overestimating the pulmonary artery systolic pressure.

3 Appendix 3: Endpoint event report process

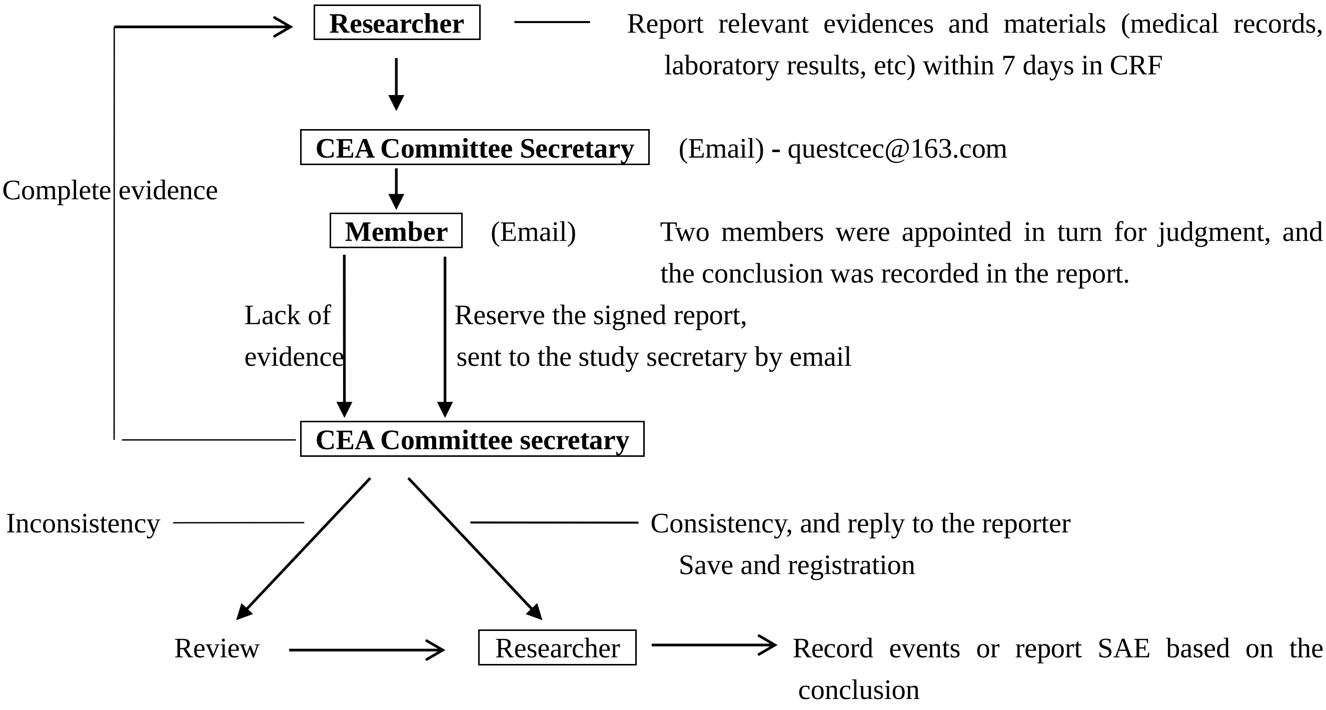

研究方案修订 V1.3，2018 年 11 月 2 日

1. 本次修订新增研究注册编号（ChiCTR1900021929）
2. 根据与研究分中心讨论的方案执行意见，对研究方案的内容进行订正，主要修改内容如下：
3. 目录索引标示订正；
4. “6.2.2 基础治疗”内容更新，入选患者拟按照《中国心力衰竭诊断和治疗指南 2018》进行规范药物治疗；
5. 整合研究中心意见，订正 “7.2. 安全性指标” 中观察的临床实验室指标为“血常规（血红蛋白、红细胞、白细胞、血小板）、尿常规（尿蛋白、尿白细胞、尿红细胞）、血清生化（尿素氮、肌酐、谷丙转氨酶、空腹血糖、钾、钠、氯、总胆固醇、甘油三酯）”；
6. 修订对“10.2. 不良事件强度判定标准”的描述，将不良事件严重程度进行分级；
7. 修订对“10.3. 不良事件与研究药物关系的判断标准”，
8. 新增“附 2：超声心动图检测指标和标准操作规程”，规范心脏超声检查的流程及参数测量；
9. 删除原“附 2:：NT-proBNP 检查采血及血样保存及运输流程”，NT-proBNP 的监测将在各分中心的进行；在分中心研究启动前，研究者需确定中心有标准化的 NT-proBNP 检测流程。

本研究方案所包含的内容为保密内容，是为临床研究人员使用。本资料为资助者或其子单位的财产，不得复印或分发给与本研究无关的人员。

芪苈强心胶囊对慢性心衰复合终点事件的评估研究

Qiliqiangxin in Heart FailUre：AssESsment of Reduction in MorTality

（**QUEST** 研究）

研究方案

Study Protocol

试验方案编号：SP-YFC-05-QUEST

试验方案版本号：V1.3

试验方案版本日期： 2018年11月2日

ChiCTR注册号：ChiCTR1900021929

课题承担单位：南京医科大学第一附属医院

课题负责人：李新立 教授

试验计划开始结束日期： 2018年8月--2021年12月

课题承担单位及负责人

主要研究单位	主要研究者
南京医科大学第一附属医院	李新立　　签名：__________________<br>日期：__________________

数据统计单位及负责人

单位	负责人
北京大学临床研究所	姚晨

药品生产企业及联系方式

单位	联系人	电话	邮箱
石家庄以岭药业股份有限公司	韩硕龙	13582167153	hanshuolong@yiling.cn

学术顾问

高润霖 院士、张伯礼 院士、张　运 院士、葛均波 院士、

韩雅玲 院士、黄从新 教授、托　尼 教授（哈佛大学）

数据和安全监测委员会

主席：陈　峰 教授

成员：朱　俊 教授　唐其柱 教授　马长生 教授　蔡廼绳 教授

事件判定委员会

主席：杨新春 教授

成员：宋　雷 教授　蔡廼绳 教授　施海明 教授　朱文玲 教授

缩略语

ACEI	血管紧张素转换酶抑制剂
ADR	可疑药物不良反应
AEs	不良事件
AHA	美国心脏学会
AMI	急性心肌梗死
ARB	血管紧张素受体拮抗剂
ARNI	血管紧张素受体脑啡肽酶抑制剂
BNP	B 型利钠肽
CEC	事件判定委员会
CGRP	降钙素基因相关肽
CHF	慢性心力衰竭
CRA	临床研究监查员
CRC	临床研究协调员
CRF	病历报告表
CV	心血管
DSMB	数据与安全监察委员会
DVP	数据核查计划
ECG	心电图
EF	射血分数
EOS	终末访视
ESC	欧洲心脏病学会
ET	内皮素
FAS	全分析集
HF	心力衰竭
LVOT	左心室流出量
MedDRA	ICH 国际医学用语词典
MI	心肌梗死
NO	一氧化氮
NT-proBNP	氨基末端 B 型利钠肽前体
NYHA	纽约心脏病学会
PPS	符合方案集
RAAS	肾素-血管紧张素-醛固酮系统
SAE	严重不良事件
SNS	交感神经系统
SS	安全性分析集
TVI	时间流速积分
UNS	计划外访视

研究方案摘要

**试验目的**	采用循证医学研究方法，以心血管死亡率和心衰恶化再住院发生率为主要研究终点，进一步阐明芪苈强心胶囊长期用药的临床疗效及安全性，明确疗效特点及适宜人群，为优化临床合理用药方案提供高质量临床证据
**试验设计**	随机、双盲、安慰剂平行对照多中心临床试验
**入选和排除标准**	**入选标准：**<br>1） 自愿参加，理解并签署知情同意书；<br>2） 年龄≥18 岁，性别不限；<br>3） 有 3 个月以上的慢性心衰病史或临床发现心衰症状 3 个月以上；慢性心衰诊断参照当地指南或中华医学会心血管病学分会发布的“中国心力衰竭诊断和治疗指南 2018”；<br>4） 心脏彩超检查提示左室射血分数（LVEF）≤40％（改良辛普森法）；<br>5） NYHA 心功能分级Ⅱ～Ⅲ，临床症状稳定，包括入选前 2 周内曾诊断为Ⅳ级者；<br>6） 血清 NT-proBNP 含量≥ 450pg/ml；<br>7） 至少已接受 2 周以上的标准化药物治疗，且未调整过给药剂量及给予静脉治疗者；标准化药物治疗包括：血管紧张素转换酶抑制剂（ACEI）或血管紧张素受体拮抗剂（ARB）或血管紧张素受体脑啡肽酶抑制剂(ARNI)、β受体阻滞剂及醛固酮受体拮抗剂（除非禁忌或不耐受，应达到最佳治疗剂量）；<br>**排除标准：**<br>1） 不符合入选标准；<br>2） 由于瓣膜病、先天性心脏病、心包疾病、心律失常所致及非心源性病因所致心衰，或肾、肝等重要脏器功能衰竭导致的心衰，及有明确肺源性或其他原因所致的右心衰、及急性心衰；<br>3） 计划于近期内行冠脉血运重建治疗或心脏再同步化治疗者，已实施心脏再同步化治疗者；<br>4） 合并肝、肾、造血系统等严重原发性疾病，肾功能异常者，肝脏转氨酶、碱性磷酸酶超出 3 倍正常值上限，血肌酐＞2mg/dl(176.82umol/L)，血钾＞5.5mmol/L；肿瘤患者，严重神经内分泌系统疾病及精神病患者；<br>5） 存在左室流出道梗阻、心肌炎、大动脉瘤、夹层动脉瘤、致明显血液动力学

	改变的未修补的心脏瓣膜病患者；<br>6） 存在心源性休克、难以控制的恶性心律失常、二度Ⅱ型以上未置入起搏器治疗的窦房或房室传导阻滞、进行性加重的不稳定心绞痛或急性心肌梗死者；<br>7） 未获控制的高血压患者，收缩压≥180/mmHg 和/或舒张压≥110mmHg；收缩压<90mmHg 和/或舒张压<60mmHg；<br>8） 1 个月内参加其他药物临床研究者；<br>9） 妊娠或正准备妊娠及哺乳期妇女；<br>10）过敏体质者，或已知对治疗药物过敏者；<br>11）根据研究者判断，患者不能完成本研究或不能遵守本研究的要求（由于管理方面的原因或其它原因）。	
**疗效性指标**	主要指标	心血管死亡和心衰恶化再住院组成的复合终点事件发生率
	次要指标	1、 全因死亡率<br>2、 复合终点事件（心衰恶化放弃治疗、心脏骤停后复苏成功、恶性心律失常、非致死性卒中）<br>3、 冠心病心衰患者的心血管死亡和心衰恶化再住院发生率<br>4、血清NT-proBNP下降率
**安全性指标**	血常规、尿常规，心电图，生化，不良事件，体格检查	
**样本量**	试验组与对照组的随机分配比例为 1:1。样本量为复合终点事件的发生例数。预计需要观察到 620 例复合终点事件。<br>假设随访期 36 个月内对照组复合终点事件的发生率为 25%，整个试验持续大约 36 个月，招募期预计 24 个月，则预计需要入组 3080 例（每组 1540 例）受试者才可获得 620 个终点事件。	
**给药方案**	试验组：慢性心衰标准化治疗+芪苈强心胶囊 4 粒/次，3 次/日，口服<br>对照组：慢性心衰标准化治疗+芪苈强心胶囊安慰剂 4 粒/次，3 次/日，口服	
**疗程**	以观察事件为指标入选病人，计划入组时间 2 年，至少服药 1 年。	
**试验统计**	北京大学临床研究所	
**预期进度**	2018 年 8 月-2021 年 8 月	

目录

1. 试验题目........8
2. 试验目的........8
3. 试验背景与原理........8
4. 试验总体设计与安排........11
4.1. 随机分组阶段（第 0 天~第 24 个月）........11
4.2. 病例数量、分组、中心........11
5. 研究人群........12
5.1. 入选标准........12
5.2. 排除标准........13
5.3. 中止研究药物治疗........13
5.4. 退出标准........14
5.5. 全面中止试验标准........14
6. 治疗........14
6.1. 试验用药物........14
6.1.1. 药物来源........14
6.1.2. 制剂、包装与标签........15
6.1.3. 保存方法........16
6.1.4. 药物的发放与回收........16
6.1.5. 药物清点........16
6.2. 治疗方案........16
6.2.1. 研究流程：........16
6.2.2. 基础治疗........16
6.2.3. 合并用药........17
6.2.4. 疗程：12 个月-36 个月。........17
6.3. 依从性评价........17
6.4. 药物不良反应........17
7. 临床观察终点和指标........17
7.1. 临床观察指标........17
7.1.1. 主要有效性终点........17
7.1.2. 次要有效性终点........17
7.2. 安全性指标包括：........18
8. 试验过程........18
9. 终点事件的含义及 CEC 判定规程........19
9.1. 本研究终点事件的含义........19
9.2. 终点事件发生时的评估与程序........20
10. 不良事件的观察........21
10.1. 定义：........21
10.2. 不良事件强度判定标准：........21
10.3. 不良事件与研究药物关系的判断标准........21
10.4. 严重不良事件的判定........22
10.4.1. 一般严重不良事件定义........22
10.4.2. 严重不良事件的研究特异性定义........22
10.5. 不良事件的随访与记录........23

10.6. 实验室结果异常 23
11. 盲法与随机化 23
11.1. 受试者随机分配方法 23
11.2. 紧急揭盲 24
11.3. 揭盲规定 24
11.4. 筛选编号 24
12. 统计分析 25
12.1. 分析人群 25
12.2. 统计分析方法 25
12.2.1. 入组及完成情况： 26
12.2.2. 依从性分析： 26
12.2.3. 疗效评价： 26
12.2.4. 安全性评价： 27
12.3. 期中分析 27
13. 数据管理 27
13.1. 数据库设计 27
13.2. 数据录入 27
13.3. 数据质疑管理 28
13.4. 医学编码 28
13.5. 数据审核 28
13.6. 数据库锁定 28
14. 质量控制 28
15. 伦理相关事宜 29
16. 试验进度 29
17. 资料保存 30
18. 临床总结 30
参考文献： 31
1 附 1：NYHA 分级评分标准 32
2 附 2：超声心动图检测指标和标准操作规程 32
3 附 3：终点事件报告流程 33

1. 试验题目

芪苈强心胶囊对慢性心衰复合终点事件的评估研究

2. 试验目的

采用循证医学研究方法，以心血管死亡率和心衰恶化再住院发生率为主要研究终点，进一步阐明芪苈强心胶囊长期用药的临床疗效及安全性，明确疗效特点及适宜人群，为优化临床合理用药方案提供高质量临床证据。

3. 试验背景与原理

在全球范围的死亡原因中心血管疾病位列前三，严重地危害着人类的生命和健康[1,2]。慢性心力衰竭（CHF）是在器质性心脏病基础上发生泵功能衰竭、射血分数减少、循环淤血，同时出现一系列神经体液改变的一系列临床综合症[3]。心衰作为各种心脏病发展的严重阶段，正在成为本世纪最重要的心血管病症。有流行病学资料显示，目前全球心衰患者的数量已高达 2250 万，且 5 年存活率与恶性肿瘤相仿。随着流行病学的变迁和社会经济的发展，发展中国家心衰的流行病学特点与发达国家日益相近，如冠心病作为心衰的病因在我国显得越来越突出[4,5]。欧洲心脏病学会（ESC）近年来通过对 51 个国家的统计发现，在约 10 亿的人群中，至少有 1500 万例心衰患者。2007 年美国心脏学会（AHA）报道，美国的心衰患者人数已经超过 500 万，并且仍以 55 万/年的速度不断增加[6]。与我国地理位置相近、种族人群特征相似的日本发病情况与欧美国家类似。2003 年，顾东风等在我国南方和北方各 5 个省市，随机抽样调查了 15518 名成年人（年龄 35～74 岁），分析发现我国心衰患病率为 0.9%，其中男性为 0.7%，女性为 1.0%[7]。近年来，我国与发达国家的心衰患者数量都在不断增加，全球患者以每年 200 万的速度递增，发病率和患病率也在逐步上升，其重要原因之一就是社会人口老龄化，随着年龄的增长心衰发病率升高[8]。而且随着治疗水平的发展，心衰患者死亡率虽较过去有所下降，但仍然处于较高水平。美国 70 岁以上的心衰患者 1 年死亡率明显较 70 岁以下患者高（22％：13.7％）[9]。日本的心衰患者 1 年和 3 年死亡率分别为 11.3％和 29.2％[10]，在欧洲，4 年生存率仅为 50％，而且有 40％因心衰入院的患者将可能在 1 年内再次入院治疗或者死亡[11,12]。慢性心衰依然是严重威胁人类生命和生活质量的主要问题，所以国际上仍将慢性心衰作为本世纪需要解决的重要课题。

近 20 年来，人们对心衰的药物治疗理念发生了极大转变，从改善血流动力学观点进展到生物学调整的观点。现代治疗模式的重点是改善肾素-血管紧张素-醛固酮系统及

交感神经系统的神经内分泌紊乱。因此，治疗的目标不仅仅是改善症状和提高生活质量，更应注重抑制和延缓心肌重构的发展，阻断恶性循环，从而降低心衰的死亡率和住院率。

心肌重构是心衰发生发展的基本机制，包括病理性心肌细胞肥大伴胚胎基因再表达、心肌细胞凋亡与坏死及心肌细胞外基质过度沉积或降解增加等。改善心肌重构对预防、控制心衰的发生、发展和改善心功能具有重要的价值。神经内分泌两个系统包括交感神经系统(SNS）、肾素-血管紧张素-醛固酮系统（RAAS）的激活和心肌重构相互促进、加重心衰的发展。ACEI、β 受体阻滞剂、ARB 和醛固酮受体拮抗剂的应用获得了有益效果，从而说明阻断这两个系统是有效的，进一步证实了对心衰的发生和发展的这一基本机制的认识是完全正确的。但心衰的发生发展机制仍有待于更深入的研究，从而发掘更多，更有效的抗心衰途径，在机制研究中推断治疗心衰的新靶点。

20 世纪 80 年代后期大量临床研究的开展，也证实了神经内分泌系统激活导致心肌重构是引起心衰发生和发展的关键因素。1987 年，应用血管紧张素转换酶抑制剂(ACEI)治疗心衰的临床试验 CONSENSUS，成功降低心衰患者总死亡率达 27%，以后 SOLVED、V-HeFY 等临床试验进一步证实 ACEI 能够有效改善心衰患预后。

20 世纪 90 年代中、后期的 CIBISII、MERIT-HF、COPERNICUS 研究证实β受体阻滞剂使心衰患者死亡率降低 34%～35%。此外，RALES 试验(1999 年)、EMPHASIS-HF(2011 年)研究显示醛固酮受体拮抗剂可使心衰患者死亡率降低 24%～30%。

2010 年以来能够给心衰患者带来获益的新药主要是 ARNI 和心脏窦房结抑制剂。PARADIGM-HF 试验中，8442 例射血分数降低的心衰(HFrEF)患者随机接受 ARNI 和依那普利，ARNI 组主要终点事件(心血管死亡和因心衰住院)发生率为 21.8%，显著低于依那普利组(26.5%)[13]。SHIFT 研究显示，与标准治疗组比较，伊伐布雷定组使心血管死亡和心衰恶化住院的相对风险降低 18%，患者左心室功能和生活质量均显著改善。[14] 同时，临床研究也发现利尿剂可有效缓解心衰患者的呼吸困难、消除液体潴留，改善心功能和运动耐量，合理恰当使用利尿剂是其他治疗心衰药物取得成功的关键和基础。

在此期间，心衰的非药物治疗也取得重要进展，在药物治疗基础上选择合适的患者，CRT 能进一步改善其心功能和生活质量，降低死亡率。

尽管在这些年心衰治疗领域取得了一些进展，但目前心衰的患病率和病死率依然居高不下。尚需要开拓新的治疗方法和研发新的药物以期在心衰治疗中取得突破。

中医学在防治心力衰竭的长期医疗实践中，积累了丰富的经验，对心衰的认识也在

不断深化。

中药治疗心衰已有一些研究和报道，中药芪苈强心胶囊治疗心衰研究初步获得成功，该研究结果于 2013 年发表于国际心血管病 JACC 杂志上[15]，并受到国外学者的关注和好评。所有入选者均采用了标准优化治疗，加用芪苈强心胶囊组较安慰剂组，血清氨基末端 B 型利钠肽前体（NT-proBNP）水平显著降低，且降幅＞30%的患者比率也显著增加，而不良反应发生率显著降低，表明芪苈强心胶囊治疗慢性心衰有效、安全，标志着中药成为心衰公认治疗药物向前迈进一大步。芪苈强心胶囊成为第一个列入《中国心力衰竭诊断和治疗指南 2014》的中成药。

芪苈强心胶囊是首次运用络病理论探讨慢性心衰的病机和治疗，提出心气虚乏是其发生的中医病机之本，络脉瘀阻是其中心环节，津液不循脉络运行渗出脉外而为水湿之邪发为水肿，瘀血水饮阻滞脉络，日久结聚成形导致心络“络息成积”是其发展加重的结果[16]，这与西医学近年提出的早期神经内分泌激活引起的血流动力学改变，进而导致心室重构是心衰发生发展的基本机制的新概念相吻合。

由于芪苈强心胶囊上市后在临床应用中显示出的独特优势，成为国内多名专家学者研究的热点。已有的研究表明该药有强心、利尿作用，可以改善 CHF 大鼠心脏功能，并且通过减少肾脏 AQP2 的表达，增加水的排出[17]。能够减少心梗后心衰大鼠的 Ang II、periostin 蛋白的表达，并具有剂量依赖性[18]。芪苈强心胶囊有效改善心功能的作用机制与其抑制心肌重构有关[19]。减少心肌细胞促炎因子和增加抗炎因子的免疫调节作用可能是中药芪苈强心改善 AMI 大鼠心功能的免疫药理机制之一[20]。在观察芪苈强心胶囊治疗对慢性充血性心力衰竭病人疗效的临床研究中，表明芪苈强心胶囊可以改善心衰患者心功能分级、Lee 氏心力衰竭计分、心脏收缩和舒张功能、射血分数(EF)、中医证候和生活质量等疗效指标及安全性指标，升高血一氧化氮(NO)、降钙素基因相关肽（CGRP）水平，降低其内皮素(ET)水平，从而明显改善心力衰竭患者的内皮功能。

综上所述，芪苈强心胶囊全方标本兼治，从多途径、多环节、多靶点治疗心功能不全，体现了复方中药在治疗心力衰竭方面从整体论治的优势，明显降低 NT-proBNP 水平，这意味着患者的长期预后可能有所改善。本研究是在芪苈强心胶囊以 NT-proBNP 为替代终点并取得重大研究成果的基础上，继续深入开展的以心血管死亡和心衰再住院组成的复合终点事件发生率为研究终点，以获得对慢性心衰患者长期预后循证医学研究证据的大型随机、双盲、多中心临床研究。

4. 试验总体设计与安排

本研究是一项在慢性心衰患者中进行的随机、双盲、安慰剂对照、平行分组的多中心临床研究。

本研究将为事件驱动型，全部随机入组的患者将保留在研究之中（无论是否服用研究药物），直至主要终点事件的发生数目达到预计（620 例），或者当满足事先定义的提前终止的疗效或安全性标准时，研究提前终止。

计划在发生 1/2、2/3 主要终点事件后进行两次期中疗效分析，以评估是否已得出无效或有效的结论而提前终止该研究。

计划整个研究将持续大约 36 个月，招募期预计 24 个月，最后一例患者入组研究后的随访期为 12 个月。预计平均随访时间约为 24 个月。

在医院开始筛选患者，临床症状稳定，入选之前已接受至少 2 周标准化方案治疗并治疗其他伴随疾病。根据当地 HF 治疗指南规定用药，药物种类、剂量固定，除非禁忌或不耐受，且此期间未静脉用药，未服用与芪苈强心胶囊成分相似中药、中成药的患者直接进入随机分组阶段。

若达不到上述要求，则可先行标准化治疗达到上述标准后再进入随机分组阶段。

4.1. 随机分组阶段（第 0 天~第 24 个月）

*请注意：*接受 2 周以上的标准化治疗方案且未使用中药治疗符合入选标准的受试者进入随机分组阶段。此期间的每一位患者使用药物种类、剂量需要固定。若属医疗需要调整用药，需记录在病例报告表中。

患者将按照 1:1 的比例随机化到试验组或对照组。患者将在当前慢性心衰标准化治疗的基础上使用研究药物。

试验组：慢性心衰标准化治疗+芪苈强心胶囊（4 粒/次，3 次/日，口服）；

对照组：慢性心衰标准化治疗+芪苈强心胶囊安慰剂（4 粒/次，3 次/日，口服）。

治疗期间应避免使用其他中药或中成药（与芪苈强心功能组成相似的中药）

患者应于随机分组后第 1 个月、第 3 个月、第 6 个月，第 9 个月，第 12 个月，此后每隔 3 个月来医院访视，进行有效性和安全性评估，直到研究全部结束。随机分组阶段共 24 个月。

4.2. 病例数量、分组、中心

参考 PARADIGM-HF 研究，中位随访 27 个月 LCZ696 组患者的心血管死亡或心衰住院

率为 21.8%，而依那普利组为 26.5%。所以我们估算基础治疗+安慰剂组随访 36 个月内，所有患者的心血管死亡和心衰住院事件发生率为 25%，基础治疗+芪苈强心组发生率为 20%。

试验组与对照组的随机分配比例为 1:1，考虑到期中分析对 I 类错误的消耗，α 调整为单侧 0.02314。样本量为复合终点事件的发生例数。预计需要观察到 620 例复合终点事件，才能提供 80%的把握度（β=0.2），经过 log-rank 检验得到试验组可以降低 20%风险的结论。

假设随访期 36 个月内对照组复合终点事件的发生率为 25%，整个试验持续大约 36 个月，招募期预计 24 个月，则预计需要入组 3080 例（每组 1540 例）受试者才可获得 620 个终点事件。

因此本研究计划纳入 3080 例患者，患者将以患者将以 1:1 的比例分配至试验组与对照组，并计划在约 100 个中心进行。

5. 研究人群

入组患者必须满足下文所列的所有入选标准，并且不符合任何一项排除标准。除下文所列标准外，接受标准化治疗期间，如果存在任何禁忌的医学状况或使用禁忌药物，也是排除患者入选的标准。

5.1. 入选标准

1) 自愿参加，理解并签署知情同意书；
2) 年龄≥18 岁，性别不限；
3) 有 3 个月以上的慢性心衰病史或临床发现心衰症状 3 个月以上；慢性心衰诊断参照当地指南或中华医学会心血管病学分会发布的“中国心力衰竭诊断和治疗指南 2018”；
4) 心脏彩超检查提示左室射血分数（LVEF）≤40％（改良辛普森法）；
5) NYHA 心功能分级Ⅱ～Ⅲ，临床症状稳定，包括入选前 2 周内曾诊断为Ⅳ级者；
6) 血清 NT-proBNP 含量≥ 450pg/ml；
7) 至少已接受 2 周以上的标准化药物治疗，且未调整过给药剂量及给予静脉治疗者；标准化药物治疗包括：血管紧张素转换酶抑制剂（ACEI）或血管紧张素受体拮抗剂（ARB）或血管紧张素受体脑啡肽酶抑制剂(ARNI)、β受体阻滞剂及醛固酮受体拮抗剂（除非禁忌或不耐受，应达到最佳治疗剂量）

5.2. 排除标准

1) 不符合入选标准；
2) 由于瓣膜病、先天性心脏病、心包疾病、心律失常及非心源性病因所致心衰，或肝、肾等重要脏器功能衰竭导致的心衰；及有明确肺源性或其他原因所致的右心衰、及急性心衰；
3) 计划于近期内行冠脉血运重建治疗者或心脏再同步化治疗者，已实施心脏再同步化治疗者；
4) 合并肝、肾、造血系统等严重原发性疾病，肾功能异常者，肝脏转氨酶、碱性磷酸酶超出 3 倍正常值上限，血肌酐＞2mg/dl(176.82umol/L)，血钾＞5.5mmol/L；肿瘤患者，严重神经内分泌系统疾病及精神病患者；
5) 存在左室流出道梗阻、心肌炎、大动脉瘤、夹层动脉瘤、致明显血液动力学改变的未修补的心脏瓣膜病患者；
6) 存在心源性休克、难以控制的恶性心律失常、二度Ⅱ型以上未置入起搏器治疗的窦房或房室传导阻滞、进行性加重的不稳定心绞痛或急性心肌梗死者；
7) 未获控制的高血压患者，收缩压≥180/mmHg 和/或舒张压≥110mmHg；收缩压<90mmHg 和/或舒张压<60mmHg；
8) 1 个月内参加其他药物临床研究者；
9) 妊娠或正准备妊娠及哺乳期妇女；
10) 过敏体质者，或已知对治疗药物过敏者；
11) 根据研究者判断，患者不能完成本研究或不能遵守本研究的要求（由于管理方面的原因或其它原因）。

5.3. 中止研究药物治疗

随机分组后，任何原因暂停研究药物不等于永久停用，也不应该导致患者退出整个研究。相反对于已经停止服用研究药物的患者，也应该参加所有方案规定的研究访视和评价项目。如果患者不能参加研究访视，应按照计划通过电话继续随访，以确定是否发生任何不良事件和终点事件，除非患者拒绝随访并撤回知情同意书。

出现以下情况时可中止研究药物治疗：

1. 患者可在任何时间中止治疗
2. 发生与研究药物明确相关的过敏反应

3. 发生与研究药物明确相关的不良症状或体征、异常检查结果，研究者判断须终止研究的情况
4. 女性于研究期间发生妊娠

试验过程中应尽可能使患者长期服用标准剂量研究药物，中止研究药物患者在排除相关原因后应尽早恢复服用研究药物并按计划进行随访。

5.4. 退出标准

所有填写了知情同意书并筛选合格进入试验的受试者，无论何时何因退出，均不会影响其后续治疗。

患者有权在任何时间以任何理由退出研究，但应当尽量避免不必要的患者退出，并积极采取措施，尽可能完成随访，以备对其疗效和安全性进行分析。但当患者决定退出时，研究者应当通过电话或个人访问形式联系患者或其责任亲属并尽可能确认退出原因，研究者应当在患者退出时回收剩余药物，完成最终评估，尽可能完成病例报告、解释退出原因，对退出患者发生终点事件进行随访。如果患者退出的原因为不良事件，则应记录于 CRF 内。

5.5. 全面中止试验标准

1) 研究进行中由于以下原因整个试验在多中心全面停止：
   - 基于 DSMB 中期分析结果建议；
   - 研究者发现严重安全性问题；
   - 方案有重大失误；
   - 资助方因经费或管理原因；
   - 行政主管部门撤消试验，均可中途停止全部试验。
2) 全面中止试验可是暂时的，也可是永久的。中止试验时，全部试验记录应予保留备查。

6. 治疗

6.1. 试验用药物

6.1.1. 药物来源

试验药品名称：芪苈强心胶囊 Qiliqiangxin Jiaonang

成份：黄芪、人参、附子、丹参、葶苈子、泽泻、玉竹、桂枝、红花、香加皮、陈皮。

性状：胶囊剂，内容物为棕褐色至黑褐色的颗粒，味苦。

规格：0.3g/粒

批号：国药准字 Z20040141

生产单位：石家庄以岭药业股份有限公司

安慰剂：芪苈强心胶囊模拟剂（模拟剂与试验药品在颜色、规格、包装、标签、内容物形状等方面完全一致）

上述所有试验药物、模拟剂均由石家庄以岭药业股份有限公司免费提供，并出具合格药检报告。

6.1.2. 制剂、包装与标签

芪苈强心胶囊将以 0.3g 胶囊制剂形式提供，安慰剂外观上与其完全一致。

药物包装

小包装：外观如下，印有“临床试验用药”字样，每小盒内以铝塑板装 36 粒药。

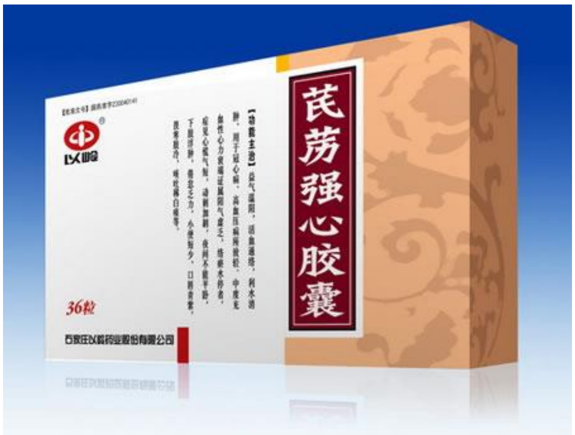

大包装：为 29.5cm×12cm×22cm 的白板纸盒，每一个大包装内含 33 个小包装，每个大盒贴有如下标签：

**芪苈强心胶囊对慢性心衰复合终点事件的评估研究用药**<br>**（仅供临床研究使用）**
**药物包装号：XXXXX**<br>【产品批号】xxxx　　【有效期至】xxxx
【国药准字】Z20040141<br>【包　　装】每小盒装 36 粒，内装 33 小盒（一名受试者 99 天用量）<br>【用法用量】每日 3 次，每次 4 粒<br>【贮　　存】密封，在干燥处、儿童不易触及处保存
**请您务必遵照医生的医嘱和指定日期到医院就诊访视，谢谢合作！**

6.1.3. 保存方法

试验药物应当在安全可控的室内区域内上锁保存，注意防潮。每个试验中心必须指定研究用药管理负责人保存、管理。

6.1.4. 药物的发放与回收

每次随访研究者将药物发给受试者时，需填写药品发放登记表，及时、准确记录发放的药品数量并提醒受试者下次就诊时将其剩余药物带回；回收并核对返还药品的数量，记录于药品发放/返还登记表中。试验结束时，全部剩余药品应返还给申办者，并填写研究用药回收表格，未发放的药品在返还时必须是密封的。试验结束后剩余药物由监查员回收，统一处理。

6.1.5. 药物清点

自服药后受试者应按照访视计划归还所有剩余药品，研究者清点剩余药品数量并记录，用以判断依从性。

6.2. 治疗方案

6.2.1. 研究流程：

入组期（第-14 天~第 0 天）：

患者如果符合本方案中标准治疗的规定，可在此期间完成所有检查，符合入选、排除标准的患者进入随机分组阶段。

随机治疗期（第 0 天~12 个月（最长 36 个月））：（以发药时间记为 0 天）

试验组：慢性心衰标准化治疗+芪苈强心胶囊（4 粒/次 3 次/日 口服）

对照组：慢性心衰标准化治疗+芪苈强心胶囊模拟剂（4 粒/次 3 次/日 口服）

研究药物推荐于每日三餐后约 30 分钟时服用。访视日早晨勿服用药物。如果患者某日未服药，次日服药剂量不得超过日剂量，本研究不允许进行剂量调整。如果患者出现无法耐受的不良事件，并且根据研究者考虑与研究药物相关，则患者应当终止研究药物治疗。

6.2.2. 基础治疗

参照当地 HF 治疗指南或中华医学会心血管病学分会发布的“中国心力衰竭诊断和治疗指南 2018”规定如下：

1) 入选前至少 2 周以上未静脉使用利尿剂、强心剂及血管扩张剂。
2) 进入随机分组阶段至少 2 周前，患者应当接受慢性心衰标准化治疗，所有药物已经调整至固定剂量，标准化药物治疗包括：血管紧张素转换酶抑制剂（ACEI）或血管

紧张素受体拮抗剂（ARB）或血管紧张素受体脑啡肽酶抑制剂(ARNI)、β受体阻滞剂及醛固酮受体拮抗剂（除非禁忌或不耐受，应达到最佳治疗剂量）；

3) 进入治疗期后每一位患者使用药物种类、剂量需延续入选前标准化治疗方案。整个治疗期间原则上不能再调整；若属医疗需要调整用药，需记录在病例报告表中，增加或减少药物剂量、种类的患者需记录复合终点事件和不良事件。

6.2.3. 合并用药

1) 接受较好地控制高血压、心绞痛、糖尿病或其他疾病的药物治疗。
2) 进入随机分组阶段之后整个治疗期均不得使用研究药物以外的其他与试验药物成分类似中药。
3) 患者应当接受有利于心脏健康的饮食指导，如低盐饮食，适量饮水等，患者同时应当接受诸如监测体重、体育锻炼、戒烟、戒酒等适当生活方式改善的咨询。
4) 目前尚未发现禁止与芪苈强心胶囊伴随使用的药物。

6.2.4. 疗程：12 个月-36 个月。

6.3. 依从性评价

通过完整记录药物的分发和回收情况来对受试者进行依从性评价，实际服用药物量在应用药物量的 80%~120%范围内，可判定为用药依从性符合方案要求。

6.4. 药物不良反应

目前，尚未发现芪苈强心胶囊存在明显的不良反应。

7. 临床观察终点和指标

7.1. 临床观察指标

7.1.1. 主要有效性终点

- 心血管死亡和心衰恶化再住院组成的复合终点事件发生率；

7.1.2. 次要有效性终点

- 全因死亡率
- 复合终点事件（心衰恶化放弃治疗、心脏骤停后复苏成功、恶性心律失常、非致死性卒中）
- 冠心病心衰患者的心血管死亡和心衰恶化再住院发生率
- 血清 NT-proBNP 下降率

注：全部终点事件需经事件判定委员会复核判定。

7.2. 安全性指标包括：

- 不良事件评价
- 临床实验室指标：血常规（血红蛋白、红细胞、白细胞、血小板）、尿常规（尿蛋白、尿白细胞、尿红细胞）、血清生化（尿素氮、肌酐、谷丙转氨酶、空腹血糖、钾、钠、氯、总胆固醇、甘油三酯）。
- 12 导联心电图
- 体格检查

8. 试验过程

所有患者包括研究完成前停用研究药物，都应继续参加表格所列的计划访视，直至研究结束。如果某次访视被推迟或者提前，不应影响下次访视。下次访视应遵守原计划时间进行。

试验流程表

访视	1	2	3	4	5	6	7	8	9	10	11	12	13	14	UNS	EOS
天/月	0天	1M	3M	6M	9M	12M	15M	18M	21M	24M	27M	30M	33M	36M		
	-14	±3	±7	±7	±7	±7	±7	±7	±7	±7	±7	±7	±7	±7		≦2周
知情同意书	X															
入选/排除标准	X															
中央随机	X															
一般资料/病史	X															
心衰病史	X															
体格检查	X	X1	X1	X1	X1	X	X1	X1	X1	X	X1	X1	X1	X	(X)	X
心衰用药	X	X	X	X	X	X	X	X	X	X	X	X	X	X	(X)	X
CV用药	X	X	X	X	X	X	X	X	X	X	X	X	X	X	(X)	X
其他用药	X	X	X	X	X	X	X	X	X	X	X	X	X	X	(X)	X
心脏彩超	X*															
血/尿常规	X	X				X				X				X	(X)	X
生化检查	X	X				X				X				X	(X)	X
妊娠试验	X					X				X				X	(X)	X
12导联心电图	X*	X				X				X				X	(X)	X
血清NT-proBNP	X	X	X											X		
分发药物	X		X	X	X	X	X	X	X	X	X	X	X	X		
回收药物			X	X	X	X	X	X	X	X	X	X	X	X		X
终点事件		X	X	X	X	X	X	X	X	X	X	X	X	X	(X)	X
AE		X	X	X	X	X	X	X	X	X	X	X	X	X	(X)	X
SAE		X	X	X	X	X	X	X	X	X	X	X	X	X	(X)	X

- ×1为简化体格检查
- ×*心脏彩超和 12 导联心电图接受入组前 6 个月内结果。
- UNS(计划外访视中):（×）标记项目为可选择操作，根据研究者判断进行。
- EOS（终末访视）:根据研究结束时间安排进行（如在试验结束前一个月内有过访视则视为终末访视但需补充完整终末访视所需项目）。
- 妊娠试验仅适用于育龄妇女（如尿妊娠试验阳性则必须进行血清妊娠试验证实）。

9. 终点事件的含义及CEC判定规程

9.1. 本研究终点事件的含义

- **心力衰竭住院是指满足下列所有标准的事件：**

1.患者因初步诊断为HF住院

2.患者住院时间延长至少24小时（或者如果不能获得住院时间和出院时间，指在日历上日期发生的变化）

3.在患者报告上记录由于HF出现新症状或者恶化症状，至少包括以下情况之一：

a.呼吸困难（用力时呼吸困难、休息时呼吸困难、端坐呼吸、夜间阵发性呼吸困难）

b.运动耐量减少

c.疲劳

4.患者具有新出现的恶化HF的客观证据，包括至少两种体检结果或一种体检结果和至少一种实验室标准，包括：

a.判断由HF导致的体检结果，包括新出现或恶化的：

1)外周性水肿。 2)腹胀或腹水增加（在无原发性肝病的情况下）。 3)肺啰音/爆裂音/湿性啰音。 4)颈静脉压升高和/或肝颈静脉回流。 5)S3 奔马律。 6)具有临床意义的或迅速的体重增加，考虑与体液潴留有关

b.在24小时内获得的，新出现或者恶化HF的实验室证据，包括：

1)与HF失代偿一致（如BNP>500pg/ml或NT-proBNP>2000pg/ml）的B型利钠肽（BNP）/N末端B型利钠肽前体（NT-proBNP）浓度增加。在利钠肽长期升高的患者中，应特别关注超过基线的显著增加。 2)肺充血的放射影像学证据。3)具有临床意义的左侧或者右侧心室充盈压升高或者心输出量降低的非侵害性诊断证据。如超声心动图标准包括：E/e´>15或者D主导肺静脉流入模式，充血性下腔静脉伴有极小程度吸气塌陷，或者左心室流出量（LVOT）微小行程距离减小（时间流速积分（TVI））。4)侵入性诊断证据：右心导管检查显示肺毛细血管楔压（肺动脉闭塞压）≧18mmHg，中央静脉压≧12mmHg，或心排血量指数＜2.2L/min/m^2。

注：如适用，即使不满足上述标准，仍需报告诊断检查中的所有结果，为上述事件的裁定提供重要信息。

5.患者接受针对HF的初期或者强化治疗，包括下列至少一种：

a.增强口服利尿药的治疗

b.静脉注射利尿药或者血管活性药物（如正性肌力药、血管升压类药物或者血管扩张剂）

c.机械或手术干预，包括：

1)机械循环支持（例如，主动脉球囊反搏、心室辅助装置、体外膜氧合、全人工心脏）

2)机械辅助去除体液（例如，超滤、血液滤过、透析）

- **心血管死亡：**包括急性心肌梗死（MI），心源性猝死，心力衰竭（HF）导致的死亡，中风导致的死亡，心血管（CV）手术导致的死亡，CV 出血以及其他 CV 原因造成的死亡。
- **全因死亡**
- **心衰恶化放弃治疗：**心衰症状和体征不断加重，需要静脉药物或机械支持治疗而患者或患者家属主动放弃治疗或自动出院，若随访其后果为死亡则列入心力衰竭死亡。
- **心脏骤停后复苏成功**
- **恶性心律失常：**对于恶性心律失常的定义， 目前还没有统一的标准，一般是指能在短时间内引起严重血流动力学障碍，导致患者晕厥甚至猝死的心律失常 。根据这个标准，恶性心律失常主要有如下类别：(1) 严重的缓慢型心律失常， 如严重的病态窦房结综合征、高度或三度房室传导阻滞；(2) 快速型心律失常， 如持续性室性心动过速、心室扑动、心室颤动， 快室率心房扑动、心房颤动、房室折返性心动过速、预激综合征伴心房颤动、窦性心动过速等。
- **非致死性卒中**

9.2. 终点事件发生时的评估与程序

研究者获知终点事件发生后，应在 7 天内收集相关支持文件报告事件判定委员会。终点事件将由独立的事件判定委员会（CEC）进行复核，因此终点事件报告表将作为 CRF 的一部分，研究者将在上述表格内记录事件并及时提交支持文件（入院与出院记录、病历记录、死亡记录、ECG 等）上述资料将提供给 CEC 以对事件进行判定。

CEC 由主席及 5-6 名成员组成，每一例事件将由委员会的两位成员进行独立审查并将结论提交至委员会主席处。如果两位审查委员之间或两位审查委员与主席的意见不一致，当具有异议的事件积累到一定数量时，整个委员会将安排会议对事件进行审

查。

10. 不良事件的观察

10.1. 定义：

- 不良事件（**AEs**）：自受试者签署知情同意书并入选试验后开始至最后一次随访之间，发生任何不利医疗事件，无论与试验药物是否有因果关系，均判定为不良事件。
- 重要不良事件：除严重不良事件外，发生的任何导致针对性医疗措施（如停药，降低剂量和对症治疗）的不良事件和血液学和/或其他实验室异常。

10.2. 不良事件强度判定标准：

在本临床研究中发生的所有临床不良事件将记录在 CRF 不良事件页上。并将不良事件严重程度进行分级。为统一标准，事件严重程度分级如下：

*严重程度分级：

1 级　轻度，无临床症状或有轻微临床症状；仅临床或实验室检查异常；不需治疗。

2 级　中度，需要微量的、局部的或非侵害性的治疗；与年龄相符的使用工具的日常生活活动[a]受限，使用工具的日常生活指做饭、购物、打电话等。

3 级　病情重或有医学上严重的症状但是暂时不会危及生命；导致住院或住院时间延长；导致残疾；日常生活自理[b]受限。日常生活自理指：洗澡、穿衣、脱衣、吃饭、去卫生间、吃药等，非卧床不起。

4 级　危及生命，需要紧急治疗。

5 级　因不良事件致死。

a：工具性日常生活活动是指做饭，购买日常用品或衣服，使用电话，理财等。

b：自理性日常生活活动是指洗澡，穿/脱衣，吃饭，盥洗，服药，并未卧床不起。

10.3. 不良事件与研究药物关系的判断标准

对所有不良事件与试验药物关系的因果分析，均按肯定有关、可能有关、可能无关、肯定无关、无法判定五级进行判断，对前三种定为药物的不良反应。因果分析的考虑因素有以下五个方面：

1) 肯定有关：AE 的发生和试验药物的使用有合理的时间顺序，AE 为试验药物已知的不良反应，停药后 AE 减轻或消失，再次用药重复出现，并无法用受试者

本身疾病来解释。

2) 可能有关：AE 的发生和试验药物的使用有合理的时间顺序，AE 为试验药物已知或疑似的不良反应，但是，有其他因素可能引起该事件，如疾病、合并用药等；试验药物停用后反应减轻或消失，或药物停用后的效果不清楚，不清晰或缺乏决定性的信息。
3) 可能无关：AE 的发生和试验药物的使用有合理的时间顺序，但该事件不属于已知的药物不良反应类型，并极可能由受试者疾病或其他治疗引起。
4) 肯定无关：AE 的发生和试验药物的使用无合理的时间顺序，如事件在试验药物使用前已发生；不属于已知的药物不良反应；或 AE 确由其他因素导致，例如：受试者疾病、其他治疗或者合并用药引起等。
5) 无法判定：AE 出现的时间与用药的时间顺序无明确关系，不良事件与试验药物已知的反应类型相似，同时使用的其他药物也可能引起相同的反应，没有足够的依据判断。

以“肯定有关”、“可能有关”、“无法判定”三者合计为试验药物的不良反应，并据此计算不良反应发生率。

10.4. 严重不良事件的判定

10.4.1. 一般严重不良事件定义

严重不良事件是指任何提示显著危害、禁忌症、副作用或者需谨慎的临床事件。不良事件符合下面一条或以上标准时归为严重不良事件：

- 死亡
- 有生命危险（指出现该事件的患者在事件发生当时存在立即死亡的风险；并不包括那些如果更加严重将有可能导致患者死亡的事件）
- 导致住院或住院时间延长
- 导致持久或显著的劳动力丧失或残疾
- 先天性畸形缺陷

有些还没有导致死亡、生命危险或需住院的医疗事件，经过适当的医学判断，认为其可能对病人或受试者造成危害或需药物或外科手术治疗以避免上述情况发生时，也应视为 SAE。

10.4.2. 严重不良事件的研究特异性定义

在本试验中，下列事件将不会作为严重不良事件报告，除非被判定阴性并且研究者

认为与研究用药相关

- ◆ 心血管死亡
- ◆ 心衰恶化再住院
- ◆ 心衰恶化放弃治疗
- ◆ 心脏骤停后复苏成功
- ◆ 恶性心律失常
- ◆ 非致死性卒中

但是，所有其他导致致死性结局的事件都将被作为严重不良事件报告。

10.5. 不良事件的随访与记录

出现的不良事件，尤其是那些与试验药物相关的事件应当随访直至它们恢复至基线状态或者趋于稳定。如果经过随访，仍无法恢复基线状态或者稳定，那么应当在 CRF 中记录说明。临床试验过程中的任何严重不良事件，必须在 24 小时内报告临床试验监查人员、主要研究单位、药品生产企业。同时研究者必须填写严重不良反应表，记录严重不良事件的发生时间、严重程度、持续时间、采取的措施和转归。

10.6. 实验室结果异常

研究者应当对实验室结果异常是否具有临床意义进行判断，并给出可能的解释。已经被报告的不良事件导致的实验室异常结果应同时作为不良事件记录在不良事件表中。具有临床意义的实验室检查异常满足以下一项或多项条件者，应作为独立诊断记录在 CRF 的不良事件页中（不包括已被报告的不良事件导致的实验室结果异常）：

- 伴有临床症状的
- 导致研究用药改变的
- 需要改变合并用药和（或）其他治疗措施的

11. 盲法与随机化

11.1. 受试者随机分配方法

由北京大学临床研究所统计专业人员，在计算机上用 SAS9.4 统计软件包，按试验组与对照组 1:1 的比例用区组随机化方法生成随机编号。根据此随机编号由与本试验无关的人员对研究药物（芪苈强心胶囊或安慰剂）进行包装编码。

本研究采用随机化与试验药物管理系统（RTSM），统计专业人员将向RTSM提供随机

编号列表。然后，由RTSM给患者分配随机编号。

在完成基线评估后，在基线访视通过RTSM分配随机编号。此后，按照访视计划通过RTSM获取药物编号，每次分配的药物编号均不相同，但药物是同一种。在患者随机分组前，研究者必须先登录RTSM，并且提供一些信息（例如受试者出生日期、性别等）。

11.2. 紧急揭盲

如果发生不良事件，只有在必须了解研究药物的使用才能治疗患者的特殊情况下，才能进行紧急揭盲。一旦决定揭盲，研究者必须记录日期、时间和破盲的原因。

研究者需要登录 RTSM 填写破盲申请，由主要研究者审核后再由破盲员破盲。一旦破盲，该病例将中止研究，作为退出处理。

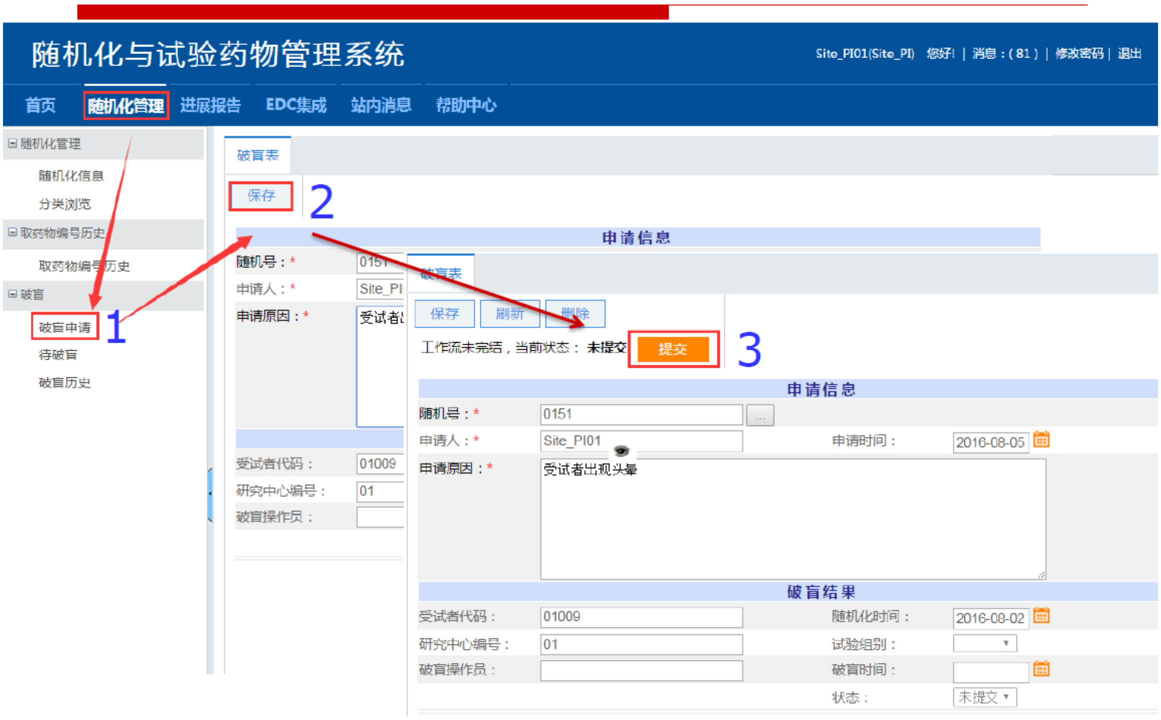

11.3. 揭盲规定

本研究采用二次揭盲法。在经盲态核查后，数据锁定，由主要研究者、医学统计专家、数据管理员、申办单位代表进行第一次揭盲，将各随机号所对应的组别以 A，B 为代号标出，以便对全部数据进行统计分析。当统计分析结束，统计报告完成时，再进行第二次揭盲，宣布 A，B 两组的确切组别。

11.4. 筛选编号

各医院按收治患者的先后顺序编排筛选号，筛选号由中心编号+3 位整数表示，如，01 中心筛选号 01001，01002，……。

12. 统计分析

试验方案确定后，由统计专业人员负责与主要研究者协商制订统计分析计划书。统计分析软件采用 SAS®9.4 软件（或更高版本）。样本量计算软件采用 PASS13。

12.1. 分析人群

研究人群分为以下几类：

- 全分析集(FAS)：是指尽可能接近意向性分析原则（intention to treat）、从所有随机化的受试者中，以最少的和合理的方法剔除受试者后得出的数据集，包含所有经过随机化并使用过一次研究药物的受试者。剔除通常包括：违反重要入组标准；受试者未接受试验用药物的治疗；随机化后无任何观测数据。主要疗效评价指标为发生复合终点事件的时间，采用生存分析的方法进行分析，在选择 FAS 进行统计分析时，对于主要终点事件的缺失按照删失处理。
- 符合方案集(PPS)：是全分析集的一个子集，这些受试者对方案更具依从性。纳入 PPS 受试者一般具有以下特征：（1）完成事先设定的试验药物的最小暴露量，即服用药物的依从性达到 80%；（2）试验中主要指标的数据均可以获得；（3）未对试验方案有重大的违背。
- 安全性分析集（SS）：所有随机化后至少接受一次治疗且有安全性评价的受试者。安全性缺失值无需结转。

疗效分析将在 FAS 和 PPS 的基础上进行。所有基线人口统计学资料分析将在 FAS 的基础上进行，安全性评价将在 SS 上进行。

12.2. 统计分析方法

- 所有的统计检验均采用双侧检验，*P* 值小于或等于 0.05 将被认为所检验的差别有统计意义。（特别说明的除外）
- 描述性分析：分类指标描述各类的例数及百分数。定量指标采用均数、标准差、最大值、最小值、中位数、下四分位数（Q1）和上四分位数（Q3）描述。
- 对两组一般情况的比较将根据指标的类型采用适当的方法进行分析，定量资料的组间比较采用成组 t 检验或 Wilcoxon 秩和检验，分类数据采用卡方检验或精确概率法，等级资料采用 Wilcoxon 秩和检验或 CMH 检验。

12.2.1. 入组及完成情况：

总结各中心入组及完成数，列出脱落病例的清单。各组不同数据集大小，各中心病例分布，总脱落率比较，终止原因详细列表。对患者的人口学特征(年龄、身高、生命体征等)、病史及用药史等进行描述，并对两组年龄、身高、体重等进行比较，以衡量两组的可比性。

12.2.2. 依从性分析：

- 用药依从性分析：比较两组病人是否按时按量使用试验药物，未用方案中禁用的药物和食物。
- 合并用药分析：需统计各组合并用药人数，并详细列表。

12.2.3. 疗效评价：

- 疗效评价同时进行 PP 分析和 FAS 分析；
- 主要疗效评价指标为发生复合终点事件（心血管死亡和心衰恶化再住院）的时间。对于主要终点事件的缺失按照删失处理。

  本研究的主要研究假设为：

  H_0： $\lambda_T/\lambda_C \geq 1$

  H_1： $\lambda_T/\lambda_C < 1$

  其中， λ_T和 λ_C分别为试验组和对照组发生终点事件的风险。

  利用 Kaplan-Meier 法估计临床终点事件发生率，两组之间进行 Log rank 检验。利用 COX 比例风险模型，以中心为协变量，计算两组间的风险比（Hazard Ratio）及其 95%可信区间。另外，对复合终点事件的两个部分分别进行分析，即心血管死亡和心衰恶化再住院。
- 次要疗效指标：

  全因死亡率：利用 Kaplan-Meier 法估计两组全因死亡率，并进行 Log rank 检验。利用 COX 比例风险模型，以中心为协变量，估计两组间的风险比（Hazard Ratio）及其 95%可信区间。

  复合终点事件（心衰恶化放弃治疗、心脏骤停后复苏成功、恶性心律失常、非致死性卒中）：分析方法与全因死亡相同。

  冠心病心衰患者的心血管死亡和心衰恶化再住院事件：按计数资料统计分析；

  血清 NT-proBNP：按计量资料分析，对两组血清 NT-proBNP 水平进行统计描述和

组间比较，并对两组与基线的变化情况进行统计描述和组间比较。

12.2.4. 安全性评价：

安全性评价基于 SS 数据集进行分析。

不良事件用不良事件发生例次、例数及发生率进行描述，并对该发生率进行组间显著性检验。同时，列表详细描述各组病例出现的全部不良事件的具体表现、程度及其与药物的关系。

对实验室指标前后变化情况进行交叉表描述，按试验组和对照组分别描述治疗前正常、治疗后异常例数及该例数所占比例。对生命体征指标进行前后比较。

12.3. 期中分析

本研究计划在收集到 1/2 和 2/3 主要终点事件后进行两次期中疗效分析，以评估是否已得出有效的结论而提前终止该研究。根据 Lan-DeMets α 消耗函数和 O’Brien-Fleming 方法，第 1 次期中分析时消耗的 I 类错误α=0.0001（单侧），第 2 次期中分析消耗α=0.00605（单侧）。

期中分析有关的具体要求和操作将在 DSMB 章程中事先规定。

13. 数据管理

本研究采用 Epidata 软件或 EDC 系统进行研究数据的采集。数据管理确保临床试验数据的真实性、完整性和准确性，数据管理过程需符合《药物临床试验质量管理规范》、《临床试验数据管理工作技术指南》等法规要求，保证临床试验数据的可溯源性。以下列出数据管理的主要流程。

13.1. 数据库设计

数据管理员根据 CRF 采用 Epidata 软件或 EDC 系统设计数据库，经测试后发布。

13.2. 数据录入

CRC 负责将 CRF 中的数据录入数据库，数据录入采用二次录入方式，由两名 CRC 分别录入一遍数据，数据管理员对两个数据库进行比对，产生数据不一致清单，CRC 按照清单对照 CRF 分别修改各自的数据库，然后再进行比对，重复以上步骤，直至两个数据库完全一致。

13.3. 数据质疑管理

数据管理员依据数据核查计划（DVP）编写数据核查 SAS 程序对数据进行核查，产生数据质疑清单，经人工核对后，生成数据质疑表，由 CRA 交研究者进行答疑，答疑后的质疑表再由 CRA 返还给数据管理员，数据管理员据此修订数据库。

13.4. 医学编码

不良事件编码采用 MedDRA21.0 或者更新版本。

13.5. 数据审核

数据库清理完成后，数据管理员撰写《数据核查报告》，用于召开数据核查会议。

审核报告重点记录内容为：入组病例数、脱落、剔除病例情况、偏离或违背方案情况、依从性数据，合并用药，不良事件，与评价指标有关的数据等。

数据审核会议上，针对审核报告的内容，讨论并确定统计人群的划分。

13.6. 数据库锁定

完成数据库锁库清单，依据数据库锁定程序完成数据库锁定。数据锁定之后发现的问题，经确认后可在统计分析程序中修正。数据锁定后如有确切证据证明有必要解锁，研究者及相关人员需签署解锁文件。

数据库锁定后，由数据管理员导出 SAS 格式的数据文件，交与统计人员进行统计分析。

14. 质量控制

1) 研究者应履行各自职责，并严格遵循临床研究方案，采用标准操作规程，对所有相关观察结果和发现都应加以核实，以保证临床研究的质量控制和质量保证系统的实施。
2) 临床研究中受试者分配必须按研究设计确定的随机分配方案进行，每名受试者的处理分组编码应作为盲底由统计单位和研究者分别保存。
3) 研究者须对参加临床研究的所有人员进行必要培训，说明有关的资料、操作规范和职责，保证将数据真实、准确、完整、及时、合法的记入病历和 CRF。CRF 必须由专人负责保管。
4) 监查员应遵循标准操作规程，督促研究方案的执行情况，确认所有数据记录与报告正确完整，所有 CRF 填写正确，并与原始资料一致。

5) 稽查人员应对临床研究相关活动和文件进行系统性检查，以评价研究是否按照方案、标准操作规程以及相关法规要求进行。
6) 临床研究中各种实验室检查数据必须准确，并应记录在案或将原始报告复印件粘贴在病例报告表上。
7) 医学统计人员应把研究数据完整、无误地纳入报告，所有涉及数据管理的各种步骤均需记录在案，以便对数据质量及实施过程进行检查。
8) 临床研究资料的统计分析过程及其结果的表达必须采用规范的统计学方法。临床研究各阶段均需有医学统计人员参与。临床研究总结报告必须与统计报告相符。
9) 各方应严格按批准方案进行临床研究，任何偏离方案的情况均需记录在案。研究方案的修改需制定修改说明，并报伦理委员会批准方可执行。
10) 各研究中心设研究负责人 1 名，固定研究组成人员若干人。严格按临床研究方案要求进行。组长单位技术人员随时与各研究中心保持密切联系，并于研究早、中、后期前往各研究中心检查病例观察记录情况，及时解决可能出现的问题。

15. 伦理相关事宜

1. 在临床研究的过程中，必须对受试者的个人权益给予充分的保障，并确保研究的科学性和可靠性。受试者的权益、安全和健康高于对科学和社会利益的考虑。
2. 研究方案需经伦理委员会审议同意并签署批准意见后方可实施。在研究进行期间，研究方案的任何修改均应经伦理委员会批准；研究中发生严重不良事件，应及时向伦理委员会报告。
3. 研究者或其指定的代表必须向受试者说明有关临床研究的详细情况，经充分和详细解释研究的内容后获得知情同意书。

16. 试验进度

2018 年 7 月	完成试验方案的制定，召开筹备会
2018 年 8 月	方案修改，通过伦理审批
2018 年 8 月	试验药物、资料准备
2018 年 9 月	试验国际注册、分中心启动
2018 年 9 月	第 1 例病例筛选入组
2020 年 9 月	完成所有病例的随机入组
2021 年 9 月	各中心完成所有病例的随访工作

2022 年 2 月　　　　完成数据输入及盲态审核

2022 年 4 月　　　　统计分析工作

2022 年 6 月　　　　完成试验总结报告

17. 资料保存

研究医院应保存这些原始资料至临床试验终止后 5 年，包括对所有参加受试者的确认（能有效的核对不同的记录资料，如 CRF 和医院原始记录）、所有原始受试者知情同意书、CRF 表、药品分发的详细记录等。

18. 临床总结

统计分析结束后由试验主要研究者负责写出本研究临床总结报告并加盖主要研究单位公章。

参考文献：

1) Lopez AD. Assessing the burden of mortaIity from cardiovascuIar diS-eases[J]. WorId Health Star Q, 1993, 46(2):91-96.
2) 中华医学会心血管病学分会. 慢性心力衰竭诊断治疗指南[J]. 中华心血管病杂志, 2007, 35(12):1076-1095.
3) 陈灏珠.实用内科学[M].北京：人民卫生出版社，2001.
4) World health organization. Cardiovascular disease:prevention and control[EB/OL]. http://www.who.int/dietphysicalactivity/ publications/facts/cvd/en/. 2010-03-07.
5) Mendez GF, Cowie MR. The epidemiological features of heart failure in developing countries:a review of the literature[J]. Int J Cardiol, 2001, 80(2-3):213-219.
6) Rosamond W, Flegal K, Friday G, et al. Heart disease and stroke statistics - 2007 update - A report from the American Heart Association Statistics Committee and Stroke Statistics Subcommittee[J]. Circulation, 2007, 115(5):E69E171.
7) 顾东风，黄广勇，吴锡桂,等. 中国心力衰竭流行病学调查及其患病率[J]. 中华心血管病杂志, 2003, 31(1):3-6.
8) Roger VL, Weston SA, Redfield MA, et al. Trends in heart failure incidence and survival in a community-based population[J]. JAMA, 2004, 292(3):344-350.
9) Pulignano G, Del S D, Tavazzi L, et al. Clinical features and outcomes of elderly outpatients with heart failure followed up in hospital cardiology units: data from a large nationwide cardiology database (IN-CHF Registry).[J]. American Heart Journal, 2002, 143(1):45-55.
10) Kawashiro N, Kasanuki H, Ogawa H, et al. Clinical characteristics and outcome of hospitalized patients with congestive heart failure: results of the HIJC-HF registry[J]. Circulation Journal Official Journal of the Japanese Circulation Society, 2008, 72(12):2015.
11) Stewart S, Macintyre K, Hole D J, et al. More 'malignant' than cancer? Five-year survival following a first admission for heart failure.[J]. European Journal of Heart Failure, 2014, 3(3):315-322.
12) Cowie M R, Wood D A, Coats A J, et al. Survival of patients with a new diagnosis of heart failure: a population based study.[J]. Heart, 2000, 83(5):505-10.
13) Mcmurray J J, Packer M, Desai A S, et al. Angiotensin-neprilysin inhibition versus enalapril in heart failure.[J]. New England Journal of Medicine, 2014, 371(11):993-1004.
14) Swedberg K, Komajda M, Böhm M, et al. Ivabradine and outcomes in chronic heart failure (SHIFT): a randomised placebo-controlled study.[J]. Lancet, 2010, 376(9758):2069-2069.
15) Li X, Zhang J, Huang J, et al. A multicenter, randomized, double-blind, parallel-group, placebo-controlled study of the effects of qili qiangxin capsules in patients with chronic heart failure[J]. Journal of the American College of Cardiology, 2013, 62(12):1065-1072.
16) 吴以岭. 脉络论[M]. 中国科学技术出版社, 2010.
17) 《芪苈强心胶囊对慢性心衰大鼠心功能与肾脏水通道蛋白 2 的影响》南方医科大学附属南方医院心内科主任许顶立 2009 西安 全国心力衰竭学术会议.
18) 李佳彧，杨萍. 芪苈强心胶囊对心梗后心力衰竭大鼠 periostin 蛋白表达干预作用的研究[J]. 中国实验诊断学, 2009, 13(2):170-172.
19) 李娅，宋优，程翔,等. 芪苈强心胶囊对大鼠心肌梗死后心肌重构及心功能的影响[J]. 中国分子心脏病学杂志, 2007, 7(4):201-204.
20) 宋优，李娅，程翔,等. 芪苈强心调节急性心肌梗死大鼠心肌 TNF-α 和 IL-10 表达[J]. 中国免疫学杂志, 2007, 23(9):806-810.

1 附 1：NYHA 分级评分标准

NYHA 心功能分级评分标准：

Ⅰ级：体力活动不受限，日常活动不引起疲乏、心悸或呼吸困难。

Ⅱ级：体力活动轻度受限，休息时无症状，日常活动可引起疲乏、心悸或呼吸困难。

Ⅲ级：体力活动明显受限，休息时无症状低于日常活动量即出现症状。

Ⅳ级：不能进行任何体力活动，休息时即出现不适，任何体力活动都使症状加重。

2 附 2：超声心动图检测指标和标准操作规程

一、人员要求

参加临床试验的超声医师均须熟悉超声心动图检测标准操作规程（SOP）。

二、超声心动图检测步骤

检查内容包括二维和多普勒超声心动图。首先进行常规测量，再分别采集胸骨旁左室长轴、左室乳头肌水平短轴、心尖四腔心、心尖二腔心这五个切面的动态图像，要求每个 Loop采集 2～3个连续心动周期。房颤患者要求采集 5～10个心动周期（可连续3个心动周期×3次）。

1．体位：患者取仰卧或左侧卧位，以图像清晰为准。

2．声窗部位：胸骨旁，心尖搏动最强处或腋中线 5-6肋间。

3．图像调节：适当调节深度（ depth）、增益 (gain)、焦点位置 (focus)及前后处理。适当应用二次谐波技术、伪彩等增加图像清晰度及对比度，达到清晰显示心内膜的目的。尽量采集最好的图像（要求至少 80%的心内膜）显示清晰以便于描迹。

4．图像要求：胸骨旁左室长轴切面及 M型曲线应清晰显示室间隔、左室腔和左室后壁；心尖四腔心切面二维图像应包括心尖在内的所有四个心腔，心尖应位于扇形扫查的顶部，图像居中。四个心腔，尤其左心室腔应充分展开，左心室内膜面应清晰可辨。左心室内膜面和房室间隔十字交叉应清晰可辨。心尖二腔心切面二维图像应包括心尖在内的左右两个心腔，心尖应位于扇形扫查的顶部，图像居中。左心室腔应充分展开，左心室内膜面应清晰可辨。

三、参数的测量

1．二维常规测量基本参数左室长轴切面测量舒张末期室间隔及左室后壁厚度、左室舒张末期内径及收缩末期内径，收缩末期左房前后径、室间隔与左室后壁厚度。

2．左室收缩功能评价应用改良 Simpson’s法测量：

1）心尖四腔心：（1）左室舒张末期容积（ EDV4c）；（2）左室收缩末期容积（ ESV4c）；（3）左室射血分数（LVEF4c）；（4）左室每搏量（SV）；（5）左室每分输出量（CO）

2) 心尖二腔心：测量指标同心尖四腔切面。

3. 根据心动图 QRS波顶峰确定左室舒张末期，根据 T波终点确定左室收缩末期。根据心动图 QRS波，先勾画心尖四腔心切面的左室舒张末期及收缩末期心内膜，自动计算左室舒张末容积、左室收缩末容积、射血分数、每搏量及心输出量。再勾画心尖二腔心切面的左室舒张末期及收缩末期心内膜，自动计算上述数值。注意：左室乳头肌不应排除在勾画线之外。

同时常规连接心电图，调整导联、增益至最佳显示。同步测量受试者的心率。（如测试仪器条件许可，根据受试者身高、体重等参数记录其心脏指数（CI）

4. 多普勒检查内容记录各瓣口前向流速、返流量。如果有三尖瓣返流，因测量三尖瓣返流速度并肺动脉收缩压，测量时请准确测量返流的最高流速，以免高估肺动脉收缩压。

3 附 3：终点事件报告流程

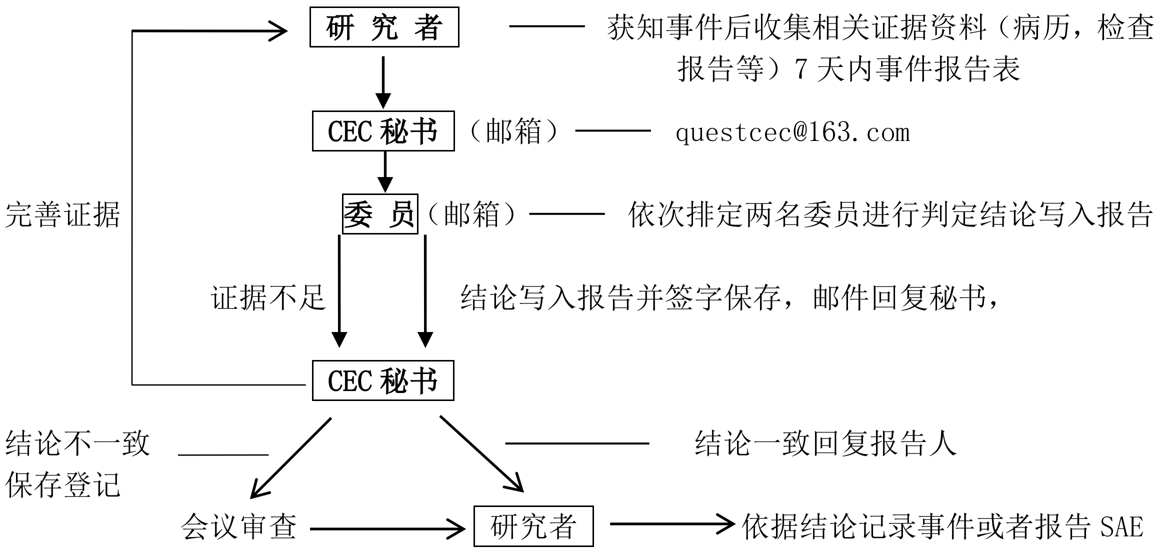

English translation version of protocol version 1.2

1. Corresponding to the amended protocol version V1.2 (in Chinese), translation date 9-Oct-2018.
2. This English version protocol was translated and submitted to the "BMC Complement Med Ther" for peer review.
3. Manuscript was published on 5 Feb, 2020, doi:10.1186/s12906-020-2821-0
4. Contents are on the basis of and translated from Chinese. If the English and Chinese contents are inconsistent, Chinese version shall prevail.

The content of this study protocol is confidential and is intended for use by clinical researchers. The material is the property of the sponsor or its sub-units and may not be copied or distributed to persons unrelated to the study.

Contents are on the basis of and translated from Chinese. If the English and Chinese contents are inconsistent, Chinese version shall prevail.

Qiliqiangxin in Heart FailUre: AssESsment of Reduction in MorTality

(QUEST STUDY)

Study Protocol

Protocol No.: SP-YFC-05-QUEST

Version No.: V1.2

Version date: Oct 9, 2018

Registration No. at ClinicalTrials.gov **********

Principal investigator: Professor LI Xinli

The First Affiliated Hospital of Nanjing Medical University

Duration of study: August 2018 to August 2021

RESPONSIBLE PARTIES

Principal Investigator of the protocol

Research unit	Principal investigator
The First Affiliated Hospital of Nanjing Medical University	LI Xinli Signature:________________ Date:________________

Statistical Analysis

Unit	Contact
Peking University Clinical Research Institute	YAO Chen

Drug Manufacturer

Unit	Contact	Tel	Email
Shijiazhuang Yiling Pharmaceutical Co., Ltd	HAN Shuolong	13582167153	hanshuolong@yiling.cn

Academic Counselling Committee

****

****

Data Safety Monitoring Committee

****

****

Clinical Event Adjudication Committee

****

****

Abbreviations

ACEI	Angiotensin converting enzyme inhibitor
ADR	Suspected adverse drug reaction
AEs	Adverse events
AHA	American Heart Association
AMI	Acute myocardial infarction
ARB	Angiotensin receptor blocker
ARNI	Angiotensin receptor neprilysin inhibitor
BNP	B-type natriuretic peptide
CEA	Clinical Event Adjudication
CGRP	Calcitonin gene-related peptide
CHF	Chronic heart failure
CRA	Clinical research auditor
CRC	Clinical research coordinator
CRF	Case report form
CV	Cardiovascular
DSMC	Data and Safety Monitoring Committee
DVP	Data verification plan
ECG	Electrocardiogram
EF	Ejection fraction
EOS	Final visit
ESC	European Society of Cardiology
ET	Endothelin
FAS	Full analysis set
HF	Heart failure
IP	Investigational Product
LVOT	Left ventricular outflow
MedDRA	ICH International Medical Dictionary
MI	Myocardial infarction
NO	Nitric oxide
NT-proBNP	N-terminal pro brain natriuretic peptide
NYHA	New York Heart Association
PPS	Per protocol set
RAAS	Renin-angiotensin-aldosterone system
SAE	Serious adverse event
SNS	Sympathetic nervous system
SAS	Safety analysis set
TVI	Time velocity integral
UNS	Unplanned visit

PROTOCOL SYNOPSIS

<table>
<tr><td>Objectives</td><td>Using evidence-based medicine research methods, with cardiovascular mortality and hospital readmission rate for worsening heart failure as the main research endpoints, further elucidating the clinical efficacy and safety of long-term use of Qiliqiangxin capsules (QLQX), clarifying the characteristics of efficacy and the suitable population, to provide high-quality clinical evidence for optimizing HF therapy.</td></tr>
<tr><td>Study design</td><td>Randomized, double-blind, placebo-controlled, multicenter clinical trial</td></tr>
<tr><td>Inclusion and Exclusion Criteria</td><td>Inclusion criteria:
1) Signed informed consent;
2) Aged ≥18 years at the time of consent;
3) Established documented diagnosis of heart failure for at least three months ago according to "Chinese Heart Failure Diagnosis and Treatment Guideline" issued by the Chinese Medical Association Cardiovascular Branch;
4) Left ventricular ejection fraction (LVEF) ≤40% (echocardiogram, radionuclide, ventriculogram, contrast angiography or cardiac MRI);
5) NYHA cardiac functional grading II to III, with stable clinical symptoms; or those diagnosed as grade IV within 2 weeks before enrollment;
6) Serum NT-proBNP ≥ 450pg/ml;
7) Those who have received standardized baseline treatment regimens without doses adjusted and given intravenously for at least two weeks prior to enrollment;
Standardized drug treatment includes angiotensin-converting enzyme inhibitor (ACEI) or angiotensin receptor blocker (ARB) or angiotensin receptor neprilysin inhibitor (ARNI), beta blocker, and aldosterone receptor antagonist (the optimal therapeutic dose should be achieved, except for contraindications or intolerance).
Exclusion criteria:
1) Patients should not enter the study if any of the following exclusion criteria are fulfilled
2) Heart failure caused by valvular disease, congenital heart disease, pericardial disease, arrhythmia or non-cardiaogenic disease, or caused by vital organ failure (such as renal, hepatic failure, etc.); and right heart failure caused by pulmonary or other definite causes; and acute heart failure;
3) Coronary revascularization (percutaneous coronary intervention [PCI] or coronary artery bypass grafting [CABG]) or cardiac synchronization therapy planned to undergo after randomization, or had received cardiac resynchronization therapy prior to enrolment;
4) Any condition outside the CV diseases such as but not limited to malignant tumor, severe mental illness, hematopoietic diseases, neuroendocrine system disease, liver transaminase and alkaline phosphatase ≥ 3 x upper limit of normal (ULN), abnormal renal function serum creatinine > 2 mg/dl (176.82 umol/L), potassium >5.5mmol/L;
5) Patient with left ventricular outflow tract obstruction, myocarditis, aortic aneurysm, aortic dissection, or obvious hemodynamic changes caused by unrepaired valve;
6) Cardiogenic shock, uncontrollable malignant arrhythmia, sinus or atrioventricular block at second degree type II or above without pacemaker treatment, progressive unstable angina pectoris or acute myocardial infarction;
7) Uncontrolled hypertension systolic blood pressure (SBP) ≥ 180mmHg and/or diastolic blood pressure (DBP) ≥ 110mmHg; or SBP < 90mmHg and/or DBP <60mmHg;</td></tr>
</table>

<table>
<tr><td></td><td colspan="2">8) Participation in another clinical study with an IP during the last month prior to enrolment;
9) Women of child-bearing potential (i.e., those who are not chemically or surgically sterilized or who are not post-menopausal) who are not willing to use a medically accepted method of contraception that is considered reliable in the judgment of the investigator, from the time of signing the informed consent throughout the study and 4 weeks thereafter, OR women who have a positive pregnancy test at enrolment or randomisation OR women who are breast-feeding;
10) Allergic constitution; known to be allergic to research drug;
11) Inability of the patient, in the opinion of the investigator, to understand and/or comply with study medications, procedures or any conditions may render the patient unable to complete the study.</td></tr>
<tr><td rowspan="2">Outcome Measures</td><td>Primary endpoints</td><td>The major adverse composite endpoint events included cardiovascular death and hospitalization for heart failure</td></tr>
<tr><td>Secondary endpoints</td><td>1. All-cause mortality
2. Secondary endpoint events (given up treatment due to worsening heart failure, successful resuscitation after cardiac arrest, malignant arrhythmia, non-fatal stroke)
3. The incidence of cardiovascular death and hospitalization for heart failure in patients with ischemic heart disease
4. Serum NT-proBNP decrease rate</td></tr>
<tr><td>Safety Outcome Measures</td><td colspan="2">To evaluate the safety and tolerability of QLQX in this patient population: changes in clinical parameters (complete blood count/ biochemistr, ECG, physical examination), and any forms of adverse events (AEs)</td></tr>
<tr><td>Sample size and Statistical methods</td><td colspan="2">The random allocation ratio between the QLQX group and control group is 1:1. The sample size is calculated based on the expected composite endpoint events of 620 composite endpoint events. Estimated incidence rate of composite endpoint events in the control group within the 36-month follow-up period is 25%. The trial lasts for approximately 36 months with an estimated recruitment period of 24 months, it is estimated that 3080 participants (1540 per group) need to be enrolled to obtain 620 endpoint events.</td></tr>
<tr><td>Dosage and mode of administration</td><td colspan="2">Study group: Standardized treatment of chronic heart failure + Qiliqiangxin capsules, 4 capsules, 3 times/day, administered orally
Control group: Standardized treatment of chronic heart failure + Placebo capsules, 4 capsules, 3 times/day, administered orally</td></tr>
<tr><td>Duration of treatment</td><td colspan="2">The patients were selected based on the criteria. The planned enrollment period was 2 years, and they took medicine for at least 1 year.</td></tr>
<tr><td>Statistical unit</td><td colspan="2">Peking University Clinical Research Institute</td></tr>
<tr><td>Expected progress</td><td colspan="2">August 2018 to August 2021</td></tr>
</table>

Contents

1. Study Title 8
2. Study Objective 8
3. Study background and rationale 8
4. Study Design 11
4.1. Randomization and Enrollment (Day 0 – 24th month) 11
4.2. Control group and Sample size 11
5. Rationale for Study Population 12
5.1. Inclusion Criteria 12
5.2. Exclusion Criteria 12
5.3. Discontinuation of investigational product (IP) 13
5.4. Withdrawal 13
5.5. Discontinuation of the study 14
6. Treatment 15
6.1 Investigational products (IP) 15
6.1.1. Products information 15
6.1.2. Package and label 15
6.1.3. Storage 16
6.1.4. Distribution and Recollection 16
6.1.5. Accountability 16
6.2. Study and Follow-up Plan 16
6.2.1. Study Procedure 16
6.2.2. Standardized heart failure treatment 17
6.2.3. Concomitant medications and other treatments 17
6.2.4. Treatment period 17
6.3. Evaluation on compliance 17
6.4 Adverse drug reaction 17
7. Outcome Measures for Analyses 18
7.1 Clinical observation endpoints 18
7.1.1. Primary outcome measure 18
7.1.2. Secondary outcome measures 18
7.2. Safety outcome measure: 18
8. Course of Study 18
9. Efficacy Assessments and Clinical Event Adjudication (CEA) Committee 20
9.1. Definitions of endpoint events 20
9.2. Endpoint reporting overview 21
10. Safety Assessment 22
10.1. Definition of Adverse Event： 22
10.2. Grading of Adverse events: 22
10.3. IP related adverse events of interest 22
10.4. Adjudication of severe adverse events 23
10.4.1. Definition of severe adverse events 23
10.4.2. Definition on specific severe adverse events 23
10.5. Recording of adverse events and follow-up 24

10.6. Adverse events based on examinations 24
11. Blinding and Unblinding 25
11.1. Random grouping of subjects 25
11.2. Unblinding and emergency unblinding protocol 25
11.3. Unblinding provisions 25
11.4. Screening number 26
12. Statistical Analysis 27
12.1. Definitions of analysis sets 27
12.2. General Statistical methods 27
12.2.1. Enrollment and completion 27
12.2.2. Compliance analysis: 27
12.2.3. Efficacy analysis: 28
12.2.4. Safety analysis: 28
12.3. Interim analysis 28
13. Data Management 30
13.1. Database Design 30
13.2. Data entry 30
13.3. Data questioning management 30
13.4. Medical coding 30
13.5. Data audit 30
13.6. Database locking 30
14. Quality Control 31
15. Ethical conduct of the study 32
16. Study timetable and end of study 32
17. Data Archiving 32
18. Clinical Summary 32
Reference: 32
1 Appendix 1: New York Heart Association (NYHA) Functional Classification 34
2 Appendix 2: NT-proBNP test, blood collection, blood sample preservation and transportation process 34
3 Appendix 3: Endpoint event report process 37

1. Study Title

Qiliqiangxin in Heart FailUre：AssESsment of Reduction in MorTality (QUEST study)

2. Study Objective

Using evidence-based medicine research methods, with cardiovascular mortality and hospital readmission rate for worsening heart failure as the main research endpoints, further elucidating the clinical efficacy and safety of long-term use of Qiliqiangxin capsules (QLQX), clarifying the characteristics of efficacy and the suitable population, to provide high-quality clinical evidence for optimizing HF therapy.

3. Study background and rationale

Cardiovascular disease is one of the major causes of death globally, seriously threatening human life and health [1, 2]. Chronic heart failure (CHF) is a series of clinical syndromes caused by pump failure, reduced ejection fraction, circulatory congestion and/or a series of neurohormonal changes on the basis of structural and/or functional abnormality [3]. As a serious stage of various heart diseases, epidemiological studies showed that the number of global heart failure patients has reached 22.5 million, and the 5-year survival rate is similar to malignant tumors.

With the changes of epidemiology and the development of social economy, the epidemiological characteristics of heart failure in developing countries are becoming similar with those in developed countries. For instance, coronary heart disease is becoming the main cause of heart failure in China [4, 5]. In recent years, the European Society of Cardiology (ESC) make a statistical study in 51 countries and has found that there are at least 15 million heart failure patients in about 1 billion people. In 2007, the American Heart Association (AHA) reported that the number of heart failure patients has exceeded 5 million in the United States, and is still increasing at a rate of 550,000/year [6]. The incidence is Japan, which has similar geographical location and ethnic group characteristics as China, is similar to that in Europe and the United States. In 2003, GU Dongfeng *et al* randomly surveyed 15,518 adults (aged 35-74 years old) in respective five provinces and cities in the south and north of China, and found that the prevalence of heart failure in China was 0.9%, including 0.7% for men and 1.0% for women [7]. The incidence and prevalence rate are also gradually increasing worldwide; the number of heart failure patients is increasing at a rate of 2 million per year.

Due to the economic improvement, diet change, and also the extending of human life, the incidence of heart failure increases with age [8]. Also, with the development of modern medicine, the mortality rate of patients with heart failure has gradually decreased, but remains in a relatively high level. The 1-year mortality rate of patients with heart failure over 70 years old is significantly higher than that of patients under 70 years old (22%: 13.7%) in the United States [9]. The 1-year and 3-year mortality rates of heart failure patients are 11.3% and 29.2% in Japan, respectively [10]. In Europe, the 4-year survival rate is only 50%, and 40% of heart failure inpatients is admitted to hospital for treatment or dies within 1 year [11, 12]. Chronic heart failure is still a major problem that seriously threatens human life and quality of life which in need to be resolved.

In the past 20 years, the concept of drug treatment for heart failure has changed greatly, from the perspective of improving hemodynamics to the point of biological adjustment. The focus of modern treatment models is to improve the neuroendocrine disorders of the renin- angiotensin-aldosterone system and the sympathetic nervous system. Therefore,

the goal of treatment is not only to improve symptoms and quality of life, but also to inhibit and delay the development of myocardial remodeling, terminate the vicious circle, thereby to reduce the mortality and hospitalization rate of heart failure.

Myocardial remodeling is the basic mechanism of heart failure, including pathological cardiomyocyte hypertrophy with re-expression of embryonic genes, cardiomyocyte apoptosis and necrosis, and excessive deposition or degradation of myocardial extracellular matrix. Slowing or preventing myocardial remodeling is of great value in preventing and controlling the occurrence and development of heart failure and improvement of cardiac function. Activation of two neuroendocrine systems, including sympathetic nervous system (SNS) and renin-angiotensin-aldosterone system (RAAS), and myocardial remodeling promote each other and aggravate the development of heart failure. ACEI, beta blockers, ARB and aldosterone receptor antagonists are shown to be beneficial, indicating the effectiveness of inhibiting these two systems. However, the mechanisms of heart failure are not limited to the SNS and RAAS, further research are needed to explore the effective anti-heart failure pathways, and infer new targets for treating heart failure.

Numbers of clinical studies in the late 1980s also confirmed that activation of neuroendocrine system leads to myocardial remodeling, which is a key factor in the occurrence and development of heart failure. In 1987, CONSENSUS, a clinical trial of angiotensin-converting enzyme inhibitor (ACEI) for heart failure, successfully reduced the total mortality of heart failure patients by 27%. Later, clinical trials such as SOLVED and V-HeFY further confirmed that ACEI can effectively improve prognosis of heart failure.

In the mid-to-late 1990s, CIBISII, MERIT-HF and COPERNICUS studies confirmed that beta blockers reduced mortality in heart failure patients by 34% to 35%. In addition, the RALES trial (1999) and EMPHASIS-HF (2011) studies had shown that aldosterone receptor antagonists can reduce mortality in heart failure patients by 24% to 30%.

New drugs that have benefited patients with heart failure since 2010 are mainly ARNI and cardiac sinus node inhibitors. In the PARADIGM-HF trial, 8442 heart failure patients with reduced ejection fraction (HFrEF) were randomized to receive ARNI and enalapril. The incidence of primary endpoints (cardiovascular death and hospitalization for heart failure) was 21.8% in ARNI group, significantly lower than the enalapril group (26.5%) [13]. The SHIFT study showed that the relative risk of hospitalization for cardiovascular death and worsening heart failure was reduced by 18% compared with the standard treatment group, and left ventricular function and quality of life were significantly improved [14] . At the same time, clinical research has also found that diuretics can effectively relieve dyspnea in patients with heart failure. Rational use of diuretics is the key and foundation for other successful treatment of heart failure by eliminates fluid retention, improves heart function and exercise tolerance.

During this period, non-pharmacological treatment of heart failure has also made important progress. Choosing the suitable patient based on drug treatment can further improve the heart function and quality of life and reduce mortality.

Despite some progress in the field of heart failure treatment in these years, the current prevalence and mortality of heart failure remain high. There is still a need to develop new treatments and develop new drugs to achieve breakthroughs in heart failure treatment.

Traditional Chinese medicine (TCM) has accumulated rich experience in the long-term medical practice of preventing and treating heart failure, and its understanding of heart failure is also deepening. There have been some researches and reports on the treatment of heart failure with TCM. The researches on the treatment of heart failure by

Chinese medicine QiliQiangxin Capsules was initially successful. The research results were published in an international journal of cardiovascular disease JACC in 2013[15] and received the attention and praise from worldwide scholars. All enrollees were treated with standard-optimized treatment, and the ratio of serum amino-terminal B-type natriuretic peptide precursor (NT-proBNP) was significantly lower in the Qiliqiangxin capsule group compared with the placebo group, higher ratio of patients with a decrease of >30% and lower incidence of adverse events were observed, indicating that Qiliqiangxin Capsules is effective and safe in the treatment of chronic heart failure. Qiliqiangxin Capsules became the first proprietary Chinese medicine to be included in the Guidelines for the Diagnosis and Treatment of Heart Failure in China 2014 and 2018.

Qiliqiangxin Capsules is the first to explore the pathogenesis and treatment of chronic heart failure with the theory of collateral disease, which proposes insufficiency of the heart-qi as the basis of TCM. Meanwhile, obstruction of collaterals is the central link. The fluid does not flow and exudes from veins which the blood stasis and water stasis blocks the veins, inducing edema [16]. The theory is similar with a new concept proposed by modern medicine in recent years that the hemodynamic changes caused by early neuroendocrine activation can lead to ventricular remodeling, which is the basic mechanism for the development of heart failure.

Due to the post-marketing unique advantages of Qiliqiangxin Capsules in the clinical application, it has become a hot spot for many experts and scholars in China. Previous studies have shown that the drug has cardiotonic and diuretic effect, can improve the heart function of CHF rats, and increase water discharge by reducing the expression of AQP2 in the kidney [17]. It also can reduce the expression of AngII and periostin proteins in rats with heart failure after myocardial infarction and is dose-dependent [18]. The mechanism of the effect of Qiliqiangxin Capsules on improving cardiac function is related to its inhibition of myocardial remodeling [19]. Reducing the pro-inflammatory factors of cardiomyocytes and increasing the immunomodulatory effects of anti-inflammatory factors may be one of the immunopharmacological mechanisms of Chinese medicine for improving heart function in AMI rats [20]. In the clinical study of taking Qiliqiangxin Capsules for the treatment of patients with chronic congestive heart failure, it is indicated that Qiliqiangxin Capsules can improve cardiac function grading, heart failure (Lee’s) score, systolic and diastolic function, ejection fraction (EF), TCM syndrome differentiation (中医证候), quality of life and other curative indicators and safety indicators, elevate blood nitric oxide (NO) and calcitonin gene-related peptide (CGRP) levels, and decrease endothelin (ET) levels; thereby significantly improving endothelial function in patients with heart failure.

In summary, Qiliqiangxin Capsules could resolve the current problems and eliminates the essential causes that treat cardiac insufficiency from multiple pathways, multiple links and multiple targets. It reflects the advantages of compound Chinese medicine in treating heart failure from the overall treatment, and significantly reduces NT- ProBNP levels, indicating improvement of the patient's long-term prognosis. This study is a large randomized, double-blind, multi-center clinical study carried out on the basis of achieving major study results with NT-proBNP as a surrogate endpoint, adopts the incidence of composite endpoint events consisting of cardiovascular death and hospitalization for heart failure as the study endpoints, in order to collect the evidence-based medical evidences for long-term prognosis in patients with chronic heart failure.

4. Study Design

This study is a randomized, double-blind, placebo-controlled, parallel-group, multicenter clinical study.

The study will be event-driven, and all randomized patients will remain in the study (whether taking the study drug or not) until the number of primary endpoint events reaches the predicted value (620 cases), or the study terminates early when it meets the pre-defined efficacy or safety criteria of early termination.

Two mid-term efficacy analyses planned to be conducted after 1/2 and 2/3 primary endpoint events to assess whether an invalid or valid conclusion was reached so as to prematurely end the study.

The entire study will last approximately 36 months, and the recruitment period will be expected to be 24 months. The follow-up period after the last case of patient is included in the study is 12 months. The average follow-up time is predicted to be about 24 months.

Patients who show stable clinical symptoms, had received at least 2 weeks of standardized treatment and treatment of other concomitant diseases before enrollment are screened at the hospital. According to the local HF treatment guidelines, the drug type and dosage are fixed, unless it is contraindicated or intolerant. The patient who have not receive anti-HF drug intravenously for at least two weeks prior to enrollment, nor take oral administration of TCM or Chinese patent medicine having similar composition with Qiliqiangxin Capsule can directly enter the random grouping stage.

If patients fail to meet the above requirements, term standardized treatment to meet the above criteria before entering the random grouping stage are needed.

4.1. Randomization and Enrollment (Day 0 – 24th month)

Noted: Patients will have to receive at least 2 weeks of standardized heart failure treatment without any other TCM and/or herbal medicine before randomization. Regimen of the heart failure treatment will be documented. Any adjustment of the medication will be recorded in the case report form (CRF).

Patients will be randomized 1:1 into study group and placebo group to receive the investigation products (IP) in addition to the standard therapy.

Study group: Standardized treatment of chronic heart failure + Qiliqiangxin capsules, 4 capsules, 3 times/day, administered orally.

Control group: Standardized treatment of chronic heart failure + Placebo capsules, 4 capsules, 3 times/day, administered orally.

Treatment with any other TCM and herbal medicine should also be avoided after randomization.

After randomization, patients will be followed up at 1st, 3rd, 6th, 9th, 12th month, and every other 3 months for efficacy and safety assessment until the end of the trial. The randomization and enrollment period will last for 24 months.

4.2. Control group and Sample size

According to the PARADIGM-HF study, the composite event of cardiovascular death and/or hospitalization for heart failure in the median follow-up of 27 months was 21.8% in the LCZ696 group and 26.5% in the Enalapril group. Therefore, we estimated that the incidence of cardiovascular death and hospitalization for heart failure is 25% in patients with standardized treatment + placebo group within 36 months of follow-up and 20% in standardized treatment +

Qiliqiangxin capsule group.

The random distribution ratio is 1:1 between study group and control group. Considering the consumption of type I error in the interim analysis, α is adjusted to unilateral 0.02314. Based on the incidence of composite endpoint events is 25%, it is expected that 620 composite endpoint events need be observed to provide 80% power of test (β= 0.2), and 20% risk can be reduced in study group by log-rank test.

The entire study will last approximately 36 months to follow up, and the recruitment period are expected to be 24 months. The sample size is expected that 3,080 patients in over 100 centers (1540 patients per group) will be enrolled and be followed up at least for 12 months.

5. Rationale for Study Population

The enrolled patients should satisfy the following inclusion criteria, and not meet any exclusion criterion. In addition to following criteria, the patient should also be excluded if there is any contraindicated medical condition or use of incompatibility drug during basic treatment period.

5.1. Inclusion Criteria

1) Signed informed consent;
2) Aged ≥18 years at the time of consent;
3) Established documented diagnosis of heart failure for at least three months ago according to "Chinese Heart Failure Diagnosis and Treatment Guideline" issued by the Chinese Medical Association Cardiovascular Branch;
4) Left ventricular ejection fraction (LVEF) ≤40% (echocardiogram, radionuclide, ventriculogram, contrast angiography or cardiac MRI);
5) NYHA cardiac functional grading II to III, with stable clinical symptoms; or those diagnosed as grade IV within 2 weeks before enrollment;
6) Serum NT-proBNP ≥450pg/ml;
7) Those who have received standardized baseline treatment regimens without doses adjusted and given intravenously for at least two weeks prior to enrollment;
8) Standardized drug treatment includes angiotensin-converting enzyme inhibitor (ACEI) or angiotensin receptor blocker (ARB) or angiotensin receptor neprilysin inhibitor (ARNI), beta blocker, and aldosterone receptor antagonist (the optimal therapeutic dose should be achieved, except for contraindications or intolerance).

5.2. Exclusion Criteria

1) Patients should not enter the study if any of the following exclusion criteria are fulfilled
2) Heart failure caused by valvular disease, congenital heart disease, pericardial disease, arrhythmia or non-cardiaogenic disease, or caused by vital organ failure (such as renal, hepatic failure, etc.); and right heart failure caused by pulmonary or other definite causes; and acute heart failure;
3) Coronary revascularization (percutaneous coronary intervention [PCI] or coronary artery bypass grafting [CABG]) or cardiac synchronization therapy planned to undergo after randomization, or had received cardiac resynchronization therapy prior to enrolment;
4) Any condition outside the CV diseases such as but not limited to malignant tumor, severe mental illness, hematopoietic diseases, neuroendocrine system disease, liver transaminase and alkaline phosphatase ≥ 3 x upper

limit of normal (ULN), abnormal renal function serum creatinine > 2 mg/dl (176.82 umol/L), potassium >5.5mmol/L;

5) Patient with left ventricular outflow tract obstruction, myocarditis, aortic aneurysm, aortic dissection, or obvious hemodynamic changes caused by unrepaired valve;

6) Cardiogenic shock, uncontrollable malignant arrhythmia, sinus or atrioventricular block at second degree type II or above without pacemaker treatment, progressive unstable angina pectoris or acute myocardial infarction;

7) Uncontrolled hypertension systolic blood pressure (SBP) ⩾ 180mmHg and/or diastolic blood pressure (DBP) ⩾ 110mmHg; or SBP < 90mmHg and/or DBP <60mmHg;

8) Participation in another clinical study with an IP during the last month prior to enrolment;

9) Women of child-bearing potential (i.e., those who are not chemically or surgically sterilized or who are not post-menopausal) who are not willing to use a medically accepted method of contraception that is considered reliable in the judgment of the investigator, from the time of signing the informed consent throughout the study and 4 weeks thereafter, OR women who have a positive pregnancy test at enrolment or randomisation OR women who are breast-feeding;

10) Allergic constitution; known to be allergic to research drug;

11) Inability of the patient, in the opinion of the investigator, to understand and/or comply with study medications, procedures or any conditions may render the patient unable to complete the study.

5.3. Discontinuation of investigational product (IP)

At any time after randomization, patients are free to discontinue for any reason. Discontinuation from IP is not the same as complete withdrawal from the study. Study drug treatment can be discontinued when:

1. The patient can stop treatment at any time;

2. The patient has allergic reactions that are clearly associated with the IP;

3. The patient has occurrence of symptoms, signs and/or abnormal examination results that are related to the IP, or the condition determined by the investigator to terminate the study;

4. Pregnancy during the study;

During the trial, the patient should take the standard dose of the IP as long as possible. The patients should resume taking the IP as soon as possible after the relevant causes are excluded and follow up as planned. Generally AEs, SAEs and potential endpoint events should not lead to IP discontinuation, unless there is a clear clinical rationale to do so.

Conversely, patients stopped taking the IP should also participate and complete in the study follow up and evaluation items. Patients that intent to discontinue will always be asked about the reason(s) and the presence of any AEs. If the patient is unable to participate in the study visit, follow-up should be continued by phone as planned to determine if any adverse events and endpoints have occurred unless the patient refuses to follow up and withdraw from the study.

5.4. Withdrawal

The patient has the right to withdraw from the study at any time for any reason. The researcher should retrieve the remaining IP when the patient withdraws. The reason for the withdrawal should be acquired by follow-up interview or telephone. Follow-up should be continued in order to ascertain whether any endpoints or safety events have occurred. Optimally, patients who discontinue from IP should continue to attend all study visits according to plan until study finish

as much as possible. Information should be recorded in the Case Report Form.

5.5. Discontinuation of the study

1) The overall study may be stopped due to the following reasons:
   - Base on Data Safety Monitoring Committee (DSMC) interim analysis results;
   - Researchers find serious safety problems;
   - Major mistakes in the study protocol;
   - The sponsors decide to suspend study due to management problems or lack of funding;
   - The competent administrative department cancels the experiment, and half-stops all studies.
2) The discontinuation of the study can be temporary or permanent. During the suspension, all study records should be kept for inspection.

6. Treatment

6.1 Investigational products (IP)

6.1.1. Products information

Study drug: Qiliqiangxin Capsule (芪苈强心胶囊)

-Ingredients: Astragalus, ginseng, monkshood, Danshen, Pepperweed Seed, rhizoma alismatis, radix polygonati officinalis, cassia twig, red flower, cortex periplocae, tangerine peel

-Property: Capsule; the contents are brown to black brown granules; bitter in taste;

-Specification: 0.3g/ granule

-Bach number: GYZZ Z20040141

-Manufacturer: Shijiazhuang Yiling Pharmaceutical Co., Ltd

Placebo: Qiliqiangxin Matching Placebo

-With identical color, specification, packaging, , property of contents and other features with Qiliqiangxin Capsule

6.1.2. Package and label

The appearance of small package is shown below and will be labeled with "For QUEST study only". Each package contains 36 capsules sealed in aluminum-plastic plates.

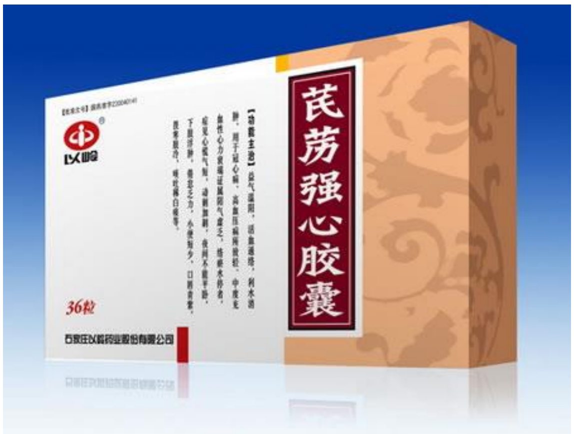

Big package: White paper box, with size of 29.5cm╳12cm╳22cm, each big package includes 33 small packages.

Each is marked with following label:

**Qiliqiangxin in Heart FailUre: AssESsment of Reduction in MorTality (QUEST)**<br>**(For Clinical Study Only)**
**Package No.: XXXXX**<br>[Lot number] xxxx [Expiry date] xxxx
[GYZZ] Z20040141<br>[Pakcage] 33 small boxes, and 36 granules in each small box (amount for 99-days)<br>[Directions] Three times a day and four granules each time<br>[Storage] Sealed in a cool dry place. Please store the product out of children's reach.
**Please be sure to follow doctor's orders and visit at the specified date to the hospital for follow-up evaluation. Thank you for your cooperation!**

6.1.3. Storage

All IP should be kept in a secure place under appropriate storage conditions. Each center must assign a committed staff to preserve and manage the study drug.

6.1.4. Distribution and Recollection

The IP provided for this study will be used only as directed in the study protocol. Patients will be asked to bring all unused study medication and empty packages to the study. During each follow-up, the investigator or delegate will collect and check the amount of returned capsule and fill in Drug Distribution Form, timely and accurately to account for all IP dispensed to and returned from the patient, in order to determine the compliance of subjects at each site visit.

At the end of study, all remaining drugs should be returned. Undistributed IPs should be sealed when returned. Remaining drugs should be retained and uniformly destroyed after study.

6.1.5. Accountability

During each follow-up, the subjects should return all remaining drugs, and researchers must make an inventory of remaining drugs and keep record

6.2. Study and Follow-up Plan

6.2.1. Study Procedure

Screening and Enrolment period (day -14 to day 0):

The investigators review the inclusion and exclusion criteria. Patients who do not meet these criteria must not be randomized into the study. During enrolment period the following assessments and procedures will be completed.

-Demography (date of birth, sex, race, ethnic group) and relevant medical and surgical history, including smoking history, will be recorded.

-General physical examination (vital signs, NYHA classification, appearance, cardiovascular systems [including edema], etc.)

-Laboratory samples will be collected and sent to the central laboratory

-ECG and echocardiogram will be recorded

Randomization and Treatment period (day 0 to 12 months [with maximum of 36 months]):

The dispensing date of the IPs is regarded as day 0. Patients will be randomized into the study or control group in a 1:1 ratio with the basis of current standardized treatments for chronic heart failure. The study drug is recommended to be taken about 30 minutes after meals, three times a day as follow.

-Study group: Standardized treatment of chronic heart failure + Qiliqiangxin Capsules (4 capsules/time, 3 times/day);

-Control group: Standardized treatment of chronic heart failure + Placebo Capsules (4 capsules/time, 3 times/day);

This study does not allow dose adjustments. If the patient miss to take the IP in a day, the accumulated dose for the next day should not exceed the daily dose. If the patient has an intolerable adverse event, which is relevant to study drug according to the judgment of researcher, the patient should terminate the following treatment with the study drug.

Do not take drugs at home on the morning of visit day. Investigators will review laboratory results received from the past visit(s). If the patient has experienced any potential endpoints, SAEs, DAEs and/or AEs of interest since the last visit,

these should be recorded in the CRF.

6.2.2. Standardized heart failure treatment

Treatment of the patients will be based on the local or regional heart failure guidelines. The treatment regimen from the 2014 China heart failure diagnosis and treatment guideline are summarized as followed:

1) Stable heart failure symptoms without the use of intravenous diuretics, inotropic, and vasodilator for 2 weeks.
2) Patients received standardized baseline treatment regimens without doses adjusted at least two weeks prior to randomization. Standardized treatment includes: angiotensin-converting enzyme inhibitor (ACEI) or angiotensin receptor blocker (ARB) or angiotensin receptor neprilysin inhibitor (ARNI), beta blocker, and aldosterone receptor antagonist (the optimal therapeutic dose should be achieved, except for contraindications or intolerance).
3) After entering the treatment period, Dose reduction or discontinuation of proven effective therapies should be avoided unless all other measures fail to improve the patient's situation. Any adjustment of the treatment regimen should be recorded in the CRF. If the patient has experienced any potential endpoints, SAEs, DAEs and/or AEs of interest since the last visit, these should be recorded in the CRF.

6.2.3. Concomitant medications and other treatments

All patients should be treated according to regional standard of care for HF and other comorbidity(s). Also, cardiac and heart failure related procedures will be captured during the study. Background medication will not be provided by the Sponsor.

1) Detailed recording of medications related to HF, HTN as well as other relevant cardiovascular medications (e.g., statins, antihypertensive and antithrombotic agents) will be made throughout the study;
2) Traditional Chinese medicines have similar contents to the test drug should not be used during the entire treatment period after entering the randomization period;
3) Patients should receive dietary guidance for heart health, such as low-salt diet, moderate drinking, etc. Patients should also receive counseling for appropriate lifestyle improvements such as weight monitoring, physical exercise, smoking cessation, and alcohol withdrawal.
4) No drug has been found to be prohibited with use of Qiliqiangxin Capsules.

6.2.4. Treatment period

12 months - 36 months

6.3. Evaluation on compliance

In order to determine the compliance of subjects, the administration (drug distribution and recovery) of all investigational products should be recorded in the appropriate sections of the eCRF.. The actual dosage should be within 80%-120% of predefined dosage.

6.4 Adverse drug reaction

There is no significant adverse drug reaction of QLQX were found.

7. Outcome Measures for Analyses

7.1 Clinical observation endpoints

7.1.1. Primary outcome measure

- The composite endpoint events consisting of cardiovascular death and/or hospitalization for heart failure;

7.1.2. Secondary outcome measures

- All-cause mortality
- Secondary endpoint events (given up treatment due to worsening heart failure, successful resuscitation after cardiac arrest, malignant arrhythmia, non-fatal stroke)
- Components of the primary endpoints in patients with ischemic heart disease
- Level of Serum NT-proBNP

Note: All endpoint events should be determined and reviewed by Clinical Event Adjudication Committee.

7.2. Safety outcome measure:

- Adverse events (Serious Adverse Events [SAEs], Discontinuation of IP due to Adverse Events, etc.)
- AEs of interest (volume depletion, renal events, etc.)
- Clinical laboratory indexes: blood routine test (hemoglobin, red blood cells, white blood cells, platelets), routine urine test (urinary protein, urinary white blood cells, urine red blood cells), serum biochemistry (urea nitrogen, creatinine, blood uric acid, total protein, albumin, alanine aminotransferase, aspartate aminotransferase, alkaline phosphatase, total bilirubin, fasting blood glucose, potassium, sodium, chlorine, total cholesterol, triglycerides, low density lipoprotein, high density lipoprotein).
- 12-lead ECG
- Physical examination

8. Course of Study

All participants, including those discontinue study drug prior to completion of study, should continuously take all planned visits listed in the table until the end of study. If a visit is postponed or taken in advance, it should not affect the next visit. The next visit should be carried out in accordance with the original planned time.

Visit	1.	2.	3.	4.	5.	6.	7.	8.	9.	10.	11.	12.	13.	14.	UNS	EOS
Day/month	Day0	1M	3M	6M	9M	12M	15M	18M	21M	24M	27M	30M	33M	36M		
	-14	±3	±7	±7	±7	±7	±7	±7	±7	±7	±7	±7	±7	±7		≥ 2 weeks
Informed consent	x															
Inclusion/ exclusion criteria	x															
Randomization	x															
General data and medical history	x															
Medical history of HF	x															
Medical history of CV Disease	x															
Physical Examination	x	x[1]	x[1]	x[1]	x[1]	x	x[1]	x[1]	x[1]	x	x[1]	x[1]	x[1]	x	(x)	x
Heart failure medications	x	x	x	x	x	x	x	x	x	x	x	x	x	x	(x)	x
Medications of other CVD	x	x	x	x	x	x	x	x	x	x	x	x	x	x	(x)	x
Other medications	x	x	x	x	x	x	x	x	x	x	x	x	x	x	(x)	x
Echocardiogram	x*															
Pregnancy tests	x					x				x				x	(x)	x
Blood/urine routine test	x	x				x				x				x	(x)	x
Biochemical test	x	x				x				x				x	(x)	x
12-lead ECG	x*	x				x				x				x	(x)	x
Serum NT-proBNP at local laboratory	x															
Serum NT-proBNP at central laboratory	x	x		x												
Dispensing IP	x		x	x	x	x	x	x	x	x	x	x	x	x		
Returning IP for accountability			x	x	x	x	x	x	x	x	x	x	x	x		x
Endpoint Event		x	x	x	x	x	x	x	x	x	x	x	x	x	(x)	x
Adverse Event		x	x	x	x	x	x	x	x	x	x	x	x	x	(x)	x
Serious Adverse Event		x	x	x	x	x	x	x	x	x	x	x	x	x	(x)	x

×[1] Simplified physical examination

- ×* Cardiac ultrasound and 12-lead ECG within the first 6 months of enrollment;
- UNS (unplanned visit): (x) The marked item is optimal and performed according to the judgment of researchers.
- EOS (final visit): Make arrangement according to the study end time (if there is a visit within one month before the end of study, it is regarded as a final visit, but needs to be supplemented with the items required completely)
- Pregnancy test is only applicable to women of childbearing age (if the urine pregnancy test is positive, it must be confirmed by serum pregnancy test).

9. Efficacy Assessments and Clinical Event Adjudication (CEA) Committee

9.1. Definitions of endpoint events

- **Hospitalization for heart failure:**

1. The patient was hospitalized for HF diagnosed preliminarily;

2. The patients who were in hospital extended at least 24 hours (or if the hospitalization time and discharge time were not available, it should indicate change of calendar date);

3. Record on the patient report that there are new symptoms or worsening symptoms due to HF, including at least one of the following:

a. Difficulty breathing (difficulty breathing on exertion, difficulty breathing on resting, orthopnea, paroxysmal breath with difficulty at night)

b. Reduced exercise tolerance

c. Fatigue

4. The patient had objective evidence of an acute exacerbation of HF, including at least two health examination results or a health examination result and at least one laboratory standard, including:

a. Determine the health examination results caused by HF, including new or deteriorated:

1) Peripheral edema; 2) Abdominal distension or increase of ascetic fluid (in the absence of primary liver disease); 3) Lung rales and/or crackles; 4) Increased jugular venous pressure and/or hepatojugular reflex (+); 5) S3 galloping; 6) Clinically significant or rapid weight gain, having relation with fluid retention;

b. Laboratory evidence of new or worsening HF obtained within 24 hours, including:

1) Increased concentration of B-type natriuretic peptide (BNP) / N-terminal B-type natriuretic peptide precursor (NT-proBNP) consistent with acute decompensated HF (eg.: BNP > 500 pg/ml or NT-proBNP > 2000 pg/ml); In patients with long-term elevation of natriuretic peptides, special attention should be paid to a significant increase beyond baseline. 2) Imaging evidence of pulmonary congestion; 3) Non-invasive diagnostic evidence of clinically significant increase in left or right ventricular filling pressure or decreased cardiac output; echocardiographic criteria includes: E/e´>15 or D leading pulmonary venous inflow pattern, congestive inferior vena cava with minimal inspiratory collapse, or reduction of small stroke distance (time velocity integral; TVI) at left ventricular outflow (LVOT). 4) Invasive diagnostic evidence: right heart catheterization showed pulmonary capillary wedge pressure (pulmonary occlusion pressure) ≧ 18mmHg, central venous pressure ≧ 12mmHg, or cardiac output index;

Note: If applicable, all results in the diagnostic check need to be reported; even if the above criteria are not met, results might provide important information for the determination of the above events.

5. The patients receive an initial or intensive treatment for HF, including at least one of the following:

a. Enhance the treatment of oral diuretics;

b. Intravenous diuretics or vasoactive drugs (such as positive inotropic drugs, vasopressors or vasodilators);

c. Mechanical or surgical intervention, including:

1) Mechanical circulation support (e.g.: Intra-aortic balloon pump, ventricular assist device, extracorporeal

membrane oxygenation, total artificial heart);

2) Mechanically assisted removal of body fluids (e.g.: ultrafiltration, hemofiltration, and dialysis);

- **Cardiovascular death:** including death caused by acute myocardial infarction (AMI), sudden cardiac death, acute decompensated heart failure, stroke, cardiovascular (CV) surgery, CV bleeding, and other CV inducing death;
- **All-cause mortality**
- **Given up treatment due to the worsening of HF:** Worsening of heart failure symptoms and signs, requiring intravenous drug or mechanical support treatment, and patients or family members voluntarily give up treatment or left hospital without cure; if the result of follow-up is death, it is included in heart failure death.
- **Successful resuscitation after cardiac arrest**
- **Malignant arrhythmia：** There is no uniform standard for the definition of malignant arrhythmia. It generally refers to arrhythmia that can cause severe hemodynamic disorder in a short period of time, causing syncope or even sudden death. According to this standard, malignant arrhythmia mainly has the following categories: (1) severe bradyarrhythmia, such as severe sick sinus syndrome, high or third degree atrioventricular block; (2) tachyarrhythmia, such as persistent ventricular tachycardia, ventricular flutter, ventricular fibrillation, atrial flutter/atrial fibrillation with rapid ventricular rates, atrioventricular reentry tachycardia, pre-excitation syndrome with atrial fibrillation, sinus tachycardia, etc.
- **Non-fatal stroke**

9.2. Endpoint reporting overview

When potential endpoint events have been identified, the researchers should collect all relevant support documents within 7 days and report to CEA committee. Investigators will record the incident in endpoint report form and submit supporting data in a timely manner (admission and discharge records, medical records, death records, ECG, etc.). The potential endpoints event (All deaths, All HF events [hospitalizations for HF or urgent HF visits], cardiac ischemic events [MI and unstable angina], cerebrovascular events [stroke and TIA], etc.) will be reviewed for central CEA process.

CEA committee consists of a chairman and 5-6 members. Each case will be independently reviewed by two members of the committee. The conclusions will be submitted to the chairman of the committee.

10. Safety Assessment

10.1. Definition of Adverse Event:

- Adverse events (AE): AE refers to any adverse medical events occurring in this clinical experiment from the moment that the patient signs the informed consent and is chosen to participate in this study to the last follow-up, whether or not the events are caused by the use of the medicine described. Major adverse events: Any adverse events and/or laboratory abnormalities, other than SAEs, that lead to targeted medical interventions (e.g., discontinuation of medication, dose adjustment, and symptomatic treatment).

10.2. Grading of Adverse events:

All clinical adverse events that occur in this clinical study will be recorded on the CRF adverse event page. The severity of adverse events will be classified. For uniform standards, the severity of events is classified as follows:

Grade 1: Mild, no clinical symptoms or mild clinical symptoms; only clinical or laboratory abnormalities; no treatment required.

Grade 2: Moderate, requiring minimal, local, or non-invasive treatment; daily life activities using age-appropriate tools [a] are restricted, such as cooking, shopping, and making phone calls.

Grade 3: Severe illness or severe medical symptoms that are temporarily not life-threatening; resulting in hospitalization or prolonged hospital stay; resulting in disability; restricted in daily living activities [b]. Activities of daily living refer to bathing, dressing, undressing, eating, using the toilet, taking medication, and being non-bedridden.

Grade 4: Life-threatening, requiring emergency treatment.

Grade 5: Death due to adverse events.

a: Daily living activities with refer to cooking, buying daily necessities or clothes, using the phone, managing finances, etc.

b: Daily living activities refer to bathing, dressing/undressing, eating, grooming, taking medication, and not being bedridden.

10.3. IP related adverse events of interest

Criteria for assessing the relationship between adverse events and investigational drugs. All causal analyses of adverse events related to investigational drugs are assessed according to five levels: definite, possible, unlikely, definite unrelated, and uncertain. The first three are considered adverse drug reactions. There are five main considerations for causal analysis:

1) Definite: There is a reasonable temporal sequence between the occurrence of the AE and the use of the investigational drug. The AE is a known adverse reaction to the investigational drug. The AE is alleviated or disappears upon discontinuation of the investigational drug, and reoccurs upon repeated use. It cannot be explained by the subject's underlying disease.
2) Possible: There is a reasonable temporal sequence between the occurrence of the AE and the use of the

investigational drug. The AE is a known or suspected adverse reaction to the investigational drug, but there may be other factors that could cause the event, such as disease or concurrent medication. The AE is alleviated or disappears upon discontinuation of the investigational drug, or the effect of discontinuation on the event is unclear, or there is a lack of decisive information.

3) Unlikely: There is a reasonable temporal sequence between the occurrence of the AE and the use of the investigational drug, but it is not a known adverse reaction to the investigational drug type, and it is highly likely to be caused by the subject's illness or other treatment.
4) Definite unrelated: There is no reasonable temporal sequence between the AE and the use of the investigational drug, such as events that occurred before the use of the investigational drug, or events that are not known adverse reactions to the investigational drug, or events that are clearly caused by other factors such as the subject's underlying disease or concurrent medication.
5) Uncertain: There is no clear relationship between the timing of the AE and the medication, and the known type of adverse reaction is similar to the investigational drug. Other co-administered drugs may cause the same reaction, and there is not enough evidence to make a clear decision.

The "definite," "possible," and "uncertain" categories are combined to calculate the incidence of adverse reactions to the investigational drug.

10.4. Adjudication of severe adverse events

10.4.1. Definition of severe adverse events

Serious adverse events (SAE) refer to any clinical events that indicate significant harm, contraindications, adverse reactions, or the need for caution. Adverse events meet the criteria for SAE when they meet one or more of the following standards:

- Death
- At life-threatening condition (the patient with this event has risk of immediate death at the time of occurring events; but do not include the aggravation of events that can lead death)
- With required hospitalization or extended hospital stay
- Sustained or significant loss of productivity or disability
- Congenital malformation

Other condition that may jeopardize the patient or may require medical intervention to prevent one of the outcomes listed above can also be classified as SAE.

10.4.2. Definition on specific severe adverse events

In this study, the following events will not be reported as serious adverse events unless they are judged negative and the researcher believes that it is related to the study drug

- ◆ Cardiovascular death
- ◆ Hospitalization for heart failure
- ◆ Given up treatment due to worsening heart failure
- ◆ Successful resuscitation after cardiac arrest

- Malignant arrhythmia
- Non-fatal stroke

Other events leading to fatal outcomes should be reported as serious adverse events.

10.5. Recording of adverse events and follow-up

If any adverse events occurred, especially those associated with the study drug, should be followed up until the patients return to baseline or tend to stabilize. If the baseline status or stability cannot be restored after follow-up, it should be noted in the CRF. All SAEs have to be reported within 24 hours, whether or not considered causally related to the investigational product, or to the study procedure(s).

10.6. Adverse events based on examinations

The results from protocol mandated laboratory tests and vital signs will be summarized and give possible interpretation. The abnormal laboratory results caused by reported adverse events should be recorded in the adverse event form. The abnormal results with clinical significance that meets one or more following conditions should be recorded as independent diagnosis on adverse event page of CRF (excluding abnormal laboratory result caused by reported adverse events):

- With associated clinical signs and symptoms
- Change in course of study drug's treatment dose
- Change in any of standard evidence based medications and (or) other treatment measures need to be changed

11. Blinding and Unblinding

11.1. Random grouping of subjects

Statistical experts at Peking University Clinical Research Institute adopts SAS 9.4 statistical software package to generate random numbers using the block randomization method according to the ratio of 1:1 between study group and control group. The study drug (Qiliqiangxin or placebo capsules) was packaged according to this random number by the person unrelated to the study.

A randomization and trail supply management system (RTSM) is used in the study, and statistical professionals will provide a random numbered list to the RTSM. The patient is then assigned a random number by the RTSM.

After completing baseline assessment, random numbers are assigned by RTSM during baseline visits. After that, the drug number is obtained through the RTSM according to the interview plan, and the serial number of drug assigned each time is different, but the drugs are the same. Before randomization of patient, the researcher must first log into the RTSM and provide the according information (e.g. the subject's date of birth, gender).

11.2. Unblinding and emergency unblinding protocol

If an adverse event occurs, emergency unblinding can only be conducted in special circumstances where it is necessary to understand the use of the investigational drug in order to treat the patient. Once the decision to unblind has been made, the researcher must record the date, time, and reason for unblinding.

The researcher needs to log in to the RTSM system to complete the unblinding application, which will be reviewed by the principal investigator before being unblinded by the unblinding officer. Once unblinded, the case will be discontinued from the study and treated as a withdrawal.

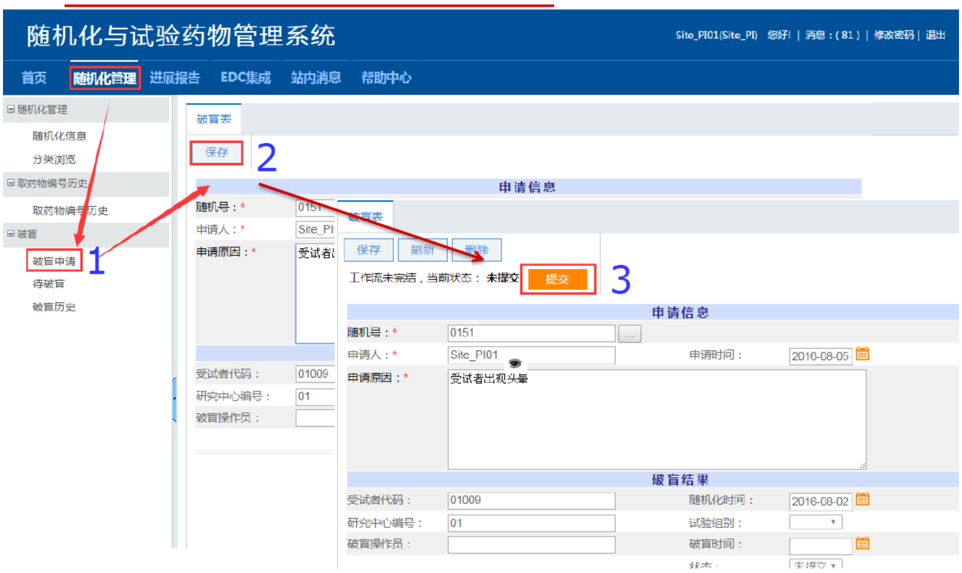

11.3. Unblinding provisions

All personnel involved with the analysis of the study will remain blinded until database lock and protocol violations have been identified and documented The study adopts two-step unblinding provision. After blind check, the data is locked, main researchers, medical statisticians, data administrators and sponsor representatives will do the first unblinding, and the random number corresponding to the group will be marked as A or B, in order to make statistical analysis on all

data. At the end of statistical analysis when summary report is completed, a secondary unblinding would be taken to reveal the group of A and B.

11.4. Screening number

The screening number was made according to the sequence of patients taking treatment at each hospital, and represented with center ID + three integers, such as the screening number is 01001, 01002 ….. for center 01.

12. Statistical Analysis

After determining study program, statistical professionals should take responsibility of formulating statistical analysis plan after consulting with main researchers. Sample size is estimated by PASS13. SAS®9.4 software (or higher version) is adopted for statistical analysis.

12.1. Definitions of analysis sets

- Full Analysis Set (FAS): refers to the data set obtained by removing the subject with the least intentional and reasonable methods from all randomized subjects as close as possible to the intention to treat. It contains all subjects who have been randomized and used study drug once. Exclusions usually included violations of important inclusion criteria, subjects not receiving treatment with the study drug, no observations after randomization. The main efficacy evaluation index was the time when the composite endpoint event occurred, and the survival analysis method was adopted for analysis. When the FAS was selected for statistical analysis, the loss of the primary endpoint event was treated according to the censorship.
- Per Protocol Set (PPS): A subset of the full analysis set, and these subjects are more compliant with the program. Subjects included in the PPS generally have the following characteristics: (1) Take the minimum exposure of study drug set in advance, that is, the compliance of taking the drug is 80%; (2) Main indicators are available in the study; (3) There was no major violation of the study protocol.
- Safety Set (SAS): All subjects who received at least one treatment after randomization and had a safety assessment. Security missing values do not need to be carried over.

12.2. General Statistical methods

Efficacy analysis is taken on the basis of FAS and PPS. All baseline demographic data analysis will be performed on the basis of FAS and safety evaluation on SAS.

- All data are performed with two-sided test, and P value less than or equal to 0.05 (two-sided test) is considered with statistical difference (unless otherwise specified).
- Descriptive analysis: Classification data is described with number of cases and percentage. Quantitative data is described with mean, standard deviation, maximum value and minimum value, median, the first quartile (Q1) and third quartile (Q3).
- Comparison of general situation should be analyzed with appropriate method based on the type of index. Quantitative data should be analyzed with paired t test or Wilcoxon rank sum test; classification data with chi-square test or precise probability method, and grade data with Wilcoxon rank-sum test or CMH test.

12.2.1. Enrollment and completion

- The number of subjects enrolled and completed at each center will be summarized, and a list of dropouts will be provided. Comparisons of group sizes, distribution of cases at each center, dropout rates, and detailed termination reasons will be presented. The demographic characteristics of patients (age, height, vital signs, etc.), medical history, and medication history will be described. The comparability of the two groups in terms of age, height, weight, etc. will also be assessed.

12.2.2. Compliance analysis:

- Medication compliance analysis: comparison between two groups of the investigational drug intake condition

(e.g. timely and correct intakes, prohibited medicine and food of the scheme, etc.)

- Drug combination analysis: analysis for the detailed list of drug combination in each group;

12.2.3. Efficacy analysis:

- PP analysis and FAS analysis were performed simultaneously with efficacy evaluation;
- Main efficacy evaluation index is the time when a composite endpoint event (cardiovascular death and hospitalization for deterioration of heart failure) occurs. The lack of primary endpoint events is consodered as censored data.

The main research hypotheses are:

H0: $\lambda T / \lambda C \geq 1$

H1: $\lambda T / \lambda C < 1$

λT and λC are the risk of endpoint events in the study group and control group respectively. The Kaplan-Meier method was used to estimate the incidence of clinical endpoints, and a Log rank test was performed for analysis between the two groups. COX proportional hazard model was used and the center was served as a covariate to estimate the hazard ratio and the 95% confidence interval. The composite endpoints, cardiovascular death and rehospitalization for deterioration of heart failure, were analyzed separately.

- Secondary efficacy indicators:
  - All-cause mortality
  - Composite endpoints (given up treatment due to worsening heart failure, successful cardiac arrest after resuscitation, malignant arrhythmia, non-fatal stroke)
  - Cardiovascular death and hospitalization for decompensated heart failure in patients with coronary heart disease
  - Serum NT-proBNP: statistical analysis of measurement data between groups.

12.2.4. Safety analysis:

Safety analysis is taken based on SS data set.

Data of adverse events (case number, times and incidence of various adverse events) are compared between the two groups. At the same time, detailed description of specific manifestation, extent of all adverse events and the relation with drugs would be further analyzed.

Crosstab is adopted to describe the change of laboratory index. Number of normal cases before treatment, number of abnormal cases after treatment and ratio of abnormal cases are analyzed in study group and control group. Indexes of vital signs are compared between before and after treatment.

12.3. Interim analysis

The study plans to perform two interim efficacy analyses after collecting 1/2 and 2/3 primary endpoints to assess whether a valid conclusion has been reached and then terminate the study early. According to Lan-DeMets α spending function and the O'Brien-Fleming method, the spending type I error was $\alpha = 0.0001$ (one side) in the first interim analysis period, and $\alpha = 0.00605$ (one side) in the second interim analysis period.

The specific requirements and operations related to the interim analysis will be specified in the DSMB in advance.

13. Data Management

This study used Epidata software to collect research data. Data management ensures the authenticity, integrality and accuracy of clinical data. The data management process needs to comply with the regulatory requirements of Clinical Trial Quality Management Regulations and Clinical Trial Data Management Work Technical Guidelines, in order to ensure traceability of study data. The main processes for data management are listed below.

13.1. Database Design

The data administrator adopts the Epidata software to design and release database according to the CRF after testing.

13.2. Data entry

Clinical research coordinator (CRC) is responsible for inputting the CRF data into the database. The data entry adopts secondary recording mode. Two CRC respectively input the data. Data administrator compares the two databases to generate the data inconsistency list. CRC modified the databases respectively according to the list and the CRF, and then made comparison again. The above steps are repeated until the two databases being identical.

13.3. Data questioning management

The data administrator wrote data verification SAS program according to the data verification plan (DVP) to verify and generated a data questioning list. The data questioning table would be generated after manual verification, and clinical research auditor (CRA) gives the data questioning table to the researcher for answer. After the researcher answering the question, CRA returned the data questioning table to data administrator and revised the database accordingly.

13.4. Medical coding

The medical coding of adverse events is done according to MedDRA 21.0 or advance version.

13.5. Data audit

After completion of database cleanup, the data administrator should write Data Verification Report for holding a data verification meeting.

The major recording contents of the audit report: number of enrolled cases, the condition of off cases and exclusion cases, the condition of deviation or violation from the program, compliance data, drug combination, adverse events, data related to the evaluation indicators, etc.

At the data audit meeting, the division of statistical population is discussed and determined according to the content of audit report.

13.6. Database locking

Complete detailed list of database locking and complete the database locking according to the program of database locking. Problems discovered after data locking can be corrected in the statistical analysis program after confirmation. After data locking, if there is clear evidence that it is necessary to unlock, the researcher and related personnel need to sign the unlocking document. After database locking, the data file is exported by the data administrator and sent to the statistician for statistical analysis.

14. Quality Control

1) Main research units and researchers should perform respective duties, strictly abide by clinical research programme, adopt standard operating procedure, verify all the related observed result, find to guarantee the quality control of clinical research, and the implementation of quality assurance system.
2) The subjects in clinical research should be distributed according to random allocation program decided by research design and the block encoding of every subject should be saved by main research units and researcher as blind codes.
3) Researcher should make necessary training, explaining related information, operation standard and responsibility to all the people participating clinical research as well as guarantee to record data into case history and CRF with sincerity, accuracy, integrity, timeliness and legitimacy. CRF must be saved by specially assigned person. Participant should own the qualification certificate of project training.
4) The supervisors should take reference to standard operation procedure, supervise and urge the execution situation of research programme, affirm correctness and integrity of all data records and reports. All CRF should be filled in correctly and keep consistency with original material.
5) The sponsors entrust auditors with systematic examination on relevant clinical activity and documents to evaluate whether the study is conducted in accordance with protocol, standard operating procedure and relevant regulatory requirement or not.
6) Various laboratory inspection data in clinical research should be recorded accurately or pasted the original report copy on CRF.
7) Medical statistician should bring research data into report timely, entirely and inerrably, all steps involved in data management should be recorded to make inspection on data quality and research implementation.
8) Statistical analysis process and the expression of results of clinical research material should adopt standard statistics method. Medical statistician should participate in every phase of clinical research. Statistical report of clinical research should conform to final report of clinical research.
9) All parties should follow with approved trial program, any situation deviating from the program should be recorded. The modification of research program should formulate modification description and be carried out after submitting to Ethics Committee for approval.
10) Each study center should consist of a study principal and several permanent researchers. The study should be conducted in strict accordance with clinical study program. The technical staff in head unit should maintain close contact with the research centers at any time, and visit each study center at early, middle and late stages of the study to inspection and timely resolve any possible problem.

15. Ethical conduct of the study

1. The rights, interests and safety of subjects should be considered prior to any science and social interest. The personal interests of subjects should be given sufficient protection and through the entire process.
2. Research program can only be implemented after deliberation agreement and signature of approval opinion by Ethics Committee. Any modification to research program should be approved by Ethics Committee during the period of research; timely report should be submitted to Ethics Committee if serious adverse event was occurred in research.
3. Researcher or the appointed representative should state the detailed research situation to all subjects. Informed consent should be acquired after sufficient and detailed explanation to research situation.

16. Study timetable and end of study

July 2018	Complete development of study programme, and hold preparatory meeting
September 2018	Modify plan and pass ethical audit
October 2018	Study drug and data preparation
January 2019	International registry of the study and start-up of sub-centers
March 2019	Select and enroll the first case
October 2020	Complete randomized grouping of all cases
October 2021	Complete follow-up of all cases in each center
December 2021	Complete data entry and blind review
April 2022	Statistical analysis
June 2022	Complete the study summary report

17. Data Archiving

All study hospitals should keep these original data at least for five years after the termination of clinical study, including confirmation of all participants (effectively verify all records, such as CRF and hospital original record), informed consent, CRF form, and detailed records of drug distribution of all subjects.

18. Clinical Summary

After the end of statistical analysis, the main researchers are responsible for composing the clinical summary report and affixing the official seal of main research unit.

Reference:

1) Lopez AD. Assessing the burden of mortality from cardiovascular diseases [J]. World Health Star Q, 1993, 46(2):91-96.
2) Cardiovascular Branch of Chinese Medical Association. Guidelines for the diagnosis and treatment of chronic heart failure [J]. Chinese Journal of Cardiovascular Diseases, 2007, 35(12):1076-1095.
3) Yuzhu CHEN. Practical Internal Medicine [M]. Beijing: People's Medical Publishing House, 2001.
4) World health organization. Cardiovascular disease: prevention and control [EB/OL].

http://www.who.int/dietphysicalactivity/ publications/facts/cvd/en/. 2010-03-07.

5) Mendez GF, Cowie MR. The epidemiological features of heart failure in developing countries:a review of the literature[J]. Int J Cardiol, 2001, 80(2-3):213-219.
6) Rosamond W, Flegal K, Friday G, et al. Heart disease and stroke statistics - 2007 update - A report from the American Heart Association Statistics Committee and Stroke Statistics Subcommittee[J]. Circulation, 2007, 115(5):E69E171.
7) Dongfeng GU, Guangyong HUANG, Xigui WU, et al. Epidemiological investigation of heart failure in China and its prevalence rate [J]. Chinese Journal of Cardiovascular Diseases, 2003, 31(1):3-6.
8) Roger VL, Weston SA, Redfield MA, et al. Trends in heart failure incidence and survival in a community-based population[J]. JAMA, 2004, 292(3):344-350.
9) Pulignano G, Del S D, Tavazzi L, et al. Clinical features and outcomes of elderly outpatients with heart failure followed up in hospital cardiology units: data from a large nationwide cardiology database (IN-CHF Registry).[J]. American Heart Journal, 2002, 143(1):45-55.
10) Kawashiro N, Kasanuki H, Ogawa H, et al. Clinical characteristics and outcome of hospitalized patients with congestive heart failure: results of the HIJC-HF registry [J]. Circulation Journal Official Journal of the Japanese Circulation Society, 2008, 72(12):2015.
11) Stewart S, Macintyre K, Hole D J, et al. More 'malignant' than cancer? Five-year survival following a first admission for heart failure.[J]. European Journal of Heart Failure, 2014, 3(3):315-322.
12) Cowie M R, Wood D A, Coats A J, et al. Survival of patients with a new diagnosis of heart failure: a population based study.[J]. Heart, 2000, 83(5):505-10.
13) Mcmurray J J, Packer M, Desai A S, et al. Angiotensin-neprilysin inhibition versus enalapril in heart failure.[J]. New England Journal of Medicine, 2014, 371(11):993-1004.
14) Swedberg K, Komajda M, Böhm M, et al. Ivabradine and outcomes in chronic heart failure (SHIFT): a randomised placebo-controlled study.[J]. Lancet, 2010, 376(9758):2069-2069.
15) Xinli LI, Jian ZHANG, Jun HUANG, et al. A multicenter, randomized, double-blind, parallel-group, placebo-controlled study of the effects of qili qiangxin capsules in patients with chronic heart failure [J]. Journal of the American College of Cardiology, 2013, 62(12):1065-1072.
16) Yiling WU. Meridian theory [M]. China Science and Technology Press, 2010.
17) Dingli XU. Effect of Qiliqiangxin Capsules on cardiac function and renal aquaporin-2 in rats with chronic heart failure. 2009 Xi'an National Heart Failure Academic Conference.
18) Jiazhen LI, Ping YANG. Study on intervention effect of Qiliqiangxin Capsules on periostin protein expression in rats with heart failure after myocardial infarction [J]. Chinese Journal of Laboratory Diagnosis, 2009, 13(2):170-172.
19) Ya LI, You SONG, Xiang CHENG, et al. Effects of Qiliqiangxin Capsules on myocardial remodeling and cardiac function after myocardial infarction in rats [J]. Chinese Journal of Molecular Cardiology, 2007, 7(4):201-204.
20) You SONG, Ya LI, Xiang CHENG, et al. Effect of Qiliqiangxin Capsules on regulation of TNF-α and IL-10 expression in myocardium of rats with acute myocardial infarction [J]. Chinese Journal of Immunology, 2007, 23(9):806-810.

1 Appendix 1: New York Heart Association (NYHA) Functional Classification

NYHA Class	Symptoms
I	No limitation of physical activity. Ordinary physical activity does not cause undue fatigue, palpitation, dyspnea (shortness of breath).
II	Slight limitation of physical activity. Comfortable at rest. Ordinary physical activity results in fatigue, palpitation, dyspnea (shortness of breath).
III	Marked limitation of physical activity. Comfortable at rest. Less than ordinary activity causes fatigue, palpitation, or dyspnea.
IV	Unable to carry on any physical activity without discomfort. Symptoms of heart failure at rest. If any physical activity is undertaken, discomfort increases.

2 Appendix 2: NT-proBNP test, blood collection, blood sample preservation and transportation process

Blood sample preparation test and standard operating procedures of cold chain transport

1. Purpose

Standardize the preservation, transportation and reception conditions and processes of blood samples

2. Scope of application

Blood sample preparation, test and transportation in a randomized, double-blind, placebo-controlled multi-center clinical trial to evaluate the efficacy and safety of Qiliqiangxin Capsules in the treatment of chronic heart failure;

3. Simple process

Blood sample preparation

Storage　Responsible party (researcher)

↓

Handover

↓

Transportation　Responsible party (express company)

↓

Receive

↓

Laboratory

4. Preparation and storage

The blood sample should be finished preparation within 2 hours after collection according to the standard operating procedures and storage of blood samples. The blood samples should be placed in the freezer tubes respectively. Attach two sheets of the adhesive Sticker to the freezer tube according to the number of the drug and the other sheet to the log sheet of reserved sample (Table 1) and fill in the log sheet of reserved sample as required.

NT-proBNP sample preparation process

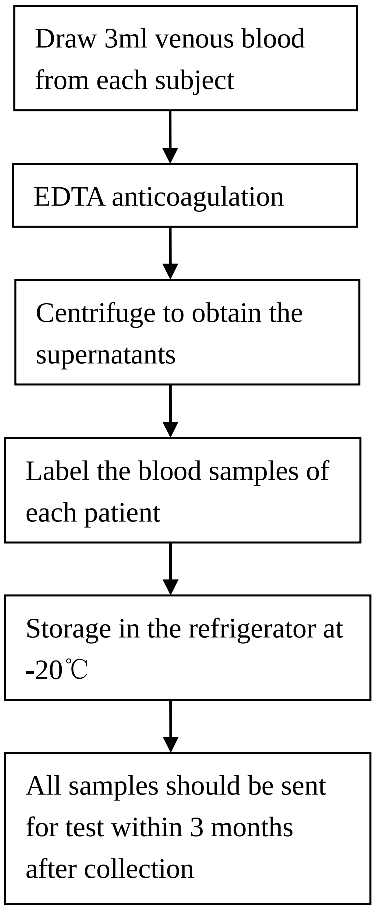

Note:

1) It should be detected in the condition of no inflammation nor infection (metabolic stability) to reduce individual differences;
2) Patient preparation: Fasting for more than 12 hours, collecting venous blood in the sitting position at 8:00 am to 9:00 am.
3) Blood collection requirements: Use a vacuum blood tube with separation gel (usually yellow cap) to collect 6 ml of venous blood; avoid the occurrence of hemolysis and lipemia (If any, please specify).
4) Serum separation: 30 minutes after the collection of specimen, the specimen should centrifuge at the speed of 3000 rpm for 5 minutes within 2 hours. Divide supernatant into two collection tube without any additives, each tube should not less than 800μl. One tube sent to the central laboratory for detection, and storage the other tube as reserved sample. Attach the same number as registration form (Table 1), including name, gender, age, specimen collection time and collection unit.

5) Transportation and preservation of serum samples: serum frozen specimens should be placed in a low temperature refrigerator at -20 °C, the maximum of retention period is 6 months. Serum samples should be placed in a dry ice box while transportation. The refrigerator temperature should be recorded daily for future reference.

Table 1 Reserved sample record table and label manuscript

Random No.	Patient name	Sex	Age	Blood collection date
Hospital (detection of NT-proBNP in Qiliqiangxin study)				

5. Transportation

After the researcher completes the tasks in the center (if the enrollment time is too long, within 2 months after the first patient was selected), dial the XXX express company's unified free order number: 400-0000-0000 by the account number: 0000000 for pick-up.

Please check and prepare the samples for the specific pickup address, contact person, contact phone number, delivery address, etc. The courier staff will arrive at the hospital, pack and hand over the samples.

The researcher fills in the blood sample specimen transfer order and attaches a copy of the sample record of the transport sample to the courier (the original is returned to the researcher by the auditor for the next inspection). The numbers of specimens, sample records, and transfer orders should match. The courier company should deliver the sample to the central lab within 24 hours.

6. Receive

The central laboratory personnel should check the blood samples, input mark code and storage the blood samples on the day the samples received. At the same time, the status of the sample should be recorded immediately.

7. Central laboratory

The central laboratory should complete the detection within 10 working days after receiving the sample; and at the same time, feedback the results to the leader unit and the monitor. The monitor should feedback the results to the corresponding research center within one week of receiving the test results.

Contact man: HAN Shuolong, Medical Department of Yiling Pharmaceutical

Tel: 13582167153

Email: hanshuolong@126.com

8. Conversion between different units

1 pg/ml = 0.118 pmol/L

pg (picogram), pmol (picomolar)

3 Appendix 3: Endpoint event report process

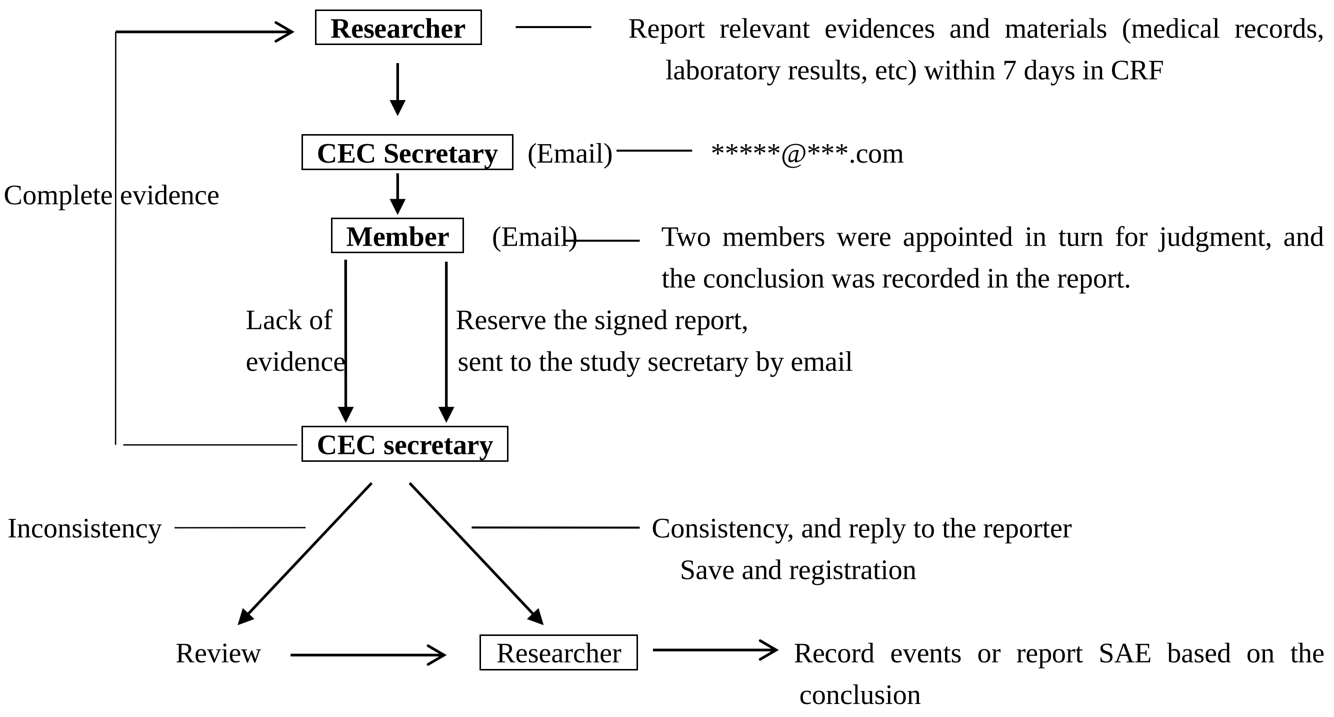

研究方案修订 V1.2，2018 年 9 月 10 日

1. 本次修订为针对方案讨论会中各专家提出的修订意见，在注册审查及研究启动前，对研究方案的内容进行订正，主要修改内容如下：
2. 补充对“7.2. 安全性指标”中观察的临床实验室指标进行详细描述，具体展开为“血常规（血红蛋白、红细胞、白细胞、血小板）、尿常规（尿蛋白、尿白细胞、尿红细胞）、血清生化（尿素氮、肌酐、血尿酸、总蛋白、白蛋白、谷丙转氨酶、谷草转氨酶、碱性磷酸酶、总胆红素、空腹血糖、钾、钠、氯、总胆固醇、甘油三酯、低密度脂蛋白、高密度脂蛋白）”；
3. 删除与“7.1.1-7.1.2”重复描述的“9.1.1-9.1.2”内容。

本研究方案所包含的内容为保密内容，是为临床研究人员使用。本资料为资助者或其子单位的财产，不得复印或分发给与本研究无关的人员。

芪苈强心胶囊对慢性心衰复合终点事件的评估研究

Qiliqiangxin in Heart FailUre：AssESsment of Reduction in

MorTality and MorbidiTy

（**QUEST** 研究）

研究方案

Study Protocol

试验方案编号：SP-YFC-05-QUEST

试验方案版本号：V1.2

试验方案版本日期： 2018年9月10日

ClinicalTrials.gov注册号：**********

主要研究者：李新立 教授

南京医科大学第一附属医院

试验计划开始结束日期： 2018年8月--2021年8月

主要研究单位及负责人

主要研究单位	主要研究者
南京医科大学第一附属医院	李新立　签名：____________<br>日期：____________

数据统计单位及负责人

单位	负责人
北京大学临床研究所	姚晨

药品生产企业及联系方式

单位	联系人	电话	邮箱
石家庄以岭药业股份有限公司	韩硕龙	13582167153	hanshuolong@yiling.cn

学术顾问

****

****

数据和安全监测委员会

****

****

事件判定委员会

****

****

缩略语

ACEI	血管紧张素转换酶抑制剂
ADR	可疑药物不良反应
AEs	不良事件
AHA	美国心脏学会
AMI	急性心肌梗死
ARB	血管紧张素受体拮抗剂
ARNI	血管紧张素受体脑啡肽酶抑制剂
BNP	B 型利钠肽
CEC	事件判定委员会
CGRP	降钙素基因相关肽
CHF	慢性心力衰竭
CRA	临床研究监查员
CRC	临床研究协调员
CRF	病历报告表
CV	心血管
DSMB	数据与安全监察委员会
DVP	数据核查计划
ECG	心电图
EF	射血分数
EOS	终末访视
ESC	欧洲心脏病学会
ET	内皮素
FAS	全分析集
HF	心力衰竭
LVOT	左心室流出量
MedDRA	ICH 国际医学用语词典
MI	心肌梗死
NO	一氧化氮
NT-proBNP	氨基末端 B 型利钠肽前体
NYHA	纽约心脏病学会
PPS	符合方案集
RAAS	肾素-血管紧张素-醛固酮系统
SAE	严重不良事件
SNS	交感神经系统
SS	安全性分析集
TVI	时间流速积分
UNS	计划外访视

研究方案摘要

<table>
<tr><td>试验目的</td><td>采用循证医学研究方法，以心血管死亡率和心衰恶化再住院发生率为主要研究终点，进一步阐明芪苈强心胶囊长期用药的临床疗效及安全性，明确疗效特点及适宜人群，为优化临床合理用药方案提供高质量临床证据</td></tr>
<tr><td>试验设计</td><td>随机、双盲、安慰剂平行对照多中心临床试验</td></tr>
<tr><td>入选和
排除标准</td><td>入选标准：
1） 自愿参加，理解并签署知情同意书；
2） 年龄≥18 岁，性别不限；
3） 有 3 个月以上的慢性心衰病史或临床发现心衰症状 3 个月以上；慢性心衰诊断参照当地指南或中华医学会心血管病学分会发布的“中国心力衰竭诊断和治疗指南 2014”；
4） 心脏彩超检查提示左室射血分数（LVEF）≤40％（改良辛普森法）；
5） NYHA 心功能分级Ⅱ～Ⅲ，临床症状稳定，包括入选前 2 周内曾诊断为Ⅳ级者；
6） 血清 NT-proBNP 含量≥ 450pg/ml；
7） 至少已接受 2 周以上的标准化药物治疗，且未调整过给药剂量及给予静脉治疗者；标准化药物治疗包括：血管紧张素转换酶抑制剂（ACEI）或血管紧张素受体拮抗剂（ARB）或血管紧张素受体脑啡肽酶抑制剂(ARNI)、β受体阻滞剂及醛固酮受体拮抗剂（除非禁忌或不耐受，应达到最佳治疗剂量）；
排除标准：
1） 不符合入选标准；
2） 由于瓣膜病、先天性心脏病、心包疾病、心律失常所致及非心源性病因所致心衰，或肾、肝等重要脏器功能衰竭导致的心衰，及有明确肺源性或其他原因所致的右心衰、及急性心衰；
3） 计划于近期内行冠脉血运重建治疗或心脏再同步化治疗者，已实施心脏再同步化治疗者；
4） 合并肝、肾、造血系统等严重原发性疾病，肾功能异常者，肝脏转氨酶、碱性磷酸 酶超出 3 倍正常值上限，血肌酐＞2mg/dl(176.82umol/L)，血钾＞5.5mmol/L；肿瘤患者，严重神经内分泌系统疾病及精神病患者；
5） 存在左室流出道梗阻、心肌炎、大动脉瘤、夹层动脉瘤、致明显血液动力学</td></tr>
</table>

	改变的未修补的心脏瓣膜病患者；<br>6） 存在心源性休克、难以控制的恶性心律失常、二度Ⅱ型以上未置入起搏器治疗的窦房或房室传导阻滞、进行性加重的不稳定心绞痛或急性心肌梗死者；<br>7） 未获控制的高血压患者，收缩压≥180/mmHg 和/或舒张压≥110mmHg；收缩压<90mmHg 和/或舒张压<60mmHg；<br>8） 1 个月内参加其他药物临床研究者；<br>9） 妊娠或正准备妊娠及哺乳期妇女；<br>10）过敏体质者，或已知对治疗药物过敏者；<br>11）根据研究者判断，患者不能完成本研究或不能遵守本研究的要求（由于管理方面的原因或其它原因）。	
**疗效性指标**	主要指标	心血管死亡和心衰恶化再住院组成的复合终点事件发生率
	次要指标	1、 全因死亡率<br>2、 复合终点事件（心衰恶化放弃治疗、心脏骤停后复苏成功、恶性心律失常、非致死性卒中）<br>3、 冠心病心衰患者的心血管死亡和心衰恶化再住院发生率<br>4、血清NT-proBNP下降率
**安全性指标**	血常规、尿常规，心电图，生化全项，不良事件，体格检查	
**样本量**	试验组与对照组的随机分配比例为 1:1。样本量为复合终点事件的发生例数。预计需要观察到 620 例复合终点事件。<br>假设随访期 36 个月内对照组复合终点事件的发生率为 25%，整个试验持续大约 36 个月，招募期预计 24 个月，则预计需要入组 3080 例（每组 1540 例）受试者才可获得 620 个终点事件。	
**给药方案**	试验组：慢性心衰标准化治疗+芪苈强心胶囊 4 粒/次，3 次/日，口服<br>对照组：慢性心衰标准化治疗+芪苈强心胶囊安慰剂 4 粒/次，3 次/日，口服	
**疗程**	以观察事件为指标入选病人，计划入组时间 2 年，至少服药 1 年。	
**试验统计**	北京大学临床研究所	
**预期进度**	2018 年 8 月-2021 年 8 月	

目录

1. 试验题目 8
2. 试验目的 8
3. 试验背景与原理 8
4. 试验总体设计与安排 11
4.1. 随机分组阶段（第 0 天~第 24 个月）： 11
4.2. 病例数量、分组、中心 11
5. 研究人群 12
5.1. 入选标准 12
5.2. 排除标准 13
5.3. 中止研究药物治疗 13
5.4. 退出标准 14
5.5. 全面中止试验标准 14
6. 治疗 14
6.1. 试验用药物 14
6.1.1. 药物来源 14
6.1.2. 制剂、包装与标签 15
6.1.3. 保存方法 16
6.1.4. 药物的发放与回收 16
6.1.5. 药物清点 16
6.2. 治疗方案 16
6.2.1. 研究流程： 16
6.2.2. 基础治疗 16
6.2.3. 合并用药 17
6.2.4. 疗程：12 个月-36 个月。 17
6.3. 依从性评价 17
6.4. 药物不良反应 17
7. 临床观察终点和指标 17
7.1. 临床观察指标 17
7.1.1. 主要有效性终点 17
7.1.2. 次要有效性终点 17
7.2. 安全性指标包括： 18
8. 试验过程 18
9. 终点事件的含义及 CEC 判定规程 19
9.1. 本研究终点事件的含义 19
9.2. 终点事件发生时的评估与程序 20
10. 不良事件的观察 21
10.1. 定义： 21
10.2. 不良事件强度判定标准： 21
10.3. 不良事件与研究药物关系的判断标准 21
10.4. 严重不良事件的判定 22
10.4.1. 一般严重不良事件定义 22
10.4.2. 严重不良事件的研究特异性定义 22
10.5. 不良事件的随访与记录 23

10.6. 实验室结果异常 23
11. 盲法与随机化 23
11.1. 受试者随机分配方法 23
11.2. 紧急揭盲 23
11.3. 揭盲规定 24
11.4. 筛选编号 24
12. 统计分析 24
12.1. 分析人群 24
12.2. 统计分析方法 25
12.2.1. 入组及完成情况： 25
12.2.2. 依从性分析： 25
12.2.3. 疗效评价： 26
12.2.4. 安全性评价： 26
12.3. 期中分析 27
13. 数据管理 27
13.1. 数据库设计 27
13.2. 数据录入 27
13.3. 数据质疑管理 27
13.4. 医学编码 27
13.5. 数据审核 27
13.6. 数据库锁定 28
14. 质量控制 28
15. 伦理相关事宜 29
16. 试验进度 29
17. 资料保存 29
18. 临床总结 29
参考文献： 30
1 附 1：NYHA 分级评分标准 31
2 附 2：NT-proBNP 检查采血及血样保存及运输流程 31
3 附 2：终点事件报告流程 34

1. 试验题目

芪苈强心胶囊对慢性心衰复合终点事件的评估研究

2. 试验目的

采用循证医学研究方法，以心血管死亡率和心衰恶化再住院发生率为主要研究终点，进一步阐明芪苈强心胶囊长期用药的临床疗效及安全性，明确疗效特点及适宜人群，为优化临床合理用药方案提供高质量临床证据。

3. 试验背景与原理

在全球范围的死亡原因中心血管疾病位列前三，严重地危害着人类的生命和健康[1,2]。慢性心力衰竭（CHF）是在器质性心脏病基础上发生泵功能衰竭、射血分数减少、循环淤血，同时出现一系列神经体液改变的一系列临床综合症[3]。心衰作为各种心脏病发展的严重阶段，正在成为本世纪最重要的心血管病症。有流行病学资料显示，目前全球心衰患者的数量已高达 2250 万，且 5 年存活率与恶性肿瘤相仿。随着流行病学的变迁和社会经济的发展，发展中国家心衰的流行病学特点与发达国家日益相近，如冠心病作为心衰的病因在我国显得越来越突出[4,5]。欧洲心脏病学会（ESC）近年来通过对 51 个国家的统计发现，在约 10 亿的人群中，至少有 1500 万例心衰患者。2007 年美国心脏学会（AHA）报道，美国的心衰患者人数已经超过 500 万，并且仍以 55 万/年的速度不断增加[6]。与我国地理位置相近、种族人群特征相似的日本发病情况与欧美国家类似。2003 年，顾东风等在我国南方和北方各 5 个省市，随机抽样调查了 15518 名成年人（年龄 35～74 岁），分析发现我国心衰患病率为 0.9%，其中男性为 0.7%，女性为 1.0%[7]。近年来，我国与发达国家的心衰患者数量都在不断增加，全球患者以每年 200 万的速度递增，发病率和患病率也在逐步上升，其重要原因之一就是社会人口老龄化，随着年龄的增长心衰发病率升高[8]。而且随着治疗水平的发展，心衰患者死亡率虽较过去有所下降，但仍然处于较高水平。美国 70 岁以上的心衰患者 1 年死亡率明显较 70 岁以下患者高（22％：13.7％）[9]。日本的心衰患者 1 年和 3 年死亡率分别为 11.3％和 29.2％[10]，在欧洲，4 年生存率仅为 50％，而且有 40％因心衰入院的患者将可能在 1 年内再次入院治疗或者死亡[11,12]。慢性心衰依然是严重威胁人类生命和生活质量的主要问题，所以国际上仍将慢性心衰作为本世纪需要解决的重要课题。

近 20 年来，人们对心衰的药物治疗理念发生了极大转变，从改善血流动力学观点进展到生物学调整的观点。现代治疗模式的重点是改善肾素-血管紧张素-醛固酮系统及

交感神经系统的神经内分泌紊乱。因此，治疗的目标不仅仅是改善症状和提高生活质量，更应注重抑制和延缓心肌重构的发展，阻断恶性循环，从而降低心衰的死亡率和住院率。

心肌重构是心衰发生发展的基本机制，包括病理性心肌细胞肥大伴胚胎基因再表达、心肌细胞凋亡与坏死及心肌细胞外基质过度沉积或降解增加等。改善心肌重构对预防、控制心衰的发生、发展和改善心功能具有重要的价值。神经内分泌两个系统包括交感神经系统(SNS）、肾素-血管紧张素-醛固酮系统（RAAS）的激活和心肌重构相互促进、加重心衰的发展。ACEI、β 受体阻滞剂、ARB 和醛固酮受体拮抗剂的应用获得了有益效果，从而说明阻断这两个系统是有效的，进一步证实了对心衰的发生和发展的这一基本机制的认识是完全正确的。但心衰的发生发展机制仍有待于更深入的研究，从而发掘更多，更有效的抗心衰途径，在机制研究中推断治疗心衰的新靶点。

20 世纪 80 年代后期大量临床研究的开展，也证实了神经内分泌系统激活导致心肌重构是引起心衰发生和发展的关键因素。1987 年，应用血管紧张素转换酶抑制剂(ACEI)治疗心衰的临床试验 CONSENSUS，成功降低心衰患者总死亡率达 27%，以后 SOLVED、V-HeFY 等临床试验进一步证实 ACEI 能够有效改善心衰患预后。

20 世纪 90 年代中、后期的 CIBISII、MERIT-HF、COPERNICUS 研究证实β受体阻滞剂使心衰患者死亡率降低 34%～35%。此外，RALES 试验(1999 年)、EMPHASIS-HF(2011 年)研究显示醛固酮受体拮抗剂可使心衰患者死亡率降低 24%～30%。

2010 年以来能够给心衰患者带来获益的新药主要是 ARNI 和心脏窦房结抑制剂。PARADIGM-HF 试验中，8442 例射血分数降低的心衰(HFrEF)患者随机接受 ARNI 和依那普利，ARNI 组主要终点事件(心血管死亡和因心衰住院)发生率为 21.8%，显著低于依那普利组(26.5%)[13]。SHIFT 研究显示，与标准治疗组比较，伊伐布雷定组使心血管死亡和心衰恶化住院的相对风险降低 18%，患者左心室功能和生活质量均显著改善。[14]同时，临床研究也发现利尿剂可有效缓解心衰患者的呼吸困难、消除液体潴留，改善心功能和运动耐量，合理恰当使用利尿剂是其他治疗心衰药物取得成功的关键和基础。

在此期间，心衰的非药物治疗也取得重要进展，在药物治疗基础上选择合适的患者，CRT 能进一步改善其心功能和生活质量，降低死亡率。

尽管在这些年心衰治疗领域取得了一些进展，但目前心衰的患病率和病死率依然居高不下。尚需要开拓新的治疗方法和研发新的药物以期在心衰治疗中取得突破。

中医学在防治心力衰竭的长期医疗实践中，积累了丰富的经验，对心衰的认识也在

不断深化。

中药治疗心衰已有一些研究和报道，中药芪苈强心胶囊治疗心衰研究初步获得成功，该研究结果于 2013 年发表于国际心血管病 JACC 杂志上[15]，并受到国外学者的关注和好评。所有入选者均采用了标准优化治疗，加用芪苈强心胶囊组较安慰剂组，血清氨基末端 B 型利钠肽前体（NT-proBNP）水平显著降低，且降幅＞30%的患者比率也显著增加，而不良反应发生率显著降低，表明芪苈强心胶囊治疗慢性心衰有效、安全，标志着中药成为心衰公认治疗药物向前迈进一大步。芪苈强心胶囊成为第一个列入《中国心力衰竭诊断和治疗指南 2014》的中成药。

芪苈强心胶囊是首次运用络病理论探讨慢性心衰的病机和治疗，提出心气虚乏是其发生的中医病机之本，络脉瘀阻是其中心环节，津液不循脉络运行渗出脉外而为水湿之邪发为水肿，瘀血水饮阻滞脉络，日久结聚成形导致心络“络息成积”是其发展加重的结果[16]，这与西医学近年提出的早期神经内分泌激活引起的血流动力学改变，进而导致心室重构是心衰发生发展的基本机制的新概念相吻合。

由于芪苈强心胶囊上市后在临床应用中显示出的独特优势，成为国内多名专家学者研究的热点。已有的研究表明该药有强心、利尿作用，可以改善 CHF 大鼠心脏功能，并且通过减少肾脏 AQP2 的表达，增加水的排出[17]。能够减少心梗后心衰大鼠的 Ang II、periostin 蛋白的表达，并具有剂量依赖性[18]。芪苈强心胶囊有效改善心功能的作用机制与其抑制心肌重构有关[19]。减少心肌细胞促炎因子和增加抗炎因子的免疫调节作用可能是中药芪苈强心改善 AMI 大鼠心功能的免疫药理机制之一[20]。在观察芪苈强心胶囊治疗对慢性充血性心力衰竭病人疗效的临床研究中，表明芪苈强心胶囊可以改善心衰患者心功能分级、Lee 氏心力衰竭计分、心脏收缩和舒张功能、射血分数(EF)、中医证候和生活质量等疗效指标及安全性指标，升高血一氧化氮(NO)、降钙素基因相关肽（CGRP）水平，降低其内皮素(ET)水平，从而明显改善心力衰竭患者的内皮功能。

综上所述，芪苈强心胶囊全方标本兼治，从多途径、多环节、多靶点治疗心功能不全，体现了复方中药在治疗心力衰竭方面从整体论治的优势，明显降低 NT-proBNP 水平，这意味着患者的长期预后可能有所改善。本研究是在芪苈强心胶囊以 NT-proBNP 为替代终点并取得重大研究成果的基础上，继续深入开展的以心血管死亡和心衰再住院组成的复合终点事件发生率为研究终点，以获得对慢性心衰患者长期预后循证医学研究证据的大型随机、双盲、多中心临床研究。

4. 试验总体设计与安排

本研究是一项在慢性心衰患者中进行的随机、双盲、安慰剂对照、平行分组的多中心临床研究。

本研究将为事件驱动型，全部随机入组的患者将保留在研究之中（无论是否服用研究药物），直至主要终点事件的发生数目达到预计（620 例），或者当满足事先定义的提前终止的疗效或安全性标准时，研究提前终止。

计划在发生 1/2、2/3 主要终点事件后进行两次期中疗效分析，以评估是否已得出无效或有效的结论而提前终止该研究。

计划整个研究将持续大约 36 个月，招募期预计 24 个月，最后一例患者入组研究后的随访期为 12 个月。预计平均随访时间约为 24 个月。

在医院开始筛选患者，临床症状稳定，入选之前已接受至少 2 周标准化方案治疗并治疗其他伴随疾病。根据当地 HF 治疗指南规定用药，药物种类、剂量固定，除非禁忌或不耐受，且此期间未静脉用药，未服用与芪苈强心胶囊成分相似中药、中成药的患者直接进入随机分组阶段。

若达不到上述要求，则可先行标准化治疗达到上述标准后再进入随机分组阶段。

4.1. 随机分组阶段（第 0 天~第 24 个月）：

*请注意：*接受 2 周以上的标准化治疗方案且未使用中药治疗符合入选标准的受试者进入随机分组阶段。此期间的每一位患者使用药物种类、剂量需要固定。若属医疗需要调整用药，需记录在病例报告表中。

患者将按照 1:1 的比例随机化到试验组或对照组。患者将在当前慢性心衰标准化治疗的基础上使用研究药物。

试验组：慢性心衰标准化治疗+芪苈强心胶囊（4 粒/次，3 次/日，口服）；

对照组：慢性心衰标准化治疗+芪苈强心胶囊安慰剂（4 粒/次，3 次/日，口服）。

治疗期间应避免使用其他中药或中成药（与芪苈强心功能组成相似的中药）

患者应于随机分组后第 1 个月、第 3 个月、第 6 个月，第 9 个月，第 12 个月，此后每隔 3 个月来医院访视，进行有效性和安全性评估，直到研究全部结束。随机分组阶段共 24 个月。

4.2. 病例数量、分组、中心

参考 PARADIGM-HF 研究，中位随访 27 个月 LCZ696 组患者的心血管死亡或心衰住院

率为 21.8%，而依那普利组为 26.5%。所以我们估算基础治疗+安慰剂组随访 36 个月内，所有患者的心血管死亡和心衰住院事件发生率为 25%，基础治疗+芪苈强心组发生率为 20%。

试验组与对照组的随机分配比例为 1:1，考虑到期中分析对 I 类错误的消耗，α 调整为单侧 0.02314。样本量为复合终点事件的发生例数。预计需要观察到 620 例复合终点事件，才能提供 80%的把握度（β=0.2），经过 log-rank 检验得到试验组可以降低 20%风险的结论。

假设随访期 36 个月内对照组复合终点事件的发生率为 25%，整个试验持续大约 36 个月，招募期预计 24 个月，则预计需要入组 3080 例（每组 1540 例）受试者才可获得 620 个终点事件。

因此本研究计划纳入 3080 例患者，患者将以患者将以 1:1 的比例分配至试验组与对照组，并计划在约 100 个中心进行。

5.　研究人群

入组患者必须满足下文所列的所有入选标准，并且不符合任何一项排除标准。除下文所列标准外，接受标准化治疗期间，如果存在任何禁忌的医学状况或使用禁忌药物，也是排除患者入选的标准。

5.1. 入选标准

1)　自愿参加，理解并签署知情同意书；
2)　年龄≥18 岁，性别不限；
3)　有 3 个月以上的慢性心衰病史或临床发现心衰症状 3 个月以上；慢性心衰诊断参照当地指南或中华医学会心血管病学分会发布的“中国心力衰竭诊断和治疗指南 2014”；
4)　心脏彩超检查提示左室射血分数（LVEF）≤40％（改良辛普森法）；
5)　NYHA 心功能分级Ⅱ～Ⅲ，临床症状稳定，包括入选前 2 周内曾诊断为Ⅳ级者；
6)　血清 NT-proBNP 含量≥ 450pg/ml；
7)　至少已接受 2 周以上的标准化药物治疗，且未调整过给药剂量及给予静脉治疗者；标准化药物治疗包括：血管紧张素转换酶抑制剂（ACEI）或血管紧张素受体拮抗剂（ARB）或血管紧张素受体脑啡肽酶抑制剂(ARNI)、β受体阻滞剂及醛固酮受体拮抗剂（除非禁忌或不耐受，应达到最佳治疗剂量）

5.2. 排除标准

1) 不符合入选标准；
2) 由于瓣膜病、先天性心脏病、心包疾病、心律失常及非心源性病因所致心衰，或肝、肾等重要脏器功能衰竭导致的心衰；及有明确肺源性或其他原因所致的右心衰、及急性心衰；
3) 计划于近期内行冠脉血运重建治疗者或心脏再同步化治疗者，已实施心脏再同步化治疗者；
4) 合并肝、肾、造血系统等严重原发性疾病，肾功能异常者，肝脏转氨酶、碱性磷酸酶超出 3 倍正常值上限，血肌酐＞2mg/dl(176.82umol/L)，血钾＞5.5mmol/L；肿瘤患者，严重神经内分泌系统疾病及精神病患者；
5) 存在左室流出道梗阻、心肌炎、大动脉瘤、夹层动脉瘤、致明显血液动力学改变的未修补的心脏瓣膜病患者；
6) 存在心源性休克、难以控制的恶性心律失常、二度Ⅱ型以上未置入起搏器治疗的窦房或房室传导阻滞、进行性加重的不稳定心绞痛或急性心肌梗死者；
7) 未获控制的高血压患者，收缩压≥180/mmHg 和/或舒张压≥110mmHg；收缩压<90mmHg 和/或舒张压<60mmHg；
8) 1 个月内参加其他药物临床研究者；
9) 妊娠或正准备妊娠及哺乳期妇女；
10) 过敏体质者，或已知对治疗药物过敏者；
11) 根据研究者判断，患者不能完成本研究或不能遵守本研究的要求（由于管理方面的原因或其它原因）。

5.3. 中止研究药物治疗

随机分组后，任何原因暂停研究药物不等于永久停用，也不应该导致患者退出整个研究。相反对于已经停止服用研究药物的患者，也应该参加所有方案规定的研究访视和评价项目。如果患者不能参加研究访视，应按照计划通过电话继续随访，以确定是否发生任何不良事件和终点事件，除非患者拒绝随访并撤回知情同意书。

出现以下情况时可中止研究药物治疗：

1. 患者可在任何时间中止治疗
2. 发生与研究药物明确相关的过敏反应

3. 发生与研究药物明确相关的不良症状或体征、异常检查结果，研究者判断须终止研究的情况
4. 女性于研究期间发生妊娠

试验过程中应尽可能使患者长期服用标准剂量研究药物，中止研究药物患者在排除相关原因后应尽早恢复服用研究药物并按计划进行随访。

5.4. 退出标准

所有填写了知情同意书并筛选合格进入试验的受试者，无论何时何因退出，均不会影响其后续治疗。

患者有权在任何时间以任何理由退出研究，但应当尽量避免不必要的患者退出，并积极采取措施，尽可能完成随访，以备对其疗效和安全性进行分析。但当患者决定退出时，研究者应当通过电话或个人访问形式联系患者或其责任亲属并尽可能确认退出原因，研究者应当在患者退出时回收剩余药物，完成最终评估，尽可能完成病例报告、解释退出原因，对退出患者发生终点事件进行随访。如果患者退出的原因为不良事件，则应记录于 CRF 内。

5.5. 全面中止试验标准

1) 研究进行中由于以下原因整个试验在多中心全面停止：
   - 基于 DSMB 中期分析结果建议；
   - 研究者发现严重安全性问题；
   - 方案有重大失误；
   - 资助方因经费或管理原因；
   - 行政主管部门撤消试验，均可中途停止全部试验。
2) 全面中止试验可是暂时的，也可是永久的。中止试验时，全部试验记录应予保留备查。

6. 治疗

6.1. 试验用药物

6.1.1. 药物来源

试验药品名称：芪苈强心胶囊 Qiliqiangxin Jiaonang

成份：黄芪、人参、附子、丹参、葶苈子、泽泻、玉竹、桂枝、红花、香加皮、陈皮。

性状：胶囊剂，内容物为棕褐色至黑褐色的颗粒，味苦。

规格：0.3g/粒

批号：国药准字 Z20040141

生产单位：石家庄以岭药业股份有限公司

安慰剂：芪苈强心胶囊模拟剂（模拟剂与试验药品在颜色、规格、包装、标签、内容物形状等方面完全一致）

上述所有试验药物、模拟剂均由石家庄以岭药业股份有限公司免费提供，并出具合格药检报告。

6.1.2.　制剂、包装与标签

芪苈强心胶囊将以 0.3g 胶囊制剂形式提供，安慰剂外观上与其完全一致。

药物包装

小包装：外观如下，印有“临床试验用药”字样，每小盒内以铝塑板装 36 粒药。

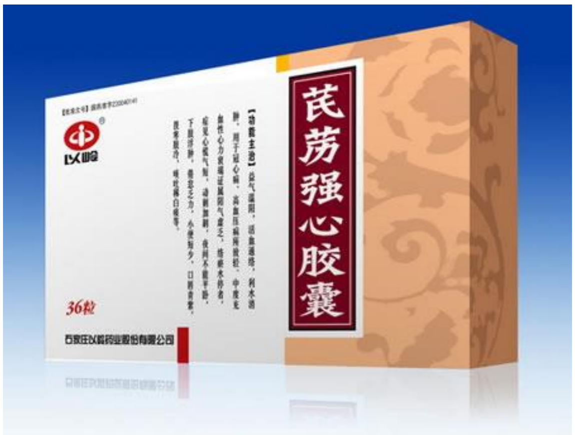

大包装：为 29.5cm×12cm×22cm 的白板纸盒，每一个大包装内含 33 个小包装，每个大盒贴有如下标签：

**芪苈强心胶囊对慢性心衰复合终点事件的评估研究用药**<br>**（仅供临床研究使用）**
**药物包装号：XXXXX**<br>【产品批号】xxxx　　　【有效期至】xxxx
【国药准字】Z20040141<br>【包　　装】每小盒装 36 粒，内装 33 小盒（一名受试者 99 天用量）<br>【用法用量】每日 3 次，每次 4 粒<br>【贮　　存】密封，在干燥处、儿童不易触及处保存
**请您务必遵照医生的医嘱和指定日期到医院就诊访视，谢谢合作！**

6.1.3.　保存方法

试验药物应当在安全可控的室内区域内上锁保存，注意防潮。每个试验中心必须指定研究用药管理负责人保存、管理。

6.1.4.　药物的发放与回收

每次随访研究者将药物发给受试者时，需填写药品发放登记表，及时、准确记录发放的药品数量并提醒受试者下次就诊时将其剩余药物带回；回收并核对返还药品的数量，记录于药品发放/返还登记表中。试验结束时，全部剩余药品应返还给申办者，并填写研究用药回收表格，未发放的药品在返还时必须是密封的。试验结束后剩余药物由监查员回收，统一处理。

6.1.5. 药物清点

自服药后受试者应按照访视计划归还所有剩余药品，研究者清点剩余药品数量并记录，用以判断依从性。

6.2. 治疗方案

6.2.1. 研究流程：

入组期（第-14 天~第 0 天）：

患者如果符合本方案中标准治疗的规定，可在此期间完成所有检查，符合入选、排除标准的患者进入随机分组阶段。

随机治疗期（第 0 天~12 个月（最长 36 个月））：（以发药时间记为 0 天）

试验组：慢性心衰标准化治疗+芪苈强心胶囊（4 粒/次 3 次/日 口服）

对照组：慢性心衰标准化治疗+芪苈强心胶囊模拟剂（4 粒/次 3 次/日 口服）

研究药物推荐于每日三餐后约 30 分钟时服用。访视日早晨勿服用药物。如果患者某日未服药，次日服药剂量不得超过日剂量，本研究不允许进行剂量调整。如果患者出现无法耐受的不良事件，并且根据研究者考虑与研究药物相关，则患者应当终止研究药物治疗。

6.2.2. 基础治疗

参照当地 HF 治疗指南或中华医学会心血管病学分会发布的“中国心力衰竭诊断和治疗指南 2014”规定如下：

1)　入选前至少 2 周以上未静脉使用利尿剂、强心剂及血管扩张剂。

2)　进入随机分组阶段至少 2 周前，患者应当接受慢性心衰标准化治疗，所有药物已经调整至固定剂量，标准化药物治疗包括：血管紧张素转换酶抑制剂（ACEI）或血管

紧张素受体拮抗剂（ARB）或血管紧张素受体脑啡肽酶抑制剂(ARNI)、β受体阻滞剂及醛固酮受体拮抗剂（除非禁忌或不耐受，应达到最佳治疗剂量）；

3) 进入治疗期后每一位患者使用药物种类、剂量需延续入选前标准化治疗方案。整个治疗期间原则上不能再调整；若属医疗需要调整用药，需记录在病例报告表中，增加或减少药物剂量、种类的患者需记录复合终点事件和不良事件。

6.2.3. 合并用药

1) 接受较好地控制高血压、心绞痛、糖尿病或其他疾病的药物治疗。
2) 进入随机分组阶段之后整个治疗期均不得使用研究药物以外的其他与试验药物成分类似中药。
3) 患者应当接受有利于心脏健康的饮食指导，如低盐饮食，适量饮水等，患者同时应当接受诸如监测体重、体育锻炼、戒烟、戒酒等适当生活方式改善的咨询。
4) 目前尚未发现禁止与芪苈强心胶囊伴随使用的药物。

6.2.4. 疗程：12 个月-36 个月。

6.3. 依从性评价

通过完整记录药物的分发和回收情况来对受试者进行依从性评价，实际服用药物量在应用药物量的 80%~120%范围内，可判定为用药依从性符合方案要求。

6.4. 药物不良反应

目前，尚未发现芪苈强心胶囊存在明显的不良反应。

7. 临床观察终点和指标

7.1. 临床观察指标

7.1.1. 主要有效性终点

- 心血管死亡和心衰恶化再住院组成的复合终点事件发生率；

7.1.2. 次要有效性终点

- 全因死亡率
- 复合终点事件（心衰恶化放弃治疗、心脏骤停后复苏成功、恶性心律失常、非致死性卒中）
- 冠心病心衰患者的心血管死亡和心衰恶化再住院发生率
- 血清 NT-proBNP 下降率

注：全部终点事件需经事件判定委员会复核判定。

7.2. 安全性指标包括：

- 不良事件评价
- 临床实验室指标：血常规（血红蛋白、红细胞、白细胞、血小板）、尿常规（尿蛋白、尿白细胞、尿红细胞）、血清生化（尿素氮、肌酐、血尿酸、总蛋白、白蛋白、谷丙转氨酶、谷草转氨酶、碱性磷酸酶、总胆红素、空腹血糖、钾、钠、氯、总胆固醇、甘油三酯、低密度脂蛋白、高密度脂蛋白）。
- 12 导联心电图
- 体格检查

8.　试验过程

所有患者包括研究完成前停用研究药物，都应继续参加表格所列的计划访视，直至研究结束。如果某次访视被推迟或者提前，不应影响下次访视。下次访视应遵守原计划时间进行。

试验流程表

访视	1	2	3	4	5	6	7	8	9	10	11	12	13	14	UNS	EOS
天/月	0天	1M	3M	6M	9M	12M	15M	18M	21M	24M	27M	30M	33M	36M		
	-14	±3	±7	±7	±7	±7	±7	±7	±7	±7	±7	±7	±7	±7		≧2周
知情同意书	×															
入选/排除标准	×															
中央随机	×															
一般资料/病史	×															
心衰病史	×															
心血管病史	×															
体格检查	×	×[1]	×[1]	×[1]	×[1]	×	×[1]	×[1]	×[1]	×	×[1]	×[1]	×[1]	×	(×)	×
心衰用药	×	×	×	×	×	×	×	×	×	×	×	×	×	×	(×)	×
CV用药	×	×	×	×	×	×	×	×	×	×	×	×	×	×	(×)	×
其他用药	×	×	×	×	×	×	×	×	×	×	×	×	×	×	(×)	×
心脏彩超	×*															
妊娠试验	×					×				×				×	(×)	×
血/尿常规	×	×				×				×				×	(×)	×
生化检查	×	×				×				×				×	(×)	×
12导联心电图	×*	×				×				×				×	(×)	×
当地实验室血清NT-proBNP	×															
中心实验室血清NT-proBNP	×	×		×												
分发药物	×		×	×	×	×	×	×	×	×	×	×	×	×		
回收药物	×		×	×	×	×	×	×	×	×	×	×	×	×		×
终点事件		×	×	×	×	×	×	×	×	×	×	×	×	×	(×)	×
不良事件		×	×	×	×	×	×	×	×	×	×	×	×	×	(×)	×

- ×[1]为简化体格检查
- ×*心脏彩超和 12 导联心电图接受入组前 6 个月内结果。
- UNS(计划外访视中):（×）标记项目为可选择操作，根据研究者判断进行。
- EOS（终末访视）:根据研究结束时间安排进行（如在试验结束前一个月内有过访视则视为终末访视但需补充完整终末访视所需项目）。

- 妊娠试验仅适用于育龄妇女（如尿妊娠试验阳性则必须进行血清妊娠试验证实）。

9. 终点事件的含义及 CEC 判定规程

9.1. 本研究终点事件的含义

- **心力衰竭住院是指满足下列所有标准的事件：**

1.患者因初步诊断为 HF 住院

2.患者住院时间延长至少 24 小时（或者如果不能获得住院时间和出院时间，指在日历上日期发生的变化）

3.在患者报告上记录由于 HF 出现新症状或者恶化症状，至少包括以下情况之一：

a.呼吸困难（用力时呼吸困难、休息时呼吸困难、端坐呼吸、夜间阵发性呼吸困难）

b.运动耐量减少

c.疲劳

4.患者具有新出现的恶化 HF 的客观证据，包括至少两种体检结果或一种体检结果和至少一种实验室标准，包括：

a.判断由 HF 导致的体检结果，包括新出现或恶化的：

1)外周性水肿。 2)腹胀或腹水增加（在无原发性肝病的情况下）。 3)肺啰音/爆裂音/湿性啰音。 4)颈静脉压升高和/或肝颈静脉回流。 5)S3 奔马律。 6)具有临床意义的或迅速的体重增加，考虑与体液潴留有关

b.在 24 小时内获得的，新出现或者恶化 HF 的实验室证据，包括：

1)与 HF 失代偿一致（如 BNP>500pg/ml 或 NT-proBNP>2000pg/ml）的 B 型利钠肽（BNP）/N 末端 B 型利钠肽前体（NT-proBNP）浓度增加。在利钠肽长期升高的患者中，应特别关注超过基线的显著增加。 2)肺充血的放射影像学证据。3)具有临床意义的左侧或者右侧心室充盈压升高或者心输出量降低的非侵害性诊断证据。如超声心动图标准包括：E/e′>15 或者 D 主导肺静脉流入模式，充血性下腔静脉伴有极小程度吸气塌陷，或者左心室流出量（LVOT）微小行程距离减小（时间流速积分（TVI））。4)侵入性诊断证据：右心导管检查显示肺毛细血管楔压（肺动脉闭塞压）≧18mmHg，中央静脉压≧12mmHg，或心排血量指数

注：如适用，即使不满足上述标准，仍需报告诊断检查中的所有结果，为上述事件的裁定提供重要信息。

5.患者接受针对 HF 的初期或者强化治疗，包括下列至少一种：

a.增强口服利尿药的治疗

b.静脉注射利尿药或者血管活性药物（如正性肌力药、血管升压类药物或者血管扩张剂）

c.机械或手术干预，包括：

1)机械循环支持（例如，主动脉球囊反搏、心室辅助装置、体外膜氧合、全人工心脏）

2)机械辅助去除体液（例如，超滤、血液滤过、透析）

- **心血管死亡：**包括急性心肌梗死（MI），心源性猝死，心力衰竭（HF）导致的死亡，中风导致的死亡，心血管（CV）手术导致的死亡，CV 出血以及其他 CV 原因造成的死亡。
- **全因死亡**
- **心衰恶化放弃治疗：**心衰症状和体征不断加重，需要静脉药物或机械支持治疗而患者或患者家属主动放弃治疗或自动出院，若随访其后果为死亡则列入心力衰竭死亡。
- **心脏骤停后复苏成功**
- **恶性心律失常：**对于恶性心律失常的定义， 目前还没有统一的标准，一般是指能在短时间内引起严重血流动力学障碍，导致患者晕厥甚至猝死的心律失常 。根据这个标准，恶性心律失常主要有如下类别：(1) 严重的缓慢型心律失常， 如严重的病态窦房结综合征、高度或三度房室传导阻滞；(2) 快速型心律失常， 如持续性室性心动过速、心室扑动、心室颤动， 快室率心房扑动、心房颤动、房室折返性心动过速、预激综合征伴心房颤动、窦性心动过速等。
- **非致死性卒中**

9.2. 终点事件发生时的评估与程序

研究者获知终点事件发生后，应在 7 天内收集相关支持文件报告事件判定委员会。终点事件将由独立的事件判定委员会（CEC）进行复核，因此终点事件报告表将作为 CRF 的一部分，研究者将在上述表格内记录事件并及时提交支持文件（入院与出院记录、病历记录、死亡记录、ECG 等）上述资料将提供给 CEC 以对事件进行判定。

CEC 由主席及 5-6 名成员组成，每一例事件将由委员会的两位成员进行独立审查并将结论提交至委员会主席处。如果两位审查委员之间或两位审查委员与主席的意见

不一致，当具有异议的事件积累到一定数量时，整个委员会将安排会议对事件进行审查。

10. 不良事件的观察

10.1. 定义：

- 不良事件（**AEs**）：自受试者签署知情同意书并入选试验后开始至最后一次随访之间，发生任何不利医疗事件，无论与试验药物是否有因果关系，均判定为不良事件。
- 重要不良事件：除严重不良事件外，发生的任何导致针对性医疗措施（如停药，降低剂量和对症治疗）的不良事件和血液学和/或其他实验室异常。

10.2. 不良事件强度判定标准：

在本临床研究中发生的所有临床不良事件将记录在 CRF 不良事件页上。并将不良事件强度进行分级。为统一标准，事件强度分级如下：

轻度	可察觉的不适感但是不影响日常活动
中度	不适感较强以致影响或减少日常活动
重度	无法工作或进行日常活动

注意区别不良事件的严重程度和强度。重度用来描述强度，不一定是严重不良事件（SAE）。例如头痛可能在强度上表现为重度，但不能列入严重不良事件，除非它符合 SAE 标准。

10.3. 不良事件与研究药物关系的判断标准

对所有不良事件与试验药物关系的因果分析，均按肯定有关、很可能有关、可能有关、可能无关、肯定无关五级进行判断，对前三种定为药物的不良反应。因果分析的考虑因素有以下五个方面：

1）开始用药时间和可疑药物不良反应（Adverse Drug Reaction，ADR）出现的时间有无合理的先后关系（用药出现）

2）所怀疑的 ADR 是否符合该药已知的 ADR（符合文献）。

3）所怀疑的 ADR 能否用合并用药、曾用药、病人的临床情况，或其他疗法的影响来解释（其他解释）。

4）停药或减量后可疑的 ADR 是否消失或减轻（停药反应）。

5）在此接触同样的药物后，可疑的 ADR 是否再次出现（再用再现）。

研究者应对不良事件和试验药物以及合并药之间可能存在的关联作出评估，参照下表

考虑因素	用药出现	符合文献	其它解释	停药消失	再用再现
肯定有关	＋	＋	－	＋	＋
很可能有关	＋	＋	－	＋	?
可能有关	＋	＋	±	±	?
可能无关	＋	－	±	±	?
肯定无关	－	－	＋	－	－

10.4. 严重不良事件的判定

10.4.1. 一般严重不良事件定义

严重不良事件是指任何提示显著危害、禁忌症、副作用或者需谨慎的临床事件。不良事件符合下面一条或以上标准时归为严重不良事件：

- 死亡
- 有生命危险（指出现该事件的患者在事件发生当时存在立即死亡的风险；并不包括那些如果更加严重将有可能导致患者死亡的事件）
- 导致住院或住院时间延长
- 导致持久或显著的劳动力丧失或残疾
- 先天性畸形缺陷

有些还没有导致死亡、生命危险或需住院的医疗事件，经过适当的医学判断，认为其可能对病人或受试者造成危害或需药物或外科手术治疗以避免上述情况发生时，也应视为 SAE。

10.4.2. 严重不良事件的研究特异性定义

在本试验中，下列事件将不会作为严重不良事件报告，除非被判定阴性并且研究者认为与研究用药相关

◆ 心血管死亡

◆ 心衰恶化再住院

◆ 心衰恶化放弃治疗

◆ 心脏骤停后复苏成功

◆ 恶性心律失常

◆ 非致死性卒中

但是，所有其他导致致死性结局的事件都将被作为严重不良事件报告。

10.5. 不良事件的随访与记录

出现的不良事件，尤其是那些与试验药物相关的事件应当随访直至它们恢复至基线状态或者趋于稳定。如果经过随访，仍无法恢复基线状态或者稳定，那么应当在 CRF 中记录说明。临床试验过程中的任何严重不良事件，必须在 24 小时内报告临床试验监查人员、主要研究单位、药品生产企业。同时研究者必须填写严重不良反应表，记录严重不良事件的发生时间、严重程度、持续时间、采取的措施和转归。

10.6. 实验室结果异常

研究者应当对实验室结果异常是否具有临床意义进行判断，并给出可能的解释。已经被报告的不良事件导致的实验室异常结果应同时作为不良事件记录在不良事件表中。具有临床意义的实验室检查异常满足以下一项或多项条件者，应作为独立诊断记录在 CRF 的不良事件页中（不包括已被报告的不良事件导致的实验室结果异常）：

- 伴有临床症状的
- 导致研究用药改变的
- 需要改变合并用药和（或）其他治疗措施的

11. 盲法与随机化

11.1. 受试者随机分配方法

由北京大学临床研究所统计专业人员，在计算机上用 SAS9.4 统计软件包，按试验组与对照组 1:1 的比例用区组随机化方法生成随机编号。根据此随机编号由与本试验无关的人员对研究药物（芪苈强心胶囊或安慰剂）进行包装编码。

本研究采用随机化与试验药物管理系统（RTSM），统计专业人员将向RTSM提供随机编号列表。然后，由RTSM给患者分配随机编号。

在完成基线评估后，在基线访视通过RTSM分配随机编号。此后，按照访视计划通过RTSM获取药物编号，每次分配的药物编号均不相同，但药物是同一种。在患者随机分组前，研究者必须先登录RTSM，并且提供一些信息（例如受试者出生日期、性别等）。

11.2. 紧急揭盲

如果发生不良事件，只有在必须了解研究药物的使用才能治疗患者的特殊情况下，才能进行紧急揭盲。一旦决定揭盲，研究者必须记录日期、时间和破盲的原因。

研究者需要登录 RTSM 填写破盲申请，由主要研究者审核后再由破盲员破盲。一旦

破盲，该病例将中止研究，作为退出处理。

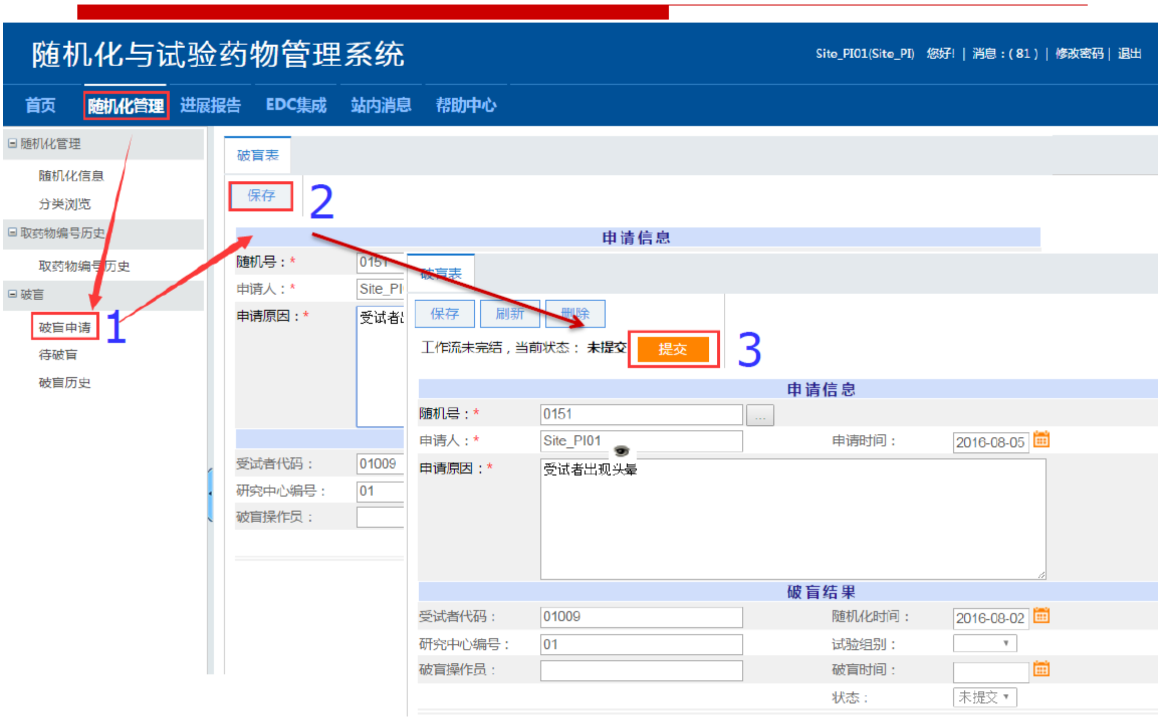

11.3. 揭盲规定

本研究采用二次揭盲法。在经盲态核查后，数据锁定，由主要研究者、医学统计专家、数据管理员、申办单位代表进行第一次揭盲，将各随机号所对应的组别以 A，B 为代号标出，以便对全部数据进行统计分析。当统计分析结束，统计报告完成时，再进行第二次揭盲，宣布 A，B 两组的确切组别。

11.4. 筛选编号

各医院按收治患者的先后顺序编排筛选号，筛选号由中心编号+3 位整数表示，如，01 中心筛选号 01001，01002，……。

12. 统计分析

试验方案确定后，由统计专业人员负责与主要研究者协商制订统计分析计划书。统计分析软件采用 SAS®9.4 软件（或更高版本）。样本量计算软件采用 PASS13。

12.1. 分析人群

研究人群分为以下几类：

- 全分析集(FAS)：是指尽可能接近意向性分析原则（intention to treat）、从所有随机化的受试者中，以最少的和合理的方法剔除受试者后得出的数据集，包含所有

经过随机化并使用过一次研究药物的受试者。剔除通常包括：违反重要入组标准；受试者未接受试验用药物的治疗；随机化后无任何观测数据。主要疗效评价指标为发生复合终点事件的时间，采用生存分析的方法进行分析，在选择 FAS 进行统计分析时，对于主要终点事件的缺失按照删失处理。

- 符合方案集(PPS)：是全分析集的一个子集，这些受试者对方案更具依从性。纳入 PPS 受试者一般具有以下特征：（1）完成事先设定的试验药物的最小暴露量，即服用药物的依从性达到 80%；（2）试验中主要指标的数据均可以获得；（3）未对试验方案有重大的违背。
- 安全性分析集（SS）：所有随机化后至少接受一次治疗且有安全性评价的受试者。安全性缺失值无需结转。

疗效分析将在 FAS 和 PPS 的基础上进行。所有基线人口统计学资料分析将在 FAS 的基础上进行，安全性评价将在 SS 上进行。

12.2. 统计分析方法

- 所有的统计检验均采用双侧检验，*P* 值小于或等于 0.05 将被认为所检验的差别有统计意义。（特别说明的除外）
- 描述性分析：分类指标描述各类的例数及百分数。定量指标采用均数、标准差、最大值、最小值、中位数、下四分位数（Q1）和上四分位数（Q3）描述。
- 对两组一般情况的比较将根据指标的类型采用适当的方法进行分析，定量资料的组间比较采用成组 t 检验或 Wilcoxon 秩和检验，分类数据采用卡方检验或精确概率法，等级资料采用 Wilcoxon 秩和检验或 CMH 检验。

12.2.1. 入组及完成情况：

总结各中心入组及完成数，列出脱落病例的清单。各组不同数据集大小，各中心病例分布，总脱落率比较，终止原因详细列表。对患者的人口学特征(年龄、身高、生命体征等)、病史及用药史等进行描述，并对两组年龄、身高、体重等进行比较，以衡量两组的可比性。

12.2.2. 依从性分析：

- 用药依从性分析：比较两组病人是否按时按量使用试验药物，未用方案中禁用的药物和食物。
- 合并用药分析：需统计各组合并用药人数，并详细列表。

12.2.3. 疗效评价：

- 疗效评价同时进行 PP 分析和 FAS 分析；
- 主要疗效评价指标为发生复合终点事件（心血管死亡和心衰恶化再住院）的时间。对于主要终点事件的缺失按照删失处理。

  本研究的主要研究假设为：

  H_0：$\lambda_T/\lambda_C \geq 1$

  H_1：$\lambda_T/\lambda_C < 1$

  其中，λ_T和λ_C分别为试验组和对照组发生终点事件的风险。

  利用 Kaplan-Meier 法估计临床终点事件发生率，两组之间进行 Log rank 检验。利用 COX 比例风险模型，以中心为协变量，计算两组间的风险比（Hazard Ratio）及其 95%可信区间。另外，对复合终点事件的两个部分分别进行分析，即心血管死亡和心衰恶化再住院。
- 次要疗效指标：

  全因死亡率：利用 Kaplan-Meier 法估计两组全因死亡率，并进行 Log rank 检验。利用 COX 比例风险模型，以中心为协变量，估计两组间的风险比（Hazard Ratio）及其 95%可信区间。

  复合终点事件（心衰恶化放弃治疗、心脏骤停后复苏成功、恶性心律失常、非致死性卒中）：分析方法与全因死亡相同。

  冠心病心衰患者的心血管死亡和心衰恶化再住院事件：按计数资料统计分析；

  血清 NT-proBNP：按计量资料分析，对两组血清 NT-proBNP 水平进行统计描述和组间比较，并对两组与基线的变化情况进行统计描述和组间比较。

12.2.4. 安全性评价：

安全性评价基于 SS 数据集进行分析。

不良事件用不良事件发生例次、例数及发生率进行描述，并对该发生率进行组间显著性检验。同时，列表详细描述各组病例出现的全部不良事件的具体表现、程度及其与药物的关系。

对实验室指标前后变化情况进行交叉表描述，按试验组和对照组分别描述治疗前正常、治疗后异常例数及该例数所占比例。对生命体征指标进行前后比较。

12.3. 期中分析

本研究计划在收集到1/2和2/3主要终点事件后进行两次期中疗效分析，以评估是否已得出有效的结论而提前终止该研究。根据 Lan-DeMets α消耗函数和O’Brien-Fleming方法，第1次期中分析时消耗的I类错误α=0.0001（单侧），第2次期中分析消耗α=0.00605（单侧）。

期中分析有关的具体要求和操作将在DSMB章程中事先规定。

13. 数据管理

本研究采用 Epidata 软件进行研究数据的采集。数据管理确保临床试验数据的真实性、完整性和准确性，数据管理过程需符合《药物临床试验质量管理规范》、《临床试验数据管理工作技术指南》等法规要求，保证临床试验数据的可溯源性。以下列出数据管理的主要流程。

13.1. 数据库设计

数据管理员根据CRF采用Epidata软件设计数据库，经测试后发布。

13.2. 数据录入

CRC负责将CRF中的数据录入数据库，数据录入采用二次录入方式，由两名CRC分别录入一遍数据，数据管理员对两个数据库进行比对，产生数据不一致清单，CRC按照清单对照CRF分别修改各自的数据库，然后再进行比对，重复以上步骤，直至两个数据库完全一致。

13.3. 数据质疑管理

数据管理员依据数据核查计划（DVP）编写数据核查SAS程序对数据进行核查，产生数据质疑清单，经人工核对后，生成数据质疑表，由CRA交研究者进行答疑，答疑后的质疑表再由CRA返还给数据管理员，数据管理员据此修订数据库。

13.4. 医学编码

不良事件编码采用MedDRA21.0或者更新版本。

13.5. 数据审核

数据库清理完成后，数据管理员撰写《数据核查报告》，用于召开数据核查会议。

审核报告重点记录内容为：入组病例数、脱落、剔除病例情况、偏离或违背方案情

况、依从性数据，合并用药，不良事件，与评价指标有关的数据等。

数据审核会议上，针对审核报告的内容，讨论并确定统计人群的划分。

13.6. 数据库锁定

完成数据库锁库清单，依据数据库锁定程序完成数据库锁定。数据锁定之后发现的问题，经确认后可在统计分析程序中修正。数据锁定后如有确切证据证明有必要解锁，研究者及相关人员需签署解锁文件。

数据库锁定后，由数据管理员导出 SAS 格式的数据文件，交与统计人员进行统计分析。

14. 质量控制

1) 研究者应履行各自职责，并严格遵循临床研究方案，采用标准操作规程，对所有相关观察结果和发现都应加以核实，以保证临床研究的质量控制和质量保证系统的实施。
2) 临床研究中受试者分配必须按研究设计确定的随机分配方案进行，每名受试者的处理分组编码应作为盲底由统计单位和研究者分别保存。
3) 研究者须对参加临床研究的所有人员进行必要培训，说明有关的资料、操作规范和职责，保证将数据真实、准确、完整、及时、合法的记入病历和 CRF。CRF 必须由专人负责保管。
4) 监查员应遵循标准操作规程，督促研究方案的执行情况，确认所有数据记录与报告正确完整，所有 CRF 填写正确，并与原始资料一致。
5) 稽查人员应对临床研究相关活动和文件进行系统性检查，以评价研究是否按照方案、标准操作规程以及相关法规要求进行。
6) 临床研究中各种实验室检查数据必须准确，并应记录在案或将原始报告复印件粘贴在病例报告表上。
7) 医学统计人员应把研究数据完整、无误地纳入报告，所有涉及数据管理的各种步骤均需记录在案，以便对数据质量及实施过程进行检查。
8) 临床研究资料的统计分析过程及其结果的表达必须采用规范的统计学方法。临床研究各阶段均需有医学统计人员参与。临床研究总结报告必须与统计报告相符。
9) 各方应严格按批准方案进行临床研究，任何偏离方案的情况均需记录在案。研究方案的修改需制定修改说明，并报伦理委员会批准方可执行。

10) 各研究中心设研究负责人 1 名，固定研究组成人员若干人。严格按临床研究方案要求进行。组长单位技术人员随时与各研究中心保持密切联系，并于研究早、中、后期前往各研究中心检查病例观察记录情况，及时解决可能出现的问题。

15. 伦理相关事宜

1. 在临床研究的过程中，必须对受试者的个人权益给予充分的保障，并确保研究的科学性和可靠性。受试者的权益、安全和健康高于对科学和社会利益的考虑。
2. 研究方案需经伦理委员会审议同意并签署批准意见后方可实施。在研究进行期间，研究方案的任何修改均应经伦理委员会批准；研究中发生严重不良事件，应及时向伦理委员会报告。
3. 研究者或其指定的代表必须向受试者说明有关临床研究的详细情况，经充分和详细解释研究的内容后获得知情同意书。

16. 试验进度

2018 年 7 月	完成试验方案的制定，召开筹备会
2018 年 8 月	方案修改，通过伦理审批
2018 年 8 月	试验药物、资料准备
2018 年 9 月	试验国际注册、分中心启动
2018 年 9 月	第 1 例病例筛选入组
2020 年 9 月	完成所有病例的随机入组
2021 年 9 月	各中心完成所有病例的随访工作
2022 年 2 月	完成数据输入及盲态审核
2022 年 4 月	统计分析工作
2022 年 6 月	完成试验总结报告

17. 资料保存

研究医院应保存这些原始资料至临床试验终止后 5 年，包括对所有参加受试者的确认（能有效的核对不同的记录资料，如 CRF 和医院原始记录）、所有原始受试者知情同意书、CRF 表、药品分发的详细记录等。

18. 临床总结

统计分析结束后由试验主要研究者负责写出本研究临床总结报告并加盖主要研究单位公章。

参考文献：

1) Lopez AD. Assessing the burden of mortaIity from cardiovascuIar diS-eases[J]. WorId Health Star Q, 1993, 46(2):91-96.
2) 中华医学会心血管病学分会. 慢性心力衰竭诊断治疗指南[J]. 中华心血管病杂志, 2007, 35(12):1076-1095.
3) 陈灏珠.实用内科学[M].北京：人民卫生出版社，2001.
4) World health organization. Cardiovascular disease:prevention and control[EB/OL]. http://www.who.int/dietphysicalactivity/ publications/facts/cvd/en/. 2010-03-07.
5) Mendez GF, Cowie MR. The epidemiological features of heart failure in developing countries:a review of the literature[J]. Int J Cardiol, 2001, 80(2-3):213-219.
6) Rosamond W, Flegal K, Friday G, et al. Heart disease and stroke statistics - 2007 update - A report from the American Heart Association Statistics Committee and Stroke Statistics Subcommittee[J]. Circulation, 2007, 115(5):E69E171.
7) 顾东风, 黄广勇, 吴锡桂,等. 中国心力衰竭流行病学调查及其患病率[J]. 中华心血管病杂志, 2003, 31(1):3-6.
8) Roger VL, Weston SA, Redfield MA, et al. Trends in heart failure incidence and survival in a community-based population[J]. JAMA, 2004, 292(3):344-350.
9) Pulignano G, Del S D, Tavazzi L, et al. Clinical features and outcomes of elderly outpatients with heart failure followed up in hospital cardiology units: data from a large nationwide cardiology database (IN-CHF Registry).[J]. American Heart Journal, 2002, 143(1):45-55.
10) Kawashiro N, Kasanuki H, Ogawa H, et al. Clinical characteristics and outcome of hospitalized patients with congestive heart failure: results of the HIJC-HF registry[J]. Circulation Journal Official Journal of the Japanese Circulation Society, 2008, 72(12):2015.
11) Stewart S, Macintyre K, Hole D J, et al. More 'malignant' than cancer? Five-year survival following a first admission for heart failure.[J]. European Journal of Heart Failure, 2014, 3(3):315-322.
12) Cowie M R, Wood D A, Coats A J, et al. Survival of patients with a new diagnosis of heart failure: a population based study.[J]. Heart, 2000, 83(5):505-10.
13) Mcmurray J J, Packer M, Desai A S, et al. Angiotensin-neprilysin inhibition versus enalapril in heart failure.[J]. New England Journal of Medicine, 2014, 371(11):993-1004.
14) Swedberg K, Komajda M, Böhm M, et al. Ivabradine and outcomes in chronic heart failure (SHIFT): a randomised placebo-controlled study.[J]. Lancet, 2010, 376(9758):2069-2069.
15) Li X, Zhang J, Huang J, et al. A multicenter, randomized, double-blind, parallel-group, placebo-controlled study of the effects of qili qiangxin capsules in patients with chronic heart failure[J]. Journal of the American College of Cardiology, 2013, 62(12):1065-1072.
16) 吴以岭. 脉络论[M]. 中国科学技术出版社, 2010.
17) 《芪苈强心胶囊对慢性心衰大鼠心功能与肾脏水通道蛋白 2 的影响》南方医科大学附属南方医院心内科主任许顶立 2009 西安 全国心力衰竭学术会议.
18) 李佳彧, 杨萍. 芪苈强心胶囊对心梗后心力衰竭大鼠 periostin 蛋白表达干预作用的研究[J]. 中国实验诊断学, 2009, 13(2):170-172.
19) 李娅, 宋优, 程翔,等. 芪苈强心胶囊对大鼠心肌梗死后心肌重构及心功能的影响[J]. 中国分子心脏病学杂志, 2007, 7(4):201-204.
20) 宋优, 李娅, 程翔,等. 芪苈强心调节急性心肌梗死大鼠心肌 TNF-α 和 IL-10 表达[J]. 中国免疫学杂志, 2007, 23(9):806-810.

1　附 1：NYHA 分级评分标准

NYHA 心功能分级评分标准：

Ⅰ级：体力活动不受限，日常活动不引起疲乏、心悸或呼吸困难。

Ⅱ级：体力活动轻度受限，休息时无症状，日常活动可引起疲乏、心悸或呼吸困难。

Ⅲ级：体力活动明显受限，休息时无症状低于日常活动量即出现症状。

Ⅳ级：不能进行任何体力活动，休息时即出现不适，任何体力活动都使症状加重。

2　附 2：NT-proBNP 检查采血及血样保存及运输流程

血样制备检验及冷链运送标准操作规程

1．目的

规范血样保存、运输、接收条件与过程

2．适用范围

随机、双盲、安慰剂平行对照评价芪苈强心胶囊治疗慢性心衰有效性与安全性的多中心临床试验血样制备检验及运送

3．简单流程

血样品制备 ——————

↓

保存　　负责方（研究者）

↓

交接 ——————

↓

运输　　负责方（快递公司）

↓

接收 ——————

↓

化验 ——————

4．制备与保存

各中心血液应在采集 2 小时之内按照血液标本制备与保存标准操作规程制备血样标本，血样品分别置于冷冻管中，按照药物的编号，将不干胶标签的其中两联贴于冷冻管上，另一联粘贴于留样记录表（表 1）上，并按要求填写留样记录表。

NT-proBNP 标本制备流程

每人抽取静脉血 3ml

↓

EDTA 抗凝

↓

离心后吸取上层血浆

↓

标记好患者标签

↓

放入-20℃冰箱保存

↓

所有标本应在采集完成后 3 个月内送检

注意：

1） 应在无炎症或感染条件下（代谢稳定）进行测定，以减少个体差异；

2） 病人准备：空腹 12 小时以上，在上午 8：00～9：00 取坐位采集静脉血。

3） 采血要求：使用含分离胶真空采血管（一般是黄帽）采集静脉血液 6ml，避免有溶血和脂血现象（若有请注明）。

4） 血清分离：标本采集后 30 分钟后，2 小时内 3000 离心 5 分钟，取分离后的上层液（血清），使用无任何添加物的采血管（一般是红帽），每份标本分成二管，每管不少于 800μl，一管送中心实验室检测，一管作为留样，上贴与登记表相同的编号（表 1），包括姓名、性别、年龄、标本采集时间和采集单位。

5） 血清标本的运送和保存：血清冷冻标本管应置于-20℃低温冰箱中，保存期最长为 6 个月，血清标本在运送时应置于干冰盒中。每日应记录冰箱温度，备查。

表 1 留样记录表及标签样稿

随机编号	患者姓名	性别	年龄	采血日期
医院（芪苈强心试验 NT-proBNP 检测）				

5．运输

研究者入组完成本中心任务数后（如果入组时间过长，可在第一例患者入选 2 个月内），拨打 XXX 快递公司统一免费下单电话：<u>400-0000-0000</u>；报出取件帐号：<u>0000000</u>，并告知具体的取件地址、联系人、联系电话、送达地址等。

请于快递公司约定的当天清点、准备好样品，快递工作人员到达医院后包装、交接样品；

研究者填写**<u>血样标本交接单</u>**并把此次运送样品的**留样记录表复印件**附上交快

递寄出，（原件由监查员下次监查时交还研究者），以备核查用。标本、留样记录表、交接单的数字应相吻合。

快递公司应于 24 小时之内送达中心实验室。

6．接收

中心实验室人员于样品送达当日进行血样核对、编码及入库；并及时填写样品交接记录。

接收时立即开箱检查，记录样品的状态。

7．检测

中心实验室应在接收样本后 10 个工作日内完成检测工作;并同时将结果反馈给组长单位及监查员。监查员在收到检验结果一个星期内应将结果反馈给相应研究中心。

联系人:以岭药业医学部 韩硕龙 电话:13582167153 邮箱 hanshuolong@126.com

8. 不同单位之间的换算

1pg/ml=0.118pmol/L　　　　pg（皮克） pmol（皮摩尔）

3 附 2：终点事件报告流程

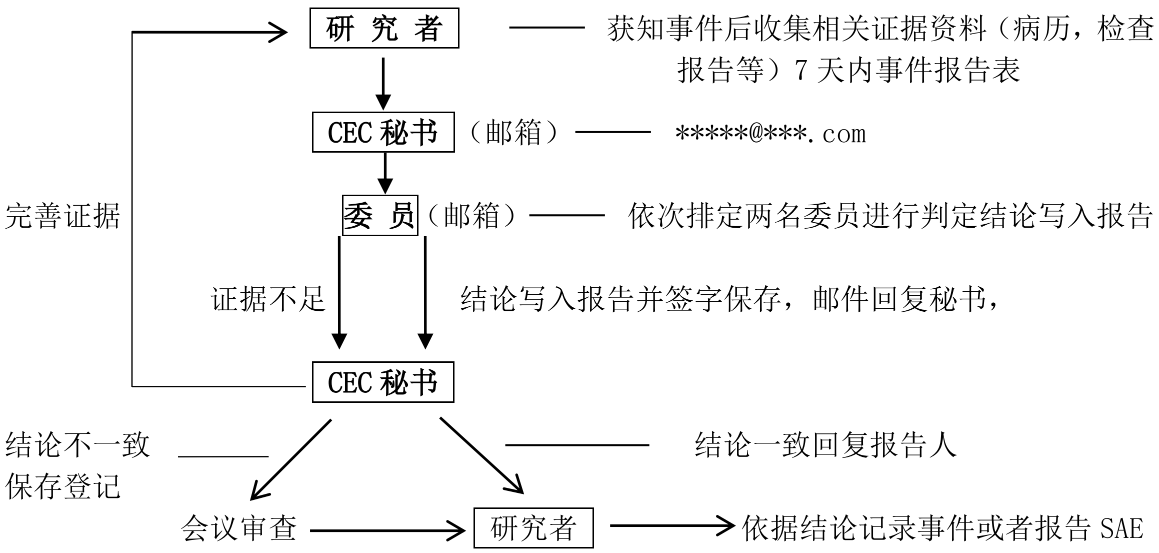

本研究方案所包含的内容为保密内容，是为临床研究人员使用。本资料为资助者或其子单位的财产，不得复印或分发给与本研究无关的人员。

芪苈强心胶囊对慢性心衰复合终点事件的评估研究

Qiliqiangxin in Heart FailUre：AssESsment of Reduction in MorTality and MorbidiTy

QUEST

研究方案

Study Protocol

试验方案编号：SP-YFC-05-QUEST

试验方案版本号：V1.0

试验方案版本日期： 2018年7月10日

美国ClinicalTrials.gov注册号：**********

主要研究者：李新立 教授

南京医科大学第一附属医院

试验计划开始结束日期： 2018年8月--2021年8月

主要研究单位及负责人

主要研究单位	主要研究者
南京医科大学第一附属医院	李新立　　签名：__________<br>日期：__________

数据统计单位及负责人

单位	负责人
北京大学临床研究所	姚晨

药品生产企业及联系方式

单位	联系人	电话	邮箱
石家庄以岭药业股份有限公司	韩硕龙	13582167153	hanshuolong@yiling.cn

学术顾问

高润霖 院士、张伯礼 院士、张　运 院士、葛均波 院士、

韩雅玲 院士、黄从新 教授、托　尼 教授（哈佛大学）

数据和安全监测委员会

主席：陈　锋 教授

成员：朱　俊 教授　唐其柱 教授　马长生 教授　蔡廼绳 教授

事件判定委员会

主席：张抒扬 教授

成员：杨新春 教授　宋　雷 教授　蔡廼绳 教授　陈　红 教授

范维琥 教授　朱文玲 教授

缩略语

ACEI	血管紧张素转换酶抑制剂
ADR	可疑药物不良反应
AEs	不良事件
AHA	美国心脏学会
AMI	急性心肌梗死
ARB	血管紧张素受体拮抗剂
ARNI	血管紧张素受体脑啡肽酶抑制剂
BNP	B 型利钠肽
CEC	事件判定委员会
CGRP	降钙素基因相关肽
CHF	慢性心力衰竭
CRA	临床研究协调员
CRC	临床研究协调员
CRF	病历报告表
CV	心血管
DSMB	数据与安全监察委员会
DVP	数据核查计划
ECG	心电图
EF	射血分数
EOS	终末访视
ESC	欧洲心脏病学会
ET	内皮素
FAS	全分析集
HF	心力衰竭
LVOT	左心室流出量
MedDRA	监管活动医学词典
MI	心肌梗死
NO	一氧化氮
NT-proBNP	N 末端 B 型利钠肽前体
NYHA	纽约心脏病学会
PPS	符合方案集
RAAS	肾素-血管紧张素-醛固酮系统
SAE	严重不良事件
SNS	交感神经系统
SS	安全性分析集
TVI	时间流速积分
UNS	计划外访视

研究方案摘要

**试验目的**	采用循证医学研究方法，以心血管死亡率和心衰恶化再住院发生率为主要研究终点，进一步阐明芪苈强心胶囊长期用药的临床疗效及安全性，明确疗效特点及适宜人群，为优化临床合理用药方案提供高质量临床证据
**试验设计**	随机、双盲、安慰剂平行对照多中心临床试验
**入选和排除标准**	**入选标准：**<br>1） 自愿参加，理解并签署知情同意书；<br>2） 年龄≥18 岁，性别不限；<br>3） 有 3 个月以上的慢性心衰病史或临床发现心衰症状 3 个月以上；慢性心衰诊断参照当地指南或中华医学会心血管病学分会发布的“中国心力衰竭诊断和治疗指南 2014”；<br>4） 心脏彩超检查提示左室射血分数（LVEF）≤40％（改良辛普森法）；<br>5） NYHA 心功能分级Ⅱ～Ⅲ，临床症状稳定，包括入选前 2 周内曾诊断为Ⅳ级者；<br>6） 血清 NT-proBNP 含量≥ 450pg/ml；<br>7） 至少已接受 2 周以上的标准化药物治疗，且未调整过给药剂量及给予静脉治疗者；标准化药物治疗包括：血管紧张素转换酶抑制剂（ACEI）或血管紧张素受体拮抗剂（ARB）或血管紧张素受体脑啡肽酶抑制剂(ARNI)、β受体阻滞剂及醛固酮受体拮抗剂（除非禁忌或不耐受，应达到最佳治疗剂量）；<br>**排除标准：**<br>1） 不符合入选标准；<br>2） 由于瓣膜病、先天性心脏病、心包疾病、心律失常所致及非心源性病因所致心衰，或肾、肝等重要脏器功能衰竭导致的心衰，及有明确肺源性或其他原因所致的右心衰、及急性心衰；<br>3） 计划于近期内行冠脉血运重建治疗或心脏再同步化治疗者，已实施心脏再同步化治疗者；<br>4） 合并肝、肾、造血系统等严重原发性疾病，肾功能异常者，肝脏转氨酶、碱性磷酸 酶超出 3 倍正常值上限，血肌酐＞2mg/dl(176.82umol/L)，血钾＞5.5mmol/L；肿瘤患者，严重神经内分泌系统疾病及精神病患者；<br>5） 存在左室流出道梗阻、心肌炎、大动脉瘤、夹层动脉瘤、致明显血液动力学

<table>
<tr><td></td><td colspan="2">改变的未修补的心脏瓣膜病患者；
6）存在心源性休克、难以控制的恶性心律失常、二度Ⅱ型以上未置入起搏器治疗的窦房或房室传导阻滞、进行性加重的不稳定心绞痛或急性心肌梗死者；
7）未获控制的高血压患者，收缩压≥180/mmHg 和/或舒张压≥110mmHg；收缩压<90mmHg 和/或舒张压<60mmHg；
8）1 个月内参加其他药物临床研究者；
9）妊娠或正准备妊娠及哺乳期妇女；
10）过敏体质者，或已知对治疗药物过敏者；
11）根据研究者判断，患者不能完成本研究或不能遵守本研究的要求（由于管理方面的原因或其它原因）。</td></tr>
<tr><td rowspan="2">疗效性指标</td><td>主要指标</td><td>心血管死亡和心衰恶化再住院组成的复合终点事件发生率</td></tr>
<tr><td>次要指标</td><td>1、全因死亡率
2、复合终点事件（心衰恶化放弃治疗、心脏骤停后复苏成功、恶性心律失常、非致死性卒中）
3、冠心病心衰患者的心血管死亡和心衰恶化再住院发生率
4、血清NT-proBNP下降率</td></tr>
<tr><td>安全性指标</td><td colspan="2">血常规、尿常规，心电图，血清生化，不良事件，体格检查</td></tr>
<tr><td>样本量</td><td colspan="2">试验组与对照组的随机分配比例为 1:1。样本量为复合终点事件的发生例数。预计需要观察到 620 例复合终点事件。
假设随访期 36 个月内对照组复合终点事件的发生率为 25%，整个试验持续大约 36 个月，招募期预计 24 个月，则预计需要入组 3080 例（每组 1540 例）受试者才可获得 620 个终点事件。</td></tr>
<tr><td>给药方案</td><td colspan="2">试验组：慢性心衰标准化治疗+芪苈强心胶囊 4 粒/次，3 次/日，口服
对照组：慢性心衰标准化治疗+芪苈强心胶囊安慰剂 4 粒/次，3 次/日，口服</td></tr>
<tr><td>疗程</td><td colspan="2">以观察事件为指标入选病人，计划入组时间 2 年，至少服药 1 年。</td></tr>
<tr><td>试验统计</td><td colspan="2">北京大学临床研究所</td></tr>
<tr><td>预期进度</td><td colspan="2">2018 年 8 月-2021 年 8 月</td></tr>
</table>

目录

1. 试验题目......8
2. 试验目的......8
3. 试验背景与原理......8
4. 试验总体设计与安排......11
4.1. 随机分组阶段（第 0 天~第 24 个月）：......11
4.2. 病例数量、分组、中心......11
5. 研究人群......12
5.1. 入选标准......12
5.2. 排除标准......13
5.3. 中止研究药物治疗......13
5.4. 退出标准......14
5.5. 全面中止试验标准......14
6. 治疗......14
6.1. 试验用药物......14
6.1.1. 药物来源......14
6.1.2. 制剂、包装与标签......15
6.1.3. 保存方法......16
6.1.4. 药物的发放与回收......16
6.1.5. 药物清点......16
6.2. 治疗方案......16
6.2.1. 研究流程：......16
6.2.2. 基础治疗......16
6.2.3. 合并用药......17
6.2.4. 疗程：12 个月-36 个月。......17
6.3. 依从性评价......17
6.4. 药物不良反应......17
7. 临床观察终点和指标......17
7.1. 临床观察指标......17
7.1.1. 主要有效性终点......17
7.1.2. 次要有效性终点......17
7.2. 安全性指标包括：......18
8. 试验过程......18
9. 疗效与安全性判定标准......19
9.1. 疗效判定标准......19
9.1.1. 主要有效性评价......19
9.1.2. 次要有效性评价：......19
9.1.2.1. 终点事件......19
9.2. 安全性判定：......21
10. 不良事件的观察......21
10.1. 定义：......21
10.2. 不良事件强度判定标准：......22
10.3. 不良事件与研究药物关系的判断标准......22
10.4. 严重不良事件的判定......23

10.4.1. 一般严重不良事件定义 23
10.4.2. 严重不良事件的研究特异性定义 23
10.5. 不良事件的随访与记录 23
10.6. 实验室结果异常 24
11. 盲法与随机化 24
11.1. 受试者随机分配方法 24
11.2. 紧急揭盲 24
11.3. 揭盲规定 25
11.4. 筛选编号 25
12. 统计分析 25
12.1. 分析人群 25
12.2. 统计分析方法 26
12.2.1. 入组及完成情况： 26
12.2.2. 依从性分析： 26
12.2.3. 疗效评价： 27
12.2.4. 安全性评价： 27
12.3. 期中分析 28
13. 数据管理 28
13.1. 数据库设计 28
13.2. 数据录入 28
13.3. 数据质疑管理 28
13.4. 医学编码 28
13.5. 数据审核 28
13.6. 数据库锁定 29
14. 质量控制 29
15. 伦理相关事宜 30
16. 试验进度 30
17. 资料保存 30
18. 临床总结 30
参考文献： 31
1 附 1：NYHA 分级评分标准 32
2 附 2：NT-proBNP 检查采血及血样保存及运输流程 32
3 附 2：终点事件报告流程 35

1.　试验题目

芪苈强心胶囊对慢性心衰复合终点事件的评估研究

2.　试验目的

采用循证医学研究方法，以心血管死亡率和心衰恶化再住院发生率为主要研究终点，进一步阐明芪苈强心胶囊长期用药的临床疗效及安全性，明确疗效特点及适宜人群，为优化临床合理用药方案提供高质量临床证据

3.　试验背景与原理

在全球范围的死亡原因中心血管疾病位列前三，严重地危害着人类的生命和健康[1,2]。慢性心力衰竭（CHF）是在器质性心脏病基础上发生泵功能衰竭、射血分数减少、循环淤血，同时出现一系列神经体液改变的一系列临床综合症[3]。心衰作为各种心脏病发展的严重阶段，正在成为本世纪最重要的心血管病症。有流行病学资料显示，目前全球心衰患者的数量已高达 2250 万，且 5 年存活率与恶性肿瘤相仿。随着流行病学的变迁和社会经济的发展，发展中国家心衰的流行病学特点与发达国家日益相近，如冠心病作为心衰的病因在我国显得越来越突出[4,5]。欧洲心脏病学会（ESC）近年来通过对 51 个国家的统计发现，在约 10 亿的人群中，至少有 1500 万例心衰患者。2007 年美国心脏学会（AHA）报道，美国的心衰患者人数已经超过 500 万，并且仍以 55 万/年的速度不断增加[6]。与我国地理位置相近、种族人群特征相似的日本发病情况与欧美国家类似。2003 年，顾东风等在我国南方和北方各 5 个省市，随机抽样调查了 15518 名成年人（年龄 35～74 岁），分析发现我国心衰患病率为 0.9%，其中男性为 0.7%，女性为 1.0%[7]。近年来，我国与发达国家的心衰患者数量都在不断增加，全球患者以每年 200 万的速度递增，发病率和患病率也在逐步上升，其重要原因之一就是社会人口老龄化，随着年龄的增长心衰发病率升高[8]。而且随着治疗水平的发展，心衰患者死亡率虽较过去有所下降，但仍然处于较高水平。美国 70 岁以上的心衰患者 1 年死亡率明显较 70 岁以下患者高（22％：13.7％）[9]。日本的心衰患者 1 年和 3 年死亡率分别为 11.3％和 29.2％[10]，在欧洲，4 年生存率仅为 50％，而且有 40％因心衰入院的患者将可能在 1 年内再次入院治疗或者死亡[11,12]。慢性心衰依然是严重威胁人类生命和生活质量的主要问题，所以国际上仍将慢性心衰作为本世纪需要解决的重要课题。

近 20 年来，人们对心衰的药物治疗理念发生了极大转变，从改善血流动力学观点进展到生物学调整的观点。现代治疗模式的重点是改善肾素血管紧张素-醛固酮系统及

交感神经系统的神经内分泌紊乱。因此，治疗的目标不仅仅是改善症状和提高生活质量，更应注重抑制和延缓心肌重构的发展，阻断恶性循环，从而降低心衰的死亡率和住院率。

心肌重构是心衰发生发展的基本机制，包括病理性心肌细胞肥大伴胚胎基因再表达、心肌细胞凋亡与坏死及心肌细胞外基质过度沉积或降解增加等。改善心肌重构对预防、控制心衰的发生、发展和改善心功能具有重要的价值。神经内分泌两个系统包括交感神经系统(SNS）、肾素-血管紧张素-醛固酮系统（RAAS）的激活和心肌重构相互促进、加重心衰的发展。ACEI、β 受体阻滞剂、ARB 和醛固酮受体拮抗剂应用的有益效果，从而说明阻断这两个系统是有效的，进一步证实了对心衰的发生和发展的这一基本机制的认识是完全正确的。心衰的发生发展机制有待于更深入的研究，从而发掘更多，更有效的抗心衰途径，在机制研究中推断治疗心衰新的靶点。

20 世纪 80 年代后期证实神经内分泌系统激活导致心肌重构是引起心衰发生和发展的关键因素。1987 年，应用血管紧张素转换酶抑制剂(ACEI)治疗心衰的临床试验 CONSENSUS，成功降低心衰患者总死亡率达 27%，以后 SOLVED、V-HeFY 等临床试验进一步证实 ACEI 能够有效改善心衰患预后。

20 世纪 90 年代中、后期的 CIBISII、MERIT-HF、COPERNICUS 研究证实β受体阻滞剂使心衰患者死亡率降低 34%～35%。此外，RALES 试验（1999 年）、EMPHASIS-HF（2011 年）研究显示醛固酮受体拮抗剂可使心衰患者死亡率降低 24%～30%。

2010 年以来能够给心衰患者带来获益的新药主要是 ARNI 和心脏窦房结抑制剂。PARADIGM-HF 试验中，8442 例射血分数降低的心衰(HFrEF)患者随机接受 ARNI 和依那普利，ARNI 组主要终点事件(心血管死亡和因心衰住院)发生率为 21.8%，显著低于依那普利组(26.5%)[13]。

另有研究显示心率加快增加心衰患者死亡率，因此心脏窦房结抑制剂受到重视。SHIFT 研究显示，与标准治疗组比较，伊伐布雷定组使心血管死亡和心衰恶化住院的相对风险降低 18%，患者左心室功能和生活质量均显著改善。[14]

利尿剂可有效缓解心衰患者的呼吸困难、消除液体潴留，改善心功能和运动耐量，合理恰当使用利尿剂是其他治疗心衰药物取得成功的关键和基础。

心衰的非药物治疗也取得重要进展：在药物治疗基础上选择合适的患者，CRT 能进一步改善其心功能和生活质量，降低死亡率。

尽管在这些年心衰治疗领域取得了一些进展，但目前心衰的患病率和病死率依然居

高不下。尚需要开拓新的治疗方法和研发新的药物以期在心衰治疗中取得突破。

中医学在防治心力衰竭的长期医疗实践中，积累了丰富的经验，对心衰的认识也在不断深化。

中药治疗心衰已有一些研究和报道，中药芪苈强心胶囊治疗心衰研究初步获得成功，该研究结果于 2013 年发表于国际心血管病 JACC 杂志上[15]，并受到国外学者的关注和好评。所有入选者均采用了标准优化治疗，加用芪苈强心胶囊组较安慰剂组，N 末端 B 型利钠肽前体（NT-proBNP）显著降低，且降幅＞30%的患者比率也显著增加，而不良反应发生率显著降低，表明芪苈强心胶囊治疗慢性心衰有效、安全，标志着中药成为心衰公认治疗药物向前迈进一大步。芪苈强心胶囊列入中华医学会心血管病学分会《中国心力衰竭诊断和治疗指南 2014》。

中药芪苈强心胶囊首次运用络病理论探讨慢性心衰的病机和治疗，提出心气虚乏是其发生的中医病机之本，络脉瘀阻是其中心环节，津液不循脉络运行渗出脉外而为水湿之邪发为水肿，瘀血水饮阻滞脉络，日久结聚成形导致心络“络息成积”是其发展加重的结果[16]，这与西医学近年提出的早期神经内分泌激活引起的血流动力学改变，进而导致心室重构是心衰发生发展的基本机制的新概念相吻合。

由于芪苈强心胶囊上市后在临床应用中显示出的独特优势，也成为国内多名专家学者研究的热点。已有的研究表明该药有强心、利尿作用，可以改善 CHF 大鼠心脏功能，并且通过减少肾脏 AQP2 的表达，增加水的排出[17]。能够减少心梗后心衰大鼠的 Ang II、periostin 蛋白的表达，并具有剂量依赖性[18]。芪苈强心胶囊有效改善心功能的作用机制与其抑制心肌重构有关[19]。减少心肌细胞促炎因子和增加抗炎因子的免疫调节作用可能是中药芪苈强心改善 AMI 大鼠心功能的免疫药理机制之一[20]。在观察芪苈强心胶囊治疗对慢性充血性心力衰竭病人疗效的临床研究中，表明芪苈强心胶囊可以改善心衰患者心功能分级、Lee 氏心力衰竭计分、心脏收缩和舒张功能、射血分数(EF)、中医证候和生活质量等疗效指标及安全性指标，升高血一氧化氮(NO)、降钙素基因相关肽（CGRP）水平，降低其内皮素(ET)水平，从而明显改善心力衰竭患者的内皮功能。

综上所述，芪苈强心胶囊全方标本兼治，从多途径、多环节、多靶点治疗心功能不全，体现了复方中药在治疗心力衰竭方面从整体论治的优势，明显降低 NT-proBNP 水平，这意味着患者的长期预后可能有所改善。然而尚需要展开一项以复合终点事件发生率为终点的大型临床随机对照研究以证实该假设。

4. 试验总体设计与安排

本研究是一项在慢性心衰患者中进行的随机、双盲、安慰剂对照、平行分组的多中心临床研究。

本研究将为事件驱动型，全部随机入组的患者将保留在研究之中（无论是否服用研究药物），直至主要终点事件的发生数目达到预计（620 例），或者当满足事先定义的提前终止的疗效或安全性标准时，研究提前终止。

计划在发生 1/2、2/3 主要终点事件后进行两次期中疗效分析，以评估是否已得出无效或有效的结论而提前终止该研究。

计划整个研究将持续大约 36 个月，招募期预计 24 个月，最后一例患者入组研究后的随访期为 12 个月。预计平均随访时间约为 24 个月。

在医院开始筛选患者，临床症状稳定，入选之前已接受至少 2 周标准化方案治疗并治疗其他伴随疾病。根据当地 HF 治疗指南规定用药，药物种类、剂量固定，除非禁忌或不耐受，且此期间未静脉用药，未服用与芪苈强心胶囊成分相似中药、中成药的患者直接进入随机分组阶段。

若达不到上述要求，则可先行标准化治疗达到上述标准后再进入随机分组阶段。

4.1. 随机分组阶段（第 0 天~第 24 个月）：

*请注意：*接受 2 周以上的标准化治疗方案且未使用中药治疗符合入选标准的受试者进入随机分组阶段。此期间的每一位患者使用药物种类、剂量需要固定，不能有变化，不能再调整。若属医疗需要调整用药，需记录在病例报告表中。

患者将按照 1:1 的比例随机化到试验组或对照组。患者将在当前慢性心衰标准化治疗的基础上使用研究药物。

试验组：慢性心衰标准化治疗+芪苈强心胶囊（4 粒/次，3 次/日，口服）；

对照组：慢性心衰标准化治疗+芪苈强心胶囊安慰剂（4 粒/次，3 次/日，口服）

治疗期间应避免使用其他中药或中成药（与芪苈强心功能组成相似的中药）

患者应于随机分组后第 1 个月、第 3 个月、第 6 个月，第 9 个月，第 12 个月，此后每隔 3 个月来医院访视，进行有效性和安全性评估，直到研究结束。随机分组阶段共 24 个月。

4.2. 病例数量、分组、中心

参考 PARADIGM-HF 研究，中位随访 27 个月 LCZ696 组患者的心血管死亡或心衰住院

率为 21.8%，而依那普利组为 26.5%。所以我们估算基础治疗+安慰剂组随访 36 个月内，所有患者的心血管死亡和心衰住院事件发生率为 25%，基础治疗+芪苈强心组发生率为 20%。

试验组与对照组的随机分配比例为 1:1，考虑到期中分析对 I 类错误的消耗，α 调整为单侧 0.02314。样本量为复合终点事件的发生例数。预计需要观察到 620 例复合终点事件，才能提供 80%的把握度（β=0.2），经过 log-rank 检验得到试验组可以降低 20%风险的结论。

假设随访期 36 个月内对照组复合终点事件的发生率为 25%，整个试验持续大约 36 个月，招募期预计 24 个月，则预计需要入组 3080 例（每组 1540 例）受试者才可获得 620 个终点事件。

因此本研究计划纳入 3080 例患者，患者将以患者将以 1:1 的比例分配至试验组与对照组，并计划在约 100 个中心进行。

5. 研究人群

入组患者必须满足下文所列的所有入选标准，并且不符合任何一项排除标准。除下文所列标准外，接受标准化治疗期间，如果存在任何禁忌的医学状况或使用禁忌药物，也是排除患者入选的标准。

5.1. 入选标准

1) 自愿参加，理解并签署知情同意书；
2) 年龄≥18 岁，性别不限；
3) 有 3 个月以上的慢性心衰病史或临床发现心衰症状 3 个月以上；慢性心衰诊断参照当地指南或中华医学会心血管病学分会发布的“中国心力衰竭诊断和治疗指南 2014”；
4) 心脏彩超检查提示左室射血分数（LVEF）≤40％（改良辛普森法）；
5) NYHA 心功能分级Ⅱ～Ⅲ，临床症状稳定，包括入选前 2 周内曾诊断为Ⅳ级者；
6) 血清 NT-proBNP 含量≥ 450pg/ml；
7) 至少已接受 2 周以上的标准化药物治疗，且未调整过给药剂量及给予静脉治疗者；标准化药物治疗包括：血管紧张素转换酶抑制剂（ACEI）或血管紧张素受体拮抗剂（ARB）或血管紧张素受体脑啡肽酶抑制剂(ARNI)、β 受体阻滞剂及醛固酮受体拮抗剂（除非禁忌或不耐受，应达到最佳治疗剂量）

5.2. 排除标准

1) 不符合入选标准；
2) 由于瓣膜病、先天性心脏病、心包疾病、心律失常及非心源性病因所致心衰，或肝、肾等重要脏器功能衰竭导致的心衰；及有明确肺源性或其他原因所致的右心衰、及急性心衰；
3) 计划于近期内行冠脉血运重建治疗者或心脏再同步化治疗者，已实施心脏再同步化治疗者；
4) 合并肝、肾、造血系统等严重原发性疾病，肾功能异常者，肝脏转氨酶、碱性磷酸酶超出 3 倍正常值上限，血肌酐＞2mg/dl(176.82umol/L)，血钾＞5.5mmol/L；肿瘤患者，严重神经内分泌系统疾病及精神病患者；
5) 存在左室流出道梗阻、心肌炎、大动脉瘤、夹层动脉瘤、致明显血液动力学改变的未修补的心脏瓣膜病患者；
6) 存在心源性休克、难以控制的恶性心律失常、二度Ⅱ型以上未置入起搏器治疗的窦房或房室传导阻滞、进行性加重的不稳定心绞痛或急性心肌梗死者；
7) 未获控制的高血压患者，收缩压≥180/mmHg 和/或舒张压≥110mmHg；收缩压<90mmHg 和/或舒张压<60mmHg；
8) 1 个月内参加其他药物临床研究者；
9) 妊娠或正准备妊娠及哺乳期妇女；
10) 过敏体质者，或已知对治疗药物过敏者；
11) 根据研究者判断，患者不能完成本研究或不能遵守本研究的要求（由于管理方面的原因或其它原因）。

5.3. 中止研究药物治疗

随机分组后，任何原因暂停研究药物不等于永久停用，也不应该导致患者退出整个研究。相反对于已经停止服用研究药物的患者，也应该参加所有方案规定的研究访视和评价项目。如果患者不能参加研究访视，应按照计划通过电话继续随访，以确定是否发生任何不良事件和终点事件，除非患者拒绝随访并撤回知情同意书。

出现以下情况时可中止研究药物治疗：

1. 患者可在任何时间中止治疗
2. 发生与研究药物明确相关的过敏反应

3. 发生与研究药物明确相关的不良症状或体征、异常检查结果，研究者判断须终止研究的情况
4. 女性于研究期间发生妊娠

试验过程中应尽可能使患者长期服用标准剂量研究药物，中止研究药物患者在排除相关原因后应尽早恢复服用研究药物并按计划进行随访。

5.4. 退出标准

所有填写了知情同意书并筛选合格进入试验的受试者，无论何时何因退出，均不会影响其后续治疗。

患者有权在任何时间以任何理由退出研究。仅在患者拒绝任何进一步评估或者联系时，才撤回知情同意书并退出。但应当尽量避免不必要的患者退出，并积极采取措施，尽可能完成随访，以备对其疗效和安全性进行分析。但当患者决定退出时，研究者应当通过电话或个人访问形式联系患者或其责任亲属并尽可能确认退出原因，研究者应当在患者退出时回收剩余药物，完成最终评估，尽可能完成病例报告、解释退出原因，对退出患者发生终点事件进行随访。如果患者退出的原因为不良事件，则应将主要事件记录于 CRF 内。

5.5. 全面中止试验标准

1) 研究进行中由于以下原因整个试验在多中心全面停止：
   - 基于 DSMB 中期分析结果建议；
   - 研究者发现严重安全性问题；
   - 方案有重大失误；
   - 资助方因经费或管理原因；
   - 行政主管部门撤消试验，均可中途停止全部试验。
2) 全面中止试验可是暂时的，也可是永久的。中止试验时，全部试验记录应予保留备查。

6. 治疗

6.1. 试验用药物

6.1.1. 药物来源

试验药品名称：芪苈强心胶囊 Qiliqiangxin Jiaonang

成份：黄芪、人参、附子、丹参、葶苈子、泽泻、玉竹、桂枝、红花、香加皮、陈皮。

性状：胶囊剂，内容物为棕褐色至黑褐色的颗粒，味苦。

规格：0.3g/粒

批号：国药准字 Z20040141

生产单位：石家庄以岭药业股份有限公司

安慰剂：芪苈强心胶囊模拟剂（模拟剂与试验药品在颜色、规格、包装、标签、内容物形状等方面完全一致）

上述所有试验药物、模拟剂均由石家庄以岭药业股份有限公司免费提供，并出具合格药检报告。

6.1.2.　制剂、包装与标签

芪苈强心胶囊将以 0.3g 胶囊制剂形式提供，安慰剂外观上与其完全一致。

药物包装

小包装：外观如下，印有“临床试验用药”字样，每小盒内以铝塑板装 36 粒药。

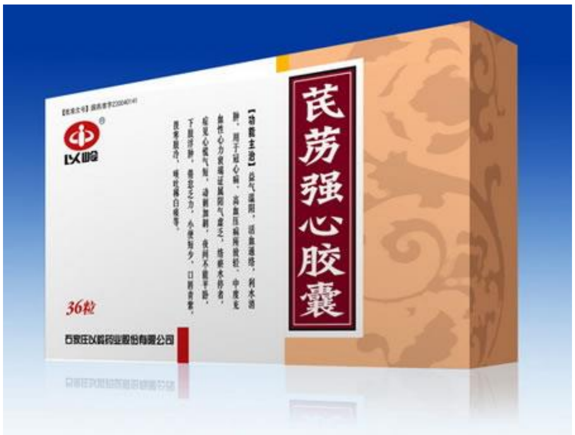

大包装：为 29.5cm×12cm×22cm 的白板纸盒，每一个大包装内含 33 个小包装，每个大盒贴有如下标签：

芪苈强心胶囊对慢性心衰复合终点事件的评估研究用药

（仅供临床研究使用）

药物包装号：XXXXX

【产品批号】xxxx　　　【有效期至】xxxx

【国药准字】Z20040141

【包　　装】每小盒装 36 粒，内装 33 小盒（一名受试者 99 天用量）

【用法用量】每日 3 次，每次 4 粒

【贮　　存】密封，在干燥处、儿童不易触及处保存

6.1.3. 保存方法

试验药物应当在安全可控的室内区域内上锁保存，注意防潮。每个试验中心必须指定研究用药管理负责人保存、管理。

6.1.4. 药物的发放与回收

每次随访研究者将药物发给受试者时，需填写药品发放登记表，及时、准确记录发放的药品数量并提醒受试者下次就诊时将其剩余药物带回；回收并核对返还药品的数量，记录于药品发放/返还登记表中。试验结束时，全部剩余药品应返还给申办者，并填写研究用药回收表格，未发放的药品在返还时必须是密封的。试验结束后剩余药物由监查员回收，统一处理。

6.1.5. 药物清点

自服药后受试者应按照访视计划归还所有剩余药品，研究者清点剩余药品数量并记录，用以判断依从性。

6.2. 治疗方案

6.2.1. 研究流程：

入组期（第-14 天~第 0 天）：

患者如果符合本方案中标准治疗的规定，可在此期间完成所有检查，符合入选、排除标准的患者进入随机分组阶段。

随机治疗期（第 0 天~12 个月（最长 36 个月））：（以发药时间记为 0 天）

试验组：慢性心衰标准化治疗+芪苈强心胶囊（4 粒/次 3 次/日 口服）

对照组：慢性心衰标准化治疗+芪苈强心胶囊模拟剂（4 粒/次 3 次/日 口服）

研究药物推荐于每日三餐后约 30 分钟时服用。访视日早晨在家勿服用药物。如果患者某日未服药，其次日服药剂量不得超过日剂量。研究不允许进行剂量调整。如果患者出现无法耐受的不良事件，并且根据研究者考虑与研究药物相关，则患者应当终止研究药物治疗。

6.2.2. 基础治疗

参照当地 HF 治疗指南或中华医学会心血管病学分会发布的“中国心力衰竭诊断和治疗指南 2014”规定如下：

1) 入选前至少 2 周以上未静脉使用利尿剂、强心剂及血管扩张剂。
2) 进入随机分组阶段至少 2 周前，患者应当接受慢性心衰标准化治疗，所有药物已经调整至固定剂量，标准化药物治疗包括：血管紧张素转换酶抑制剂（ACEI）或血管紧张素受体拮抗剂（ARB）或血管紧张素受体脑啡肽酶抑制剂(ARNI)、β受体阻滞剂及醛固酮受体拮抗剂（除非禁忌或不耐受，应达到最佳治疗剂量）；
3) 进入治疗期后每一位患者使用药物种类、剂量需延续入选前标准化治疗方案。整个治疗期间原则上不能再调整；若属医疗需要调整用药，需记录在病例报告表中，增加或减少药物剂量、种类的患者需记录复合终点事件和不良事件。

6.2.3. 合并用药

1) 接受较好地控制高血压、心绞痛、糖尿病或其他疾病的药物治疗。
2) 患者进入随机分组阶段之后整个治疗期均不得使用研究药物以外的其他与试验药物成分类似中药。
3) 患者应当接受有利于心脏健康的饮食指导，如低盐饮食，适量饮水等，患者同时应当接受诸如监测体重、体育锻炼、戒烟、戒酒等适当生活方式改善的咨询。
4) 目前尚未发现禁止与芪苈强心胶囊伴随使用的药物。

6.2.4. 疗程：12 个月-36 个月。

6.3. 依从性评价

通过完整记录药物的分发和回收情况来对受试者进行依从性评价，实际服用药物量在应用药物量的 80%~120%范围内，可判定为用药依从性符合方案要求。

6.4. 药物不良反应

目前，尚未发现芪苈强心胶囊存在明显的不良反应。

7. 临床观察终点和指标

7.1. 临床观察指标

7.1.1. 主要有效性终点

- 心血管死亡和心衰恶化再住院组成的复合终点事件发生率；

7.1.2. 次要有效性终点

- 全因死亡率
- 复合终点事件（心衰恶化放弃治疗、心脏骤停后复苏成功、恶性心律失常、非

致死性卒中）

- 冠心病心衰患者的心血管死亡和心衰恶化再住院发生率
- 血清 NT-proBNP 下降率

注：全部终点事件需经事件判定委员会复核判定。

7.2. 安全性指标包括：

- 不良事件评价
- 临床实验室指标：血常规、尿常规、血清生化。
- 12 导联心电图
- 体格检查

8.　试验过程

所有患者包括研究完成前停用研究药物，都应继续参加表格所列的计划访视，直至研究结束。如果某次访视被推迟或者提前，不应影响下次访视。下次访视应遵守原计划时间进行。

试验流程表

访视	1	2	3	4	5	6	7	8	9	10	11	12	13	14	UNS	EOS
天/月	0天	1M	3M	6M	9M	12M	15M	18M	21M	24M	27M	30M	33M	36M		
	-14	±3	±7	±7	±7	±7	±7	±7	±7	±7	±7	±7	±7	±7		≧2周
知情同意书	×															
入选/排除标准	×															
中央随机	×															
一般资料/病史	×															
心衰病史	×															
心血管病史	×															
体格检查	×	×[1]	×[1]	×[1]	×[1]	×	×[1]	×[1]	×[1]	×	×[1]	×[1]	×[1]	×	(×)	×
心衰用药	×	×	×	×	×	×	×	×	×	×	×	×	×	×	(×)	×
CV用药	×	×	×	×	×	×	×	×	×	×	×	×	×	×	(×)	×
其他用药	×	×	×	×	×	×	×	×	×	×	×	×	×	×	(×)	×
心脏彩超	×*															
妊娠试验	×					×				×				×	(×)	×
血/尿常规	×	×				×				×				×	(×)	×
生化检查	×	×				×				×				×	(×)	×
12导联心电图	×*	×				×				×				×	(×)	×
当地实验室血清NT-proBNP	×															
中心实验室血清NT-proBNP	×	×		×												
分发药物	×		×	×	×	×	×	×	×	×	×	×	×	×		
回收药物	×		×	×	×	×	×	×	×	×	×	×	×	×		×
终点事件		×	×	×	×	×	×	×	×	×	×	×	×	×	(×)	×
不良事件		×	×	×	×	×	×	×	×	×	×	×	×	×	(×)	×

- ×[1]为简化体格检查
- ×*心脏彩超和 12 导联心电图接受入组前 6 个月内结果。
- UNS(计划外访视中):（×）标记项目为可选择操作，根据研究者判断进行。
- EOS（终末访视）:根据研究结束时间安排进行（如在试验结束前一个月内有过访视则视为终末访视但需补充完整终末访视所需项目）。
- 妊娠试验仅适用于育龄妇女（如尿妊娠试验阳性则必须进行血清妊娠试验证实）。

9. 疗效与安全性判定标准

9.1. 疗效判定标准

9.1.1. 主要有效性评价

- 心血管死亡和心衰恶化再住院组成的复合终点事件发生率；

9.1.2. 次要有效性评价：

- 全因死亡率
- 复合终点事件（心衰恶化放弃治疗、心脏骤停后复苏成功、恶性心律失常、非致死性卒中）
- 冠心病心衰患者的心血管死亡和心衰恶化再住院事件发生率
- 血清 NT-proBNP 下降率

9.1.2.1. 终点事件

- **心力衰竭住院是指满足下列所有标准的事件：**

1.患者因初步诊断为 HF 住院

2.患者住院时间延长至少 24 小时（或者如果不能获得住院时间和出院时间，指在日历上日期发生的变化）

3.在患者报告上记录由于 HF 出现新症状或者恶化症状，至少包括以下情况之一：

a.呼吸困难（用力时呼吸困难、休息时呼吸困难、端坐呼吸、夜间阵发性呼吸困难）

b.运动耐量减少

c.疲劳

4.患者具有新出现的恶化 HF 的客观证据，包括至少两种体检结果或一种体检结果和至少一种实验室标准，包括：

a.判断由 HF 导致的体检结果，包括新出现或恶化的：

1)外周性水肿。 2)腹胀或腹水增加（在无原发性肝病的情况下）。 3)肺啰音/爆裂音/湿性啰音。 4)颈静脉压升高和/或肝颈静脉回流。 5)S3 奔马律。 6)具有临床意义的或迅速的体重增加，考虑与体液潴留有关

b.在 24 小时内获得的，新出现或者恶化 HF 的实验室证据，包括：

1)与 HF 失代偿一致（如 BNP>500pg/ml 或 NT-proBNP>2000pg/ml）的 B 型利钠肽（BNP）/N 末端 B 型利钠肽前体（NT-proBNP）浓度增加。在利钠肽长期升高的患者中，应特别关注超过基线的显著增加。 2)肺充血的放射影像学

证据。3)具有临床意义的左侧或者右侧心室充盈压升高或者心输出量降低的非侵害性诊断证据。如超声心动图标准包括：E/e´>15 或者 D 主导肺静脉流入模式，充血性下腔静脉伴有极小程度吸气塌陷，或者左心室流出量（LVOT）微小行程距离减小（时间流速积分（TVI））。4)侵入性诊断证据：右心导管检查显示肺毛细血管楔压（肺动脉闭塞压）≧18mmHg，中央静脉压≧12mmHg，或心排血量指数

注：如适用，即使不满足上述标准，仍需报告诊断检查中的所有结果，为上述事件的裁定提供重要信息。

5.患者接受针对 HF 的初期或者强化治疗，包括下列至少一种：

a.增强口服利尿药的治疗

b.静脉注射利尿药或者血管活性药物（如正性肌力药、血管升压类药物或者血管扩张剂）

c.机械或手术干预，包括：

1)机械循环支持（例如，主动脉球囊反搏、心室辅助装置、体外膜氧合、全人工心脏）

2)机械辅助去除体液（例如，超滤、血液滤过、透析）

- **心血管死亡：**包括急性心肌梗死（MI），心源性猝死，心力衰竭（HF）导致的死亡，中风导致的死亡，心血管（CV）手术导致的死亡，CV 出血以及其他 CV 原因造成的死亡。
- **全因死亡**
- **心衰恶化放弃治疗：**心衰症状和体征不断加重，需要静脉药物或机械支持治疗而患者或患者家属主动放弃治疗或自动出院，若随访其后果为死亡则列入心力衰竭死亡。
- **心脏骤停后复苏成功**
- **恶性心律失常：**对于恶性心律失常的定义， 目前还没有统一的标准，一般是指能在短时间内引起严重血流动力学障碍，导致患者晕厥甚至猝死的心律失常 。根据这个标准，恶性心律失常主要有如下类别：(1) 严重的缓慢型心律失常， 如严重的病态窦房结综合征、高度或三度房室传导阻滞；(2) 快速型心律失常， 如持续性室性心动过速、心室扑动、心室颤动， 快室率心房扑动、心房颤动、房室折返性心动过速、预激综合征伴心房颤动、窦性心动过速等。

- **非致死性卒中**

  注：终点事件发生时的评估与程序

研究者获知终点事件发生后，应在 7 天内收集相关支持文件报告事件判定委员会。终点事件将由独立的事件判定委员会（CEC）进行复核，因此终点事件报告表将作为 CRF 的一部分，研究者将在上述表格内记录事件并及时提交支持文件（入院与出院记录、病历记录、死亡记录、ECG 等）上述资料将提供给 CEC 以对事件进行判定。

CEC 由主席及 5-6 名成员组成，每一例事件将由委员会的两位成员进行独立审查并将结论提交至委员会主席处。如果两位审查委员之间或两位审查委员与主席的意见不一致，当具有异议的事件积累到一定数量时，整个委员会将安排会议对事件进行审查。

9.2. 安全性判定：

- 不良事件评价
- 临床实验室指标：

  在空腹状态下（至少禁食 10 小时：允许患者饮水但不允许饮用咖啡或茶）采集血样及尿样送往各个中心的实验室，按照统一标准进行检测

  血常规、尿常规、血清生化
- 12 导联心电图
- 体格检查：

  全面体格检查：一般情况（包括身高、体重）、生命体征（包括血压、脉率）、皮肤（包括头发与指甲）、眼耳鼻喉、颈部/甲状腺、胸部/肺、心血管系统，腹部/胃肠道系统，生殖-泌尿系统（选择性）、神经系统、淋巴与骨骼肌肉；

  简化体格检查：一般情况（包括体重）、生命体征（包括血压、脉率）、胸部/肺、心血管系统

10. 不良事件的观察

10.1. 定义：

- 不良事件（**AEs**）：自受试者签署知情同意书并入选试验后开始至最后一次随访之间，发生任何不利医疗事件，无论与试验药物是否有因果关系，均判定为不良事件。
- 重要不良事件：除严重不良事件外，发生的任何导致针对性医疗措施（如停药，

降低剂量和对症治疗）的不良事件和血液学和/或其他实验室异常。

10.2. 不良事件强度判定标准：

在本临床研究中发生的所有临床不良事件将记录在 CRF 不良事件页上。并将不良事件强度进行分级。为统一标准，事件强度分级如下：

轻度　　可察觉的不适感但是不影响日常活动

中度　　不适感较强以致影响或减少日常活动

重度　　无法工作或进行日常活动

注意区别不良事件的严重程度和强度。重度用来描述强度，不一定是严重不良事件（SAE）。例如头痛可能在强度上表现为重度，但不能列入严重不良事件，除非它符合 SAE 标准。

10.3. 不良事件与研究药物关系的判断标准

对所有不良事件与试验药物关系的因果分析，均按肯定有关、很可能有关、可能有关、可能无关、肯定无关五级进行判断，对前三种定为药物的不良反应。因果分析的考虑因素有以下五个方面：

1）开始用药时间和可疑药物不良反应（Adverse Drug Reaction，ADR）出现的时间有无合理的先后关系（用药出现）

2）所怀疑的 ADR 是否符合该药已知的 ADR（符合文献）。

3）所怀疑的 ADR 能否用合并用药、曾用药、病人的临床情况，或其他疗法的影响来解释（其他解释）。

4）停药或减量后可疑的 ADR 是否消失或减轻（停药反应）。

5）在此接触同样的药物后，可疑的 ADR 是否再次出现（再用再现）。

研究者应对不良事件和试验药物以及合并药之间可能存在的关联作出评估，参照下表

考虑因素	用药出现	符合文献	其它解释	停药消失	再用再现
肯定有关	＋	＋	－	＋	＋
很可能有关	＋	＋	－	＋	?
可能有关	＋	＋	±	±	?
可能无关	＋	－	±	±	?
肯定无关	－	－	＋	－	－

10.4. 严重不良事件的判定

10.4.1. 一般严重不良事件定义

严重不良事件是指任何提示显著危害、禁忌症、副作用或者需谨慎的临床事件。不良事件符合下面一条或以上标准时归为严重不良事件：

- 死亡
- 有生命危险（指出现该事件的患者在事件发生当时存在立即死亡的风险；并不包括那些如果更加严重将有可能导致患者死亡的事件）
- 导致住院或住院时间延长
- 导致持久或显著的劳动力丧失或残疾
- 先天性畸形缺陷

有些还没有导致死亡、生命危险或需住院的医疗事件，经过适当的医学判断，认为其可能对病人或受试者造成危害或需药物或外科手术治疗以避免上述情况发生时，也应视为 SAE。

10.4.2. 严重不良事件的研究特异性定义

在本试验中，下列事件将不会作为严重不良事件报告，除非被判定阴性并且研究者认为与研究用药相关

- ◆ 心血管死亡
- ◆ 心衰恶化再住院
- ◆ 心衰恶化放弃治疗
- ◆ 心脏骤停后复苏成功
- ◆ 恶性心律失常
- ◆ 非致死性卒中

但是，所有其他导致致死性结局的事件都将被作为严重不良事件报告。

10.5. 不良事件的随访与记录

出现的不良事件，尤其是那些与试验药物相关的事件应当随访直至它们恢复至基线状态或者趋于稳定。如果经过随访，仍无法恢复基线状态或者稳定，那么应当在 CRF 中记录说明。临床试验过程中的任何严重不良事件，必须在 24 小时内报告临床试验监查人员、主要研究单位、药品生产企业。同时研究者必须填写严重不良反应表，记录严重不良事件的发生时间、严重程度、持续时间、采取的措施和转归。

10.6. 实验室结果异常

研究者应当对实验室结果异常是否具有临床意义进行判断，并给出可能的解释。已经被报告的不良事件导致的实验室异常结果应同时作为不良事件记录在不良事件表中。具有临床意义的实验室检查异常满足以下一项或多项条件者，应作为独立诊断记录在 CRF 的不良事件页中（不包括已被报告的不良事件导致的实验室结果异常）：

- 伴有临床症状的
- 导致研究用药改变的
- 需要改变合并用药和（或）其他治疗措施的

11. 盲法与随机化

11.1. 受试者随机分配方法

由北京大学临床研究所统计专业人员，在计算机上用 SAS9.4 统计软件包，按试验组与对照组 1:1 的比例用区组随机化方法生成随机编号。根据此随机编号由与本试验无关的人员对研究药物（芪苈强心胶囊或安慰剂）进行包装编码。

本研究采用随机化与试验药物管理系统（RTSM），统计专业人员将向RTSM提供随机编号列表。然后，由RTSM给患者分配随机编号。

在完成基线评估后，在基线访视通过RTSM分配随机编号。此后，按照访视计划通过RTSM获取药物编号，每次分配的药物编号均不相同，但药物是同一种。在患者随机分组前，研究者必须先登录RTSM，并且提供一些信息（例如受试者出生日期、性别等）。

11.2. 紧急揭盲

如果发生不良事件，只有在必须了解研究药物的使用才能治疗患者的特殊情况下，才能进行紧急揭盲。一旦决定揭盲，研究者必须记录日期、时间和破盲的原因。

研究者需要登录 RTSM 填写破盲申请，由主要研究者审核后再由破盲员破盲。一旦破盲，该病例将中止研究，作为退出处理。

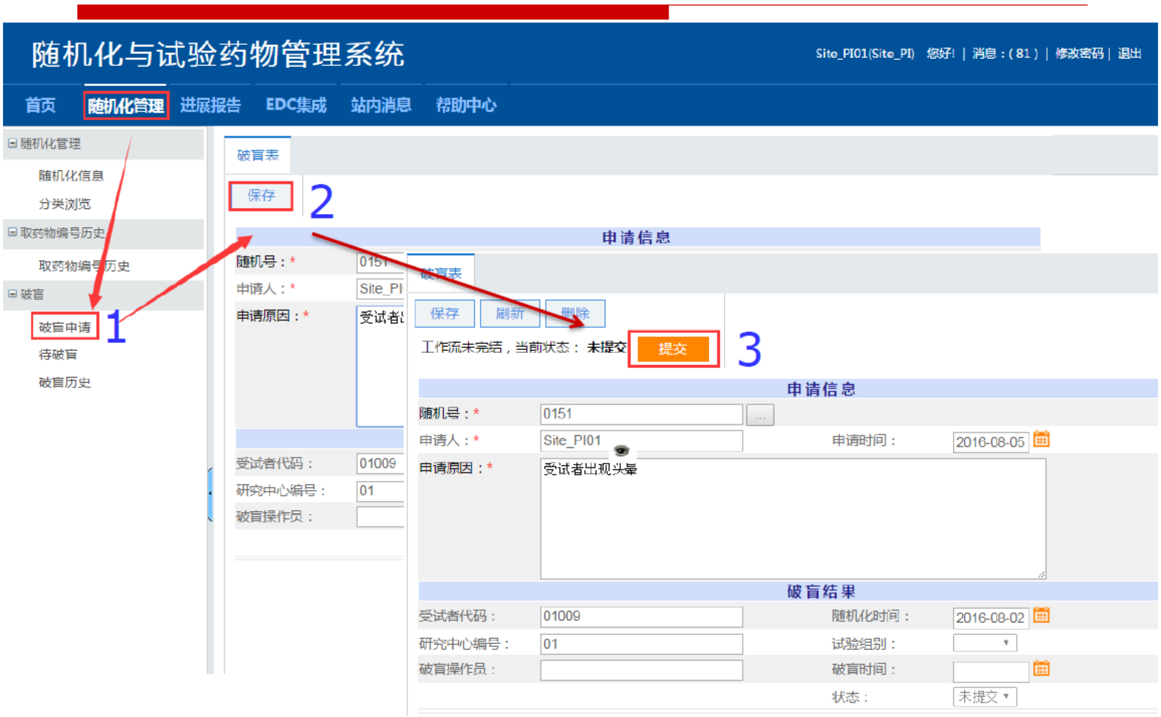

11.3. 揭盲规定

本研究采用二次揭盲法。在经盲态核查后，数据锁定，由主要研究者、医学统计专家、数据管理员、申办单位代表进行第一次揭盲，将各随机号所对应的组别以 A，B 为代号标出，以便对全部数据进行统计分析。当统计分析结束，统计报告完成时，再进行第二次揭盲，宣布 A，B 两组的确切组别。

11.4. 筛选编号

各医院按收治患者的先后顺序编排筛选号，筛选号由中心编号+3 位整数表示，如，01 中心筛选号 01001，01002，……。

12. 统计分析

试验方案确定后，由统计专业人员负责与主要研究者协商制订统计分析计划书。统计分析软件采用 SAS®9.4 软件（或更高版本）。样本量计算软件采用 PASS13。

12.1. 分析人群

研究人群分为以下几类：

- 全分析集(FAS)：是指尽可能接近意向性分析原则（intention to treat）、从所有随机化的受试者中，以最少的和合理的方法剔除受试者后得出的数据集，包含所有经过随机化并使用过一次研究药物的受试者。剔除通常包括：违反重要入组标准；

受试者未接受试验用药物的治疗；随机化后无任何观测数据。主要疗效评价指标为发生复合终点事件的时间，采用生存分析的方法进行分析，在选择 FAS 进行统计分析时，对于主要终点事件的缺失按照删失处理。

- 符合方案集(PPS)：是全分析集的一个子集，这些受试者对方案更具依从性。纳入 PPS 受试者一般具有以下特征：（1）完成事先设定的试验药物的最小暴露量，即服用药物的依从性达到 80%；（2）试验中主要指标的数据均可以获得；（3）未对试验方案有重大的违背。
- 安全性分析集（SS）：所有随机化后至少接受一次治疗且有安全性评价的受试者。安全性缺失值无需结转。

疗效分析将在 FAS 和 PPS 的基础上进行。所有基线人口统计学资料分析将在 FAS 的基础上进行，安全性评价将在 SS 上进行。

12.2. 统计分析方法

- 所有的统计检验均采用双侧检验，*P* 值小于或等于 0.05 将被认为所检验的差别有统计意义。（特别说明的除外）
- 描述性分析：分类指标描述各类的例数及百分数。定量指标采用均数、标准差、最大值、最小值、中位数、下四分位数（Q1）和上四分位数（Q3）描述。
- 对两组一般情况的比较将根据指标的类型采用适当的方法进行分析，定量资料的组间比较采用成组 t 检验或 Wilcoxon 秩和检验，分类数据采用卡方检验或精确概率法，等级资料采用 Wilcoxon 秩和检验或 CMH 检验。

12.2.1. 入组及完成情况：

总结各中心入组及完成数，列出脱落病例的清单。各组不同数据集大小，各中心病例分布，总脱落率比较，终止原因详细列表。对患者的人口学特征(年龄、身高、生命体征等)、病史及用药史等进行描述，并对两组年龄、身高、体重等进行比较，以衡量两组的可比性。

12.2.2. 依从性分析：

- 用药依从性分析：比较两组病人是否按时按量使用试验药物，未用方案中禁用的药物和食物。
- 合并用药分析：需统计各组合并用药人数，并详细列表。

12.2.3. 疗效评价：

- 疗效评价同时进行 PP 分析和 FAS 分析；
- 主要疗效评价指标为发生复合终点事件（心血管死亡和心衰恶化再住院）的时间。对于主要终点事件的缺失按照删失处理。

本研究的主要研究假设为：

H_0：$\lambda_T/\lambda_C \geq 1$

H_1：$\lambda_T/\lambda_C < 1$

其中，λ_T和λ_C分别为试验组和对照组发生终点事件的风险。

利用 Kaplan-Meier 法估计临床终点事件发生率，两组之间进行 Log rank 检验。

利用 COX 比例风险模型，以中心为协变量，计算两组间的风险比（Hazard Ratio）及其 95%可信区间。另外，对复合终点事件的两个部分分别进行分析，即心血管死亡和心衰恶化再住院。

- 次要疗效指标：

全因死亡率：利用 Kaplan-Meier 法估计两组全因死亡率，并进行 Log rank 检验。利用 COX 比例风险模型，以中心为协变量，估计两组间的风险比（Hazard Ratio）及其 95%可信区间。

复合终点事件（心衰恶化放弃治疗、心脏骤停后复苏成功、恶性心律失常、非致死性卒中）：分析方法与全因死亡相同。

冠心病心衰患者的心血管死亡和心衰恶化再住院事件：按计数资料统计分析；

血清 NT-proBNP：按计量资料分析，对两组血清 NT-proBNP 水平进行统计描述和组间比较，并对两组与基线的变化情况进行统计描述和组间比较。

12.2.4. 安全性评价：

安全性评价基于 SS 数据集进行分析。

不良事件用不良事件发生例次、例数及发生率进行描述，并对该发生率进行组间显著性检验。同时，列表详细描述各组病例出现的全部不良事件的具体表现、程度及其与药物的关系。

对实验室指标前后变化情况进行交叉表描述，按试验组和对照组分别描述治疗前正常、治疗后异常例数及该例数所占比例。对生命体征指标进行前后比较。

12.3. 期中分析

本研究计划在收集到 1/2 和 2/3 主要终点事件后进行两次期中疗效分析，以评估是否已得出有效的结论而提前终止该研究。根据 Lan-DeMets α消耗函数和 O’Brien-Fleming 方法，第 1 次期中分析时消耗的 I 类错误α=0.0001（单侧），第 2 次期中分析消耗α=0.00605（单侧）。

期中分析有关的具体要求和操作将在 DSMB 章程中事先规定。

13. 数据管理

本研究采用 Epidata3.1 软件进行研究数据的采集。数据管理确保临床试验数据的真实性、完整性和准确性，数据管理过程需符合《药物临床试验质量管理规范》、《临床试验数据管理工作技术指南》等法规要求，保证临床试验数据的可溯源性。以下列出数据管理的主要流程。

13.1. 数据库设计

数据管理员根据 CRF 采用 Epidata3.1 软件设计数据库，经测试后发布。

13.2. 数据录入

CRC 负责将 CRF 中的数据录入数据库，数据录入采用二次录入方式，由两名 CRC 分别录入一遍数据，数据管理员对两个数据库进行比对，产生数据不一致清单，CRC 按照清单对照 CRF 分别修改各自的数据库，然后再进行比对，重复以上步骤，直至两个数据库完全一致。

13.3. 数据质疑管理

数据管理员依据数据核查计划（DVP）编写数据核查 SAS 程序对数据进行核查，产生数据质疑清单，经人工核对后，生成数据质疑表，由 CRA 交研究者进行答疑，答疑后的质疑表再由 CRA 返还给数据管理员，数据管理员据此修订数据库。

13.4. 医学编码

不良事件编码采用 MedDRA21.0。

13.5. 数据审核

数据库清理完成后，数据管理员撰写《数据核查报告》，用于召开数据核查会议。

审核报告重点记录内容为：入组病例数、脱落、剔除病例情况、偏离或违背方案情

况、依从性数据，合并用药，不良事件，与评价指标有关的数据等。

数据审核会议上，针对审核报告的内容，讨论并确定统计人群的划分。

13.6. 数据库锁定

完成数据库锁库清单，依据数据库锁定程序完成数据库锁定。数据锁定之后发现的问题，经确认后可在统计分析程序中修正。数据锁定后如有确切证据证明有必要解锁，研究者及相关人员需签署解锁文件。

数据库锁定后，由数据管理员导出 SAS 格式的数据文件，交与统计人员进行统计分析。

14. 质量控制

1) 研究者应履行各自职责，并严格遵循临床研究方案，采用标准操作规程，对所有相关观察结果和发现都应加以核实，以保证临床研究的质量控制和质量保证系统的实施。
2) 临床研究中受试者分配必须按研究设计确定的随机分配方案进行，每名受试者的处理分组编码应作为盲底由统计单位和研究者分别保存。
3) 研究者须对参加临床研究的所有人员进行必要培训，说明有关的资料、操作规范和职责，保证将数据真实、准确、完整、及时、合法的记入病历和 CRF。CRF 必须由专人负责保管。
4) 监查员应遵循标准操作规程，督促研究方案的执行情况，确认所有数据记录与报告正确完整，所有 CRF 填写正确，并与原始资料一致。
5) 稽查人员应对临床研究相关活动和文件进行系统性检查，以评价研究是否按照方案、标准操作规程以及相关法规要求进行。
6) 临床研究中各种实验室检查数据必须准确，并应记录在案或将原始报告复印件粘贴在病例报告表上。
7) 医学统计人员应把研究数据完整、无误地纳入报告，所有涉及数据管理的各种步骤均需记录在案，以便对数据质量及实施过程进行检查。
8) 临床研究资料的统计分析过程及其结果的表达必须采用规范的统计学方法。临床研究各阶段均需有医学统计人员参与。临床研究总结报告必须与统计报告相符。
9) 各方应严格按批准方案进行临床研究，任何偏离方案的情况均需记录在案。研究方案的修改需制定修改说明，并报伦理委员会批准方可执行。

10) 各研究中心设研究负责人 1 名，固定研究组成人员若干人。严格按临床研究方案要求进行。组长单位技术人员随时与各研究中心保持密切联系，并于研究早、中、后期前往各研究中心检查病例观察记录情况，及时解决可能出现的问题。

15. 伦理相关事宜

1. 在临床研究的过程中，必须对受试者的个人权益给予充分的保障，并确保研究的科学性和可靠性。受试者的权益、安全和健康高于对科学和社会利益的考虑。
2. 研究方案需经伦理委员会审议同意并签署批准意见后方可实施。在研究进行期间，研究方案的任何修改均应经伦理委员会批准；研究中发生严重不良事件，应及时向伦理委员会报告。
3. 研究者或其指定的代表必须向受试者说明有关临床研究的详细情况，经充分和详细解释研究的内容后获得知情同意书。

16. 试验进度

2018 年 7 月	完成试验方案的制定，召开筹备会
2018 年 8 月	方案修改，通过伦理审批
2018 年 8 月	试验药物、资料准备
2018 年 9 月	试验国际注册、分中心启动
2018 年 9 月	第 1 例病例筛选入组
2020 年 9 月	完成所有病例的随机入组
2021 年 9 月	各中心完成所有病例的随访工作
2022 年 2 月	完成数据输入及盲态审核
2022 年 4 月	统计分析工作
2022 年 6 月	完成试验总结报告

17. 资料保存

研究医院应保存这些原始资料至临床试验终止后 5 年，包括对所有参加受试者的确认（能有效的核对不同的记录资料，如 CRF 和医院原始记录）、所有原始受试者知情同意书、CRF 表、药品分发的详细记录等。

18. 临床总结

统计分析结束后由试验主要研究者负责写出本研究临床总结报告并加盖主要研究单位公章。

参考文献：

1) Lopez AD. Assessing the burden of mortaIity from cardiovascuIar diS-eases[J]. WorId Health Star Q, 1993, 46(2):91-96.
2) 中华医学会心血管病学分会. 慢性心力衰竭诊断治疗指南[J]. 中华心血管病杂志, 2007, 35(12):1076-1095.
3) 陈灏珠.实用内科学[M].北京：人民卫生出版社，2001.
4) World health organization. Cardiovascular disease:prevention and control[EB/OL]. http://www.who.int/dietphysicalactivity/ publications/facts/cvd/en/. 2010-03-07.
5) Mendez GF, Cowie MR. The epidemiological features of heart failure in developing countries:a review of the literature[J]. Int J Cardiol, 2001, 80(2-3):213-219.
6) Rosamond W, Flegal K, Friday G, et al. Heart disease and stroke statistics - 2007 update - A report from the American Heart Association Statistics Committee and Stroke Statistics Subcommittee[J]. Circulation, 2007, 115(5):E69E171.
7) 顾东风, 黄广勇, 吴锡桂,等. 中国心力衰竭流行病学调查及其患病率[J]. 中华心血管病杂志, 2003, 31(1):3-6.
8) Roger VL, Weston SA, Redfield MA, et al. Trends in heart failure incidence and survival in a community-based population[J]. JAMA, 2004, 292(3):344-350.
9) Pulignano G, Del S D, Tavazzi L, et al. Clinical features and outcomes of elderly outpatients with heart failure followed up in hospital cardiology units: data from a large nationwide cardiology database (IN-CHF Registry).[J]. American Heart Journal, 2002, 143(1):45-55.
10) Kawashiro N, Kasanuki H, Ogawa H, et al. Clinical characteristics and outcome of hospitalized patients with congestive heart failure: results of the HIJC-HF registry[J]. Circulation Journal Official Journal of the Japanese Circulation Society, 2008, 72(12):2015.
11) Stewart S, Macintyre K, Hole D J, et al. More 'malignant' than cancer? Five-year survival following a first admission for heart failure.[J]. European Journal of Heart Failure, 2014, 3(3):315-322.
12) Cowie M R, Wood D A, Coats A J, et al. Survival of patients with a new diagnosis of heart failure: a population based study.[J]. Heart, 2000, 83(5):505-10.
13) Mcmurray J J, Packer M, Desai A S, et al. Angiotensin-neprilysin inhibition versus enalapril in heart failure.[J]. New England Journal of Medicine, 2014, 371(11):993-1004.
14) Swedberg K, Komajda M, Böhm M, et al. Ivabradine and outcomes in chronic heart failure (SHIFT): a randomised placebo-controlled study.[J]. Lancet, 2010, 376(9758):2069-2069.
15) Li X, Zhang J, Huang J, et al. A multicenter, randomized, double-blind, parallel-group, placebo-controlled study of the effects of qili qiangxin capsules in patients with chronic heart failure[J]. Journal of the American College of Cardiology, 2013, 62(12):1065-1072.
16) 吴以岭. 脉络论[M]. 中国科学技术出版社, 2010.
17）《芪苈强心胶囊对慢性心衰大鼠心功能与肾脏水通道蛋白 2 的影响》南方医科大学附属南方医院心内科主任许顶立 2009 西安 全国心力衰竭学术会议.
18) 李佳彧, 杨萍. 芪苈强心胶囊对心梗后心力衰竭大鼠 periostin 蛋白表达干预作用的研究[J]. 中国实验诊断学, 2009, 13(2):170-172.
19) 李娅, 宋优, 程翔,等. 芪苈强心胶囊对大鼠心肌梗死后心肌重构及心功能的影响[J]. 中国分子心脏病学杂志, 2007, 7(4):201-204.
20) 宋优, 李娅, 程翔,等. 芪苈强心调节急性心肌梗死大鼠心肌 TNF-α 和 IL-10 表达[J]. 中国免疫学杂志, 2007, 23(9):806-810.

1 附 1：NYHA 分级评分标准

NYHA 心功能分级评分标准：

Ⅰ级：体力活动不受限，日常活动不引起疲乏、心悸或呼吸困难。

Ⅱ级：体力活动轻度受限，休息时无症状，日常活动可引起疲乏、心悸或呼吸困难。

Ⅲ级：体力活动明显受限，休息时无症状低于日常活动量即出现症状。

Ⅳ级：不能进行任何体力活动，休息时即出现不适，任何体力活动都使症状加重。

2 附 2：NT-proBNP 检查采血及血样保存及运输流程

血样制备检验及冷链运送标准操作规程

1．目的

规范血样保存、运输、接收条件与过程

2．适用范围

随机、双盲、安慰剂平行对照评价芪苈强心胶囊治疗慢性心衰有效性与安全性的多中心临床试验血样制备检验及运送

3．简单流程

血样品制备 ———
↓
保存 负责方（研究者）
↓
交接 ———
↓
运输 负责方（快递公司）
↓
接收 ———
↓
化验 ———

4．制备与保存

各中心血液应在采集 2 小时之内按照血液标本制备与保存标准操作规程制备血样标本，血样品分别置于冷冻管中，按照药物的编号，将不干胶标签的其中两联贴于冷冻管上，另一联粘贴于留样记录表（表 1）上，并按要求填写留样记录表。

NT-proBNP 标本制备流程

每人抽取静脉血 3ml
↓
EDTA 抗凝

↓

离心后吸取上层血浆

↓

标记好患者标签

↓

放入-20℃冰箱保存

↓

所有标本应在采集完成后 3 个月内送检

注意：

1） 应在无炎症或感染条件下（代谢稳定）进行测定，以减少个体差异；

2） 病人准备：空腹 12 小时以上，在上午 8：00～9：00 取坐位采集静脉血。

3） 采血要求：使用含分离胶真空采血管（一般是黄帽）采集静脉血液 6ml，避免有溶血和脂血现象（若有请注明）。

4） 血清分离：标本采集后 30 分钟后，2 小时内 3000 离心 5 分钟，取分离后的上层液（血清），使用无任何添加物的采血管（一般是红帽），每份标本分成二管，每管不少于 800µl，一管送中心实验室检测，一管作为留样，上贴与登记表相同的编号（表 1），包括姓名、性别、年龄、标本采集时间和采集单位。

5） 血清标本的运送和保存：血清冷冻标本管应置于-20℃低温冰箱中，保存期最长为 6 个月，血清标本在运送时应置于干冰盒中。每日应记录冰箱温度，备查。

表 1 留样记录表及标签样稿

随机编号	患者姓名	性别	年龄	采血日期
医院（芪苈强心试验 NT-proBNP 检测）				

5．运输

研究者入组完成本中心任务数后（如果入组时间过长，可在第一例患者入选 2 个月内），拨打 XXX 快递公司统一免费下单电话：<u>400-0000-0000</u>；报出取件帐号：<u>0000000</u>，并告知具体的取件地址、联系人、联系电话、送达地址等。

请于快递公司约定的当天清点、准备好样品，快递工作人员到达医院后包装、交接样品；

研究者填写**<u>血样标本交接单</u>**并把此次运送样品的**留样记录表复印件**附上交快

递寄出，（原件由监查员下次监查时交还研究者），以备核查用。标本、留样记录表、交接单的数字应相吻合。

快递公司应于 24 小时之内送达中心实验室。

6. 接收

中心实验室人员于样品送达当日进行血样核对、编码及入库；并及时填写样品交接记录。

接收时立即开箱检查，记录样品的状态。

7. 检测

中心实验室应在接收样本后 10 个工作日内完成检测工作;并同时将结果反馈给组长单位及监查员。监查员在收到检验结果一个星期内应将结果反馈给相应研究中心。

联系人:以岭药业医学部 韩硕龙 电话:13582167153 邮箱 hanshuolong@126.com

8. 不同单位之间的换算

1pg/ml=0.118pmol/L　　　pg（皮克） pmol（皮摩尔）

3　附 2：终点事件报告流程

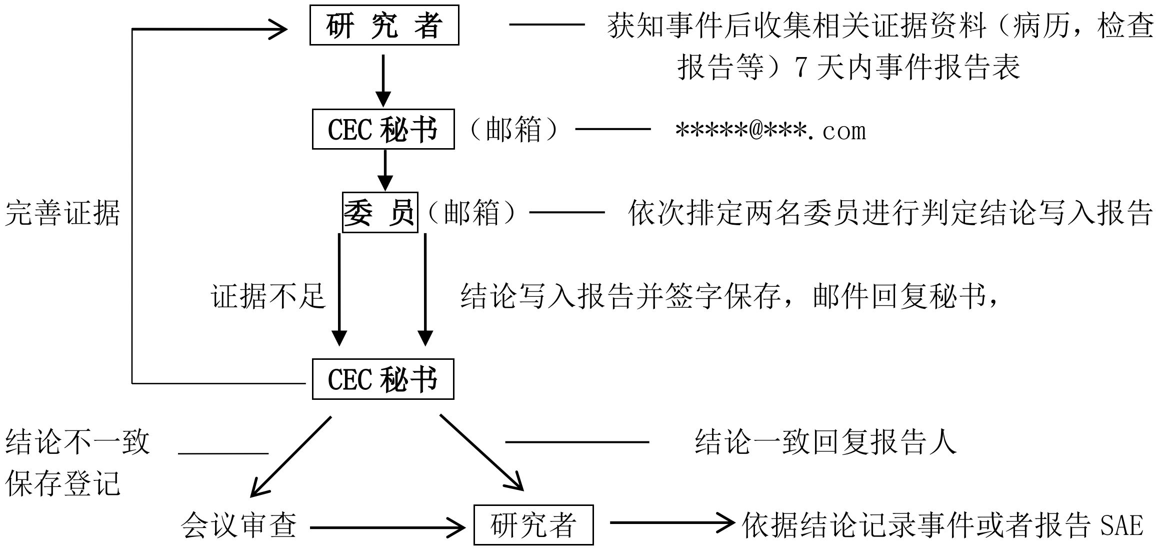

English translation version of SAP version 1.1

1. Corresponding to the amended SAP version V1.1 (in Chinese), translation date 15-Jan-2023.
2. Table of contents and Title number correction.
3. Table 9.2.1.: Baseline of eGFR was added.
4. Table 9.4.4-5: Baseline of HF treatment was added.
5. Table 9.5.1-3: Primary analysis based on the Type I error (95.372% CI) was added.
6. Table 9.6.73-123: Subgroup analysis of interest were complemented.
7. Syntax and typo corrections.
8. Contents are on the basis of and translated from Chinese. If the English and Chinese contents are inconsistent, Chinese version shall prevail.

Qiliqiangxin in Heart FailUre:
AssESsment of Reduction in MorTality
(QUEST)

Statistical Analysis Plan V1.1

Research Unit：The First Affiliated Hospital of Nanjing Medical University

Drug Manufacturer：Shijiazhuang Yiling Pharmaceutical Co., Ltd

Statistical Analysis：Peking University Clinical Research Institute

Version date：Jan 15th 2023

Author： ____________________ **Date：** ____________________

Approve： ____________________ **Date：** ____________________

目录

1. **ABBREVIATIONS** **9**

2. **STUDY TITLE** **10**

3. **STUDY OBJECTIVE** **10**

4. **STUDY DESIGN** **10**

4.1. CONTROL GROUP AND SAMPLE SIZE 11
4.2. BLINDING AND UNBLINDING 12
4.3.1. Random grouping of subjects *12*
4.3.2. Unblinding and emergency unblinding protocol *12*
4.3.3. Unblinding provisions *13*
4.3.4. Screening number *13*
4.3. TREATMENT PLAN 13
4.4.1. Study Process *13*
4.4.2. Concomitant medications and other treatments *14*
4.4.3. Adverse drug reaction *15*
4.4.4. Evaluation on compliance *15*

5. **RATIONALE FOR STUDY POPULATION** **16**

5.1. INCLUSION CRITERIA 16
5.2. EXCLUSION CRITERIA 16
5.3. PATIENT ENROLMENT AND FOLLOW-UP VISIT 18
5.4. DISCONTINUATION OF INVESTIGATIONAL PRODUCT (IP) 18
5.5. WITHDRAWAL 19
5.6. DISCONTINUATION OF THE STUDY 19

6. **CLINICAL OBSERVATION ENDPOINTS AND INDICATORS** **19**

6.1. CLINICAL OBSERVATION ENDPOINTS 19
6.1.1. Primary outcome measure *19*
6.1.2. Secondary outcome measures *19*
6.2. SAFETY OUTCOME MEASURE: 20
6.2.1. Definition of Adverse Event *20*
6.2.2. Criteria on severity of adverse events: *20*
6.2.3. Adverse events of interest *21*
6.2.4. Definition serious adverse events *22*
6.2.5. Definition on specificity of serious adverse events *22*
6.2.6. Recording of adverse events and follow-up *23*
6.2.7. Adverse events based on examinations *23*
6.3. EFFICACY ASSESSMENTS 23
6.3.1. Endpoint reporting overview *23*
6.3.2. Potential endpoint events *24*

7. DATA MANAGEMENT 26

7.1. DATABASE DESIGN 26
7.2. DATA ENTRY 26
7.3. DATA QUESTIONING MANAGEMENT 27
7.4. MEDICAL CODING 27
7.5. DATA AUDIT 27
7.6. DATABASE LOCKING 27

8. STATISTICAL ANALYSIS METHOD 27

8.1. DEFINITIONS OF ANALYSIS SETS 27
8.2. GENERAL STATISTICAL METHODS 28
8.2.1. Enrollment and completion analysis *29*
8.2.2. Compliance analysis: *29*
8.2.3. Efficacy analysis: *29*
8.2.4. Subgroup analysis: *30*
8.2.5. Safety analysis: *31*
8.3. INTERIM ANALYSIS 32

9. STATISTICAL ANALYSIS RESULTS 33

9.1. CASE DISTRIBUTION (FOR ALL RANDOMIZED PATIENTS) 33
Table 9.1.1 Distribution of cases in each centerDefinitions of analysis sets *33*
Table 9.1.2 Comparison of dropout and elimination between two groups *33*
Table 9.1.3 Population in each center *33*
Table 9.1.4 Enrolled cases and safety and efficacy analysis data sets *34*
9.2. DEMOGRAPHIC AND BASELINE INFORMATION (FAS) 35
Table 9.2.1 Demographic *35*
Table 9.2.2 Personal History *35*
Table 9.2.3 Comorbidities and past medical history *36*
Table 9.2.4 Baseline of past medical history *37*
Table 9.2.5 Analysis of past medical history (SOC/PT) *37*
Table 9.2.6 Analysis of major past medical history （PT） *37*
Table 9.2.7 NYHA heart functional classification *38*
Table 9.2.8 Echocardiogram *38*
Table 9.2.9 Serum NT-proBNP *38*
Table 9.2.10 Vitals *39*
Table 9.2.11 Baseline creatinine and eGFR *40*
9.3. FOLLOW UP PERIOD 40
Table 9.3.1 Follow time descriptions *40*
Table 9.3.2 Median follow time *40*
9.4. MEDICATION (SS) 41
Table 9.4.1 Medication compliance *41*
Table 9.4.2 Past Medication/Concomitant Medication *41*
Table 9.4.3 Coding of Concomitant Medication (ATC1/ATC2) *41*

Table 9.4.4 Usage of Spironolactone, ARNi, SGLT2i, and cardiac glycoside 42
Table 9.4.5 Triple therapy（RAAS+Beta+MRA） 42
9.5. EVALUATION OF PRIMARY EFFICACY INDICATORS (FAS&PPS) 43
Table 9.5.1 Major adverse cardiovascular events (MACE, CV death and HHF) 43
Figure 1 Kaplan-Meier analysis of MACEs (FAS) 43
Figure 2 Kaplan-Meier analysis of MACEs (PPS) 43
Table 9.5.2 Cardiovascular death 44
Figure 3 Kaplan-Meier analysis of Cardiovascular death (FAS) 44
Figure 4 Kaplan-Meier analysis of Cardiovascular death (PPS) 44
Table 9.5.3 Hospitalization of heart failure 45
Figure 5 Kaplan-Meier analysis of Hospitalization of heart failure (FAS) 45
Figure 6 Kaplan-Meier analysis of Hospitalization of heart failure (PPS) 45
9.6. EVALUATION OF MAIN EFFICACY INDICATORS - SUBGROUP ANALYSIS 46
Table 9.6.1 MACE (CV death and HHF) (≤70 year old） 46
Table 9.6.2 MACE (CV death and HHF) (>70 year old） 46
Table 9.6.3 CV Death (≤70 year old) 46
Table 9.6.4 CV Death (>70 year old) 46
Table 9.6.5 HHF (≤70 year old) 47
Table 9.6.6 HHF (>70 year old) 47
Table 9.6.7 MACE (CV death and HHF) (Male) 47
Table 9.6.8 MACE (CV death and HHF) (Female) 47
Table 9.6.9 CV Death (Male) 47
Table 9.6.10 CV Death (Female) 47
Table 9.6.11 HHF (Male) 47
Table 9.6.12 HHF (Female) 47
Table 9.6.13 MACE (CV death and HHF) (NYHA calss I and II) 47
Table 9.6.14 MACE (CV death and HHF) (NYHA calss III and IV) 47
Table 9.6.15 CV Death (NYHA calss I and II) 47
Table 9.6.16 CV Death (NYHA calss III and IV) 47
Table 9.6.17 HHF (NYHA calss I and II) 47
Table 9.6.18 HHF (NYHA calss III and IV) 47
Table 9.6.19 MACE (CV death and HHF)（LVEF≤30%） 48
Table 9.6.20 MACE (CV death and HHF)（LVEF>30%） 48
Table 9.6.21 CV Death（LVEF≤30%） 48
Table 9.6.22 CV Death（LVEF>30%） 48
Table 9.6.23 HHF（LVEF≤30%） 48
Table 9.6.24 HHF（LVEF>30%） 48
Table 9.6.25 MACE (CV death and HHF) (Course of heart failure ≤3 yr) 48
Table 9.6.26 MACE (CV death and HHF) (Course of heart failure >3 yr) 48
Table 9.6.27 CV Death (Course of heart failure ≤3 yr) 48
Table 9.6.28 CV Death (Course of heart failure >3 yr) 48
Table 9.6.29 HHF (Course of heart failure ≤3 yr) 48

Table 9.6.30 HHF (Course of heart failure >3 yr) 48
Table 9.6.31 MACE (CV death and HHF) (with ischemic heart disease) 48
Table 9.6.32 MACE (CV death and HHF) (without ischemic heart disease) 48
Table 9.6.33 CV Death (with ischemic heart disease) 49
Table 9.6.34 CV Death (without ischemic heart disease) 49
Table 9.6.35 HHF (with ischemic heart disease) 49
Table 9.6.36 HHF (without ischemic heart disease) 49
Table 9.6.37 MACE (CV death and HHF) (with non-ischaemic cardiomyopathy) 49
Table 9.6.38 MACE (CV death and HHF) (without cardiomyopathy) 49
Table 9.6.39 CV Death (with non-ischaemic cardiomyopathy) 49
Table 9.6.40 CV Death (without non-ischaemic cardiomyopathy) 49
Table 9.6.41 HHF (with non-ischaemic cardiomyopathy) 49
Table 9.6.42 HHF (without non-ischaemic cardiomyopathy) 49
Table 9.6.43 MACE (CV death and HHF) (with hypertension) 49
Table 9.6.44 MACE (CV death and HHF) (without hypertension) 49
Table 9.6.45 CV Death (with hypertension) 49
Table 9.6.46 CV Death (without hypertension) 49
Table 9.6.47 HHF (with hypertension) 50
Table 9.6.48 HHF (without hypertension) 50
Table 9.6.49 MACE (CV death and HHF) (with diabetes) 50
Table 9.6.50 MACE (CV death and HHF) (without diabetes) 50
Table 9.6.51 CV Death (with diabetes) 50
Table 9.6.52 CV Death (without diabetes) 50
Table 9.6.53 HHF (with diabetes) 50
Table 9.6.54 HHF (without diabetes) 50
Table 9.6.55 MACE (CV death and HHF) (with atrial fibrillation) 50
Table 9.6.56 MACE (CV death and HHF) (without atrial fibrillation) 50
Table 9.6.57 CV Death (with atrial fibrillation) 50
Table 9.6.58 CV Death (without atrial fibrillation) 50
Table 9.6.59 HHF (with atrial fibrillation) 50
Table 9.6.60 HHF (without atrial fibrillation) 50
Table 9.6.61 MACE (CV death and HHF) (with arrhythmias) 51
Table 9.6.62 MACE (CV death and HHF) (without arrhythmias) 51
Table 9.6.63 CV Death (with arrhythmias) 51
Table 9.6.64 CV Death (without arrhythmias) 51
Table 9.6.65 HHF (with arrhythmias) 51
Table 9.6.66 HH (without arrhythmias) 51
Table 9.6.67 MACE (CV death and HHF) (NT-proBNP ≤Median) 51
Table 9.6.68 MACE (CV death and HHF) (NT-proBNP >Median) 51
Table 9.6.69 CV Death (NT-proBNP ≤Median) 51
Table 9.6.70 CV Death (NT-proBNP >Median) 51
Table 9.6.71 HHF (NT-proBNP ≤Median) 51

Table 9.6.72 HHF (NT-proBNP >Median) ... 51
Table 9.6.73 MACE (CV death and HHF) (BMI <25) ... 51
Table 9.6.74 MACE (CV death and HHF) (BMI≥25) ... 52
Table 9.6.75 MACE (CV death and HHF) (eGFR <90) ... 52
Table 9.6.76 MACE (CV death and HHF) (eGFR ≥90） ... 52
Table 9.6.77 MACE (CV death and HHF) (eGFR Untested) ... 52
Table 9.6.78 MACE (CV death and HHF) (with ACEI/ARB/ARNi at baseline) ... 52
Table 9.6.79 MACE (CV death and HHF) (without ACEI/ARB/ARNi at baseline) ... 52
Table 9.6.80 MACE (CV death and HHF) (with Beta blocker at baseline) ... 52
Table 9.6.81 MACE (CV death and HHF) (without Beta blocker at baseline) ... 52
Table 9.6.82 MACE (CV death and HHF) (with MRA at baseline) ... 52
Table 9.6.83 MACE (CV death and HHF) (without MRA at baseline) ... 52
Table 9.6.84 MACE (CV death and HHF) (with ARNi at baseline) ... 52
Table 9.6.85 MACE (CV death and HHF) (without ARNi at baseline) ... 52
Table 9.6.86 MACE (CV death and HHF) (with SGLT2i at baseline) ... 52
Table 9.6.87 MACE (CV death and HHF) (without SGLT2i at baseline) ... 52
Table 9.6.88 MACE (CV death and HHF) (with triple therapy at baseline) ... 52
Table 9.6.89 MACE (CV death and HHF) (without triple therapy at baseline) ... 53
Table 9.6.90 HHF (BMI <25) ... 53
Table 9.6.91 HHF (BMI ≥25) ... 53
Table 9.6.92 HHF (eGFR <90) ... 53
Table 9.6.93 HHF (eGFR ≥90) ... 53
Table 9.6.94 HHF (eGFR Untested) ... 53
Table 9.6.95 HHF (with ACEI/ARB/ARNi at baseline) ... 53
Table 9.6.96 HHF (without ACEI/ARB/ARNi at baseline) ... 53
Table 9.6.97 HHF (with Beta blocker at baseline) ... 53
Table 9.6.98 HHF (without Beta blocker at baseline) ... 53
Table 9.6.99 HHF (with MRA at baseline) ... 53
Table 9.6.100 HHF (without MRA at baseline) ... 53
Table 9.6.101 HHF (with ARNi at baseline) ... 53
Table 9.6.102 HHF (without ARNi at baseline) ... 53
Table 9.6.103 HHF (with SGLT2i at baseline) ... 53
Table 9.6.104 HHF (without SGLT2i at baseline) ... 54
Table 9.6.105 HHF (with triple therapy at baseline) ... 54
Table 9.6.106 HHF (without triple therapy at baseline) ... 54
Table 9.6.107 CV Death (BMI <25) ... 54
Table 9.6.108 CV Death (BMI ≥25) ... 54
Table 9.6.109 CV Death (eGFR <90) ... 54
Table 9.6.110 CV Death (eGFR ≥90) ... 54
Table 9.6.111 CV Death (eGFR Untested) ... 54
Table 9.6.112 CV Death (with ACEI/ARB/ARNi at baseline) ... 54
Table 9.6.113 CV Death (without ACEI/ARB/ARNi at baseline) ... 54

Table 9.6.114 CV Death (with Beta blocker at baseline) 54
Table 9.6.115 CV Death (without Beta blocker at baseline) 54
Table 9.6.116 CV Death (with MRA at baseline) 54
Table 9.6.117 CV Death (without MRA at baseline) 54
Table 9.6.118 CV Death (with ARNi at baseline) 55
Table 9.6.119 CV Death (without ARNi at baseline) 55
Table 9.6.120 CV Death (with SGLT2i at baseline) 55
Table 9.6.121 CV Death (without SGLT2i at baseline) 55
Table 9.6.122 CV Death (with triple therapy at baseline) 55
Table 9.6.123 CV Death (without triple therapy at baseline) 55
9.7. EVALUATION OF SECONDARY EFFICACY INDICATORS (FAS&PPS) 55
Table 9.7.1 All-cause mortality 55
Table 9.7.2 Composite endpoint (consisting of worsening heart failure leading to withdrawal of treatment, successful resuscitation after cardiac arrest, malignant arrhythmia, and non-fatal stroke) 56
Table 9.7.3 Worsening heart failure leading to withdrawal of treatment 56
Table 9.7.4 Successful resuscitation after cardiac arrest 56
Table 9.7.5 Malignant arrhythmia 57
Table 9.7.6 Non-fatal stroke 57
Table 9.7.7 Cardiovascular death and hospitalization for heart failure in patients with ischemic heart disease 58
Table 9.7.8 Serum NT-proBNP level baseline and change from baseline (1month) 58
Table 9.7.9 Seurm NT-proBNP decrease rate from baseline (1 month) 59
Table 9.7.10 Seurm NT-proBNP decrease rate from baseline (3 months) 59
9.8. SAFETY EVALUATION (SS) 59
Table 9.8.1 Summary of Adverse Events 59
Table 9.8.2 Summary of adverse events 60
Table 9.8.3 Coding of adverse event (SOC/PT) 60
Table 9.8.4 Coding of adverse drug reaction (SOC/PT) 60
Table 9.8.5 Comparison of vital signs (12 months ± 7 days) - systolic blood pressure (mmHg) 60
Table 9.8.6 Comparison of vital signs (12 months ± 7 days) - diastolic blood pressure (mmHg) 61
Table 9.8.7 Comparison of vital signs (12 months ± 7 days) - heart rate (bpm) 61
Table 9.8.8 Comparison of vital signs (12 months ± 7 days) - pluse (bpm) 61
Table 9.8.9 Comparison of vital signs (12 months ± 7 days) – Body temperature (℃) 61
Table 9.8.10 Crosstabulation of Complete blood count - white blood cell count (12 months ± 7 days) 62
Table 9.8.11 Crosstabulation of Complete blood count - hemoglobin (12 months ± 7 days) 62
Table 9.8.12 Crosstabulation of Complete blood count - red blood cell count (12 months ± 7 days) 62
Table 9.8.13 Crosstabulation of Complete blood count - platelets (12 months ± 7 days) 62
Table 9.8.14 Summary of Complete blood count - 12 months ± 7 days (SS) 62
Table 9.8.15 Crosstabulation of biochemistry - Alanine aminotransferase (ALT) (12 months ± 7 days) 63
Table 9.8.16 Crosstabulation of biochemistry – Creatinine (Cr) (12 months ± 7 days) 63
Table 9.8.17 Crosstabulation of biochemistry – Blood Uear Nitrogrn (BUN) (12 months ± 7 days) 63
Table 9.8.18 Crosstabulation of biochemistry – Total cholesterol (TC) (12 months ± 7 days) 63
Table 9.8.19 Crosstabulation of biochemistry – Triglyciride (TG) (12 months ± 7 days) 63

Table 9.8.20 Crosstabulation of biochemistry – Potassium (K) (12 months ± 7 days) 63
Table 9.8.21 Crosstabulation of biochemistry – Sodium (Na) (12 months ± 7 days) 63
Table 9.8.22 Crosstabulation of biochemistry – Chlorine (Cl) (12 months ± 7 days) 63
Table 9.8.23 Crosstabulation of biochemistry – Fasting blood glucose (GLU) (12 months ± 7 days) 63
Table 9.8.24 Summary of Biochemistry - 12 months ± 7 days (SS) 63
Table 9.8.25 Crosstabulation of Urine routine – Urine protine (12 months ± 7 days) 64
Table 9.8.26 Crosstabulation of Urine routine – Urine white blood cell (12 months ± 7 days) 64
Table 9.8.27 Crosstabulation of Urine routine – Urine red white blood cell (12 months ± 7 days) 64
Table 9.8.28 Summary of Urine routine - 12 months ± 7 days 64
Table 9.8.29 Crosstabulation of Physical examination – General condition (12 months ± 7 days) 64
Table 9.8.30 Crosstabulation of Physical examination – Nervous system (12 months ± 7 days) 64
Table 9.8.31 Crosstabulation of Physical examination – Skin, superficial lymph nodes (12 months ± 7 days) 64
Table 9.8.32 Crosstabulation of Physical examination – head and neck (12 months ± 7 days) 64
Table 9.8.33 Crosstabulation of Physical examination – Cardiovascular (12 months ± 7 days) 65
Table 9.8.34 Crosstabulation of Physical examination – Respiratory (12 months ± 7 days) 65
Table 9.8.35 Crosstabulation of Physical examination – Abdominal (12 months ± 7 days) 65
Table 9.8.36 Crosstabulation of Physical examination – Genitourinary (12 months ± 7 days) 65
Table 9.8.37 Crosstabulation of Physical examination – Spine and Limbs (12 months ± 7 days) 65
Table 9.8.38 Crosstabulation of Physical examination – Other (12 months ± 7 days) 65
Table 9.8.39 Summary of Urine routine - 12 months ± 7 days 65
Table 9.8.40 Crosstabulation of electrocardiogram (12 months ± 7 days) 66
Table 9.8.41 Summary of electrocardiogram - 12 months ± 7 days 66
9.9. LIST OF APPENDIXES 67

1. Abbreviations

ACEI	Angiotensin converting enzyme inhibitor
ADR	Suspected adverse drug reaction
AEs	Adverse events
AHA	American Heart Association
AMI	Acute myocardial infarction
ARB	Angiotensin receptor blocker
ARNI	Angiotensin receptor neprilysin inhibitor
BNP	B-type natriuretic peptide
CEA	Clinical Event Adjudication
CGRP	Calcitonin gene-related peptide
CHF	Chronic heart failure
CRA	Clinical research auditor
CRC	Clinical research coordinator
CRF	Case report form
CV	Cardiovascular
DSMC	Data and Safety Monitoring Committee
DVP	Data verification plan
ECG	Electrocardiogram
EF	Ejection fraction
EOS	Final visit
ESC	European Society of Cardiology
ET	Endothelin
FAS	Full analysis set
HF	Heart failure
IP	Investigational Product
LVOT	Left ventricular outflow
MedDRA	ICH International Medical Dictionary
MI	Myocardial infarction
NO	Nitric oxide

NT-proBNP	N-terminal pro brain natriuretic peptide
NYHA	New York Heart Association
PPS	Per protocol set
RAAS	Renin-angiotensin-aldosterone system
SAE	Serious adverse event
SNS	Sympathetic nervous system
SS	Safety analysis set
TVI	Time velocity integral
UNS	Unplanned visit

2. Study Title

Qiliqiangxin in Heart FailUre: AssESsment of Reduction in MorTality (QUEST study)

3. Study Objective

Using evidence-based medicine research methods, with cardiovascular mortality and hospital readmission rate for worsening heart failure as the main research endpoints, further elucidating the clinical efficacy and safety of long-term use of Qiliqiangxin capsules (QLQX), clarifying the characteristics of efficacy and the suitable population, to provide high-quality clinical evidence for optimizing HF therapy.

4. Study design

This study is a randomized, double-blind, placebo-controlled, parallel-group, multicenter clinical study.

The study will be event-driven, and all randomized patients will remain in the study (whether taking the study drug or not) until the number of primary endpoint events reaches the predicted value (620 cases), or the study terminates early when it meets the pre-defined efficacy or safety criteria of early termination.

Two mid-term efficacy analyses planned to be conducted after 1/2 and 2/3 primary endpoint events to assess whether an invalid or valid conclusion was reached so as to prematurely end the study.

The entire study will last approximately 36 months, and the recruitment period will be expected to be 24 months. The follow-up period after the last case of patient is included in the study is 12 months. The average follow-up time is predicted to be about 24 months.

Patients who show stable clinical symptoms, had received at least 2 weeks of standardized treatment and treatment of other concomitant diseases before enrollment are screened at the hospital. According to the local HF treatment guidelines, the drug type and dosage are fixed, unless it is contraindicated or intolerant. The patient who have not receive anti-HF drug intravenously for at least two weeks prior to enrollment, nor take oral administration of TCM or Chinese patent medicine having similar composition with Qiliqiangxin Capsule can directly enter the random grouping stage.

If patients fail to meet the above requirements, term standardized treatment to meet the above criteria before entering the random grouping stage are needed.

4.1. Control group and Sample size

According to the PARADIGM-HF study, the composite event of cardiovascular death and/or hospitalization for heart failure in the median follow-up of 27 months was 21.8% in the LCZ696 group and 26.5% in the Enalapril group. Therefore, we estimated that the incidence of cardiovascular death and hospitalization for heart failure is 25% in patients with standardized treatment + placebo group within 36 months of follow-up and 20% in standardized treatment + Qiliqiangxin capsule group.

The random distribution ratio is 1:1 between study group and control group. Considering the consumption of type I error in the interim analysis, α is adjusted to unilateral 0.02314. Based on the incidence of composite endpoint events is 25%, it is expected that 620 composite endpoint events need be observed to provide 80% power of test (β= 0.2), and 20% risk can be reduced in study group by log-rank test.

The entire study will last approximately 36 months to follow up, and the recruitment period are expected to be 24 months. The sample size is expected that 3,080 patients in over 100 centers (1540 patients per group) will be enrolled and be followed up at least for 12 months.

4.2. Blinding and Unblinding

4.3.1. Random grouping of subjects

Statistical experts at Peking University Clinical Research Institute adopts SAS 9.4 statistical software package to generate random numbers using the block randomization method according to the ratio of 1:1 between study group and control group. The study drug (Qiliqiangxin or placebo capsules) was packaged according to this random number by the person unrelated to the study.

A randomization and trail supply management system (RTSM) is used in the study, and statistical professionals will provide a random numbered list to the RTSM. The patient is then assigned a random number by the RTSM.

After completing baseline assessment, random numbers are assigned by RTSM during baseline visits. After that, the drug number is obtained through the RTSM according to the interview plan, and the serial number of drug assigned each time is different, but the drugs are the same. Before randomization of patient, the researcher must first log into the RTSM and provide the according information (e.g. the subject's date of birth, gender).

4.3.2. Unblinding and emergency unblinding protocol

If an adverse event occurs, emergency unblinding can only be conducted in special circumstances where it is necessary to understand the use of the investigational drug in order to treat the patient. Once the decision to unblind has been made, the researcher must record the date, time, and reason for unblinding.

The researcher needs to log in to the RTSM system to complete the unblinding application, which will be reviewed by the principal investigator before being unblinded by the unblinding officer. Once unblinded, the case will be discontinued from the study and treated as a withdrawal.

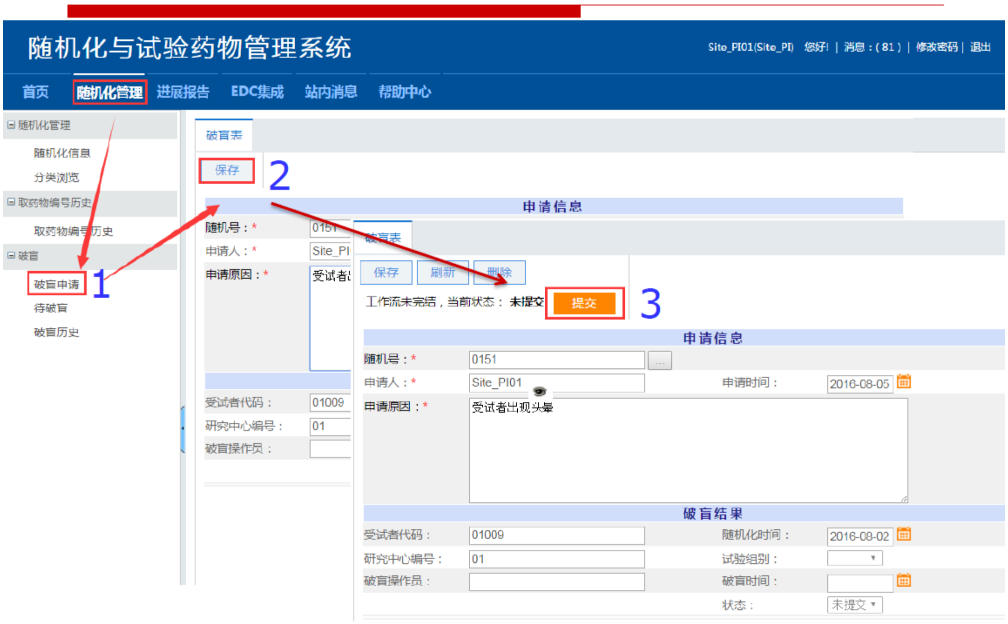

4.3.3. Unblinding provisions

All personnel involved with the analysis of the study will remain blinded until database lock and protocol violations have been identified and documented The study adopts two-step unblinding provision. After blind check, the data is locked, main researchers, medical statisticians, data administrators and sponsor representatives will do the first unblinding, and the random number corresponding to the group will be marked as A or B, in order to make statistical analysis on all data. At the end of statistical analysis when summary report is completed, a secondary unblinding would be taken to reveal the group of A and B.

4.3.4. Screening number

The screening number was made according to the sequence of patients taking treatment at each hospital, and represented with center ID + three integers, such as the screening number is 01001, 01002 ….. for center 01.

4.3. Treatment Plan

4.4.1. Study Process

Enrolment period (day -14 to day 0):

The investigators will review the inclusion and exclusion criteria. Patients who do not meet these criteria must not be randomized into the study. Set of assessments will be completed for these patients.

-Demography (date of birth, sex, race, ethnic group) and relevant medical and surgical history, including smoking history, will be recorded.

-General physical examination (vital signs, NYHA classification, appearance, cardiovascular systems [including edema], etc.)

-Laboratory samples will be collected and sent to the central laboratory

-ECG and echocardiogram will be recorded.

Randomization and Treatment period (day 0 to 12 months [with maximum of 36 months]):

The dispensing date of the IPs is regarded as day 0. Patients will be randomized into the study or control group in a 1:1 ratio with the basis of current standardized treatments for chronic heart failure. The study drug is recommended to be taken about 30 minutes after meals, three times a day as follow.

-Study group: Standardized treatment of chronic heart failure + Qiliqiangxin Capsules (4 capsules/time, 3 times/day);

-Control group: Standardized treatment of chronic heart failure + Placebo Capsules (4 capsules/time, 3 times/day);

This study does not allow dose adjustments. If the patient miss to take the IP in a day, the accumulated dose for the next day should not exceed the daily dose. If the patient has an intolerable adverse event, which is relevant to study drug according to the judgment of researcher, the patient should terminate the following treatment with the study drug.

Do not take drugs at home on the morning of visit day. Investigators will review laboratory results received from the past visit(s). If the patient has experienced any potential endpoints, SAEs, DAEs and/or AEs of interest since the last visit, these should be recorded in the CRF.

4.4.2. Concomitant medications and other treatments

All patients should be treated according to regional standard of care for HF and other comorbidity(s). Also, cardiac and heart failure related procedures will be captured during the study. Background medication will not be provided by the Sponsor.

1) Detailed recording of medications related to HF, HTN as well as other relevant cardiovascular medications (e.g., statins, antihypertensive and antithrombotic agents) will be made throughout

the study;

2) TCM and/or herbal medication have similar contents to the test drug should not be used during the entire treatment period after entering the randomization period.

3) Patients should receive dietary guidance for heart health, such as low-salt diet, moderate drinking, etc. Patients should also receive counseling for appropriate lifestyle improvements such as weight monitoring, physical exercise, smoking cessation, and alcohol withdrawal.

4) No drug has been found to be contraindicated with use of Qiliqiangxin Capsules.

Heart failure medications

Treatment of the patients will be based on the local or regional heart failure guidelines. The treatment regimen from the 2018 China heart failure diagnosis and treatment guideline are summarized as followed:

1) Stable heart failure symptoms without the use of intravenous diuretics, inotropic, and vasodilator for 2 weeks.

2) Patients received standardized baseline treatment regimens without doses adjusted at least two weeks prior to randomization. Standardized treatment includes: angiotensin-converting enzyme inhibitor (ACEI) or angiotensin receptor blocker (ARB) or angiotensin receptor neprilysin inhibitor (ARNI), beta blocker, and aldosterone receptor antagonist (the optimal therapeutic dose should be achieved, except for contraindications or intolerance).

3) After entering the treatment period, Dose reduction or discontinuation of proven effective therapies should be avoided unless all other measures fail to improve the patient's situation. Any adjustment of the treatment regimen should be recorded in the CRF. If the patient has experienced any potential endpoints, SAEs, DAEs and/or AEs of interest since the last visit, these should be recorded in the CRF.

4.4.3. Adverse drug reaction

There is no significant adverse drug reaction of QLQX were found.

4.4.4. Evaluation on compliance

In order to determine the compliance of subjects, the administration (drug distribution and recovery) of all investigational products should be recorded in the appropriate sections of the eCRF. The actual dosage should be within 80%-120% of predefined dosage.

5. Rationale for study population

The enrolled patients should satisfy the following inclusion criteria, and not meet any exclusion criterion. In addition to following criteria, the patient should also be excluded if there is any contraindicated medical condition or use of incompatibility drug during basic treatment period.

5.1. Inclusion criteria

1) Signed informed consent;
2) Aged ≥18 years at the time of consent;
3) Established documented diagnosis of heart failure for at least three months ago according to "Chinese Heart Failure Diagnosis and Treatment Guideline" issued by the Chinese Medical Association Cardiovascular Branch;
4) Left ventricular ejection fraction (LVEF) ≤40% (echocardiogram, radionuclide, ventriculogram, contrast angiography or cardiac MRI);
5) NYHA cardiac functional grading II to III, with stable clinical symptoms; or those diagnosed as grade IV within 2 weeks before enrollment;
6) Serum NT-proBNP ≥450pg/ml;
7) Those who have received standardized baseline treatment regimens without doses adjusted and given intravenously for at least two weeks prior to enrollment; Standardized drug treatment includes angiotensin-converting enzyme inhibitor (ACEI) or angiotensin receptor blocker (ARB) or angiotensin receptor neprilysin inhibitor (ARNI), beta blocker, and aldosterone receptor antagonist (the optimal therapeutic dose should be achieved, except for contraindications or intolerance).

5.2. Exclusion criteria

1) Patients should not enter the study if any of the following exclusion criteria are fulfilled
2) Heart failure caused by valvular disease, congenital heart disease, pericardial disease,

arrhythmia or non-cardiaogenic disease, or caused by vital organ failure (such as renal, hepatic failure, etc.); and right heart failure caused by pulmonary or other definite causes; and acute heart failure;

3) Coronary revascularization (percutaneous coronary intervention [PCI] or coronary artery bypass grafting [CABG]) or cardiac synchronization therapy planned to undergo after randomization, or had received cardiac resynchronization therapy prior to enrolment;
4) Any condition outside the CV diseases such as but not limited to malignant tumor, severe mental illness, hematopoietic diseases, neuroendocrine system disease, liver transaminase and alkaline phosphatase ⩾ 3 x upper limit of normal (ULN), abnormal renal function serum creatinine > 2 mg/dl (176.82 umol/L), potassium >5.5mmol/L;
5) Patient with left ventricular outflow tract obstruction, myocarditis, aortic aneurysm, aortic dissection, or obvious hemodynamic changes caused by unrepaired valve;
6) Cardiogenic shock, uncontrollable malignant arrhythmia, sinus or atrioventricular block at second degree type II or above without pacemaker treatment, progressive unstable angina pectoris or acute myocardial infarction;
7) uncontrolled hypertension systolic blood pressure (SBP) ⩾ 180mmHg and/or diastolic blood pressure (DBP) ⩾ 110mmHg; or SBP < 90mmHg and/or DBP <60mmHg;
8) Participation in another clinical study with an IP during the last month prior to enrolment;
9) Women of child-bearing potential (i.e., those who are not chemically or surgically sterilized or who are not post-menopausal) who are not willing to use a medically accepted method of contraception that is considered reliable in the judgment of the investigator, from the time of signing the informed consent throughout the study and 4 weeks thereafter, OR women who have a positive pregnancy test at enrolment or randomisation OR women who are breast-feeding;
10) Allergic constitution; known to be allergic to research drug;
11) Inability of the patient, in the opinion of the investigator, to understand and/or comply with study medications, procedures or any conditions may render the patient unable to complete the study.

5.3. Patient enrolment and Follow-up visit

Patients who fail to meet the eligibility criteria should not, under any circumstances, be enrolled or receive study medication. Patient should receive at least 2 weeks of standardized treatment. Patient should not receive other traditional Chinese medicine or Chinese patent medicine (with similar function and composition with Qiliqiangxin Capsules).

Patients meet the inclusion criteria can enter the randomization. During this period, the drug and dosage are fixed for each patient. If it is necessary to make adjustment, it should be recorded in the case report form (CRF).

Patients should visit the hospital for efficacy and safety assessment during the 1st, 3rd, 6th, 9th, and 12th months after the random grouping until the study finish.

5.4. Discontinuation of investigational product (IP)

At any time after randomization, patients are free to discontinue for any reason. Discontinuation from IP is not the same as complete withdrawal from the study. According to the intention-to-treat principle, patients discontinuation from IP will be continue in follow-up and recorded in CRF. For patients could not participate in outpatient follow-up, virtual interview and follow-up according to plan will be proceeded for the assessment of the adverse events and/or endpoint events unless the patient refuses to follow up and withdraw from the study. Study drug treatment can be discontinued when:

1. The patient can stop treatment at any time;
2. The patient has allergic reactions that are clearly associated with the IP;
3. The patient has occurrence of symptoms, signs and/or abnormal examination results that are related to the IP, or the condition determined by the investigator to terminate the study;
4. Pregnancy during the study;

During the trial, the patient should take the standard dose of the IP as long as possible. The patients should resume taking the IP as soon as possible after the relevant causes are excluded and follow up as planned.

5.5. Withdrawal

The patient has the right to withdraw from the study at any time for any reason. The withdrawal of the trial will not intervein the regimen and management.

The researcher should retrieve the remaining IP when the patient withdraws from study. The reason for the withdrawal should be recorded in CRF by follow-up interview or telephone. Follow-up should be continued in order to ascertain whether any endpoints or safety events have occurred. Optimally, patients who discontinue from IP should continue to attend all study visits according to plan until study finish as much as possible. Information should be recorded in CRF.

5.6. Discontinuation of the study

1) The overall study may be stopped due to the following reasons:
   - ➢ Base on Data Safety Monitoring Committee (DSMC) interim analysis results;
   - ➢ Researchers find serious safety problems;
   - ➢ Major mistakes in the study protocol;
   - ➢ The sponsors decide to suspend study due to management problems or lack of funding;
   - ➢ The competent administrative department cancels the experiment, and half-stops all studies.
2) The discontinuation of the study can be temporary or permanent. During the suspension, all study records should be kept for inspection.

6. Clinical observation endpoints and indicators

6.1. Clinical observation endpoints

6.1.1. Primary outcome measure

- The composite endpoint events consisting of cardiovascular death and/or hospitalization for heart failure;

6.1.2. Secondary outcome measures

- All-cause mortality
- Secondary endpoint events (given up treatment due to worsening heart failure, successful resuscitation after cardiac arrest, malignant arrhythmia, non-fatal stroke)
- Components of the primary endpoints in patients with ischemic heart disease

- Level of Serum NT-proBNP

Note: All endpoint events should be determined and reviewed by Clinical Event Adjudication Committee.

6.2. Safety outcome measure:

- Adverse events (Serious Adverse Events [SAEs], Discontinuation of IP due to Adverse Events, etc.)
- Clinical laboratory indexes: complete blood count (hemoglobin, red blood cells, white blood cells, platelets), routine urine test (urinary protein, urinary white blood cells, urine red blood cells), serum biochemistry (urea nitrogen, creatinine, alanine aminotransferase, fasting blood glucose, potassium, sodium, chlorine, total cholesterol, triglycerides).
- 12-lead ECG
- Physical examination

6.2.1. Definition of Adverse Event

- Adverse events (AE): AE refers to any adverse medical events occurring in this clinical experiment from the moment that the patient signs the informed consent and is chosen to participate in this study to the last follow-up, whether or not the events are caused by the use of the medicine described.

6.2.2. Criteria on severity of adverse events:

All clinical adverse events that occur in this clinical study will be recorded on the CRF adverse event page. The severity of adverse events will be classified. For uniform standards, the severity of events is classified as follows:

Grade 1: Mild, no clinical symptoms or mild clinical symptoms; only clinical or laboratory abnormalities; no treatment required.

Grade 2: Moderate, requiring minimal, local, or non-invasive treatment; daily life activities using age-appropriate tools a are restricted, such as cooking, shopping, and making phone calls.

Grade 3: Severe illness or severe medical symptoms that are temporarily not life-threatening; resulting in hospitalization or prolonged hospital stay; resulting in disability; restricted in daily

living activities b. Activities of daily living refer to bathing, dressing, undressing, eating, using the toilet, taking medication, and being non-bedridden.

Grade 4: Life-threatening, requiring emergency treatment.

Grade 5: Death due to adverse events.

a: Daily living activities with refer to cooking, buying daily necessities or clothes, using the phone, managing finances, etc.

b: Daily living activities refer to bathing, dressing/undressing, eating, grooming, taking medication, and not being bedridden.

6.2.3. Adverse events of interest

Criteria for assessing the relationship between adverse events and investigational drugs. All causal analyses of adverse events related to investigational drugs are assessed according to five levels: definite, possible, unlikely, definite unrelated, and uncertain. The first three are considered adverse drug reactions. There are five main considerations for causal analysis:

1) Definite: There is a reasonable temporal sequence between the occurrence of the AE and the use of the investigational drug. The AE is a known adverse reaction to the investigational drug. The AE is alleviated or disappears upon discontinuation of the investigational drug, and reoccurs upon repeated use. It cannot be explained by the subject's underlying disease.

2) Possible: There is a reasonable temporal sequence between the occurrence of the AE and the use of the investigational drug. The AE is a known or suspected adverse reaction to the investigational drug, but there may be other factors that could cause the event, such as disease or concurrent medication. The AE is alleviated or disappears upon discontinuation of the investigational drug, or the effect of discontinuation on the event is unclear, or there is a lack of decisive information.

3) Unlikely: There is a reasonable temporal sequence between the occurrence of the AE and the use of the investigational drug, but it is not a known adverse reaction to the investigational drug type, and it is highly likely to be caused by the subject's illness or other treatment.

4) Definite unrelated: There is no reasonable temporal sequence between the AE and the use of the investigational drug, such as events that occurred before the use of the investigational drug,

or events that are not known adverse reactions to the investigational drug, or events that are clearly caused by other factors such as the subject's underlying disease or concurrent medication.

5) Uncertain: There is no clear relationship between the timing of the AE and the medication, and the known type of adverse reaction is similar to the investigational drug. Other co-administered drugs may cause the same reaction, and there is not enough evidence to make a clear decision.

The "definite," "possible," and "uncertain" categories are combined to calculate the incidence of adverse reactions to the investigational drug.

6.2.4. Definition serious adverse events

Serious adverse events (SAE) refer to any clinical events that indicate significant harm, contraindications, adverse reactions, or the need for caution. Adverse events meet the criteria for SAE when they meet one or more of the following standards:

- Death
- Life-threatening (referring to the immediate risk of death for the patient at the time of the event; this does not include events that may lead to death if they become more serious)
- Result in hospitalization or prolonged hospital stay
- Result in persistent or significant work loss or disability
- Congenital malformations or defects

Other events that have not resulted in death, life-threatening situations, or the need for hospitalization but are considered to be harmful to patients or subjects, or require drug or surgical treatment to avoid the above situations upon appropriate medical judgment, should also be considered as SAE.

6.2.5. Definition on specificity of serious adverse events

In this study, the following events will not be reported as serious adverse events unless they are judged negative and the researcher believes that it is related to the study drug

◆ Cardiovascular death

◆ Hospitalization for heart failure

- Given up treatment due to worsening heart failure
- Successful resuscitation after cardiac arrest
- Malignant arrhythmia
- Non-fatal stroke

Other events leading to fatal outcomes should be reported as serious adverse events.

6.2.6. Recording of adverse events and follow-up

If any adverse events occurred, especially those associated with the study drug, should be followed up until the patients return to baseline or tend to stabilize. If the baseline status or stability cannot be restored after follow-up, it should be noted in the CRF. All SAEs must be reported within 24 hours, whether or not considered causally related to the investigational product, or to the study procedure(s). At the same time, researchers must complete a serious adverse event form, recording the time, severity, duration, measures taken, and outcome of serious adverse events.

6.2.7. Adverse events based on examinations

The results from protocol mandated laboratory tests and vital signs will be summarized and give possible interpretation. The abnormal laboratory results caused by reported adverse events should be recorded in the adverse event form. The abnormal results with clinical significance that meets one or more following conditions should be recorded as independent diagnosis on adverse event page of CRF (excluding abnormal laboratory result caused by reported adverse events):

- With associated clinical signs and symptoms
- Change in course of study drug’s treatment dose
- Change in any of standard evidence-based medications and (or) other treatment measures need to be changed.

6.3. Efficacy Assessments

6.3.1. Endpoint reporting overview

When potential endpoint events have been identified, the researchers should collect all relevant support documents within 7 days and report to CEA committee. Investigators will record the incident in endpoint report form and submit supporting data in a timely manner (admission and discharge records, medical records, death records, ECG, etc.). The potential endpoints event

(All deaths, All HF events [hospitalizations for HF or urgent HF visits], cardiac ischemic events [MI and unstable angina], cerebrovascular events [stroke and TIA], etc.) will be reviewed for central CEA process.

CEA committee consists of a chairman and 5-6 members. Each case will be independently reviewed by two members of the committee. The conclusions will be submitted to the chairman of the committee.

6.3.2. Potential endpoint events

For each potential endpoint event, the investigator or delegate will record information in the CRF

- **Hospitalization for heart failure:**

1. The patient was hospitalized for HF diagnosed preliminarily;
2. The patients who were admitted in hospital extended for at least 24 hours (or if the hospitalization time and discharge time were not available, it should indicate change of calendar date);
3. Record on the patient report that there are new symptoms or worsening symptoms due to HF, including at least one of the following:

a. Difficulty breathing (difficulty breathing on exertion, difficulty breathing on resting, orthopnea, paroxysmal breath with difficulty at night)

b. Reduced exercise tolerance

c. Fatigue

4. The patient had objective evidence of an acute exacerbation of HF, including at least two health examination results or a health examination result and at least one laboratory standard, including:

a. Determine the health examination results caused by HF, including new or deteriorated:

1) Peripheral edema; 2) Abdominal distension or increase of ascetic fluid (in the absence of primary liver disease); 3) Lung rales and/or crackles; 4) Increased jugular venous pressure and/or hepatojugular reflex (+); 5) S3 galloping; 6) Clinically significant or rapid weight gain, having relation with fluid retention.

b. Laboratory evidence of new or worsening HF obtained within 24 hours, including:

1) Increased concentration of B-type natriuretic peptide (BNP) / N-terminal B-type natriuretic peptide precursor (NT-proBNP) consistent with acute decompensated HF (eg.: BNP > 500 pg/ml or NT-proBNP > 2000 pg/ml); In patients with long-term elevation of natriuretic peptides, special attention should be paid to a significant increase beyond baseline. 2) Imaging evidence of pulmonary congestion; 3) Non-invasive diagnostic evidence of clinically significant increase in left or right ventricular filling pressure or decreased cardiac output; echocardiographic criteria includes: E/e´>15 or D leading pulmonary venous inflow pattern, congestive inferior vena cava with minimal inspiratory collapse, or reduction of small stroke distance (time velocity integral; TVI) at left ventricular outflow (LVOT). 4) Invasive diagnostic evidence: right heart catheterization showed pulmonary capillary wedge pressure (pulmonary artery wedge pressure) ≧18mmHg, central venous pressure ≧ 12mmHg, or cardiac output index <2.2L/min/m2;

Note: If applicable, all results in the diagnostic test need to be recorded; even if the above criteria are not met, results might provide important information for the determination of the above events.

5. The patients receive an initial or intensive treatment for HF, including at least one of the following:

a. Enhance the treatment of oral diuretics;

b. Intravenous diuretics or vasoactive drugs (such as inotropics, vasopressors or vasodilators);

c. Mechanical or surgical intervention, including:

1) Mechanical circulation support (e.g.: Intra-aortic balloon pump, ventricular assist device, extracorporeal membrane oxygenation, total artificial heart);

2) Mechanically assisted removal of body fluids (e.g.: ultrafiltration, hemofiltration, and dialysis).

- **Cardiovascular death: i**ncluding death caused by acute myocardial infarction (AMI), sudden

cardiac death, acute decompensated heart failure, stroke, cardiovascular (CV) surgery, CV bleeding, and other CV inducing death;

- **All-cause mortality**
- **Given up treatment due to the worsening of HF:** Worsening of heart failure symptoms and signs, requiring intravenous drug or mechanical support treatment, and patients or family members voluntarily give up treatment or left hospital without cure; if the result of follow-up is death, it is included in heart failure death.
- **Successful resuscitation after cardiac arrest**
- **Malignant arrhythmia：** There is no uniform standard for the definition of malignant arrhythmia. It generally refers to arrhythmia that can cause severe hemodynamic disorder in a short period of time, causing syncope or even sudden death. According to this standard, malignant arrhythmia mainly has the following categories: (1) severe bradyarrhythmia, such as severe sick sinus syndrome, high or third degree atrioventricular block; (2) tachyarrhythmia, such as persistent ventricular tachycardia, ventricular flutter, ventricular fibrillation, atrial flutter/atrial fibrillation with rapid ventricular rates, atrioventricular reentry tachycardia, pre-excitation syndrome with atrial fibrillation, sinus tachycardia, etc.
- **Non-fatal stroke**

7. Data Management

This study used Epidata software to collect research data. Data management ensures the authenticity, integrality and accuracy of clinical data. The data management process needs to comply with the regulatory requirements of Clinical Trial Quality Management Regulations and Clinical Trial Data Management Work Technical Guidelines, in order to ensure traceability of study data. The main processes for data management are listed below.

7.1. Database Design

The data administrator adopts the Epidata software to design and release database according to the CRF after testing.

7.2. Data entry

Clinical research coordinator (CRC) is responsible for inputting the CRF data into the database. The data entry adopts secondary recording mode. Two CRC respectively input the data. Data administrator compares the two databases to generate the data inconsistency list. CRC modified the databases respectively according to the list and the CRF, and then made comparison again. The above steps are repeated until the two databases being identical.

7.3. Data questioning management

The data administrator wrote data verification SAS program according to the data verification plan (DVP) to verify and generated a data questioning list. The data questioning table would be generated after manual verification, and clinical research auditor (CRA) gives the data questioning table to the researcher for answer. After the researcher answering the question, CRA returned the data questioning table to data administrator and revised the database accordingly.

7.4. Medical coding

The medical coding of adverse events is done according to MedDRA 21.0 or advance version.

7.5. Data audit

After completion of database cleanup, the data administrator should write Data Verification Report for holding a data verification meeting.

The major recording contents of the audit report: number of enrolled cases, the condition of off cases and exclusion cases, the condition of deviation or violation from the program, compliance data, drug combination, adverse events, data related to the evaluation indicators, etc.

At the data audit meeting, the division of statistical population is discussed and determined according to the content of audit report.

7.6. Database locking

Complete the database locking list and lock the database according to the database locking program. Any issues discovered after locking the data can be corrected in the statistical analysis program once confirmed. If there is solid evidence showing that it is necessary to unlock the data, the researchers and related personnel must sign an unlocking document.

After the database is locked, the data administrator exports the data in SAS format and hands it over to the statistical personnel for statistical analysis.

8. Statistical Analysis Method

After the research protocol is established, the statistical professionals, in collaboration with the primary researchers, will develop a statistical analysis plan. SAS®9.4 software or a higher version will be used for statistical analysis, and PASS13 was used for sample size calculations.

8.1. Definitions of analysis sets

The study population will be divided into the following categories:

- Full Analysis Set (FAS): This refers to the dataset that includes all randomized subjects who received the study drug at least once, with minimal and reasonable exclusion of subjects adhering to the intention-to-treat principle. Exclusions typically include: important inclusion criteria violations, subjects who did not receive the study drug treatment, and subjects with no post-randomization observation data. The primary efficacy endpoint is the time to composite endpoint event, analyzed using survival analysis methods. In selecting FAS for statistical analysis, missing data for the primary endpoint event will be handled by deletion.
- Per Protocol Set (PPS): This is a subset of FAS, in which the subjects have better adherence to the protocol. Subjects included in PPS typically have the following characteristics: (1) completed the minimum exposure to the study drug as predetermined in the protocol, with adherence to drug administration of at least 80%; (2) data for the primary endpoint event are available; and (3) no significant protocol violations.
- Safety Set (SS): This includes all randomized subjects who received at least one treatment and have safety evaluations. No imputations will be made for safety data.

Efficacy analysis will be based on FAS and PPS, while safety analysis will be based on SS. Baseline demographics will be analyzed based on FAS.

8.2. General Statistical methods

Efficacy analysis is taken on the basis of FAS and PPS. All baseline demographic data analysis will be performed on the basis of FAS and safety evaluation on SS.

- All data are performed with two-sided test, and P value less than 0.05 (two-sided test) is considered with statistically significant (If no other specification).
- Descriptive analysis: The number of subjects, mean, median, standard deviation, the first quartile (Q1), the third quartile, minimum, and maximum will be included in the summary statistics of continuous variables. The number of subjects, frequency and percentages will be included in summary statistics of the categorical variables.
- Comparison of general situation should be analyzed with appropriate method based on the type of variables . Continuous variables should be analyzed with paired t-test or Wilcoxon rank-sum test , as appropriate. Categorical variables will be compared with chi-square test or Fisher's

exact test, as appropriate. Ranked variables will be compared with Wilcoxon rank-sum test or CMH test.

8.2.1. Enrollment and completion analysis

The number of subjects enrolled and completed at each center will be summarized, and a list of withdrawal will be provided. Comparisons of group sizes, distribution of cases at each center, dropout rates, and detailed termination reasons will be presented. The demographic characteristics of patients (age, height, vital signs, etc.), medical history, and medication history will be described. The comparability of the two groups in terms of age, height, weight, etc. will also be assessed.

8.2.2. Compliance analysis:

- Medication adherence will be compared between the two groups to evaluate whether the study drug was used on time and in the correct dosage, without using any prohibited drugs or food.
- Co-medication usage will be counted for each group and listed in detail.

8.2.3. Efficacy analysis:

- PP analysis and FAS analysis were performed simultaneously with efficacy evaluation;
- Main efficacy evaluation index is the time when a composite endpoint event (cardiovascular death and hospitalization for deterioration of heart failure) occurs. The lack of primary endpoint events is consodered as censored data.

The main research hypotheses are as following:

H_0: $\lambda_T / \lambda_C \geq 1$

H_1: $\lambda_T / \lambda_C < 1$

λ_T and λ_C represent the risk of the primary endpoint events in the study group and control group respectively. The Kaplan-Meier method will be used to estimate the incidence of clinical endpoints, and a Log-rank test will be performed between the two groups. The Hazard Ratio and its 95% confidence interval between the two groups will be calculated by COX Proportional Hazard Model with center as the covariate. At the same time, two components of the composite endpoint event, the cardiovascular death and hospitalization due to the worsening of heart failure will be analyzed separately.

Since one interim analysis was performed during this trial (see section 8.3) , the type I error for the final analysis should be α=0.02314 (one-sided), that is, 0.04628 (two-sided test). The Hazard Ratio and its 95.372% confidence interval will be calculated for the primary efficacy endpoint at the same time. A P value less than 0.04628 (two-sided test) is considered statistically significant for the final analysis of the primary endpoint.

- Secondary efficacy indicators:
    - All-cause Mortality: The Kaplan-Meier method will be used to estimate the all-cause mortality rates of the two groups, and the Log Rank test will be used for statistical comparison. The COX proportional hazard model will be utilized to estimate the hazard ratios (HRs) and their 95% confidence intervals (CIs) with center as a covariate.
    - Composite endpoint (consisting of worsening heart failure leading to withdrawal of treatment, successful resuscitation after cardiac arrest, malignant arrhythmia, and non-fatal stroke): Analyzed using the same method as all-cause mortality.
    - Cardiovascular death and hospitalization for heart failure in patients with ischemic heart disease: Analyzed as all-cause mortality.
  - Serum NT-proBNP level: Analyzed using measurement data, with statistical description and inter-group comparison for the two groups' baseline and change from baseline.

8.2.4. Subgroup analysis:

Subgroup analysis will be conducted in the study to compare the efficacy between subgroups. For each subgroup, the primary efficacy endpoint will be analyzed and 2 components of the composite endpoint event, the cardiovascular death and hospitalization due to the worsening of heart failure will be analyzed separately. The hazard ratio and corresponding 95%CI will be computed and compared between study group and control group within each subgroup. The exploratory subgroup analysis will be based on FAS and PPS.

19 subgroups will be included in the subgroup analysis:

- Age: ≤70 yr, >70 yr
- Sex: Male, Female
- NYHA class: class I & II, class III & IV

- LVEF: ≤30%, >30%
- Course of heart failure: ≤3 yr, >3 yr
- Ischemic heart disease: Yes, No
- Non-ischaemic cardiomyopathy: Yes, No
- Hypertension: Yes, No
- Diabetes: Yes, No
- Atrial fibrillation: Yes, No
- Arrhythmias: Yes, No
- NT-proBNP: ≤median (based on FAS) , > median (based on FAS)
- BMI: <25, ≥25
- eGFR: <90, ≥90, Untested
- ACEI/ARB/ARNi at baseline: Yes, No
- MRA at baseline: Yes, No
- Beta-Blocker at baseline: Yes, No
- SGLT2i at baseline: Yes, No
- Triple therapy: Yes, No

8.2.5. Safety analysis:

Safety analysis will be based on SS.

Data of adverse events (case number, times and incidence of various adverse events) are compared between the two groups. At the same time, detailed description of specific manifestation, extent of all adverse events and the relation with drugs would be further analyzed.

Crosstab is adopted to describe the change of laboratory index. Number of normal cases before treatment, number of abnormal cases after treatment and ratio of abnormal cases are analyzed in study group and control group. Indexes of vital signs are compared between before and after treatment.

8.3. Interim analysis

The study plans to perform two interim efficacy analyses after collecting 1/2 and 2/3 of the primary endpoints to assess whether a valid conclusion has been reached and then terminate the study early. According to Lan-DeMets α spending function and the O'Brien-Fleming method, the spending type I error was α= 0.0001 (one-sided) in the first interim analysis period, and α= 0.00605 (one-sided) in the second interim analysis period.

The specific requirements and operations related to the interim analysis will be specified in the DSMB in advance.

Only one interim analysis was performed after 2/3 of the primary endpoint events were collected due to pandemic. During the interim analysis, the one-sided α=0.006048 was used, and the two-sided α is 0.012097. The type I error for the final analysis should be α=0.02314 (one-sided).

9. Statistical Analysis Results

9.1. Case distribution (for all randomized patients)

Table 9.1.1 Distribution of cases in each centerDefinitions of analysis sets

Centers	Group	Enrollment	Dropout	Dropout Rate (%)	Elimination	Elimination Rate (%)	Complet
The First Affiliated Hospital	Study						
With Nanjing Medical University	Placebo						
	Total						
Hebei Yiling Hospital	Study						
	Placebo						
	Total						
……	Study						
	Placebo						
	Total						
Overall	Study						
	Placebo						
	Total						

Table 9.1.2 Comparison of dropout and elimination between two groups

Group	Dropout Rate (%)	P	Elimination Rate (%)	P
QLQX				
Placebo				

Note: Dropout refers to subjects who did not complete the trial, and elimination refers to subjects who completed the trial but were excluded from the PPS set.

Table 9.1.3 Population in each center

Centers	PPS			FAS			SS		
	Study	Placebo	Total	Study	Placebo	Total	Study	Placebo	Total
The First Affiliated Hospital With Nanjing Medical University									
Hebei Yiling Hospital									
……									
Overall									

Note: The center of the following table is indicated by the center number

Table 9.1.4 Enrolled cases and safety and efficacy analysis data sets

Study Set	Study Group	Placebo Group	Total
Full analysis set (FAS)			
Randomizied entry			
Complet Trial			
Discontinuation during the trial			
Eliminated			
Adverse events			
Lack of efficacy			
Protocol violations (including poor compliance)			
Withdrawal of informed consent			
Lost to follow-up			
Other reasons			
Safety Analysis Set			
Safety set (SS)			
Efficacy Analysis Sets			
FAS			
Per Protocol Set (PPS)			

9.2. Demographic and Baseline Information (FAS)

Table 9.2.1 Demographic

Item	Index	Study Group	Placebo Group	Total	Statistics	P
Age, yrs	N(Missing)					
	Mean(Sd)					
	Median					
	Q1,Q3					
	Min,Max					
Sex	Male, n (%)					
	Female, n (%)					
	Total (Missing)					
Marriage	Unmarried, n(%)					
	Married, n(%)					
	Other, n(%)					
	Total (Missing)					
Ethnicity	Han, n (%)					
	Other, n (%)					
	Total (Missing)					
Height (cm)	N(Missing)					
	Mean (Sd)					
	Median					
	Q1, Q3					
	Min, Max					
Weight (kg)	N(Missing)					
	Mean (Sd)					
	Median					
	Q1, Q3					
	Min, Max					
BMI(kg/m^2)	N(Missing)					
	Mean (Sd)					
	Median					
	Q1, Q3					
	Min, Max					
Nationality	Chinese, n (%)					
	Other, n (%)					
	Total (Missing)					

Table 9.2.2 Personal History

Item	Index	Study Group	Placebo Group	Total	Statistics	P
Smoking	Yes n(%)					
	No n(%)					
	Quitted n(%)					
	Total (Missing)					

Item	Index	Study Group	Placebo Group	Total	Statistics	P
Dayily Cigarettes	N(Missing)					
	Mean(Sd)					
	Median					
	Q1,Q3					
	Min,Max					
Alcohol Consumption	Yes n(%)					
	No n(%)					
	Quitted n(%)					
	Total (Missing)					
Drinking Quantity (Unit/ 50g*d)	N(Missing)					
	Mean(Sd)					
	Median					
	Q1,Q3					
	Min,Max					
Menstration	Non-menopausal n(%)					
	Menopausal n(%)					
	Not applicable n(%)					
	Total (Missing)					
History of allergy	Yes n(%)					
	No n(%)					
	Total (Missing)					

Table 9.2.3 Comorbidities and past medical history

Item	Index	Study Group	Placebo Group	Total	Statistics	P
Ischemic heart disease	No n(%)					
	Yes n(%)					
	Total (Missing)					
Non-ischaemic cardiomyopathy	No n(%)					
	Yes n(%)					
	Total (Missing)					
Hypertension	No n(%)					
	Yes n(%)					
	Total (Missing)					
Other	No n(%)					
	Yes n(%)					
	Total (Missing)					
Heart failure course	≤3 year					
	>3 year					
	Total (Missing)					
Heart failure course (months)	N(Missing)					
	Mean(Sd)					
	Median					

Item	Index	Study Group	Placebo Group	Total	Statistics	P
	Q1,Q3					
	Min,Max					

Table 9.2.4 Baseline of past medical history

Item	Index	Study Group	Placebo Group	Total	Statistics	P
Myocarial Infraction	No n(%)					
	Yes n(%)					
	Total (Missing)					
Hypertension	No n(%)					
	Yes n(%)					
	Total (Missing)					
Diabetes	No n(%)					
	Yes n(%)					
	Total (Missing)					
Hyperlipidemia	No n(%)					
	Yes n(%)					
	Total (Missing)					
Stroke	No n(%)					
	Yes n(%)					
	Total (Missing)					
Atrial fibrillation	No n(%)					
	Yes n(%)					
	Total (Missing)					
Arrhythmia	No n(%)					
	Yes n(%)					
	Total (Missing)					

Note: Medical history is categorized by the corresponding coding.

Table 9.2.5 Analysis of past medical history (SOC/PT)

	Study Group			Placebo Group			
Item	Incident	Patients	N (%)	Incident	Patients	N (%)	P
Past medical history							

Table 9.2.6 Analysis of major past medical history（PT）

	Study Group			Placebo Group			
Item	Incident	Patients	N (%)	Incident	Patients	N (%)	P
Hypertension							
DIabetes							

Item	Study Group			Placebo Group			
	Incident	Patients	N (%)	Incident	Patients	N (%)	P
Hyperlipidemia							
Atrial fibrillation							
Myocarial Infraction							
Stroke							
Arrhythmia							

The above medical histories are classified according to coding.

Table 9.2.7 NYHA heart functional classification

Item	Index	Study	Placebo	Total	Statistics	P
NYHA class	Class I, No limitation of physical activity;※Should not be included; n(%)					
	Calss II, Slight limitation of physical activity (fatigue, dyspnea); n(%)					
	Class III, Marked limitation of physical activity; n(%)					
	Class IV, Unable to carry on any physical activity;※inclusion assessment					
	Total (Missing)					

Table 9.2.8 Echocardiogram

Item	Index	Study	Placebo	Total	Statistics	P
Underwent examination	Yes n(%)					
	No n(%)					
	Total (Missing)					
LVEF (%)	N(Missing)					
	Mean(Sd)					
	Median					
	Q1,Q3					
	Min,Max					

Table 9.2.9 Serum NT-proBNP

Item	Index	Study	Placebo	Total	Statistics	P
Testing on Visit 1	Yes n(%)					
	No n(%)					
	Total (Missing)					
NT-proBNP (pg/ml)	N(Missing)					

Item	Index	Study	Placebo	Total	Statistics	P
	Mean(Sd)					
	Median					
	Q1,Q3					
	Min,Max					
Testing on Visit 2	Yes n(%)					
	No n(%)					
	Total (Missing)					
NT-proBNP (pg/ml)	N(Missing)					
	Mean(Sd)					
	Median					
	Q1,Q3					
	Min,Max					
Testing on Visit 3	Yes n(%)					
	No n(%)					
	Total (Missing)					
NT-proBNP (pg/ml)	N(Missing)					
	Mean(Sd)					
	Median					
	Q1,Q3					
	Min,Max					

Table 9.2.10 Vitals

Item	Index	Study	Placebo	Total	Statistics	P
SBP (mmHg)	N(Missing)					
	Mean(Sd)					
	Median					
	Q1,Q3					
	Min,Max					
DBP (mmHg)	N(Missing)					
	Mean(Sd)					
	Median					
	Q1,Q3					
	Min,Max					
Heart rate (bpm)	N(Missing)					
	Mean(Sd)					
	Median					
	Q1,Q3					
	Min,Max					
Pluse (bpm)	N(Missing)					
	Mean(Sd)					
	Median					
	Q1,Q3					
	Min,Max					
Temperature (°C)	N(Missing)					
	Mean(Sd)					
	Median					

Item	Index	Study	Placebo	Total	Statistics	P
	Q1,Q3					
	Min,Max					

Table 9.2.11 Baseline creatinine and eGFR

Item	Index	Study	Placebo	Total	Statistics	P
Baseline creatinine	N(Missing)					
	Mean(Sd)					
	Median					
	Q1,Q3					
	Min,Max					
eGFR	N(Missing)					
	Mean(Sd)					
	Median					
	Q1,Q3					
	Min,Max					
eGFR Class	$<$90ml/min/1.73m^2 n(%)					
	⩾90ml/min/1.73m^2 n(%)					
	Missing n(%)					
	Total (Missing)					

Note: eGFR is classified according to 90ml/min/1.73 m2

9.3. Follow up period

Table 9.3.1 Follow time descriptions

		FAS		PPS	
Item	Index	Study	Placebo	Study	Placebo
Follow-up (Day)	N(Missing)				
	Mean(Sd)				
	Median				
	Q1,Q3				
	Min,Max				
Comparsion	Statistics				
	P				

Table 9.3.2 Median follow time

		FAS		PPS	
Item	Index	Study	Placebo	Study	Placebo
Follow up	N(Missing)				
	Endpoint event (%)				
	Censored (%)				
	Median				

Item	Index	FAS		PPS	
		Study	Placebo	Study	Placebo
	95%CI				
	Q1,Q3				
	Log-rank test				
	P				
Cox regression	Hazard Ratio				
	95%CI				
	Wald χ^2				
	P				

9.4. Medication (SS)

Table 9.4.1 Medication compliance

Item	Index	Study	Placebo	Total	Statistics	P
Medication Compliance	<80% n(%)					
	80%-120% n(%)					
	>120% n(%)					
	Total (Missing)					

Note: The actual dose of drugs taken is within the range of 80% to 120% of the amount of drugs used, which can be judged as compliance with the protocol requirements.

Table 9.4.2 Past Medication/Concomitant Medication

Item	Index	Study	Placebo	Total	Statistics	P
Past Medication/ Concomitant Medication	No n(%)					
	Yes n(%)					
	Total (Missing)					

Table 9.4.3 Coding of Concomitant Medication (ATC1/ATC2)

Item	Study Group			Placebo Group			P
	Incident	Patients	N (%)	Incident	Patients	N (%)	
ATC1							
ATC2							

Table 9.4.4 Usage of Spironolactone, ARNi, SGLT2i, and cardiac glycoside

Item	Index	Study	Placebo	Total	Statistics	P
Spironolactone	No n(%)					
	Yes n(%)					
	Total (Missing)					
ARNi	No n(%)					
	Yes n(%)					
	Total (Missing)					
SGLT2i	No n(%)					
	Yes n(%)					
	Total (Missing)					
Cardiac glycoside	No n(%)					
	Yes n(%)					
	Total (Missing)					

Table 9.4.5 Triple therapy（RAAS+Beta+MRA）

Item	Index	Study	Placebo	Total	Statistics	P
RAAS+Beta+MRA	No n(%)					
	Yes n(%)					
	Total (Missing)					

9.5. Evaluation of primary efficacy indicators (FAS&PPS)

Table 9.5.1 Major adverse cardiovascular events (MACE, CV death and HHF)

		FAS		PPS	
Item	Index	Study	Placebo	Study	Placebo
MACE time	N(Missing)				
	Endpoint event (%)				
	Censored (%)				
	Median				
	95%CI				
	Q1,Q3				
	Log-rank test				
	P				
Cox Regression	Hazard Ratio				
	95%CI				
	95.372%CI				
	Wald χ^2				
	P				

*Cox regression covariates is center, the same below。

Figure 1 Kaplan-Meier analysis of MACEs (FAS)

Figure 2 Kaplan-Meier analysis of MACEs (PPS)

Table 9.5.2 Cardiovascular death

		FAS		PPS	
Item	Index	Study	Placebo	Index	Placebo
CV Death	N(Missing)				
	Endpoint event (%)				
	Censored (%)				
	Median				
	95%CI				
	Q1,Q3				
	Log-rank test				
	P				
Cox Regression	Hazard Ratio				
	95%CI				
	95.372%CI				
	Wald χ^2				
	P				

Figure 3 Kaplan-Meier analysis of Cardiovascular death (FAS)

Figure 4 Kaplan-Meier analysis of Cardiovascular death (PPS)

Table 9.5.3 Hospitalization of heart failure

		FAS			PPS
Item	Index	Study	Item	Index	Study
Hospitalization of heart failure	N(Missing)				
	Endpoint event (%)				
	Censored (%)				
	Median				
	95%CI				
	Q1,Q3				
	Log-rank test				
	P				
Cox Regression	Hazard Ratio				
	95%CI				
	95.372%CI				
	Wald χ^2				
	P				

Figure 5 Kaplan-Meier analysis of Hospitalization of heart failure (FAS)

Figure 6 Kaplan-Meier analysis of Hospitalization of heart failure (PPS)

9.6. Evaluation of main efficacy indicators - subgroup analysis

Table 9.6.1 MACE (CV death and HHF) (≤70 year old）

Item	Index	FAS Study	FAS Placebo	PPS Study	PPS Placebo
MACE	N(Missing)				
	Endpoint event (%)				
	Censored (%)				
	Median				
	95%CI				
	Q1,Q3				
	Log-rank test				
	P				
Cox Regression	Hazard Ratio				
	95%CI				
	Wald χ^2				
	P				

Table 9.6.2 MACE (CV death and HHF) (>70 year old）

Item	Index	FAS Study	FAS Placebo	PPS Study	PPS Placebo
MACE	N(Missing)				
	Endpoint event (%)				
	Censored (%)				
	Median				
	95%CI				
	Q1,Q3				
	Log-rank test				
	P				
Cox Regression	Hazard Ratio				
	95%CI				
	Wald χ^2				
	P				

Table 9.6.3 CV Death (≤70 year old)

The following subgroup analysis results, the form of the table is the same as above

Table 9.6.4 CV Death (>70 year old)

Table 9.6.5 HHF (≤70 year old)

Table 9.6.6 HHF (>70 year old)

Table 9.6.7 MACE (CV death and HHF) (Male)

Table 9.6.8 MACE (CV death and HHF) (Female)

Table 9.6.9 CV Death (Male)

Table 9.6.10 CV Death (Female)

Table 9.6.11 HHF (Male)

Table 9.6.12 HHF (Female)

Table 9.6.13 MACE (CV death and HHF) (NYHA calss I and II)

Table 9.6.14 MACE (CV death and HHF) (NYHA calss III and IV)

Table 9.6.15 CV Death (NYHA calss I and II)

Table 9.6.16 CV Death (NYHA calss III and IV)

Table 9.6.17 HHF (NYHA calss I and II)

Table 9.6.18 HHF (NYHA calss III and IV)

Table 9.6.19 MACE (CV death and HHF)（LVEF≤30%）

Table 9.6.20 MACE (CV death and HHF)（LVEF>30%）

Table 9.6.21 CV Death（LVEF≤30%）

Table 9.6.22 CV Death（LVEF>30%）

Table 9.6.23 HHF（LVEF≤30%）

Table 9.6.24 HHF（LVEF>30%）

Table 9.6.25 MACE (CV death and HHF) (Course of heart failure ≤3 yr)

Table 9.6.26 MACE (CV death and HHF) (Course of heart failure >3 yr)

Table 9.6.27 CV Death (Course of heart failure ≤3 yr)

Table 9.6.28 CV Death (Course of heart failure >3 yr)

Table 9.6.29 HHF (Course of heart failure ≤3 yr)

Table 9.6.30 HHF (Course of heart failure >3 yr)

Table 9.6.31 MACE (CV death and HHF) (with ischemic heart disease)

Table 9.6.32 MACE (CV death and HHF) (without ischemic heart disease)

Table 9.6.33 CV Death (with ischemic heart disease)

Table 9.6.34 CV Death (without ischemic heart disease)

Table 9.6.35 HHF (with ischemic heart disease)

Table 9.6.36 HHF (without ischemic heart disease)

Table 9.6.37 MACE (CV death and HHF) (with non-ischaemic cardiomyopathy)

Table 9.6.38 MACE (CV death and HHF) (without non-ischaemic cardiomyopathy)

Table 9.6.39 CV Death (with non-ischaemic cardiomyopathy)

Table 9.6.40 CV Death (without non-ischaemic cardiomyopathy)

Table 9.6.41 HHF (with non-ischaemic cardiomyopathy)

Table 9.6.42 HHF (without non-ischaemic cardiomyopathy)

Table 9.6.43 MACE (CV death and HHF) (with hypertension)

Table 9.6.44 MACE (CV death and HHF) (without hypertension)

Table 9.6.45 CV Death (with hypertension)

Table 9.6.46 CV Death (without hypertension)

Table 9.6.47 HHF (with hypertension)

Table 9.6.48 HHF (without hypertension)

Table 9.6.49 MACE (CV death and HHF) (with diabetes)

Table 9.6.50 MACE (CV death and HHF) (without diabetes)

Table 9.6.51 CV Death (with diabetes)

Table 9.6.52 CV Death (without diabetes)

Table 9.6.53 HHF (with diabetes)

Table 9.6.54 HHF (without diabetes)

Table 9.6.55 MACE (CV death and HHF) (with atrial fibrillation)

Table 9.6.56 MACE (CV death and HHF) (without atrial fibrillation)

Table 9.6.57 CV Death (with atrial fibrillation)

Table 9.6.58 CV Death (without atrial fibrillation)

Table 9.6.59 HHF (with atrial fibrillation)

Table 9.6.60 HHF (without atrial fibrillation)

Table 9.6.61 MACE (CV death and HHF) (with arrhythmias)

Table 9.6.62 MACE (CV death and HHF) (without arrhythmias)

Table 9.6.63 CV Death (with arrhythmias)

Table 9.6.64 CV Death (without arrhythmias)

Table 9.6.65 HHF (with arrhythmias)

Table 9.6.66 HH (without arrhythmias)

Table 9.6.67 MACE (CV death and HHF) (NT-proBNP ≤Median)

Note: The median of NT-proBNP is based on FAS.

Table 9.6.68 MACE (CV death and HHF) (NT-proBNP >Median)

Note: The median of NT-proBNP is based on FAS.

Table 9.6.69 CV Death (NT-proBNP ≤Median)

Note: The median of NT-proBNP is based on FAS.

Table 9.6.70 CV Death (NT-proBNP >Median)

Note: The median of NT-proBNP is based on FAS.

Table 9.6.71 HHF (NT-proBNP ≤Median)

Note: The median of NT-proBNP is based on FAS.

Table 9.6.72 HHF (NT-proBNP >Median)

Note: The median of NT-proBNP is based on FAS.

Table 9.6.73 MACE (CV death and HHF) (BMI <25)

Table 9.6.74 MACE (CV death and HHF) (BMI⩾25)

Table 9.6.75 MACE (CV death and HHF) (eGFR <90)

Table 9.6.76 MACE (CV death and HHF) (eGFR ⩾90）

Table 9.6.77 MACE (CV death and HHF) (eGFR Untested)

Table 9.6.78 MACE (CV death and HHF) (with ACEI/ARB/ARNi at baseline)

Table 9.6.79 MACE (CV death and HHF) (without ACEI/ARB/ARNi at baseline)

Table 9.6.80 MACE (CV death and HHF) (with Beta blocker at baseline)

Table 9.6.81 MACE (CV death and HHF) (without Beta blocker at baseline)

Table 9.6.82 MACE (CV death and HHF) (with MRA at baseline)

Table 9.6.83 MACE (CV death and HHF) (without MRA at baseline)

Table 9.6.84 MACE (CV death and HHF) (with ARNi at baseline)

Table 9.6.85 MACE (CV death and HHF) (without ARNi at baseline)

Table 9.6.86 MACE (CV death and HHF) (with SGLT2i at baseline)

Table 9.6.87 MACE (CV death and HHF) (without SGLT2i at baseline)

Table 9.6.88 MACE (CV death and HHF) (with triple therapy at baseline)

Table 9.6.89 MACE (CV death and HHF) (without triple therapy at baseline)

Table 9.6.90 HHF (BMI <25)

Table 9.6.91 HHF (BMI ⩾25)

Table 9.6.92 HHF (eGFR <90)

Table 9.6.93 HHF (eGFR ⩾90)

Table 9.6.94 HHF (eGFR Untested)

Table 9.6.95 HHF (with ACEI/ARB/ARNi at baseline)

Table 9.6.96 HHF (without ACEI/ARB/ARNi at baseline)

Table 9.6.97 HHF (with Beta blocker at baseline)

Table 9.6.98 HHF (without Beta blocker at baseline)

Table 9.6.99 HHF (with MRA at baseline)

Table 9.6.100HHF (without MRA at baseline)

Table 9.6.101HHF (with ARNi at baseline)

Table 9.6.102HHF (without ARNi at baseline)

Table 9.6.103HHF (with SGLT2i at baseline)

Table 9.6.104HHF (without SGLT2i at baseline)

Table 9.6.105HHF (with triple therapy at baseline)

Table 9.6.106HHF (without triple therapy at baseline)

Table 9.6.107CV Death (BMI <25)

Table 9.6.108CV Death (BMI ⩾25)

Table 9.6.109CV Death (eGFR <90)

Table 9.6.110CV Death (eGFR ⩾90)

Table 9.6.111CV Death (eGFR Untested)

Table 9.6.112CV Death (with ACEI/ARB/ARNi at baseline)

Table 9.6.113CV Death (without ACEI/ARB/ARNi at baseline)

Table 9.6.114CV Death (with Beta blocker at baseline)

Table 9.6.115CV Death (without Beta blocker at baseline)

Table 9.6.116CV Death (with MRA at baseline)

Table 9.6.117CV Death (without MRA at baseline)

Table 9.6.118CV Death (with ARNi at baseline)

Table 9.6.119CV Death (without ARNi at baseline)

Table 9.6.120CV Death (with SGLT2i at baseline)

Table 9.6.121CV Death (without SGLT2i at baseline)

Table 9.6.122CV Death (with triple therapy at baseline)

Table 9.6.123CV Death (without triple therapy at baseline)

9.7. Evaluation of secondary efficacy indicators (FAS&PPS)

Table 9.7.1 All-cause mortality

		FAS		PPS	
Item	Index	Study	Placebo	Study	Placebo
All-cause mortality	N(Missing)				
	Endpoint event (%)				
	Censored (%)				
	Median				
	95%CI				
	Q1,Q3				
	Log-rank test				
	P				
Cox Regression	Hazard Ratio				
	95%CI				
	Wald χ^2				
	P				

Table 9.7.2 Composite endpoint (consisting of worsening heart failure leading to withdrawal of treatment, successful resuscitation after cardiac arrest, malignant arrhythmia, and non-fatal stroke)

Item	Index	FAS		PPS	
		Study	Placebo	Study	Placebo
secondary composite outcome (abandoning treatment due to heart failure exacerbation, cardiac arrest resuscitation, malignant arrhythmia, non-fatal stroke)	N(Missing)				
	Endpoint event (%)				
	Censored (%)				
	Median				
	95%CI				
	Q1,Q3				
	Log-rank test				
	P				
Cox Regression	Hazard Ratio				
	95%CI				
	Wald χ^2				
	P				

Table 9.7.3 Worsening heart failure leading to withdrawal of treatment

Item	Index	FAS		PPS	
		Study	Placebo	Study	Placebo
worsening heart failure leading to withdrawal of treatment	N(Missing)				
	Endpoint event (%)				
	Censored (%)				
	Median				
	95%CI				
	Q1,Q3				
	Log-rank test				
	P				
Cox Regression	Hazard Ratio				
	95%CI				
	Wald χ^2				
	P				

Table 9.7.4 Successful resuscitation after cardiac arrest

Item	Index	FAS		PPS	
		Study	Placebo	Study	Placebo
successful resuscitation after cardiac arrest	N(Missing)				
	Endpoint event (%)				
	Censored (%)				
	Median				
	95%CI				
	Q1,Q3				
	Log-rank test				

Item	Index	FAS		PPS	
		Study	Placebo	Study	Placebo
	P				
Cox Regression	Hazard Ratio				
	95%CI				
	Wald χ^2				
	P				

Table 9.7.5 Malignant arrhythmia

Item	Index	FAS		PPS	
		Study	Placebo	Study	Placebo
malignant arrhythmia	N(Missing)				
	Endpoint event (%)				
	Censored (%)				
	Median				
	95%CI				
	Q1,Q3				
	Log-rank test				
	P				
Cox Regression	Hazard Ratio				
	95%CI				
	Wald χ^2				
	P				

Table 9.7.6 Non-fatal stroke

Item	Index	FAS		PPS	
		Study	Placebo	Study	Placebo
non-fatal stroke	N(Missing)				
	Endpoint event (%)				
	Censored (%)				
	Median				
	95%CI				
	Q1,Q3				
	Log-rank test				
	P				
Cox Regression	Hazard Ratio				
	95%CI				
	Wald χ^2				
	P				

Table 9.7.7 Cardiovascular death and hospitalization for heart failure in patients with ischemic heart disease

Item	Index	FAS Study	FAS Placebo	PPS Study	PPS Placebo
MACE	Yes n(%)				
	No n(%)				
	Total (Missing)				
	Statistics				
	P				
CV death	Yes n(%)				
	No n(%)				
	Total (Missing)				
	Statistics				
	P				
HHF	Yes n(%)				
	No n(%)				
	Total (Missing)				
	Statistics				
	P				

Table 9.7.8 Serum NT-proBNP level baseline and change from baseline (1month)

Item	Index	FAS Study	FAS Placebo	PPS Study	PPS Placebo
Baseline NT-proBNP (pg/ml)	N(Missing)				
	Mean(Sd)				
	Median				
	Q1,Q3				
	Min,Max				
Baseline comparsion	Statistics				
	P				
1-month	N(Missing)				
	Mean(Sd)				
	Median				
	Q1,Q3				
	Min,Max				
1-month comparsion	Statistics				
	P				
1-month and baseline difference	N(Missing)				
	Mean(Sd)				
	Median				
	Q1,Q3				

Item	Index	FAS		PPS	
		Study	Placebo	Study	Placebo
	Min,Max				
1-month intragroup difference	Statistics				
	P				
Inter-group difference	Statistics				
	P				

Table 9.7.8 Serum NT-proBNP level baseline and change from baseline (1month)

Format same as above

Table 9.7.9 Seurm NT-proBNP decrease rate from baseline (1 month)

Item	Index	FAS		PPS	
		Study	Placebo	Study	Placebo
Decrease rate of marker (%)	N(Missing)				
	Mean(Sd)				
	Median				
	Q1,Q3				
	Min,Max				
Comparison	Statistics				
	P				
Decrease rate ≥30%	No n(%)				
	Yes n(%)				
	Total (Missing)				
Comparison	Statistics				
	P				

Note: Decrease rate = (baseline result - 1 month result) / baseline result * 100%

Table 9.7.10 Seurm NT-proBNP decrease rate from baseline (3 months)

Format same as above

9.8. Safety Evaluation (SS)

Table 9.8.1 Summary of Adverse Events

Item	Index	Study	Placebo	Total	Statistics	P
Adverse Events	No n(%)					
	Yes n(%)					
	Total (Missing)					
Major Adverse Events	No n(%)					
	Yes n(%)					

Item	Index	Study	Placebo	Total	Statistics	P
	Total (Missing)					

Table 9.8.2 Summary of adverse events

Item	Study Group Incident	Study Group Patients N (%)	Placebo Group Incident	Placebo Group Patients N (%)	P
Adverse event					
Adverse events related to study drug					
Severity grade 3 4 5 adverse events					
Severe adverse events					
Severe adverse events related to study drug					
Adverse events leading to withdrawal					
Study drug-related adverse events leading to withdrawal					

Note: Related to the study drug is defined as "definite," "possible," and "uncertain".

Table 9.8.3 Coding of adverse event (SOC/PT)

Code	Study Group Incident	Study Group Patients	Study Group N (%)	Placebo Group Incident	Placebo Group Patients	Placebo Group N (%)	P
Adverse event							

Table 9.8.4 Coding of adverse drug reaction (SOC/PT)

Item	Study Group Incident	Study Group Patients	Study Group N (%)	Placebo Group Incident	Placebo Group Patients	Placebo Group N (%)	P
Adverse drug reaction							

Table 9.8.5 Comparison of vital signs (12 months ± 7 days) - systolic blood pressure (mmHg)

Item	Index	Study Group	SS Placebo Group
Baseline systolic blood pressure	N(Missing)		
	Mean(Sd)		
	Median		
	Q1,Q3		
	Min,Max		
	Statistics		
	P		
12-month systolic blood pressure	N(Missing)		
	Mean(SD)		

Item	Index	SS<br>Study Group	Placebo Group
	Median		
	Q1,Q3		
	Min,Max		
Intergroup comparison in 12-month	Statistics		
	P		
Intragroup difference between baseline and 12-month	N(Missing)		
	Mean(SD)		
	Median		
	Q1,Q3		
	Min,Max		
Intragroup comparison	Statistics		
	P		
Intragroup difference comparison	Statistics		
	P		
Rate of change	N(Missing)		
	Mean(SD)		
	Median		
	Q1,Q3		
	Min,Max		
Intragroup comparison of the rate of change	Statistics		
	P		
Intergroupomparison of the rate of change	Statistics		
	P		

Table 9.8.6 Comparison of vital signs (12 months ± 7 days) - diastolic blood pressure (mmHg)

Format same as above

Table 9.8.7 Comparison of vital signs (12 months ± 7 days) - heart rate (bpm)

Format same as above

Table 9.8.8 Comparison of vital signs (12 months ± 7 days) - pluse (bpm)

Format same as above

Table 9.8.9 Comparison of vital signs (12 months ± 7 days) – Body temperature (°C)

Format same as above

Table 9.8.10 Crosstabulation of Complete blood count - white blood cell count (12 months ± 7 days)

		White blood cell count					
Group	Before	Normal	Insignificant abnormal	Significant abnormal	Untested	Missing	Total
Study							
	Normal						
	Insignificant abnormal						
	Significant abnormal						
	Untested						
	Missing						
	Total						
Placebo							
	Normal						
	Insignificant abnormal						
	Significant abnormal						
	Untested						
	Missing						
	Total						

Table 9.8.11 Crosstabulation of Complete blood count - hemoglobin (12 months ± 7 days)

Format same as above

Table 9.8.12 Crosstabulation of Complete blood count - red blood cell count (12 months ± 7 days)

Table 9.8.13 Crosstabulation of Complete blood count - platelets (12 months ± 7 days)

Table 9.8.14 Summary of Complete blood count - 12 months ± 7 days (SS)

		Normal before IPs		Abnormal before IPs	
Index	Group	Normal after IPs	Abnormal after IPs	Normal after IPs	Abnormal after IPs
white blood cell count	Study				
	Placebo				
hemoglobin	Study				
	Placebo				
red blood cell count	Study				
	Placebo				
platelets	Study				
	Placebo				

Table 9.8.15 Crosstabulation of biochemistry - Alanine aminotransferase (ALT) (12 months ± 7 days)

Table 9.8.16 Crosstabulation of biochemistry – Creatinine (Cr) (12 months ± 7 days)

Table 9.8.17 Crosstabulation of biochemistry – Blood Uear Nitrogrn (BUN) (12 months ± 7 days)

Table 9.8.18 Crosstabulation of biochemistry – Total cholesterol (TC) (12 months ± 7 days)

Table 9.8.19 Crosstabulation of biochemistry – Triglyciride (TG) (12 months ± 7 days)

Table 9.8.20 Crosstabulation of biochemistry – Potassium (K) (12 months ± 7 days)

Table 9.8.21 Crosstabulation of biochemistry – Sodium (Na) (12 months ± 7 days)

Table 9.8.22 Crosstabulation of biochemistry – Chlorine (Cl) (12 months ± 7 days)

Table 9.8.23 Crosstabulation of biochemistry – Fasting blood glucose (GLU) (12 months ± 7 days)

Table 9.8.24 Summary of Biochemistry - 12 months ± 7 days (SS)

		Normal before IPs		Abnormal before IPs	
Index	Group	Normal after IPs	Abnormal after IPs	Normal after IPs	Abnormal after IPs
ALT	Study				
	Placebo				
Cr	Study				
	Placebo				
BUN	Study				
	Placebo				
TC	Study				
	Placebo				
TG	Study				
	Placebo				
K	Study				
	Placebo				

Index	Group	Normal before IPs		Abnormal before IPs	
		Normal after IPs	Abnormal after IPs	Normal after IPs	Abnormal after IPs
Na	Study				
	Placebo				
Cl	Study				
	Placebo				
GLU	Study				
	Placebo				

Table 9.8.25 Crosstabulation of Urine routine – Urine protine (12 months ± 7 days)

Table 9.8.26 Crosstabulation of Urine routine – Urine white blood cell (12 months ± 7 days)

Table 9.8.27 Crosstabulation of Urine routine – Urine red white blood cell (12 months ± 7 days)

Table 9.8.28 Summary of Urine routine - 12 months ± 7 days

Index	Group	Normal before IPs		Abnormal before IPs	
		Normal after IPs	Abnormal after IPs	Normal after IPs	Abnormal after IPs
Urine protine	Study				
	Placebo				
Urine white blood cell	Study				
	Placebo				
Urine red blood cell	Study				
	Placebo				

Table 9.8.29 Crosstabulation of Physical examination – General condition (12 months ± 7 days)

Table 9.8.30 Crosstabulation of Physical examination – Nervous system (12 months ± 7 days)

Table 9.8.31 Crosstabulation of Physical examination – Skin, superficial lymph nodes (12 months ± 7 days)

Table 9.8.32 Crosstabulation of Physical examination – head and neck (12 months ± 7 days)

Table 9.8.33 Crosstabulation of Physical examination – Cardiovascular (12 months ± 7 days)

Table 9.8.34 Crosstabulation of Physical examination – Respiratory (12 months ± 7 days)

Table 9.8.35 Crosstabulation of Physical examination – Abdominal (12 months ± 7 days)

Table 9.8.36 Crosstabulation of Physical examination – Genitourinary (12 months ± 7 days)

Table 9.8.37 Crosstabulation of Physical examination – Spine and Limbs (12 months ± 7 days)

Table 9.8.38 Crosstabulation of Physical examination – Other (12 months ± 7 days)

Table 9.8.39 Summary of Urine routine - 12 months ± 7 days

		Normal before IPs		Abnormal before IPs	
Index	Group	Normal after IPs	Abnormal after IPs	Normal after IPs	Abnormal after IPs
General condition	Study				
	Placebo				
Nervous system	Study				
	Placebo				
Skin, superficial lymph nodes	Study				
	Placebo				
Head and neck	Study				
	Placebo				
Cardiovascular	Study				
	Placebo				
Respiratory	Study				
	Placebo				
Abdominal	Study				
	Placebo				
Genitourinary	Study				
	Placebo				
Spine and Limbs	Study				
	Placebo				
Other	Study				
	Placebo				

Table 9.8.40 Crosstabulation of electrocardiogram (12 months ± 7 days)

Table 9.8.41 Summary of electrocardiogram - 12 months ± 7 days

		Normal before IPs		Abnormal before IPs	
Index	Group	Normal after IPs	Abnormal after IPs	Normal after IPs	Abnormal after IPs
Electrocardiogram with clinical significance	Study				
	Placebo				

9.9. List of appendixes

Appendix 1 – Dropout/ Elimination List (all randomized populations)

Center	Random No.	Group	Completion	Dropout Date	Dropout Reason	Detail	Condition	Dropout classification	Specific description	FAS	PPS	SS

Appendix 2 – Demographics (FAS)

Center	Random No.	Group	Date of birth	Age (yrs)	Sex	Marriage	Ethnic	Other	Height (cm)	Weight (kg)	BMI (kg/m^2)	Nationality	Other

Appendix 3 – Concomitant medication

Center	Random No.	Group	W/O Concomitant medication	Drug name (generic name)	Reason for medication	AE No.	Medical history coding	Dosage	Start date	End date	Continued at last follow-up	ATC1 term	ATC2 term

Appendix 4- Medication Compliance List (Dispensation and Use of Study Drugs) (FAS)

Center	Random No.	Group	Drug dispense date	Last visit date	Days of medication	Dosage to be taken (tablets)	Actual dosage be taken (tablets)	Compliance (%)	FAS	PPS

Appendix 5 - List of Primary Effecacy Endpoint

Center	Random No.	Group	Randomized date	MACE Date	Censored MACE	Time (days)	CV death Date	Censored CV death	Time (days)	HHF Date	Censored HHF	Time (days)	FAS	PPSSS

Appendix 6 - List of Secondary Efficacy Endpoint

Center	Random No.	Group	Randomized date	All-cause mortality Date	Censored 1	Time (days)	Composite endpoint (worsening heart failure leading to withdrawal of treatment, successful resuscitation after cardiac arrest, malignant arrhythmia, and non-fatal stroke) Date	Censored 2	Time (days)	Worsening heart failure leading to withdrawal of treatment Date	Censored 3	Time (days)	FAS	PPS

Appendix 6-1 - List of Secondary Efficacy Endpoint - Continuation

Center	Random No.	Group	Randomized date	successful resuscitation after cardiac arrest	Censored 4	Time (days)	Malignant arrhythmia	Censored 5	Time (days)	non-fatal stroke	Censored 6	Time (days)	FAS	PPS

Appendix 7- Serum NT-proBNP

Center	Random No.	Group	Baseline NT-proBNP(pg/ml)	1 month NT-proBNP(pg/ml)	Difference (Basline-1month)	Decrease rate (%)	Decrease rate ≥30%	3 months NT-proBNP(pg/ml)	Difference (Baseline-3months)	Decrease rate (%)	Decrease rate ≥30%	FAS	PPS

Appendix 8 - List of Adverse Events

Center	Random No.	Group	Y/N AE	AE Date	AE Grade	Y/N SAE	Death	Life-threadening	Hospitalization or prolong stay	Causes permanent or significant disability	Congenital malformation	Medical event	Actions Taken on IPs	Whether to treat	Whether to withdraw from the trial due to adverse events	outcome	Relationship to Study Drug	Detailed description of adverse events	Termination/Vestination Date	version number	SOC	PT

Appendix 9 - List of Severe Adverse Events

Center	Random No.	Group	Y/N AE	AE Date	AE Grade	Y/N SAE	Death	Life-threadening	Hospitalization or prolong stay	Causes permanent or significant disability	Congenital malformation	Medical event	Actions Taken on IPs	Whether to treat	Whether to withdraw from the trial due to adverse events	outcome	Relationship to Study Drug	Detailed description of adverse events	Termination/Vestination Date	version number	SOC PT

Appendix 10 – List of abnormoal complete blood count from normal baseline (SS)

Center	Random No.	Index	Group	Before IPs	Clinical significance	After IPs	Clinical significance

Appendix 11 - List of abnormoal complete blood count from abnormal baseline (SS)

Format same as above

Appendix 12 - List of abnormoal biochemistry from normal baseline (SS)

Format same as above

Appendix 13 - List of abnormoal biochemistry from abnormoal baseline (SS)

Format same as above

Appendix 14 - List of abnormoal urine routine from normal baseline (SS)

Format same as above

Appendix 15 - List of abnormoal urine routine from abnormal baseline (SS)

Format same as above

Appendix 16 - List of abnormoal physical examination from normal baseline (SS)

Format same as above

Appendix 17 - List of abnormoal physical examination from abnormal baseline (SS)

Format same as above

Appendix 18 - List of abnormoal electrocardiogram from normal baseline (SS)

Format same as above

Appendix 19 - List of abnormoal electrocardiogram from abnormal baseline (SS)

Format same as above

Appendix 20- Trial Completion List (All Randomized Populations)

Center	Random No.	Group	Completion	Dropout Date	Dropout Reason	Detailed	Y/N Unblinding	Unblinding Date	Elimination Date
								.	
								.	
								.	

Appendix 21- List of Unblinding List (All Randomized Populations)

Center	Random No.	Group	Completion	Randomized Date	Y/N Unblinding	Unblinding Date	Unblinding Date Reason	Death	Date of death

统计分析计划 V1.1 更新

1. 目录和题号更正。
2. 表 9.2.1.：增加了 eGFR 的基线。
3. 表 9.4.4-5：增加了 HF 治疗的基线。
4. 表 9.5.1-3：增加了基于 I 类错误 (95.372% CI) 的主要研究终点分析。
5. 表 9.6.73-123：对感兴趣的亚组分析进行了补充。
6. 语法错误更正。

芪苈强心胶囊对慢性心衰复合终点事件的评估研究

统计分析计划 V1.1

课题承担单位：南京医科大学第一附属医院

药品生产单位：石家庄以岭药业股份有限公司

统计分析单位：北京大学临床研究所

版本日期：2023 年 01 月 10 日

作者：______________________　　　日期：______________________

签署人：____________________　　　日期：______________________

目录

1. 缩略语 9

2. 试验题目 10

3. 试验目的 10

4. 试验总体设计与安排 10

4.1. 随机分组阶段（第 0 天~第 24 个月） 11
4.2. 病例数量、分组、中心 11
4.3. 盲法与随机化 12
4.3.1. 受试者随机分配方法 12
4.3.2. 紧急揭盲 12
4.3.3. 揭盲规定 13
4.3.4. 筛选编号 13
4.4. 治疗方案 13
4.4.1. 研究流程 13
4.4.2. 基础治疗 14
4.4.3. 合并用药 14
4.4.4. 疗程：12 个月-36 个月 14
4.5. 依从性评价 14
4.6. 药物不良反应 14

5. 研究人群 15

5.1. 入选标准 15
5.2. 排除标准 15
5.3. 中止研究药物治疗 16
5.4. 退出标准 16
5.5. 全面中止试验标准 17

6. 临床观察终点和指标 17

6.1. 临床观察指标 17
6.1.1. 主要有效终点 17
6.1.2. 次要有效终点 17
6.2. 安全性指标包括： 17
6.2.1. 不良事件定义 18
6.2.2. 不良事件强度判定标准 18
6.2.3. 不良事件与研究药物关系的判断标准 18
6.2.4. 严重不良事件 19
6.2.5. 不良事件的随访与记录 20
6.2.6. 实验室结果异常 20
6.3. 终点事件的含义及 CEC 判定程序 20
6.3.1. 本研究终点事件的含义 20

6.3.2. 终点事件发生时的评估与程序 *22*

7. 数据管理 **22**

7.1. 数据库设计 23
7.2. 数据录入 23
7.3. 数据质疑管理 23
7.4. 医学编码 23
7.5. 数据审核 23
7.6. 数据库锁定 23

8. 统计分析 **23**

8.1. 分析人群 24
8.2. 统计分析方法 24
8.2.1. 入组及完成情况 *24*
8.2.2. 依从性分析 *25*
8.2.3. 疗效评价 *25*
8.2.4. 安全性评价 *26*
8.3. 期中分析 27

9. 统计分析结果 **28**

9.1. 病例分布（所有随机化人群） 28
表 9.1.1 各中心病例分布 *28*
表 9.1.2 两组脱落剔除情况比较 *28*
表 9.1.3 各中心的人群划分情况描述 *28*
表 9.1.4 入组病例及安全性有效性分析数据集 *29*
9.2. 人口学和基线信息(FAS) 30
表 9.2.1 人口学资料 *30*
表 9.2.2 个人史 *30*
表 9.2.3 疾病情况和既往病史 *31*
表 9.2.4 重要既往病史分析 *32*
表 9.2.5 既往病史分析（SOC/PT） *32*
表 9.2.6 重要病史（PT） *33*
表 9.2.7 NYHA 心功能分级 *33*
表 9.2.8 心脏彩色超声检查 *33*
表 9.2.9 血清 NT-proBNP 检测 *34*
表 9.2.10 生命体征 *34*
表 9.2.11 基线肌酐及 eGFR *35*
9.3. 中位随访时间 35
表 9.3.1 随访时间描述 *35*
表 9.3.2 中位随访时间 *36*
9.4. 用药情况(SS) 36
表 9.4.1 用药依从性 *36*
表 9.4.2 既往用药/合并用药 *36*

表 9.4.3 合并用药编码（ATC1/ATC2） 36
表 9.4.4 螺内酯、ARNi、 SGLT2i 和 强心苷使用情况 37
表 9.4.5 接受三联治疗（RAAS+Beta+MRA）的情况 37
9.5. 主要疗效指标评价（FAS&PPS） 38
表 9.6.1 发生复合终点事件（心血管死亡和心衰恶化再住院）的时间 38
图 1 发生复合终点事件 KM 曲线图（FAS 数据集） 38
图 2 发生复合终点事件 KM 曲线图（PPS 数据集） 38
表 9.6.2 发生心血管死亡的时间 39
图 3 心血管死亡 KM 曲线图（FAS 数据集） 39
图 4 心血管死亡 KM 曲线图（PPS 数据集） 39
表 9.6.3 发生心衰恶化再住院的时间 40
图 5 心衰恶化再住院 KM 曲线图（FAS 数据集） 40
图 6 心衰恶化再住院 KM 曲线图（PPS 数据集） 40
9.6. 主要疗效指标评价-亚组分析 41
表 9.6.1 复合终点事件（心血管死亡和心衰恶化再住院）（≤70 岁） 41
表 9.6.2 复合终点事件（心血管死亡和心衰恶化再住院）（>70 岁） 41
表 9.6.3 发生心血管死亡的时间（≤70 岁） 41
表 9.6.4 发生心血管死亡的时间（>70 岁） 41
表 9.6.5 发生心衰恶化再住院的时间（≤70 岁） 42
表 9.6.6 发生心衰恶化再住院的时间（>70 岁） 42
表 9.6.7 复合终点事件（心血管死亡和心衰恶化再住院）（男性） 42
表 9.6.8 复合终点事件（心血管死亡和心衰恶化再住院）（女性） 42
表 9.6.9 发生心血管死亡的时间（男性） 42
表 9.6.10 发生心血管死亡的时间（女性） 42
表 9.6.11 发生心衰恶化再住院的时间（男性） 42
表 9.6.12 发生心衰恶化再住院的时间（女性） 42
表 9.6.13 复合终点事件（心血管死亡和心衰恶化再住院）（NYHA 分级 I 级或 II 级） 42
表 9.6.14 复合终点事件（心血管死亡和心衰恶化再住院）（NYHA 分级 III 级或Ⅳ级） 42
表 9.6.15 发生心血管死亡的时间（NYHA 分级 I 级或 II 级） 42
表 9.6.16 发生心血管死亡的时间（NYHA 分级 III 级或Ⅳ级） 42
表 9.6.17 发生心衰恶化再住院的时间（NYHA 分级 I 级或 II 级） 42
表 9.6.18 发生心衰恶化再住院的时间（NYHA 分级 III 级或Ⅳ级） 42
表 9.6.19 复合终点事件（心血管死亡和心衰恶化再住院）（LVEF≤30%） 43
表 9.6.20 复合终点事件（心血管死亡和心衰恶化再住院）（LVEF>30%） 43
表 9.6.21 发生心血管死亡的时间（LVEF≤30%） 43
表 9.6.22 发生心血管死亡的时间（LVEF>30%） 43
表 9.6.23 发生心衰恶化再住院的时间（LVEF≤30%） 43
表 9.6.24 发生心衰恶化再住院的时间（LVEF>30%） 43
表 9.6.25 复合终点事件（心血管死亡和心衰恶化再住院）（心衰病程≤3 年） 43
表 9.6.26 复合终点事件（心血管死亡和心衰恶化再住院）（心衰病程>3 年） 43
表 9.6.27 发生心血管死亡的时间（心衰病程≤3 年） 43
表 9.6.28 发生心血管死亡的时间（心衰病程>3 年） 43

表 9.6.29 发生心衰恶化再住院的时间（心衰病程≤3 年）........ 43
表 9.6.30 发生心衰恶化再住院的时间（心衰病程>3 年）........ 43
表 9.6.31 复合终点事件（心血管死亡和心衰恶化再住院）（有冠心病）........ 43
表 9.6.32 复合终点事件（心血管死亡和心衰恶化再住院）（无冠心病）........ 43
表 9.6.33 发生心血管死亡的时间（有冠心病）........ 44
表 9.6.34 发生心血管死亡的时间（无冠心病）........ 44
表 9.6.35 发生心衰恶化再住院的时间（有冠心病）........ 44
表 9.6.36 发生心衰恶化再住院的时间（无冠心病）........ 44
表 9.6.37 复合终点事件（心血管死亡和心衰恶化再住院）（有心肌病）........ 44
表 9.6.38 复合终点事件（心血管死亡和心衰恶化再住院）（无心肌病）........ 44
表 9.6.39 发生心血管死亡的时间（有心肌病）........ 44
表 9.6.40 发生心血管死亡的时间（无心肌病）........ 44
表 9.6.41 发生心衰恶化再住院的时间（有心肌病）........ 44
表 9.6.42 发生心衰恶化再住院的时间（无心肌病）........ 44
表 9.6.43 复合终点事件（心血管死亡和心衰恶化再住院）（有高血压病）........ 44
表 9.6.44 复合终点事件（心血管死亡和心衰恶化再住院）（无高血压病）........ 44
表 9.6.45 发生心血管死亡的时间（有高血压病）........ 44
表 9.6.46 发生心血管死亡的时间（无高血压病）........ 44
表 9.6.47 发生心衰恶化再住院的时间（有高血压病）........ 45
表 9.6.48 发生心衰恶化再住院的时间（无高血压病）........ 45
表 9.6.49 复合终点事件（心血管死亡和心衰恶化再住院）（有糖尿病）........ 45
表 9.6.50 复合终点事件（心血管死亡和心衰恶化再住院（无糖尿病）........ 45
表 9.6.51 发生心血管死亡的时间（有糖尿病）........ 45
表 9.6.52 发生心血管死亡的时间（无糖尿病）........ 45
表 9.6.53 发生心衰恶化再住院的时间（有糖尿病）........ 45
表 9.6.54 发生心衰恶化再住院的时间（无糖尿病）........ 45
表 9.6.55 复合终点事件（心血管死亡和心衰恶化再住院）（有房颤）........ 45
表 9.6.56 复合终点事件（心血管死亡和心衰恶化再住院）（无房颤）........ 45
表 9.6.57 发生心血管死亡的时间（有房颤）........ 45
表 9.6.58 发生心血管死亡的时间（无房颤）........ 45
表 9.6.59 发生心衰恶化再住院的时间（有房颤）........ 45
表 9.6.60 发生心衰恶化再住院的时间（无房颤）........ 45
表 9.6.61 复合终点事件（心血管死亡和心衰恶化再住院）（有心率失常）........ 46
表 9.6.62 复合终点事件（心血管死亡和心衰恶化再住院）（无心率失常）........ 46
表 9.6.63 发生心血管死亡的时间（有心率失常）........ 46
表 9.6.64 发生心血管死亡的时间（无心率失常）........ 46
表 9.6.65 发生心衰恶化再住院的时间（有心率失常）........ 46
表 9.6.66 发生心衰恶化再住院的时间（无心率失常）........ 46
表 9.6.67 复合终点事件（心血管死亡和心衰恶化再住院）（BNP≤中位数）........ 46
表 9.6.68 复合终点事件（心血管死亡和心衰恶化再住院）（BNP>中位数）........ 46
表 9.6.69 发生心血管死亡的时间（BNP≤中位数）........ 46
表 9.6.70 发生心血管死亡的时间（BNP>中位数）........ 46

表 9.6.71 发生心衰恶化再住院的时间（BNP≤中位数） 46
表 9.6.72 发生心衰恶化再住院的时间（BNP>中位数） 46
表 9.6.73 复合终点事件（心血管死亡和心衰恶化再住院）（BMI＜25） 46
表 9.6.74 复合终点事件（心血管死亡和心衰恶化再住院）（BMI≥25） 47
表 9.6.75 复合终点事件（心血管死亡和心衰恶化再住院）（eGFR＜90） 47
表 9.6.76 复合终点事件（心血管死亡和心衰恶化再住院）（eGFR≥90） 47
表 9.6.77 复合终点事件（心血管死亡和心衰恶化再住院）（eGFR 未查） 47
表 9.6.78 复合终点事件（心血管死亡和心衰恶化再住院）（基线使用 ACEI/ARB/ARNi 的受试者） 47
表 9.6.79 复合终点事件（心血管死亡和心衰恶化再住院）（基线未使用 ACEI/ARB/ARNi 的受试者） 47
表 9.6.80 复合终点事件（心血管死亡和心衰恶化再住院）（基线接受 β 受体阻滞剂治疗） 47
表 9.6.81 复合终点事件（心血管死亡和心衰恶化再住院）（基线未接受 β 受体阻滞剂治疗） 47
表 9.6.82 复合终点事件（心血管死亡和心衰恶化再住院）（基线接受 MRA 治疗） 47
表 9.6.83 复合终点事件（心血管死亡和心衰恶化再住院）（基线未接受 MRA 治疗） 47
表 9.6.84 复合终点事件（心血管死亡和心衰恶化再住院）（基线接受 ARNi 治疗） 47
表 9.6.85 复合终点事件（心血管死亡和心衰恶化再住院）（基线未接受 ARNi 治疗） 47
表 9.6.86 复合终点事件（心血管死亡和心衰恶化再住院）（基线有 SGLT2i） 47
表 9.6.87 复合终点事件（心血管死亡和心衰恶化再住院）（基线无 SGLT2i） 47
表 9.6.88 复合终点事件（心血管死亡和心衰恶化再住院）（基线接受三联治疗） 47
表 9.6.89 复合终点事件（心血管死亡和心衰恶化再住院）（基线未接受三联治疗） 48
表 9.6.90 发生心衰恶化再住院的时间（BMI＜25） 48
表 9.6.91 发生心衰恶化再住院的时间（BMI≥25） 48
表 9.6.92 发生心衰恶化再住院的时间（eGFR＜90） 48
表 9.6.93 发生心衰恶化再住院的时间（eGFR≥90） 48
表 9.6.94 发生心衰恶化再住院的时间（eGFR 未查） 48
表 9.6.95 发生心衰恶化再住院的时间（基线使用 ACEI/ARB/ARNi 的受试者） 48
表 9.6.96 发生心衰恶化再住院的时间（基线未使用 ACEI/ARB/ARNi 的受试者） 48
表 9.6.97 发生心衰恶化再住院的时间（基线接受 β 受体阻滞剂治疗） 48
表 9.6.98 发生心衰恶化再住院的时间（基线未接受 β 受体阻滞剂治疗） 48
表 9.6.99 发生心衰恶化再住院的时间（基线接受 MRA 治疗） 48
表 9.6.100 发生心衰恶化再住院的时间（基线未接受 MRA 治疗） 48
表 9.6.101 发生心衰恶化再住院的时间（基线接受 ARNi 治疗） 48
表 9.6.102 发生心衰恶化再住院的时间（基线未接受 ARNi 治疗） 48
表 9.6.103 发生心衰恶化再住院的时间（基线有 SGLT2i） 48
表 9.6.104 发生心衰恶化再住院的时间（基线无 SGLT2i） 49
表 9.6.105 发生心衰恶化再住院的时间（基线接受三联治疗） 49
表 9.6.106 发生心衰恶化再住院的时间（基线未接受三联治疗） 49
表 9.6.107 发生心血管死亡的时间（BMI＜25） 49
表 9.6.108 发生心血管死亡的时间（BMI≥25） 49
表 9.6.109 发生心血管死亡的时间（eGFR＜90） 49
表 9.6.110 发生心血管死亡的时间（eGFR≥90） 49
表 9.6.111 发生心血管死亡的时间（eGFR 未查） 49
表 9.6.112 发生心血管死亡的时间（基线使用 ACEI/ARB/ARNi 的受试者） 49

表 9.6.113　发生心血管死亡的时间（基线未使用 ACEI/ARB/ARNi 的受试者） 49
表 9.6.114　发生心血管死亡的时间（基线接受 β 受体阻滞剂治疗） 49
表 9.6.115　发生心血管死亡的时间（基线未接受 β 受体阻滞剂治疗） 49
表 9.6.116　发生心血管死亡的时间（基线接受 MRA 治疗） 49
表 9.6.117　发生心血管死亡的时间（基线未接受 MRA 治疗） 49
表 9.6.118　发生心血管死亡的时间（基线接受 ARNi 治疗） 50
表 9.6.119　发生心血管死亡的时间（基线未接受 ARNi 治疗） 50
表 9.6.120　发生心血管死亡的时间（基线有 SGLT2i） 50
表 9.6.121　发生心血管死亡的时间（基线无 SGLT2i） 50
表 9.6.122　发生心血管死亡的时间（基线接受三联治疗） 50
表 9.6.123　发生心血管死亡的时间（基线未接受三联治疗） 50
9.7. 次要疗效指标评价（FAS&PPS） 50
表 9.7.1　全因死亡率 50
表 9.7.2　复合终点事件（心衰恶化放弃治疗、心脏骤停后复苏成功、恶性心律失常、非致死性卒中）率 50
表 9.7.3　心衰恶化放弃治疗率 51
表 9.7.4　心脏骤停后复苏成功率 51
表 9.7.5　恶性心律失常率 52
表 9.7.6　非致死性卒中率 52
表 9.7.7　冠心病心衰患者的心血管死亡和心衰恶化再住院 53
表 9.7.8　血清 NT-proBNP 较基线变化情况（1 个月） 53
表 9.7.9　血清 NT-proBNP 较基线变化情况（3 个月） 54
表 9.7.10　血清 NT-proBNP 较基线下降率（1 个月） 54
表 9.7.11　血清 NT-proBNP 较基线下降率（3 个月） 54
9.8. 安全性评价（SS） 54
表 9.8.1　不良事件汇总比较 54
表 9.8.2　研究期间不良事件汇总比较 55
表 9.8.3　不良事件编码情况（SOC/PT） 55
表 9.8.4　不良反应编码情况（SOC/PT） 55
表 9.8.5　生命体征前后比较(12 个月±7 天)-收缩压（mmHg） 55
表 9.8.6　生命体征前后比较(12 个月±7 天)-舒张压（mmHg） 56
表 9.8.7　生命体征前后比较(12 个月±7 天)-心率（次/分） 56
表 9.8.8　生命体征前后比较(12 个月±7 天)-脉搏（次/分） 56
表 9.8.9　生命体征前后比较(12 个月±7 天)-体温（℃） 56
表 9.8.10　血常规-12 个月±7 天白细胞总数前后交叉表 57
表 9.8.11　血常规-12 个月±7 天血红蛋白前后交叉表 57
表 9.8.12　血常规-12 个月±7 天红细胞总数前后交叉表 57
表 9.8.13　血常规-12 个月±7 天血小板前后交叉表 57
表 9.8.14　血常规-12 个月±7 天汇总表（SS） 57
表 9.8.15　血生化-12 个月±7 天谷丙转氨酶前后交叉表 58
表 9.8.16　血生化-12 个月±7 天肌酐前后交叉表 58
表 9.8.17　血生化-12 个月±7 天尿素氮前后交叉表 58
表 9.8.18　血生化-12 个月±7 天总胆固醇前后交叉表 58

表 9.8.19　血生化-12 个月±7 天甘油三酯前后交叉表 58
表 9.8.20　血生化-12 个月±7 天钾前后交叉表 58
表 9.8.21　血生化-12 个月±7 天钠前后交叉表 58
表 9.8.22　血生化-12 个月±7 天氯前后交叉表 58
表 9.8.23　血生化-12 个月±7 天空腹血糖前后交叉表 58
表 9.8.24　血生化-12 个月±7 天汇总表 58
表 9.8.25　尿常规-12 个月±7 天尿蛋白前后交叉表 59
表 9.8.26　尿常规-12 个月±7 天尿白细胞前后交叉表 59
表 9.8.27　尿常规-12 个月±7 天尿红细胞前后交叉表 59
表 9.8.28　尿常规-12 个月±7 天汇总表 59
表 9.8.29　体格检查-12 个月±7 天一般情况前后交叉表 59
表 9.8.30　体格检查-12 个月±7 天神经系统前后交叉表 59
表 9.8.31　体格检查-12 个月±7 天皮肤、浅表淋巴结前后交叉表 59
表 9.8.32　体格检查-12 个月±7 天头颈部前后交叉表 60
表 9.8.33　体格检查-12 个月±7 天心脏前后交叉表 60
表 9.8.34　体格检查-12 个月±7 天肺部前后交叉表 60
表 9.8.35　体格检查-12 个月±7 天腹部前后交叉表 60
表 9.8.36　体格检查-12 个月±7 天泌尿生殖系统前后交叉表 60
表 9.8.37　体格检查-12 个月±7 天脊柱四肢前后交叉表 60
表 9.8.38　体格检查-12 个月±7 天其他前后交叉表 60
表 9.8.39　体格检查-12 个月±7 天汇总表 60
表 9.8.40　心电图-12 个月±7 天前后交叉表 61
表 9.8.41　心电图-12 个月±7 天汇总表 61
9.9.　清单 62

1. 缩略语

ACEI	血管紧张素转换酶抑制剂
ADR	可疑药物不良反应
AEs	不良事件
AHA	美国心脏学会
AMI	急性心肌梗死
ARB	血管紧张素受体拮抗剂
ARNI	血管紧张素受体脑啡肽酶抑制剂
BNP	B 型利钠肽
CEC	事件判定委员会
CGRP	降钙素基因相关肽
CHF	慢性心力衰竭
CRA	临床研究监查员
CRC	临床研究协调员
CRF	病历报告表
CV	心血管
DSMB	数据与安全监察委员会
DVP	数据核查计划
ECG	心电图
EF	射血分数
EOS	终末访视
ESC	欧洲心脏病学会
ET	内皮素
FAS	全分析集
HF	心力衰竭
LVOT	左心室流出量
MedDRA	ICH 国际医学用语词典

MI	心肌梗死
NO	一氧化氮
NT-proBNP	氨基末端 B 型利钠肽前体
NYHA	纽约心脏病学会
PPS	符合方案集
RAAS	肾素-血管紧张素-醛固酮系统
SAE	严重不良事件
SNS	交感神经系统
SS	安全性分析集
TVI	时间流速积分
UNS	计划外访视

2. 试验题目

芪苈强心胶囊对慢性心衰复合终点事件的评估研究

3. 试验目的

采用循证医学研究方法，以心血管死亡率和心衰恶化再住院发生率为主要研究终点，进一步阐明芪苈强心胶囊长期用药的临床疗效及安全性，明确疗效特点及适宜人群，为优化临床合理用药方案提供高质量临床证据。

4. 试验总体设计与安排

本研究是一项在慢性心衰患者中进行的随机、双盲、安慰剂对照、平行分组的多中心临床研究。

本研究将为事件驱动型，全部随机入组的患者将保留在研究之中（无论是否服用研究药物），直至主要终点事件的发生数目达到预计（620 例），或者当满足事先定义的提前终止的疗效或安全性标准时，研究提前终止。

计划在发生 1/2、2/3 主要终点事件后进行两次期中疗效分析，以评估是否已得出无效或有效的结论而提前终止该研究。

计划整个研究将持续大约 36 个月，招募期预计 24 个月，最后一例患者入组研究后的随访期为 12 个月。预计平均随访时间约为 24 个月。

在医院开始筛选患者，临床症状稳定，入选之前已接受至少 2 周标准化方案治疗并治疗其他伴随疾病。根据当地 HF 治疗指南规定用药，药物种类、剂量固定，除非禁忌或不耐受，且此期间未静脉用药，未服用与芪苈强心胶囊成分相似中药、中成药的患者直接进入随机分组阶段。

若达不到上述要求，则可先行标准化治疗达到上述标准后再进入随机分组阶段。

4.1. 随机分组阶段（第 0 天~第 24 个月）

*请注意：*接受 2 周以上的标准化治疗方案且未使用中药治疗符合入选标准的受试者进入随机分组阶段。此期间的每一位患者使用药物种类、剂量需要固定。若属医疗需要调整用药，需记录在病例报告表中。

患者将按照 1:1 的比例随机化到试验组或对照组。患者将在当前慢性心衰标准化治疗的基础上使用研究药物。

试验组：慢性心衰标准化治疗+芪苈强心胶囊（4 粒/次，3 次/日，口服）;

对照组：慢性心衰标准化治疗+芪苈强心胶囊安慰剂（4 粒/次，3 次/日，口服）。

治疗期间应避免使用其他中药或中成药（与芪苈强心功能组成相似的中药）

患者应于随机分组后第 1 个月、第 3 个月、第 6 个月，第 9 个月，第 12 个月，此后每隔 3 个月来医院访视，进行有效性和安全性评估，直到研究全部结束。随机分组阶段共 24 个月。

4.2. 病例数量、分组、中心

参考 PARADIGM-HF 研究，中位随访 27 个月 LCZ696 组患者的心血管死亡或心衰住院率为 21.8%，而依那普利组为 26.5%。所以我们估算基础治疗+安慰剂组随访 36 个月内，所有患者的心血管死亡和心衰住院事件发生率为 25%，基础治疗+芪苈强心组发生率为 20%。

试验组与对照组的随机分配比例为 1:1，考虑到期中分析对 I 类错误的消耗，α 调整为单侧 0.02314。样本量为复合终点事件的发生例数。预计需要观察到 620 例复合终点事件，才能提供 80%的把握度（β=0.2），经过 log-rank 检验得到试验组可以降低 20%风险的结论。

假设随访期 36 个月内对照组复合终点事件的发生率为 25%，整个试验持续大约 36 个月，招募期预计 24 个月，则预计需要入组 3080 例（每组 1540 例）受试者才可获得 620 个终点事件。

因此本研究计划纳入 3080 例患者，患者将以患者将以 1:1 的比例分配至试验组与对照组，并计划在约 100 个中心进行。

4.3. 盲法与随机化

4.3.1. 受试者随机分配方法

由北京大学临床研究所统计专业人员，在计算机上用 SAS9.4 统计软件包，按试验组与对照组 1:1 的比例用区组随机化方法生成随机编号。根据此随机编号由与本试验无关的人员对研究药物（芪苈强心胶囊或安慰剂）进行包装编码。

本研究采用随机化与试验药物管理系统（RTSM）, 统计专业人员将向RTSM提供随机编号列表。然后，由RTSM给患者分配随机编号。

在完成基线评估后，在基线访视通过 RTSM 分配随机编号。此后，按照访视计划通过 RTSM 获取药物编号，每次分配的药物编号均不相同，但药物是同一种。在患者随机分组前，研究者必须先登录 RTSM，并且提供一些信息（例如受试者出生日期、性别等）。

4.3.2. 紧急揭盲

如果发生不良事件，只有在必须了解研究药物的使用才能治疗患者的特殊情况下，才能进行紧急揭盲。一旦决定揭盲，研究者必须记录日期、时间和破盲的原因。

研究者需要登录 RTSM 填写破盲申请，由主要研究者审核后再由破盲员破盲。一旦破盲，该病例将中止研究，作为退出处理。

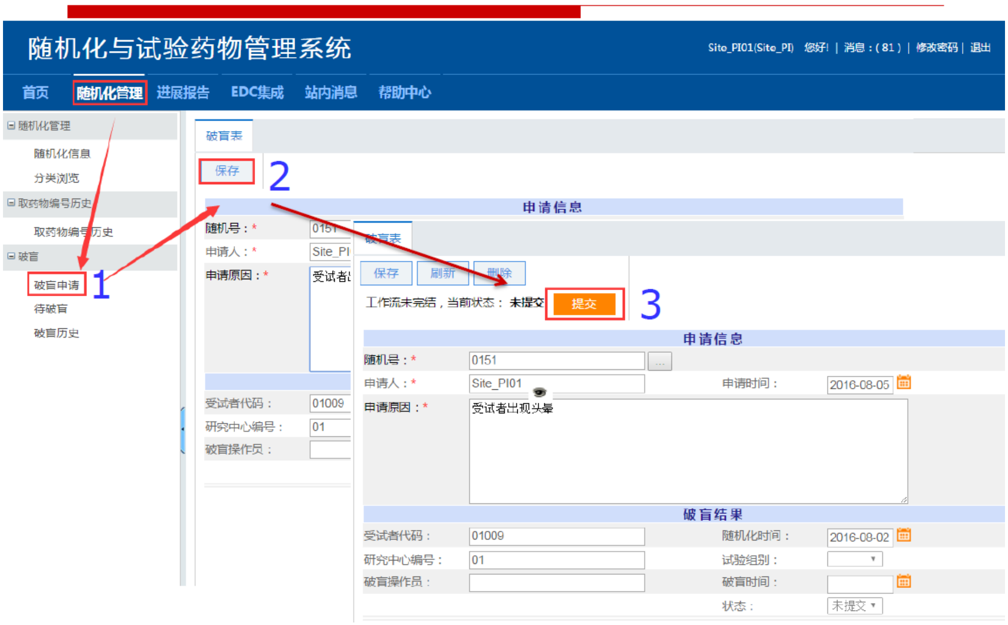

4.3.3. 揭盲规定

本研究采用二次揭盲法。在经盲态核查后，数据锁定，由主要研究者、医学统计专家、数据管理员、申办单位代表进行第一次揭盲，将各随机号所对应的组别以 A，B 为代号标出，以便对全部数据进行统计分析。当统计分析结束，统计报告完成时，再进行第二次揭盲，宣布 A，B 两组的确切组别。

4.3.4. 筛选编号

各医院按收治患者的先后顺序编排筛选号，筛选号由中心编号+3 位整数表示，如，01 中心筛选号 01001，01002，……。

4.4. 治疗方案

4.4.1. 研究流程

入组期（第-14 天~第 0 天）：

患者如果符合本方案中标准治疗的规定，可在此期间完成所有检查，符合入选、排除标准的患者进入随机分组阶段。

随机治疗期（第 0 天~12 个月（最长 36 个月））：（以发药时间记为 0 天）

试验组：慢性心衰标准化治疗+芪苈强心胶囊（4 粒/次 3 次/日 口服）

对照组：慢性心衰标准化治疗+芪苈强心胶囊模拟剂（4 粒/次 3 次/日 口服）

研究药物推荐于每日三餐后约 30 分钟时服用。访视日早晨勿服用药物。如果患者某日未服药，次日服药剂量不得超过日剂量，本研究不允许进行剂量调整。如果患者出现

无法耐受的不良事件，并且根据研究者考虑与研究药物相关，则患者应当终止研究药物治疗

4.4.2. 基础治疗

参照当地 HF 治疗指南或中华医学会心血管病学分会发布的"中国心力衰竭诊断和治疗指南 2018"规定如下：

1) 入选前至少 2 周以上未静脉使用利尿剂、强心剂及血管扩张剂。
2) 进入随机分组阶段至少 2 周前，患者应当接受慢性心衰标准化治疗，所有药物已经调整至固定剂量，标准化药物治疗包括：血管紧张素转换酶抑制剂（ACEI）或血管紧张素受体拮抗剂（ARB）或血管紧张素受体脑啡肽酶抑制剂(ARNI)、β 受体阻滞剂及醛固酮受体拮抗剂（除非禁忌或不耐受，应达到最佳治疗剂量）；

进入治疗期后每一位患者使用药物种类、剂量需延续入选前标准化治疗方案。整个治疗期间原则上不能再调整；若属医疗需要调整用药，需记录在病例报告表中，增加或减少药物剂量、种类的患者需记录复合终点事件和不良事件。

4.4.3. 合并用药

1) 接受较好地控制高血压、心绞痛、糖尿病或其他疾病的药物治疗。
2) 进入随机分组阶段之后整个治疗期均不得使用研究药物以外的其他与试验药物成分类似中药。
3) 患者应当接受有利于心脏健康的饮食指导，如低盐饮食，适量饮水等，患者同时应当接受诸如监测体重、体育锻炼、戒烟、戒酒等适当生活方式改善的咨询。
4) 目前尚未发现禁止与芪苈强心胶囊伴随使用的药物。

4.4.4. 疗程：12 个月-36 个月

4.5. 依从性评价

通过完整记录药物的分发和回收情况来对受试者进行依从性评价，实际服用药物量在应用药物量的 80%~120%范围内，可判定为用药依从性符合方案要求。

4.6. 药物不良反应

目前，尚未发现芪苈强心胶囊存在明显的不良反应。

5. 研究人群

入组患者必须满足下文所列的所有入选标准，并且不符合任何一项排除标准。除下文所列标准外，接受标准化治疗期间，如果存在任何禁忌的医学状况或使用禁忌药物，也是排除患者入选的标准。

5.1. 入选标准

1) 自愿参加，理解并签署知情同意书；
2) 年龄≥18 岁，性别不限；
3) 有 3 个月以上的慢性心衰病史或临床发现心衰症状 3 个月以上；慢性心衰诊断参照当地指南或中华医学会心血管病学分会发布的“中国心力衰竭诊断和治疗指南 2018”；
4) 心脏彩超检查提示左室射血分数（LVEF）≤40％（改良辛普森法）；
5) NYHA 心功能分级II～III，临床症状稳定，包括入选前 2 周内曾诊断为IV级者；
6) 血清 NT-proBNP 含量≥ 450pg/ml；
7) 至少已接受 2 周以上的标准化药物治疗，且未调整过给药剂量及给予静脉治疗者；

标准化药物治疗包括：血管紧张素转换酶抑制剂（ACEI）或血管紧张素受体拮抗剂（ARB）或血管紧张素受体脑啡肽酶抑制剂(ARNI)、β 受体阻滞剂及醛固酮受体拮抗剂（除非禁忌或不耐受，应达到最佳治疗剂量）

5.2. 排除标准

1) 不符合入选标准；
2) 由于瓣膜病、先天性心脏病、心包疾病、心律失常及非心源性病因所致心衰，或肝、肾等重要脏器功能衰竭导致的心衰；及有明确肺源性或其他原因所致的右心衰、及急性心衰；
3) 计划于近期内行冠脉血运重建治疗者或心脏再同步化治疗者，已实施心脏再同步化治疗者；
4) 合并肝、肾、造血系统等严重原发性疾病，肾功能异常者，肝脏转氨酶、碱性磷酸酶超出 3 倍正常值上限，血肌酐＞2mg/dl(176.82umol/L)，血钾＞5.5mmol/L；肿瘤患者，严重神经内分泌系统疾病及精神病患者；
5) 存在左室流出道梗阻、心肌炎、大动脉瘤、夹层动脉瘤、致明显血液动力学改变的未修补的心脏瓣膜病患者；

6) 存在心源性休克、难以控制的恶性心律失常、二度II型以上未置入起搏器治疗的窦房或房室传导阻滞、进行性加重的不稳定心绞痛或急性心肌梗死者；
7) 未获控制的高血压患者，收缩压≥180/mmHg 和/或舒张压≥110mmHg；收缩压<90mmHg 和/或舒张压<60mmHg；
8) 1 个月内参加其他药物临床研究者；
9) 妊娠或正准备妊娠及哺乳期妇女；
10) 过敏体质者，或已知对治疗药物过敏者；
11) 根据研究者判断，患者不能完成本研究或不能遵守本研究的要求（由于管理方面的原因或其它原因）。

5.3. 中止研究药物治疗

随机分组后，任何原因暂停研究药物不等于永久停用，也不应该导致患者退出整个研究。相反对于已经停止服用研究药物的患者，也应该参加所有方案规定的研究访视和评价项目。如果患者不能参加研究访视，应按照计划通过电话继续随访，以确定是否发生任何不良事件和终点事件，除非患者拒绝随访并撤回知情同意书。

出现以下情况时可中止研究药物治疗：

1. 患者可在任何时间中止治疗
2. 发生与研究药物明确相关的过敏反应
3. 发生与研究药物明确相关的不良症状或体征、异常检查结果，研究者判断须终止研究的情况
4. 女性于研究期间发生妊娠

试验过程中应尽可能使患者长期服用标准剂量研究药物，中止研究药物患者在排除相关原因后应尽早恢复服用研究药物并按计划进行随访。

5.4. 退出标准

所有填写了知情同意书并筛选合格进入试验的受试者，无论何时何因退出，均不会影响其后续治疗。

患者有权在任何时间以任何理由退出研究，但应当尽量避免不必要的患者退出，并积极采取措施，尽可能完成随访，以备对其疗效和安全性进行分析。但当患者决定退出时，研究者应当通过电话或个人访问形式联系患者或其责任亲属并尽可能确认退出原因，研究者

应当在患者退出时回收剩余药物，完成最终评估，尽可能完成病例报告、解释退出原因，对退出患者发生终点事件进行随访。如果患者退出的原因为不良事件，则应记录于 CRF 内。

5.5. 全面中止试验标准

1) 研究进行中由于以下原因整个试验在多中心全面停止：

- 基于 DSMB 中期分析结果建议；
- 研究者发现严重安全性问题；
- 方案有重大失误；
- 资助方因经费或管理原因；
- 行政主管部门撤消试验，均可中途停止全部试验。

2) 全面中止试验可是暂时的，也可是永久的。中止试验时，全部试验记录应予保留备查。

6. 临床观察终点和指标

6.1. 临床观察指标

6.1.1. 主要有效终点

- 心血管死亡和心衰恶化再住院组成的复合终点事件发生率；

6.1.2. 次要有效终点

- 全因死亡率
- 复合终点事件（心衰恶化放弃治疗、心脏骤停后复苏成功、恶性心律失常、非致死性卒中）
- 冠心病心衰患者的心血管死亡和心衰恶化再住院发生率
- 血清 NT-proBNP 下降率

注：全部终点事件需经事件判定委员会复核判定。

6.2. 安全性指标包括：

- 不良事件评价
- 临床实验室指标：血常规（血红蛋白、红细胞、白细胞、血小板）、尿常规（尿蛋白、尿白细胞、尿红细胞）、血清生化（尿素氮、肌酐、谷丙转氨酶、空腹血糖、钾、钠、氯、总胆固醇、甘油三酯）。

- 12 导联心电图
- 体格检查

6.2.1. 不良事件定义

- 不良事件（**AEs**）：自受试者签署知情同意书并入选试验后开始至最后一次随访之间，发生任何不利医疗事件，无论与试验药物是否有因果关系，均判定为不良事件。
- 重要不良事件：除严重不良事件外，发生的任何导致针对性医疗措施（如停药，降低剂量和对症治疗）的不良事件和血液学和/或其他实验室异常。

6.2.2. 不良事件强度判定标准

在本临床研究中发生的所有临床不良事件将记录在 CRF 不良事件页上。并将不良事件严重程度进行分级。为统一标准，事件严重程度分级如下：

*严重程度分级：

1 级　轻度，无临床症状或有轻微临床症状；仅临床或实验室检查异常；不需治疗。

2 级　中度，需要微量的、局部的或非侵害性的治疗；与年龄相符的使用工具的日常生活活动[a]受限，使用工具的日常生活指做饭、购物、打电话等。

3 级　病情重或有医学上严重的症状但是暂时不会危及生命；导致住院或住院时间延长；导致残疾；日常生活自理[b]受限。日常生活自理指：洗澡、穿衣、脱衣、吃饭、去卫生间、吃药等，非卧床不起。

4 级　危及生命，需要紧急治疗。

5 级　因不良事件致死。

a：工具性日常生活活动是指做饭，购买日常用品或衣服，使用电话，理财等。

b：自理性日常生活活动是指洗澡，穿/脱衣，吃饭，盥洗，服药，并未卧床不起。

6.2.3. 不良事件与研究药物关系的判断标准

对所有不良事件与试验药物关系的因果分析，均按肯定有关、可能有关、可能无关、肯定无关、无法判定五级进行判断，对前三种定为药物的不良反应。因果分析的考虑因素有以下五个方面：

1) 肯定有关：AE 的发生和试验药物的使用有合理的时间顺序，AE 为试验药物已知的不良反应，停药后 AE 减轻或消失，再次用药重复出现，并无法用受试者本身疾病来解释。

2) 可能有关：AE 的发生和试验药物的使用有合理的时间顺序，AE 为试验药物已知或疑似的不良反应，但是，有其他因素可能引起该事件，如疾病、合并用药等；试验药物停用后反应减轻或消失，或药物停用后的效果不清楚，不清晰或缺乏决定性的信息。
3) 可能无关：AE 的发生和试验药物的使用有合理的时间顺序，但该事件不属于已知的药物不良反应类型，并极可能由受试者疾病或其他治疗引起。
4) 肯定无关：AE 的发生和试验药物的使用无合理的时间顺序，如事件在试验药物使用前已发生；不属于已知的药物不良反应；或 AE 确由其他因素导致，例如：受试者疾病、其他治疗或者合并用药引起等。
5) 无法判定：AE 出现的时间与用药的时间顺序无明确关系，不良事件与试验药物已知的反应类型相似，同时使用的其他药物也可能引起相同的反应，没有足够的依据判断。

以"肯定有关"、"可能有关"、"无法判定"三者合计为试验药物的不良反应，并据此计算不良反应发生率。

6.2.4. 严重不良事件

（1） 一般严重不良事件定义

严重不良事件是指任何提示显著危害、禁忌症、副作用或者需谨慎的临床事件。不良事件符合下面一条或以上标准时归为严重不良事件：

- 死亡
- 有生命危险（指出现该事件的患者在事件发生当时存在立即死亡的风险；并不包括那些如果更加严重将有可能导致患者死亡的事件）
- 导致住院或住院时间延长
- 导致持久或显著的劳动力丧失或残疾
- 先天性畸形缺陷

有些还没有导致死亡、生命危险或需住院的医疗事件，经过适当的医学判断，认为其可能对病人或受试者造成危害或需药物或外科手术治疗以避免上述情况发生时，也应视为 SAE。

（2） 严重不良事件的研究特异性定义

在本试验中，下列事件将不会作为严重不良事件报告，除非被判定阴性并且研究者认为与研究用药相关

◆ 心血管死亡

- ◆ 心衰恶化再住院
- ◆ 心衰恶化放弃治疗
- ◆ 心脏骤停后复苏成功
- ◆ 恶性心律失常
- ◆ 非致死性卒中

但是，所有其他导致致死性结局的事件都将被作为严重不良事件报告。

6.2.5. 不良事件的随访与记录

出现的不良事件，尤其是那些与试验药物相关的事件应当随访直至它们恢复至基线状态或者趋于稳定。如果经过随访，仍无法恢复基线状态或者稳定，那么应当在 CRF 中记录说明。临床试验过程中的任何严重不良事件，必须在 24 小时内报告临床试验监查人员、主要研究单位、药品生产企业。同时研究者必须填写严重不良反应表，记录严重不良事件的发生时间、严重程度、持续时间、采取的措施和转归。

6.2.6. 实验室结果异常

研究者应当对实验室结果异常是否具有临床意义进行判断，并给出可能的解释。已经被报告的不良事件导致的实验室异常结果应同时作为不良事件记录在不良事件表中。具有临床意义的实验室检查异常满足以下一项或多项条件者，应作为独立诊断记录在 CRF 的不良事件页中（不包括已被报告的不良事件导致的实验室结果异常）：

- 伴有临床症状的
- 导致研究用药改变的
- 需要改变合并用药和（或）其他治疗措施的

6.3. 终点事件的含义及 CEC 判定程序

6.3.1. 本研究终点事件的含义

- **心力衰竭住院是指满足下列所有标准的事件：**

1.患者因初步诊断为 HF 住院

2.患者住院时间延长至少 24 小时（或者如果不能获得住院时间和出院时间，指在日历上日期发生的变化）

3.在患者报告上记录由于 HF 出现新症状或者恶化症状，至少包括以下情况之一：

a.呼吸困难（用力时呼吸困难、休息时呼吸困难、端坐呼吸、夜间阵发性呼吸困难）

b.运动耐量减少

c.疲劳

4.患者具有新出现的恶化 HF 的客观证据，包括至少两种体检结果或一种体检结果和至少一种实验室标准，包括：

a.判断由 HF 导致的体检结果，包括新出现或恶化的：

1)外周性水肿。 2)腹胀或腹水增加（在无原发性肝病的情况下）。 3)肺啰音/爆裂音/湿性啰音。 4)颈静脉压升高和/或肝颈静脉回流。 5)S3 奔马律。 6)具有临床意义的或迅速的体重增加，考虑与体液潴留有关

b.在 24 小时内获得的，新出现或者恶化 HF 的实验室证据，包括：

1)与 HF 失代偿一致（如 BNP>500pg/ml 或 NT-proBNP>2000pg/ml）的 B 型利钠肽（BNP）/N 末端 B 型利钠肽前体（NT-proBNP）浓度增加。在利钠肽长期升高的患者中，应特别关注超过基线的显著增加。 2)肺充血的放射影像学证据。3)具有临床意义的左侧或者右侧心室充盈压升高或者心输出量降低的非侵害性诊断证据。如超声心动图标准包括：E/e´>15 或者 D 主导肺静脉流入模式，充血性下腔静脉伴有极小程度吸气塌陷，或者左心室流出量（LVOT）微小行程距离减小（时间流速积分（TVI））。4)侵入性诊断证据：右心导管检查显示肺毛细血管楔压（肺动脉闭塞压）≧18mmHg，中央静脉压≧12mmHg，或心排血量指数＜$2.2L/min/m^2$。

注：如适用，即使不满足上述标准，仍需报告诊断检查中的所有结果，为上述事件的裁定提供重要信息。

5.患者接受针对 HF 的初期或者强化治疗，包括下列至少一种：

a.增强口服利尿药的治疗

b.静脉注射利尿药或者血管活性药物（如正性肌力药、血管升压类药物或者血管扩张剂）

c.机械或手术干预，包括：

1)机械循环支持（例如，主动脉球囊反搏、心室辅助装置、体外膜氧合、全人工心脏）

2)机械辅助去除体液（例如，超滤、血液滤过、透析）

- **心血管死亡：**包括急性心肌梗死（MI），心源性猝死，心力衰竭（HF）导致的死亡，中风导致的死亡，心血管（CV）手术导致的死亡，CV 出血以及其他 CV 原因造成的死亡。
- 全因死亡
- **心衰恶化放弃治疗：**心衰症状和体征不断加重，需要静脉药物或机械支持治疗而患者或患者家属主动放弃治疗或自动出院，若随访其后果为死亡则列入心力衰竭死亡。
- 心脏骤停后复苏成功
- **恶性心律失常：**对于恶性心律失常的定义， 目前还没有统一的标准，一般是指能在短时间内引起严重血流动力学障碍，导致患者晕厥甚至猝死的心律失常 。根据这个标准，恶性心律失常主要有如下类别：(1) 严重的缓慢型心律失常， 如严重的病态窦房结综合征、高度或三度房室传导阻滞；(2) 快速型心律失常， 如持续性室性心动过速、心室扑动、心室颤动， 快室率心房扑动、心房颤动、房室折返性心动过速、预激综合征伴心房颤动、窦性心动过速等。
- 非致死性卒中

6.3.2. 终点事件发生时的评估与程序

研究者获知终点事件发生后，应在 7 天内收集相关支持文件报告事件判定委员会。终点事件将由独立的事件判定委员会（CEC）进行复核，因此终点事件报告表将作为 CRF 的一部分，研究者将在上述表格内记录事件并及时提交支持文件（入院与出院记录、病历记录、死亡记录、ECG 等）上述资料将提供给 CEC 以对事件进行判定。

CEC 由主席及 5-6 名成员组成，每一例事件将由委员会的两位成员进行独立审查并将结论提交至委员会主席处。如果两位审查委员之间或两位审查委员与主席的意见不一致，当具有异议的事件积累到一定数量时，整个委员会将安排会议对事件进行审查。

7. 数据管理

本研究采用 Epidata 软件或 EDC 系统进行研究数据的采集。数据管理确保临床试验数据的真实性、完整性和准确性，数据管理过程需符合《药物临床试验质量管理规范》、《临床试验数据管理工作技术指南》等法规要求，保证临床试验数据的可溯源性。以下列出数据管理的主要流程。

7.1. 数据库设计

数据管理员根据 CRF 采用 Epidata 软件或 EDC 系统设计数据库，经测试后发布。

7.2. 数据录入

CRC 负责将 CRF 中的数据录入数据库，数据录入采用二次录入方式，由两名 CRC 分别录入一遍数据，数据管理员对两个数据库进行比对，产生数据不一致清单，CRC 按照清单对照 CRF 分别修改各自的数据库，然后再进行比对，重复以上步骤，直至两个数据库完全一致。

7.3. 数据质疑管理

数据管理员依据数据核查计划（DVP）编写数据核查 SAS 程序对数据进行核查，产生数据质疑清单，经人工核对后，生成数据质疑表，由 CRA 交研究者进行答疑，答疑后的质疑表再由 CRA 返还给数据管理员，数据管理员据此修订数据库。

7.4. 医学编码

不良事件编码采用 MedDRA21.0 或者更新版本。

7.5. 数据审核

数据库清理完成后，数据管理员撰写《数据核查报告》，用于召开数据核查会议。

审核报告重点记录内容为：入组病例数、脱落、剔除病例情况、偏离或违背方案情况、依从性数据，合并用药，不良事件，与评价指标有关的数据等。

数据审核会议上，针对审核报告的内容，讨论并确定统计人群的划分。

7.6. 数据库锁定

完成数据库锁库清单，依据数据库锁定程序完成数据库锁定。数据锁定之后发现的问题，经确认后可在统计分析程序中修正。数据锁定后如有确切证据证明有必要解锁，研究者及相关人员需签署解锁文件。

数据库锁定后，由数据管理员导出 SAS 格式的数据文件，交与统计人员进行统计分析。

8. 统计分析

试验方案确定后，由统计专业人员负责与主要研究者协商制订统计分析计划书。统计分析软件采用 SAS®9.4 软件（或更高版本）。样本量计算软件采用 PASS13。

8.1. 分析人群

研究人群分为以下几类：

- 全分析集(FAS)：是指尽可能接近意向性分析原则（intention to treat）、从所有随机化的受试者中，以最少的和合理的方法剔除受试者后得出的数据集，包含所有经过随机化并使用过一次研究药物的受试者。剔除通常包括：违反重要入组标准；受试者未接受试验用药物的治疗；随机化后无任何观测数据。主要疗效评价指标为发生复合终点事件的时间，采用生存分析的方法进行分析，在选择 FAS 进行统计分析时，对于主要终点事件的缺失按照删失处理。
- 符合方案集(PPS)：是全分析集的一个子集，这些受试者对方案更具依从性。纳入 PPS 受试者一般具有以下特征：（1）完成事先设定的试验药物的最小暴露量，即服用药物的依从性达到 80%；（2）试验中主要指标的数据均可以获得；（3）未对试验方案有重大的违背。
- 安全性分析集（SS）：所有随机化后至少接受一次治疗且有安全性评价的受试者。安全性缺失值无需结转。

疗效分析将在 FAS 和 PPS 的基础上进行。所有基线人口统计学资料分析将在 FAS 的基础上进行，安全性评价将在 SS 上进行

8.2. 统计分析方法

- 所有的统计检验均采用双侧检验，*P* 值小于或等于 0.05 将被认为所检验的差别有统计意义。(特别说明的除外）
- 描述性分析：分类指标描述各类的例数及百分数。定量指标采用均数、标准差、最大值、最小值、中位数、下四分位数（Q1）和上四分位数（Q3）描述。
- 对两组一般情况的比较将根据指标的类型采用适当的方法进行分析，定量资料的组间比较采用成组 t 检验或 Wilcoxon 秩和检验，分类数据采用卡方检验或精确概率法，等级资料采用 Wilcoxon 秩和检验或 CMH 检验。

8.2.1. 入组及完成情况

总结各中心入组及完成数，列出脱落病例的清单。各组不同数据集大小，各中心病例分布，总脱落率比较，终止原因详细列表。对患者的人口学特征(年龄、身高、生命体征等)、病史及用药史等进行描述，并对两组年龄、身高、体重等进行比较，以衡量两组的可比性。

8.2.2. 依从性分析

- 用药依从性分析：比较两组病人是否按时按量使用试验药物，未用方案中禁用的药物和食物。
- 合并用药分析：需统计各组合并用药人数，并详细列表。

8.2.3. 疗效评价

疗效评价同时进行 PP 分析和 FAS 分析；

- **主要疗效评价指标**为发生复合终点事件（心血管死亡和心衰恶化再住院）的时间。

对于主要终点事件的缺失按照删失处理。

本研究的主要研究假设为：

H_0：$\lambda_T/\lambda_C \geq 1$

H_1：$\lambda_T/\lambda_C < 1$

其中，λ_T 和 λ_C 分别为试验组和对照组发生终点事件的风险。

利用 Kaplan-Meier 法估计临床终点事件发生率，两组之间进行 Log rank 检验。利用 COX 比例风险模型，以中心为协变量，计算两组间的风险比（Hazard Ratio）及其 95%可信区间。另外，对复合终点事件的两个部分分别进行分析，即心血管死亡和心衰恶化再住院。

由于本次试验进行了一次期中分析（见 8.3 节），最终分析的 I 类误差应为 α =0.02314（单侧），即 0.04628（双侧试验）。将同时计算主要疗效终点的风险比及其 95.372% 置信区间。 P 值小于 0.04628（双侧检验）被认为对于主要终点的最终分析具有统计学意义。

- **次要疗效指标：**

  全因死亡率：利用 Kaplan-Meier 法估计两组全因死亡率，并进行 Log-rank 检验。利用 COX 比例风险模型，以中心为协变量，估计两组间的风险比（Hazard Ratio）及其 95%可信区间。

  复合终点事件（心衰恶化放弃治疗、心脏骤停后复苏成功、恶性心律失常、非致死性卒中）：分析方法与全因死亡相同。

  冠心病心衰患者的心血管死亡和心衰恶化再住院事件：按计数资料统计分析；

  血清 NT-proBNP：按计量资料分析，对两组血清 NT-proBNP 水平进行统计描述和组间比较，并对两组与基线的变化情况进行统计描述和组间比较。

8.2.4. 亚组分析

研究将进行亚组分析以对比治疗组间的疗效。对于每个亚组，将分析主要疗效终点，并分别分析复合终点事件的 2 个组成部分，心血管死亡和心力衰竭住院。计算风险比和相应的 95%CI，并在每个亚组内的研究组和对照组之间进行比较。 探索性亚组分析将基于 FAS 和 PPS。

对以下 19 个亚组进行分析:

- 年龄：≤70，>70 岁
- 性别：男，女
- NYHA 等级：I&II 级，III&IV 级
- LVEF: ≤30%, >30%
- 心力衰竭病程：≤3 年，>3 年
- 冠心病：是，否
- 心肌病：是，否
- 高血压：是，否
- 糖尿病：是，否
- 房颤：是，否
- 心律失常：是，否
- NT-proBNP：≤中位数（基于 FAS），> 中位数（基于 FAS）
- BMI: <25, ≥25
- eGFR: <90, ≥90, 未查
- 基线接受 ACEI/ARB/ARNi：是，否
- 基线接受 Beta-Blocker at baseline：是，否
- 基线接受 MRA：是，否
- 基线接受 SGLT2：是，否
- 基线接受三联治疗：是，否

8.2.5. 安全性评价

安全性评价基于 SS 数据集进行分析。

不良事件用不良事件发生例次、例数及发生率进行描述，并对该发生率进行组间显著性检验。同时，列表详细描述各组病例出现的全部不良事件的具体表现、程度及其与药物的关系。

对实验室指标前后变化情况进行交叉表描述，按试验组和对照组分别描述治疗前正常、治疗后异常例数及该例数所占比例。对生命体征指标进行前后比较。

8.3. 期中分析

本研究计划在收集到 1/2 和 2/3 主要终点事件后进行两次期中疗效分析，以评估是否已得出有效的结论而提前终止该研究。根据 Lan-DeMets α 消耗函数和 O’Brien-Fleming 方法，第 1 次期中分析时消耗的 I 类错误 α=0.0001（单侧），第 2 次期中分析消耗 α=0.00605（单侧）。

期中分析有关的具体要求和操作将在 DSMB 章程中事先规定。

在收集了 2/3 的主要终点事件后进行的中期分析中，结果显示单侧 α =0.006048，双侧 α 为 0.012097。 最终分析的 I 类错误 α =0.02314（单侧）。

9. 统计分析结果

9.1. 病例分布（所有随机化人群）

表9.1.1 各中心病例分布

中心	组别	入组数	脱落数	脱落率 (%)	剔除数	剔除率 (%)	完成
南京医科大学第一附属医院	试验组						
	对照组						
	合计						
河北以岭医院	试验组						
	对照组						
	合计						
……	试验组						
	对照组						
	合计						
合计	试验组						
	对照组						
	合计						

表9.1.2 两组脱落剔除情况比较

组别	脱落率(%)	P	剔除率(%)	P
试验组				
对照组				

注：脱落是指未完成试验的受试者，剔除是指完成试验但剔除 PPS 集的受试者。

表9.1.3 各中心的人群划分情况描述

中心	PPS			FAS			SS		
	试验组	对照组	合计	试验组	对照组	合计	试验组	对照组	合计
南京医科大学第一附属医院									
河北以岭医院									
……									
合计									

注：下表开始中心都以中心编号表示

表9.1.4　入组病例及安全性有效性分析数据集

项目	试验组	对照组	合计
全数据集			
随机入组			
完成试验			
试验期间中止			
不符合入选标准或属于排除标准			
不良事件			
缺乏疗效			
违背试验方案(包括依从性差)			
撤回知情同意书			
失访或患者未按时来院复诊			
其他原因			
安全性分析集			
SS			
有效性分析集			
FAS			
PPS			

9.2. 人口学和基线信息(FAS)

表9.2.1 人口学资料

项目	指标	试验组	对照组	合计	统计量	P
年龄（岁）	N(Missing)					
	Mean(Sd)					
	Median					
	Q1,Q3					
	Min,Max					
性别	男 n(%)					
	女 n(%)					
	合计(缺失)					
婚姻	未婚 n(%)					
	已婚 n(%)					
	其他 n(%)					
	合计(缺失)					
民族	汉族 n(%)					
	其他 n(%)					
	合计(缺失)					
身高(cm)	N(Missing)					
	Mean(Sd)					
	Median					
	Q1,Q3					
	Min,Max					
体重(kg)	N(Missing)					
	Mean(Sd)					
	Median					
	Q1,Q3					
	Min,Max					
BMI(kg/m^2)	N(Missing)					
	Mean(Sd)					
	Median					
	Q1,Q3					
	Min,Max					
国籍	中国 n(%)					
	其他 n(%)					
	合计(缺失)					

表9.2.2 个人史

项目	指标	试验组	对照组	合计	统计量	P
吸烟史	有 n(%)					
	无 n(%)					

项目	指标	试验组	对照组	合计	统计量	P
	已戒烟 n(%)					
	合计(缺失)					
吸烟数量(支/日)	N(Missing)					
	Mean(Sd)					
	Median					
	Q1,Q3					
	Min,Max					
饮酒史	有 n(%)					
	无 n(%)					
	已戒酒 n(%)					
	合计(缺失)					
饮酒数量（两/日	N(Missing)					
	Mean(Sd)					
	Median					
	Q1,Q3					
	Min,Max					
月经史	未绝经 n(%)					
	已绝经 n(%)					
	不适用 n(%)					
	合计(缺失)					
药物过敏史	有 n(%)					
	无 n(%)					
	合计(缺失)					

表9.2.3　疾病情况和既往病史

项目	指标	试验组	对照组	合计	统计量	P
冠心病	否 n(%)					
	是 n(%)					
	合计(缺失)					
心肌病	否 n(%)					
	是 n(%)					
	合计(缺失)					
高血压性心脏病	否 n(%)					
	是 n(%)					
	合计(缺失)					
其他	否 n(%)					
	是 n(%)					
	合计(缺失)					
心衰病程	≤3 年					
	>3 年					

项目	指标	试验组	对照组	合计	统计量	P
	合计(缺失)					
心衰病程(月)	N(Missing)					
	Mean(Sd)					
	Median					
	Q1,Q3					
	Min,Max					

表9.2.4　重要既往病史分析

项目	指标	试验组	对照组	合计	统计量	P
心肌梗死	无 n(%)					
	有 n(%)					
	合计(缺失)					
高血压	无 n(%)					
	有 n(%)					
	合计(缺失)					
糖尿病	无 n(%)					
	有 n(%)					
	合计(缺失)					
高脂血症	无 n(%)					
	有 n(%)					
	合计(缺失)					
脑卒中	无 n(%)					
	有 n(%)					
	合计(缺失)					
房颤	无 n(%)					
	有 n(%)					
	合计(缺失)					
心率失常	无 n(%)					
	有 n(%)					
	合计(缺失)					

注：重要病史按既往病史编码后的首选术语进行归类。

表9.2.5　既往病史分析（SOC/PT）

	试验组			对照组			
项目	例次	人数	百分率	例次	人数	百分率	P 值
既往病史							

表9.2.6　重要病史（PT）

项目	试验组			对照组			P 值
	例次	人数	百分率	例次	人数	百分率	
高血压							
糖尿病							
高脂血症							
房颤							
心肌梗死							
脑卒中							
心律失常							

以上重要病史按既往病史编码后的首选术语进行归类。

表9.2.7　NYHA 心功能分级

项目	指标	试验组	对照组	合计	统计量	P
NYHA 心功能分级	1.I级，日常活动无心衰症状；（※I级者，请不要入组）n(%)					
	2.II级，日常活动出现心衰症状（呼吸困难、乏力）；n(%)					
	3.III级，低于日常活动出现心衰症状；n(%)					
	4.IV级，在休息时出现心衰症状；（※IV级者，需考虑试验期间标准					
	合计(缺失)					

表9.2.8　心脏彩色超声检查

项目	指标	试验组	对照组	合计	统计量	P
是否检测	是 n(%)					
	否 n(%)					
	合计(缺失)					
左室射血分数(LVEF)(%)	N(Missing)					
	Mean(Sd)					
	Median					
	Q1,Q3					
	Min,Max					

表9.2.9 血清 NT-proBNP 检测

项目	指标	试验组	对照组	合计	统计量	P
是否检测-访视 1	是 n(%)					
	否 n(%)					
	合计(缺失)					
NT-proBNP (pg/ml)	N(Missing)					
	Mean(Sd)					
	Median					
	Q1,Q3					
	Min,Max					
是否检测-访视 2	是 n(%)					
	否 n(%)					
	合计(缺失)					
NT-proBNP (pg/ml)	N(Missing)					
	Mean(Sd)					
	Median					
	Q1,Q3					
	Min,Max					
是否检测-访视 3	是 n(%)					
	否 n(%)					
	合计(缺失)					
NT-proBNP (pg/ml)	N(Missing)					
	Mean(Sd)					
	Median					
	Q1,Q3					
	Min,Max					

表9.2.10 生命体征

项目	指标	试验组	对照组	合计	统计量	P
收缩压(mmHg)	N(Missing)					
	Mean(Sd)					
	Median					
	Q1,Q3					
	Min,Max					
舒张压(mmHg)	N(Missing)					
	Mean(Sd)					
	Median					
	Q1,Q3					
	Min,Max					
心率(次/分)	N(Missing)					
	Mean(Sd)					
	Median					
	Q1,Q3					
	Min,Max					

项目	指标	试验组	对照组	合计	统计量	P
脉搏(次/分)	N(Missing)					
	Mean(Sd)					
	Median					
	Q1,Q3					
	Min,Max					
体温(℃)	N(Missing)					
	Mean(Sd)					
	Median					
	Q1,Q3					
	Min,Max					

表9.2.11 基线肌酐及 eGFR

项目	指标	试验组	对照组	合计	统计量	P
基线的肌酐	N(Missing)					
	Mean(Sd)					
	Median					
	Q1,Q3					
	Min,Max					
eGFR	N(Missing)					
	Mean(Sd)					
	Median					
	Q1,Q3					
	Min,Max					
eGFR 分类	<60ml/min/1.73m^2 n(%)					
	>=60ml/min/1.73m^{2}n(%)					
	未查 n(%)					
	合计(缺失)					

注：eGFR 按照 60ml/min/1.73 m^{2}进行分类。

9.3. 中位随访时间

表9.3.1 随访时间描述

		FAS		PPS	
项目	指标	试验组	对照组	试验组	对照组
随访时间(天)	N(Missing)				
	Mean(Sd)				
	Median				
	Q1,Q3				
	Min,Max				
两组比较	统计量				
	P 值				

表9.3.2 中位随访时间

项目	指标	FAS 试验组	FAS 对照组	PPS 试验组	PPS 对照组
随访	N(Missing)				
	终点事件(%)				
	删失(%)				
	中位随访时间				
	95%CI				
	Q1,Q3				
	Log-rank 检验				
	P 值				
Cox 回归	Hazard Ratio				
	95%CI				
	Wald 卡方				
	P 值				

9.4. 用药情况(SS)

表9.4.1 用药依从性

项目	指标	试验组	对照组	合计	统计量	P 值
用药依从性	<80%n(%)					
	80%-120%n(%)					
	>120%n(%)					
	合计(Missing)					

注：实际服用药物量在应用药物量的 80%~120%范围内，可判定为用药依从性符合方案要求.

表9.4.2 既往用药/合并用药

项目	指标	试验组	对照组	合计	统计量	P
是否有既往用药/合并用药	否 n(%)					
	是 n(%)					
	合计(缺失)					

表9.4.3 合并用药编码（ATC1/ATC2）

项目	试验组 例次	试验组 人数	试验组 百分率	对照组 例次	对照组 人数	对照组 百分率	P 值
ATC1							
ATC2							

表9.4.4 螺内酯、ARNi、 SGLT2i 和 强心苷使用情况

项目	指标	试验组	对照组	合计	统计量	P
螺内酯	无 n(%)					
	有 n(%)					
	合计(缺失)					
ARNi	无 n(%)					
	有 n(%)					
	合计(缺失)					
SGLT2i	无 n(%)					
	有 n(%)					
	合计(缺失)					
强心苷	无 n(%)					
	有 n(%)					
	合计(缺失)					

表9.4.5 接受三联治疗（RAAS+Beta+MRA）的情况

项目	指标	试验组	对照组	合计	统计量	P
RAAS+Beta+MRA	否 n(%)					
	是 n(%)					
	合计(缺失)					

9.5. 主要疗效指标评价（FAS&PPS）

表9.5.1 发生复合终点事件（心血管死亡和心衰恶化再住院）的时间

项目	指标	FAS		PPS	
		试验组	对照组	试验组	对照组
复合终点事件发生时间	N(Missing)				
	终点事件(%)				
	删失(%)				
	中位生存时间				
	95%CI				
	Q1,Q3				
	Log-rank 检验				
	P 值				
Cox 回归	Hazard Ratio				
	95%CI				
	95.372%CI				
	Wald 卡方				
	P 值				

*Cox 回归协变量为中心，下同。

图 1 发生复合终点事件 KM 曲线图（FAS 数据集）

图 2 发生复合终点事件 KM 曲线图（PPS 数据集）

表9.5.2 发生心血管死亡的时间

		FAS		PPS	
项目	指标	试验组	对照组	试验组	对照组
心血管死亡发生时间	N(Missing)				
	终点事件(%)				
	删失(%)				
	中位发生时间				
	95%CI				
	Q1,Q3				
	Log-rank 检验				
	P 值				
Cox 回归	Hazard Ratio				
	95%CI				
	95.372%CI				
	Wald 卡方				
	P 值				

*Cox 回归协变量为中心，下同。

图 3 心血管死亡 KM 曲线图（FAS 数据集）

图 4 心血管死亡 KM 曲线图（PPS 数据集）

表9.5.3 发生心衰恶化再住院的时间

项目	指标	FAS		PPS	
		试验组	对照组	试验组	对照组
心衰恶化再住院发生时间	N(Missing)				
	终点事件(%)				
	删失(%)				
	中位发生时间				
	95%CI				
	Q1,Q3				
	Log-rank 检验				
	P 值				
Cox 回归	Hazard Ratio				
	95%CI				
	95.372%CI				
	Wald 卡方				
	P 值				

图 5 心衰恶化再住院 KM 曲线图（FAS 数据集）

图 6 心衰恶化再住院 KM 曲线图（PPS 数据集）

9.6. 主要疗效指标评价-亚组分析

表9.6.1 复合终点事件（心血管死亡和心衰恶化再住院）（≤70 岁）

		FAS		PPS	
项目	指标	试验组	对照组	试验组	对照组
复合终点事件发生时间	N(Missing)				
	终点事件(%)				
	删失(%)				
	中位生存时间				
	95%CI				
	Q1,Q3				
	Log-rank 检验				
	P 值				
Cox 回归	Hazard Ratio				
	95%CI				
	Wald 卡方				
	P 值				

表9.6.2 复合终点事件（心血管死亡和心衰恶化再住院）（＞70 岁）

		FAS		PPS	
项目	指标	试验组	对照组	试验组	对照组
复合终点事件发生时间	N(Missing)				
	终点事件(%)				
	删失(%)				
	中位生存时间				
	95%CI				
	Q1,Q3				
	Log-rank 检验				
	P 值				
Cox 回归	Hazard Ratio				
	95%CI				
	Wald 卡方				
	P 值				

表9.6.3 发生心血管死亡的时间（≤70 岁）

下述亚组分析结果，表格形式同上

表9.6.4 发生心血管死亡的时间（>70 岁）

表9.6.5 发生心衰恶化再住院的时间（≤70 岁）

表9.6.6 发生心衰恶化再住院的时间（>70 岁）

表9.6.7 复合终点事件（心血管死亡和心衰恶化再住院）（男性）

表9.6.8 复合终点事件（心血管死亡和心衰恶化再住院）（女性）

表9.6.9 发生心血管死亡的时间（男性）

表9.6.10 发生心血管死亡的时间（女性）

表9.6.11 发生心衰恶化再住院的时间（男性）

表9.6.12 发生心衰恶化再住院的时间（女性）

表9.6.13 复合终点事件（心血管死亡和心衰恶化再住院）（NYHA 分级 I 级或 II 级）

表9.6.14 复合终点事件（心血管死亡和心衰恶化再住院）（NYHA 分级 III 级或IV级）

表9.6.15 发生心血管死亡的时间（NYHA 分级 I 级或 II 级）

表9.6.16 发生心血管死亡的时间（NYHA 分级 III 级或IV级）

表9.6.17 发生心衰恶化再住院的时间（NYHA 分级 I 级或 II 级）

表9.6.18 发生心衰恶化再住院的时间（NYHA 分级 III 级或IV级）

表9.6.19 复合终点事件（心血管死亡和心衰恶化再住院）（LVEF≤30%）

表9.6.20 复合终点事件（心血管死亡和心衰恶化再住院）（LVEF>30%）

表9.6.21 发生心血管死亡的时间（LVEF≤30%）

表9.6.22 发生心血管死亡的时间（LVEF>30%）

表9.6.23 发生心衰恶化再住院的时间（LVEF≤30%）

表9.6.24 发生心衰恶化再住院的时间（LVEF>30%）

表9.6.25 复合终点事件（心血管死亡和心衰恶化再住院）（心衰病程≤3 年）

表9.6.26 复合终点事件（心血管死亡和心衰恶化再住院）（心衰病程>3 年）

表9.6.27 发生心血管死亡的时间（心衰病程≤3 年）

表9.6.28 发生心血管死亡的时间（心衰病程>3 年）

表9.6.29 发生心衰恶化再住院的时间（心衰病程≤3 年）

表9.6.30 发生心衰恶化再住院的时间（心衰病程>3 年）

表9.6.31 复合终点事件（心血管死亡和心衰恶化再住院）（有冠心病）

表9.6.32 复合终点事件（心血管死亡和心衰恶化再住院）（无冠心病）

表9.6.33 发生心血管死亡的时间（有冠心病）

表9.6.34 发生心血管死亡的时间（无冠心病）

表9.6.35 发生心衰恶化再住院的时间（有冠心病）

表9.6.36 发生心衰恶化再住院的时间（无冠心病）

表9.6.37 复合终点事件（心血管死亡和心衰恶化再住院）（有心肌病）

表9.6.38 复合终点事件（心血管死亡和心衰恶化再住院）（无心肌病）

表9.6.39 发生心血管死亡的时间（有心肌病）

表9.6.40 发生心血管死亡的时间（无心肌病）

表9.6.41 发生心衰恶化再住院的时间（有心肌病）

表9.6.42 发生心衰恶化再住院的时间（无心肌病）

表9.6.43 复合终点事件（心血管死亡和心衰恶化再住院）（有高血压病）

表9.6.44 复合终点事件（心血管死亡和心衰恶化再住院）（无高血压病）

表9.6.45 发生心血管死亡的时间（有高血压病）

表9.6.46 发生心血管死亡的时间（无高血压病）

表9.6.47 发生心衰恶化再住院的时间（有高血压病）

表9.6.48 发生心衰恶化再住院的时间（无高血压病）

表9.6.49 复合终点事件（心血管死亡和心衰恶化再住院）（有糖尿病）

表9.6.50 复合终点事件（心血管死亡和心衰恶化再住院（无糖尿病）

表9.6.51 发生心血管死亡的时间（有糖尿病）

表9.6.52 发生心血管死亡的时间（无糖尿病）

表9.6.53 发生心衰恶化再住院的时间（有糖尿病）

表9.6.54 发生心衰恶化再住院的时间（无糖尿病）

表9.6.55 复合终点事件（心血管死亡和心衰恶化再住院）（有房颤）

表9.6.56 复合终点事件（心血管死亡和心衰恶化再住院）（无房颤）

表9.6.57 发生心血管死亡的时间（有房颤）

表9.6.58 发生心血管死亡的时间（无房颤）

表9.6.59 发生心衰恶化再住院的时间（有房颤）

表9.6.60 发生心衰恶化再住院的时间（无房颤）

表9.6.61 复合终点事件（心血管死亡和心衰恶化再住院）（有心率失常）

表9.6.62 复合终点事件（心血管死亡和心衰恶化再住院）（无心率失常）

表9.6.63 发生心血管死亡的时间（有心率失常）

表9.6.64 发生心血管死亡的时间（无心率失常）

表9.6.65 发生心衰恶化再住院的时间（有心率失常）

表9.6.66 发生心衰恶化再住院的时间（无心率失常）

表9.6.67 复合终点事件（心血管死亡和心衰恶化再住院）（NT-proBNP ≤中位数）

注：中位数为总体（FAS 人群中）基线 NT-proBNP 中位数。

表9.6.68 复合终点事件（心血管死亡和心衰恶化再住院）（NT-proBNP >中位数）

注：中位数为总体（FAS 人群中）基线 NT-proBNP 中位数值。

表9.6.69 发生心血管死亡的时间（NT-proBNP ≤中位数）

注：中位数为总体（FAS 人群中）基线 NT-proBNP 中位数值。

表9.6.70 发生心血管死亡的时间（NT-proBNP >中位数）

注：中位数为总体（FAS 人群中）基线 NT-proBNP 中位数值。

表9.6.71 发生心衰恶化再住院的时间（NT-proBNP ≤中位数）

注：中位数为总体（FAS 人群中）基线 NT-proBNP 中位数值。

表9.6.72 发生心衰恶化再住院的时间（NT-proBNP >中位数）

注：中位数为总体（FAS 人群中）基线 NT-proBNP 中位数值。

表9.6.73 复合终点事件（心血管死亡和心衰恶化再住院）（BMI＜25）

表9.6.74 复合终点事件（心血管死亡和心衰恶化再住院）（BMI≥25）

表9.6.75 复合终点事件（心血管死亡和心衰恶化再住院）（eGFR＜90）

表9.6.76 复合终点事件（心血管死亡和心衰恶化再住院）（eGFR≥90）

表9.6.77 复合终点事件（心血管死亡和心衰恶化再住院）（eGFR 未查）

表9.6.78 复合终点事件（心血管死亡和心衰恶化再住院）（基线使用 ACEI/ARB/ARNi 的受试者）

表9.6.79 复合终点事件（心血管死亡和心衰恶化再住院）（基线未使用 ACEI/ARB/ARNi 的受试者）

表9.6.80 复合终点事件（心血管死亡和心衰恶化再住院）（基线接受 β 受体阻滞剂治疗）

表9.6.81 复合终点事件（心血管死亡和心衰恶化再住院）（基线未接受 β 受体阻滞剂治疗）

表9.6.82 复合终点事件（心血管死亡和心衰恶化再住院）（基线接受 MRA 治疗）

表9.6.83 复合终点事件（心血管死亡和心衰恶化再住院）（基线未接受 MRA 治疗）

表9.6.84 复合终点事件（心血管死亡和心衰恶化再住院）（基线接受 ARNi 治疗）

表9.6.85 复合终点事件（心血管死亡和心衰恶化再住院）（基线未接受 ARNi 治疗）

表9.6.86 复合终点事件（心血管死亡和心衰恶化再住院）（基线有 SGLT2i）

表9.6.87 复合终点事件（心血管死亡和心衰恶化再住院）（基线无 SGLT2i）

表9.6.88 复合终点事件（心血管死亡和心衰恶化再住院）（基线接受三联治疗）

表9.6.89 复合终点事件（心血管死亡和心衰恶化再住院）（基线未接受三联治疗）

表9.6.90 发生心衰恶化再住院的时间（BMI＜25）

表9.6.91 发生心衰恶化再住院的时间（BMI≥25）

表9.6.92 发生心衰恶化再住院的时间（eGFR＜90）

表9.6.93 发生心衰恶化再住院的时间（eGFR≥90）

表9.6.94 发生心衰恶化再住院的时间（eGFR 未查）

表9.6.95 发生心衰恶化再住院的时间（基线使用 ACEI/ARB/ARNi 的受试者）

表9.6.96 发生心衰恶化再住院的时间（基线未使用 ACEI/ARB/ARNi 的受试者）

表9.6.97 发生心衰恶化再住院的时间（基线接受β受体阻滞剂治疗）

表9.6.98 发生心衰恶化再住院的时间（基线未接受β受体阻滞剂治疗）

表9.6.99 发生心衰恶化再住院的时间（基线接受 MRA 治疗）

表9.6.100 发生心衰恶化再住院的时间（基线未接受 MRA 治疗）

表9.6.101 发生心衰恶化再住院的时间（基线接受 ARNi 治疗）

表9.6.102 发生心衰恶化再住院的时间（基线未接受 ARNi 治疗）

表9.6.103 发生心衰恶化再住院的时间（基线有 SGLT2i）

表9.6.104 发生心衰恶化再住院的时间（基线无 SGLT2i）

表9.6.105 发生心衰恶化再住院的时间（基线接受三联治疗）

表9.6.106 发生心衰恶化再住院的时间（基线未接受三联治疗）

表9.6.107 发生心血管死亡的时间（BMI＜25）

表9.6.108 发生心血管死亡的时间（BMI≥25）

表9.6.109 发生心血管死亡的时间（eGFR＜90）

表9.6.110 发生心血管死亡的时间（eGFR≥90）

表9.6.111 发生心血管死亡的时间（eGFR 未查）

表9.6.112 发生心血管死亡的时间（基线使用 ACEI/ARB/ARNi 的受试者）

表9.6.113 发生心血管死亡的时间（基线未使用 ACEI/ARB/ARNi 的受试者）

表9.6.114 发生心血管死亡的时间（基线接受β受体阻滞剂治疗）

表9.6.115 发生心血管死亡的时间（基线未接受β受体阻滞剂治疗）

表9.6.116 发生心血管死亡的时间（基线接受 MRA 治疗）

表9.6.117 发生心血管死亡的时间（基线未接受 MRA 治疗）

表9.6.118 发生心血管死亡的时间（基线接受 ARNi 治疗）

表9.6.119 发生心血管死亡的时间（基线未接受 ARNi 治疗）

表9.6.120 发生心血管死亡的时间（基线有 SGLT2i）

表9.6.121 发生心血管死亡的时间（基线无 SGLT2i）

表9.6.122 发生心血管死亡的时间（基线接受三联治疗）

表9.6.123 发生心血管死亡的时间（基线未接受三联治疗）

9.7. 次要疗效指标评价（FAS&PPS）

表9.7.1 全因死亡率

项目	指标	FAS 试验组	FAS 对照组	PPS 试验组	PPS 对照组
全因死亡	N(Missing)				
	终点事件(%)				
	删失(%)				
	中位生存时间				
	95%CI				
	Q1,Q3				
	Log-rank 检验				
	P 值				
Cox 回归	Hazard Ratio				
	95%CI				
	Wald 卡方				
	P 值				

表9.7.2 复合终点事件（心衰恶化放弃治疗、心脏骤停后复苏成功、恶性心律失常、非致死性卒中）率

项目	指标	FAS 试验组	FAS 对照组	PPS 试验组	PPS 对照组
复合终点事件（心衰恶化放弃治疗、心脏骤停后复苏成功、恶性心律失常、非致死性卒中）	N(Missing)				

项目	指标	FAS 试验组	FAS 对照组	PPS 试验组	PPS 对照组
	终点事件(%)				
	删失(%)				
	中位生存时间				
	95%CI				
	Q1,Q3				
	Log-rank 检验				
	P 值				
Cox 回归	Hazard Ratio				
	95%CI				
	Wald 卡方				
	P 值				

表9.7.3　心衰恶化放弃治疗率

项目	指标	FAS		PPS	
		试验组	对照组	试验组	对照组
心衰恶化放弃治疗	N(Missing)				
	终点事件(%)				
	删失(%)				
	中位生存时间				
	95%CI				
	Q1,Q3				
	Log-rank 检验				
	P 值				
Cox 回归	Hazard Ratio				
	95%CI				
	Wald 卡方				
	P 值				

表9.7.4　心脏骤停后复苏成功率

项目	指标	FAS		PPS	
		试验组	对照组	试验组	对照组
心脏骤停后复苏成功	N(Missing)				
	终点事件(%)				
	删失(%)				
	中位生存时间				
	95%CI				
	Q1,Q3				
	Log-rank 检验				
	P 值				

项目	指标	FAS 试验组	FAS 对照组	PPS 试验组	PPS 对照组
Cox 回归	Hazard Ratio				
	95%CI				
	Wald 卡方				
	P 值				

表9.7.5 恶性心律失常率

项目	指标	FAS 试验组	FAS 对照组	PPS 试验组	PPS 对照组
恶性心律失常	N(Missing)				
	终点事件(%)				
	删失(%)				
	中位生存时间				
	95%CI				
	Q1,Q3				
	Log-rank 检验				
	P 值				
Cox 回归	Hazard Ratio				
	95%CI				
	Wald 卡方				
	P 值				

表9.7.6 非致死性卒中率

项目	指标	FAS 试验组	FAS 对照组	PPS 试验组	PPS 对照组
非致死性卒中	N(Missing)				
	终点事件(%)				
	删失(%)				
	中位生存时间				
	95%CI				
	Q1,Q3				
	Log-rank 检验				
	P 值				
Cox 回归	Hazard Ratio				
	95%CI				
	Wald 卡方				
	P 值				

表9.7.7 冠心病心衰患者的心血管死亡和心衰恶化再住院

		FAS		PPS	
项目	指标	试验组	对照组	试验组	对照组
复合事件	是 n(%)				
	否 n(%)				
	合计(缺失)				
	统计量				
	P 值				
心血管死亡	是 n(%)				
	否 n(%)				
	合计(缺失)				
	统计量				
	P 值				
心衰恶化再住院	是 n(%)				
	否 n(%)				
	合计(缺失)				
	统计量				
	P 值				

表9.7.8 血清 NT-proBNP 较基线变化情况（1 个月）

		FAS		PPS	
项目	指标	试验组	对照组	试验组	对照组
基线 NT-proBNP(pg/ml)	N(Missing)				
	Mean(Sd)				
	Median				
	Q1,Q3				
	Min,Max				
基线两组比较	统计量				
	P 值				
治疗 1 个月	N(Missing)				
	Mean(SD)				
	Median				
	Q1,Q3				
	Min,Max				
治疗 1 个月两组比较	统计量				
	P 值				
前后差值	N(Missing)				
	Mean(SD)				
	Median				
	Q1,Q3				

项目	指标	FAS 试验组	FAS 对照组	PPS 试验组	PPS 对照组
	Min,Max				
组内前后比较	统计量				
	P 值				
前后差值两组比较	统计量				
	P 值				

表9.7.9　血清 NT-proBNP 较基线变化情况（3 个月）

表格同上

表9.7.10 血清 NT-proBNP 较基线下降率（1 个月）

		FAS		PPS	
项目	指标	试验组	对照组	试验组	对照组
下降率（%）	N(Missing)				
	Mean(Sd)				
	Median				
	Q1,Q3				
	Min,Max				
两组比较	统计量				
	P 值				
下降率≥30%	否 n(%)				
	是 n(%)				
	合计(缺失)				
两组比较	统计量				
	P 值				

注：下降率=（基线结果-1 个月结果）/基线结果*100%

表9.7.11 血清 NT-proBNP 较基线下降率（3 个月）

表格同上

9.8.　安全性评价（SS）

表9.8.1　不良事件汇总比较

项目	指标	试验组	对照组	合计	统计量	P
是否不良事件	否 n(%)					
	是 n(%)					
	合计(缺失)					
是否严重不良事件	否 n(%)					

项目	指标	试验组	对照组	合计	统计量	P
	是 n(%)					
	合计(缺失)					

表9.8.2 研究期间不良事件汇总比较

项目	试验组			对照组			
	例次	人数	百分率	例次	人数	百分率	P 值
不良事件							
与研究药物相关不良事件							
严重程度 3 4 5 级不良事件							
严重不良事件							
与研究药物相关的严重不良事件							
导致脱落的不良事件							
导致脱落的与研究药物相关的不良事件							

注：与研究药物相关定义为肯定有关、可能有关、无法判定。

表9.8.3 不良事件编码情况（SOC/PT）

项目	试验组			对照组			
	例次	人数	百分率	例次	人数	百分率	P 值
不良事件							

表9.8.4 不良反应编码情况（SOC/PT）

项目	试验组			对照组			
	例次	人数	百分率	例次	人数	百分率	P 值
不良事件							

表9.8.5 生命体征前后比较(12 个月±7 天)-收缩压（mmHg）

项目	指标	试验组	对照组
		SS	
基线收缩压	N(Missing)		
	Mean(Sd)		
	Median		
	Q1,Q3		
	Min,Max		
	统计量		

项目	指标	SS 试验组	对照组
	P 值		
入组 12 个月收缩压	N(Missing)		
	Mean(SD)		
	Median		
	Q1,Q3		
	Min,Max		
入组 12 个月两组比较	统计量		
	P 值		
前后差值	N(Missing)		
	Mean(SD)		
	Median		
	Q1,Q3		
	Min,Max		
组内前后比较	统计量		
	P 值		
前后差值两组比较	统计量		
	P 值		
前后变化率	N(Missing)		
	Mean(SD)		
	Median		
	Q1,Q3		
	Min,Max		
组内前后变化率比较	统计量		
	P 值		
两组前后变化率比较	统计量		
	P 值		

表9.8.6　生命体征前后比较(12 个月±7 天)-舒张压（mmHg）

表格同上

表9.8.7　生命体征前后比较(12 个月±7 天)-心率（次/分）

表格同上

表9.8.8　生命体征前后比较(12 个月±7 天)-脉搏（次/分）

表格同上

表9.8.9　生命体征前后比较(12 个月±7 天)-体温（℃）

表格同上

表9.8.10 血常规-12 个月±7 天白细胞总数前后交叉表

		白细胞总数					
组别	用药前	正常	异常无意义	异常有意义	未查	缺失	合计
试验组							
	正常						
	异常无意义						
	异常有意义						
	未查						
	缺失						
	合计						
对照组							
	正常						
	异常无意义						
	异常有意义						
	未查						
	缺失						
	合计						

表9.8.11 血常规-12 个月±7 天血红蛋白前后交叉表

下述交叉表，表格格式同上

表9.8.12 血常规-12 个月±7 天红细胞总数前后交叉表

表9.8.13 血常规-12 个月±7 天血小板前后交叉表

表9.8.14 血常规-12 个月±7 天汇总表（SS）

		用药前正常		用药前异常	
指标	组别	用药后正常	用药后异常	用药后正常	用药后异常
白细胞总数（WBC）	试验组				
	对照组				
血红蛋白（Hb）	试验组				
	对照组				
红细胞总数（RBC）	试验组				
	对照组				
血小板（PLT）	试验组				
	对照组				

表9.8.15 血生化-12 个月±7 天谷丙转氨酶前后交叉表

表9.8.16 血生化-12 个月±7 天肌酐前后交叉表

表9.8.17 血生化-12 个月±7 天尿素氮前后交叉表

表9.8.18 血生化-12 个月±7 天总胆固醇前后交叉表

表9.8.19 血生化-12 个月±7 天甘油三酯前后交叉表

表9.8.20 血生化-12 个月±7 天钾前后交叉表

表9.8.21 血生化-12 个月±7 天钠前后交叉表

表9.8.22 血生化-12 个月±7 天氯前后交叉表

表9.8.23 血生化-12 个月±7 天空腹血糖前后交叉表

表9.8.24 血生化-12 个月±7 天汇总表

		用药前正常		用药前异常	
指标	组别	用药后正常	用药后异常	用药后正常	用药后异常
谷丙转氨酶（ALT/GPT）	试验组				
	对照组				
肌酐（Cr）	试验组				
	对照组				
尿素氮（BUN）	试验组				
	对照组				
总胆固醇（TC）	试验组				
	对照组				
甘油三酯（TG）	试验组				
	对照组				
钾（K）	试验组				

指标	组别	用药前正常		用药前异常	
		用药后正常	用药后异常	用药后正常	用药后异常
	对照组				
钠（Na）	试验组				
	对照组				
氯（Cl）	试验组				
	对照组				
空腹血糖（GLU）	试验组				
	对照组				

表9.8.25 尿常规-12 个月±7 天尿蛋白前后交叉表

表9.8.26 尿常规-12 个月±7 天尿白细胞前后交叉表

表9.8.27 尿常规-12 个月±7 天尿红细胞前后交叉表

表9.8.28 尿常规-12 个月±7 天汇总表

指标	组别	用药前正常		用药前异常	
		用药后正常	用药后异常	用药后正常	用药后异常
尿蛋白（PRO）	试验组				
	对照组				
尿白细胞（WBC）	试验组				
	对照组				
尿红细胞（RBC）	试验组				
	对照组				

表9.8.29 体格检查-12 个月±7 天一般情况前后交叉表

表9.8.30 体格检查-12 个月±7 天神经系统前后交叉表

表9.8.31 体格检查-12 个月±7 天皮肤、浅表淋巴结前后交叉表

表9.8.32 体格检查-12 个月±7 天头颈部前后交叉表

表9.8.33 体格检查-12 个月±7 天心脏前后交叉表

表9.8.34 体格检查-12 个月±7 天肺部前后交叉表

表9.8.35 体格检查-12 个月±7 天腹部前后交叉表

表9.8.36 体格检查-12 个月±7 天泌尿生殖系统前后交叉表

表9.8.37 体格检查-12 个月±7 天脊柱四肢前后交叉表

表9.8.38 体格检查-12 个月±7 天其他前后交叉表

表9.8.39 体格检查-12 个月±7 天汇总表

		用药前正常		用药前异常	
指标	组别	用药后正常	用药后异常	用药后正常	用药后异常
一般情况	试验组				
	对照组				
神经系统	试验组				
	对照组				
皮肤、浅表淋巴结	试验组				
	对照组				
头颈部	试验组				
	对照组				
心脏	试验组				
	对照组				
肺部	试验组				
	对照组				
腹部	试验组				
	对照组				
泌尿生殖系统	试验组				
	对照组				
脊柱四肢	试验组				

指标	组别	用药前正常		用药前异常	
		用药后正常	用药后异常	用药后正常	用药后异常
	对照组				
其他	试验组				
	对照组				

表9.8.40 心电图-12 个月±7 天前后交叉表

表9.8.41 心电图-12 个月±7 天汇总表

指标	组别	用药前正常		用药前异常	
		用药后正常	用药后异常	用药后正常	用药后异常
心电图临床意义	试验组				
	对照组				

9.9. 清单

附表 1-脱落剔除清单（所有随机化人群）

中心	随机号	组别	是否完成试验	脱落日期	脱落原因	详述	状态	脱落或方案偏离分类	脱落或方案偏离具体原因	FAS	PPS	SS

附表 2-人口资料学清单（FAS）

中心	随机号	组别	出生日期	年龄(岁)	性别	婚姻	民族	其他	身高(cm)	体重(kg)	BMI(kg/m^2)	国籍	其他

附表 3-既往合并用药清单

中心	随机号	组别	有无既往/合并用药	药物名称（通用名）	用药原因	AE 编号	病史编号	剂量用法	开始日期	结束日期	末次随访是否持续	ATC1 术语	ATC2 术语

附表 4-用药依从性清单（研究药物发放使用情况）（FAS）

中心	随机号	组别	发药日期	最后访视日期	服药天数	应服药量（粒）	实际服药量（粒）	依从性(%)	FAS	PPS

附表 5-主要疗效清单

中心	随机号	组别	随机日期	复合终点事件日期	复合终点事件删失	时间（天）	心血管死亡发生时间	心血管死亡删失	时间（天）	心衰恶化再住院时间	心衰恶化再住院删失	时间（天）	FAS	PPS	SS

附表 6-次要指标疗效清单

中心	随机号	组别	随机日期	全因死亡日期	删失 1	时间(天)	复合终点事件（心衰恶化放弃治疗、心脏骤停后复苏成功、恶性心律失常、非致死性卒中）日期	删失 2	时间(天)	心衰恶化放弃治疗日期	删失 3	时间(天)	FAS	PPS

附表 6-1-次要指标疗效清单 -续表

中心	随机号	组别	随机日期	心脏骤停后复苏成功日期	删失 4	时间(天)	恶性心律失常日期	删失 5	时间(天)	非致死性卒中日期	删失 6	时间(天)	FAS	PPS

附表 7-血清 NT-proBNP

中心	随机号	组别	基线血清 NT-proBNP(pg/ml)	1 月血清 NT-proBNP(pg/ml)	差值(基线-1 个月)	下降率(%)	下降率≥30%	3 月血清 NT-proBNP(pg/ml)	差值(基线-3 个月)	下降率(%)	下降率≥30%	FAS	PPS

附表 8-不良事件清单

中心	随机号	组别	有无AE	AE 名称	发生日期	严重程度分级	是否SAE	死亡	危及生命	需要住院治疗或原来住院时间延长	致永久性或显著性残障/残疾或无行为能力	先天性畸形或出生缺陷	重要医学事件	对试验药物采取的措施	是否治疗	是否因此不良事件退出试验	转归	与研究药物的关系	不良事件详情描述	终止/转归日期	版本号	SOC	PT

附表 9-严重不良事件清单

中心	随机号	组别	有无AE	AE 名称	发生日期	严重程度分级	是否SAE	死亡	危及生命	需要住院治疗或原来住院时间延长	致永久性或显著性残障/残疾或无行为能力	先天性畸形或出生缺陷	重要医学事件	对试验药物采取的措施	是否治疗	是否因此不良事件退出试验	转归	与研究药物的关系	不良事件详情描述	终止/转归日期	版本号	SOC	PT

附表 10-血常规前正常后异常清单（SS）

中心	随机号	指标	分组	治疗前	治疗前临床意义	治疗后	治疗后临床意义

附表 11-血常规前异常后异常清单（SS）

同上

附表 12-血生化前正常后异常清单（SS）

同上

附表 13-血生化前异常后异常清单（SS）

同上

附表 14-尿常规前正常后异常清单（SS）

同上

附表 15-尿常规前异常后异常清单（SS）

同上

附表 16-体格检查前正常后异常清单（SS）

同上

附表 17-体格检查前异常后异常清单（SS）

同上

附表 18-心电图前正常后异常清单（SS）

同上

附表 19-心电图前异常后异常清单（SS）

同上

附表 20- 试验完成情况清单（所有随机化人群）

中心	随机号	组别	完成试验	脱落日期	脱落原因	详细记录	是否破盲	破盲原因	脱落日期
								.	
								.	
								.	

附表 21-破盲清单（所有随机化人群）

中心	随机号	组别	是否完成试验	随机日期	是否破盲	破盲日期	破盲原因	是否死亡	死亡日期

Qiliqiangxin in Heart FailUre:
AssESsment of Reduction in MorTality
(QUEST)

Statistical Analysis Plan V1.0

Research Unit：The First Affiliated Hospital of Nanjing Medical University

Drug Manufacturer：Shijiazhuang Yiling Pharmaceutical Co., Ltd

Statistical Analysis：Peking University Clinical Research Institute

Version date：Nov 30th 2022

Author： ____________________ **Date：** ____________________

Approve： ___________________ **Date：** ____________________

目录

1. ABBREVIATIONS 8

2. STUDY TITLE 9

3. STUDY OBJECTIVE 9

4. STUDY DESIGN 9

4.1. CONTROL GROUP AND SAMPLE SIZE 10
4.2. BLINDING AND UNBLINDING 11
4.3.1. Random grouping of subjects 11
4.3.2. Unblinding and emergency unblinding protocol 11
4.3.3. Unblinding provisions 12
4.3.4. Screening number 12
4.3. TREATMENT PLAN 12
4.4.1. Study Process 12
4.4.2. Concomitant medications and other treatments 13
4.4.3. Adverse drug reaction 14
4.4.4. Evaluation on compliance 14

5. RATIONALE FOR STUDY POPULATION 15

5.1. INCLUSION CRITERIA 15
5.2. EXCLUSION CRITERIA 15
5.3. PATIENT ENROLMENT AND FOLLOW-UP VISIT 17
5.4. DISCONTINUATION OF INVESTIGATIONAL PRODUCT (IP) 17
5.5. WITHDRAWAL 18
5.6. DISCONTINUATION OF THE STUDY 18

6. CLINICAL OBSERVATION ENDPOINTS AND INDICATORS 18

6.1. CLINICAL OBSERVATION ENDPOINTS 18
6.1.1. Primary outcome measure 18
6.1.2. Secondary outcome measures 18
6.2. SAFETY OUTCOME MEASURE: 19
6.2.1. Definition of Adverse Event 19
6.2.2. Criteria on severity of adverse events: 19
6.2.3. Adverse events of interest 20
6.2.4. Definition serious adverse events 21
6.2.5. Definition on specificity of serious adverse events 21
6.2.6. Recording of adverse events and follow-up 22
6.2.7. Adverse events based on examinations 22
6.3. EFFICACY ASSESSMENTS 22
6.3.1. Endpoint reporting overview 22
6.3.2. Potential endpoint events 23

7. DATA MANAGEMENT 25

7.1. DATABASE DESIGN 25
7.2. DATA ENTRY 25
7.3. DATA QUESTIONING MANAGEMENT 26
7.4. MEDICAL CODING 26
7.5. DATA AUDIT 26
7.6. DATABASE LOCKING 26

8. STATISTICAL ANALYSIS METHOD 26

8.1. DEFINITIONS OF ANALYSIS SETS 26
8.2. GENERAL STATISTICAL METHODS 27
8.2.1. Enrollment and completion analysis 28
8.2.2. Compliance analysis: 28
8.2.3. Efficacy analysis: 28
8.2.4. Safety analysis: 29
8.3. INTERIM ANALYSIS 29

9. STATISTICAL ANALYSIS RESULTS 30

9.1. CASE DISTRIBUTION (FOR ALL RANDOMIZED PATIENTS) 30
Table 9.1.1 Distribution of cases in each centerDefinitions of analysis sets 30
Table 9.1.2 Comparison of dropout and elimination between two groups 30
Table 9.1.3 Population in each center 30
Table 9.1.4 Enrolled cases and safety and efficacy analysis data sets 31
9.2. DEMOGRAPHIC AND BASELINE INFORMATION (FAS) 32
Table 9.2.1 Demographic 32
Table 9.2.2 Personal History 32
Table 9.2.3 Comorbidities and past medical history 33
Table 9.2.4 Baseline of past medical history 34
Table 9.2.5 Analysis of past medical history (SOC/PT) 34
Table 9.2.6 Analysis of major past medical history（PT） 34
Table 9.2.7 NYHA heart functional classification 35
Table 9.2.8 Echocardiogram 35
Table 9.2.9 Serum NT-proBNP 35
Table 9.2.10 Vitals 36
9.3. FOLLOW UP PERIOD 37
Table 9.3.1 Follow time descriptions 37
Table 9.3.2 Median follow time 37
9.4. MEDICATION (SS) 37
Table 9.4.1 Medication compliance 37
Table 9.4.2 Past Medication/Concomitant Medication 38
Table 9.4.3 Coding of Concomitant Medication (ATC1/ATC2) 38
Table 9.4.4 Usage of Spironolactone, ARNi, SGLT2i, and cardiac glycoside 39
Table 9.4.5 Triple therapy（RAAS+Beta+MRA） 39

9.5. EVALUATION OF PRIMARY EFFICACY INDICATORS (FAS&PPS) 40
Table 9.6.1 Major adverse cardiovascular events (MACE, CV death and HHF) 40
Figure 1 Kaplan-Meier analysis of MACEs (FAS) 40
Figure 2 Kaplan-Meier analysis of MACEs (PPS) 40
Table 9.6.2 Cardiovascular death 41
Figure 3 Kaplan-Meier analysis of Cardiovascular death (FAS) 41
Figure 4 Kaplan-Meier analysis of Cardiovascular death (PPS) 41
Table 9.6.3 Hospitalization of heart failure 42
Figure 5 Kaplan-Meier analysis of Hospitalization of heart failure (FAS) 42
Figure 6 Kaplan-Meier analysis of Hospitalization of heart failure (PPS) 42
9.6. EVALUATION OF MAIN EFFICACY INDICATORS - SUBGROUP ANALYSIS 43
Table 9.6.2 MACE (CV death and HHF) (>70 year old） 43
Table 9.6.3 CV Death (≤70 year old) 43
Table 9.6.4 CV Death (>70 year old) 43
Table 9.6.5 HHF (≤70 year old) 44
Table 9.6.6 HHF (>70 year old) 44
Table 9.6.7 MACE (CV death and HHF) (Male) 44
Table 9.6.8 MACE (CV death and HHF) (Female) 44
Table 9.6.9 CV Death (Male) 44
Table 9.6.10 CV Death (Female) 44
Table 9.6.11 HHF (Male) 44
Table 9.6.12 HHF (Female) 44
Table 9.6.13 MACE (CV death and HHF) (NYHA calss I and II) 44
Table 9.6.14 MACE (CV death and HHF) (NYHA calss III and IV) 44
Table 9.6.15 CV Death (NYHA calss I and II) 44
Table 9.6.16 CV Death (NYHA calss III and IV) 44
Table 9.6.17 HHF (NYHA calss I and II) 44
Table 9.6.18 HHF (NYHA calss III and IV) 44
Table 9.6.19 MACE (CV death and HHF)（LVEF≤30%） 45
Table 9.6.20 MACE (CV death and HHF)（LVEF>30%） 45
Table 9.6.21 CV Death（LVEF≤30%） 45
Table 9.6.22 CV Death（LVEF>30%） 45
Table 9.6.23 HHF（LVEF≤30%） 45
Table 9.6.24 HHF（LVEF>30%） 45
Table 9.6.25 MACE (CV death and HHF) (Course of heart failure ≤3 yr) 45
Table 9.6.26 MACE (CV death and HHF) (Course of heart failure >3 yr) 45
Table 9.6.27 CV Death (Course of heart failure ≤3 yr) 45
Table 9.6.28 CV Death (Course of heart failure >3 yr) 45
Table 9.6.29 HHF (Course of heart failure ≤3 yr) 45
Table 9.6.30 HHF (Course of heart failure >3 yr) 45
Table 9.6.31 MACE (CV death and HHF) (with ischemic heart disease) 45
Table 9.6.32 MACE (CV death and HHF) (without ischemic heart disease) 45

Table 9.6.33 CV Death (with ischemic heart disease) 46
Table 9.6.34 CV Death (without ischemic heart disease) 46
Table 9.6.35 HHF (with ischemic heart disease) 46
Table 9.6.36 HHF (without ischemic heart disease) 46
Table 9.6.37 MACE (CV death and HHF) (with cardiomyopathy) 46
Table 9.6.38 MACE (CV death and HHF) (without cardiomyopathy) 46
Table 9.6.39 CV Death (with cardiomyopathy) 46
Table 9.6.40 CV Death (without cardiomyopathy) 46
Table 9.6.41 HHF (with cardiomyopathy) 46
Table 9.6.42 HHF (without cardiomyopathy) 46
Table 9.6.43 MACE (CV death and HHF) (with hypertension) 46
Table 9.6.44 MACE (CV death and HHF) (without hypertension) 46
Table 9.6.45 CV Death (with hypertension) 46
Table 9.6.46 CV Death (without hypertension) 46
Table 9.6.47 HHF (with hypertension) 47
Table 9.6.48 HHF (without hypertension) 47
Table 9.6.49 MACE (CV death and HHF) (with diabetes) 47
Table 9.6.50 MACE (CV death and HHF) (without diabetes) 47
Table 9.6.51 CV Death (with diabetes) 47
Table 9.6.52 CV Death (without diabetes) 47
Table 9.6.53 HHF (with diabetes) 47
Table 9.6.54 HHF (without diabetes) 47
Table 9.6.55 MACE (CV death and HHF) (with atrial fibrillation) 47
Table 9.6.56 MACE (CV death and HHF) (without atrial fibrillation) 47
Table 9.6.57 CV Death (with atrial fibrillation) 47
Table 9.6.58 CV Death (without atrial fibrillation) 47
Table 9.6.59 HHF (with atrial fibrillation) 47
Table 9.6.60 HHF (without atrial fibrillation) 47
Table 9.6.61 MACE (CV death and HHF) (with arrhythmias) 48
Table 9.6.62 MACE (CV death and HHF) (without arrhythmias) 48
Table 9.6.63 CV Death (with arrhythmias) 48
Table 9.6.64 CV Death (without arrhythmias) 48
Table 9.6.65 HHF (with arrhythmias) 48
Table 9.6.66 HH (without arrhythmias) 48
Table 9.6.67 MACE (CV death and HHF) (NT-proBNP ≤Median) 48
Table 9.6.68 MACE (CV death and HHF) (NT-proBNP >Median) 48
Table 9.6.69 CV Death (NT-proBNP ≤Median) 48
Table 9.6.70 CV Death (NT-proBNP >Median) 48
Table 9.6.71 HHF (NT-proBNP ≤Median) 48
Table 9.6.72 HHF (NT-proBNP >Median) 48
9.7. EVALUATION OF SECONDARY EFFICACY INDICATORS (FAS&PPS) 49
Table 9.7.1 All-cause mortality 49

Table 9.7.2 Composite endpoint (consisting of worsening heart failure leading to withdrawal of treatment, successful resuscitation after cardiac arrest, malignant arrhythmia, and non-fatal stroke) 49
Table 9.7.3 Worsening heart failure leading to withdrawal of treatment 49
Table 9.7.4 Successful resuscitation after cardiac arrest 50
Table 9.7.5 Malignant arrhythmia 50
Table 9.7.6 Non-fatal stroke 51
Table 9.7.7 Cardiovascular death and hospitalization for heart failure in patients with ischemic heart disease 51
Table 9.7.8 Serum NT-proBNP level baseline and change from baseline (1month) 51
Table 9.7.9 Seurm NT-proBNP decrease rate from baseline (1 month) 52
Table 9.7.10 Seurm NT-proBNP decrease rate from baseline (3 months) 53
9.8. SAFETY EVALUATION (SS) 53
Table 9.8.1 Summary of Adverse Events 53
Table 9.8.2 Summary of adverse events 53
Table 9.8.3 Coding of adverse event (SOC/PT) 53
Table 9.8.4 Coding of adverse drug reaction (SOC/PT) 54
Table 9.8.5 Comparison of vital signs (12 months ± 7 days) - systolic blood pressure (mmHg) 54
Table 9.8.6 Comparison of vital signs (12 months ± 7 days) - diastolic blood pressure (mmHg) 55
Table 9.8.7 Comparison of vital signs (12 months ± 7 days) - heart rate (bpm) 55
Table 9.8.8 Comparison of vital signs (12 months ± 7 days) - pluse (bpm) 55
Table 9.8.9 Comparison of vital signs (12 months ± 7 days) – Body temperature (℃) 55
Table 9.8.10 Crosstabulation of Complete blood count - white blood cell count (12 months ± 7 days) 55
Table 9.8.11 Crosstabulation of Complete blood count - hemoglobin (12 months ± 7 days) 55
Table 9.8.12 Crosstabulation of Complete blood count - red blood cell count (12 months ± 7 days) 55
Table 9.8.13 Crosstabulation of Complete blood count - platelets (12 months ± 7 days) 55
Table 9.8.14 Summary of Complete blood count - 12 months ± 7 days (SS) 56
Table 9.8.15 Crosstabulation of biochemistry - Alanine aminotransferase (ALT) (12 months ± 7 days) 56
Table 9.8.16 Crosstabulation of biochemistry – Creatinine (Cr) (12 months ± 7 days) 56
Table 9.8.17 Crosstabulation of biochemistry – Blood Uear Nitrogrn (BUN) (12 months ± 7 days) 56
Table 9.8.18 Crosstabulation of biochemistry – Total cholesterol (TC) (12 months ± 7 days) 56
Table 9.8.19 Crosstabulation of biochemistry – Triglyciride (TG) (12 months ± 7 days) 56
Table 9.8.20 Crosstabulation of biochemistry – Potassium (K) (12 months ± 7 days) 56
Table 9.8.21 Crosstabulation of biochemistry – Sodium (Na) (12 months ± 7 days) 56
Table 9.8.22 Crosstabulation of biochemistry – Chlorine (Cl) (12 months ± 7 days) 56
Table 9.8.23 Crosstabulation of biochemistry – Fasting blood glucose (GLU) (12 months ± 7 days) 56
Table 9.8.24 Summary of Biochemistry - 12 months ± 7 days (SS) 57
Table 9.8.25 Crosstabulation of Urine routine – Urine protine (12 months ± 7 days) 57
Table 9.8.26 Crosstabulation of Urine routine – Urine white blood cell (12 months ± 7 days) 57
Table 9.8.27 Crosstabulation of Urine routine – Urine red blood cell (12 months ± 7 days) 57
Table 9.8.28 Summary of Urine routine - 12 months ± 7 days 57
Table 9.8.29 Crosstabulation of Physical examination – General condition (12 months ± 7 days) 58
Table 9.8.30 Crosstabulation of Physical examination – Nervous system (12 months ± 7 days) 58
Table 9.8.31 Crosstabulation of Physical examination – Skin, superficial lymph nodes (12 months ± 7 days) 58

Table 9.8.32 Crosstabulation of Physical examination – head and neck (12 months ± 7 days) 58
Table 9.8.33 Crosstabulation of Physical examination – Cardiovascular (12 months ± 7 days) 58
Table 9.8.34 Crosstabulation of Physical examination – Respiratory (12 months ± 7 days) 58
Table 9.8.35 Crosstabulation of Physical examination – Abdominal (12 months ± 7 days) 58
Table 9.8.36 Crosstabulation of Physical examination – Genitourinary (12 months ± 7 days) 58
Table 9.8.37 Crosstabulation of Physical examination – Spine and Limbs (12 months ± 7 days) 58
Table 9.8.38 Crosstabulation of Physical examination – Other (12 months ± 7 days) 58
Table 9.8.39 Summary of Urine routine - 12 months ± 7 days 58
Table 9.8.40 Crosstabulation of electrocardiogram (12 months ± 7 days) 59
Table 9.8.41 Summary of electrocardiogram - 12 months ± 7 days 59
9.9. LIST OF APPENDIXES 60

1. Abbreviations

ACEI	Angiotensin converting enzyme inhibitor
ADR	Suspected adverse drug reaction
AEs	Adverse events
AHA	American Heart Association
AMI	Acute myocardial infarction
ARB	Angiotensin receptor blocker
ARNI	Angiotensin receptor neprilysin inhibitor
BNP	B-type natriuretic peptide
CEA	Clinical Event Adjudication
CGRP	Calcitonin gene-related peptide
CHF	Chronic heart failure
CRA	Clinical research auditor
CRC	Clinical research coordinator
CRF	Case report form
CV	Cardiovascular
DSMC	Data and Safety Monitoring Committee
DVP	Data verification plan
ECG	Electrocardiogram
EF	Ejection fraction
EOS	Final visit
ESC	European Society of Cardiology
ET	Endothelin
FAS	Full analysis set
HF	Heart failure
IP	Investigational Product
LVOT	Left ventricular outflow
MedDRA	ICH International Medical Dictionary
MI	Myocardial infarction
NO	Nitric oxide

NT-proBNP	N-terminal pro brain natriuretic peptide
NYHA	New York Heart Association
PPS	Per protocol set
RAAS	Renin-angiotensin-aldosterone system
SAE	Serious adverse event
SNS	Sympathetic nervous system
SS	Safety analysis set
TVI	Time velocity integral
UNS	Unplanned visit

2. Study Title

Qiliqiangxin in Heart FailUre: AssESsment of Reduction in MorTality (QUEST study)

3. Study Objective

Using evidence-based medicine research methods, with cardiovascular mortality and hospital readmission rate for worsening heart failure as the main research endpoints, further elucidating the clinical efficacy and safety of long-term use of Qiliqiangxin capsules (QLQX), clarifying the characteristics of efficacy and the suitable population, to provide high-quality clinical evidence for optimizing HF therapy.

4. Study design

This study is a randomized, double-blind, placebo-controlled, parallel-group, multicenter clinical study.

The study will be event-driven, and all randomized patients will remain in the study (whether taking the study drug or not) until the number of primary endpoint events reaches the predicted value (620 cases), or the study terminates early when it meets the pre-defined efficacy or safety criteria of early termination.

Two mid-term efficacy analyses planned to be conducted after 1/2 and 2/3 primary endpoint events to assess whether an invalid or valid conclusion was reached so as to prematurely end the study.

The entire study will last approximately 36 months, and the recruitment period will be expected to be 24 months. The follow-up period after the last case of patient is included in the study is 12 months. The average follow-up time is predicted to be about 24 months.

Patients who show stable clinical symptoms, had received at least 2 weeks of standardized treatment and treatment of other concomitant diseases before enrollment are screened at the hospital. According to the local HF treatment guidelines, the drug type and dosage are fixed, unless it is contraindicated or intolerant. The patient who have not receive anti-HF drug intravenously for at least two weeks prior to enrollment, nor take oral administration of TCM or Chinese patent medicine having similar composition with Qiliqiangxin Capsule can directly enter the random grouping stage.

If patients fail to meet the above requirements, term standardized treatment to meet the above criteria before entering the random grouping stage are needed.

4.1. Control group and Sample size

According to the PARADIGM-HF study, the composite event of cardiovascular death and/or hospitalization for heart failure in the median follow-up of 27 months was 21.8% in the LCZ696 group and 26.5% in the Enalapril group. Therefore, we estimated that the incidence of cardiovascular death and hospitalization for heart failure is 25% in patients with standardized treatment + placebo group within 36 months of follow-up and 20% in standardized treatment + Qiliqiangxin capsule group.

The random distribution ratio is 1:1 between study group and control group. Considering the consumption of type I error in the interim analysis, α is adjusted to unilateral 0.02314. Based on the incidence of composite endpoint events is 25%, it is expected that 620 composite endpoint events need be observed to provide 80% power of test (β= 0.2), and 20% risk can be reduced in study group by log-rank test.

The entire study will last approximately 36 months to follow up, and the recruitment period are expected to be 24 months. The sample size is expected that 3,080 patients in over 100 centers (1540 patients per group) will be enrolled and be followed up at least for 12 months.

4.2. Blinding and Unblinding

4.3.1. Random grouping of subjects

Statistical experts at Peking University Clinical Research Institute adopts SAS 9.4 statistical software package to generate random numbers using the block randomization method according to the ratio of 1:1 between study group and control group. The study drug (Qiliqiangxin or placebo capsules) was packaged according to this random number by the person unrelated to the study.

A randomization and trail supply management system (RTSM) is used in the study, and statistical professionals will provide a random numbered list to the RTSM. The patient is then assigned a random number by the RTSM.

After completing baseline assessment, random numbers are assigned by RTSM during baseline visits. After that, the drug number is obtained through the RTSM according to the interview plan, and the serial number of drug assigned each time is different, but the drugs are the same. Before randomization of patient, the researcher must first log into the RTSM and provide the according information (e.g. the subject's date of birth, gender).

4.3.2. Unblinding and emergency unblinding protocol

If an adverse event occurs, emergency unblinding can only be conducted in special circumstances where it is necessary to understand the use of the investigational drug in order to treat the patient. Once the decision to unblind has been made, the researcher must record the date, time, and reason for unblinding.

The researcher needs to log in to the RTSM system to complete the unblinding application, which will be reviewed by the principal investigator before being unblinded by the unblinding officer. Once unblinded, the case will be discontinued from the study and treated as a withdrawal.

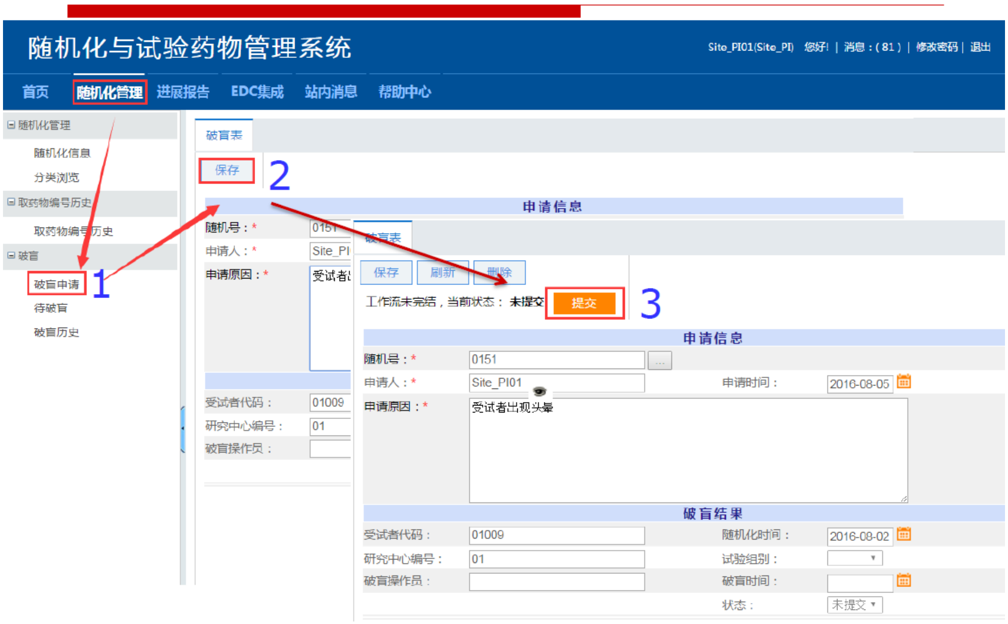

4.3.3. Unblinding provisions

All personnel involved with the analysis of the study will remain blinded until database lock and protocol violations have been identified and documented The study adopts two-step unblinding provision. After blind check, the data is locked, main researchers, medical statisticians, data administrators and sponsor representatives will do the first unblinding, and the random number corresponding to the group will be marked as A or B, in order to make statistical analysis on all data. At the end of statistical analysis when summary report is completed, a secondary unblinding would be taken to reveal the group of A and B.

4.3.4. Screening number

The screening number was made according to the sequence of patients taking treatment at each hospital, and represented with center ID + three integers, such as the screening number is 01001, 01002 ….. for center 01.

4.3. Treatment Plan

4.4.1. Study Process

Enrolment period (day -14 to day 0):

The investigators will review the inclusion and exclusion criteria. Patients who do not meet these criteria must not be randomized into the study. Set of assessments will be completed for these patients.

-Demography (date of birth, sex, race, ethnic group) and relevant medical and surgical history, including smoking history, will be recorded.

-General physical examination (vital signs, NYHA classification, appearance, cardiovascular systems [including edema], etc.)

-Laboratory samples will be collected and sent to the central laboratory

-ECG and echocardiogram will be recorded.

Randomization and Treatment period (day 0 to 12 months [with maximum of 36 months]):

The dispensing date of the IPs is regarded as day 0. Patients will be randomized into the study or control group in a 1:1 ratio with the basis of current standardized treatments for chronic heart failure. The study drug is recommended to be taken about 30 minutes after meals, three times a day as follow.

-Study group: Standardized treatment of chronic heart failure + Qiliqiangxin Capsules (4 capsules/time, 3 times/day);

-Control group: Standardized treatment of chronic heart failure + Placebo Capsules (4 capsules/time, 3 times/day);

This study does not allow dose adjustments. If the patient miss to take the IP in a day, the accumulated dose for the next day should not exceed the daily dose. If the patient has an intolerable adverse event, which is relevant to study drug according to the judgment of researcher, the patient should terminate the following treatment with the study drug.

Do not take drugs at home on the morning of visit day. Investigators will review laboratory results received from the past visit(s). If the patient has experienced any potential endpoints, SAEs, DAEs and/or AEs of interest since the last visit, these should be recorded in the CRF.

4.4.2. Concomitant medications and other treatments

All patients should be treated according to regional standard of care for HF and other comorbidity(s). Also, cardiac and heart failure related procedures will be captured during the study. Background medication will not be provided by the Sponsor.

1) Detailed recording of medications related to HF, HTN as well as other relevant cardiovascular medications (e.g., statins, antihypertensive and antithrombotic agents) will be made throughout

the study;

2) TCM and/or herbal medication have similar contents to the test drug should not be used during the entire treatment period after entering the randomization period.

3) Patients should receive dietary guidance for heart health, such as low-salt diet, moderate drinking, etc. Patients should also receive counseling for appropriate lifestyle improvements such as weight monitoring, physical exercise, smoking cessation, and alcohol withdrawal.

4) No drug has been found to be contraindicated with use of Qiliqiangxin Capsules.

Heart failure medications

Treatment of the patients will be based on the local or regional heart failure guidelines. The treatment regimen from the 2018 China heart failure diagnosis and treatment guideline are summarized as followed:

1) Stable heart failure symptoms without the use of intravenous diuretics, inotropic, and vasodilator for 2 weeks.

2) Patients received standardized baseline treatment regimens without doses adjusted at least two weeks prior to randomization. Standardized treatment includes: angiotensin-converting enzyme inhibitor (ACEI) or angiotensin receptor blocker (ARB) or angiotensin receptor neprilysin inhibitor (ARNI), beta blocker, and aldosterone receptor antagonist (the optimal therapeutic dose should be achieved, except for contraindications or intolerance).

3) After entering the treatment period, Dose reduction or discontinuation of proven effective therapies should be avoided unless all other measures fail to improve the patient’s situation. Any adjustment of the treatment regimen should be recorded in the CRF. If the patient has experienced any potential endpoints, SAEs, DAEs and/or AEs of interest since the last visit, these should be recorded in the CRF.

4.4.3. Adverse drug reaction

There is no significant adverse drug reaction of QLQX were found.

4.4.4. Evaluation on compliance

In order to determine the compliance of subjects, the administration (drug distribution and recovery) of all investigational products should be recorded in the appropriate sections of the eCRF. The actual dosage should be within 80%-120% of predefined dosage.

5. Rationale for study population

The enrolled patients should satisfy the following inclusion criteria, and not meet any exclusion criterion. In addition to following criteria, the patient should also be excluded if there is any contraindicated medical condition or use of incompatibility drug during basic treatment period.

5.1. Inclusion criteria

1) Signed informed consent;
2) Aged ≥18 years at the time of consent;
3) Established documented diagnosis of heart failure for at least three months ago according to "Chinese Heart Failure Diagnosis and Treatment Guideline" issued by the Chinese Medical Association Cardiovascular Branch;
4) Left ventricular ejection fraction (LVEF) ≤40% (echocardiogram, radionuclide, ventriculogram, contrast angiography or cardiac MRI);
5) NYHA cardiac functional grading II to III, with stable clinical symptoms; or those diagnosed as grade IV within 2 weeks before enrollment;
6) Serum NT-proBNP ≥450pg/ml;
7) Those who have received standardized baseline treatment regimens without doses adjusted and given intravenously for at least two weeks prior to enrollment; Standardized drug treatment includes angiotensin-converting enzyme inhibitor (ACEI) or angiotensin receptor blocker (ARB) or angiotensin receptor neprilysin inhibitor (ARNI), beta blocker, and aldosterone receptor antagonist (the optimal therapeutic dose should be achieved, except for contraindications or intolerance).

5.2. Exclusion criteria

1) Patients should not enter the study if any of the following exclusion criteria are fulfilled
2) Heart failure caused by valvular disease, congenital heart disease, pericardial disease,

arrhythmia or non-cardiaogenic disease, or caused by vital organ failure (such as renal, hepatic failure, etc.); and right heart failure caused by pulmonary or other definite causes; and acute heart failure;

3) Coronary revascularization (percutaneous coronary intervention [PCI] or coronary artery bypass grafting [CABG]) or cardiac synchronization therapy planned to undergo after randomization, or had received cardiac resynchronization therapy prior to enrolment;
4) Any condition outside the CV diseases such as but not limited to malignant tumor, severe mental illness, hematopoietic diseases, neuroendocrine system disease, liver transaminase and alkaline phosphatase ⩾ 3 x upper limit of normal (ULN), abnormal renal function serum creatinine > 2 mg/dl (176.82 umol/L), potassium >5.5mmol/L;
5) Patient with left ventricular outflow tract obstruction, myocarditis, aortic aneurysm, aortic dissection, or obvious hemodynamic changes caused by unrepaired valve;
6) Cardiogenic shock, uncontrollable malignant arrhythmia, sinus or atrioventricular block at second degree type II or above without pacemaker treatment, progressive unstable angina pectoris or acute myocardial infarction;
7) uncontrolled hypertension systolic blood pressure (SBP) ⩾ 180mmHg and/or diastolic blood pressure (DBP) ⩾ 110mmHg; or SBP < 90mmHg and/or DBP <60mmHg;
8) Participation in another clinical study with an IP during the last month prior to enrolment;
9) Women of child-bearing potential (i.e., those who are not chemically or surgically sterilized or who are not post-menopausal) who are not willing to use a medically accepted method of contraception that is considered reliable in the judgment of the investigator, from the time of signing the informed consent throughout the study and 4 weeks thereafter, OR women who have a positive pregnancy test at enrolment or randomisation OR women who are breast-feeding;
10) Allergic constitution; known to be allergic to research drug;
11) Inability of the patient, in the opinion of the investigator, to understand and/or comply with study medications, procedures or any conditions may render the patient unable to complete the study.

5.3. Patient enrolment and Follow-up visit

Patients who fail to meet the eligibility criteria should not, under any circumstances, be enrolled or receive study medication. Patient should receive at least 2 weeks of standardized treatment. Patient should not receive other traditional Chinese medicine or Chinese patent medicine (with similar function and composition with Qiliqiangxin Capsules).

Patients meet the inclusion criteria can enter the randomization. During this period, the drug and dosage are fixed for each patient. If it is necessary to make adjustment, it should be recorded in the case report form (CRF).

Patients should visit the hospital for efficacy and safety assessment during the 1st, 3rd, 6th, 9th, and 12th months after the random grouping until the study finish.

5.4. Discontinuation of investigational product (IP)

At any time after randomization, patients are free to discontinue for any reason. Discontinuation from IP is not the same as complete withdrawal from the study. According to the intention-to-treat principle, patients discontinuation from IP will be continue in follow-up and recorded in CRF. For patients could not participate in outpatient follow-up, virtual interview and follow-up according to plan will be proceeded for the assessment of the adverse events and/or endpoint events unless the patient refuses to follow up and withdraw from the study. Study drug treatment can be discontinued when:

1. The patient can stop treatment at any time;
2. The patient has allergic reactions that are clearly associated with the IP;
3. The patient has occurrence of symptoms, signs and/or abnormal examination results that are related to the IP, or the condition determined by the investigator to terminate the study;
4. Pregnancy during the study;

During the trial, the patient should take the standard dose of the IP as long as possible. The patients should resume taking the IP as soon as possible after the relevant causes are excluded and follow up as planned.

5.5. Withdrawal

The patient has the right to withdraw from the study at any time for any reason. The withdrawal of the trial will not intervein the regimen and management.

The researcher should retrieve the remaining IP when the patient withdraws from study. The reason for the withdrawal should be recorded in CRF by follow-up interview or telephone. Follow-up should be continued in order to ascertain whether any endpoints or safety events have occurred. Optimally, patients who discontinue from IP should continue to attend all study visits according to plan until study finish as much as possible. Information should be recorded in CRF.

5.6. Discontinuation of the study

1) The overall study may be stopped due to the following reasons:
   - Base on Data Safety Monitoring Committee (DSMC) interim analysis results;
   - Researchers find serious safety problems;
   - Major mistakes in the study protocol;
   - The sponsors decide to suspend study due to management problems or lack of funding;
   - The competent administrative department cancels the experiment, and half-stops all studies.
2) The discontinuation of the study can be temporary or permanent. During the suspension, all study records should be kept for inspection.

6. Clinical observation endpoints and indicators

6.1. Clinical observation endpoints

6.1.1. Primary outcome measure

- The composite endpoint events consisting of cardiovascular death and/or hospitalization for heart failure;

6.1.2. Secondary outcome measures

- All-cause mortality
- Secondary endpoint events (given up treatment due to worsening heart failure, successful resuscitation after cardiac arrest, malignant arrhythmia, non-fatal stroke)
- Components of the primary endpoints in patients with ischemic heart disease

- Level of Serum NT-proBNP

Note: All endpoint events should be determined and reviewed by Clinical Event Adjudication Committee.

6.2. Safety outcome measure:

- Adverse events (Serious Adverse Events [SAEs], Discontinuation of IP due to Adverse Events, etc.)
- Clinical laboratory indexes: complete blood count (hemoglobin, red blood cells, white blood cells, platelets), routine urine test (urinary protein, urinary white blood cells, urine red blood cells), serum biochemistry (urea nitrogen, creatinine, alanine aminotransferase, fasting blood glucose, potassium, sodium, chlorine, total cholesterol, triglycerides).
- 12-lead ECG
- Physical examination

6.2.1. Definition of Adverse Event

- Adverse events (AE): AE refers to any adverse medical events occurring in this clinical experiment from the moment that the patient signs the informed consent and is chosen to participate in this study to the last follow-up, whether or not the events are caused by the use of the medicine described.

6.2.2. Criteria on severity of adverse events:

All clinical adverse events that occur in this clinical study will be recorded on the CRF adverse event page. The severity of adverse events will be classified. For uniform standards, the severity of events is classified as follows:

Grade 1: Mild, no clinical symptoms or mild clinical symptoms; only clinical or laboratory abnormalities; no treatment required.

Grade 2: Moderate, requiring minimal, local, or non-invasive treatment; daily life activities using age-appropriate tools a are restricted, such as cooking, shopping, and making phone calls.

Grade 3: Severe illness or severe medical symptoms that are temporarily not life-threatening; resulting in hospitalization or prolonged hospital stay; resulting in disability; restricted in daily

living activities b. Activities of daily living refer to bathing, dressing, undressing, eating, using the toilet, taking medication, and being non-bedridden.

Grade 4: Life-threatening, requiring emergency treatment.

Grade 5: Death due to adverse events.

a: Daily living activities with refer to cooking, buying daily necessities or clothes, using the phone, managing finances, etc.

b: Daily living activities refer to bathing, dressing/undressing, eating, grooming, taking medication, and not being bedridden.

6.2.3. Adverse events of interest

Criteria for assessing the relationship between adverse events and investigational drugs. All causal analyses of adverse events related to investigational drugs are assessed according to five levels: definite, possible, unlikely, definite unrelated, and uncertain. The first three are considered adverse drug reactions. There are five main considerations for causal analysis:

1) Definite: There is a reasonable temporal sequence between the occurrence of the AE and the use of the investigational drug. The AE is a known adverse reaction to the investigational drug. The AE is alleviated or disappears upon discontinuation of the investigational drug, and reoccurs upon repeated use. It cannot be explained by the subject's underlying disease.

2) Possible: There is a reasonable temporal sequence between the occurrence of the AE and the use of the investigational drug. The AE is a known or suspected adverse reaction to the investigational drug, but there may be other factors that could cause the event, such as disease or concurrent medication. The AE is alleviated or disappears upon discontinuation of the investigational drug, or the effect of discontinuation on the event is unclear, or there is a lack of decisive information.

3) Unlikely: There is a reasonable temporal sequence between the occurrence of the AE and the use of the investigational drug, but it is not a known adverse reaction to the investigational drug type, and it is highly likely to be caused by the subject's illness or other treatment.

4) Definite unrelated: There is no reasonable temporal sequence between the AE and the use of the investigational drug, such as events that occurred before the use of the investigational drug,

or events that are not known adverse reactions to the investigational drug, or events that are clearly caused by other factors such as the subject's underlying disease or concurrent medication.

5) Uncertain: There is no clear relationship between the timing of the AE and the medication, and the known type of adverse reaction is similar to the investigational drug. Other co-administered drugs may cause the same reaction, and there is not enough evidence to make a clear decision.

The "definite," "possible," and "uncertain" categories are combined to calculate the incidence of adverse reactions to the investigational drug.

6.2.4. Definition serious adverse events

Serious adverse events (SAE) refer to any clinical events that indicate significant harm, contraindications, adverse reactions, or the need for caution. Adverse events meet the criteria for SAE when they meet one or more of the following standards:

- Death
- Life-threatening (referring to the immediate risk of death for the patient at the time of the event; this does not include events that may lead to death if they become more serious)
- Result in hospitalization or prolonged hospital stay
- Result in persistent or significant work loss or disability
- Congenital malformations or defects

Other events that have not resulted in death, life-threatening situations, or the need for hospitalization but are considered to be harmful to patients or subjects, or require drug or surgical treatment to avoid the above situations upon appropriate medical judgment, should also be considered as SAE.

6.2.5. Definition on specificity of serious adverse events

In this study, the following events will not be reported as serious adverse events unless they are judged negative and the researcher believes that it is related to the study drug

- ◆ Cardiovascular death
- ◆ Hospitalization for heart failure

- Given up treatment due to worsening heart failure
- Successful resuscitation after cardiac arrest
- Malignant arrhythmia
- Non-fatal stroke

Other events leading to fatal outcomes should be reported as serious adverse events.

6.2.6. Recording of adverse events and follow-up

If any adverse events occurred, especially those associated with the study drug, should be followed up until the patients return to baseline or tend to stabilize. If the baseline status or stability cannot be restored after follow-up, it should be noted in the CRF. All SAEs must be reported within 24 hours, whether or not considered causally related to the investigational product, or to the study procedure(s). At the same time, researchers must complete a serious adverse event form, recording the time, severity, duration, measures taken, and outcome of serious adverse events.

6.2.7. Adverse events based on examinations

The results from protocol mandated laboratory tests and vital signs will be summarized and give possible interpretation. The abnormal laboratory results caused by reported adverse events should be recorded in the adverse event form. The abnormal results with clinical significance that meets one or more following conditions should be recorded as independent diagnosis on adverse event page of CRF (excluding abnormal laboratory result caused by reported adverse events):

- With associated clinical signs and symptoms
- Change in course of study drug's treatment dose
- Change in any of standard evidence-based medications and (or) other treatment measures need to be changed.

6.3. Efficacy Assessments

6.3.1. Endpoint reporting overview

When potential endpoint events have been identified, the researchers should collect all relevant support documents within 7 days and report to CEA committee. Investigators will record the incident in endpoint report form and submit supporting data in a timely manner (admission and discharge records, medical records, death records, ECG, etc.). The potential endpoints event

(All deaths, All HF events [hospitalizations for HF or urgent HF visits], cardiac ischemic events [MI and unstable angina], cerebrovascular events [stroke and TIA], etc.) will be reviewed for central CEA process.

CEA committee consists of a chairman and 5-6 members. Each case will be independently reviewed by two members of the committee. The conclusions will be submitted to the chairman of the committee.

6.3.2. Potential endpoint events

For each potential endpoint event, the investigator or delegate will record information in the CRF

- **Hospitalization for heart failure:**

1. The patient was hospitalized for HF diagnosed preliminarily;
2. The patients who were admitted in hospital extended for at least 24 hours (or if the hospitalization time and discharge time were not available, it should indicate change of calendar date);
3. Record on the patient report that there are new symptoms or worsening symptoms due to HF, including at least one of the following:

a. Difficulty breathing (difficulty breathing on exertion, difficulty breathing on resting, orthopnea, paroxysmal breath with difficulty at night)

b. Reduced exercise tolerance

c. Fatigue

4. The patient had objective evidence of an acute exacerbation of HF, including at least two health examination results or a health examination result and at least one laboratory standard, including:

a. Determine the health examination results caused by HF, including new or deteriorated:

1) Peripheral edema; 2) Abdominal distension or increase of ascetic fluid (in the absence of primary liver disease); 3) Lung rales and/or crackles; 4) Increased jugular venous pressure and/or hepatojugular reflex (+); 5) S3 galloping; 6) Clinically significant or rapid weight gain, having relation with fluid retention.

b. Laboratory evidence of new or worsening HF obtained within 24 hours, including:

1) Increased concentration of B-type natriuretic peptide (BNP) / N-terminal B-type natriuretic peptide precursor (NT-proBNP) consistent with acute decompensated HF (eg.: BNP > 500 pg/ml or NT-proBNP > 2000 pg/ml); In patients with long-term elevation of natriuretic peptides, special attention should be paid to a significant increase beyond baseline. 2) Imaging evidence of pulmonary congestion; 3) Non-invasive diagnostic evidence of clinically significant increase in left or right ventricular filling pressure or decreased cardiac output; echocardiographic criteria includes: E/e´>15 or D leading pulmonary venous inflow pattern, congestive inferior vena cava with minimal inspiratory collapse, or reduction of small stroke distance (time velocity integral; TVI) at left ventricular outflow (LVOT). 4) Invasive diagnostic evidence: right heart catheterization showed pulmonary capillary wedge pressure (pulmonary artery wedge pressure) ≧18mmHg, central venous pressure ≧ 12mmHg, or cardiac output index <2.2L/min/m2;

Note: If applicable, all results in the diagnostic test need to be recorded; even if the above criteria are not met, results might provide important information for the determination of the above events.

5. The patients receive an initial or intensive treatment for HF, including at least one of the following:

a. Enhance the treatment of oral diuretics;

b. Intravenous diuretics or vasoactive drugs (such as inotropics, vasopressors or vasodilators);

c. Mechanical or surgical intervention, including:

1) Mechanical circulation support (e.g.: Intra-aortic balloon pump, ventricular assist device, extracorporeal membrane oxygenation, total artificial heart);

2) Mechanically assisted removal of body fluids (e.g.: ultrafiltration, hemofiltration, and dialysis).

- **Cardiovascular death: i**ncluding death caused by acute myocardial infarction (AMI), sudden

cardiac death, acute decompensated heart failure, stroke, cardiovascular (CV) surgery, CV bleeding, and other CV inducing death;

- **All-cause mortality**
- **Given up treatment due to the worsening of HF:** Worsening of heart failure symptoms and signs, requiring intravenous drug or mechanical support treatment, and patients or family members voluntarily give up treatment or left hospital without cure; if the result of follow-up is death, it is included in heart failure death.
- **Successful resuscitation after cardiac arrest**
- **Malignant arrhythmia:** There is no uniform standard for the definition of malignant arrhythmia. It generally refers to arrhythmia that can cause severe hemodynamic disorder in a short period of time, causing syncope or even sudden death. According to this standard, malignant arrhythmia mainly has the following categories: (1) severe bradyarrhythmia, such as severe sick sinus syndrome, high or third degree atrioventricular block; (2) tachyarrhythmia, such as persistent ventricular tachycardia, ventricular flutter, ventricular fibrillation, atrial flutter/atrial fibrillation with rapid ventricular rates, atrioventricular reentry tachycardia, pre-excitation syndrome with atrial fibrillation, sinus tachycardia, etc.
- **Non-fatal stroke**

7. Data Management

This study used Epidata software to collect research data. Data management ensures the authenticity, integrality and accuracy of clinical data. The data management process needs to comply with the regulatory requirements of Clinical Trial Quality Management Regulations and Clinical Trial Data Management Work Technical Guidelines, in order to ensure traceability of study data. The main processes for data management are listed below.

7.1. Database Design

The data administrator adopts the Epidata software to design and release database according to the CRF after testing.

7.2. Data entry

Clinical research coordinator (CRC) is responsible for inputting the CRF data into the database. The data entry adopts secondary recording mode. Two CRC respectively input the data. Data administrator compares the two databases to generate the data inconsistency list. CRC modified the databases respectively according to the list and the CRF, and then made comparison again. The above steps are repeated until the two databases being identical.

7.3. Data questioning management

The data administrator wrote data verification SAS program according to the data verification plan (DVP) to verify and generated a data questioning list. The data questioning table would be generated after manual verification, and clinical research auditor (CRA) gives the data questioning table to the researcher for answer. After the researcher answering the question, CRA returned the data questioning table to data administrator and revised the database accordingly.

7.4. Medical coding

The medical coding of adverse events is done according to MedDRA 21.0 or advance version.

7.5. Data audit

After completion of database cleanup, the data administrator should write Data Verification Report for holding a data verification meeting.

The major recording contents of the audit report: number of enrolled cases, the condition of off cases and exclusion cases, the condition of deviation or violation from the program, compliance data, drug combination, adverse events, data related to the evaluation indicators, etc.

At the data audit meeting, the division of statistical population is discussed and determined according to the content of audit report.

7.6. Database locking

Complete the database locking list and lock the database according to the database locking program. Any issues discovered after locking the data can be corrected in the statistical analysis program once confirmed. If there is solid evidence showing that it is necessary to unlock the data, the researchers and related personnel must sign an unlocking document.

After the database is locked, the data administrator exports the data in SAS format and hands it over to the statistical personnel for statistical analysis.

8. Statistical Analysis Method

After the research protocol is established, the statistical professionals, in collaboration with the primary researchers, will develop a statistical analysis plan. SAS®9.4 software or a higher version will be used for statistical analysis, and PASS13 was used for sample size calculations.

8.1. Definitions of analysis sets

The study population will be divided into the following categories:

- Full Analysis Set (FAS): This refers to the dataset that includes all randomized subjects who received the study drug at least once, with minimal and reasonable exclusion of subjects adhering to the intention-to-treat principle. Exclusions typically include: important inclusion criteria violations, subjects who did not receive the study drug treatment, and subjects with no post-randomization observation data. The primary efficacy endpoint is the time to composite endpoint event, analyzed using survival analysis methods. In selecting FAS for statistical analysis, missing data for the primary endpoint event will be handled by deletion.
- Per Protocol Set (PPS): This is a subset of FAS, in which the subjects have better adherence to the protocol. Subjects included in PPS typically have the following characteristics: (1) completed the minimum exposure to the study drug as predetermined in the protocol, with adherence to drug administration of at least 80%; (2) data for the primary endpoint event are available; and (3) no significant protocol violations.
- Safety Set (SS): This includes all randomized subjects who received at least one treatment and have safety evaluations. No imputations will be made for safety data.

Efficacy analysis will be based on FAS and PPS, while safety analysis will be based on SS. Baseline demographics will be analyzed based on FAS.

8.2. General Statistical methods

Efficacy analysis is taken on the basis of FAS and PPS. All baseline demographic data analysis will be performed on the basis of FAS and safety evaluation on SS.

- All data are performed with two-sided test, and P value less than 0.05 (two-sided test) is considered with statistically significant (If no other specification).
- Descriptive analysis: The number of subjects, mean, median, standard deviation, the first quartile (Q1), the third quartile, minimum, and maximum will be included in the summary statistics of continuous variables. The number of subjects, frequency and percentages will be included in summary statistics of the categorical variables.
- Comparison of general situation should be analyzed with appropriate method based on the type of variables . Continuous variables should be analyzed with paired t-test or Wilcoxon rank-sum test , as appropriate. Categorical variables will be compared with chi-square test or Fisher's

exact test, as appropriate. Ranked variables will be compared with Wilcoxon rank-sum test or CMH test.

8.2.1. Enrollment and completion analysis

The number of subjects enrolled and completed at each center will be summarized, and a list of withdrawal will be provided. Comparisons of group sizes, distribution of cases at each center, dropout rates, and detailed termination reasons will be presented. The demographic characteristics of patients (age, height, vital signs, etc.), medical history, and medication history will be described. The comparability of the two groups in terms of age, height, weight, etc. will also be assessed.

8.2.2. Compliance analysis:

- Medication adherence will be compared between the two groups to evaluate whether the study drug was used on time and in the correct dosage, without using any prohibited drugs or food.
- Co-medication usage will be counted for each group and listed in detail.

8.2.3. Efficacy analysis:

- PP analysis and FAS analysis were performed simultaneously with efficacy evaluation;
- Main efficacy evaluation index is the time when a composite endpoint event (cardiovascular death and hospitalization for deterioration of heart failure) occurs. The lack of primary endpoint events is consodered as censored data.

  The main research hypotheses are as following:

  H_0: $\lambda_T / \lambda_C \geq 1$

  H_1: $\lambda_T / \lambda_C < 1$

λ_T and λ_C represent the risk of the primary endpoint events in the study group and control group respectively. The Kaplan-Meier method will be used to estimate the incidence of clinical endpoints, and a Log-rank test will be performed between the two groups. The Hazard Ratio and its 95% confidence interval between the two groups will be calculated by COX Proportional Hazard Model with center as the covariate. At the same time, two components of the composite endpoint event, the cardiovascular death and hospitalization due to the worsening of heart failure will be analyzed separately.

- Secondary efficacy indicators:

- All-cause Mortality: The Kaplan-Meier method will be used to estimate the all-cause mortality rates of the two groups, and the Log Rank test will be used for statistical comparison. The COX proportional hazard model will be utilized to estimate the hazard ratios (HRs) and their 95% confidence intervals (CIs) with center as a covariate.
- Composite endpoint (consisting of worsening heart failure leading to withdrawal of treatment, successful resuscitation after cardiac arrest, malignant arrhythmia, and non-fatal stroke): Analyzed using the same method as all-cause mortality.
- Cardiovascular death and hospitalization for heart failure in patients with ischemic heart disease: Analyzed as all-cause mortality.
- Serum NT-proBNP level: Analyzed using measurement data, with statistical description and inter-group comparison for the two groups' baseline and change from baseline.

8.2.4. Safety analysis:

Safety analysis will be based on SS.

Data of adverse events (case number, times and incidence of various adverse events) are compared between the two groups. At the same time, detailed description of specific manifestation, extent of all adverse events and the relation with drugs would be further analyzed.

Crosstab is adopted to describe the change of laboratory index. Number of normal cases before treatment, number of abnormal cases after treatment and ratio of abnormal cases are analyzed in study group and control group. Indexes of vital signs are compared between before and after treatment.

8.3. Interim analysis

The study plans to perform two interim efficacy analyses after collecting 1/2 and 2/3 of the primary endpoints to assess whether a valid conclusion has been reached and then terminate the study early. According to Lan-DeMets α spending function and the O'Brien-Fleming method, the spending type I error was $\alpha= 0.0001$ (one-sided) in the first interim analysis period, and $\alpha= 0.00605$ (one-sided) in the second interim analysis period.

The specific requirements and operations related to the interim analysis will be specified in the DSMB in advance.

9. Statistical Analysis Results

9.1. Case distribution (for all randomized patients)

Table 9.1.1 Distribution of cases in each centerDefinitions of analysis sets

Centers	Group	Enrollment	Dropout	Dropout Rate (%)	Elimination	Elimination Rate (%)	Complet
The First Affiliated Hospital	Study						
With Nanjing Medical University	Placebo						
	Total						
Hebei Yiling Hospital	Study						
	Placebo						
	Total						
……	Study						
	Placebo						
	Total						
Overall	Study						
	Placebo						
	Total						

Table 9.1.2 Comparison of dropout and elimination between two groups

Group	Dropout Rate (%)	P	Elimination Rate (%)	P
QLQX				
Placebo				

Note: Dropout refers to subjects who did not complete the trial, and elimination refers to subjects who completed the trial but were excluded from the PPS set.

Table 9.1.3 Population in each center

Centers	PPS			FAS			SS		
	Study	Placebo	Total	Study	Placebo	Total	Study	Placebo	Total
The First Affiliated Hospital With Nanjing Medical University									
Hebei Yiling Hospital									
……									
Overall									

Note: The center of the following table is indicated by the center number

Table 9.1.4 Enrolled cases and safety and efficacy analysis data sets

Study Set	Study Group	Placebo Group	Total
Full analysis set (FAS)			
Randomizied entry			
Complet Trial			
Discontinuation during the trial			
Eliminated			
Adverse events			
Lack of efficacy			
Protocol violations (including poor compliance)			
Withdrawal of informed consent			
Lost to follow-up			
Other reasons			
Safety Analysis Set			
Safety set (SS)			
Efficacy Analysis Sets			
FAS			
Per Protocol Set (PPS)			

9.2. Demographic and Baseline Information (FAS)

Table 9.2.1 Demographic

Item	Index	Study Group	Placebo Group	Total	Statistics	P
Age, yrs	N(Missing)					
	Mean(Sd)					
	Median					
	Q1,Q3					
	Min,Max					
Sex	Male, n (%)					
	Female, n (%)					
	Total (Missing)					
Marriage	Unmarried, n(%)					
	Married, n(%)					
	Other, n(%)					
	Total (Missing)					
Ethnicity	Han, n (%)					
	Other, n (%)					
	Total (Missing)					
Height (cm)	N(Missing)					
	Mean (Sd)					
	Median					
	Q1, Q3					
	Min, Max					
Weight (kg)	N(Missing)					
	Mean (Sd)					
	Median					
	Q1, Q3					
	Min, Max					
BMI(kg/m^2)	N(Missing)					
	Mean (Sd)					
	Median					
	Q1, Q3					
	Min, Max					
Nationality	Chinese, n (%)					
	Other, n (%)					
	Total (Missing)					

Table 9.2.2 Personal History

Item	Index	Study Group	Placebo Group	Total	Statistics	P
Smoking	Yes n(%)					
	No n(%)					
	Quitted n(%)					
	Total (Missing)					

Item	Index	Study Group	Placebo Group	Total	Statistics	P
Dayily Cigarettes	N(Missing)					
	Mean(Sd)					
	Median					
	Q1,Q3					
	Min,Max					
Alcohol Consumption	Yes n(%)					
	No n(%)					
	Quitted n(%)					
	Total (Missing)					
Drinking Quantity (Unit/ 50g*d)	N(Missing)					
	Mean(Sd)					
	Median					
	Q1,Q3					
	Min,Max					
Menstration	Non-menopausal n(%)					
	Menopausal n(%)					
	Not applicable n(%)					
	Total (Missing)					
History of allergy	Yes n(%)					
	No n(%)					
	Total (Missing)					

Table 9.2.3 Comorbidities and past medical history

Item	Index	Study Group	Placebo Group	Total	Statistics	P
Ischemic heart disease	No n(%)					
	Yes n(%)					
	Total (Missing)					
Cardiomyopathy	No n(%)					
	Yes n(%)					
	Total (Missing)					
Hypertension	No n(%)					
	Yes n(%)					
	Total (Missing)					
Other	No n(%)					
	Yes n(%)					
	Total (Missing)					
Heart failure course	≤3 year					
	>3 year					
	Total (Missing)					
Heart failure course (months)	N(Missing)					
	Mean(Sd)					
	Median					

Item	Index	Study Group	Placebo Group	Total	Statistics	P
	Q1,Q3					
	Min,Max					

Table 9.2.4 Baseline of past medical history

Item	Index	Study Group	Placebo Group	Total	Statistics	P
Myocarial Infraction	No n(%)					
	Yes n(%)					
	Total (Missing)					
Hypertension	No n(%)					
	Yes n(%)					
	Total (Missing)					
Diabetes	No n(%)					
	Yes n(%)					
	Total (Missing)					
Hyperlipidemia	No n(%)					
	Yes n(%)					
	Total (Missing)					
Stroke	No n(%)					
	Yes n(%)					
	Total (Missing)					
Atrial fibrillation	No n(%)					
	Yes n(%)					
	Total (Missing)					
Arrhythmia	No n(%)					
	Yes n(%)					
	Total (Missing)					

Note: Medical history is categorized by the corresponding coding.

Table 9.2.5 Analysis of past medical history (SOC/PT)

	Study Group			Placebo Group			
Item	Incident	Patients	N (%)	Incident	Patients	N (%)	P
Past medical history							

Table 9.2.6 Analysis of major past medical history（PT）

	Study Group			Placebo Group			
Item	Incident	Patients	N (%)	Incident	Patients	N (%)	P
Hypertension							
DIabetes							

Item	Study Group Incident	Study Group Patients	Study Group N (%)	Placebo Group Incident	Placebo Group Patients	Placebo Group N (%)	P
Hyperlipidemia							
Atrial fibrillation							
Myocarial Infraction							
Stroke							
Arrhythmia							

The above medical histories are classified according to coding.

Table 9.2.7 NYHA heart functional classification

Item	Index	Study	Placebo	Total	Statistics	P
NYHA class	Class I, No limitation of physical activity;※Should not be included; n(%)					
	Calss II, Slight limitation of physical activity (fatigue, dyspnea); n(%)					
	Class III, Marked limitation of physical activity; n(%)					
	Class IV, Unable to carry on any physical activity;※inclusion assessment					
	Total (Missing)					

Table 9.2.8 Echocardiogram

Item	Index	Study	Placebo	Total	Statistics	P
Underwent examination	Yes n(%)					
	No n(%)					
	Total (Missing)					
LVEF (%)	N(Missing)					
	Mean(Sd)					
	Median					
	Q1,Q3					
	Min,Max					

Table 9.2.9 Serum NT-proBNP

Item	Index	Study	Placebo	Total	Statistics	P
Testing on Visit 1	Yes n(%)					
	No n(%)					
	Total (Missing)					
NT-proBNP (pg/ml)	N(Missing)					

Item	Index	Study	Placebo	Total	Statistics	P
	Mean(Sd)					
	Median					
	Q1,Q3					
	Min,Max					
Testing on Visit 2	Yes n(%)					
	No n(%)					
	Total (Missing)					
NT-proBNP (pg/ml)	N(Missing)					
	Mean(Sd)					
	Median					
	Q1,Q3					
	Min,Max					
Testing on Visit 3	Yes n(%)					
	No n(%)					
	Total (Missing)					
NT-proBNP (pg/ml)	N(Missing)					
	Mean(Sd)					
	Median					
	Q1,Q3					
	Min,Max					

Table 9.2.10 Vitals

Item	Index	Study	Placebo	Total	Statistics	P
SBP (mmHg)	N(Missing)					
	Mean(Sd)					
	Median					
	Q1,Q3					
	Min,Max					
DBP (mmHg)	N(Missing)					
	Mean(Sd)					
	Median					
	Q1,Q3					
	Min,Max					
Heart rate (bpm)	N(Missing)					
	Mean(Sd)					
	Median					
	Q1,Q3					
	Min,Max					
Pluse (bpm)	N(Missing)					
	Mean(Sd)					
	Median					
	Q1,Q3					
	Min,Max					
Temperature (°C)	N(Missing)					
	Mean(Sd)					
	Median					

Item	Index	Study	Placebo	Total	Statistics	P
	Q1,Q3					
	Min,Max					

9.3. Follow up period

Table 9.3.1 Follow time descriptions

		FAS		PPS	
Item	Index	Study	Placebo	Study	Placebo
Follow-up (Day)	N(Missing)				
	Mean(Sd)				
	Median				
	Q1,Q3				
	Min,Max				
Comparsion	Statistics				
	P				

Table 9.3.2 Median follow time

		FAS		PPS	
Item	Index	Study	Placebo	Study	Placebo
Follow up	N(Missing)				
	Endpoint event (%)				
	Censored (%)				
	Median				
	95%CI				
	Q1,Q3				
	Log-rank test				
	P				
Cox regression	Hazard Ratio				
	95%CI				
	Wald χ^2				
	P				

9.4. Medication (SS)

Table 9.4.1 Medication compliance

Item	Index	Study	Placebo	Total	Statistics	P
Medication	<80% n(%)					
Compliance	80%-120% n(%)					
	>120% n(%)					
	Total (Missing)					

Note: The actual dose of drugs taken is within the range of 80% to 120% of the amount of drugs used, which can be judged as compliance with the protocol requirements.

Table 9.4.2 Past Medication/Concomitant Medication

Item	Index	Study	Placebo	Total	Statistics	P
Past Medication/	No n(%)					
Concomitant Medication	Yes n(%)					
	Total (Missing)					

Table 9.4.3 Coding of Concomitant Medication (ATC1/ATC2)

	Study Group			Placebo Group			
Item	Incident	Patients	N (%)	Incident	Patients	N (%)	P
ATC1							
ATC2							

Table 9.4.4 Usage of Spironolactone, ARNi, SGLT2i, and cardiac glycoside

Item	Index	Study	Placebo	Total	Statistics	P
Spironolactone	No n(%)					
	Yes n(%)					
	Total (Missing)					
ARNi	No n(%)					
	Yes n(%)					
	Total (Missing)					
SGLT2i	No n(%)					
	Yes n(%)					
	Total (Missing)					
Cardiac glycoside	No n(%)					
	Yes n(%)					
	Total (Missing)					

Table 9.4.5 Triple therapy（RAAS+Beta+MRA）

Item	Index	Study	Placebo	Total	Statistics	P
RAAS+Beta+MRA	No n(%)					
	Yes n(%)					
	Total (Missing)					

9.5. Evaluation of primary efficacy indicators (FAS&PPS)

Table 9.6.1 Major adverse cardiovascular events (MACE, CV death and HHF)

		FAS		PPS	
Item	Index	Study	Placebo	Study	Placebo
MACE time	N(Missing)				
	Endpoint event (%)				
	Censored (%)				
	Median				
	95%CI				
	Q1,Q3				
	Log-rank test				
	P				
Cox Regression	Hazard Ratio				
	95%CI				
	Wald χ^2				
	P				

*Cox regression covariates is center, the same below。

Figure 1 Kaplan-Meier analysis of MACEs (FAS)

Figure 2 Kaplan-Meier analysis of MACEs (PPS)

Table 9.6.2 Cardiovascular death

		FAS		PPS	
Item	Index	Study	Placebo	Index	Placebo
CV Death	N(Missing)				
	Endpoint event (%)				
	Censored (%)				
	Median				
	95%CI				
	Q1,Q3				
	Log-rank test				
	P				
Cox Regression	Hazard Ratio				
	95%CI				
	Wald χ^2				
	P				

Figure 3 Kaplan-Meier analysis of Cardiovascular death (FAS)

Figure 4 Kaplan-Meier analysis of Cardiovascular death (PPS)

Table 9.6.3 Hospitalization of heart failure

		FAS			PPS	
Item	Index	Study	Item	Index	Study	
Hospitalization of heart failure	N(Missing)					
	Endpoint event (%)					
	Censored (%)					
	Median					
	95%CI					
	Q1,Q3					
	Log-rank test					
	P					
Cox Regression	Hazard Ratio					
	95%CI					
	Wald χ^2					
	P					

Figure 5 Kaplan-Meier analysis of Hospitalization of heart failure (FAS)

Figure 6 Kaplan-Meier analysis of Hospitalization of heart failure (PPS)

9.6. Evaluation of main efficacy indicators - subgroup analysis

Table 9.6.1 MACE (CV death and HHF) (≤70 year old)

Item	Index	FAS		PPS	
		Study	Placebo	Study	Placebo
MACE	N(Missing)				
	Endpoint event (%)				
	Censored (%)				
	Median				
	95%CI				
	Q1,Q3				
	Log-rank test				
	P				
Cox Regression	Hazard Ratio				
	95%CI				
	Wald χ^2				
	P				

Table 9.6.2 MACE (CV death and HHF) (>70 year old）

Item	Index	FAS		PPS	
		Study	Placebo	Study	Placebo
MACE	N(Missing)				
	Endpoint event (%)				
	Censored (%)				
	Median				
	95%CI				
	Q1,Q3				
	Log-rank test				
	P				
Cox Regression	Hazard Ratio				
	95%CI				
	Wald χ^2				
	P				

Table 9.6.3 CV Death (≤70 year old)

The following subgroup analysis results, the form of the table is the same as above

Table 9.6.4 CV Death (>70 year old)

Table 9.6.5 HHF (≤70 year old)

Table 9.6.6 HHF (>70 year old)

Table 9.6.7 MACE (CV death and HHF) (Male)

Table 9.6.8 MACE (CV death and HHF) (Female)

Table 9.6.9 CV Death (Male)

Table 9.6.10 CV Death (Female)

Table 9.6.11 HHF (Male)

Table 9.6.12 HHF (Female)

Table 9.6.13 MACE (CV death and HHF) (NYHA calss I and II)

Table 9.6.14 MACE (CV death and HHF) (NYHA calss III and IV)

Table 9.6.15 CV Death (NYHA calss I and II)

Table 9.6.16 CV Death (NYHA calss III and IV)

Table 9.6.17 HHF (NYHA calss I and II)

Table 9.6.18 HHF (NYHA calss III and IV)

Table 9.6.19 MACE (CV death and HHF)（LVEF≤30%）

Table 9.6.20 MACE (CV death and HHF)（LVEF>30%）

Table 9.6.21 CV Death（LVEF≤30%）

Table 9.6.22 CV Death（LVEF>30%）

Table 9.6.23 HHF（LVEF≤30%）

Table 9.6.24 HHF（LVEF>30%）

Table 9.6.25 MACE (CV death and HHF) (Course of heart failure ≤3 yr)

Table 9.6.26 MACE (CV death and HHF) (Course of heart failure >3 yr)

Table 9.6.27 CV Death (Course of heart failure ≤3 yr)

Table 9.6.28 CV Death (Course of heart failure >3 yr)

Table 9.6.29 HHF (Course of heart failure ≤3 yr)

Table 9.6.30 HHF (Course of heart failure >3 yr)

Table 9.6.31 MACE (CV death and HHF) (with ischemic heart disease)

Table 9.6.32 MACE (CV death and HHF) (without ischemic heart disease)

Table 9.6.33 CV Death (with ischemic heart disease)

Table 9.6.34 CV Death (without ischemic heart disease)

Table 9.6.35 HHF (with ischemic heart disease)

Table 9.6.36 HHF (without ischemic heart disease)

Table 9.6.37 MACE (CV death and HHF) (with cardiomyopathy)

Table 9.6.38 MACE (CV death and HHF) (without cardiomyopathy)

Table 9.6.39 CV Death (with cardiomyopathy)

Table 9.6.40 CV Death (without cardiomyopathy)

Table 9.6.41 HHF (with cardiomyopathy)

Table 9.6.42 HHF (without cardiomyopathy)

Table 9.6.43 MACE (CV death and HHF) (with hypertension)

Table 9.6.44 MACE (CV death and HHF) (without hypertension)

Table 9.6.45 CV Death (with hypertension)

Table 9.6.46 CV Death (without hypertension)

Table 9.6.47 HHF (with hypertension)

Table 9.6.48 HHF (without hypertension)

Table 9.6.49 MACE (CV death and HHF) (with diabetes)

Table 9.6.50 MACE (CV death and HHF) (without diabetes)

Table 9.6.51 CV Death (with diabetes)

Table 9.6.52 CV Death (without diabetes)

Table 9.6.53 HHF (with diabetes)

Table 9.6.54 HHF (without diabetes)

Table 9.6.55 MACE (CV death and HHF) (with atrial fibrillation)

Table 9.6.56 MACE (CV death and HHF) (without atrial fibrillation)

Table 9.6.57 CV Death (with atrial fibrillation)

Table 9.6.58 CV Death (without atrial fibrillation)

Table 9.6.59 HHF (with atrial fibrillation)

Table 9.6.60 HHF (without atrial fibrillation)

Table 9.6.61 MACE (CV death and HHF) (with arrhythmias)

Table 9.6.62 MACE (CV death and HHF) (without arrhythmias)

Table 9.6.63 CV Death (with arrhythmias)

Table 9.6.64 CV Death (without arrhythmias)

Table 9.6.65 HHF (with arrhythmias)

Table 9.6.66 HH (without arrhythmias)

Table 9.6.67 MACE (CV death and HHF) (NT-proBNP ≤Median)

Note: The median of NT-proBNP is based on FAS.

Table 9.6.68 MACE (CV death and HHF) (NT-proBNP >Median)

Note: The median of NT-proBNP is based on FAS.

Table 9.6.69 CV Death (NT-proBNP ≤Median)

Note: The median of NT-proBNP is based on FAS.

Table 9.6.70 CV Death (NT-proBNP >Median)

Note: The median of NT-proBNP is based on FAS.

Table 9.6.71 HHF (NT-proBNP ≤Median)

Note: The median of NT-proBNP is based on FAS.

Table 9.6.72 HHF (NT-proBNP >Median)

Note: The median of NT-proBNP is based on FAS.

9.7. Evaluation of secondary efficacy indicators (FAS&PPS)

Table 9.7.1 All-cause mortality

		FAS		PPS	
Item	Index	Study	Placebo	Study	Placebo
All-cause mortality	N(Missing)				
	Endpoint event (%)				
	Censored (%)				
	Median				
	95%CI				
	Q1,Q3				
	Log-rank test				
	P				
Cox Regression	Hazard Ratio				
	95%CI				
	Wald χ^2				
	P				

Table 9.7.2 Composite endpoint (consisting of worsening heart failure leading to withdrawal of treatment, successful resuscitation after cardiac arrest, malignant arrhythmia, and non-fatal stroke)

		FAS		PPS	
Item	Index	Study	Placebo	Study	Placebo
secondary composite outcome (abandoning treatment due to heart failure exacerbation, cardiac arrest resuscitation, malignant arrhythmia, non-fatal stroke)	N(Missing)				
	Endpoint event (%)				
	Censored (%)				
	Median				
	95%CI				
	Q1,Q3				
	Log-rank test				
	P				
Cox Regression	Hazard Ratio				
	95%CI				
	Wald χ^2				
	P				

Table 9.7.3 Worsening heart failure leading to withdrawal of treatment

		FAS		PPS	
Item	Index	Study	Placebo	Study	Placebo
worsening heart failure leading to withdrawal of treatment	N(Missing)				
	Endpoint event (%)				
	Censored (%)				
	Median				

Item	Index	FAS Study	FAS Placebo	PPS Study	PPS Placebo
	95%CI				
	Q1,Q3				
	Log-rank test				
	P				
Cox Regression	Hazard Ratio				
	95%CI				
	Wald χ^2				
	P				

Table 9.7.4 Successful resuscitation after cardiac arrest

Item	Index	FAS Study	FAS Placebo	PPS Study	PPS Placebo
successful resuscitation after cardiac arrest	N(Missing)				
	Endpoint event (%)				
	Censored (%)				
	Median				
	95%CI				
	Q1,Q3				
	Log-rank test				
	P				
Cox Regression	Hazard Ratio				
	95%CI				
	Wald χ^2				
	P				

Table 9.7.5 Malignant arrhythmia

Item	Index	FAS Study	FAS Placebo	PPS Study	PPS Placebo
malignant arrhythmia	N(Missing)				
	Endpoint event (%)				
	Censored (%)				
	Median				
	95%CI				
	Q1,Q3				
	Log-rank test				
	P				
Cox Regression	Hazard Ratio				
	95%CI				
	Wald χ^2				
	P				

Table 9.7.6 Non-fatal stroke

		FAS		PPS	
Item	Index	Study	Placebo	Study	Placebo
non-fatal stroke	N(Missing)				
	Endpoint event (%)				
	Censored (%)				
	Median				
	95%CI				
	Q1,Q3				
	Log-rank test				
	P				
Cox Regression	Hazard Ratio				
	95%CI				
	Wald χ^2				
	P				

Table 9.7.7 Cardiovascular death and hospitalization for heart failure in patients with ischemic heart disease

		FAS		PPS	
Item	Index	Study	Placebo	Study	Placebo
MACE	Yes n(%)				
	No n(%)				
	Total (Missing)				
	Statistics				
	P				
CV death	Yes n(%)				
	No n(%)				
	Total (Missing)				
	Statistics				
	P				
HHF	Yes n(%)				
	No n(%)				
	Total (Missing)				
	Statistics				
	P				

Table 9.7.8 Serum NT-proBNP level baseline and change from baseline (1month)

		FAS		PPS	
Item	Index	Study	Placebo	Study	Placebo
Baseline NT-proBNP (pg/ml)	N(Missing)				
	Mean(Sd)				

Item	Index	FAS Study	FAS Placebo	PPS Study	PPS Placebo
	Median				
	Q1,Q3				
	Min,Max				
Baseline comparsion	Statistics				
	P				
1-month	N(Missing)				
	Mean(Sd)				
	Median				
	Q1,Q3				
	Min,Max				
1-month comparsion	Statistics				
	P				
1-month and baseline difference	N(Missing)				
	Mean(Sd)				
	Median				
	Q1,Q3				
	Min,Max				
1-month intragroup difference	Statistics				
	P				
Inter-group difference	Statistics				
	P				

Table 9.7.8 Serum NT-proBNP level baseline and change from baseline (1month)

Format same as above

Table 9.7.9 Seurm NT-proBNP decrease rate from baseline (1 month)

Item	Index	FAS Study	FAS Placebo	PPS Study	PPS Placebo
Decrease rate of marker (%）	N(Missing)				
	Mean(Sd)				
	Median				
	Q1,Q3				
	Min,Max				
Comparison	Statistics				
	P				
Decrease rate ≥30%	No n(%)				
	Yes n(%)				
	Total (Missing)				

Item	Index	FAS Study	FAS Placebo	PPS Study	PPS Placebo
Comparison	Statistics				
	P				

Note: Decrease rate = (baseline result - 1 month result) / baseline result * 100%

Table 9.7.10 Seurm NT-proBNP decrease rate from baseline (3 months)

Format same as above

9.8. Safety Evaluation (SS)

Table 9.8.1 Summary of Adverse Events

Item	Index	Study	Placebo	Total	Statistics	P
Adverse Events	No n(%)					
	Yes n(%)					
	Total (Missing)					
Major Adverse Events	No n(%)					
	Yes n(%)					
	Total (Missing)					

Table 9.8.2 Summary of adverse events

Item	Study Group Incident Patients	Study Group N (%)	Placebo Group Incident Patients	Placebo Group N (%)	P
Adverse event					
Adverse events related to study drug					
Severity grade 3 4 5 adverse events					
Severe adverse events					
Severe adverse events related to study drug					
Adverse events leading to withdrawal					
Study drug-related adverse events leading to withdrawal					

Note: Related to the study drug is defined as "definite," "possible," and "uncertain".

Table 9.8.3 Coding of adverse event (SOC/PT)

Code	Study Group Incident	Study Group Patients	Study Group N (%)	Placebo Group Incident	Placebo Group Patients	Placebo Group N (%)	P
Adverse event							

Table 9.8.4 Coding of adverse drug reaction (SOC/PT)

	Study Group			Placebo Group			
Item	Incident	Patients	N (%)	Incident	Patients	N (%)	P
Adverse drug reaction							

Table 9.8.5 Comparison of vital signs (12 months ± 7 days) - systolic blood pressure (mmHg)

		SS	
Item	Index	Study Group	Placebo Group
Baseline systolic blood pressure	N(Missing)		
	Mean(Sd)		
	Median		
	Q1,Q3		
	Min,Max		
	Statistics		
	P		
12-month systolic blood pressure	N(Missing)		
	Mean(SD)		
	Median		
	Q1,Q3		
	Min,Max		
Intergroup comparison in 12-month	Statistics		
	P		
Intragroup difference between baseline and 12-month	N(Missing)		
	Mean(SD)		
	Median		
	Q1,Q3		
	Min,Max		
Intragroup comparison	Statistics		
	P		
Intragroup difference comparison	Statistics		
	P		
Rate of change	N(Missing)		
	Mean(SD)		
	Median		
	Q1,Q3		
	Min,Max		
Intragroup comparison of the rate of change	Statistics		
	P		
Intergroupomparison of the rate of change	Statistics		
	P		

Table 9.8.6 Comparison of vital signs (12 months ± 7 days) - diastolic blood pressure (mmHg)

Format same as above

Table 9.8.7 Comparison of vital signs (12 months ± 7 days) - heart rate (bpm)

Format same as above

Table 9.8.8 Comparison of vital signs (12 months ± 7 days) - pluse (bpm)

Format same as above

Table 9.8.9 Comparison of vital signs (12 months ± 7 days) – Body temperature (℃)

Format same as above

Table 9.8.10 Crosstabulation of Complete blood count - white blood cell count (12 months ± 7 days)

		White blood cell count					
Group	Before	Normal	Insignificant abnormal	Significant abnormal	Untested	Missing	Total
Study							
	Normal						
	Insignificant abnormal						
	Significant abnormal						
	Untested						
	Missing						
	Total						
Placebo							
	Normal						
	Insignificant abnormal						
	Significant abnormal						
	Untested						
	Missing						
	Total						

Table 9.8.11 Crosstabulation of Complete blood count - hemoglobin (12 months ± 7 days)

Format same as above

Table 9.8.12 Crosstabulation of Complete blood count - red blood cell count (12 months ± 7 days)

Table 9.8.13 Crosstabulation of Complete blood count - platelets (12 months ± 7 days)

Table 9.8.14 Summary of Complete blood count - 12 months ± 7 days (SS)

		Normal before IPs		Abnormal before IPs	
Index	Group	Normal after IPs	Abnormal after IPs	Normal after IPs	Abnormal after IPs
white blood cell count	Study				
	Placebo				
hemoglobin	Study				
	Placebo				
red blood cell count	Study				
	Placebo				
platelets	Study				
	Placebo				

Table 9.8.15 Crosstabulation of biochemistry - Alanine aminotransferase (ALT) (12 months ± 7 days)

Table 9.8.16 Crosstabulation of biochemistry – Creatinine (Cr) (12 months ± 7 days)

Table 9.8.17 Crosstabulation of biochemistry – Blood Uear Nitrogrn (BUN) (12 months ± 7 days)

Table 9.8.18 Crosstabulation of biochemistry – Total cholesterol (TC) (12 months ± 7 days)

Table 9.8.19 Crosstabulation of biochemistry – Triglyciride (TG) (12 months ± 7 days)

Table 9.8.20 Crosstabulation of biochemistry – Potassium (K) (12 months ± 7 days)

Table 9.8.21 Crosstabulation of biochemistry – Sodium (Na) (12 months ± 7 days)

Table 9.8.22 Crosstabulation of biochemistry – Chlorine (Cl) (12 months ± 7 days)

Table 9.8.23 Crosstabulation of biochemistry – Fasting blood glucose (GLU) (12 months ± 7 days)

Table 9.8.24 Summary of Biochemistry - 12 months ± 7 days (SS)

		Normal before IPs		Abnormal before IPs	
Index	Group	Normal after IPs	Abnormal after IPs	Normal after IPs	Abnormal after IPs
ALT	Study				
	Placebo				
Cr	Study				
	Placebo				
BUN	Study				
	Placebo				
TC	Study				
	Placebo				
TG	Study				
	Placebo				
K	Study				
	Placebo				
Na	Study				
	Placebo				
Cl	Study				
	Placebo				
GLU	Study				
	Placebo				

Table 9.8.25 Crosstabulation of Urine routine – Urine protine (12 months ± 7 days)

Table 9.8.26 Crosstabulation of Urine routine – Urine white blood cell (12 months ± 7 days)

Table 9.8.27 Crosstabulation of Urine routine – Urine red blood cell (12 months ± 7 days)

Table 9.8.28 Summary of Urine routine - 12 months ± 7 days

		Normal before IPs		Abnormal before IPs	
Index	Group	Normal after IPs	Abnormal after IPs	Normal after IPs	Abnormal after IPs
Urine protine	Study				
	Placebo				
Urine white blood cell	Study				
	Placebo				
Urine red blood cell	Study				
	Placebo				

Table 9.8.29 Crosstabulation of Physical examination – General condition (12 months ± 7 days)

Table 9.8.30 Crosstabulation of Physical examination – Nervous system (12 months ± 7 days)

Table 9.8.31 Crosstabulation of Physical examination – Skin, superficial lymph nodes (12 months ± 7 days)

Table 9.8.32 Crosstabulation of Physical examination – head and neck (12 months ± 7 days)

Table 9.8.33 Crosstabulation of Physical examination – Cardiovascular (12 months ± 7 days)

Table 9.8.34 Crosstabulation of Physical examination – Respiratory (12 months ± 7 days)

Table 9.8.35 Crosstabulation of Physical examination – Abdominal (12 months ± 7 days)

Table 9.8.36 Crosstabulation of Physical examination – Genitourinary (12 months ± 7 days)

Table 9.8.37 Crosstabulation of Physical examination – Spine and Limbs (12 months ± 7 days)

Table 9.8.38 Crosstabulation of Physical examination – Other (12 months ± 7 days)

Table 9.8.39 Summary of Urine routine - 12 months ± 7 days

		Normal before IPs		Abnormal before IPs	
Index	Group	Normal after IPs	Abnormal after IPs	Normal after IPs	Abnormal after IPs
General condition	Study				
	Placebo				
Nervous system	Study				
	Placebo				
Skin, superficial lymph nodes	Study				
	Placebo				
Head and neck	Study				
	Placebo				

Index	Group	Normal before IPs		Abnormal before IPs	
		Normal after IPs	Abnormal after IPs	Normal after IPs	Abnormal after IPs
Cardiovascular	Study				
	Placebo				
Respiratory	Study				
	Placebo				
Abdominal	Study				
	Placebo				
Genitourinary	Study				
	Placebo				
Spine and Limbs	Study				
	Placebo				
Other	Study				
	Placebo				

Table 9.8.40 Crosstabulation of electrocardiogram (12 months ± 7 days)

Table 9.8.41 Summary of electrocardiogram - 12 months ± 7 days

Index	Group	Normal before IPs		Abnormal before IPs	
		Normal after IPs	Abnormal after IPs	Normal after IPs	Abnormal after IPs
Electrocardiogram with clinical significance	Study				
	Placebo				

9.9. List of appendixes

Appendix 1 – Dropout/ Elimination List (all randomized populations)

Center	Random No.	Group	Completion	Dropout Date	Dropout Reason	Detail	Condition	Dropout classification	Specific description	FAS	PPS	SS

Appendix 2 – Demographics (FAS)

Center	Random No.	Group	Date of birth	Age (yrs)	Sex	Marriage	Ethnic	Other	Height (cm)	Weight (kg)	BMI (kg/m^2)	Nationality	Other

Appendix 3 – Concomitant medication

Center	Random No.	Group	W/O Concomitant medication	Drug name (generic name)	Reason for medication	AE No.	Medical history coding	Dosage	Start date	End date	Continued at last follow-up	ATC1 term	ATC2 term

Appendix 4- Medication Compliance List (Dispensation and Use of Study Drugs) (FAS)

Center	Random No.	Group	Drug dispense date	Last visit date	Days of medication	Dosage to be taken (tablets)	Actual dosage be taken (tablets)	Compliance (%)	FAS	PPS

Appendix 5 - List of Primary Effecacy Endpoint

Center	Random No.	Group	Randomized date	MACE Date	Censored MACE	Time (days)	CV death Date	Censored CV death	Time (days)	HHF Date	Censored HHF	Time (days)	FAS	PPS	SS

Appendix 6 - List of Secondary Efficacy Endpoint

Center	Random No.	Group	Randomized date	All-cause mortality Date	Censored 1	Time (days)	Composite endpoint (worsening heart failure leading to withdrawal of treatment, successful resuscitation after cardiac arrest, malignant arrhythmia, and non-fatal stroke) Date	Censored 2	Time (days)	Worsening heart failure leading to withdrawal of treatment Date	Censored 3	Time (days)	FAS	PPS

Appendix 6-1 - List of Secondary Efficacy Endpoint - Continuation

Center	Random No.	Group	Randomized date	successful resuscitation after cardiac arrest	Censored 4	Time (days)	Malignant arrhythmia	Censored 5	Time (days)	non-fatal stroke	Censored 6	Time (days)	FAS	PPS

Appendix 7- Serum NT-proBNP

Center	Random No.	Group	Baseline NT-proBNP(pg/ml)	1 month NT-proBNP(pg/ml)	Difference (Basline-1month)	Decrease rate (%)	Decrease rate ≥30%	3 months NT-proBNP(pg/ml)	Difference (Baseline-3months)	Decrease rate (%)	Decrease rate ≥30%	FAS	PPS

Appendix 8 - List of Adverse Events

Center	Random No.	Group	Y/N AE	AE Date	AE Grade	Y/N SAE	Death	Life-threadening	Hospitalization or prolong stay	Causes permanent or significant disability	Congenital malformation	Medical event	Actions Taken on IPs	Whether to treat	Whether to withdraw from the trial due to adverse events	outcome	Relationship to Study Drug	Detailed description of adverse events	Termination/Vestination Date	version number	SOC	PT

Appendix 9 - List of Severe Adverse Events

Center	Random No.	Group	Y/N AE	AE Date	AE Grade	Y/N SAE	Death	Life-threadening	Hospitalization or prolong stay	Causes permanent or significant disability	Congenital malformation	Medical event	Actions Taken on IPs	Whether to treat	Whether to withdraw from the trial due to adverse events	outcome	Relationship to Study Drug	Detailed description of adverse events	Termination/Vestination Date	version number	SOC	PT

Appendix 10 – List of abnormoal complete blood count from normal baseline (SS)

Center	Random No.	Index	Group	Before IPs	Clinical significance	After IPs	Clinical significance

Appendix 11 - List of abnormoal complete blood count from abnormal baseline (SS)

Format same as above

Appendix 12 - List of abnormoal biochemistry from normal baseline (SS)

Format same as above

Appendix 13 - List of abnormoal biochemistry from abnormoal baseline (SS)

Format same as above

Appendix 14 - List of abnormoal urine routine from normal baseline (SS)

Format same as above

Appendix 15 - List of abnormoal urine routine from abnormal baseline (SS)

Format same as above

Appendix 16 - List of abnormoal physical examination from normal baseline (SS)

Format same as above

Appendix 17 - List of abnormoal physical examination from abnormal baseline (SS)

Format same as above

Appendix 18 - List of abnormoal electrocardiogram from normal baseline (SS)

Format same as above

Appendix 19 - List of abnormoal electrocardiogram from abnormal baseline (SS)

Format same as above

Appendix 20- Trial Completion List (All Randomized Populations)

Center	Random No.	Group	Completion	Dropout Date	Dropout Reason	Detailed	Y/N Unblinding	Unblinding Date	Elimination Date
								.	
								.	
								.	

Appendix 21- List of Unblinding List (All Randomized Populations)

Center	Random No.	Group	Completion	Randomized Date	Y/N Unblinding	Unblinding Date	Unblinding Date Reason	Death	Date of death

芪苈强心胶囊对慢性心衰复合终点事件的评估研究

统计分析计划 V1.0

课题承担单位：南京医科大学第一附属医院

药品生产单位：石家庄以岭药业股份有限公司

统计分析单位：北京大学临床研究所

版本日期：2022 年 11 月 21 日

作者：____________________　　　日期：____________________

签署人：____________________　　　日期：____________________

目录

1. 缩略语 8

2. 试验题目 9

3. 试验目的 9

4. 试验总体设计与安排 9

4.1. 随机分组阶段（第 0 天~第 24 个月） 10
4.2. 病例数量、分组、中心 10
4.3. 盲法与随机化 11
4.3.1. 受试者随机分配方法 11
4.3.2. 紧急揭盲 11
4.3.3. 揭盲规定 12
4.3.4. 筛选编号 12
4.4. 治疗方案 12
4.4.1. 研究流程 12
4.4.2. 基础治疗 13
4.4.3. 合并用药 13
4.4.4. 疗程：12 个月-36 个月 13
4.5. 依从性评价 13
4.6. 药物不良反应 13

5. 研究人群 14

5.1. 入选标准 14
5.2. 排除标准 14
5.3. 中止研究药物治疗 15
5.4. 退出标准 15
5.5. 全面中止试验标准 16

6. 临床观察终点和指标 16

6.1. 临床观察指标 16
6.1.1. 主要有效终点 16
6.1.2. 次要有效终点 16
6.2. 安全性指标包括： 16
6.2.1. 不良事件定义 17
6.2.2. 不良事件强度判定标准 17
6.2.3. 不良事件与研究药物关系的判断标准 17
6.2.4. 严重不良事件 18
6.2.5. 不良事件的随访与记录 19
6.2.6. 实验室结果异常 19
6.3. 终点事件的含义及 CEC 判定程序 19
6.3.1. 本研究终点事件的含义 19

6.3.2. 终点事件发生时的评估与程序 *21*

7. 数据管理 21

7.1. 数据库设计 22
7.2. 数据录入 22
7.3. 数据质疑管理 22
7.4. 医学编码 22
7.5. 数据审核 22
7.6. 数据库锁定 22

8. 统计分析 22

8.1. 分析人群 23
8.2. 统计分析方法 23
8.2.1. 入组及完成情况 *23*
8.2.2. 依从性分析 *24*
8.2.3. 疗效评价 *24*
8.2.4. 安全性评价 *24*
8.3. 期中分析 25

9. 统计分析结果 26

9.1. 病例分布（所有随机化人群） 26
表 9.1.1 各中心病例分布 *26*
表 9.1.2 两组脱落剔除情况比较 *26*
表 9.1.3 各中心的人群划分情况描述 *26*
表 9.1.4 入组病例及安全性有效性分析数据集 *27*
9.2. 人口学和基线信息(FAS) 28
表 9.2.1 人口学资料 *28*
表 9.2.2 个人史 *28*
表 9.2.3 疾病情况和既往病史 *29*
表 9.2.4 重要既往病史分析 *30*
表 9.2.5 既往病史分析（SOC/PT） *30*
表 9.2.6 重要病史（PT） *31*
表 9.2.7 NYHA 心功能分级 *31*
表 9.2.8 心脏彩色超声检查 *31*
表 9.2.9 血清 NT-proBNP 检测 *32*
表 9.2.10 生命体征 *32*
9.3. 中位随访时间 33
表 9.3.1 随访时间描述 *33*
表 9.3.2 中位随访时间 *33*
9.4. 用药情况(SS) 34
表 9.4.1 用药依从性 *34*
表 9.4.2 既往用药/合并用药 *34*
表 9.4.3 合并用药编码（ATC1/ATC2） *34*

9.5. 主要疗效指标评价（FAS&PPS）......35
表 9.6.1 发生复合终点事件（心血管死亡和心衰恶化再住院）的时间......35
图 1 发生复合终点事件 KM 曲线图（FAS 数据集）......35
图 2 发生复合终点事件 KM 曲线图（PPS 数据集）......35
表 9.6.2 发生心血管死亡的时间......36
图 3 心血管死亡 KM 曲线图（FAS 数据集）......36
图 4 心血管死亡 KM 曲线图（PPS 数据集）......36
表 9.6.3 发生心衰恶化再住院的时间......37
图 5 心衰恶化再住院 KM 曲线图（FAS 数据集）......37
图 6 心衰恶化再住院 KM 曲线图（PPS 数据集）......37
9.6. 主要疗效指标评价-亚组分析......38
表 9.6.1 复合终点事件（心血管死亡和心衰恶化再住院）（≤70 岁）......38
表 9.6.2 复合终点事件（心血管死亡和心衰恶化再住院）（＞70 岁）......38
表 9.6.3 发生心血管死亡的时间（≤70 岁）......38
表 9.6.4 发生心血管死亡的时间（>70 岁）......38
表 9.6.5 发生心衰恶化再住院的时间（≤70 岁）......39
表 9.6.6 发生心衰恶化再住院的时间（>70 岁）......39
表 9.6.7 复合终点事件（心血管死亡和心衰恶化再住院）（男性）......39
表 9.6.8 复合终点事件（心血管死亡和心衰恶化再住院）（女性）......39
表 9.6.9 发生心血管死亡的时间（男性）......39
表 9.6.10 发生心血管死亡的时间（女性）......39
表 9.6.11 发生心衰恶化再住院的时间（男性）......39
表 9.6.12 发生心衰恶化再住院的时间（女性）......39
表 9.6.13 复合终点事件（心血管死亡和心衰恶化再住院）（NYHA 分级 I 级或 II 级）......39
表 9.6.14 复合终点事件（心血管死亡和心衰恶化再住院）（NYHA 分级 III 级或IV级）......39
表 9.6.15 发生心血管死亡的时间（NYHA 分级 I 级或 II 级）......39
表 9.6.16 发生心血管死亡的时间（NYHA 分级 III 级或IV级）......39
表 9.6.17 发生心衰恶化再住院的时间（NYHA 分级 I 级或 II 级）......39
表 9.6.18 发生心衰恶化再住院的时间（NYHA 分级 III 级或IV级）......39
表 9.6.19 复合终点事件（心血管死亡和心衰恶化再住院）（LVEF≤30%）......40
表 9.6.20 复合终点事件（心血管死亡和心衰恶化再住院）（LVEF>30%）......40
表 9.6.21 发生心血管死亡的时间（LVEF≤30%）......40
表 9.6.22 发生心血管死亡的时间（LVEF>30%）......40
表 9.6.23 发生心衰恶化再住院的时间（LVEF≤30%）......40
表 9.6.24 发生心衰恶化再住院的时间（LVEF>30%）......40
表 9.6.25 复合终点事件（心血管死亡和心衰恶化再住院）（心衰病程≤3 年）......40
表 9.6.26 复合终点事件（心血管死亡和心衰恶化再住院）（心衰病程>3 年）......40
表 9.6.27 发生心血管死亡的时间（心衰病程≤3 年）......40
表 9.6.28 发生心血管死亡的时间（心衰病程>3 年）......40
表 9.6.29 发生心衰恶化再住院的时间（心衰病程≤3 年）......40
表 9.6.30 发生心衰恶化再住院的时间（心衰病程>3 年）......40
表 9.6.31 复合终点事件（心血管死亡和心衰恶化再住院）（有冠心病）......40

表 9.6.32 复合终点事件（心血管死亡和心衰恶化再住院）（无冠心病） 40
表 9.6.33 发生心血管死亡的时间（有冠心病） 41
表 9.6.34 发生心血管死亡的时间（无冠心病） 41
表 9.6.35 发生心衰恶化再住院的时间（有冠心病） 41
表 9.6.36 发生心衰恶化再住院的时间（无冠心病） 41
表 9.6.37 复合终点事件（心血管死亡和心衰恶化再住院）（有心肌病） 41
表 9.6.38 复合终点事件（心血管死亡和心衰恶化再住院）（无心肌病） 41
表 9.6.39 发生心血管死亡的时间（有心肌病） 41
表 9.6.40 发生心血管死亡的时间（无心肌病） 41
表 9.6.41 发生心衰恶化再住院的时间（有心肌病） 41
表 9.6.42 发生心衰恶化再住院的时间（无心肌病） 41
表 9.6.43 复合终点事件（心血管死亡和心衰恶化再住院）（有高血压病） 41
表 9.6.44 复合终点事件（心血管死亡和心衰恶化再住院）（无高血压病） 41
表 9.6.45 发生心血管死亡的时间（有高血压病） 41
表 9.6.46 发生心血管死亡的时间（无高血压病） 41
表 9.6.47 发生心衰恶化再住院的时间（有高血压病） 42
表 9.6.48 发生心衰恶化再住院的时间（无高血压病） 42
表 9.6.49 复合终点事件（心血管死亡和心衰恶化再住院）（有糖尿病） 42
表 9.6.50 复合终点事件（心血管死亡和心衰恶化再住院（无糖尿病） 42
表 9.6.51 发生心血管死亡的时间（有糖尿病） 42
表 9.6.52 发生心血管死亡的时间（无糖尿病） 42
表 9.6.53 发生心衰恶化再住院的时间（有糖尿病） 42
表 9.6.54 发生心衰恶化再住院的时间（无糖尿病） 42
表 9.6.55 复合终点事件（心血管死亡和心衰恶化再住院）（有房颤） 42
表 9.6.56 复合终点事件（心血管死亡和心衰恶化再住院）（无房颤） 42
表 9.6.57 发生心血管死亡的时间（有房颤） 42
表 9.6.58 发生心血管死亡的时间（无房颤） 42
表 9.6.59 发生心衰恶化再住院的时间（有房颤） 42
表 9.6.60 发生心衰恶化再住院的时间（无房颤） 42
表 9.6.61 复合终点事件（心血管死亡和心衰恶化再住院）（有心率失常） 43
表 9.6.62 复合终点事件（心血管死亡和心衰恶化再住院）（无心率失常） 43
表 9.6.63 发生心血管死亡的时间（有心率失常） 43
表 9.6.64 发生心血管死亡的时间（无心率失常） 43
表 9.6.65 发生心衰恶化再住院的时间（有心率失常） 43
表 9.6.66 发生心衰恶化再住院的时间（无心率失常） 43
表 9.6.67 复合终点事件（心血管死亡和心衰恶化再住院）（BNP≤中位数） 43
表 9.6.68 复合终点事件（心血管死亡和心衰恶化再住院）（BNP>中位数） 43
表 9.6.69 发生心血管死亡的时间（BNP≤中位数） 43
表 9.6.70 发生心血管死亡的时间（BNP>中位数） 43
表 9.6.71 发生心衰恶化再住院的时间（BNP≤中位数） 43
表 9.6.72 发生心衰恶化再住院的时间（BNP>中位数） 43
9.7. 次要疗效指标评价（FAS&PPS） 44

表 9.7.1 全因死亡率 44
表 9.7.2 复合终点事件（心衰恶化放弃治疗、心脏骤停后复苏成功、恶性心律失常、非致死性卒中）率 44
表 9.7.3 心衰恶化放弃治疗率 44
表 9.7.4 心脏骤停后复苏成功率 45
表 9.7.5 恶性心律失常率 45
表 9.7.6 非致死性卒中率 46
表 9.7.7 冠心病心衰患者的心血管死亡和心衰恶化再住院 46
表 9.7.8 血清 NT-proBNP 较基线变化情况（1 个月） 47
表 9.7.9 血清 NT-proBNP 较基线变化情况（3 个月） 47
表 9.7.10 血清 NT-proBNP 较基线下降率（1 个月） 47
表 9.7.11 血清 NT-proBNP 较基线下降率（3 个月） 48
9.8. 安全性评价（SS） 48
表 9.8.1 不良事件汇总比较 48
表 9.8.2 研究期间不良事件汇总比较 48
表 9.8.3 不良事件编码情况（SOC/PT） 49
表 9.8.4 不良反应编码情况（SOC/PT） 49
表 9.8.5 生命体征前后比较(12 个月±7 天)-收缩压（mmHg） 49
表 9.8.6 生命体征前后比较(12 个月±7 天)-舒张压（mmHg） 50
表 9.8.7 生命体征前后比较(12 个月±7 天)-心率（次/分） 50
表 9.8.8 生命体征前后比较(12 个月±7 天)-脉搏（次/分） 50
表 9.8.9 生命体征前后比较(12 个月±7 天)-体温（℃） 50
表 9.8.10 血常规-12 个月±7 天白细胞总数前后交叉表 50
表 9.8.11 血常规-12 个月±7 天血红蛋白前后交叉表 51
表 9.8.12 血常规-12 个月±7 天红细胞总数前后交叉表 51
表 9.8.13 血常规-12 个月±7 天血小板前后交叉表 51
表 9.8.14 血常规-12 个月±7 天汇总表（SS） 51
表 9.8.15 血生化-12 个月±7 天谷丙转氨酶前后交叉表 51
表 9.8.16 血生化-12 个月±7 天肌酐前后交叉表 51
表 9.8.17 血生化-12 个月±7 天尿素氮前后交叉表 51
表 9.8.18 血生化-12 个月±7 天总胆固醇前后交叉表 51
表 9.8.19 血生化-12 个月±7 天甘油三酯前后交叉表 51
表 9.8.20 血生化-12 个月±7 天钾前后交叉表 51
表 9.8.21 血生化-12 个月±7 天钠前后交叉表 52
表 9.8.22 血生化-12 个月±7 天氯前后交叉表 52
表 9.8.23 血生化-12 个月±7 天空腹血糖前后交叉表 52
表 9.8.24 血生化-12 个月±7 天汇总表 52
表 9.8.25 尿常规-12 个月±7 天尿蛋白前后交叉表 52
表 9.8.26 尿常规-12 个月±7 天尿白细胞前后交叉表 52
表 9.8.27 尿常规-12 个月±7 天尿红细胞前后交叉表 52
表 9.8.28 尿常规-12 个月±7 天汇总表 53
表 9.8.29 体格检查-12 个月±7 天一般情况前后交叉表 53
表 9.8.30 体格检查-12 个月±7 天神经系统前后交叉表 53

表 9.8.31 体格检查-12 个月±7 天皮肤、浅表淋巴结前后交叉表 53
表 9.8.32 体格检查-12 个月±7 天头颈部前后交叉表 53
表 9.8.33 体格检查-12 个月±7 天心脏前后交叉表 53
表 9.8.34 体格检查-12 个月±7 天肺部前后交叉表 53
表 9.8.35 体格检查-12 个月±7 天腹部前后交叉表 53
表 9.8.36 体格检查-12 个月±7 天泌尿生殖系统前后交叉表 53
表 9.8.37 体格检查-12 个月±7 天脊柱四肢前后交叉表 53
表 9.8.38 体格检查-12 个月±7 天其他前后交叉表 53
表 9.8.39 体格检查-12 个月±7 天汇总表 54
表 9.8.40 心电图-12 个月±7 天前后交叉表 54
表 9.8.41 心电图-12 个月±7 天汇总表 54
9.9. 清单 55

1. 缩略语

ACEI	血管紧张素转换酶抑制剂
ADR	可疑药物不良反应
AEs	不良事件
AHA	美国心脏学会
AMI	急性心肌梗死
ARB	血管紧张素受体拮抗剂
ARNI	血管紧张素受体脑啡肽酶抑制剂
BNP	B 型利钠肽
CEC	事件判定委员会
CGRP	降钙素基因相关肽
CHF	慢性心力衰竭
CRA	临床研究监查员
CRC	临床研究协调员
CRF	病历报告表
CV	心血管
DSMB	数据与安全监察委员会
DVP	数据核查计划
ECG	心电图
EF	射血分数
EOS	终末访视
ESC	欧洲心脏病学会
ET	内皮素
FAS	全分析集
HF	心力衰竭
LVOT	左心室流出量
MedDRA	ICH 国际医学用语词典

MI	心肌梗死
NO	一氧化氮
NT-proBNP	氨基末端 B 型利钠肽前体
NYHA	纽约心脏病学会
PPS	符合方案集
RAAS	肾素-血管紧张素-醛固酮系统
SAE	严重不良事件
SNS	交感神经系统
SS	安全性分析集
TVI	时间流速积分
UNS	计划外访视

2. 试验题目

芪苈强心胶囊对慢性心衰复合终点事件的评估研究

3. 试验目的

采用循证医学研究方法，以心血管死亡率和心衰恶化再住院发生率为主要研究终点，进一步阐明芪苈强心胶囊长期用药的临床疗效及安全性，明确疗效特点及适宜人群，为优化临床合理用药方案提供高质量临床证据。

4. 试验总体设计与安排

本研究是一项在慢性心衰患者中进行的随机、双盲、安慰剂对照、平行分组的多中心临床研究。

本研究将为事件驱动型，全部随机入组的患者将保留在研究之中（无论是否服用研究药物），直至主要终点事件的发生数目达到预计（620 例），或者当满足事先定义的提前终止的疗效或安全性标准时，研究提前终止。

计划在发生 1/2、2/3 主要终点事件后进行两次期中疗效分析，以评估是否已得出无效或有效的结论而提前终止该研究。

计划整个研究将持续大约 36 个月，招募期预计 24 个月，最后一例患者入组研究后的随访期为 12 个月。预计平均随访时间约为 24 个月。

在医院开始筛选患者，临床症状稳定，入选之前已接受至少 2 周标准化方案治疗并治疗其他伴随疾病。根据当地 HF 治疗指南规定用药，药物种类、剂量固定，除非禁忌或不耐受，且此期间未静脉用药，未服用与芪苈强心胶囊成分相似中药、中成药的患者直接进入随机分组阶段。

若达不到上述要求，则可先行标准化治疗达到上述标准后再进入随机分组阶段。

4.1. 随机分组阶段（第 0 天~第 24 个月）

*请注意：*接受 2 周以上的标准化治疗方案且未使用中药治疗符合入选标准的受试者进入随机分组阶段。此期间的每一位患者使用药物种类、剂量需要固定。若属医疗需要调整用药，需记录在病例报告表中。

患者将按照 1:1 的比例随机化到试验组或对照组。患者将在当前慢性心衰标准化治疗的基础上使用研究药物。

试验组：慢性心衰标准化治疗+芪苈强心胶囊（4 粒/次，3 次/日，口服）；

对照组：慢性心衰标准化治疗+芪苈强心胶囊安慰剂（4 粒/次，3 次/日，口服）。

治疗期间应避免使用其他中药或中成药（与芪苈强心功能组成相似的中药）

患者应于随机分组后第 1 个月、第 3 个月、第 6 个月，第 9 个月，第 12 个月，此后每隔 3 个月来医院访视，进行有效性和安全性评估，直到研究全部结束。随机分组阶段共 24 个月。

4.2. 病例数量、分组、中心

参考 PARADIGM-HF 研究，中位随访 27 个月 LCZ696 组患者的心血管死亡或心衰住院率为 21.8%，而依那普利组为 26.5%。所以我们估算基础治疗+安慰剂组随访 36 个月内，所有患者的心血管死亡和心衰住院事件发生率为 25%，基础治疗+芪苈强心组发生率为 20%。

试验组与对照组的随机分配比例为 1:1，考虑到期中分析对 I 类错误的消耗，α 调整为单侧 0.02314。样本量为复合终点事件的发生例数。预计需要观察到 620 例复合终点事件，才能提供 80%的把握度（β=0.2），经过 log-rank 检验得到试验组可以降低 20%风险的结论。

假设随访期 36 个月内对照组复合终点事件的发生率为 25%，整个试验持续大约 36 个月，招募期预计 24 个月，则预计需要入组 3080 例（每组 1540 例）受试者才可获得 620 个终点事件。

因此本研究计划纳入 3080 例患者，患者将以患者将以 1:1 的比例分配至试验组与对照组，并计划在约 100 个中心进行。

4.3. 盲法与随机化

4.3.1. 受试者随机分配方法

由北京大学临床研究所统计专业人员，在计算机上用 SAS9.4 统计软件包，按试验组与对照组 1:1 的比例用区组随机化方法生成随机编号。根据此随机编号由与本试验无关的人员对研究药物（芪苈强心胶囊或安慰剂）进行包装编码。

本研究采用随机化与试验药物管理系统（RTSM）, 统计专业人员将向RTSM提供随机编号列表。然后，由RTSM给患者分配随机编号。

在完成基线评估后，在基线访视通过 RTSM 分配随机编号。此后，按照访视计划通过 RTSM 获取药物编号，每次分配的药物编号均不相同，但药物是同一种。在患者随机分组前，研究者必须先登录 RTSM，并且提供一些信息（例如受试者出生日期、性别等）。

4.3.2. 紧急揭盲

如果发生不良事件，只有在必须了解研究药物的使用才能治疗患者的特殊情况下，才能进行紧急揭盲。一旦决定揭盲，研究者必须记录日期、时间和破盲的原因。

研究者需要登录 RTSM 填写破盲申请，由主要研究者审核后再由破盲员破盲。一旦破盲，该病例将中止研究，作为退出处理。

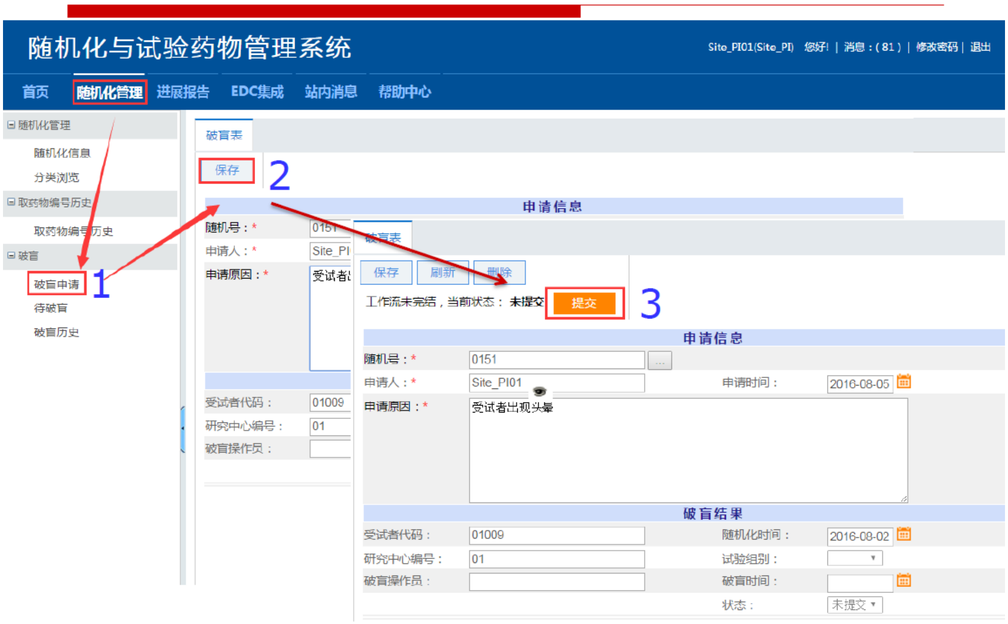

4.3.3. 揭盲规定

本研究采用二次揭盲法。在经盲态核查后，数据锁定，由主要研究者、医学统计专家、数据管理员、申办单位代表进行第一次揭盲，将各随机号所对应的组别以 A，B 为代号标出，以便对全部数据进行统计分析。当统计分析结束，统计报告完成时，再进行第二次揭盲，宣布 A，B 两组的确切组别。

4.3.4. 筛选编号

各医院按收治患者的先后顺序编排筛选号，筛选号由中心编号+3 位整数表示，如，01 中心筛选号 01001，01002，……。

4.4. 治疗方案

4.4.1. 研究流程

入组期（第-14 天~第 0 天）：

患者如果符合本方案中标准治疗的规定，可在此期间完成所有检查，符合入选、排除标准的患者进入随机分组阶段。

随机治疗期（第 0 天~12 个月（最长 36 个月））：（以发药时间记为 0 天）

试验组：慢性心衰标准化治疗+芪苈强心胶囊（4 粒/次 3 次/日 口服）

对照组：慢性心衰标准化治疗+芪苈强心胶囊模拟剂（4 粒/次 3 次/日 口服）

研究药物推荐于每日三餐后约 30 分钟时服用。访视日早晨勿服用药物。如果患者某日未服药，次日服药剂量不得超过日剂量，本研究不允许进行剂量调整。如果患者出现

无法耐受的不良事件，并且根据研究者考虑与研究药物相关，则患者应当终止研究药物治疗

4.4.2. 基础治疗

参照当地 HF 治疗指南或中华医学会心血管病学分会发布的“中国心力衰竭诊断和治疗指南 2018”规定如下：

1) 入选前至少 2 周以上未静脉使用利尿剂、强心剂及血管扩张剂。
2) 进入随机分组阶段至少 2 周前，患者应当接受慢性心衰标准化治疗，所有药物已经调整至固定剂量，标准化药物治疗包括：血管紧张素转换酶抑制剂（ACEI）或血管紧张素受体拮抗剂（ARB）或血管紧张素受体脑啡肽酶抑制剂(ARNI)、β 受体阻滞剂及醛固酮受体拮抗剂（除非禁忌或不耐受，应达到最佳治疗剂量）;

进入治疗期后每一位患者使用药物种类、剂量需延续入选前标准化治疗方案。整个治疗期间原则上不能再调整；若属医疗需要调整用药，需记录在病例报告表中，增加或减少药物剂量、种类的患者需记录复合终点事件和不良事件。

4.4.3. 合并用药

1) 接受较好地控制高血压、心绞痛、糖尿病或其他疾病的药物治疗。
2) 进入随机分组阶段之后整个治疗期均不得使用研究药物以外的其他与试验药物成分类似中药。
3) 患者应当接受有利于心脏健康的饮食指导，如低盐饮食，适量饮水等，患者同时应当接受诸如监测体重、体育锻炼、戒烟、戒酒等适当生活方式改善的咨询。
4) 目前尚未发现禁止与芪苈强心胶囊伴随使用的药物。

4.4.4. 疗程：12 个月-36 个月

4.5. 依从性评价

通过完整记录药物的分发和回收情况来对受试者进行依从性评价，实际服用药物量在应用药物量的 80%~120%范围内，可判定为用药依从性符合方案要求。

4.6. 药物不良反应

目前，尚未发现芪苈强心胶囊存在明显的不良反应。

5. 研究人群

入组患者必须满足下文所列的所有入选标准，并且不符合任何一项排除标准。除下文所列标准外，接受标准化治疗期间，如果存在任何禁忌的医学状况或使用禁忌药物，也是排除患者入选的标准。

5.1. 入选标准

1) 自愿参加，理解并签署知情同意书；
2) 年龄≥18 岁，性别不限；
3) 有 3 个月以上的慢性心衰病史或临床发现心衰症状 3 个月以上；慢性心衰诊断参照当地指南或中华医学会心血管病学分会发布的“中国心力衰竭诊断和治疗指南 2018”；
4) 心脏彩超检查提示左室射血分数（LVEF）≤40％（改良辛普森法）；
5) NYHA 心功能分级II～III，临床症状稳定，包括入选前 2 周内曾诊断为IV级者；
6) 血清 NT-proBNP 含量≥ 450pg/ml；
7) 至少已接受 2 周以上的标准化药物治疗，且未调整过给药剂量及给予静脉治疗者；

标准化药物治疗包括：血管紧张素转换酶抑制剂（ACEI）或血管紧张素受体拮抗剂（ARB）或血管紧张素受体脑啡肽酶抑制剂(ARNI)、β 受体阻滞剂及醛固酮受体拮抗剂（除非禁忌或不耐受，应达到最佳治疗剂量）

5.2. 排除标准

1) 不符合入选标准；
2) 由于瓣膜病、先天性心脏病、心包疾病、心律失常及非心源性病因所致心衰，或肝、肾等重要脏器功能衰竭导致的心衰；及有明确肺源性或其他原因所致的右心衰、及急性心衰；
3) 计划于近期内行冠脉血运重建治疗者或心脏再同步化治疗者，已实施心脏再同步化治疗者；
4) 合并肝、肾、造血系统等严重原发性疾病，肾功能异常者，肝脏转氨酶、碱性磷酸酶超出 3 倍正常值上限，血肌酐＞2mg/dl(176.82umol/L)，血钾＞5.5mmol/L；肿瘤患者，严重神经内分泌系统疾病及精神病患者；
5) 存在左室流出道梗阻、心肌炎、大动脉瘤、夹层动脉瘤、致明显血液动力学改变的未修补的心脏瓣膜病患者；

6) 存在心源性休克、难以控制的恶性心律失常、二度II型以上未置入起搏器治疗的窦房或房室传导阻滞、进行性加重的不稳定心绞痛或急性心肌梗死者；
7) 未获控制的高血压患者，收缩压≥180/mmHg 和/或舒张压≥110mmHg；收缩压<90mmHg 和/或舒张压<60mmHg；
8) 1 个月内参加其他药物临床研究者；
9) 妊娠或正准备妊娠及哺乳期妇女；
10) 过敏体质者，或已知对治疗药物过敏者；
11) 根据研究者判断，患者不能完成本研究或不能遵守本研究的要求（由于管理方面的原因或其它原因）。

5.3. 中止研究药物治疗

随机分组后，任何原因暂停研究药物不等于永久停用，也不应该导致患者退出整个研究。相反对于已经停止服用研究药物的患者，也应该参加所有方案规定的研究访视和评价项目。如果患者不能参加研究访视，应按照计划通过电话继续随访，以确定是否发生任何不良事件和终点事件，除非患者拒绝随访并撤回知情同意书。

出现以下情况时可中止研究药物治疗：

1. 患者可在任何时间中止治疗
2. 发生与研究药物明确相关的过敏反应
3. 发生与研究药物明确相关的不良症状或体征、异常检查结果，研究者判断须终止研究的情况
4. 女性于研究期间发生妊娠

试验过程中应尽可能使患者长期服用标准剂量研究药物，中止研究药物患者在排除相关原因后应尽早恢复服用研究药物并按计划进行随访。

5.4. 退出标准

所有填写了知情同意书并筛选合格进入试验的受试者，无论何时何因退出，均不会影响其后续治疗。

患者有权在任何时间以任何理由退出研究，但应当尽量避免不必要的患者退出，并积极采取措施，尽可能完成随访，以备对其疗效和安全性进行分析。但当患者决定退出时，研究者应当通过电话或个人访问形式联系患者或其责任亲属并尽可能确认退出原因，研究者

应当在患者退出时回收剩余药物，完成最终评估，尽可能完成病例报告、解释退出原因，对退出患者发生终点事件进行随访。如果患者退出的原因为不良事件，则应记录于 CRF 内。

5.5. 全面中止试验标准

1) 研究进行中由于以下原因整个试验在多中心全面停止：

- 基于 DSMB 中期分析结果建议；
- 研究者发现严重安全性问题；
- 方案有重大失误；
- 资助方因经费或管理原因；
- 行政主管部门撤消试验，均可中途停止全部试验。

2) 全面中止试验可是暂时的，也可是永久的。中止试验时，全部试验记录应予保留备查。

6. 临床观察终点和指标

6.1. 临床观察指标

6.1.1. 主要有效终点

- 心血管死亡和心衰恶化再住院组成的复合终点事件发生率；

6.1.2. 次要有效终点

- 全因死亡率
- 复合终点事件（心衰恶化放弃治疗、心脏骤停后复苏成功、恶性心律失常、非致死性卒中）
- 冠心病心衰患者的心血管死亡和心衰恶化再住院发生率
- 血清 NT-proBNP 下降率

注：全部终点事件需经事件判定委员会复核判定。

6.2. 安全性指标包括：

- 不良事件评价
- 临床实验室指标：血常规（血红蛋白、红细胞、白细胞、血小板）、尿常规（尿蛋白、尿白细胞、尿红细胞）、血清生化（尿素氮、肌酐、谷丙转氨酶、空腹血糖、钾、钠、氯、总胆固醇、甘油三酯）。

- 12 导联心电图
- 体格检查

6.2.1. 不良事件定义

- 不良事件（**AEs**）：自受试者签署知情同意书并入选试验后开始至最后一次随访之间，发生任何不利医疗事件，无论与试验药物是否有因果关系，均判定为不良事件。
- 重要不良事件：除严重不良事件外，发生的任何导致针对性医疗措施（如停药，降低剂量和对症治疗）的不良事件和血液学和/或其他实验室异常。

6.2.2. 不良事件强度判定标准

在本临床研究中发生的所有临床不良事件将记录在 CRF 不良事件页上。并将不良事件严重程度进行分级。为统一标准，事件严重程度分级如下：

*严重程度分级：

1 级 轻度，无临床症状或有轻微临床症状；仅临床或实验室检查异常；不需治疗。

2 级 中度，需要微量的、局部的或非侵害性的治疗；与年龄相符的使用工具的日常生活活动[a]受限，使用工具的日常生活指做饭、购物、打电话等。

3 级 病情重或有医学上严重的症状但是暂时不会危及生命；导致住院或住院时间延长；导致残疾；日常生活自理[b]受限。日常生活自理指：洗澡、穿衣、脱衣、吃饭、去卫生间、吃药等，非卧床不起。

4 级 危及生命，需要紧急治疗。

5 级 因不良事件致死。

a：工具性日常生活活动是指做饭，购买日常用品或衣服，使用电话，理财等。

b：自理性日常生活活动是指洗澡，穿/脱衣，吃饭，盥洗，服药，并未卧床不起。

6.2.3. 不良事件与研究药物关系的判断标准

对所有不良事件与试验药物关系的因果分析，均按肯定有关、可能有关、可能无关、肯定无关、无法判定五级进行判断，对前三种定为药物的不良反应。因果分析的考虑因素有以下五个方面：

1) 肯定有关：AE 的发生和试验药物的使用有合理的时间顺序，AE 为试验药物已知的不良反应，停药后 AE 减轻或消失，再次用药重复出现，并无法用受试者本身疾病来解释。

2) 可能有关：AE 的发生和试验药物的使用有合理的时间顺序，AE 为试验药物已知或疑似的不良反应，但是，有其他因素可能引起该事件，如疾病、合并用药等；试验药物停用后反应减轻或消失，或药物停用后的效果不清楚，不清晰或缺乏决定性的信息。
3) 可能无关：AE 的发生和试验药物的使用有合理的时间顺序，但该事件不属于已知的药物不良反应类型，并极可能由受试者疾病或其他治疗引起。
4) 肯定无关：AE 的发生和试验药物的使用无合理的时间顺序，如事件在试验药物使用前已发生；不属于已知的药物不良反应；或 AE 确由其他因素导致，例如：受试者疾病、其他治疗或者合并用药引起等。
5) 无法判定：AE 出现的时间与用药的时间顺序无明确关系，不良事件与试验药物已知的反应类型相似，同时使用的其他药物也可能引起相同的反应，没有足够的依据判断。

以"肯定有关"、"可能有关"、"无法判定"三者合计为试验药物的不良反应，并据此计算不良反应发生率。

6.2.4. 严重不良事件

（1） 一般严重不良事件定义

严重不良事件是指任何提示显著危害、禁忌症、副作用或者需谨慎的临床事件。不良事件符合下面一条或以上标准时归为严重不良事件：

- 死亡
- 有生命危险（指出现该事件的患者在事件发生当时存在立即死亡的风险；并不包括那些如果更加严重将有可能导致患者死亡的事件）
- 导致住院或住院时间延长
- 导致持久或显著的劳动力丧失或残疾
- 先天性畸形缺陷

有些还没有导致死亡、生命危险或需住院的医疗事件，经过适当的医学判断，认为其可能对病人或受试者造成危害或需药物或外科手术治疗以避免上述情况发生时，也应视为 SAE。

（2） 严重不良事件的研究特异性定义

在本试验中，下列事件将不会作为严重不良事件报告，除非被判定阴性并且研究者认为与研究用药相关

◆ 心血管死亡

- ◆ 心衰恶化再住院
- ◆ 心衰恶化放弃治疗
- ◆ 心脏骤停后复苏成功
- ◆ 恶性心律失常
- ◆ 非致死性卒中

但是，所有其他导致致死性结局的事件都将被作为严重不良事件报告。

6.2.5. 不良事件的随访与记录

出现的不良事件，尤其是那些与试验药物相关的事件应当随访直至它们恢复至基线状态或者趋于稳定。如果经过随访，仍无法恢复基线状态或者稳定，那么应当在 CRF 中记录说明。临床试验过程中的任何严重不良事件，必须在 24 小时内报告临床试验监查人员、主要研究单位、药品生产企业。同时研究者必须填写严重不良反应表，记录严重不良事件的发生时间、严重程度、持续时间、采取的措施和转归。

6.2.6. 实验室结果异常

研究者应当对实验室结果异常是否具有临床意义进行判断，并给出可能的解释。已经被报告的不良事件导致的实验室异常结果应同时作为不良事件记录在不良事件表中。具有临床意义的实验室检查异常满足以下一项或多项条件者，应作为独立诊断记录在 CRF 的不良事件页中（不包括已被报告的不良事件导致的实验室结果异常）：

- 伴有临床症状的
- 导致研究用药改变的
- 需要改变合并用药和（或）其他治疗措施的

6.3. 终点事件的含义及 CEC 判定程序

6.3.1. 本研究终点事件的含义

- **心力衰竭住院是指满足下列所有标准的事件：**

1.患者因初步诊断为 HF 住院

2.患者住院时间延长至少 24 小时（或者如果不能获得住院时间和出院时间，指在日历上日期发生的变化）

3.在患者报告上记录由于 HF 出现新症状或者恶化症状，至少包括以下情况之一：

a.呼吸困难（用力时呼吸困难、休息时呼吸困难、端坐呼吸、夜间阵发性呼吸困难）

b.运动耐量减少

c.疲劳

4.患者具有新出现的恶化 HF 的客观证据，包括至少两种体检结果或一种体检结果和至少一种实验室标准，包括：

a.判断由 HF 导致的体检结果，包括新出现或恶化的：

1)外周性水肿。 2)腹胀或腹水增加（在无原发性肝病的情况下）。 3)肺啰音/爆裂音/湿性啰音。 4)颈静脉压升高和/或肝颈静脉回流。 5)S3 奔马律。 6)具有临床意义的或迅速的体重增加，考虑与体液潴留有关

b.在 24 小时内获得的，新出现或者恶化 HF 的实验室证据，包括：

1)与 HF 失代偿一致（如 BNP>500pg/ml 或 NT-proBNP>2000pg/ml）的 B 型利钠肽（BNP）/N 末端 B 型利钠肽前体（NT-proBNP）浓度增加。在利钠肽长期升高的患者中，应特别关注超过基线的显著增加。 2)肺充血的放射影像学证据。3)具有临床意义的左侧或者右侧心室充盈压升高或者心输出量降低的非侵害性诊断证据。如超声心动图标准包括：E/e′>15 或者 D 主导肺静脉流入模式，充血性下腔静脉伴有极小程度吸气塌陷，或者左心室流出量（LVOT）微小行程距离减小（时间流速积分（TVI））。4)侵入性诊断证据：右心导管检查显示肺毛细血管楔压（肺动脉闭塞压）≧18mmHg，中央静脉压≧12mmHg，或心排血量指数＜2.2L/min/m^2。

注：如适用，即使不满足上述标准，仍需报告诊断检查中的所有结果，为上述事件的裁定提供重要信息。

5.患者接受针对 HF 的初期或者强化治疗，包括下列至少一种：

a.增强口服利尿药的治疗

b.静脉注射利尿药或者血管活性药物（如正性肌力药、血管升压类药物或者血管扩张剂）

c.机械或手术干预，包括：

1)机械循环支持（例如，主动脉球囊反搏、心室辅助装置、体外膜氧合、全人工心脏）

2)机械辅助去除体液（例如，超滤、血液滤过、透析）

- **心血管死亡：**包括急性心肌梗死（MI），心源性猝死，心力衰竭（HF）导致的死亡，中风导致的死亡，心血管（CV）手术导致的死亡，CV 出血以及其他 CV 原因造成的死亡。
- **全因死亡**
- **心衰恶化放弃治疗：**心衰症状和体征不断加重，需要静脉药物或机械支持治疗而患者或患者家属主动放弃治疗或自动出院，若随访其后果为死亡则列入心力衰竭死亡。
- **心脏骤停后复苏成功**
- **恶性心律失常：**对于恶性心律失常的定义， 目前还没有统一的标准，一般是指能在短时间内引起严重血流动力学障碍，导致患者晕厥甚至猝死的心律失常 。根据这个标准，恶性心律失常主要有如下类别：(1) 严重的缓慢型心律失常， 如严重的病态窦房结综合征、高度或三度房室传导阻滞；(2) 快速型心律失常， 如持续性室性心动过速、心室扑动、心室颤动， 快室率心房扑动、心房颤动、房室折返性心动过速、预激综合征伴心房颤动、窦性心动过速等。
- **非致死性卒中**

6.3.2. 终点事件发生时的评估与程序

研究者获知终点事件发生后，应在 7 天内收集相关支持文件报告事件判定委员会。终点事件将由独立的事件判定委员会（CEC）进行复核，因此终点事件报告表将作为 CRF 的一部分，研究者将在上述表格内记录事件并及时提交支持文件（入院与出院记录、病历记录、死亡记录、ECG 等）上述资料将提供给 CEC 以对事件进行判定。

CEC 由主席及 5-6 名成员组成，每一例事件将由委员会的两位成员进行独立审查并将结论提交至委员会主席处。如果两位审查委员之间或两位审查委员与主席的意见不一致，当具有异议的事件积累到一定数量时，整个委员会将安排会议对事件进行审查。

7. 数据管理

本研究采用 Epidata 软件或 EDC 系统进行研究数据的采集。数据管理确保临床试验数据的真实性、完整性和准确性，数据管理过程需符合《药物临床试验质量管理规范》、《临床试验数据管理工作技术指南》等法规要求，保证临床试验数据的可溯源性。以下列出数据管理的主要流程。

7.1. 数据库设计

数据管理员根据 CRF 采用 Epidata 软件或 EDC 系统设计数据库，经测试后发布。

7.2. 数据录入

CRC 负责将 CRF 中的数据录入数据库，数据录入采用二次录入方式，由两名 CRC 分别录入一遍数据，数据管理员对两个数据库进行比对，产生数据不一致清单，CRC 按照清单对照 CRF 分别修改各自的数据库，然后再进行比对，重复以上步骤，直至两个数据库完全一致。

7.3. 数据质疑管理

数据管理员依据数据核查计划（DVP）编写数据核查 SAS 程序对数据进行核查，产生数据质疑清单，经人工核对后，生成数据质疑表，由 CRA 交研究者进行答疑，答疑后的质疑表再由 CRA 返还给数据管理员，数据管理员据此修订数据库。

7.4. 医学编码

不良事件编码采用 MedDRA21.0 或者更新版本。

7.5. 数据审核

数据库清理完成后，数据管理员撰写《数据核查报告》，用于召开数据核查会议。

审核报告重点记录内容为：入组病例数、脱落、剔除病例情况、偏离或违背方案情况、依从性数据，合并用药，不良事件，与评价指标有关的数据等。

数据审核会议上，针对审核报告的内容，讨论并确定统计人群的划分。

7.6. 数据库锁定

完成数据库锁库清单，依据数据库锁定程序完成数据库锁定。数据锁定之后发现的问题，经确认后可在统计分析程序中修正。数据锁定后如有确切证据证明有必要解锁，研究者及相关人员需签署解锁文件。

数据库锁定后，由数据管理员导出 SAS 格式的数据文件，交与统计人员进行统计分析。

8. 统计分析

试验方案确定后，由统计专业人员负责与主要研究者协商制订统计分析计划书。统计分析软件采用 SAS®9.4 软件（或更高版本）。样本量计算软件采用 PASS13。

8.1. 分析人群

研究人群分为以下几类：

- 全分析集(FAS)：是指尽可能接近意向性分析原则（intention to treat）、从所有随机化的受试者中，以最少的和合理的方法剔除受试者后得出的数据集，包含所有经过随机化并使用过一次研究药物的受试者。剔除通常包括：违反重要入组标准；受试者未接受试验用药物的治疗；随机化后无任何观测数据。主要疗效评价指标为发生复合终点事件的时间，采用生存分析的方法进行分析，在选择 FAS 进行统计分析时，对于主要终点事件的缺失按照删失处理。
- 符合方案集(PPS)：是全分析集的一个子集，这些受试者对方案更具依从性。纳入 PPS 受试者一般具有以下特征：（1）完成事先设定的试验药物的最小暴露量，即服用药物的依从性达到 80%；（2）试验中主要指标的数据均可以获得；（3）未对试验方案有重大的违背。
- 安全性分析集（SS）：所有随机化后至少接受一次治疗且有安全性评价的受试者。安全性缺失值无需结转。

疗效分析将在 FAS 和 PPS 的基础上进行。所有基线人口统计学资料分析将在 FAS 的基础上进行，安全性评价将在 SS 上进行

8.2. 统计分析方法

- 所有的统计检验均采用双侧检验，*P* 值小于或等于 0.05 将被认为所检验的差别有统计意义。(特别说明的除外)
- 描述性分析：分类指标描述各类的例数及百分数。定量指标采用均数、标准差、最大值、最小值、中位数、下四分位数（Q1）和上四分位数（Q3）描述。
- 对两组一般情况的比较将根据指标的类型采用适当的方法进行分析，定量资料的组间比较采用成组 t 检验或 Wilcoxon 秩和检验，分类数据采用卡方检验或精确概率法，等级资料采用 Wilcoxon 秩和检验或 CMH 检验。

8.2.1. 入组及完成情况

总结各中心入组及完成数，列出脱落病例的清单。各组不同数据集大小，各中心病例分布，总脱落率比较，终止原因详细列表。对患者的人口学特征(年龄、身高、生命体征等)、病史及用药史等进行描述，并对两组年龄、身高、体重等进行比较，以衡量两组的可比性。

8.2.2. 依从性分析

- 用药依从性分析：比较两组病人是否按时按量使用试验药物，未用方案中禁用的药物和食物。
- 合并用药分析：需统计各组合并用药人数，并详细列表。

8.2.3. 疗效评价

疗效评价同时进行 PP 分析和 FAS 分析；

- **主要疗效评价指标**为发生复合终点事件（心血管死亡和心衰恶化再住院）的时间。

对于主要终点事件的缺失按照删失处理。

本研究的主要研究假设为：

H_0：$\lambda_T/\lambda_C \geq 1$

H_1：$\lambda_T/\lambda_C < 1$

其中，λ_T 和 λ_C 分别为试验组和对照组发生终点事件的风险。

利用 Kaplan-Meier 法估计临床终点事件发生率，两组之间进行 Log rank 检验。利用 COX 比例风险模型，以中心为协变量，计算两组间的风险比（Hazard Ratio）及其 95%可信区间。另外，对复合终点事件的两个部分分别进行分析，即心血管死亡和心衰恶化再住院。

- **次要疗效指标：**

全因死亡率：利用 Kaplan-Meier 法估计两组全因死亡率，并进行 Log-rank 检验。利用 COX 比例风险模型，以中心为协变量，估计两组间的风险比（Hazard Ratio）及其 95%可信区间。

复合终点事件（心衰恶化放弃治疗、心脏骤停后复苏成功、恶性心律失常、非致死性卒中）：分析方法与全因死亡相同。

冠心病心衰患者的心血管死亡和心衰恶化再住院事件：按计数资料统计分析；

血清 NT-proBNP：按计量资料分析，对两组血清 NT-proBNP 水平进行统计描述和组间比较，并对两组与基线的变化情况进行统计描述和组间比较。

8.2.4. 安全性评价

安全性评价基于 SS 数据集进行分析。

不良事件用不良事件发生例次、例数及发生率进行描述，并对该发生率进行组间显著性检验。同时，列表详细描述各组病例出现的全部不良事件的具体表现、程度及其与药物的关系。

对实验室指标前后变化情况进行交叉表描述，按试验组和对照组分别描述治疗前正常、治疗后异常例数及该例数所占比例。对生命体征指标进行前后比较。

8.3. 期中分析

本研究计划在收集到 1/2 和 2/3 主要终点事件后进行两次期中疗效分析，以评估是否已得出有效的结论而提前终止该研究。根据 Lan-DeMets α 消耗函数和 O’Brien-Fleming 方法，第 1 次期中分析时消耗的 I 类错误 $\alpha=0.0001$（单侧），第 2 次期中分析消耗 $\alpha=0.00605$（单侧）。

期中分析有关的具体要求和操作将在 DSMB 章程中事先规定。

9. 统计分析结果

9.1. 病例分布（所有随机化人群）

表9.1.1 各中心病例分布

中心	组别	入组数	脱落数	脱落率 (%)	剔除数	剔除率 (%)	完成
南京医科大学第一附属医院	试验组						
	对照组						
	合计						
河北以岭医院	试验组						
	对照组						
	合计						
……	试验组						
	对照组						
	合计						
合计	试验组						
	对照组						
	合计						

表9.1.2 两组脱落剔除情况比较

组别	脱落率(%)	P	剔除率(%)	P
试验组				
对照组				

注：脱落是指未完成试验的受试者，剔除是指完成试验但剔除 PPS 集的受试者。

表9.1.3 各中心的人群划分情况描述

中心	PPS			FAS			SS		
	试验组	对照组	合计	试验组	对照组	合计	试验组	对照组	合计
南京医科大学第一附属医院									
河北以岭医院									
……									
合计									

注：下表开始中心都以中心编号表示

表9.1.4　入组病例及安全性有效性分析数据集

项目	试验组	对照组	合计
全数据集			
随机入组			
完成试验			
试验期间中止			
不符合入选标准或属于排除标准			
不良事件			
缺乏疗效			
违背试验方案(包括依从性差)			
撤回知情同意书			
失访或患者未按时来院复诊			
其他原因			
安全性分析集			
SS			
有效性分析集			
FAS			
PPS			

9.2. 人口学和基线信息(FAS)

表9.2.1 人口学资料

项目	指标	试验组	对照组	合计	统计量	P
年龄（岁）	N(Missing)					
	Mean(Sd)					
	Median					
	Q1,Q3					
	Min,Max					
性别	男 n(%)					
	女 n(%)					
	合计(缺失)					
婚姻	未婚 n(%)					
	已婚 n(%)					
	其他 n(%)					
	合计(缺失)					
民族	汉族 n(%)					
	其他 n(%)					
	合计(缺失)					
身高(cm)	N(Missing)					
	Mean(Sd)					
	Median					
	Q1,Q3					
	Min,Max					
体重(kg)	N(Missing)					
	Mean(Sd)					
	Median					
	Q1,Q3					
	Min,Max					
BMI(kg/m^2)	N(Missing)					
	Mean(Sd)					
	Median					
	Q1,Q3					
	Min,Max					
国籍	中国 n(%)					
	其他 n(%)					
	合计(缺失)					

表9.2.2 个人史

项目	指标	试验组	对照组	合计	统计量	P
吸烟史	有 n(%)					
	无 n(%)					

项目	指标	试验组	对照组	合计	统计量	P
	已戒烟 n(%)					
	合计(缺失)					
吸烟数量(支/日)	N(Missing)					
	Mean(Sd)					
	Median					
	Q1,Q3					
	Min,Max					
饮酒史	有 n(%)					
	无 n(%)					
	已戒酒 n(%)					
	合计(缺失)					
饮酒数量（两/日	N(Missing)					
	Mean(Sd)					
	Median					
	Q1,Q3					
	Min,Max					
月经史	未绝经 n(%)					
	已绝经 n(%)					
	不适用 n(%)					
	合计(缺失)					
药物过敏史	有 n(%)					
	无 n(%)					
	合计(缺失)					

表9.2.3　疾病情况和既往病史

项目	指标	试验组	对照组	合计	统计量	P
冠心病	否 n(%)					
	是 n(%)					
	合计(缺失)					
心肌病	否 n(%)					
	是 n(%)					
	合计(缺失)					
高血压性心脏病	否 n(%)					
	是 n(%)					
	合计(缺失)					
其他	否 n(%)					
	是 n(%)					
	合计(缺失)					
心衰病程	≤3 年					
	>3 年					

项目	指标	试验组	对照组	合计	统计量	P
	合计(缺失)					
心衰病程(月)	N(Missing)					
	Mean(Sd)					
	Median					
	Q1,Q3					
	Min,Max					

表9.2.4　重要既往病史分析

项目	指标	试验组	对照组	合计	统计量	P
心肌梗死	无 n(%)					
	有 n(%)					
	合计(缺失)					
高血压	无 n(%)					
	有 n(%)					
	合计(缺失)					
糖尿病	无 n(%)					
	有 n(%)					
	合计(缺失)					
高脂血症	无 n(%)					
	有 n(%)					
	合计(缺失)					
脑卒中	无 n(%)					
	有 n(%)					
	合计(缺失)					
房颤	无 n(%)					
	有 n(%)					
	合计(缺失)					
心率失常	无 n(%)					
	有 n(%)					
	合计(缺失)					

注：重要病史按既往病史编码后的首选术语进行归类。

表9.2.5　既往病史分析（SOC/PT）

项目	试验组			对照组			P 值
	例次	人数	百分率	例次	人数	百分率	
既往病史							

表9.2.6　重要病史（PT）

项目	试验组 例次	试验组 人数	试验组 百分率	对照组 例次	对照组 人数	对照组 百分率	P 值
高血压							
糖尿病							
高脂血症							
房颤							
心肌梗死							
脑卒中							
心律失常							

以上重要病史按既往病史编码后的首选术语进行归类。

表9.2.7　NYHA 心功能分级

项目	指标	试验组	对照组	合计	统计量	P
NYHA 心功能分级	1.I级，日常活动无心衰症状；（※I级者，请不要入组）n(%)					
	2.II级，日常活动出现心衰症状（呼吸困难、乏力）；n(%)					
	3.III级，低于日常活动出现心衰症状；n(%)					
	4.IV级，在休息时出现心衰症状；（※IV级者，需考虑试验期间标准					
	合计(缺失)					

表9.2.8　心脏彩色超声检查

项目	指标	试验组	对照组	合计	统计量	P
是否检测	是 n(%)					
	否 n(%)					
	合计(缺失)					
左室射血分数(LVEF)(%)	N(Missing)					
	Mean(Sd)					
	Median					
	Q1,Q3					
	Min,Max					

表9.2.9 血清 NT-proBNP 检测

项目	指标	试验组	对照组	合计	统计量	P
是否检测-访视 1	是 n(%)					
	否 n(%)					
	合计(缺失)					
NT-proBNP (pg/ml)	N(Missing)					
	Mean(Sd)					
	Median					
	Q1,Q3					
	Min,Max					
是否检测-访视 2	是 n(%)					
	否 n(%)					
	合计(缺失)					
NT-proBNP (pg/ml)	N(Missing)					
	Mean(Sd)					
	Median					
	Q1,Q3					
	Min,Max					
是否检测-访视 3	是 n(%)					
	否 n(%)					
	合计(缺失)					
NT-proBNP (pg/ml)	N(Missing)					
	Mean(Sd)					
	Median					
	Q1,Q3					
	Min,Max					

表9.2.10 生命体征

项目	指标	试验组	对照组	合计	统计量	P
收缩压(mmHg)	N(Missing)					
	Mean(Sd)					
	Median					
	Q1,Q3					
	Min,Max					
舒张压(mmHg)	N(Missing)					
	Mean(Sd)					
	Median					
	Q1,Q3					
	Min,Max					
心率(次/分)	N(Missing)					
	Mean(Sd)					
	Median					
	Q1,Q3					
	Min,Max					

项目	指标	试验组	对照组	合计	统计量	P
脉搏(次/分)	N(Missing)					
	Mean(Sd)					
	Median					
	Q1,Q3					
	Min,Max					
体温(℃)	N(Missing)					
	Mean(Sd)					
	Median					
	Q1,Q3					
	Min,Max					

9.3. 中位随访时间

表9.3.1 随访时间描述

		FAS		PPS	
项目	指标	试验组	对照组	试验组	对照组
随访时间(天)	N(Missing)				
	Mean(Sd)				
	Median				
	Q1,Q3				
	Min,Max				
两组比较	统计量				
	P 值				

表9.3.2 中位随访时间

		FAS		PPS	
项目	指标	试验组	对照组	试验组	对照组
随访	N(Missing)				
	终点事件(%)				
	删失(%)				
	中位随访时间				
	95%CI				
	Q1,Q3				
	Log-rank 检验				
	P 值				
Cox 回归	Hazard Ratio				
	95%CI				
	Wald 卡方				
	P 值				

9.4. 用药情况(SS)

表9.4.1 用药依从性

项目	指标	试验组	对照组	合计	统计量	P 值
用药依从性	<80%n(%)					
	80%-120%n(%)					
	>120%n(%)					
	合计(Missing)					

注：实际服用药物量在应用药物量的 80%~120%范围内，可判定为用药依从性符合方案要求.

表9.4.2 既往用药/合并用药

项目	指标	试验组	对照组	合计	统计量	P
是否有既往用药/合并用药	否 n(%)					
	是 n(%)					
	合计(缺失)					

表9.4.3 合并用药编码（ATC1/ATC2）

	试验组			对照组			
项目	例次	人数	百分率	例次	人数	百分率	P 值
ATC1							
ATC2							

9.5. 主要疗效指标评价（FAS&PPS）

表9.6.1 发生复合终点事件（心血管死亡和心衰恶化再住院）的时间

		FAS		PPS	
项目	指标	试验组	对照组	试验组	对照组
复合终点事件发生时间	N(Missing)				
	终点事件(%)				
	删失(%)				
	中位生存时间				
	95%CI				
	Q1,Q3				
	Log-rank 检验				
	P 值				
Cox 回归	Hazard Ratio				
	95%CI				
	Wald 卡方				
	P 值				

*Cox 回归协变量为中心，下同。

图 1 发生复合终点事件 KM 曲线图（FAS 数据集）

图 2 发生复合终点事件 KM 曲线图（PPS 数据集）

表9.6.2　发生心血管死亡的时间

项目	指标	FAS 试验组	FAS 对照组	PPS 试验组	PPS 对照组
心血管死亡发生时间	N(Missing)				
	终点事件(%)				
	删失(%)				
	中位发生时间				
	95%CI				
	Q1,Q3				
	Log-rank 检验				
	P 值				
Cox 回归	Hazard Ratio				
	95%CI				
	Wald 卡方				
	P 值				

*Cox 回归协变量为中心，下同。

图 3 心血管死亡 KM 曲线图（FAS 数据集）

图 4 心血管死亡 KM 曲线图（PPS 数据集）

表9.6.3　发生心衰恶化再住院的时间

项目	指标	FAS 试验组	FAS 对照组	PPS 试验组	PPS 对照组
心衰恶化再住院发生时间	N(Missing)				
	终点事件(%)				
	删失(%)				
	中位发生时间				
	95%CI				
	Q1,Q3				
	Log-rank 检验				
	P 值				
Cox 回归	Hazard Ratio				
	95%CI				
	Wald 卡方				
	P 值				

图 5 心衰恶化再住院 KM 曲线图（FAS 数据集）

图 6 心衰恶化再住院 KM 曲线图（PPS 数据集）

9.6. 主要疗效指标评价-亚组分析

表9.6.1 复合终点事件（心血管死亡和心衰恶化再住院）（≤70 岁）

		FAS		PPS	
项目	指标	试验组	对照组	试验组	对照组
复合终点事件发生时间	N(Missing)				
	终点事件(%)				
	删失(%)				
	中位生存时间				
	95%CI				
	Q1,Q3				
	Log-rank 检验				
	P 值				
Cox 回归	Hazard Ratio				
	95%CI				
	Wald 卡方				
	P 值				

表9.6.2 复合终点事件（心血管死亡和心衰恶化再住院）（＞70 岁）

		FAS		PPS	
项目	指标	试验组	对照组	试验组	对照组
复合终点事件发生时间	N(Missing)				
	终点事件(%)				
	删失(%)				
	中位生存时间				
	95%CI				
	Q1,Q3				
	Log-rank 检验				
	P 值				
Cox 回归	Hazard Ratio				
	95%CI				
	Wald 卡方				
	P 值				

表9.6.3 发生心血管死亡的时间（≤70 岁）

下述亚组分析结果，表格形式同上

表9.6.4 发生心血管死亡的时间（>70 岁）

表9.6.5 发生心衰恶化再住院的时间（≤70 岁）

表9.6.6 发生心衰恶化再住院的时间（>70 岁）

表9.6.7 复合终点事件（心血管死亡和心衰恶化再住院）（男性）

表9.6.8 复合终点事件（心血管死亡和心衰恶化再住院）（女性）

表9.6.9 发生心血管死亡的时间（男性）

表9.6.10 发生心血管死亡的时间（女性）

表9.6.11 发生心衰恶化再住院的时间（男性）

表9.6.12 发生心衰恶化再住院的时间（女性）

表9.6.13 复合终点事件（心血管死亡和心衰恶化再住院）（NYHA 分级 I 级或 II 级）

表9.6.14 复合终点事件（心血管死亡和心衰恶化再住院）（NYHA 分级 III 级或IV级）

表9.6.15 发生心血管死亡的时间（NYHA 分级 I 级或 II 级）

表9.6.16 发生心血管死亡的时间（NYHA 分级 III 级或IV级）

表9.6.17 发生心衰恶化再住院的时间（NYHA 分级 I 级或 II 级）

表9.6.18 发生心衰恶化再住院的时间（NYHA 分级 III 级或IV级）

表9.6.19 复合终点事件（心血管死亡和心衰恶化再住院）（LVEF≤30%）

表9.6.20 复合终点事件（心血管死亡和心衰恶化再住院）（LVEF>30%）

表9.6.21 发生心血管死亡的时间（LVEF≤30%）

表9.6.22 发生心血管死亡的时间（LVEF>30%）

表9.6.23 发生心衰恶化再住院的时间（LVEF≤30%）

表9.6.24 发生心衰恶化再住院的时间（LVEF>30%）

表9.6.25 复合终点事件（心血管死亡和心衰恶化再住院）（心衰病程≤3 年）

表9.6.26 复合终点事件（心血管死亡和心衰恶化再住院）（心衰病程>3 年）

表9.6.27 发生心血管死亡的时间（心衰病程≤3 年）

表9.6.28 发生心血管死亡的时间（心衰病程>3 年）

表9.6.29 发生心衰恶化再住院的时间（心衰病程≤3 年）

表9.6.30 发生心衰恶化再住院的时间（心衰病程>3 年）

表9.6.31 复合终点事件（心血管死亡和心衰恶化再住院）（有冠心病）

表9.6.32 复合终点事件（心血管死亡和心衰恶化再住院）（无冠心病）

表9.6.33 发生心血管死亡的时间（有冠心病）

表9.6.34 发生心血管死亡的时间（无冠心病）

表9.6.35 发生心衰恶化再住院的时间（有冠心病）

表9.6.36 发生心衰恶化再住院的时间（无冠心病）

表9.6.37 复合终点事件（心血管死亡和心衰恶化再住院）（有心肌病）

表9.6.38 复合终点事件（心血管死亡和心衰恶化再住院）（无心肌病）

表9.6.39 发生心血管死亡的时间（有心肌病）

表9.6.40 发生心血管死亡的时间（无心肌病）

表9.6.41 发生心衰恶化再住院的时间（有心肌病）

表9.6.42 发生心衰恶化再住院的时间（无心肌病）

表9.6.43 复合终点事件（心血管死亡和心衰恶化再住院）（有高血压病）

表9.6.44 复合终点事件（心血管死亡和心衰恶化再住院）（无高血压病）

表9.6.45 发生心血管死亡的时间（有高血压病）

表9.6.46 发生心血管死亡的时间（无高血压病）

表9.6.47 发生心衰恶化再住院的时间（有高血压病）

表9.6.48 发生心衰恶化再住院的时间（无高血压病）

表9.6.49 复合终点事件（心血管死亡和心衰恶化再住院）（有糖尿病）

表9.6.50 复合终点事件（心血管死亡和心衰恶化再住院（无糖尿病）

表9.6.51 发生心血管死亡的时间（有糖尿病）

表9.6.52 发生心血管死亡的时间（无糖尿病）

表9.6.53 发生心衰恶化再住院的时间（有糖尿病）

表9.6.54 发生心衰恶化再住院的时间（无糖尿病）

表9.6.55 复合终点事件（心血管死亡和心衰恶化再住院）（有房颤）

表9.6.56 复合终点事件（心血管死亡和心衰恶化再住院）（无房颤）

表9.6.57 发生心血管死亡的时间（有房颤）

表9.6.58 发生心血管死亡的时间（无房颤）

表9.6.59 发生心衰恶化再住院的时间（有房颤）

表9.6.60 发生心衰恶化再住院的时间（无房颤）

表9.6.61 复合终点事件（心血管死亡和心衰恶化再住院）（有心率失常）

表9.6.62 复合终点事件（心血管死亡和心衰恶化再住院）（无心率失常）

表9.6.63 发生心血管死亡的时间（有心率失常）

表9.6.64 发生心血管死亡的时间（无心率失常）

表9.6.65 发生心衰恶化再住院的时间（有心率失常）

表9.6.66 发生心衰恶化再住院的时间（无心率失常）

表9.6.67 复合终点事件（心血管死亡和心衰恶化再住院）（BNP≤中位数）

注：中位数为总体（FAS 人群中）基线 BNP 中位数。

表9.6.68 复合终点事件（心血管死亡和心衰恶化再住院）（BNP>中位数）

注：中位数为总体（FAS 人群中）基线 BNP 中位数值。

表9.6.69 发生心血管死亡的时间（BNP≤中位数）

注：中位数为总体（FAS 人群中）基线 BNP 中位数值。

表9.6.70 发生心血管死亡的时间（BNP>中位数）

注：中位数为总体（FAS 人群中）基线 BNP 中位数值。

表9.6.71 发生心衰恶化再住院的时间（BNP≤中位数）

注：中位数为总体（FAS 人群中）基线 BNP 中位数值。

表9.6.72 发生心衰恶化再住院的时间（BNP>中位数）

注：中位数为总体（FAS 人群中）基线 BNP 中位数值。

9.7. 次要疗效指标评价（FAS&PPS）

表9.7.1 全因死亡率

项目	指标	FAS 试验组	FAS 对照组	PPS 试验组	PPS 对照组
全因死亡	N(Missing)				
	终点事件(%)				
	删失(%)				
	中位生存时间				
	95%CI				
	Q1,Q3				
	Log-rank 检验				
	P 值				
Cox 回归	Hazard Ratio				
	95%CI				
	Wald 卡方				
	P 值				

表9.7.2 复合终点事件（心衰恶化放弃治疗、心脏骤停后复苏成功、恶性心律失常、非致死性卒中）率

项目	指标	FAS 试验组	FAS 对照组	PPS 试验组	PPS 对照组
复合终点事件（心衰恶化放弃治疗、心脏骤停后复苏成功、恶性心律失常、非致死性卒中）	N(Missing)				
	终点事件(%)				
	删失(%)				
	中位生存时间				
	95%CI				
	Q1,Q3				
	Log-rank 检验				
	P 值				
Cox 回归	Hazard Ratio				
	95%CI				
	Wald 卡方				
	P 值				

表9.7.3 心衰恶化放弃治疗率

项目	指标	FAS 试验组	FAS 对照组	PPS 试验组	PPS 对照组
心衰恶化放弃治疗	N(Missing)				
	终点事件(%)				
	删失(%)				

项目	指标	FAS 试验组	FAS 对照组	PPS 试验组	PPS 对照组
	中位生存时间				
	95%CI				
	Q1,Q3				
	Log-rank 检验				
	P 值				
Cox 回归	Hazard Ratio				
	95%CI				
	Wald 卡方				
	P 值				

表9.7.4　心脏骤停后复苏成功率

项目	指标	FAS 试验组	FAS 对照组	PPS 试验组	PPS 对照组
心脏骤停后复苏成功	N(Missing)				
	终点事件(%)				
	删失(%)				
	中位生存时间				
	95%CI				
	Q1,Q3				
	Log-rank 检验				
	P 值				
Cox 回归	Hazard Ratio				
	95%CI				
	Wald 卡方				
	P 值				

表9.7.5　恶性心律失常率

项目	指标	FAS 试验组	FAS 对照组	PPS 试验组	PPS 对照组
恶性心律失常	N(Missing)				
	终点事件(%)				
	删失(%)				
	中位生存时间				
	95%CI				
	Q1,Q3				
	Log-rank 检验				
	P 值				
Cox 回归	Hazard Ratio				

项目	指标	FAS 试验组	FAS 对照组	PPS 试验组	PPS 对照组
	95%CI				
	Wald 卡方				
	P 值				

表9.7.6　非致死性卒中率

项目	指标	FAS		PPS	
		试验组	对照组	试验组	对照组
非致死性卒中	N(Missing)				
	终点事件(%)				
	删失(%)				
	中位生存时间				
	95%CI				
	Q1,Q3				
	Log-rank 检验				
	P 值				
Cox 回归	Hazard Ratio				
	95%CI				
	Wald 卡方				
	P 值				

表9.7.7　冠心病心衰患者的心血管死亡和心衰恶化再住院

项目	指标	FAS		PPS	
		试验组	对照组	试验组	对照组
复合事件	是 n(%)				
	否 n(%)				
	合计(缺失)				
	统计量				
	P 值				
心血管死亡	是 n(%)				
	否 n(%)				
	合计(缺失)				
	统计量				
	P 值				
心衰恶化再住院	是 n(%)				
	否 n(%)				
	合计(缺失)				
	统计量				
	P 值				

表9.7.8 血清 NT-proBNP 较基线变化情况（1 个月）

项目	指标	FAS 试验组	FAS 对照组	PPS 试验组	PPS 对照组
基线 NT-proBNP(pg/ml)	N(Missing)				
	Mean(Sd)				
	Median				
	Q1,Q3				
	Min,Max				
基线两组比较	统计量				
	P 值				
治疗 1 个月	N(Missing)				
	Mean(SD)				
	Median				
	Q1,Q3				
	Min,Max				
治疗 1 个月两组比较	统计量				
	P 值				
前后差值	N(Missing)				
	Mean(SD)				
	Median				
	Q1,Q3				
	Min,Max				
组内前后比较	统计量				
	P 值				
前后差值两组比较	统计量				
	P 值				

表9.7.9 血清 NT-proBNP 较基线变化情况（3 个月）

表格同上

表9.7.10 血清 NT-proBNP 较基线下降率（1 个月）

项目	指标	FAS 试验组	FAS 对照组	PPS 试验组	PPS 对照组
下降率（%）	N(Missing)				
	Mean(Sd)				
	Median				
	Q1,Q3				
	Min,Max				

项目	指标	FAS 试验组	FAS 对照组	PPS 试验组	PPS 对照组
两组比较	统计量				
	P 值				
下降率≥30%	否 n(%)				
	是 n(%)				
	合计(缺失)				
两组比较	统计量				
	P 值				

注：下降率=（基线结果-1 个月结果）/基线结果*100%

表9.7.11 血清 NT-proBNP 较基线下降率（3 个月）

表格同上

9.8. 安全性评价（SS）

表9.8.1 不良事件汇总比较

项目	指标	试验组	对照组	合计	统计量	P
是否不良事件	否 n(%)					
	是 n(%)					
	合计(缺失)					
是否严重不良事件	否 n(%)					
	是 n(%)					
	合计(缺失)					

表9.8.2 研究期间不良事件汇总比较

项目	试验组 例次	试验组 人数	试验组 百分率	对照组 例次	对照组 人数	对照组 百分率	P 值
不良事件							
与研究药物相关不良事件							
严重程度 3 4 5 级不良事件							
严重不良事件							
与研究药物相关的严重不良事件							
导致脱落的不良事件							
导致脱落的与研究药物相关的不良事件							

注：与研究药物相关定义为肯定有关、可能有关、无法判定。

表9.8.3　不良事件编码情况（SOC/PT）

项目	试验组			对照组			P 值
	例次	人数	百分率	例次	人数	百分率	
不良事件							

表9.8.4　不良反应编码情况（SOC/PT）

项目	试验组			对照组			P 值
	例次	人数	百分率	例次	人数	百分率	
不良事件							

表9.8.5　生命体征前后比较(12 个月±7 天)-收缩压（mmHg）

项目	指标	试验组	SS<br>对照组
基线收缩压	N(Missing)		
	Mean(Sd)		
	Median		
	Q1,Q3		
	Min,Max		
	统计量		
	P 值		
入组 12 个月收缩压	N(Missing)		
	Mean(SD)		
	Median		
	Q1,Q3		
	Min,Max		
入组 12 个月两组比较	统计量		
	P 值		
前后差值	N(Missing)		
	Mean(SD)		
	Median		
	Q1,Q3		
	Min,Max		
组内前后比较	统计量		
	P 值		
前后差值两组比较	统计量		
	P 值		
前后变化率	N(Missing)		
	Mean(SD)		

项目	指标	SS<br>试验组	对照组
	Median		
	Q1,Q3		
	Min,Max		
组内前后变化率比较	统计量		
	P 值		
两组前后变化率比较	统计量		
	P 值		

表9.8.6 生命体征前后比较(12 个月±7 天)-舒张压（mmHg）

表格同上

表9.8.7 生命体征前后比较(12 个月±7 天)-心率（次/分）

表格同上

表9.8.8 生命体征前后比较(12 个月±7 天)-脉搏（次/分）

表格同上

表9.8.9 生命体征前后比较(12 个月±7 天)-体温（℃）

表格同上

表9.8.10 血常规-12 个月±7 天白细胞总数前后交叉表

		白细胞总数					
组别	用药前	正常	异常无意义	异常有意义	未查	缺失	合计
试验组							
	正常						
	异常无意义						
	异常有意义						
	未查						
	缺失						
	合计						
对照组							
	正常						
	异常无意义						
	异常有意义						
	未查						
	缺失						
	合计						

表9.8.11 血常规-12 个月±7 天血红蛋白前后交叉表

下述交叉表，表格格式同上

表9.8.12 血常规-12 个月±7 天红细胞总数前后交叉表

表9.8.13 血常规-12 个月±7 天血小板前后交叉表

表9.8.14 血常规-12 个月±7 天汇总表（SS）

指标	组别	用药前正常<br>用药后正常	用药前正常<br>用药后异常	用药前正常<br>用药后正常	用药前正常<br>用药后异常
白细胞总数（WBC）	试验组				
	对照组				
血红蛋白（Hb）	试验组				
	对照组				
红细胞总数（RBC）	试验组				
	对照组				
血小板（PLT）	试验组				
	对照组				

表9.8.15 血生化-12 个月±7 天谷丙转氨酶前后交叉表

表9.8.16 血生化-12 个月±7 天肌酐前后交叉表

表9.8.17 血生化-12 个月±7 天尿素氮前后交叉表

表9.8.18 血生化-12 个月±7 天总胆固醇前后交叉表

表9.8.19 血生化-12 个月±7 天甘油三酯前后交叉表

表9.8.20 血生化-12 个月±7 天钾前后交叉表

表9.8.21 血生化-12 个月±7 天钠前后交叉表

表9.8.22 血生化-12 个月±7 天氯前后交叉表

表9.8.23 血生化-12 个月±7 天空腹血糖前后交叉表

表9.8.24 血生化-12 个月±7 天汇总表

指标	组别	用药前正常<br>用药后正常	用药前正常<br>用药后异常	用药前正常<br>用药后正常	用药前正常<br>用药后异常
谷丙转氨酶（ALT/GPT）	试验组				
	对照组				
肌酐（Cr）	试验组				
	对照组				
尿素氮（BUN）	试验组				
	对照组				
总胆固醇（TC）	试验组				
	对照组				
甘油三酯（TG）	试验组				
	对照组				
钾（K）	试验组				
	对照组				
钠（Na）	试验组				
	对照组				
氯（Cl）	试验组				
	对照组				
空腹血糖（GLU）	试验组				
	对照组				

表9.8.25 尿常规-12 个月±7 天尿蛋白前后交叉表

表9.8.26 尿常规-12 个月±7 天尿白细胞前后交叉表

表9.8.27 尿常规-12 个月±7 天尿红细胞前后交叉表

表9.8.28 尿常规-12 个月±7 天汇总表

指标	组别	用药前正常		用药前正常	
		用药后正常	用药后异常	用药后正常	用药后异常
尿蛋白（PRO）	试验组				
	对照组				
尿白细胞（WBC）	试验组				
	对照组				
尿红细胞（RBC）	试验组				
	对照组				

表9.8.29 体格检查-12 个月±7 天一般情况前后交叉表

表9.8.30 体格检查-12 个月±7 天神经系统前后交叉表

表9.8.31 体格检查-12 个月±7 天皮肤、浅表淋巴结前后交叉表

表9.8.32 体格检查-12 个月±7 天头颈部前后交叉表

表9.8.33 体格检查-12 个月±7 天心脏前后交叉表

表9.8.34 体格检查-12 个月±7 天肺部前后交叉表

表9.8.35 体格检查-12 个月±7 天腹部前后交叉表

表9.8.36 体格检查-12 个月±7 天泌尿生殖系统前后交叉表

表9.8.37 体格检查-12 个月±7 天脊柱四肢前后交叉表

表9.8.38 体格检查-12 个月±7 天其他前后交叉表

表9.8.39 体格检查-12 个月±7 天汇总表

		用药前正常		用药前正常	
指标	组别	用药后正常	用药后异常	用药后正常	用药后异常
一般情况	试验组				
	对照组				
神经系统	试验组				
	对照组				
皮肤、浅表淋巴结	试验组				
	对照组				
头颈部	试验组				
	对照组				
心脏	试验组				
	对照组				
肺部	试验组				
	对照组				
腹部	试验组				
	对照组				
泌尿生殖系统	试验组				
	对照组				
脊柱四肢	试验组				
	对照组				
其他	试验组				
	对照组				

表9.8.40 心电图-12 个月±7 天前后交叉表

表9.8.41 心电图-12 个月±7 天汇总表

		用药前正常		用药前正常	
指标	组别	用药后正常	用药后异常	用药后正常	用药后异常
心电图临床意义	试验组				
	对照组				

9.9. 清单

附表 1-脱落剔除清单（所有随机化人群）

中心	随机号	组别	是否完成试验	脱落日期	脱落原因	详述	状态	脱落或方案偏离分类	脱落或方案偏离具体原因	FAS	PPS	SS

附表 2-人口资料学清单（FAS）

中心	随机号	组别	出生日期	年龄(岁)	性别	婚姻	民族	其他	身高(cm)	体重(kg)	BMI(kg/m^2)	国籍	其他

附表 3-既往合并用药清单

中心	随机号	组别	有无既往/合并用药	药物名称（通用名）	用药原因	AE 编号	病史编号	剂量用法	开始日期	结束日期	末次随访是否持续	ATC1 术语	ATC2 术语

附表 4-用药依从性清单（研究药物发放使用情况）(FAS)

中心	随机号	组别	发药日期	最后访视日期	服药天数	应服药量（粒）	实际服药量（粒）	依从性(%)	FAS	PPS

附表 5-主要疗效清单

中心	随机号	组别	随机日期	复合终点事件日期	复合终点事件删失	时间（天）	心血管死亡发生时间	心血管死亡删失	时间（天）	心衰恶化再住院时间	心衰恶化再住院删失	时间（天）	FAS	PPS	SS

附表 6-次要指标疗效清单

中心	随机号	组别	随机日期	全因死亡日期	删失 1	时间(天)	复合终点事件（心衰恶化放弃治疗、心脏骤停后复苏成功、恶性心律失常、非致死性卒中）日期	删失 2	时间(天)	心衰恶化放弃治疗日期	删失 3	时间(天)	FAS	PPS

附表 6-1-次要指标疗效清单 -续表

中心	随机号	组别	随机日期	心脏骤停后复苏成功日期	删失 4	时间(天)	恶性心律失常日期	删失 5	时间(天)	非致死性卒中日期	删失 6	时间(天)	FAS	PPS

附表 7-血清 NT-proBNP

中心	随机号	组别	基线血清 NT-proBNP(pg/ml)	1 月血清 NT-proBNP(pg/ml)	差值(基线-1 个月)	下降率(%)	下降率≥30%	3 月血清 NT-proBNP(pg/ml)	差值(基线-3 个月)	下降率(%)	下降率≥30%	FAS	PPS

附表 8-不良事件清单

中心	随机号	组别	有无AE	AE 名称	发生日期	严重程度分级	是否SAE	死亡	危及生命	需要住院治疗或原来住院时间延长	致永久性或显著性残障/残疾或无行为能力	先天性畸形或出生缺陷	重要医学事件	对试验药物采取的措施	是否治疗	是否因此不良事件退出试验	转归	与研究药物的关系	不良事件详情描述	终止/转归日期	版本号	SOC	PT

附表 9-严重不良事件清单

中心	随机号	组别	有无AE	AE 名称	发生日期	严重程度分级	是否SAE	死亡	危及生命	需要住院治疗或原来住院时间延长	致永久性或显著性残障/残疾或无行为能力	先天性畸形或出生缺陷	重要医学事件	对试验药物采取的措施	是否治疗	是否因此不良事件退出试验	转归	与研究药物的关系	不良事件详情描述	终止/转归日期	版本号	SOC	PT

附表 10-血常规前正常后异常清单（SS）

中心	随机号	指标	分组	治疗前	治疗前临床意义	治疗后	治疗后临床意义

附表 11-血常规前异常后异常清单（SS）

同上

附表 12-血生化前正常后异常清单（SS）

同上

附表 13-血生化前异常后异常清单（SS）

同上

附表 14-尿常规前正常后异常清单（SS）

同上

附表 15-尿常规前异常后异常清单（SS）

同上

附表 16-体格检查前正常后异常清单（SS）

同上

附表 17-体格检查前异常后异常清单（SS）

同上

附表 18-心电图前正常后异常清单（SS）

同上

附表 19-心电图前异常后异常清单（SS）

同上

附表 20- 试验完成情况清单（所有随机化人群）

中心	随机号	组别	完成试验	脱落日期	脱落原因	详细记录	是否破盲	破盲原因	脱落日期
								.	
								.	
								.	

附表 21-破盲清单（所有随机化人群）

中心	随机号	组别	是否完成试验	随机日期	是否破盲	破盲日期	破盲原因	是否死亡	死亡日期

QILI QIANGXIN CAPSULES
0.3 G/CAPSULE

CMC INFORMATION

Shijiazhuang Yiling Pharmaceutical Co., Ltd.

December, 2022

中国河北省石家庄市高新技术开发区天山大街 238 号 PC: 050035
No. 238 Tianshan Street, High-Tech Area, Shijiazhuang Hebei Province, China.
TEL: 0086 311 85901719 FAX: 0086 311 85901719 http://www.yiling.cn

Table of Contents

1 Drug Substance

1.1 Nomenclature

1.2General properties

1.3 Manufacturers

1.4 Description of Manufacturing Process and Process Controls

1.5 Specification of Drug Substance

1.6 Batch Analysis

1.7 Analytical Procedures

1.8 Reference Standards or Materials

1.9 Storage Condition

2. Drug Product

2.1 Description and Composition of the Product

2.2 Formulation Development

2.3 Manufacturing Process

2.4 Specifications

2.5 Batch Analysis

2.6 Analytical Procedures

2.7 Reference Standards or Materials

2.8 Stability Summary and Conclusion

2.9 Container Closure System

2.10 Package in China

Appendix

QILI QIANGXIN CAPSULES
0.3 G/CAPSULE

CMC INFORMATION

1. DRUG SUBSTANCE

Shijiazhuang Yiling Pharmaceutical Co., Ltd.

December, 2022

1.1 Nomenclature

No.	Ingredients	Scientific Name	English Name	Plant sources	Used part of Plant
1	Astragali Radix	*Astragalus membranaceus* (Fisch.) Bge. var. *mongholicus* (Bge.) Hsiao or *Astragalus membranaceus* (Fisch.) Bge.	Milkvetch Root	*Astragalus membranaceus* (Fisch.) Bge. var. *mongholicus* (Bge.) Hsiao or *Astragalus membranaceus* (Fisch.) Bge.	Root
2	Ginseng Radix et Rhizoma	*Panax ginseng* C. A.Mey.	Ginseng	*Panax ginseng* C. A.Mey.	Root
3	Aconiti Lateralis Radix Praeparata (heishunpian)	*Aconitum carmichaeli* Debx.	Prepared Common Monkshood Daughter Root	*Aconitum carmichaeli* Debx.	Root
4	Salviae Miltiorrhizae Radix et Rhizoma	*Salvia miltiorrhiza* Bge.	Danshen Root	*Salvia miltiorrhiza* Bge.	Root and rhizome
5	Descurainiae Semen	*Descurainia Sophia* (L.) Webb ex Prantl	Pepperweed Seed	*Descurainia Sophia* (L.) Webb ex Prantl	Ripe seed
6	Alismatis Rhizoma	*Alisma orientale* (Sam.) Juzep.	Oriental Waterplantian Rhizome	*Alisma orientale* (Sam.) Juzep.	Tuber
7	Polygonati Odorati Rhizoma	*Polygonatum odoratum* (Mill.) Druce	Fragrant Solomonseal Rhizome	*Polygonatum odoratum* (Mill.) Druce	Rhizome
8	Cinnamomi Ramulus	*Cinnamomum cassia* Presl	Cassia Twig	*Cinnamomum cassia*	Young branch

				Presl	
9	Carthami Flos	*Carthamus tinctorius* L.	Safflower	*Carthamus tinctorius* L.	Flower
10	Periplocae Cortex	*Periploca sepium* Bge.	Chinese Silkvine Root-bark	*Periploca sepium* Bge.	Root bark
11	Citri Reticulatae Pericarpium	*Citrus reticulata* Blanco	Dried Tangerine Peel	*Citrus reticulata* Blanco	Pericarp

1.2 General Properties

Qili Qiangxin Capsules is invented by Professor Wu Yiling, from Hebei Yiling Medicine Research Institute. He uses Vein sickness theory to treat chronic congestive heart failure. According to years of clinical experiences, he invented the pure Chinese medicine compound prescription. It is consist of Astragali Radix, Ginseng Radix et Rhizoma, Aconiti Lateralis Radix Praeparata, Salviae Miltiorrhizae Radix et Rhizoma, Descurainiae Semen, Alismatis Rhizoma, Polygonati Odorati Rhizoma, Cinnamomi Ramulus , Carthami Flos, Periplocae Cortex and Citri Reticulatae Pericarpium. It is beneficial to *Yang qi*, the blood circulation. It is advantageous in the detumescence. It is suitable for mild and moderate chronic congestive heart failure (lack of Yang Qi, blood stasis and water stop syndrome). All the botanical raw materials are listed in the Chinese Pharmacopoeia (2020 edition).

中国河北省石家庄市高新技术开发区天山大街 238 号 PC：050035
No.238 Tianshan Street, High-Tech Area, Shijiazhuang Hebei Province, China.
TEL: 0086 311 85901719 FAX: 0086 311 85901719 http://www.yiling.cn

**Ingredient （Latin Name）**	**Astragali Radix**
**Original plants （Botanical Name）**	*Astragalus membranaceus* (Fisch.) Bge. var. *mongholicus* (Bge.) Hsiao or *Astragalus membranaceus* (Fisch.) Bge.
**Part used**	Root
**Description**	Cylindrical, some branched, upper part relatively thick, 30-90 cm long, 1-3.5 cm in diameter.Externally pale brownish-yellow or pale brown, with irregular, longitudinal wrinkles or furrows. Texture hard and tenacious, uneasily broken, fracture highly fibrous and starchy, bark yellowish-white, wood pale yellow, with radiate striations and fissures, the centre part of old root occasionally rotten-wood-shaped, blackish-brown or hollowed. Odour, weak; taste, slightly sweet and slightly bean-like on chewing.
**Plant**	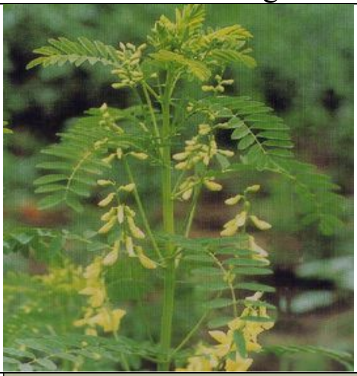
**Slices used**	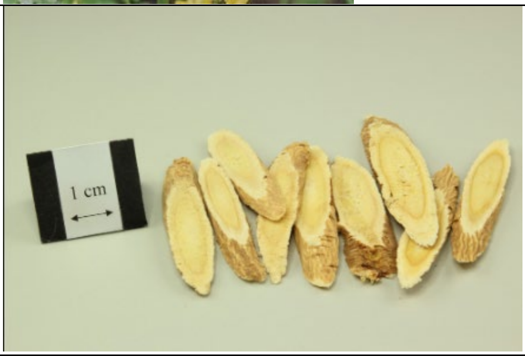

**Ingredient (Latin Name)**	**Ginseng Radix et Rhizoma**
**Original plants (Botanical Name)**	*Panax ginseng* C.A Mey.
**Part used**	Root
**Description**	Main roots fusiform or cylindrical, 3-15 cm long, 1-2 cm in diameter; externally greyish-yellow, the upper part or entire root exhibiting sparse, shallow, interrupted and coarse transverse-striations and distinct longitudinal wrinkles; the lower part bearing 2-3 branch roots and numerous slender rootlets with inconspicuous minute tubercles. Rhizomes (Lutou) 1-4 cm long, 0.3-1.5 cm in diameter, mostly constricted and curved, bearing adventitious roots (Ding) and showing sparse depressed- circular stem scars (Luwan). Texture relatively hard, fracture yellowish-white, starchy, cambium ring brownish- yellow, bark exhibiting yellow-brown dotted resin canals and radial clefts. Odour, characteristic; taste, slightly bitter and sweet.<br>Alternatively, main roots as long as or shorter than thizome, cylindrical, thomboid or V-shaped, 1-6 cm long; externally greyish-yellow, longitudinally wrinkled, the upper or middle-lower part with annulations, branch roots mostly 2-3, rootlets less and slender, orderly arranged and showing some distinct warts. Rhizomes slender, a few stout, the upper part exhibiting sparse or dense deep depressed stem scars7 adventitious roots relatively thin, mostly reclinate.
**Plant**	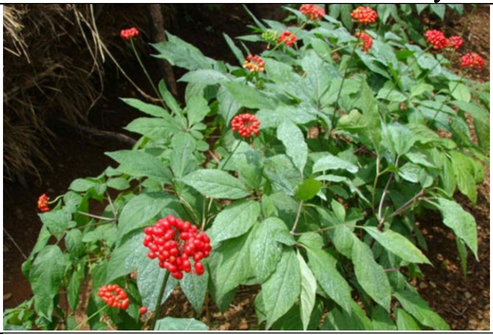
**Slices used**	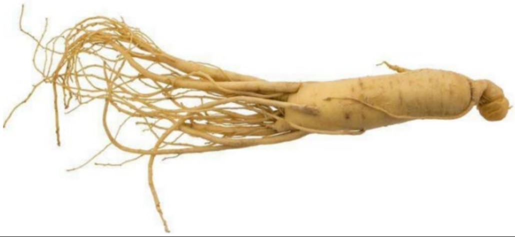 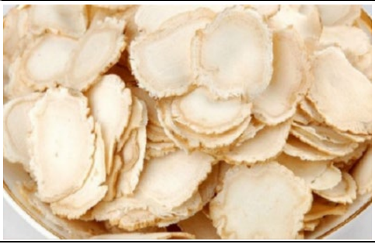

石家庄以岭药业股份有限公司
YILING SHIJIAZHUANGYILING PHARMACEUTICAL CO.,LTD
中国河北省石家庄市高新技术开发区天山大街 238 号 PC：050035
No.238 Tianshan Street, High-Tech Area, Shijiazhuang Hebei Province, China.
TEL: 0086 311 85901719 FAX: 0086 311 85901719 http://www.yiling.cn

**Ingredient （Latin Name）**	**Aconiti Lateralis Radix Praeparata (heishunpian)**
**Original plants （Botanical Name）**	*Aconitum carmichaeli* Debx.
**Part used**	Daughter root
**Description**	Heishunpian (black slice) Longitudinal slices, the upper portion wide and the lower portion narrow, 1.7-5 cm long, 0.9-3 cm wide, 0.2-0.5 cm thick. The outer bark blackish-brown, cut surface dark yellow, oily and lustrous, translucent and showing longitudinal vascular bundles. Texture hard and fragile.Fracture horny. Odour, slight; taste, weak.
**Plant**	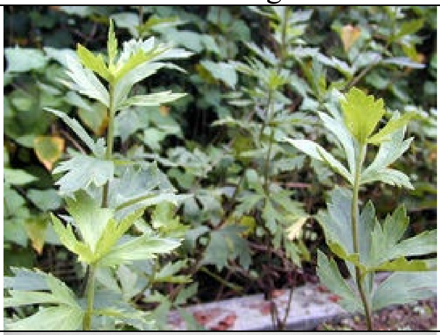
**Slices used**	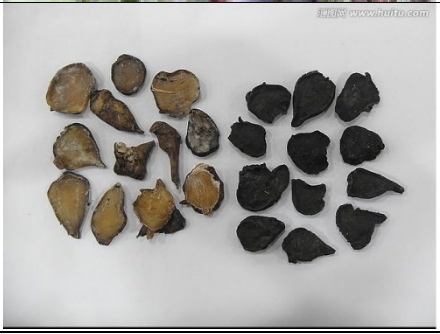

**Ingredient (Latin Name)**	**Salviae Miltiorrhizae Radix et Rhizoma**
**Original plants (Botanical Name)**	*Salvia miltiorrhiza* Bge.
**Part used**	Root and rhizome
**Description**	Rhizomes short and stout, sometimes with remains of a stem at the apex. Several roots, long cylindrical, slightly curved, some branched and with rootlets, 10-20 cm long, 0.3-1 cm in diameter. Externally brownish-red or dark brownish-red.rough, longitudinally wrinkled. The bark of old roots loose.mostly purplish-brown, usually scaling off. Texture hard and fragile, fracture loose with clefts or slightly even and dense, with brownish-red bark and greyish-yellow or purplish-brown wood.showing bundles of vessels, yellowish-white, arranged radially.Odour, slight; taste, slightly bitter and astringent.Cultivars relatively stout, 0.5-1.5 cm in diameter. Externally reddish-brown, longitudinally wrinkled, the bark closely adhering to wood and uneasy to be scaled off. Texture compact, fracture relatively even, slight horny.
**Plant**	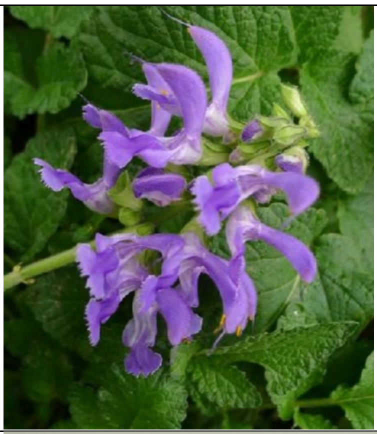
**Slices used**	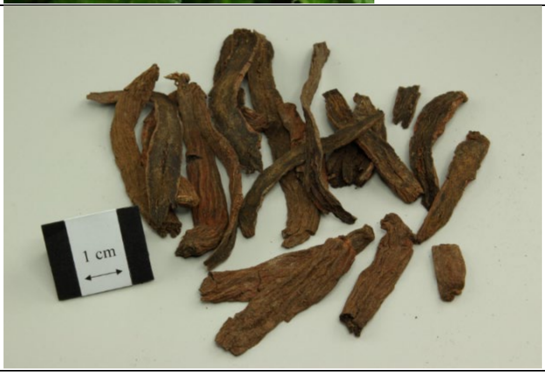

**Ingredient (Latin Name)**	**Descurainiae Semen**
**Original plants (Botanical Name)**	*Descurainia Sophia* (L.) Webb ex Prantl
**Part used**	Ripe seed
**Description**	Flattened-ovoid,0.8-1.2 mm long, about 0.5 mm wide. Externally brown or reddish-brown, somewhat lustrous, with 2 longitudinal furrows, one of them relatively distinct. One end obtuse, the other flat or slightly concave, hilum off-white, situated at the concave end. Odour, slight; taste, slightly pungent and bitter, relatively viscous.
**Plant**	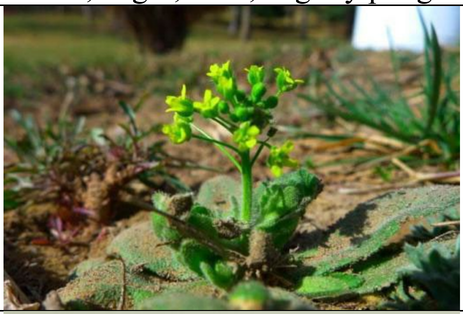
**Slices used**	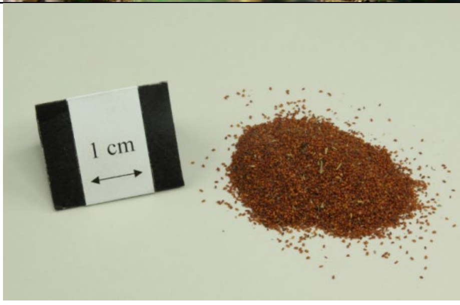

石家庄以岭药业股份有限公司
YILING SHIJIAZHUANGYILING PHARMACEUTICAL CO.,LTD
中国河北省石家庄市高新技术开发区天山大街 238 号 PC: 050035
No.238 Tianshan Street, High-Tech Area, Shijiazhuang Hebei Province, China.
TEL: 0086 311 85901719 FAX: 0086 311 85901719 http://www.yiling.cn

**Ingredient (Latin Name)**	**Alismatis Rhizoma**
**Original plants (Botanical Name)**	*Alisma orientale* (Sam.) Juzep.
**Part used**	Tuber
**Description**	Subspherical, elliptical or ovate, 2-7 cm long, 2-6 cm in diameter. Externally light yellow to yellowish-brown, with irregular transverse-annular shallow furrows and numerous small raised fibrous root scars, occasionally tuberculate bud scars attached to the base. Texture compact, fracture yellowish-white, starchy, with numerous small pores. Odour, slight; taste, slightly bitter.
**Plant**	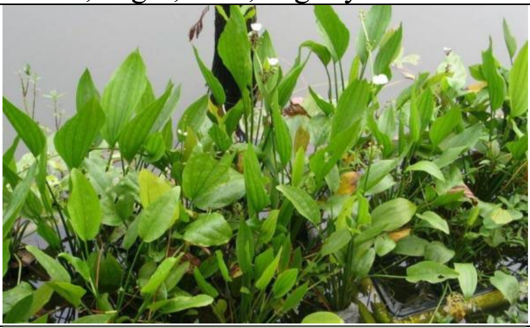
**Slices used**	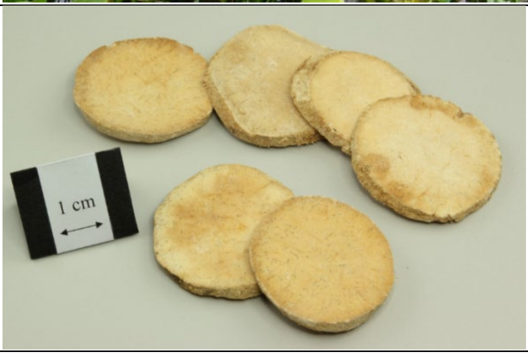

石家庄以岭药业股份有限公司
SHIJIAZHUANGYILING PHARMACEUTICAL CO.,LTD
中国河北省石家庄市高新技术开发区天山大街 238 号 PC：050035
No.238 Tianshan Street, High-Tech Area, Shijiazhuang Hebei Province, China.
TEL: 0086 311 85901719 FAX: 0086 311 85901719 http://www.yiling.cn

**Ingredient （Latin Name）**	**Polygonati Odorati Rhizoma**
**Original plants （Botanical Name）**	*Polygonatum odoratum* (Mill.) Druce
**Part used**	Rhizome
**Description**	Long cylindrical, slightly flattened, occasionally branched, 4-18 cm long, 0.3-1.6 cm in diameter. Externally yellowish-white or pale yellowish-brown, translucent, with longitudinal wrinkles and slightly raised annulations, exhibiting white rounded-dotted fibrous root scars and a disk-like stem scar. Texture hard and fragile or slightly soft, easily broken, fracture horny or granular.Odour slight; taste, sweetish and viscous on chewing.
**Plant**	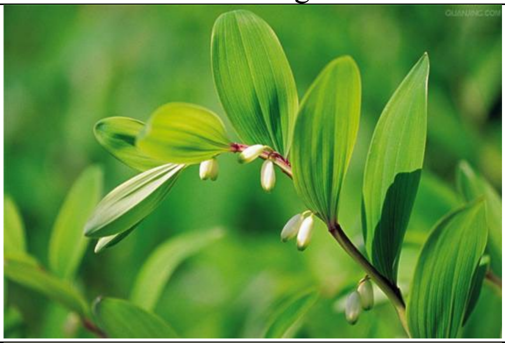
**Slices used**	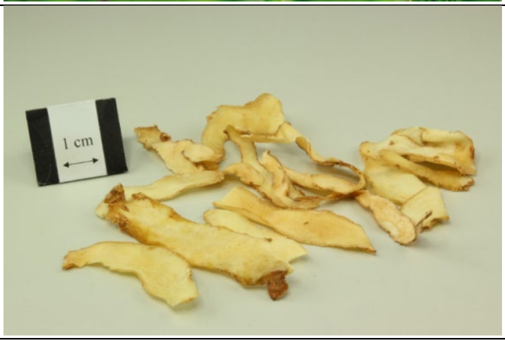

石家庄以岭药业股份有限公司
YILING SHIJIAZHUANGYILING PHARMACEUTICAL CO.,LTD
中国河北省石家庄市高新技术开发区天山大街238号 PC：050035
No.238 Tianshan Street, High-Tech Area, Shijiazhuang Hebei Province, China.
TEL: 0086 311 85901719 FAX: 0086 311 85901719 http://www.yiling.cn

**Ingredient (Latin Name)**	**Cinnamomi Ramulus**
**Original plants (Botanical Name)**	*Cinnamomum cassia* Presl
**Part used**	Young branch
**Description**	Long cylindrical, much-branched, 30-75 cm long, thick end 0.3-1 cm in diameter. Externally brown to reddish-brown, with longitudinal ridges, fine wrinkles, dotted leaf-scars, branch-scars and bud-scars, lenticels dotted. Texture hard and fragile, easily broken. Slices 2-4 mm thick, cut surface showing reddish-brown in bark, yellowish-white to pale yellowish-brown in wood, pith subsquare. Odour, characteristically aromatic; taste, sweet and slightly pungent, relatively strong for bark.
**Plant**	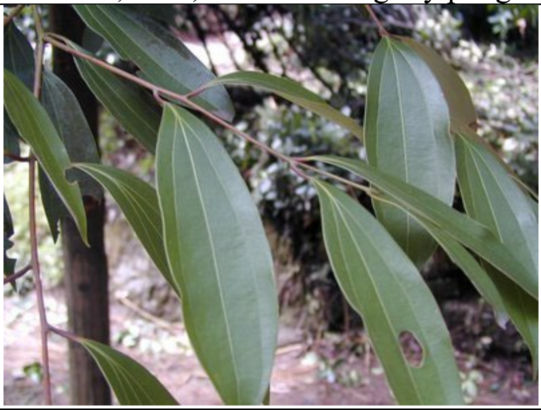
**Slices used**	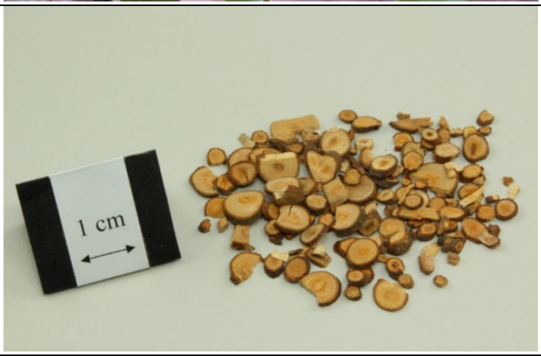

石家庄以岭药业股份有限公司
SHIJIAZHUANGYILING PHARMACEUTICAL CO.,LTD
中国河北省石家庄市高新技术开发区天山大街 238 号 PC：050035
No.238 Tianshan Street, High-Tech Area, Shijiazhuang Hebei Province, China.
TEL: 0086 311 85901719 FAX: 0086 311 85901719 http://www.yiling.cn

**Ingredient (Latin Name)**	**Carthami Flos**
**Original plants (Botanical Name)**	*Carthamus tinctorius* L.
**Part used**	Flower
**Description**	The drug consisting of tubular flowers without ovaries, 1-2 cm long. Externally reddish-yellow or red.Corolla tubes slender, 5-lobed at the apex, the lobes narrowly belt-shaped, 5-8 mm long. Stamens 5, anthers aggregated to a tube, yellowish-white. Stigma long cylindrical, slightly 2-cleft. Texture pliable. Odour, slightly aromatic; taste, slightly bitter.
**Plant**	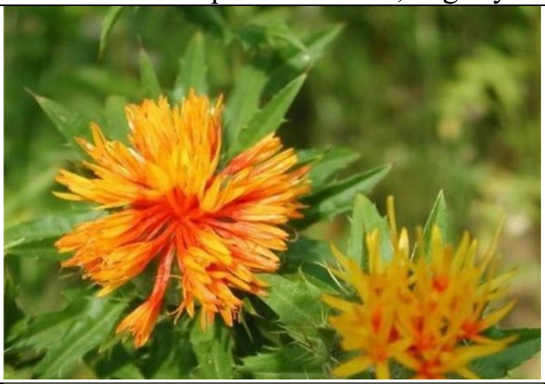
**Slices used**	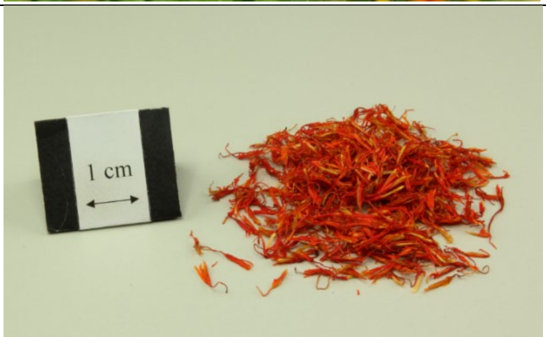

石家庄以岭药业股份有限公司
YILING SHIJIAZHUANGYILING PHARMACEUTICAL CO.,LTD
中国河北省石家庄市高新技术开发区天山大街238号 PC：050035
No.238 Tianshan Street, High-Tech Area, Shijiazhuang Hebei Province, China.
TEL: 0086 311 85901719 FAX: 0086 311 85901719 http://www.yiling.cn

**Ingredient (Latin Name)**	**Periplocae Cortex**
**Original plants (Botanical Name)**	*Periploca sepium* Bge.
**Part used**	Root bark
**Description**	Quilled or channelled, a few pieced irregularly, 3-10 cm long, 1-2 cm in diameter, 2-4 mm thick Outer surface greyish-brown or yellowish-brown, cork soft and loose, often scaly, easily exfoliated; inner surface pale yellow or pale yellowish-brown, relatively smooth, with fine longitudinal striations. Texture light and fragile, easily broken, fracture uneven, yellowish-white. Odour, char acteristic and aromatic; taste, bitter.
**Plant**	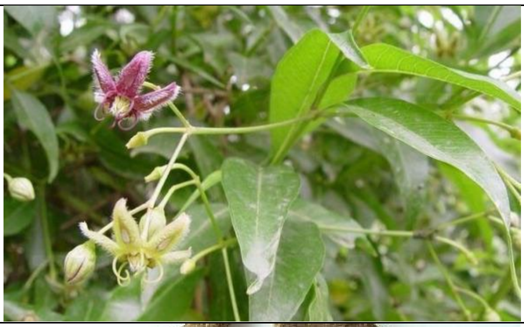
**Slices used**	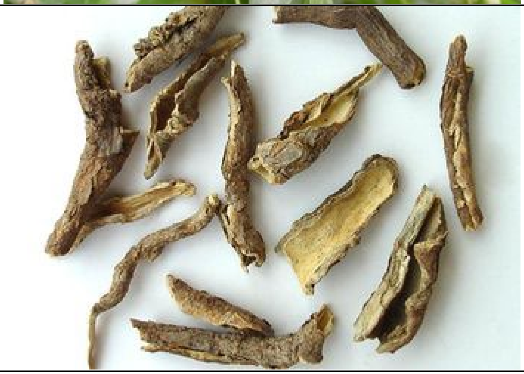

石家庄以岭药业股份有限公司
YILING SHIJIAZHUANGYILING PHARMACEUTICAL CO.,LTD
中国河北省石家庄市高新技术开发区天山大街 238 号 PC：050035
No.238 Tianshan Street, High-Tech Area, Shijiazhuang Hebei Province, China.
TEL: 0086 311 85901719 FAX: 0086 311 85901719 http://www.yiling.cn

**Ingredient （Latin Name）**	**Citri Reticulatae Pericarpium**
**Original plants （Botanical Name）**	*Citrus reticulata* Blanco
**Part used**	Pericarp
**Description**	Often peeled in several lobes connecting at the base, or in irregular slices, 1-4 mm thick. Outer surface orange-red to reddish-brown, with fine wrinkles and concave dotted cavity, inner surface pale yellowish-white, rough, bearing yellowish-white or yellowish-brown vein-like vascular bundles. Texture slightly hard and fragile. Odour, aromatic; taste, pungent and bitter.
**Plant**	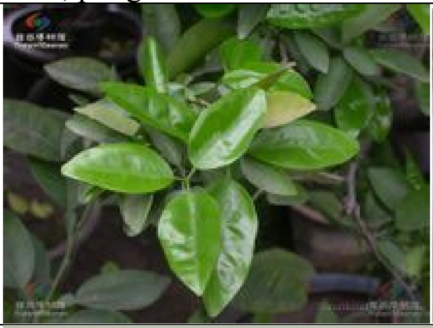 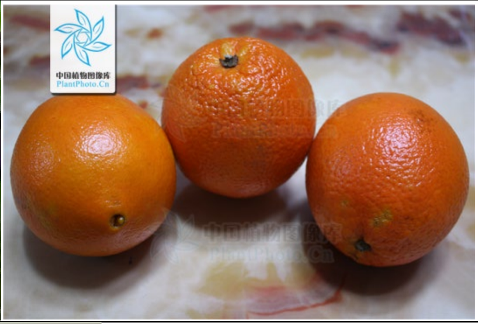
**Slices used**	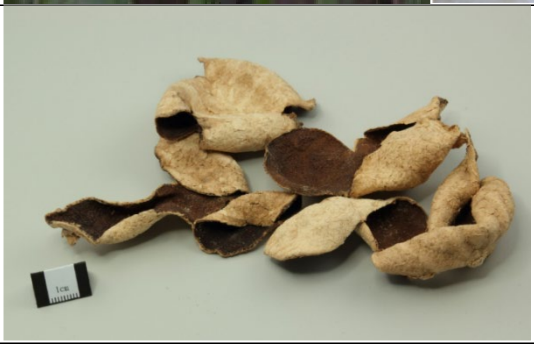

1.3 Manufactures

Active ingredient	Supplier
Astragali Radix	Shijiazhuang Yiling Hebal Pieces Co., Ltd.
Ginseng Radix et Rhizoma	Tonghua Bozhen Ginseng Industry Co., Ltd.
Aconiti Lateralis Radix Praeparata (heishunpian)	Sichuan New Lotus Herbal Pieces Co., Ltd.
Salviae Miltiorrhizae Radix et Rhizoma	Shandong Ruixiang Chinese Medicine Co., Ltd.
	Yiyuan Yikangyuan Chinese Medicine Co. Ltd.
Descurainiae Semen	Weishi Zhongxin Chinese Medicine Co., Ltd.
Alismatis Rhizoma	Sichuan Santai Hongcheng Jiaxin Medicine Planting Co., Ltd.
Polygonati Odorati Rhizoma	Shijiazhuang Yiling Hebal Pieces Co., Ltd.
Cinnamomi Ramulus	Bozhou Ronggui Pharmaceutical Co. Ltd.
Carthami Flos	Xinjiang Safflower Development Limited Company of Yabao Pharmaceutical Group
Periplocae Cortex	Anguo Senkang Chinese Medicine Co. Ltd.
	Shexian Mingwen Agricultural Development Co., Ltd.
Citri Reticulatae Pericarpium	Shijiazhuang Yiling Hebal Pieces Co., Ltd.

中国河北省石家庄市高新技术开发区天山大街 238 号 PC：050035
No.238 Tianshan Street, High-Tech Area, Shijiazhuang Hebei Province, China.
TEL: 0086 311 85901719 FAX: 0086 311 85901719 http://www.yiling.cn

1.4 Description of manufacturing process

No.	Ingredients	Harvest	Processing
1.	Astragali Radix	The drug is collected in spring or autumn, removed from rootlet and root stock, dried in the sun.	Eliminate foreign matter, grade according to size, wash clean, soften thoroughly, cut into thick slices and dry.
2.	Ginseng Radix et Rhizoma	The drug is collected in autumn and washed clean.	Soften thoroughly, cut into thin slices, and dry, or pulverize or break to pieces before use.
3.	Aconiti Lateralis Radix Praeparata (heishunpian)	The drug is collected in late June to early August, removed from the parent root, rootlet and soil.	Grade Nifuzi according to size, wash clean and soak in mother liquor of mineral salt preparation for several days. Boil in the infusion thoroughly. Take out, rinse in water, cut longitudinally into slices about 0. 5 cm in thickness. Soak and rinse in water once again. Stain the slices dark brown and steam them until the slices turn to be oily and lustrous. Bake the slices to half-dryness, and then sun-dry or bake to complete dryness.
4.	Salviae Miltiorrhizae Radix et Rhizoma	The drug is collected in spring or autumn, removed from soil, and dried.	Remove foreign matter and remains of stems, wash clean, soften thoroughly, cut into thick slices, and dry.
5.	Descurainiae Semen	The plant is collected in sunmer when the fruit is ripe, dried in the sun, and the seed is gathered, removed from foreign matter.	Eliminate foreign matter.

中国河北省石家庄市高新技术开发区天山大街 238 号 PC：050035
No.238 Tianshan Street, High-Tech Area, Shijiazhuang Hebei Province, China.
TEL: 0086 311 85901719 FAX: 0086 311 85901719 http://www.yiling.cn

6.	Alismatis Rhizoma	The drug is collected in winter when the stem withered, washed clean, dried, and removed from the fibrous roots and coarse outer tissue.	Processing Alismatis Rhizoma Eliminate foreign matter, soak slightly, soften thoroughly, cut into thick slices, and dry.
7.	Polygonati Odorati Rhizoma	The drug is collected in autumn, removed from the fibrous root, washed clean, dried in the sun to soften, rubbed repeatly and dried in the air until the drug is devoid of hard core, and then dried in the sun thoroughly. Or rubbed to be translucent after steaming thoroughly, and dried in the sun.	Eliminate foreign matter, wash clean, soften thoroughly, cut into thick slices or sections and dry.
8.	Cinnamomi Ramulus	The drug is collected in spring and summer, removed from leaf, dried in the sun, or dried in the sun after sliced.	Eliminate foreign matter, wash clean, soften thoroughly, cut into thick slices, and dry.
9.	Carthami Flos	The drug is collected in summer when its colour turns from yellow to red, and dried in shade or in the sun.	The drug is collected in summer when its colour turns from yellow to red, and dried in shade or in the sun.
10.	Periplocae Cortex	The root is collected in spring and autumn, the root bark is stripped off, and dried in the sun.	Eliminate foreign matter, wash clean, soften thoroughly, cut into thick slices, and dry in the sun.
11.	Citri Reticulatae Pericarpium	The fruit is collected when the fruit is ripe, the pericarp is peeled off and dried in the sun or at a low temperature.	Eliminated foreign matter, spray with water, soften thoroughly, cut into slivers, dry in the shade.

中国河北省石家庄市高新技术开发区天山大街 238 号 PC：050035
NO. 238, Tianshan Avenue, Hi-Tech Development Zone, Shijiazhuang, Hebei, China
TEL: 0086 311 85901719 FAX: 0086 311 85901719 http://www.yiling.cn

1.5 Specification of Drug Substance

No.	Chinese Name	Latin Name	Reference Standard	Appendix
1	黄芪	Astragali Radix	Chinese Pharmacopoeia (2020), Volume I	Appendix 1
2	人参	Ginseng Radix et Rhizoma	Chinese Pharmacopoeia (2020), Volume I	Appendix 2
3	附子	Aconiti Lateralis Radix Praeparata (heishunpian)	Chinese Pharmacopoeia (2020), Volume I	Appendix 3
4	丹参	Salviae Miltiorrhizae Radix et Rhizoma	Chinese Pharmacopoeia (2020), Volume I	Appendix 4
5	葶苈子	Descurainiae Semen	Chinese Pharmacopoeia (2020), Volume I	Appendix 5
6	泽泻	Alismatis Rhizoma	Chinese Pharmacopoeia (2020), Volume I	Appendix 6
7	玉竹	Polygonati Odorati Rhizoma	Chinese Pharmacopoeia (2020), Volume I	Appendix 7
8	桂枝	Cinnamomi Ramulus	Chinese Pharmacopoeia (2020), Volume I	Appendix 8
9	红花	Carthami Flos	Chinese Pharmacopoeia (2020), Volume I	Appendix 9
10	香加皮	Periplocae Cortex	Chinese Pharmacopoeia (2020), Volume I	Appendix 10
11	陈皮	Citri Reticulatae Pericarpium	Chinese Pharmacopoeia (2020), Volume I	Appendix 11

1.5.1 Astragali Radix

**Inspection Items**	**Standard Criteria**
**Description**	Should correspond to the description of Astragali Radix.
**Identification**	
(1) Microscopic Character	Should correspond to the Microscopic Character of Astragali Radix.
(2) (3) Thin Layer Chromatography	Test solution should show corresponding spots of ammonium astragaloside *IV* CRS and Astragali Radix reference drug.
**Other requirements**	
Water	Not more than 10.0%.
Total ash	Not more than 5.0%.
Lead	Not more than 5 mg/kg
Copper	Not more than 20 mg/kg
Cadmium	Not more than 0.3 mg/kg
Arsenic	Not more than 2 mg/kg
Mercury	Not more than 0.2 mg/kg
BHC	Not more than 0.2 mg/kg
DDT	Not more than 0.2 mg/kg
PCNB	Not more than 0.1 mg/kg
Residual Sulfur Dioxide	Not more than 150 mg/kg
**Extractives**	Not less than 17.0%
**Assay**	
Astragaloside *IV*	It contains not less than 0.060% of astragaloside *IV* ($C_{41}H_{68}O_{14}$), calculated with reference to the dried drug
Calycosin-7-O-β-D-glycoside	It contains not less than 0.020% of Calycosin-7-O-β-D-glycoside ($C_{22}H_{22}O_{10}$), calculated with reference to the dried drug

中国河北省石家庄市高新技术开发区天山大街 238 号 PC：050035
NO. 238, Tianshan Avenue, Hi-Tech Development Zone, Shijiazhuang, Hebei, China
TEL: 0086 311 85901719 FAX: 0086 311 85901719 http://www.yiling.cn

1.5.2 Ginseng Radix et Rhizoma

**Inspection Items**	**Standard Criteria**
**Description**	Should correspond to the description of Ginseng Radix et Rhizoma
**Identification**	
(1) Microscopic Character	Should correspond to the microscopic character of Ginseng Radix et Rhizoma
(2) (3) Thin Layer Chromatography	Test solution should show corresponding spots of Ginseng Radix et Rhizoma reference drug and ginsenoside Rb_1, Re, Rf and Rg_1.
**Other requirements**	
Water	Not more than 12.0%
Total ash	Not more than 5.0%
Lead	Not more than 5 ppm
Cadmium	Not more than 0.3 ppm
Arsenic	Not more than 2 ppm
Mercury	Not more than 0.2 ppm
Copper	Not more than 20 ppm
BHC	Not more than 0.2 mg/kg
DDT	Not more than 0.2 mg/kg
PCNB	Not more than 0.1 mg/kg
Hexachlorobenzene	Not more than 0.1 mg/kg
Heptachlor	Not more than 0.05 mg/kg
Aldrin	Not more than 0.05 mg/kg
Chlordane	Not more than 0.1 mg/kg
Residual Sulfur Dioxide	Not more than 150 mg/kg
**Assay**	
(1)	It contains not less than 0.30 % of total ginsenoside Rg_1 ($C_{42}H_{72}O_{14}$) and ginsenoside Re ($C_{48}H_{82}O_{18}$), calculated with reference to the dried drug.
(2)	It contains not less than 0.20 % of ginsenoside Rb_1 ($C_{54}H_{92}O_{23}$)

1.5.3 Aconiti Lateralis Radix Praeparata (heishunpian)

**Inspection Items**	**Standard Criteria**
**Description**	Should correspond to the description of Aconiti Lateralis Radix Praeparata.
**Identification**	
Thin Layer Chromatography	It should show corresponding spots of benzoylacointine CRS, benzoylhypaconitine CRS and benzoylmesaconine CRS.
**Other requirements**	
Water	Not more than 15.0 %.
Diester-alkaloids	It contains not more than 0.020% of the total amount of mesaconine($C_{33}H_{45}NO_{11}$), hypaconitine ($C_{33}H_{45}NO_{10}$) and aconitine($C_{34}H_{47}NO_{11}$)
Residual Sulfur Dioxide	Not more than 150 mg/kg
**Assay**	It contains not less than 0.010% of the total amount of benzoylmesaconine ($C_{31}H_{43}NO_{10}$), benzoylacointine ($C_{32}H_{45}NO_{10}$) and benzoylhypaconitine ($C_{31}H_{43}NO_9$).

中国河北省石家庄市高新技术开发区天山大街 238 号 PC：050035
NO. 238, Tianshan Avenue, Hi-Tech Development Zone, Shijiazhuang, Hebei, China
TEL: 0086 311 85901719 FAX: 0086 311 85901719 http://www.yiling.cn

1.5.4 Salviae Miltiorrhizae Radix et Rhizoma

**Inspection Items**	**Standard Criteria**
**Description**	Should correspond to the description of Salviae Miltiorrhizae Radix et Rhizoma.
**Identification**	
(1) Microscopic character	Should correspond to the microscopic character of Salviae Miltiorrhizae Radix et Rhizoma
(2) (3) (4) (5) (6) Thin Layer Chromatography	Test solution should show corresponding spots of Salviae Miltiorrhizae Radix et Rhizoma reference drug, tanshinone IIA CRS, salvianolic acid B CRS.
**Other requirements**	
Water	Not more than 13.0 %
Total ash	Not more than 10.0 %
Acid-insoluble ash	Not more than 3.0%
**Heavy metal**	
Lead	Not more than 5 ppm
Copper	Not more than 20 ppm
Cadmium	Not more than 0.3 ppm
Arsenic	Not more than 2 ppm
Mercury	Not more than 0.2 ppm
Residual Sulfur Dioxide	Not more than 150 mg/kg
**Extractives**	
Water-soluble extractives	Not less than 35.0%
Ethanol-soluble extractives	Not less than 15.0%
**Assay**	
**Tanshinones**	It contains not less than 0.25 % of the total amount of tanshinone II_A ($C_{19}H_{18}O_3$), tanshinone I ($C_{19}H_{20}O_3$) and cryptotanshinone ($C_{18}H_{12}O_3$), calculated with reference to the dried drug.
**Salvianoic acid B**	It contains not less than 3.0 % of salvianoic acid B ($C_{36}H_{30}O_{16}$), calculated with reference to the dried drug.

1.5.5 Descurainiae Semen

**Inspection Items**	**Standard Criteria**
**Description**	Should correspond to the description of Descurainiae Semen or Lepidii Semen
**Identification**	
(1) Physical character	Should correspond to the standard.
(2) Microscopic Character	Should correspond to the Microscopic Character of Descurainiae Semen.
(3) Thin Layer Chromatography	*Nan tinglizi* should show corresponding spots to the quercetin-3-O-β-D-glucose-7-O-β-D-gentiobioside CRS.
**Other requirements**	
Water	Not more than 9.0%.
Total ash	Not more than 8.0%.
Acid-insoluble ash	Not more than 3.0%.
Swelling capacity	Not less than 3 for *Nan Tinglizi*.
Residual Sulfur Dioxide	Not more than 150 mg/kg
**Assay**	
quercetin-3-O-β-D-glucose -7 -O-β-D-gentiobioside	It contains not less than 0.075% ofquercetin-3-O-β-D-glucose-7-O-β-D-gentiobioside ($C_{33}H_{40}O_{22}$), calculated with reference to the dried drug.

中国河北省石家庄市高新技术开发区天山大街 238 号 PC：050035
NO. 238, Tianshan Avenue, Hi-Tech Development Zone, Shijiazhuang, Hebei, China
TEL: 0086 311 85901719 FAX: 0086 311 85901719 http://www.yiling.cn

1.5.6 Alismatis Rhizoma

Inspection Items	Standard Criteria
**Description**	Should correspond to the description of Alismatis Rhizoma.
**Identification**	
(1) Microscopic Character	Should correspond to the Microscopic Character of Alismatis Rhizoma.
(2) Thin Layer Chromatography	Test solution should show corresponding spots to the 23-acetate alisol B CRS.
**Other requirements**	
Water	Not more than 14.0 %.
Total ash	Not more than 5.0%
Residual Sulfur Dioxide	Not more than 150 mg/kg
**Extractives**	Not less than 10.0%
**Assay**	
23-acetate alisol B	It contains not less than 0.050 % of 23-acetate alisol B ($C_{32}H_{50}O_5$), calculated with reference to the dried drug.

中国河北省石家庄市高新技术开发区天山大街 238 号 PC: 050035
NO. 238, Tianshan Avenue, Hi-Tech Development Zone, Shijiazhuang, Hebei, China
TEL: 0086 311 85901719 FAX: 0086 311 85901719 http://www.yiling.cn

1.5.7 Polygonati Odorati Rhizoma

**Inspection Items**	**Standard Criteria**
**Description**	Should correspond to the description of Polygonati Odorati Rhizoma.
**Identification**	
(1) Microscopic Character	Should correspond to the Microscopic Character of Polygonati Odorati Rhizoma.
**Other requirements**	
Water	Not more than 16.0 %.
Total ash	Not more than 3.0%.
Residual Sulfur Dioxide	Not more than 150 mg/kg
**Extractives**	Not less than 50.0%
**Assay**	
Polysaccharide	It contains not less than 6.0 % ofpolysaccharide, calculated as glucose ($C_6H_{12}O_6$) with reference to the dried drug.

1.5.8 Cinnamomi Ramulus

**Inspection Items**	**Standard Criteria**
**Description**	Should correspond to the description of Cinnamomi Ramulus.
**Identification**	
(1) Microscopic Character	Should correspond to the Microscopic Character of Cinnamomi Ramulus.
(2) (3) Thin Layer Chromatography	Test solution should show corresponding spots to the cinnamic aldehyde CRS and Cinnamomi Ramulus reference drug.
**Other requirements**	
Water	Not more than 12.0 %.
Total ash	Not more than 3.0%.
Residual Sulfur Dioxide	Not more than 150 mg/kg
**Extractives**	Not less than 6.0%
**Assay**	
Cinnamaldehyde	It contains not less than 1.0 % of cinnamic aldehyde (C_9H_8O), calculated with reference to the dried drug.

中国河北省石家庄市高新技术开发区天山大街 238 号 PC：050035
NO. 238, Tianshan Avenue, Hi-Tech Development Zone, Shijiazhuang, Hebei, China
TEL: 0086 311 85901719 FAX: 0086 311 85901719 http://www.yiling.cn

1.5.9 Carthami Flos

**Inspection Items**	**Standard Criteria**
**Description**	Should correspond to the description of Carthami Flos.
**Identification**	
(1) Microscopic Character	Should correspond to the Microscopic Character of Carthami Flos.
(2) Thin Layer Chromatography	Test solution should show corresponding spots to the Carthami Flos reference drug.
**Other requirements**	
Foreign matter	Not more than 2%.
Water	Not more than 13.0%.
Total ash	Not more than15.0%.
Acid-insoluble ash	Not more than 5.0%.
Absorbance	Not less than 0.20 at 518nm
Residual Sulfur Dioxide	Not more than 150 mg/kg
**Extractives**	Not less than 30.0%
**Assay**	
Hydroxysafflor yellow A	It contains not less than 1.0 % of Hydroxysafflor yellow A ($C_{27}H_{32}O_{16}$), calculated with reference to the dried drug.
Kaempferol	It contains not less than 0.050 % of Kaempferol ($C_{15}H_{10}O_6$), calculated with reference to the dried drug.

1.5.10 Periplocae Cortex

**Inspection Items**	**Standard Criteria**
**Description**	Should correspond to the description of Periplocae Cortex.
**Identification**	
(1) Microscopic Character	Should correspond to the Microscopic Character of Periplocae Cortex.
(2) Chemical reaction	Should show positive reaction.
(3) UV spectrum	An absorption should exhibit a maximum at 278nm.
(4) Thin Layer Chromatography	Test solution should show corresponding spots to the 4-methoxy salicylic aldehyde CRS.
**Other requirements**	
Water	Not more than 13.0 %.
Total ash	Not more than10.0%.
Acid-insoluble ash	Not more than 4.0%.
**Extractives**	Not less than 20.0%
**Assay**	
4-methoxy salicylic aldehyde	It contains not less than 0.20% of 4-methoxy salicylic aldehyde($C_8H_8O_3$), calculated on the basis dried at 60°C for 4 hours.

中国河北省石家庄市高新技术开发区天山大街 238 号 PC：050035
NO. 238, Tianshan Avenue, Hi-Tech Development Zone, Shijiazhuang, Hebei, China
TEL: 0086 311 85901719 FAX: 0086 311 85901719 http://www.yiling.cn

1.5.11 Citri Reticulatae Pericarpium

**Inspection Items**	**Standard Criteria**
**Description**	Should correspond to the description of Citri Reticulatae Pericarpium.
**Identification**	
(1) Microscopic Character	Should correspond to the Microscopic Character of Citri Reticulatae Pericarpium.
(2) Thin Layer Chromatography	Test solution should show corresponding spots to the hesperidin CRS
**Other requirements**	
Water	Not more than 13.0 %.
Aflatoxin	It contains not more than 5μg of aflatoxin B_1 per 1000g
	Not more than 10μg of the sum of aflatoxin G_2, aflatoxin G_1, aflatoxin B_2 and aflatoxin B_1 per 1000g.
Residual Sulfur Dioxide	Not more than 150 mg/kg
**Assay**	
Hesperidin	It contains not less than 2.5 % of hesperidin($C_{28}H_{34}O_{15}$), calculated with reference to the dried drug.

1.6 Batch Analyses

1.6.1. Astragali Radix

**Items**	**Standard Criteria**	**Batch No. M210226-1**	**Batch No. M220531-1**
**Description**	Should correspond to the description of Astragali Radix.	Corresponding	Corresponding
**Identification**			
(1) Microscopic Character	Should correspond to the Microscopic Character of Astragali Radix.	Corresponding	Corresponding
(2) (3) Thin Layer Chromatography	Test solution should show corresponding spots of ammonium astragaloside *IV* CRS and Astragali Radix reference drug.	Corresponding	Corresponding
**Other requirements**			
Water	Not more than 10.0%.	7.4%	7.0%
Total ash	Not more than 5.0%.	4.1%	4.0%
Lead	Not more than 5 mg/kg	0.2 mg/kg	0.4 mg/kg
Copper	Not more than 20 mg/kg	6.0 mg/kg	6.2 mg/kg
Cadmium	Not more than 1 mg/kg	0.02 mg/kg	0.09 mg/kg
Arsenic	Not more than 2 mg/kg	0.5 mg/kg	0.5 mg/kg
Mercury	Not more than 0.2 mg/kg	Not detected (The detection limit is 0.8μg/kg)	Not detected (The detection limit is 0.8μg/kg)
PCNB	Not more than 0.1 mg/kg	Not detected	Not detected
Residual Sulfur Dioxide	Not more than 150 mg/kg	0 mg/kg	5 mg/kg
**Extractives**	Not less than 17.0%	38.2%	32.6%
**Assay**			
Astragaloside *IV*	It contains not less than 0.080% of astragaloside *IV* ($C_{41}H_{68}O_{14}$), calculated with reference to the dried drug	0.247%	0.418%
Calycosin-7-O-β-D-glycoside	It contains not less than 0.020% of Calycosin-7-O-β-D-glycoside ($C_{22}H_{22}O_{10}$), calculated with reference to the dried drug	0.048%	0.072%
**Conclusion**	The results of the two batch comply with the Chinese Pharmacopoeia (2020), Volume I		

1.6.2. Ginseng Radix et Rhizoma

**Inspection Items**	**Standard Criteria**	**Batch No.** M210312-1	**Batch No.** M220521-3
**Description**	Should correspond to the description of Ginseng Radix et Rhizoma	Corresponding	Corresponding
**Identification**			
(1) Microscopic Character	Should correspond to the microscopic character of Ginseng Radix et Rhizoma	Corresponding	Corresponding
(2) (3) Thin Layer Chromatography	Test solution should show corresponding spots of Ginseng Radix et Rhizoma reference drug and ginsenoside Rb_1, Re, R_f and Rg_1.	Corresponding	Corresponding
**Other requirements**			
Water	Not more than 12.0%	9.8%	9.8%
Total ash<br>Lead<br>Cadmium	Not more than 5.0%<br>Not more than 5 mg/kg<br>Not more than 1 mg/kg	4.2%<br>0.05 mg/kg<br>0.09 mg/kg	4.0%<br>0.05 mg/kg<br>0.2 mg/kg
Arsenic	Not more than 2 mg/kg	0.1 mg/kg	0.2 mg/kg
Mercury	Not more than 0.2 mg/kg	Not detected (detection limit 0.8 µg/kg)	Not detected (detection limit 0.8 µg/kg)
Copper	Not more than 20 mg/kg	9.3 mg/kg	8.5 mg/kg
PCNB<br>BHC<br>Heptachlor<br>Chlordane	Not more than 0.1 mg/kg<br>Not more than 0.1 mg/kg<br>Not more than 0.05 mg/kg<br>Not more than 0.1 mg/kg	0.002 mg/kg<br>Not detected<br>Not detected<br>Not detected	0.02 mg/kg<br>Not detected<br>0.02 mg/kg<br>Not detected
Residual Sulfur Dioxide	Not more than 150 mg/kg	2 mg/kg	0 mg/kg
**Assay**			
(1)	It contains not less than 0.30 % of total ginsenoside Rg_1 ($C_{42}H_{72}O_{14}$) and ginsenoside Re ($C_{48}H_{82}O_{18}$), calculated with reference to the dried drug.	0.51 %	0.58 %
(2)	It contains not less than 0.20 % of ginsenoside Rb_1 ($C_{54}H_{92}O_{23}$)	0.22 %	0.29 %
**Conclusion:**	The results of the two batches comply to Chinese Pharmacopoeia (2020), Volume I		

中国河北省石家庄市高新技术开发区天山大街 238 号 PC: 050035
No.238 Tianshan Street, High-Tech Area, Shijiazhuang Hebei Province, China.
TEL: 0086 311 85901719 FAX: 0086 311 85901719 http://www.yiling.cn

1.6.3. Aconiti Lateralis Radix Praeparata (heishunpian)

**Inspection Items**	**Standard Criteria**	**Batch No. Z210224-1**	**Batch No. Z210310-2**
**Description**	Should correspond to the description of Aconiti Lateralis Radix Praeparata.	Corresponding. It was *Heishunpian (Black slice)*	Corresponding. It was *Heishunpian (Black slice)*
**Identification**			
Thin Layer Chromatography	It should show corresponding spots of benzoylacointine CRS, benzoylhypaconitine CRS and benzoylmesaconine CRS.	Corresponding	Corresponding
**Other requirements**			
Water	Not more than 15.0 %.	11.8 %	12.9 %
Diester-alkaloids	It contains not more than 0.020% of the total amount of mesaconine($C_{33}H_{45}NO_{11}$), hypaconitin ($C_{33}H_{45}NO_{10}$) and aconitine($C_{34}H_{47}NO_{11}$)	0.003%	0.003%
Residual Sulfur Dioxide	Not more than 150 mg/kg	0 mg/kg	0 mg/kg
**Assay**	It contains not less than 0.010% of the total amou of benzoylmesaconine ($C_{31}H_{43}NO_{10}$), benzoylacointine ($C_{32}H_{45}NO_{10}$) and benzoylhypaconitine ($C_{31}H_{43}NO_{9}$).	0.030%	0.010%
**Conclusion**	The results comply with Chinese Pharmacopoeia (2020), Volume I		

1.6.4. Salviae Miltiorrhizae Radix et Rhizoma

**Inspection Items**	**Standard Criteria**	**Batch No. M210224-1**	**Batch No. M220607-1**
**Description**	Should correspond to the description of Salviae Miltiorrhizae Radix et Rhizoma.	Corresponding, Cultivated product.	Corresponding, Cultivated product.
**Identification**			
(1) Microscopic character	Should correspond to the microscopic character of Salviae Miltiorrhizae Radix et Rhizoma	Corresponding	Corresponding
(2) (3) (4) (5) (6) Thin Layer Chromatography	Test solution should show corresponding spots of Salviae Miltiorrhizae Radix et Rhizoma reference drug, tanshinone IIA CRS, salvianolic acid B CRS.	Corresponding spots	Corresponding spots
**Other requirements**			
Water	Not more than 13.0 %	10.7%	7.2%
Total ash	Not more than 10.0 %	7.1%	4.7%
Acid-insoluble ash	Not more than 3.0%	2.0%	1.0%
**Heavy metal**<br>Lead<br>Copper<br>Cadmium<br>Arsenic<br>Mercury	 Not more than 5 mg/kg<br>Not more than 20 mg/kg<br>Not more than 1 mg/kg<br>Not more than 2 mg/kg<br>Not more than 0.2 mg/kg	 2 mg/kg<br>12 mg/kg<br>0.08 mg/kg<br>1 mg/kg<br>Not detected (detection limit 0.8μg/kg)	 0.5 mg/kg<br>9.8 mg/kg<br>0.04 mg/kg<br>0.3 mg/kg<br>Not detected (detection limit 0.8μg/kg)
Residual Sulfur Dioxide	Not more than 150 mg/kg	0 mg/kg	0 mg/kg
**Extractives**			
Water-soluble extractives	Not less than 35.0%	54.4%	64.6%
Ethanol-soluble extractives	Not less than 15.0%	18.2%	19.6%
**Assay**			
**Tanshinones**	It contains not less than 0.25 % of the total amount of tanshinone II_A ($C_{19}H_{18}O_3$), tanshinone I ($C_{19}H_{20}O_3$) and cryptotanshinone ($C_{18}H_{12}O_3$), calculated with reference to the dried drug.	0.54%	0.71%

**Salvianoic acid B**	It contains not less than 3.0 % of salvianoic acid B ($C_{36}H_{30}O_{16}$), calculated with reference to the dried drug.	6.0%	6.8%
**Conclusion:**	The results comply to Chinese Pharmacopoeia (2020), Volume I		

1.6.5. Descurainiae Semen

**Inspection Items**	**Standard Criteria**	**Batch No. M210122-1**	**Batch No. M210308-1**
**Description**	Should correspond to the description of Descurainiae Semen or Lepidii Semen	Corresponding It was Descurainiae Semen (*Nan tinglizi)*	Corresponding It was Descurainiae Semen (*Nan tinglizi)*
**Identification**			
(1) Physical character	Should correspond to the standard.	Corresponding	Corresponding
(2) Microscopic Character	Should correspond to the Microscopic Character of Descurainiae Semen.	Corresponding	Corresponding
(3) Thin Layer Chromatography	*Nan tinglizi* should show corresponding spots to the quercetin-3-O-β-D-glucose -7 -O-β-D-gentiobioside CRS.	Corresponding	Corresponding
**Other requirements**			
Water	Not more than 9.0%.	7.4%	7.7%
Total ash	Not more than 8.0%.	4.7%	4.7%
Acid-insoluble ash	Not more than 3.0%.	1.1%	0.9%
Swelling capacity	Not less than 3 for *Nan Tinglizi*.	5	5
Residual Sulfur Dioxide	Not more than 150 mg/kg	62 mg/kg	36 mg/kg
**Assay**			
quercetin-3-O-β-D-glucose -7 -O-β-D-gentiobioside	It contains not less than 0.075 % ofquercetin-3-O-β-D-glucose -7 -O-β-D-gentiobioside ($C_{33}H_{40}O_{22}$), calculated with reference to the dried drug.	0.097%	0.098%
**Conclusion**	The results comply with Chinese Pharmacopoeia (2020), Volume I		

1.6.6. Alismatis Rhizoma

**Inspection Items**	**Standard Criteria**	**Batch No. Z210315-1**	**Batch No. Z210320-1**
**Description**	Should correspond to the description of Alismatis Rhizoma.	Corresponding	Corresponding
**Identification**			
(1) Microscopic Character	Should correspond to the Microscopic Character of Alismatis Rhizoma.	Corresponding	Corresponding
(2) (3)Thin Layer Chromatography	Test solution should show corresponding spots to the Alismatis Rhizoma reference drug, 23-acetate alisol B CRS, 23-acetate alisol C CRS.	Corresponding	Corresponding
**Other requirements**			
Water	Not more than 14.0 %.	10.2%	12.3%
Total ash	Not more than 5.0%	2.7%	2.6%
Residual Sulfur Dioxide	Not more than 150 mg/kg	0 mg/kg	0 mg/kg
**Extractives**	Not less than 10.0%	15.6%	15.2%
**Assay**			
23-acetate alisol B	It contains not less than 0.10 % of the total of 23-acetate alisol B ($C_{32}H_{50}O_5$) and 23-acetate alisol C ($C_{32}H_{48}O_6$), calculated with reference to the dried drug.	0.34 %	0.22 %
**Conclusion**	The results comply with Chinese Pharmacopoeia (2020), Volume I		

1.6.7. Polygonati Odorati Rhizoma

**Inspection Items**	**Standard Criteria**	**Batch No. M210125-1**	**Batch No. M210325-1**
**Description**	Should correspond to the description of Polygonati Odorati Rhizoma.	Corresponding	Corresponding
**Identification**			
Microscopic Character	Should correspond to the Microscopic Character of Polygonati Odorati Rhizoma.	Corresponding	Corresponding
**Other requirements**			
Water	Not more than 16.0 %.	11.6 %	12.3%
Total ash	Not more than 3.0%.	2.7 %	2.7 %
Residual Sulfur Dioxide	Not more than 150 mg/kg	0 mg/kg	69 mg/kg
**Extractives**	Not less than 50.0%	73.1 %	69.0 %
**Assay**			
Polysaccharide	It contains not less than 6.0 % ofpolysaccharide, calculated as glucose ($C_6H_{12}O_6$) with reference to the dried drug.	16.6 %	8.5 %
**Conclusion**	The results comply with Chinese Pharmacopoeia (2020), Volume I		

1.6.8. Cinnamomi Ramulus

**Inspection Items**	**Standard Criteria**	**Batch No. M201228-1**	**Batch No. M210207-1**
**Description**	Should correspond to the description of Cinnamomi Ramulus.	Corresponding	Corresponding
**Identification**			
(1) Microscopic Character	Should correspond to the Microscopic Character of Cinnamomi Ramulus.	Corresponding	Corresponding
(2) (3) Thin Layer Chromatography	Test solution should show corresponding spots to the cinnamic aldehyde CRS and Cinnamomi Ramulus reference drug.	Corresponding	Corresponding
**Other requirements**			
Water	Not more than 12.0 %.	10.2%	10.3%
Total ash	Not more than 3.0%.	1.8%	2.0 %
Residual Sulfur Dioxide	Not more than 150 mg/kg	0 mg/kg	0 mg/kg
**Extractives**	Not less than 6.0%	6.4%	6.9 %
**Assay**			
Cinnamaldehyde	It contains not less than 1.0 % of cinnamic aldehyde (C_9H_8O), calculated with reference to the dried drug.	1.5%	1.8 %
**Conclusion**	The results comply with Chinese Pharmacopoeia (2020), Volume I		

1.6.9. Carthami Flos

**Inspection Items**	**Standard Criteria**	**Batch No. M210227-1**	**Batch No. M210308-1**
**Description**	Should correspond to the description of Carthami Flos.	Corresponding	Corresponding
**Identification**			
(1) Microscopic Character	Should correspond to the Microscopic Character of Carthami Flos.	Corresponding	Corresponding
(2) Thin Layer Chromatography	Test solution should show corresponding spots to the Carthami Flos reference drug.	Corresponding	Corresponding
**Other requirements**			
Foreign matter	Not more than 2%.	1 %	0 %
Water	Not more than 13.0%.	12.7 %	11.6 %
Total ash	Not more than15.0%.	7.1 %	7.2 %
Acid-insoluble ash	Not more than 5.0%.	1.6%	1.4 %
Absorbance	Not less than 0.20 at 518nm	0.34	0.35
Residual Sulfur Dioxide	Not more than 150 mg/kg	42 mg/kg	0 mg/kg
**Extractives**	Not less than 30.0%	43.3%	45.5 %
**Assay**			
Hydroxysafflor yellow A	It contains not less than 1.0 % of Hydroxysafflor yellow A ($C_{27}H_{32}O_{16}$), calculated with reference to the dried drug.	2.3%	2.3%
Kaempferol	It contains not less than 0.050 % of Kaempferol ($C_{15}H_{10}O_{6}$), calculated with reference to the dried drug.	0.113%	0.133%
**Conclusion**	The results comply with Chinese Pharmacopoeia (2020), Volume I		

1.6.10. Periplocae Cortex

**Inspection Items**	**Standard Criteria**	**Batch No. M210201-1**	**Batch No. M210201-1**
**Description**	Should correspond to the description of Periplocae Cortex.	Corresponding	Corresponding
**Identification**			
(1) Microscopic Character	Should correspond to the Microscopic Character of Periplocae Cortex.	Corresponding	Corresponding
(2) Chemical reaction	Should show positive reaction.	Positive	Positive
(3) UV spectrum	An absorption should exhibit a maximum at 278nm.	A maximum absorption at 280nm	A maximum absorption at 279nm
(4) Thin Layer Chromatography	Test solution should show corresponding spots to the 4-methoxy salicylic aldehyde CRS.	Corresponding	Corresponding
**Other requirements**			
Water	Not more than 13.0 %.	10.2%	10.2%
Total ash	Not more than10.0%.	7.5%	8.2%
Acid-insoluble ash	Not more than 4.0%.	2.3%	2.5%
**Extractives**	Not less than 20.0%	27.7%	31.7%
**Assay**			
4-methoxy salicylic aldehyde	It contains not less than 0.20% of 4-methoxy salicylic aldehyde($C_8H_8O_3$), calculated on the basis dried at 60°C for 4 hours.	0.47%	0.47%
**Conclusion**	The results comply with Chinese Pharmacopoeia (2020), Volume I		

1.6.11. Citri Reticulatae Pericarpium

**Inspection Items**	**Standard Criteria**	**Batch No. Z210131-1**	**Batch No. Z210315-1**
**Description**	Should correspond to the description of Citri Reticulatae Pericarpium.	Corresponding	Corresponding
**Identification**			
(1) Microscopic Character	Should correspond to the Microscopic Character of Citri Reticulatae Pericarpium.	Corresponding	Corresponding
(2) Thin Layer Chromatography	Test solution should show corresponding spots to the hesperidin CRS	Corresponding	Corresponding
**Other requirements**			
Water	Not more than 13.0 %.	10.7%	11.9%
Aflatoxin	It contains not more than 5μg of aflatoxin B_1 per 1000g	Not detected (detection limit 0.15μg/kg)	Not detected (detection limit 0.15μg/kg)
	Not more than 10μg of the sum of aflatoxin G_2, aflatoxin G_1, aflatoxin B_2 and aflatoxin B_1 per 1000g.	Not detected. (detection limit 0.67μg/kg)	Not detected. (detection limit of aflatoxin G2, G1, B2, B1 are 0.13 μg/kg, 0.29 μg/kg, 0.10 μg/kg, 0.15 μg/kg)
Residual Sulfur Dioxide	Not more than 150 mg/kg	0 mg/kg	0 mg/kg
**Assay**			
Hesperidin	It contains not less than 3.5 % of hesperidin($C_{28}H_{34}O_{15}$), calculated with reference to the dried drug.	6.3%	7.0%
**Conclusion**	The results comply with the Chinese Pharmacopoeia (2020), Volume I		

中国河北省石家庄市高新技术开发区天山大街 238 号 PC： 050035
NO. 238, Tianshan Avenue, Hi-Tech Development Zone, Shijiazhuang, Hebei, China
TEL: 0086 311 85901719 FAX: 0086 311 85901719 http://www.yiling.cn

1.7 Analytical procedures

No.	Chinese Name	Latin Name	Reference
1	黄芪	Astragali Radix	Chinese Pharmacopoeia (2020), Volume I
2	人参	Ginseng Radix et Rhizoma	Chinese Pharmacopoeia (2020), Volume I
3	附子	Aconiti Lateralis Radix Praeparata (heishunpian)	Chinese Pharmacopoeia (2020), Volume I
4	丹参	Salviae Miltiorrhizae Radix et Rhizoma	Chinese Pharmacopoeia (2020), Volume I
5	葶苈子	Descurainiae Semen	Chinese Pharmacopoeia (2020), Volume I
6	泽泻	Alismatis Rhizoma	Chinese Pharmacopoeia (2020), Volume I
7	玉竹	Polygonati Odorati Rhizoma	Chinese Pharmacopoeia (2020), Volume I
8	桂枝	Cinnamomi Ramulus	Chinese Pharmacopoeia (2020), Volume I
9	红花	Carthami Flos	Chinese Pharmacopoeia (2020), Volume I
10	香加皮	Periplocae Cortex	Chinese Pharmacopoeia (2020), Volume I
11	陈皮	Citri Reticulatae Pericarpium	Chinese Pharmacopoeia (2020), Volume I

中国河北省石家庄市高新技术开发区天山大街 238 号 PC：050035
NO. 238, Tianshan Avenue, Hi-Tech Development Zone, Shijiazhuang, Hebei, China
TEL: 0086 311 85901719 FAX: 0086 311 85901719 http://www.yiling.cn

1.7.1 Astragali Radix
（黄芪，Huangqi）
Milkvetch Root

Description Cylindrical, some branched, upper part relatively thick, 30-90 cm long, 1-3.5 cm in diameter. Externally pale brownish-yellow or pale brown, with irregular, longitudinal wrinkles or furrows. Texture hard and tenacious, uneasily broken, fracture highly fibrous and starchy, bark yellowish-white, wood pale yellow, with radiate striations and fissures, the centre part of old root occasionally rotten-wood-shaped, blackish-brown or hollowed. Odour, weak; taste, slightly sweet and slightly bean- like on chewing.
Identification (1) Transverse section: Cork consisting of many rows of cells. Phelloderm of 3-5 rows of collenchymatous cells. Outer part of phloem rays often curved and fissured; fibres in bundles, walls thickened and lignified or slightly lignified, arranged alternately with sieve tube groups; stone cells sometimes visible near phelloderm. Cambium in a ring. Xylem vessels scattered singly or 2-3 aggregated in groups; wood fibres existing among vessels; sometimes stone cells visible in rays, singly or 2-4 in groups. Parenchymatous cells containing starch granules.
Powder: Yellowish-white. Fibres in bundles or scattered, 8-30 μm in diameter, thick-walled, with longitudinal fissures on the surface, the primary walls often separated from the secondary walls, both ends often broken to tassel- like, or slightly truncated. Bordered-pitted vessels colourless or orange, bordered pits arranged closely. Stone cells occasionally visible, rounded, oblong or irregular, slightly thick-walled.
(2) To 3 g of the powder add 20 ml of methanol and heat under reflux on a water bath for 1 hour and filter. Apply the filtrate to a prepared neutral aluminium oxide column (100-120 mesh, 5 g, 10-15 mm in inner diameter) and elute with 100 ml of 40% methanol, collect the eluate and evaporate it on a water bath to dryness. Dissolve the residue in 30 ml of water and extract by shaking with two 20-ml quantities of *n*-butanol saturated with water, combine the *n* -butanol solutions and wash with two 20-ml quantities of water. Discard the water solution and evaporate the *n*-butanol solution to dryness on a water bath. Dissolve the residue in 0.5 ml of methanol as the test solution. Dissolve astragaloside IV CRS in methanol to produce a solution containing 1 mg per ml as the reference solution. Carry out the method for thin layer chromatography *<0502>* using silica gel G as the coating substance and the lower layer of a mixture of chloroform, methanol and water (13:7:2) as the mobile phase. Apply separately to the plate 2 μl of each of the above two solutions. After developing and removal of the plate, dry in air, spray with a 10% solution of sulfuric acid in ethanol, and heat at 105°C to the spots distinct. A brown spot in the chromatogram obtained with the test solution corresponds in position and colour to the spot in the

chromatogram obtained with the reference solution. Examine under ultraviolet light at 365 nm, the same orange-yellow fluorescent spots are shown in both chromatogams.
(3) To 2 g of the powder add 30 ml of ethanol, heat under reflux for 20 minutes, filter and evaporate the filtrate to dryness. Dissolve the residue in 15 ml of 0.3% solution of sodium hydroxide and filter. Adjust the filtrate to pH 5-6 with dilute hydrochloric acid, extract with 15 ml of ethyl acetate by shaking, separate the ethyl acetate solution and filter through a filter paper covered with a quantity of anhydrous sodium sulfate. Evaporate the filtrate to dryness and dissolve the residue in 1 ml of ethyl acetate as the test solution. Prepare a solution of Astragali Radix reference drug in the same manner as the reference drug solution. Carry out the method for thin layer chromatography <*0502*>, using silica gel G as the coating substance and a mixture of chloroform and methanol (10:1) as the mobile phase. Apply separately to the plate 10 µl of each of the above two solutions. After developing and removal of the plate, dry in air, expose to ammonia vapour and examine under ultraviolet light at 365 nm. The main fluorescent spots in the chromatogram obtained with the test solution correspond in position and colour to the spots obtained with the reference drug solution.
Water Not more than 10.0 per cent <*0832, method 2*>.
Total ash Not more than 5.0 per cent <*2302*>.
Heavy metals and harmful elements Carry out the methods for determinations of lead, cadmium, arsenic, mercury and copper <*2321, atomic absorption spectrophotometry, or inductively coupled plasma mass spectrometry*) , not more than 5 mg/kg of lead, 0.3 mg/kg of cadmium, 2 mg/kg of arsenic, 0.2 mg/kg of mercury and 20 mg/kg of copper.
Pesticide residues Carry out the method for determination of pesticide residues <*2341, organochlorine pesticide residues, method 1*>, not more than 0.2 mg/kg of benzene hexachloride (BHC) (sum of α-,β-,γ-, y- and δ-BHC), 0.2 mg/kg of dichlorodiphenyl trichloroethane (DDT) (sum of pp'-DDE, pp'-DDD, op'-DDT and pp'-DDT), and 0.1 mg/ kg of pentachloronitrobenzene (PCNB) .
Sulfur dioxide residue Not more than 150mg/kg<2331>.
Extractives Carry out the method for determination of water-soluble extractives <*2201, the cold maceration method*>, not less than 17.0 per cent.
Assay *Astragaloside IV* Carry out the method for high performance liquid chromatography <*0512*>.
Chromatographic system and system suitability Use octadecylsilane bonded silica gel as the stationary phase and a mixture of acetonitrile and water (32:68) as the mobile phase. Use an evaporative light scattering detector. The number of theoretical plates of the column is not less than 4000, calculated with reference to the peak of astragaloside IV.
Reference solution Weigh accurately a quantity of astragaloside IV CRS, dissolve in methanol to produce a solution containing 0.5 mg per ml.

Test solution Weigh accurately 4 g of the powder to a Soxhlet's extractor, add 40 ml of methanol, macerate overnight, add again an appropriate quantity of methanol, heat under reflux on a water bath for 4 hours, concentrate the extracts to dryness.

Add 10 ml of water and slightly heat to dissolve the residue, extract by shaking with four 40-ml quantities of *n*-butanol saturated with water, combine the extracts and wash with two 40-ml quantities of ammonia TS. Discard the ammonia solutions and evaporate the *n*-butanol extracts to dryness, dissolve the residue in 5 ml of water and cool. Apply it to a column (1.5 cm in inner diameter, 12 cm in length) packed with D101 macroporous resin, elute with 50 ml of water, 30 ml of 40% ethanol, 80 ml of 70% ethanol successively, discard the water and 40% ethanol eluates, collect the 70% ethanol eluates and evaporate to dryness. Dissolve the residue in methanol, transfer to a 5 ml volumetric flask, dilute with methanol to volume and mix well.

Procedure Inject accurately 10 μl, 20 μl of the reference solution and 20 μl of the test solution, respectively, into the column, and calculate the content, using a calibration equation of logarithm conversion of external standard method of two points.

It contains not less than 0.040 per cent of astragaloside IV ($C_{41}H_{68}O_{14}$), calculated with reference to the dried drug.

Calycosin-7-O-β-D-glycoside Carry out the method for high performance liquid chromatography *<0512>*.

Chromatographic system and system suitability Use octadecylsilane bonded silica gel as the stationary phase, acetonitrile as the mobile phase A and a 0.2% solution of formic acid as the mobile phase B, elute in gradient as the following:

Time (min)	Mobile phase A (per cent *V/V*)	Mobile phase B (per cent *V/V*)
0-20	20→40	80→60
20-30	40	60

As detector a spectrophotometer set at 260 nm. The number of theoretical plates of the column is not less than 3000, calculated with reference to the peak of calycosin-7-*O-β-D-glycoside*.

Reference solution Dissolve a quantity of calycosin-7-O-β-D-glycoside CRS, accurately weighed, in methanol to produce a solution containing 50 μg per ml.

Test solution Weigh accurately 1 g of the powder (through No. 4 sieve) to a stoppered eonical flask, accurately add 50 ml of methanol and weigh. Heat under reflux for 4 hours, cool and weigh again, replenish the loss of solvent with methanol, mix well and filter. Measure accurately 25 ml of the successive filtrate and evaporate to dryness. Dissolve the residue in methanol and transfer to a 5 ml volumetric flask, dilute with methanol to volume, mix well, filter and use the successive filtrate as the test solution.

Procedure Inject accurately 10 μl of each of the reference solution and the test solution, respectively, into the column, and calculate the content.

It contains not less than 0.020 per cent of calycosin-7-*O*-*β*-D-glycoside ($C_{22}H_{22}O_{10}$), calculated with reference to the dried drug.

1.7.2 Ginseng Radix et Rhizoma
(人参，Renshen)
Ginseng

Description Main roots fusiform or cylindrical, 3-15 cm long, 1-2 cm in diameter; externally greyish-yellow, the upper part or entire root exhibiting sparse, shallow, interrupted and coarse transverse-striations and distinct longitudinal wrinkles; the lower part bearing 2-3 branch roots and numerous slender rootlets with inconspicuous minute tubercles. Rhiwmes (Lutou) 1-4 cm long, 0.3-1.5 cm in diameter, mostly constricted and curved, bearing adventitious roots (Ding) and showing sparse depressed circular stem scars (Luwan). Texture relatively hard, fracture yellowish-white, starchy, cambium ring brownish-yellow, bark exhibiting yellow-brown dotted resin canals and radial clefts. Odour, characteristic; taste, slightly bitter and sweet. Alternatively, main roots as long as or shorter than rhizome, cylindrical, rhomboid or V-shaped, 1-6 cm long; externally greyish-yellow, longitudinally wrinkled, the upper or middle-lower part with annulations, branch roots mostly 2-3, rootlets less and slender, orderly arranged and showing some distinct warts. Rhizomes slender, a few stout, the upper part exhibiting sparse or dense deep depressed stem scars, adventitious roots relatively thin, mostly reclinate.
Identification (l) Transverse section: Cork consisting of several rows of cells. Phelloderm narrow. Phloem showing clefts in the outer part, and parenchymatous cells densely arranged and scattered with resin canals, containing yellow secretions in the inner part. Cambium in a ring. Xylem rays broad, vessels singly scattered or grouped, interruptedly arranged radially, occasionally accompanied by non-lignified fibres. Parenchymatous cells containing clusters of calcium oxalate.
Powder: Pale yellowish-white. Fragments of resin canals easily visible, containing yellow masses of secretion. Clusters of calcium oxalate 20-68 μm in diameter, with acute angles. Cork cells subsquare or polygonal with sinuous walls in surface view. Reticulated and scalariform vessels 10-56 μm in diameter. Starch granules fairly abundant, simple granules subspheroidal, semi-circular or irregular polyonal, 4-20 μm in diameter, hilum pointed or slit-shaped; compound granules consisting of 2-6 components.
(2) To 1 g of the powder add 40 ml of chloroform, heat under reflux on a water bath for 1 hour, discard the chloroform layer, evaporate the residue to dryness. Moisten the residue with 0.5 ml of water, add 10 ml of *n*-butanol saturated with water, ultrasonicate for 30 minutes. To the supernatant liquid add 3 volumes of ammonia TS, mix well, stand for demixing. Evaporate the supernatant liquid to dryness, dissolve the residue in 1 ml of methanol as the test solution. Prepare a solution of 1 g of Ginseng Radix et Rhizoma reference drug in the same manner as the reference drug solution. Dissolve ginsenosides Rb_1 CRS, Re CRS, Rf CRS and Rg_1 CRS in methanol to produce a mixture containing 2 mg of each per ml as the reference solution. Carry out the method for thin layer chromatography *<0502>* , using si lica gel Gas the coating substance and the lower layer of a mixture of chloroform, ethyl acetate, methanol and water (15:40:22:10), standing below l0°C , as the mobile phase. Apply separately to the plate 1-2 μl of each of the above three solutions. After developing and removal of the plate, dry in air, spray with a 10% solution of sulfuric acid in

ethanol, heat at 105°C to the spots clear. Examine in daylight and under ultraviolet light at 365 nm. The spots or fluorescent spots in the chromatograms obtained with the test solution correspond in position and colour to the spots or fluorescent spots in the chromatograms obtained with the reference drug solution and the reference solution.

Water Not more than 12.0 per cent *<0832, method 2>*.

Total ash Not more than 5.0 per cent *<2302>*.

Heavy metals and harmful elements Carry out the methods for determinations of lead, cadmium, arsenic, mercury and copper *<2321, atomic absorption spectrophotometry, or inductively coupled plasma mass spectrometry*) , not more than 5 mg/kg of lead, 0.3 mg/kg of cadmium, 2 mg/kg of arsenic, 0.2 mg/kg of mercury and 20 mg/kg of copper.

Pesticide Residues Carry out the method for determination of pesticide residues *<2341, detennination of organochlonne pesticide residues, method 2>*. Not more than 0.2 mg/kg of hexachlorocyclohexane (BHC) (sum of α-BHC, β-BHC, γ-BHC and δ-BHC), 0.2 mg/kg of chlorophenothane (DDT) (sum of pp'-DDE, pp'-DDD, op'-DDT, pp'-DDT), 0.1 mg/ kg of pentachloronitrobenzene (PCNB), 0.1 mg/kg of hexachlorobenzene, 0.05 mg/kg of heptachlor (sum of heptachlor and heptachlor epoxide), 0.05 mg/kg of aldrin, 0.1 mg/kg of chlordane (sum of *cis*-chlordane, *trans*-chlordane and *oxy*-chlordane).

Sulfur dioxide residue Not more than 150mg/kg<2331>.

Assay Carry out the method for high performance liquid chromatography *<0512>*.

Chromatographic system and system suitability Use octadecylsilane bonded silica gel as the stationary phase, acetonitrile as the mobile phase A and water as the mobile phase B, elute in gradient as the following:

As detector a spectrophotometer set at 203 nm. The number of theoretical plates of the column is not less than 6000, calculated with reference to the peak of ginsenoside Rg_1.

Time (min)	Mobile phase A (per cent *V/V*)	Mobile phase B (per cent *V/V*)
0-35	19	81
35-55	19→29	81→71
55-70	29	71
70-100	29→40	71→60

Reference solution Dissolve ginsenosides Rg_1 CRS, Re CRS, Rf CRS and Rb_1 CRS, accurately weighed, in methanol to produce a mixture containing 0.2 mg of each per ml as the reference solution.

Test solution Weigh accurately 1 g of the powder (through No. 4 sieve) to a Soxhlet's extractor, add 40 ml of chloroform, heat under reflux on a water bath for 3 hours, discard the chloroform solution, expel the solvent from the residue. Transfer it with the filter paper tube into a 100 ml conical flask. Accurately add 50 ml of *n*-butanol saturated with water, tightly stopper, allow to stand overnight, ultrasonicate (power 250 W, frequency 50 kHz) for 30 minutes and filter. Evaporate accurately 25 ml of the successive filtrate to dryness in an evaporating dish, dissolve the residue in methanol,

中国河北省石家庄市高新技术开发区天山大街 238 号 PC：050035
NO. 238, Tianshan Avenue, Hi-Tech Development Zone, Shijiazhuang, Hebei, China
TEL: 0086 311 85901719 FAX: 0086 311 85901719 http://www.yiling.cn

transfer to a 5 ml volumetric flask, dilute with methanol to volume and mix well.
Procedure Inject accurately 10 μl of the reference solution and 10-20 μI of the test solution, respectively, into the column, and calculate the content.
It contains not less than 0.30 per cent of the total amount of gensenoside Rg_1 ($C_{42}H_{72}O_{14}$) and ginsenoside Re ($C_{48}H_{82}O_{18}$), and not less than 0.20 per cent of gensenoside Rb_1 ($C_{54}H_{92}O_{23}$), calculated with reference to the dried drug.

1.7.3 Aconiti Lateralis Radix Praeparata (heishunpian)
（附子，Fuzi）
Prepared Common Monkshood Daughter Root

Description *Heishunpian* (*black slice*) Longitudinal slices, the upper portion wide and the lower portion narrow, 1.7-5 cm long, 0.9- 3 cm wide, 0.2-0.5 cm thick. The outer bark blackish-brown, cut surface dark yellow, oily and lustrous, translucent and showing longitudinal vascular bundles. Texture hard and fragile. Fracture horny. Odour, slight; taste, weak.

Identification Macerate 2 g of the powder with 3 ml of ammonia TS, add 25 ml of ether, ultrasonicate for 30 minutes and filter, evaporate the filtrate to dryness and dissolve in 0.5 ml of dichloromethane as the test solution. Dissolve benzoylmesaconine CRS, benzoylaconitine CRS and benzoylhypaconitine CRS and in a mixture of isopropanol and dichloromethane (1:1) to produce a mixture containing 1 mg of each per ml as the reference solution (monoesteralkaloids). Dissolve a quantity of mesaconine CRS, hypaconitine CRS and aconitine CRS in a mixture of isopropanol and dichloromethane (1:1) to produce a mixture containing 1 mg of each per ml as the reference solution (diester-alkaioids). Carry out the method for thin layer chromatography *<0502>*, using silica gel G as the coating substance and a mixture of *n*-hexane, ethyl acetate and methanol (6.4:3.6:1) as the mobile phase. Apply separately to the plate 5-10 μl of each of the test solution and the reference solutions. After developing in a chamber preequilibrated with ammonia vapor for 20 minutes and removal of the plate, dry in air, spray with dilute potassium iodobismuthate TS. The spots in the chro-matogram obtained with the test solution of *Yanfuzi* correspond in position and colour to the spots of mesaconine CRS, hypaconitine CRS and aconitine CRS obtained with the reference solution. The spots in the chromatogram obtained with the test solution of *Heishunpian or Baifupian* correspond in position and colour to the spots of benzoylaconitine CRS, benzoylhypaconitine CRS and benzoylmesaconine CRS obtained with the reference solution.

Water Not more than 15.0 per cent *<0832, method 2>*.

Diester-alkaloids *Chromatographic system and system suitability and Test solution* As described under Assay.

Reference solution Weigh accurately mesaconine CRS, hypaconitine CRS and aconitine CRS respectively, dissolve in a mixture of isopropanol and dichloromethane (1:1) to produce a mixture containing 5 μg of each per ml.

Procedure Inject accurately 10 μl of each of the reference solution and the test solution obtained under Assay, respectively, into the column, and calculate the content. It contains not more than 0.020 per cent of the total amount of mesaconine ($C_{33}H_{45}NO_{11}$), hypaconitine ($C_{33}H_{45}NO_{10}$) and aconitine ($C_{34}H_{47}NO_{11}$), calculated with reference to the dried drug.

Sulfur dioxide residue Not more than 150mg/kg<2331>.

Assay Carry out the method for high performance liquid chromatography *<0512>*.

Chromatographic system and system suitability Use octadecylsilane bonded silica gel as the stationary phase, a mixture of acetonitrile and tetrahydrofuran (25:15) as the mobile phase A, a 0.1 mol/L solution of ammonium acetate (add 0.5 ml of glacial acetic acid per 1000 ml) as the mobile phase B, elute in gradient as the following:

Time (min)	Mobile phase A (per cent *V/V*)	Mobile phase B (per cent *V/V*)
0-48	15→26	85→74
48-49	26→35	74→65
49-58	35	65
58-65	35→15	65→85

As detector a spectrophotometer set at 235 nm. The number of theoretical plates of the column is not less than 3000, calculated with reference to the peak of benzylmesaconine.

Reference solution Dissolve benzoylmesaconine CRS, benzoylaconitine CRS and benzoylhypaconitine CRS in a mixture of isopropanol and dichloromethane (1:1) to produce a mixture containing 10 µg of each per ml.

Test solution Weigh accurately 2 g of the powder (through No. 3 sieve) to a stoppered conical flask, accurately add 3 ml of ammonia TS and 50 ml of a mixture of isopropanol and ethyl acetate (1:1) and weigh. Ultrasonicate (power 300 W, frequency 40 kHz, water temperature below 30°C) for 30 minutes, cool and weigh again, replenish the loss of solvent with the mixture of isopropanol and ethyl acetate (1:1) and mix well. Evaporate accurately 25 ml of the successive filtrate to dryness under reduced pressure below 40°C. Dissolve exactly the residue in 3 ml of a mixture of isopropanol and dichloromethane (1:1), mix well, filter and use the filtrate as the test solution.

Procedure Inject accurately 10 µl of each of the reference solution and the test solution, respectively, into the column, and calculate the content.

It contains not less than 0.010 per cent of the total amount of benzoylmesaconine ($C_{31}H_{43}NO_{10}$), benzoylaconitine ($C_{32}H_{45}NO_{10}$), and benwylhypaconitine ($C_{31}H_{43}NO_9$), calculated with reference to the dried drug.

1.7.4 Salviae Miltiorrhizae Radix et Rhizoma
（丹参，Danshen）
Danshen Root

Description Rhizomes short and stout, sometimes with remains of a stem at the apex. Several roots, long cylindrical, slightly curved, some branched and with rootlets, 10-20 cm long, 0.3-1 cm in diameter. Externally brownish-red or dark brownish-red, rough, longitudinally wrinkled. The bark of old roots loose, mostly purplish-brown, usually scaling off. Texture hard and fragile, fracture loose with clefts or slightly even and dense, with brownish-red bark and greyish-yellow or purplish-brown wood, showing bundles of vessels, yellowish-white, arranged radially. Odour, slight; taste, slightly bitter and astringent.
Cultivars relatively stout, 0.5-1.5 cm in diameter. Externally reddish-brown, longitudinally wrinkled, the bark closely adhering to wood and uneasy to be scaled off. Texture compact, fracture relatively even, slight horny.
Identification (1) Powder: Reddish-brown Stone cells subrounded, subtriangular, oblong or irregular, some elongated in fibre-shaped, edge uneven, 14-70 μm in diameter, up to 257 μm long, pit canals distinct, some lumina containing yellowish-brown content. Xylary fibres mostly consisting of fibre tracheids, long shuttle-shaped, ends oblique pointed or obtuse, 12-27 μm in diameter, bordered pits point-shaped, pits oblique cleft-shaped or cross-shaped, pit canals sparse. Reticulate and bordered pitted vessels 11-60 μm in diameter.
(2) To 1 g of the powder add 5 ml of ethanol, ultrasonicate for 15 minutes, centrifuge, and use the supernatant as the test solution. Prepare a solution of 1 g of Salviae Miltiorrhizae Radix et Rhizoma reference drug in the same manner as the reference drug solution. Dissolve tanshinone II_A CRS and salvianolic acid B CRS in ethanol to produce a mixture containing 0.5 mg and 1.5 mg per ml, respectively, as the reference solution. Carry out the method for thin layer chromatography <*0502*>, using silica gel G as the coating substance, a mixture of trichloromethane, methylbenzene, ethyl acetate, methanol and formic acid (6:4:8:1:4) as the mobile phase. Apply separately 5 μl of each of the above three solutions to the plate. After developing for about 4 cm and removal of the plate, dry in air. Using a mixture of petroleum ether (60-90°C) and ethyl acetate (4:1) as the mobile phase, after developing again for about 8 cm and removal of the plate, dry in air, examine in daylight and under ultraviolet light at 365nm respectively. The spots or fluorescence spots in the chromatogram obtained with the test solution correspond in position and colour to the spots in the chromatogram obtained with the reference drug solution and reference solution.
Water Not more than 13.0 percent <*0832, method 2*>.
Total ash Not more than 10.0 percent <*2302*>.
Acid-insoluble ash Not more than 3.0 percent <*2302*>.

石家庄以岭药业股份有限公司
YILING SHIJIAZHUANGYILING PHARMACEUTICAL CO.,LTD
中国河北省石家庄市高新技术开发区天山大街 238 号 PC： 050035
NO. 238, Tianshan Avenue, Hi-Tech Development Zone, Shijiazhuang, Hebei, China
TEL: 0086 311 85901719 FAX: 0086 311 85901719 http://www.yiling.cn

Heavy metals and harmful elements Carry out the methods for determination of lead, cadmium, arsenic, mercury and copper *<2321, atomic absorption spectra-photometry or inductively coupled plasma mass spec-trometry>*, not more than 5 mg/kg of lead, 0.3 mg/kg of cadmium, 2 mg/kg of arsenic, 0.2 mg/kg of mercury and 20 mg/kg of copper.

Sulfur dioxide residue Not more than 150mg/kg<2331>.

Extractives *Water-soluble extractives* Carry out the method for determination of water-soluble extractives *<2201, the cold maceration method>*, not less than 35.0 percent.

Ethanol-soluble extractives Carry out the method for determination of ethanol-soluble extractives <2201, the hot extraction method>, using ethanol as the solvent, not less than 15.0 percent.

Assay ***Tanshinones*** Carry out the method for high performance liquid chromatography <0512>.

Chromatographic system and system suitability Use octadecylsilane bonded silica gel as the stationary phase, acetonitrile as the mobile phase A and 0.02% phosphoric acid solution as the mobile phase B, elute in gradient as the following:

Time (min)	Mobile phase A (per cent *V/V*)	Mobile phase B (per cent *V/V*)
0-6	61	39
6-20	61→90	39→10
20-20.5	90→61	10→39
20.5-25	61	39

Column temperature 20°C, as detector a spectrophotometer set at 270 nm. The number of theoretical plates of the column is not less than 60 000, calculated with reference to the peak of tanshinone II_A.

Reference solution Weight accurately a quantity of tanshinone II_A CRS to an amber volumetric flask, dissolve in methanol to produce a solution containing 20 µg per ml as the reference solution.

Test solution Weight accurately 0.3 g of the powder (through No.3 sieve) in a stopper conical flask, add accurately 50 ml of methanol, weigh and ultrasonicate (power 140 W, frequency 42 kHz) for 30 minutes. Cool, weigh again, replenish the loss of weight with methanol, mix well, filter, and use the successive filtrate as the test solution.

Procedure Inject accurately 10 µl of each of the reference solution and the test solution into the column, calculate the content. With the peak of tanshinone II_A as the reference, calculate the relative retention time of cryptotanshinone and tanshinone I. The relative retention times and correction factors of compounds as following:

The compound (peak)	Relative retention (time)	Correction factor
cryptotanshinone	0.75	1.18
tanshinone I	0.79	1.31
tanshinone II_A	1.00	1.00

The relative retention time of the test peak should keep in±5% of the above values. With the peak area of tanshinone II_A as the external standard calculate the contents of cryptotanshinone, tanshinone I and tanshinone II_A with the corresponding correction factors.
It contains not less than 0.25 per cent of the total amount of tanshinone II_A ($C_{19}H_{18}O_3$), tanshinone I ($C_{19}H_{20}O_3$) and cryptotanshinone ($C_{18}H_{12}O_3$), calculated with reference to the dried drug.
Salvianoic acid B Carry out the method for high performance liquid chromatography *<0512>*.
Chromatographic system and system suitability Use octadecylsilane bonded silica gel as the stationary phase, acetonitrile and 0.1% phosphoric acid solution (22:78) as the mobile phase, column temperature 20°C, flow rate 1.2 ml per minutes, as detector a spectrophotometer set at 286 nm. The number of theoretical plates of the column is not less than 6000, calculated with reference to the peak of salvianoic acid B.
Reference solution Dissolve a quantity of salvianoic acid B CRS, weight accurately, in 80% methanol to produce a solution containing 0.10 mg per ml as the reference solution.
Test solution Weight accurately 0.15 g of the powder (through No.3 sieve) in a stopper conical flask, add accurately 50 ml of 80% methanol, weigh, ultrasonicate (power 140W, frequency 42 kHz) for 30 minutes, cool and weigh again, replenish the loss of weight with 80% methanol, mix well and filter. Measure accurately 5 ml of the successive filtrate to a 10 ml volumetric flask, dilute with 80% methanol to volume, mix well, filter, and use the successive filtrate as the test solution.
Procedure Inject accurately 10 μl of each of the reference solution and the test solution into the column, calculate the content.
It contains not less than 3.0 percent of salvianoic acid B ($C_{36}H_{30}O_{16}$), calculated with reference to the dried drug.

1.7.5 Descurainiae Semen
(葶苈子Tinglizi)
Pepperweed Seed

Description Seed of *Descurainia Sophia* Flattened-ovoid, 0.8-1.2 mm long, about 0.5 mm wide. Externally brown or reddish-brown, somewhat lustrous, with 2 longitudinal furrows, one of them relatively distinct. One end obtuse, the other flat or slightly concave, hilum off-white, situated at the concave end. Odour, slight; taste, slightly pungent and bitter, relatively viscous.
Identification (1) Macerate a small quantity of the drug in water, observe under a magnifier, the transparent mucilage layer of *Seed of Descurainia sophia* is thin and occurs in less than about 1/5 of the width of seed.
(2*) Seed of Descurainia sophia* Powder: Yellowish-brown. Epidermal cells of testa consisting of mucilage cells, subsquare in section view, thickened inner-walls extending outwards to form cellulose columns, cellulose columns 8-18 μm long, obtuse-rounded, oblique or truncate at apex, mucilage striations visible in periphery. Inner epidermal cells of testa yellow, longpolygonal in surface view, 15-42 μm in diameter, walls 5-8 μm thick.
(3) *Seed of Descurainia sophia* To 1 g of the powder, add 20 ml of 70% ethanol, heat under reflux for 1 hour, filter, and use the filtrate as the test solution. Dissolve quercetin-3-*O*-*β*-D-glucose-7-*O*-*β*-D-gentiobioside CRS in 30% methanol to produce a solution containing 90 μg per ml as the reference solution. Carry out the method for thin layer chromatography *<0502>*, using polyamide as the coating substance and a mixture of ethyl acetate, methanol and water (7:2:1) as the mobile phase. Apply separately 1 μl of each of the above two solutions to the film. After developing and removal of the film, dry in air. Spray with a solution of a solution of 2% aluminum chloride in ethanol, dry in a current of hot air; examine under ultraviolet light at 365 nm. The yellow fluorescent spot in the chromatogram obtained with the test solution corresponds in position and colour to the fluorescent spot in the chromatogram obtained with the reference solution
Water Not more than 9.0 per cent *<0832, method 2>*.
Total ash Not more than 8.0 per cent *<2302>*.
Acid-insoluble ash Not more than 3.0 per cent *<2302>*.
Swelling capacity Weigh about 0.6 g of the drug, carry out the method for the determination of swelling capacity *<2101>*, not less than 3 for Descurainiae Semen.
Sulfur dioxide residue Not more than 150mg/kg<2331>.
Assay *Seed of Descurainia sophia* Carry out the method for high performance liquid chromatography *<0512>*.
Chromatographic system and system suitability Use octadecylsilane bonded silica gel as the stationary phase and a mixture of acetonitrile and 0.1% acetic acid (11:89) as the mobile phase. As detector a spectrophotometer set at 254 nm. The number of

theoretical plates of the column is not less than 5800, calculated with the reference to the peak of quercetin-3-*O*-*β*-D-glucopyranosyl-7-*O*-*β*-D-gentiobioside.

Reference solution Weigh accurately a quantity of quercetin-3-*O*-*β*-D-glucopyranosyl-7-*O*-*β*-D-Gentiobioside CRS, dissolved in the 30% methanol to produce a solution of 20 µg per ml as the reference solution.

Test solution Weigh accurately 1 g of the powder (through No. 4 sieve) in a conical flask, add accurately 50 ml of 70% methanol, weigh accurately, heat under reflux for 1 hour, allow to cool and weigh again, replenish the loss in weight with 70% methanol, mix well and collect the successive filtrate as the test solution.

Procedure Inject accurately 25 µl of each of the reference solution and test solution respectively into column, and calculate the content.

It contains not less than 0.075 per cent of-3-*O*-*β*-D-glucopyranosyl-7-*O*-*β*-D-gentiobioside ($C_{33}H_{40}O_{22}$), calculated with reference to the dried drug.

1.7.6 Alismatis Rhizoma
（泽泻，Zexie）
Oriental Waterplantain Rhizome

Description Subspherical, elliptical or ovate, 2-7 cm long, 2-6 cm in diameter. Externally light yellow to yellowish-brown, with irregular transverse-annular shallow furrows and numerous small raised fibrous root scars, occasionally tuberculate bud scars attached to the base. Texture compact, fracture yellowish-white, starchy, with numerous small pores. Odour, slight; taste, slightly bitter.

Identification (1) Powder: Yellowish-brown. Starch granules numerous, simple granules long-ovoid, subspherical or ellipsoid, 3-14 µm in diameter, hilurn V-shaped, shortly slit-shaped or Y-shaped; compound granules of 2-3 components. Parenchymatous cells subrounded, with many elliptical pits aggvegated into pit areas. Anticlinal walls of endodermis cells sinuous, relatively thick, lignified, with sparse and minute pit-canals. Oil cavities mostly broken, whole ones subrounded, 54-110 µm in diameter, sometimes oil drops in secretory cells visible.

(2) To 2 g of the powder add 20 ml of ethyl acetate, ultrasonicate for 30 minutes and filter. Apply the filtrate to an alumina column (200-300 mesh, 5 g, 1 cm in inner diameter, packed by dry method), elute with 10 ml of ethyl acetate, collect the eluates and evaporate to dryness, dissolve the residue in 1 ml of ethyl acetate as the test solution. Dissolve 23-acetate alisol B CRS in ethyl acetate to produce a solution containing 2 mg per ml as the reference solution. Carry out the method for thin layer chromatography *<0502>*, using silica gel H as the coating substance and a mixture of cyclohexane and ethyl acetate (1:1) as the mobile phase. Apply separately to the plate 5 µl of each of the above two solutions. After developing and removal of the plate, dry in air, spray with a 5% solution of silicotungstic acid in ethanol and heat at 105°C to the spots clear. The spot in the chromatogram obtained with the test solution corresponds in position and colour to the spot in the chromatogram obtained with the reference solution.

Water Not more than 14.0 per cent *<0832, method 2>*.

Total ash Not more than 5.0 per cent *<2302>*.

Sulfur dioxide residue Not more than 150mg/kg<2331>.

Extractives Carry out the method for determination of ethanol-soluble extractives *<2201, the hot extraction method>*, using ethanol as the solvent, not less than 10.0 per cent.

Assay Carry out the method for high performance liquid chromatography *<0512>*.

Chromatographic system and system suitability Use octadecylsilane bonded silica gel as the stationary phase and a mixture of acetonitrile and water (73:27) as the mobile phase. As detector a spectrophotometer set at 208 nm. The number of theoretical plates of the column is not less than 3000, calculated with reference to the peak of 23-acetate alisol B.

中国河北省石家庄市高新技术开发区天山大街 238 号 PC：050035
NO. 238, Tianshan Avenue, Hi-Tech Development Zone, Shijiazhuang, Hebei, China
TEL: 0086 311 85901719 FAX: 0086 311 85901719 http://www.yiling.cn

Reference solution Weigh accurately a quantity of 23-acetate alisol B CRS, dissolve in acetonitrile to produce a solution containing 20 µg per ml.

Test solution Weigh accurately 0.5 g of the powder (through No. 5 sieve) to a stoppered conical flask, add accurately 25 ml of acetonitrile, stopper tightly, weigh and ultrasonicate (power 250 W, frequency 50 kHz) for 30 minutes, cool and weigh again, replenish the loss of the solvent with acetonitrile, mix well, filter and use the successive filtrate as the test solution.

Procedure Inject accurately 10 µl of each of the reference solution and the test solution, respectively, into the column, and calculate the content.

It contains not less than 0.050 per cent of 23-acetate alisol B ($C_{32}H_{50}O_5$), calculated with reference to the dried drug.

1.7.7 Polygonati Odorati Rhizoma
（玉竹，Yuzhu）
Fragrant Solomonseal Rhizome

Description Long cylindrical, slightly flattened, occasionally branched, 4-18cm long, 0.3-1.6 cm in diameter. Externally yellowish-white or pale yellowish-brown, translucent, with longitudinal wrinkles and slightly raised annulations, exhibiting white rounded-dotted fib rous root scars and a disk-like stem scar. Texture hard and fragile or slightly soft, easily broken, fracture horny or granular. Odour slight; taste, sweetish and viscous on chewing.
Identification Transvese section: Epidermal cells oblate or flattened- rectangular, outer walls slightly thickened, cutinized. Numerous mucilage cells scattered throughout parenchyma, 80-140 μm in diameter, containing raphides of calcium oxalate. Collateral bundles as well as a few amphivasal bundles scattered.
Water Not more than 16.0 per cent *<0832, method 2>*. ‘
Total ash Not more than 3.0 per cent *<2302>*.
Sulfur dioxide residue Not more than 150mg/kg<2331>.
Extractives Carry out the method for determination of ethanol-sotuble extraetives *<2201, the cold maceration method>*, using 70% ethanol as the solvent, not less than 50.0 per cent.
Assay *Reference solution* Weigh accurately a quantity of anhydrous glucose CRS, dissolve in water to prepare a solution containing 0.6 mg of anhydrous glucose per ml.
Calibration standard Measure accurately 1.0, 1.5, 2.0, 2.5, 3.0 ml of the reference solution in 50 ml volumetric flasks, respectively, dilute with water to volume and mix well. Transfer accurately 2 ml of each of the resulting solutions to stopper tubes respectively, add 1 ml of 4% phenol solution, mix well, add 7.0 ml of sulfuric acid quickly, shake, keep in a 40°C water bath for 30 minutes, then move to ice bath for 5 minutes. Carry out the method for ultraviolet-visible spectrophotometry *<0401>*, measure the absorbance at 490 nm, taking the mixture of corresponding solvents as a blank, and plot the standard curve, using absorbance as ordinate and concentration as abscissa.
Procedure Weigh accurately 1 g of the coarse powder to a round bottom flask, add 100 ml of water, heat under reflux for 1 hour, filter with defatted cotton. Repeat the extraction as above once, combine the filtrates, concentrate and transfer to a 100 ml volumetric flask, dilute with water to volume, mix well. Measure accurately 2 ml of the solution, add 10 ml of ethanol, stir and centrifuge. Dissolve the precipitate in water, transfer to a 50 ml volumetric flask, and dilute to volume. Measure 2 ml of the resulting solution , carry out the procedure as described under *Calibration standard*, begining at the words"add 1 ml of 4% phenol" , measure the absorbance, read out the weight (mg) of the anhydraous glucose of the test solution from the standard curve and calculate.

中国河北省石家庄市高新技术开发区天山大街238号 PC：050035
NO. 238, Tianshan Avenue, Hi-Tech Development Zone, Shijiazhuang, Hebei, China
TEL: 0086 311 85901719 FAX: 0086 311 85901719 http://www.yiling.cn

It contains not less than 6.0 per cent of polysaccharide, calculated as glucose ($C_6H_{12}O_6$) with reference to the dried drug.

1.7.8 Cinnamomi Ramulus
（桂枝，Guizhi）
Cassia Twig

Description Long cylindrical, much-branched, 30-75 cm long, thick end 0.3-1 cm in diameter. Externally brown to reddish-brown, with longitudinal ridges, fine wrinkles, dotted leaf-scars, branch-scars and bud-scars, lenticels dotted. Texture hard and fragile, easily broken. Slices 2-4 mm thick, cut surface showing reddish-brown in bark, yellowish-white to pale yellowish-brown in wood, pith subsquare. Odour, characteristically aromatic; taste, sweet and slightly pungent, relatively strong for bark.

Identification (1) Transverse section: Epidermis consisting of 1 layer of cells, non-glandular hairs unicellular, visible in young branches. Cork consisting of 3-5 layers of cells, the inmost cells with thickened outer walls. Oil cells and stone cells scattered in cortex. Groups of stone cells in pericycle interruptedly arranged in a ring, accompanied by fibre bundles. Secretory cells and fibres scattered in phloem. Cambium distinct. Xylem rays 1-2 cells wide, containing brown contents; vessels scattered singly or 2 to several aggregated; xylary fibres with relatively thin walls, and differentiated uneasily from xylary parenchymatous cells. In pith, the walls of cells slightly thickened and lignified. Cells of rays containing fine needle crystals of calcium oxalate.

Powder: reddish-brown. Stone cells subsquare or subrounded, 30-64 μm in diameter, walls thickened, some walls thin at one side. Most of bast fibres in bundles or isolated, colourless or brown, fusiform, some margin serrate, 12-40 μm in diameter, with heavily thickened walls, lignified, pit canals indistinct. Oil cells subrounded or elliptical, 41-104 μm in diameter. Xylary fibres numerous, usually in bundles, pits oblique or crossed. Cork cells yellowish-brown, polygonal in surface view, containing reddish-brown matter. Vessels mainly with bordered pits, up to 76 μm in diameter.

(2) To 0.5 g of the powder add 10 ml of ethanol, stoppered tightly, macerate for 20 minutes with frequent shaking, filter, use the filtrate as the test solution. Dissolve cinnamic aldehyde CRS in ethanol to produce a solution containing 1 μl per ml as the reference solution. Carry out the method for thin layer chromatography *<0502>*, using silica gel G as the coating substance and a mixture of petroleum ether (60-90℃) and ethyl acetate (17:3) as the mobile phase. Apply separately to the plate 10-15 μl of the test solution and 2 μl of the reference solution. After developing and removal of the plate, dry in air, and spray with a 0.1% solution of 2,4-dinitrophenylhydrazine in ethanol. The orange-red spot in the chromatogram obtained with the test solution corresponds in position and colour to the spot in the chromatogram obtained with the reference solution.

(3) To 2 g of the powder add 10 ml of ether, macerate for 30 minutes with frequent shaking, and filter. Evaporate the filtrate to dryness, and dissolve the residue in 1 ml

of chloroform as the test solution. Prepare a solution with 2 g of Cinnamomi Ramulus reference drug in the same manner as the reference drug solution. Carry out the method for thin layer chromatography <*0502*>, using silica gel G as the coating substance and a mixture of petroleum ether (60- 90°C) and ethyl acetate (17:3) as the mobile·phase. Apply in strips separately 15 µl of each of the two solutions to the plate. After developing and removal of the plate, dry in air, spray with sulfuric acid-vanillin TS and heat at 105°C to the strips clear. The strips in the chromatogram obtained with the test solution correspond in position and colour to the strips in the chromatogram obtained with the reference drug solution.

Water Not more than 12.0 per cent <*0832, method 4*>.

Total ash Not more than 3.0 per cent <*2302*>.

Sulfur dioxide residue Not more than 150mg/kg<2331>.

Extractives Carry out the method for determination of ethanol-soluble extractives <*2201 the hot extraction method*>, using ethanol as the solvent, not less than 6.0 per cent.

Assay Carry out the method for high performance liquid chromatography <*0512*>.

Chromatographic system and system suitability Use octadecylsilane bonded silica gel as the stationary phase and a mixture of acetonitrile and water (32:68) as the mobile phase. As detector a spectrophotometer set at 290 nm. The number of theoretical plates of the column is not less than 3000, calculated with reference to the peak of cinnamaldehyde.

Reference solution Dissolve a quantity of cinnamic aldehyde CRS, weighed accurately, in methanol to produce a solution containing 10 µg per ml.

Test solution Weigh accurately 0.5 g of the powder (through No. 4 sieve) to a stoppered conical flask, add accurately 25 ml of methanol, weigh, ultrasonicate (power 250 W, frequency 40 kHz) for 30 minutes, allow to cool, weigh again, replenish the loss of the weight with methanol, mix well, and filter. Accurately measure 1 ml of the successive filtrate to a 25 ml volumetric flask, dilute with methanol to volume, mix well.

Procedure Inject accurately 10 µl of each of the reference solution and the test solution, respectively, into the column, and calculate the content.

It contains not less than 1.0 per cent of cinnamic aldehyde (C_9H_8O), calculated with reference to the dried drug.

1.7.9 Carthami Flos
（红花，Honghua）
Safflower

Description The drug consisting of tubular flowers without ovaries, 1-2 cm long. Externally reddish-yellow or red. Corolla tubes slender, 5-lobed at the apex, the lobes narrowly belt-shaped, 5-8 mm long. Stamens 5, anthers aggregated to a tube, yellowish-white. Stigma long cylindrical, slightly 2-cleft. Texture pliable. Odour, slightly aromatic; taste, slightly bitter.
Identification (l) Powder: Orange-yellow. The fragments of corolla, filament and stigma frequently visible. Long tubular secretory cells present, generally accompanied by vessels, up to 66 µm in diameter, containing yellowish-brown to reddish-brown secretion. Outer walls of terminal epidermal cells of corolla lobes projecting to be tomentellate. Upper epidermal cells of stigma and style differentiated into conical unicellular hairs, acuminate or slightly obtuse at the apex. Pollen grains subrounded, elliptical or olivary, up to 60 µm in diameter, with 3 germinal pores, exine dentate-spinose. Prisms of calcium oxalate occurring in paren-chymatous cells, 2-6 µm in diameter.
(2) To 0.5 g of the powder add 5 ml of 80% acetone solution, stopper tightly, shake constantly for 15 minutes and allow to stand; the supernatant liquid is used as the test solution. Prepare the reference drug solution in the same manner using 0.5 g of Carthami Flos reference drug. Carry out the method for thin layer chromatography <*0502*>, using silica gel H as the coating substance and a mixture of ethyl acetate, formic acid, water and methanol (7:2:3:0.4) as the mobile phase. Apply separately to the plate 5 µl of each of the solutions. After developing and removal of the plate, dry in air. The spots in the chromatogram obtained with the test solution correspond in position and colour to the spots obtained with the reference drug solution.
Foreign matter Not more than 2 per cent <*2301*>.
Water Not more than 13.0 per cent <*0832, method 2*>.
Total ash Not more than 15.0 per cent <*2302*>.
Acid-insoluble ash Not more than 5.0 per cent <*2302*>.
Sulfur dioxide residue Not more than 150mg/kg<2331>.
Absorbance *Red pigment* Dry the drug in a desiccator over silica gel for 24 hours and grind into a fine powder. Macerate warmly about 0.25 g of the fine powder, accurately weighed, with 50 ml of 80%, acetone solution in a conical flask at 50°C on a water bath for 90 minutes, cool, filter through a sintered glass funnel (No.3) into a 100 ml volumetric flask, wash the residue with 25 ml of 80% acetone solution in portions. Transfer the washings into the volumetric flask, add 80%, acetone solution to the volume, and mix well. Carry out the method for ultraviolet-visible spectrophotometry <*0401*>, measure the absorbance of the solution at 518 nm, not less than 0.20.

Extractives Carry out the method for determination of water-soluble extractives <*2201, the cold maceration method*>, not less than 30.0 per cent

Assay ***Hydroxysaf flor yellow A*** Carry out the method for high performance liquid chromatography <0512>.

Chromatographic system and system suitability Use octadecylsilane bonded silica gel as the stationary phase and a mixture of methanol, acetonitrile and 0.7% phosphate acid solution (26:2:72) as the mobile phase. As detector a spectrophotometer set at 403 nm. The number of theoretical plates of the column is not less than 3000, calculated with reference to the peak of hydroxysafflor yellow A.

Reference solution Dissolve a quantity of hydroxysafflor yellow A CRS, accurately weighed, in 25% methanol to produce a solution of 0.13 mg per ml.

Test solution Weigh accurately 0.4 g of the powder (through No. 3 sieve) in a conical flask with stopper, add 50 ml of 25% methanol, accurately measured, weigh accurately and ultrasonicate (power 300 W, frequency 50 kHz) for 40 minutes, allow it to cool and weigh again, replenish the loss of the weight with 25% methanol, and mix well. Filter and use the successive filtrate as the test solution

Procedure Inject accurately 10 µl of each of the two solutions into the column, and calculate the content.

It contains not less than 1.0 per cent of hydroxysafflor yellow A ($C_{27}H_{32}O_{16}$), calculated with reference to the dried drug.

Kaempferol Carry out the method for high performance liquid chromatography <*0512*>.

Chromatographic system and system suitability Use octadecylsilane bonded silica gel as the stationary phase and a mixture of methanol and 0.4% phosphate acid solution (52:48) as the mobile phase. As detector a spectrophotometer set at 367 nm. The number of theoretical plates of the column is not less than 3000, calculated with reference to the peak of kaempferol

Reference solution Dissolve a quantity of kaempferol CRS, accurately weighed, in methanol to produce a solution of 9 µg per ml.

Test solution Weigh accurately 0.5 g of the powder (through No. 3 sieve) in a conical flask with stopper, add 25 ml of methanol, accurately measured, weigh accurately and heat under reflux for 30 minutes, allow it to cool and weigh again, replenish the loss of the solvent with methanol, mix well and filter. Measure accurately 15 ml of the successive filtrate to a flat bottom flask, add 5 ml of hydrochloric acid (15→37), mix well and heat to hydrolysis on a water bath for 30 minutes, cool immediately, transfer to a 25 ml volumetric flask, dilute with methanol to volume and mix well.

Procedure Inject accurately 10 µl of each of the two solutions into the column, and calculate the content. It contains not less than 0.050 per cent of kaempferol ($C_{15}H_{10}O_6$), calculated with reference to the dried drug.

1.7.10 Periplocae Cortex
（香加皮，Xiangjiapi）
Chinese Silkvine Root-bark

Description Quilled or channelled, a few pieced irregularly, 3-10 cm long, 1-2 cm in diameter, 2-4 mm thick. Outer surface greyish-brown or yellowish-brown, cork soft and loose, often scaly, easily exfoliated; inner surface pale yellow or pale yellowish-brown, relatively smooth, with fine longitudinal striations. Texture light and fragile, easily broken, fracture uneven, yellowish-white. Odour, characteristic and aromatic; taste, bitter.
Identification (1) Powder: Pale brown. Prisms of calcium oxalate rare, 9-20 μm in diameter. Stone cells rectangular or subpolygonal, 24-70 μm in diameter. Laticiferous tubes containing colourless oily granules. Cork cells brownish-yellow. Starch granules numerous, simple granules subrounded or oblong, 3-11 μm in diameter; compound granules of 2-6 components.
(2) Distill 10 g of the powder with 150 ml of water in a 250 ml flask, the odour of distillate characteristic and aromatic. Transfer 10 ml of the distillate to two test tubes. To one test tube add 1 drop of 1% solution of ferric chloride, a brownish-red colour is produced. To another tube add 5 ml of saturated solution of hydrazine sulfate and a few crystals of sodium acetate, heat slightly and cool, a pale yellowish-green precipitate is produced, examine under ultraviolet light at 365 nm, showing a strong yellow fluorescence.
(3) Heat under reflux 1 g of the powder with 10 ml of ethanol for 1 hour, filter. Transfer the filtrate to a 25 ml volumetric flask, and dilute with ethanol to volume, shake well Transfer 1 ml of the ethanol solution to a 20 ml volumetric flask, dilute with ethanol to volume, shake well. Carry out the method for ultraviolet- visible spectrophotometry <*0401*>, the absorption exhibits a maximum at 278 nm.
(4) To 2 g of the powder add 30 ml of methanol, heat under reflux for 1 hour, and filter. Evaporate the filtrate to dryness, and dissolve the residue in 2 ml of methanol as the test solution. Dissolve 4-methoxy salicylic aldehyde CRS in methanol to prepare a solution containing 1 mg per ml as the reference solution. Carry out the method for thin layer chromatography <*0502*>, using silica gel G as the coating substance and a mixture of petroleum ether (60-90°C), ethyl acetate and glacial acetic acid (20:3:0.5) as the mobile phase. Apply separately to the plate 2 μl of each of the above two solutions. After developing and removal of the plate, dry in air. Spray with dinitrophenylhydrazine TS. The spot in the chromatogram obtained with the test solution corresponds in position and colour to the spot in the chromatogram obtained with the reference solution.
Water Not more than 13.0 per cent <*0832, method 2*>.
Total ash Not more than 10.0 per cent <*2302*>.
Acid-insoluble ash Not more than 4.0 per cent <*2302*>.

中国河北省石家庄市高新技术开发区天山大街 238 号 PC：050035
NO. 238, Tianshan Avenue, Hi-Tech Development Zone, Shijiazhuang, Hebei, China
TEL: 0086 311 85901719 FAX: 0086 311 85901719 http://www.yiling.cn

Extractives Carry out the method for determination of ethanol-soluble extractives <*2201, the hot extraction method*>, using dilute ethanol as the solvent, not less than 20.0 per cent.

Assay Carry out the method for high performance liquid chromatography <*0512*>.

Chromatographic system and system suitability Use octadecylsilane bonded silica gel as the stationary phase and a mixture of methanol, water and acetic acid (70:30:2) as the mobile phase. As detector a spectrophotometer set at 278 nm. The number of theoretical plates of the column is not less than 1000, calculated with reference to the peak of 4-methoxyl salicylic aldehyde.

Internal standard solution Dissolve a quantity of *n*-butyl-*p*- hydroxybenzoate, accurately weighed, in 60% methanol to produce a solution containing 6 mg per ml as the internal standard solution.

Procedure Weigh accurately a quantity of 4-methoxyl salicylic aldehyde CRS in a brown volumetric flask, dissolve and dilute with 60% methanol to produce a solution containing 1 mg per ml. Accurately transfer 4 ml of the above solution and 2 ml of the internal standard solution to a 25 ml volumetric flask, dilute with 60% methanol to volume, and mix well. Inject accurately 20 µl into the column and plot the chromatogram. Accurately weigh 250-500 mg of the coarse powder, dried at 60°C for 4 hours, in a 50 ml flask, add 15 ml of 60% methanol, heat under reflux for 1.5 hours, and filter. Transfer the filtrate to a 25 ml volumetric flask, wash the container with a small quantity of 60% methanol, filter the washings to the same flask, add accurately 2 ml of the internal standard solution, dilute with 60% methanol to volume, and mix well. Filter and use the successive filtrate as the test solution. Inject 20 µl into the column, measure the peak area and calculate the content with corrected internal standard method. It contains not less than 0.20 per cent of 4-methoxyl salicylic aldehyde ($C_8H_8O_3$), calculated on the basis dried at 60°C for 4 hours.

1.7.11 Citri Reticulatae Pericarpium
（陈皮，Chen pi）
Dried Tangerine Peel

Description *Chenpi* Often peeled in several lobes connecting at the base, or in irregular slices, 1-4 mm thick. Outer surface orange-red to reddish-brown, with fine wrinkles and concave dotted cavity, inner surface pale yellowish-white, rough, bearing yellowish-white or yellowish-brown vein-like vascular bundles. Texture slightly hard and fragile. Odour, aromatic; taste, pungent and bitter.
Guang Chenpi Often in three lobes connected at the base, regular in shape and even in thickness, about 1 mm thick. The dotted oil cavity relatively large, transparent when observed against light. Texture slightly soft.
Identification (1) Powder: Yellowish-white to yellowish-brown. Parenchymatous cells of mesocarp numerous, cells irregular, with unevenly thickened walls, sometimes beaded. Epidermal cells of pericarp polygonal, subsquare or rectangular in surface view, anticlinal walls slightly thickened, stomata subrounded, 18-26 μm in diameter, subsidiary cells indistinct; covered with cuticle in lateral view and the outer radial wall thickened. Numerous prisms of calcium oxalate containing in parenchymatous cells of the mesocarp, polyhedral, rhombic or biconical, 3-34 μm in diameter, 5-53 μm long; sometimes two parallel polyhedral crystals or 3-5 prisms occurring in one cell. Crystals of hesperidin mostly present in parenchymatous cells, yellow or colourless, in rounded or amorphous masses, some crystals with radial striations. Spiral, pitted and reticulated vessels and tracheids small.
(2) To 0.3 g of the powder add 10 ml of methanol, heat under reflux for 20 minutes and filter. Concentrate 5 ml of the filtrate to about 1 ml as the test solution. Dissolve hesperidin CRS in methanol to produce a saturated solution as the reference solution. Carry out the method for thin layer chromatography *<0502>*, using silica gel G mixed with 0.5% solution of sodium hydroxide as the coating substance and a mixture of ethyl acetate-methanol- water (100:17:13) as the mobile phase. Apply separately 2 μl of each of the two solutions to the plate. After developing about 3 cm and removal of the plate, dry in air, and then use the upper layer of toluene-ethyl acetate-formic acid-water (20:10:1:1) as the mobile phase, after developing 8 cm and removal of the plate, dry in air. Spray with a solution of aluminum chloride TS. Examine under ultraviolet light at 365 nm, the fluorescent spot in the chroma-togram obtained with the test solution corresponds in position and colour to the spot in the chromagrain obtained with the reference solution.
Water Carry out the method for the determination of water *<0832, method 4>*, not more than 13.0 per cent.
Aflatoxins Carry out the method for determination of aflatoxins *<2351>*.

Weigh accurately 5 g of the powder (through No. 2 sieve), add 3 g of sodium chloride, prepare it in the same manner as described under the method for aflatoxin, test and caiculate the content.

It contains not more than 5 µg/kg of aflatoxin B_1 and not more than 10 µg/kg of the sum of aflatoxin G_2, aflatoxin G_1, aflatoxin B_2 and aflatoxin B_1.

Sulfur dioxide residue Not more than 150mg/kg<2331>.

Assay Carry out the method for high performance liquid chromatography *<0512>*.

Chromatographic system and system suitability Use octadecylsilane bonded silica gel as the stationary phase and a mixture of methanol, acetic acid and water (35:4:61) as the mobile phase. As detector a spectrophotometer set at 283 nm. The number of theoretic plates of the column is not less than 2000, calculated with reference to the peak of hesperidin.

Reference solution Dissolve a quantity of hesperidin, accurately weighed, in methanol to produce a solution containing 0.4 mg per ml and mix well.

Test solution Weigh accurately 1 g of the coarse powder in a Soxhlet' s extractor, add 80 ml of petroleum ether. (60-90°C), heat under reflux for 2-3 hours. Discard the petroleum ether extract and evaporate the residue to dryness, add 80 ml of methanol, heat under reflux until the extract solution is colourless, cool and filter. Transfer the filtrate to a 100 ml of volumetric flask, wash the container with a small quantity of methanol for several times, filter the washings to the same flask, dilute with methanol to volume, and mix well.

Procedure Inject accurately 5 µl of each of the reference solution and the test solution, into the column, and calculate the content.

It contains not less than 3.5 per cent of hesperidin ($C_{28}H_{34}O_{15}$), calculated with reference to the dried drug.

中国河北省石家庄市高新技术开发区天山大街 238 号 PC： 050035
No.238 Tianshan Street, High-Tech Area, Shijiazhuang Hebei Province, China.
TEL: 0086 311 85901719 FAX: 0086 311 85901719 http://www.yiling.cn

1.8 Reference Standards or Materials

Ingredients	Reference drug	Supplier	Batch No.
Astragali Radix	Astragali Radix reference drug	National institutes for food and drug control	120974-201813
	Astragaloside IV	National institutes for food and drug control	110781-202118
	Calycosin-7-O-β-D-glycoside	National institutes for food and drug control	111920-201907
Ginseng Radix et Rhizoma	Ginseng Radix et Rhizoma reference drug	National institutes for food and drug control	120917-201712
	ginsenosides Rb_1	National institutes for food and drug control	110704-202129
	ginsenosides Re	National institutes for food and drug control	110754-202028
	ginsenosides Rf	National institutes for food and drug control	111719-201806
	ginsenosides Rg_1	National institutes for food and drug control	110703-202034
Aconiti Lateralis Radix Praeparata	benzoylmesaconine	National institutes for food and drug control	111794-202006
	benzoylaconitine	National institutes for food and drug control	111795-201805
	benzoylhypaconitine	National institutes for food and drug control	111796-201906
	mesaconine	National institutes for food and drug control	110799-201608
	hypaconitine	National institutes for food and drug control	110798-201609
	aconitine	PCL	A1907010
Salviae Miltiorrhizae Radix et Rhizoma	Salviae Miltiorrhizae Radix et Rhizoma reference drug	National institutes for food and drug control	120923-201816
	tanshinone IIA	National institutes for food and drug control	110766-202022
	salvianolic acid B	National institutes for food and drug control	111562-201917
Descurainiae Semen	quercetin-3-O-β-D-glucose-7-O-β-D gentiobioside	National institutes for food and drug control	111854-201905

中国河北省石家庄市高新技术开发区天山大街 238 号 PC：050035
No.238 Tianshan Street, High-Tech Area, Shijiazhuang Hebei Province, China.
TEL: 0086 311 85901719 FAX: 0086 311 85901719 http://www.yiling.cn

Alismatis Rhizoma	Alismatis Rhizoma reference drug	National institutes for food and drug control	121081-201807
	23-acetate alisol B	National institutes for food and drug control	111846-202006
	23-acetate alisol C	National institutes for food and drug control	112062-202102
Cinnamomi Ramulus	cinnamic aldehyde	National institutes for food and drug control	110710-202022
	Cinnamomi Ramulus reference drug	National institutes for food and drug control	121191-201906
Carthami Flos	Carthami Flos reference drug	National institutes for food and drug control	120907-201713
	Hydroxysafflor yellow A	National institutes for food and drug control	111637-201810
	Kaempferol	National institutes for food and drug control	110861-202013
Periplocae Cortex	4-methoxy salicylic aldehyde	National institutes for food and drug control	110790-201605
Citri Reticulatae Pericarpium	hesperidin	National institutes for food and drug control	110721-202019

中国河北省石家庄市高新技术开发区天山大街 238 号 PC：050035
No.238 Tianshan Street, High-Tech Area, Shijiazhuang Hebei Province, China.
TEL: 0086 311 85901719 FAX: 0086 311 85901719 http://www.yiling.cn

1.9 Storage conditions

All the drug substance packaged in polypropylene woven bags, stored in specified condition described as the storage of each ingredient in Chinese Pharmacopoeia 2020, volume I.

Ingredients	Storage
Astragali Radix	Preserve in a ventilated and dry place, and protect from moisture and moth.
Ginseng Radix et Rhizoma	Preserve in a well closed container, store in a cool and dry place, and protect from moth.
Aconiti Lateralis Radix Praeparata (heishunpian)	Preserve in a dry place, protected from moisture.
Salviae Miltiorrhizae Radix et Rhizoma	Preserve in a dry place.
Descurainiae Semen	Preserve in a dry place.
Alismatis Rhizoma	Preserve in a dry place, and protect from moth.
Polygonati Odorati Rhizoma	Preserve in a ventilated and dry place, and protect from mould and moth.
Cinnamomi Ramulus	Preserve in a cool and dry place.
Carthami Flos	Preserve in a cool and dry place, and protect from " moisture and moth.
Periplocae Cortex	Preserve in a cool and dry place.
Citri Reticulatae Pericarpium	Preserve in a cool and dry place, and protect from mold and moth.

QILI QIANGXIN CAPSULES
0.3 G/CAPSULE

CMC INFORMATION

2. DRUG PRODUCT

Shijiazhuang Yiling Pharmaceutical Co., Ltd.

December, 2022

中国河北省石家庄市高新技术开发区天山大街 238 号 PC：050035
No.238 Tianshan Street, High-Tech Area, Shijiazhuang Hebei Province, China.
TEL: 0086 311 85901719 FAX: 0086 311 85901719 http://www.yiling.cn

2.1 Description and composition of the herbal product

Components of Qili Qiangxin Capsules

PRODUCT NAME: Qili Qiangxin Capsules	PRODUCT TYPE: Hard capsule
DESCRIPTION: Hard capsules, containing dark brownish to black-brownish granules; taste, bitter.	
PACKING MATERIALS: Gelatin hollow capsule, compound membrane, PTP aluminum foil, parmaceutical polyvinyl chloride PVC hard tablet, PVC film, big carton, packaging box.	
Stength: 300 mg/ Capsule	
Functions and Indications: Tonify *qi* and warm *Yang*, activate blood and unblock collaterals, induce diuresis to alleviate edema, it is used for mild and moderate congestive heart failure caused by coronary heart disease and hypertension, indicated by *yang* deficiency, blood blocking and liquid stagnation; with the symptoms as flusteredness and short breath, more seriously when moving; unable to lie on back during sleep; legs edema; tireness and asthenia; less urine; mouth and lips hematocyanosis; chilly and cold in limbs, spit watery white phlegm.	

No.	Ingredients	Specification
1.	Astragali Radix	Chinese Pharmacopeia 2020, volume I
2.	Ginseng Radix et Rhizoma	Chinese Pharmacopeia 2020, volume I
3.	Aconiti Lateralis Radix Praeparata (heishunpian)	Chinese Pharmacopeia 2020, volume I
4.	Salviae Miltiorrhizae Radix et Rhizoma	Chinese Pharmacopeia 2020, volume I
5.	Descurainiae Semen	Chinese Pharmacopeia 2020, volume I
6.	Alismatis Rhizoma	Chinese Pharmacopeia 2020, volume I
7.	Polygonati Odorati Rhizoma	Chinese Pharmacopeia 2020, volume I
8.	Cinnamomi Ramulus	Chinese Pharmacopeia 2020, volume I
9.	Carthami Flos	Chinese Pharmacopeia 2020, volume I
10.	Periplocae Cortex	Chinese Pharmacopeia 2020, volume I

中国河北省石家庄市高新技术开发区天山大街 238 号 PC：050035
No.238 Tianshan Street, High-Tech Area, Shijiazhuang Hebei Province, China.
TEL: 0086 311 85901719 FAX: 0086 311 85901719 http://www.yiling.cn

11.	Citri Reticulatae Pericarpium	Chinese Pharmacopeia 2020, volume I
12.	Dextrin	Chinese Pharmacopeia 2020, volume IV
13.	1# Vacant Gelatin Capsules	Chinese Pharmacopeia 2020, volume IV

2.2 Formulation development

1. Source of Formula

The formula of Qili Qiangxin Capsules is invented by Professor Wu Yiling, from Hebei Yiling Medicine Research Institute. He uses Vein sickness theory to treat chronic congestive heart failure. According to years of clinical experiences, he invented the pure Chinese medicine compound prescription. It is consist of Astragali Radix , Ginseng Radix et Rhizoma, Aconiti Lateralis Radix Praeparata, Salviae Miltiorrhizae Radix et Rhizoma, Descurainiae Semen, Alismatis Rhizoma, Polygonati Odorati Rhizoma, Cinnamomi Ramulus , Carthami Flos, Periplocae Cortex and Citri Reticulatae Pericarpium. It is beneficial to *Yang qi*, the blood circulation. It is advantageous in the detumescence. It is suitable for mild and moderate chronic congestive heart failure (lack of Yang Qi, blood stasis and water stop syndrome).

2. Ingredients

Astragali Radix, Ginseng Radix et Rhizoma, Aconiti Lateralis Radix Praeparata, Salviae Miltiorrhizae Radix et Rhizoma, Descurainiae Semen, Alismatis Rhizoma, Polygonati Odorati Rhizoma, Cinnamomi Ramulus, Carthami Flos, Periplocae Cortex, Citri Reticulatae Pericarpium. Excipient: Dextrin.

3. Functions and Indications

Tonify *qi* and warm *Yang*, activate blood and unblock collaterals, induce diuresis to alleviate edema, it is used for mild and moderate congestive heart failure caused by coronary heart disease and hypertension, indicated by *yang* deficiency, blood blocking and liquid stagnation; with the symptoms as flusteredness and short breath, more seriously when moving; unable to lie on back during sleep; legs edema; tireness and asthenia; less urine; mouth and lips hematocyanosis; chilly and cold in limbs, spit watery white phlegm.

4. Administration and Dosage

Confidential information
机密信息，注意保密

For oral administration, 4 capsules once, 3 times daily.

5. Basis of Formulation

Qi Li Qiang Xin Capsules is a newly developed Chinese herbal medicine for chronic congestive heart failure.

Chronic congestive heart failure refers to cardiac primary injury due to multiple reasons and late-stage turnover of heart function compensation. It is clinically characterized by abnormal cardiac functioning, and decreased exercise tolerance and neuroendocrine activation It always develops from coronary artery disease, hypertension, and cardiomyopathy and can be life threatening. Chronic congestive heart failure is located in the heart and characterized by weakness in myocardial contraction in modern medicine. However, TCM holds that it involves general collaterals and multiple zang-fu organs. Heart-qi deficiency is the underlying cause stagnant blood obstructing the collaterals is the key link, and masses in the heart collaterals due to dampness, stasis, and water retention are results of the progression. This matches with the concept in modern medicine that earlier neuroendocrine system activation may cause hemodynamic changes and subsequently lead to ventricle reconstruction and eventually heart failure.

The treatment principle for chronic congestive heart failure should be to supplement qi and warm yang (for the underlying cause) and resolve stasis and clear the collaterals (for the key pathologic link).

Among the components of the product, Huang Qi (Astragali Radix) and Fu Zi (Aconiti Lateralis Radix Praeparata) act as monarch herbs to supplement qi promote diuresis, and warm yang for heart-qi (yang) deficiency. Ren Shen (Ginseng Radix et Rhizoma), Dan Shen (Salviae Miltiorrhizae Radix et Rhizoma), and Ting Li Zi (Descurainiae Semen) act as minister herbs to tonify qi, clear the collaterals, regulate and circulate blood, clear the lungs, and promote diuresis for three major pathological changes: qi (yang) deficiency stasis obstruction of the collaterals, and water-retention. Hong Hua (Carthami Flos), Ze Xie (Alismatis Rhizoma) Xiang Jia Pi (Periplocae Cortex), Yu Zhu (Polygonati Odorati Rhizoma), and Chen Pi (Citri Reticulatae Pericarpium) act as assistant herbs to circulate blood resolve stasis, promote diuresis to remove edema strengthen the heart and promote diuresis, nourish heart-yin and protect anti-pathogenic qi, and regulate qi activity. Gui Zhi (Cinnamomi Ramulus) acts as a guide herb to warm yang, clear the collaterals, and induce other herbs into the collaterals.

In summary Qi Li Qiang Xin Capsules acts to strengthen the heart by selecting herbs to supplement qi and warm yang, resolve blood stasis in the collaterals by selecting herbs to circulate blood and clear the collaterals, and promote diuresis to remove edema. Pharmacological study has shown that it can significantly improve the hemodynamic markers in animal models with heart failure, and that it can improve heart failure symptoms with its actions of heart-tonification, diuresis, and vasodilation. In addition,

Confidential information
机密信息，注意保密

it can significantly reduce angiotensin II, inhibit the increase of aldosterone contents, and improve the ventricular wall thickness and cardiac index. This suggests that it can improve the biological foundation of the structure and function of people with chronic congestive heart failure. In summary, this compound product can treat the condition comprehensively with multiple means on multiple targets through multiple links. This product is generally indicated for mild or moderate chronic heart failure

Confidential information
机密信息，注意保密

2.3 Manufacturing Process

All the medicinal materials used in this medicine are the first-grade products, and have passed inspections, then the following dressing operations are performed:
Name of products: Qili Qiangxin Capsules.
Manufacturer: Shijiazhuang Yiling Pharmaceutical Co., Ltd.
Specification: ChP 2020
Batch quantity: 2,400,000 capsules per batch

Stage 1. Ethanol extract

Putting Astragali Radix, Ginseng Radix et Rhizoma, Descurainiae Semen, Alismatis Rhizoma, Periplocae Cortex in multifunctional extractive tank, extract with 8 times of 70% ethanol for 3 hours, filter and collect the ethanol extract solution, add 8 times of 70% ethanol for 2 hours, filter and collect the ethanol extract solution, combine the two ethanol extract solution. Vacuum evaporate to recover ethanol at 40~90°C/0.04MPa~0.1MPa until there is no odor of ethanol to obtain Qili Qiangxin Ethanol Extract Paste (1.25-1.30 g/ml at 60°C).

Stage 2. Distilling extract of volatile oil

Putting Cinnamomi Ramulus and Citri Reticulatae Pericarpium in multifunctional extractive tank, add 6 times of water, extract volatile oil for 6 hours, collect the volatile oil for later use, collect the water extract solution. Add 6 times of water to the residues of decoction, extract for 1 hour, filter and collect the water extract solution, combine the water solution for later use.

Stage 3. Water extract

Putting Aconiti Lateralis Radix Praeparata (heishunpian), Salviae Miltiorrhizae Radix et Rhizoma, Polygonati Odorati Rhizoma, Carthami Flos in multifunctional extractive tank. Extract with 9 times of water for 2 hours, filter and collect the water extract solution, add 9 times of water in the tank extract for 2 hours, filter and collect the water extract solution, combine the two water extract solution, filter for later use.

Stage 4 Concentration and Ethanol precipitation

Combine the water solution obtained in stage 2 and stage 3 into double-effect evaporator, evaporate solvent at 55-90°C/0.03-0.09MPa for the first time and 40-80°C/0.05-0.1MPa to a kind of clear paste with a relative density of 1.25-1.30 g/ml (60°C).
Add 95% ethanol solution to the clear paste until the concentration down to 70% ethanol solution, stirring, incubated at 0~4°C for 24 hours, the supernatant was filtered. Put the filtrate into the vacuum concentration tank, vacuum concentration (temperature 40 ~ 90 °C, vacuum degree 0.04MPa ~ 0.1MPa) until the relative density of 1.25 ~ 1.30 g/ml (60°C), Qili Qiangxin Ethanol Precipitation Paste is obtained.

Stage 5 Drying

Combine the Qili Qiangxin Ethanol Extract Paste in stage 1 and Qili Qiangxin Ethanol Precipitation Paste in stage 4 in a material tank, stirring well, put the materials into belt vacuum drying machine, drying at a 100-125°C/>90KPa to obtain Qili Qiangxin Dry Extract.

Stage 6. Granulating and mixing

Putting Qili Qiangxin Dry Extract in pellet machine, granulating by using 1# sieve. Mixing Qili Qiangxin Dry Extract and Dextrin in wet mixed pellet machine, adding volatile oil while stirring for about 5-10 minutes, swing granulation through screen, then transfer to the mixing tank.

Mixing with speed of 8 rpm for 20 minutes, tightly seal and incubate for additional 30 minutes to obtain Qili Qiangxin Granules. Keep the granules in plastic bags, tightly seal.

Stage 7. Capsule filling

Fill the granules into red 1# empty capsule shells with auto capsule filling machine.

Filling speed:

Auto capsule filling machine

In-house control: Average filling weight: 0.3 g ± 7%

Good capsules are stored directly in suitable bins, with mark on them.

Record weight deviation on Batch Manufacturing Record.

Stage 8. Packing

Carry out the packing using Automatic Packing Line Machine.

The capsules are packed into blisters on the automatic lines (12 capsules/blister). Blisters are made of overprinted Aluminum/PVC Foils.

The machine for packing of the capsules into the blisters is placed in the separated room.

Automatic Packing Line Machines is equipped in the control system of filling of the blisters, presence of the blisters in the individual carton box and presence of the leaflet in the individual carton box.

Random check samples were taken to visually inspect labeling / insert leaflet / blisters and capsules count.

Stage 9. Quality control of finish product

Send the finished product to Q.C laboratory and ask for final analysis.

Stage 10. Warehouse entry

If the samples are approved by Q.C Lab, whole batch of finished products will be sent to the warehouse.

石家庄以岭药业股份有限公司
YILING SHIJIAZHUANGYILING PHARMACEUTICAL CO.,LTD
中国河北省石家庄市高新技术开发区天山大街 238 号 PC: 050035
No.238 Tianshan Street, High-Tech Area, Shijiazhuang Hebei Province, China.
TEL: 0086 311 85901719 FAX: 0086 311 85901719 http://www.yiling.cn

Processing Flow Chart of Qili Qiangxin Capsules

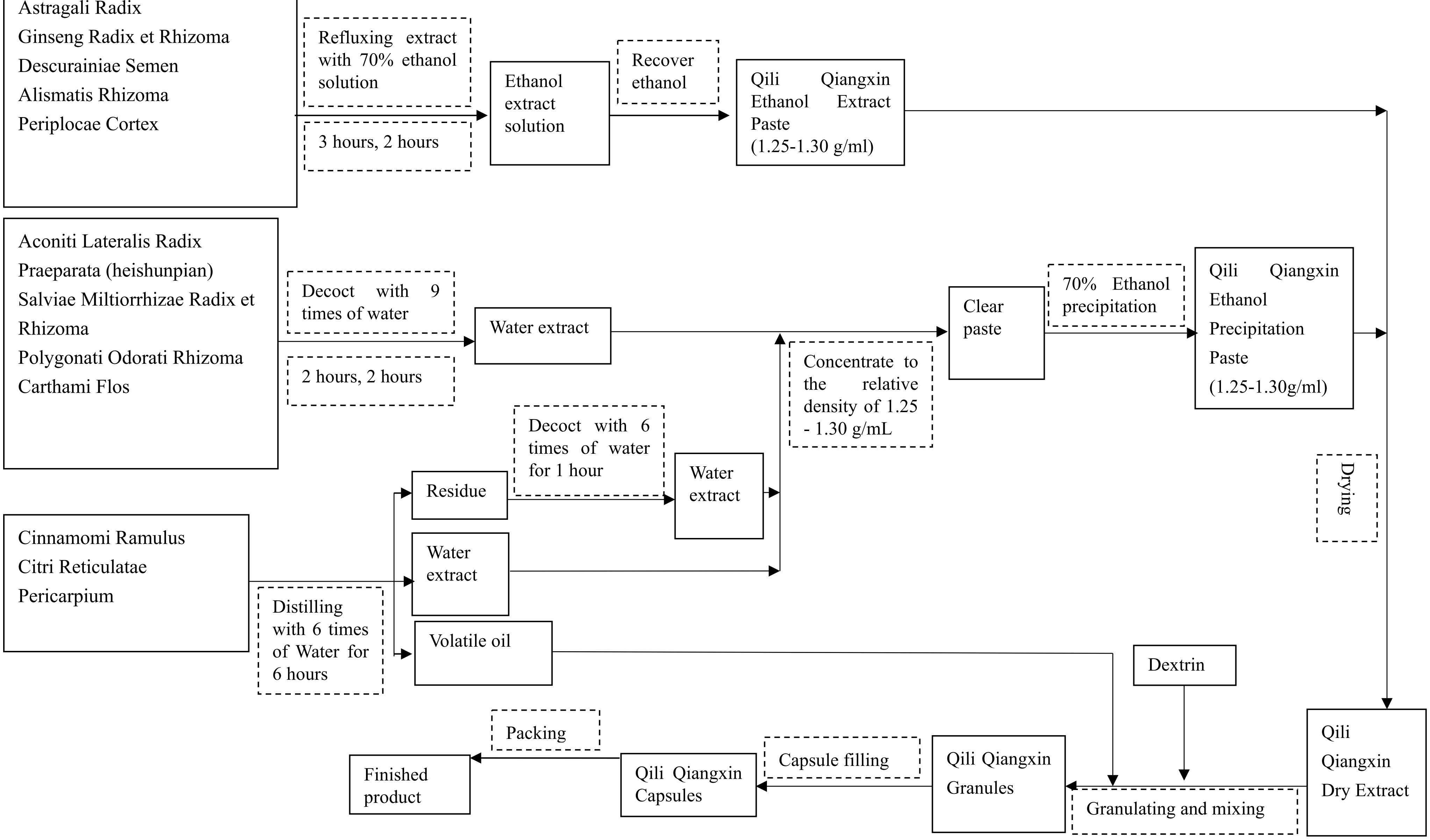

2.4 Specification (s)

Product Name: Qili Qiangxin Capsules
Strength: 0.3g/capsule
Package size: Aluminium-plastic package, 36 capsules/box.
Storage condition: Preserve in tightly closed containers.
Shelf-life: 30 months
Reference standard: Chinese pharmacopeia 2020, volume I (Appendix 12)

Inspection Items		Standard criteria
**Description**		It should be hard capsules, containing dark brownish to black-brownish granules; taste, bitter.
**Identification**		
(1) HPLC		The peaks in the chromatogram obtained with the test solution should correspond in the retention time to the peaks in the chromatogram obtained with the reference solution.
(2) (3) (4) (5) TLC		TLC of Salviae Miltiorrhizae Rhizoma et Radix reference drug, Periplocae Cortex reference drug, cinnamaldehyde CRS, hesperidin CRS
**Other requirements**		
Filling variation		Should be within ±10.0%.
Water determination		Not more than 9.0%
Disintegration test		Should disintegrate within 30 minutes
Limit of aconitine		The spot in the chromatogram obtained with the test solution is not bigger than the spot in the chromatogram obtained with the reference solution, or there is no spot reveals in the chromotogram obtained with the test solution.
Chromium content in capsule shell		Not more than 2ppm.
**Assay**		It contains not less than 0.12 mg of astragaloside IV ($C_{41}H_{68}O_{14}$) per capsule, referred to Astragals Radix.
Microbial limit	Total Aerobic Microbial Count	Not more than 10^3cfu/g.
	Total combined Yeast and Mold Count	Not more than 10^2cfu/g.
	E.Coli.	Absent/g.

中国河北省石家庄市高新技术开发区天山大街 238 号 PC：050035
No.238 Tianshan Street, High-Tech Area, Shijiazhuang Hebei Province, China.
TEL: 0086 311 85901719 FAX: 0086 311 85901719 http://www.yiling.cn

2.5 Batch analyses

Inspection Item	Standard criteria	B2102002 Testing Data	B2102003 Testing Data	B2102004 Testing Data
**Description**	It should be hard capsules, containing dark brownish to black-brownish granules; taste, bitter.	Hard capsules, containing dark brownish to black-brownish granules; taste, bitter.	Hard capsules, containing dark brownish to black-brownish granules; taste, bitter.	Hard capsules, containing dark brownish to black-brownish granules; taste, bitter.
**(1)** HPLC	The peaks in the chromatogram obtained with the test solution should correspond in the retention time to the peaks in the chromatogram obtained with the reference solution.	The peaks in the test solution correspond in the retention time to the peaks in the chromatogram obtained with the reference solution.	The peaks in the test solution correspond in the retention time to the peaks in the chromatogram obtained with the reference solution.	The peaks in the test solution correspond in the retention time to the peaks in the chromatogram obtained with the reference solution.
(2) (3) (4) (5) TLC	TLC of Salviae Miltiorrhizae Rhizoma et Radix reference drug, Periplocae Cortex reference drug, cinnamaldehyde CRS, hesperidin CRS	Positive spots correspond to the chemical reference substance and reference drugs	Positive spots correspond to the chemical reference substance and reference drugs	Positive spots correspond to the chemical reference substance and reference drugs
Filling variation	Should be within ±10.0%.	Complies	Complies	Complies
Water determination	Not more than 9.0%	3.1%	4.3%	3.7%
Disintegration test	Should disintegrate within 30 minutes	9 minutes	9 minutes	9 minutes
Limit of aconitine	The spot in the chromatogram obtained with the test solution is not bigger than the spot in the chromatogram obtained with the	Complies	Complies	Complies

		reference solution, or there is no spot reveals in the chromotogram obtained with the test solution.			
Chromium content in capsule shell		Not more than 2ppm.	Complies	Complies	Complies
Microbial limit	TAMC	Not more than 10^3cfu/g.	< 100 cfu	< 100 cfu	< 100 cfu
	TYMC	Not more than 10^2cfu/g.	< 10 cfu	< 10 cfu	< 10 cfu
	E.Coli.	Absent/g.	Not Detected	Not Detected	Not Detected
**Assay**		It contains not less than 0.12 mg of astragaloside IV ($C_{41}H_{68}O_{14}$) per capsule, referred to Astragals Radix.	0.60 mg/capsule	0.62 mg/capsule	0.68 mg/capsule

石家庄以岭药业股份有限公司
YILING SHIJIAZHUANGYILING PHARMACEUTICAL CO.,LTD
中国河北省石家庄市高新技术开发区天山大街 238 号 PC: 050035
NO. 238, Tianshan Avenue, Hi-Tech Development Zone, Shijiazhuang, Hebei, China
TEL: 0086 311 85901719 FAX: 0086 311 85901719 http://www.yiling.cn

SHIJIAZHUANG YILING PHARMACEUTICAL CO., LTD

Certificate of Analysis for Finished Product

Report No. Cheng 045 (Bao) B2102002 L-QB-045-C00-002

Product Name	Qili Qiangxin Capsules	Batch No.	B2102002
Strength	0.3 g per capsule	Manufacture Date	Feb 04, 2021
Inspection Purpose	Releasing inspection	Expiry Date	July 2023
Deliver Party	Oral Preparation Workshop	Deliver Quantity	20 boxes + 30 blisters
Package	0.3 g *12 capsule * 3 blister/box	Inspection Date	Feb 07, 2021
Inspection Items	All items	Report Date	Feb 12, 2021
Criteria of Inspection	Chinese Pharmacopoeia 2020, Volume I		

**Inspection Items**	**Standard criteria**	**Testing Data**	**Conclusion**
**Description**	It should be hard capsules, containing dark brownish to black-brownish granules; taste, bitter.	Hard capsules, containing dark brownish to black-brownish granules; taste, bitter.	Approved
**Identification**			
(1) HPLC	The peaks in the chromatogram obtained with the test solution should correspond in the retention time to the peaks in the chromatogram obtained with the reference solution.	The peaks in the test solution correspond in the retention time to the peaks in the chromatogram obtained with the reference solution.	Approved
(2) (3) (4) (5) TLC	TLC of Salviae Miltiorrhizae Rhizoma et Radix reference drug, Periplocae Cortex reference drug, cinnamaldehyde CRS, hesperidin CRS	Positive spots correspond to the chemical reference substance and reference drugs	Approved
**Other requirements**			
Filling variation	Should be within ±10.0%.	Complies	Approved
Water	Not more than 9.0%	3.1%	Approved
Disintegration test	Should disintegrate within 30 minutes	9 minutes	Approved
Limit of aconitine	The spot in the chromatogram obtained with the test solution is not bigger than the spot in the chromatogram obtained with the reference solution, or there is no spot reveals in the chromotogram obtained with the test solution.	Complies	Approved
Microbial limit	Total Aerobic Microbial Count < 10^3cfu/g.<br>Total Yeast and Mold Count < 10^2cfu/g.<br>E.Coli.: Absent/g.	Complies<br>Complies<br>Complies	Approved<br>Approved<br>Approved
Chromium content in capsule shell	Not more than 2ppm.	Complies	Approved
**Assay**	It contains not less than 0.12 mg of astragaloside IV ($C_{41}H_{68}O_{14}$) per capsule, referred to Astragals Radix.	0.60 mg/capsule	Approved

Conclusion: The results meet the requirements of Chinese Pharmacopoeia 2020, Volume I

QC Director: [signature] Checker: [signature] Inspector: [signature]

石家庄以岭药业股份有限公司
YILING SHIJIAZHUANGYILING PHARMACEUTICAL CO.,LTD
中国河北省石家庄市高新技术开发区天山大街 238 号 PC：050035
NO. 238, Tianshan Avenue, Hi-Tech Development Zone, Shijiazhuang, Hebei, China
TEL: 0086 311 85901719 FAX: 0086 311 85901719 http://www.yiling.cn

SHIJIAZHUANG YILING PHARMACEUTICAL CO., LTD

Certificate of Analysis for Finished Product

Report No. Cheng 045 (Bao) B2102003 L-QB-045-C00-002

Product Name	Qili Qiangxin Capsules	Batch No.	B2102003
Strength	0.3 g per capsule	Manufacture Date	Feb 05, 2021
Inspection Purpose	Releasing inspection	Expiry Date	July 2023
Deliver Party	Oral Preparation Workshop	Deliver Quantity	20 boxes + 30 blisters
Package	0.3 g *12 capsule * 3 blister/box	Inspection Date	Feb 08, 2021
Inspection Items	All items	Report Date	Feb 13, 2021
Criteria of Inspection	Chinese Pharmacopoeia 2020, Volume I		

**Inspection Items**	**Standard criteria**	**Testing Data**	**Conclusion**
**Description**	It should be hard capsules, containing dark brownish to black-brownish granules; taste, bitter.	Hard capsules, containing dark brownish to black-brownish granules; taste, bitter.	Approved
**Identification**			
(2) HPLC	The peaks in the chromatogram obtained with the test solution should correspond in the retention time to the peaks in the chromatogram obtained with the reference solution.	The peaks in the test solution correspond in the retention time to the peaks in the chromatogram obtained with the reference solution.	Approved
(2) (3) (4) (5) TLC	TLC of Salviae Miltiorrhizae Rhizoma et Radix reference drug, Periplocae Cortex reference drug, cinnamaldehyde CRS, hesperidin CRS	Positive spots correspond to the chemical reference substance and reference drugs	Approved
**Other requirements**			
Filling variation	Should be within ±10.0%.	Complies	Approved
Water	Not more than 9.0%	4.3%	Approved
Disintegration test	Should disintegrate within 30 minutes	9 minutes	Approved
Limit of aconitine	The spot in the chromatogram obtained with the test solution is not bigger than the spot in the chromatogram obtained with the reference solution, or there is no spot reveals in the chromotogram obtained with the test solution.	Complies	Approved
Microbial limit	Total Aerobic Microbial Count $< 10^3$cfu/g.<br>Total Yeast and Mold Count $< 10^2$cfu/g.<br>E.Coli.: Absent/g.	Complies<br>Complies<br>Complies	Approved<br>Approved<br>Approved
Chromium content in capsule shell	Not more than 2ppm.	Complies	Approved
**Assay**	It contains not less than 0.12 mg of astragaloside IV ($C_{41}H_{68}O_{14}$) per capsule, referred to Astragals Radix.	0.62 mg/capsule	Approved

Conclusion: The results meet the requirements of Chinese Pharmacopoeia 2020, Volume I

QC Director: ____________ Checker: ____________ Inspector: __________

石家庄以岭药业股份有限公司
YILING SHIJIAZHUANGYILING PHARMACEUTICAL CO.,LTD
中国河北省石家庄市高新技术开发区天山大街 238 号 PC：050035
NO. 238, Tianshan Avenue, Hi-Tech Development Zone, Shijiazhuang, Hebei, China
TEL: 0086 311 85901719 FAX: 0086 311 85901719 http://www.yiling.cn

SHIJIAZHUANG YILING PHARMACEUTICAL CO., LTD

Certificate of Analysis for Finished Product

Report No. Cheng 045 (Bao) B2102004 L-QB-045-C00-002

Product Name	Qili Qiangxin Capsules	Batch No.	B2102004
Strength	0.3 g per capsule	Manufacture Date	Feb 05, 2021
Inspection Purpose	Releasing inspection	Expiry Date	July 2023
Deliver Party	Oral Preparation Workshop	Deliver Quantity	20 boxes + 30 blisters
Package	0.3 g *12 capsule * 3 blister/box	Inspection Date	Feb 09, 2021
Inspection Items	All items	Report Date	Feb 14, 2021
Criteria of Inspection	Chinese Pharmacopoeia 2020, Volume I		

**Inspection Items**	**Standard criteria**	**Testing Data**	**Conclusion**
**Description**	It should be hard capsules, containing dark brownish to black-brownish granules; taste, bitter.	Hard capsules, containing dark brownish to black-brownish granules; taste, bitter.	Approved
**Identification**			
(3) HPLC	The peaks in the chromatogram obtained with the test solution should correspond in the retention time to the peaks in the chromatogram obtained with the reference solution.	The peaks in the test solution correspond in the retention time to the peaks in the chromatogram obtained with the reference solution.	Approved
(2) (3) (4) (5) TLC	TLC of Salviae Miltiorrhizae Rhizoma et Radix reference drug, Periplocae Cortex reference drug, cinnamaldehyde CRS, hesperidin CRS	Positive spots correspond to the chemical reference substance and reference drugs	Approved
**Other requirements**			
Filling variation	Should be within ±10.0%.	Complies	Approved
Water	Not more than 9.0%	3.7%	Approved
Disintegration test	Should disintegrate within 30 minutes	9 minutes	Approved
Limit of aconitine	The spot in the chromatogram obtained with the test solution is not bigger than the spot in the chromatogram obtained with the reference solution, or there is no spot reveals in the chromotogram obtained with the test solution.	Complies	Approved
Microbial limit	Total Aerobic Microbial Count < 10^3cfu/g.<br>Total Yeast and Mold Count < 10^2cfu/g.<br>E.Coli.: Absent/g.	Complies<br>Complies<br>Complies	Approved<br>Approved<br>Approved
Chromium content in capsule shell	Not more than 2ppm.	Complies	Approved
**Assay**	It contains not less than 0.12 mg of astragaloside IV ($C_{41}H_{68}O_{14}$) per capsule, referred to Astragals Radix.	0.68 mg/capsule	Approved

Conclusion: The results meets the requirements of Chinese Pharmacopoeia 2020, Volume I

QC Director: ____________ Checker: ____________ Inspector: ____________

2.6 Analysis Procedures for Finished Product

Description

It should be hard capsules, containing dark brownish to black-brownish granules; taste, bitter.

Identification

(1) Take the solution under the Assay as the test solution. Dissolve quantities of ginsenoside Rb_1 CRS ginsenoside Rb_2 and ginsenoside Rf CRS in methanol to produce a mixture containing 0.2 mg of each per ml as the reference solution. Carry out the method described under the Assay. Inject separately 5-15 µl of the test solution obtained under the Assay and the above reference solution into the column and record the chromatogram.

(2) Ultrasonicate 2 g of content of the capsules with 25 ml of methanol for 30 minutes, filter, evaporate the filtrate to dryness, dissolve the residue with 25 ml of water, adjust pH to 1-2 with hydrochloric acid, extract with two 15-ml quantities of ethyl acetate, combine the extracts, evaporate to dryness, and dissolve the residue in 1 ml of dehydrated ethanol as the test solution. To 0.5 g of Salviae Miltiorrhizae Rhizoma et Radix reference drug, add 30 ml of water, heat under reflux for 1 hour, filter, and treat the filtrate in the same manner as above, beginning at "adjust pH to 1-2 with hydrochloric acid", to prepare the reference drug solution. Carry out the method for thin layer chromatography, using silica gel G as the coating substance and a mixture of toluene, dichloromathane, ethyl acetate and formic acid (5:5:5:0.8) as the mobile phase. Apply separately to the plate 3-7µl each of the above two solutions. After developing and removal of the plate, dry in air, and spray with 2% solution of ferric chloride in ethanol, heat to the spots clear. The spots in the chromatogram obtained with the test solution correspond in position and colour to the spots in the chromatogram obtained with the reference drug solution.

(3) Concentrate the remaining methanol solution obtained under Assay to about 2 ml as the test solution. Ultrasonicate 0.5 g of Periplocae Cortex reference drug with 10 ml of methanol for 30 minutes, filter, concentrate the filtrate to about 2 ml as the reference drug solution. Carry out the method for thin layer chromatography, using silica gel G as the coating substance and a mixture of petroleum (60-90℃), ethyl acetate and glacial acetic acid (20:3:0.5) as the mobil phase. Apply separately to the plate 5-10 µl of the test solution and 2-4 µl the reference drug solution. After developing and removal of the plate, dry in air, and examine under ultraviolet light at 254 nm. The fluorescent spots in the chromotogram obtained with the test solution correspond in position and colour to the spots in the chromotogram obtained with reference drug solution.

(4) Macerate 2 g of content of the capsules with 20 ml of ethanol for 20 minutes in a conical flask with stopper, shake for 10 minutes, filter, and use the filtrate as the test solution. Dissolve a quantity of cinnamaldehyde CRS in methanol to produce a solution containing 1µl per ml as the reference solution. Carry out the method for thin

中国河北省石家庄市高新技术开发区天山大街 238 号 PC：050035
NO. 238, Tianshan Avenue, Hi-Tech Development Zone, Shijiazhuang, Hebei, China
TEL: 0086 311 85901719 FAX: 0086 311 85901719 http://www.yiling.cn

layer chromatography, using silica gel G as the coating substance and a mixture of petroleum ether (60-90°C) and ethyl acetate (17:3) as the mobile phase. Apply separately to the plate 10 μl-15 μl of the test solutions and 2 μl of the reference solution. After developing and removal of the plate, dry in air, spry with dinitrophenylhydrazine TS in ethanol, allow to stand 5 minutes, and examine in daylight. The spot in the chromatogram obtained with the test solution corresponds in position and colour to the spot in the chromotogram obtained with reference solution.
(5) Evaporate the test solution obtained under *Identification* (3) to dryness, dissolve the residue with 10 ml of water, extract with two 15-ml quantities of chloroform, discard the chloroform layer, extract the aqueous solution with two 15- ml quantities of ethyl acetate, evaporate the supernatant to dryness, and dissolve the residue in 1 ml of methanol as the test solution. Dissolve a quantity of hesperidin CRS in methanol to produce a saturated solution as the reference solution. Carry out the method for thin layer chromatography, using silica gel G as the coating substance and the lower layer of a mixture of chloroform, ethyl acetate, methanol and water (15:40:22:10), stood below 10°C, as the mobile phase. Apply separately to the plate 5-10 μl of the test solution and 2 μl of the reference solution. After developing at 4°C temperature, and removal of the plate, dry in air, spray with aluminum trichloride TS, and examine under ultraviolet light at 365nm. The fluorescent spot in the chromotogram obtained with the test solution corresponds in position and colour to the spot in the chromotogram obtained with reference solution.
Filling variation Should comply to the standard

Other requirements

Filling Variation

Procedure Weigh accurately each of 10 capsules for Chinese medicine, unless otherwise specified. Open each capsule without loss of shell material, and remove the content as completely as possible: for hard capsules, clean the shell with a small brush. Weigh the shell of each capsule. Calculate the weight of content of each capsule and the average weight. Not more than 2 of the individual weights should deviate from the average (or labelled) weight by more than the weight variation limit shown in the table, and none should deviate by more than twice the limit. Filling Variation should be within ±10.0%.

Water

The water content of the contents of the Toluene distillation method.

Apparatus The apparatus consists of a 500 ml round bottom flask (A), a graduated receiving tube (B) and a reflux condenser (C) approximately 40 cm in length. All parts of the apparatus should be cleaned and dried in an oven.

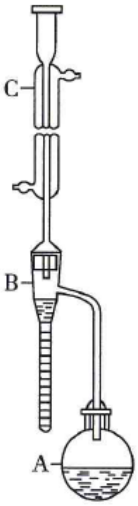

Procedure Place a quantity of the substance being examined which is anticipated to yield about 1-4 ml of water, accurately weighed, in the flask A. Add about 200 ml of toluene and dry clean unglazed porcelain or a few glass beads if necessary. Assemble the apparatus and fill the receiving tube B with toluene through the condenser. Heat the flask gently, when toluene begins to boil, adjust the temperature and allow to distill at a rate of 2 drops per second. When the volume of water in the receiving tube does not increase any more, rinse the inside of condenser with toluene and push down the toluene adhering to the wall with a brush or other suitable tools. Continue the distillation for 5 minutes, cool to room temperature and disconnect the apparatus. Push down any droplet of water adhering to the wall of the receiving tube with a copper wire wetted with toluene, Allow to stand until water is completely separated from toluene in the receiving tube (a small amount of methylene blue may be added to facilitate observation). Record the volume of water distilled and calculate the percentage content of water in the substance being examined.

NOTE (1) The toluene to be used should be prepared as follows. Add a small quantity of water. Shake thoroughly and allow to stand for a while. Discard the water layer and distill the remainder. (2) The substance of Chinese medicine for determination should be broken into granules or pieces with no more than 3 mm in diametre. The substances which have less than 3 mm in length and diametre, can be examined without further breaking.

The water content is not more than 9.0 per cent.

Disintegration test

Disintegration should be achieved when no residue, except fragments of undissolved tablet coating or of capsule shell, remains on the screen of the test apparatus.

Disintegration can be also considered to be achieved when the residue remained consists of a soft mass having no palpably, unmoistened core, or floats because of light weight.

Apparatus The apparatus mainly consists of a basket-rack assembly with disks and a metallic device capable of raising and lowering the basket in the liquid medium at a constant rate between 30 and 32 cycles per minute through a distance of 55 mm±2 mm.

Procedure Take 6 test samples, the hard capsules should all disintegrate within 30 minutes. If one of the capsules has not disintegrated, repeat the test on further six capsules, all the capsules should comply with the test.

Microbial limit

Microbial limit of Qili Qiangxin Capsules should comply with the Mierobiological Examination of Nonsterile Products: Microbial Enumeration Tests.

***Escherichia* coli** Microbiological Examination of Nonsterile Products: Test for Specified Microorganisms.

Sample preparation and pre-incubation According to the method of Microbial enumeration tests to make a 1: 10 dilution of the product. Inoculate sample equivalent to 1 g or 1 ml of the product to be examined to a suitable amount (determined as described under Suitability of the test method) of soya bean casein digest broth, mix and incubate at 30-35°C for 18-24 hours.

Selection and subculture Transfer 1 ml pre-incubation broth to 100 ml macconkey broth and incubate at 42-44°C for 24-48 hours. Subculture a small amount of macconkey broth onto macconkey agar and incubate at 30-35°Cfor 18-72 hours.

Interpretation Growth of colonies on macconkey agar indicates the possible presence of *E. coli*. This is confirmed by isolation, purification and appropriate identification tests. The product is qualified if no colonies are present or if the identification tests are negative.

TAMC-Total Aerobic Microbial Count: Not more than 10^3cfu.

TYMC-Total combined Yeast and Mold Count: Not more than 10^2cfu.

Specified Microorganisms: Absence of *Escherichia coli* per 1 g

Chromium content in capsule shell

Chromium Place 0.5 g in a PTFE vessel, add 5-10 ml of nitric acid, shake thoroughly, allow to stand overnight Stopper and allow to digest in a microwave digestion system. After digestion has been completed, evaporate the solution on the eletric heating plate until reddish-brown fumes are no longer evolved and near to dryness by gently heating. Transfer to a 50 ml volumetric flask with 2% nitric acid, and dilute to volume as test solution. Perform a blank test omitting the substance being exarined. Use the solution obtained as blank solution. Transfer accurately a quantity of chromium standard solution, dilute with 2% nitric acid solution to produce a solution of 1.0 g per ml as chromium standard stock solution. Before determination, transfer accurately a quantity of chromium standard stock solution, dilute with 2% nitric acid solution to produce a successive solution of 0-80 ng per ml as chromium reference solutions. Carry out the method for atomic absorbance spectrophotometry measure the absorbances of reference

solutions and test solution at 357.9 nm. Calculate the content of chromium: not more than 0.0002%.

Limit of aconitine

To 18 g of the content of capsules in a conical flask with stopper, add 10 ml of ammonia TS, shake for 30 minutes, add 100 ml of ether, stopper the flask, shake for 15 minutes, allow to stand for 24 hours, filter the ether layer, wash the residue and filter paper with 10 ml of ether, combine the filtrates, evaporate the solvent at low temperature, and dissolve the residue with dehydrated ethanol, transfer to a 2 ml volumetric flask, and dilute with ethanol to the volume as the test solution. Dissolve a quantity of aconitine CRS in dehydrated ethanol to produce a solution containing 1.0 mg per ml as the reference solution. Carry out the method for thin layer chromatography, using silica gel G as the coating substance and a mixture of *n*-hexane, ethyl acetate and ethanol (6.4:3.6:1) as the mobile phase. Apply separately to the plate 12 µl of the test solution and 5 µl of the reference solution. After developing in a chamber saturated with ammonia vapour, and removal of the plate, dry in air, spray with dilute bismuth potassium iodide TS, and examine in daylight. The spot in the chromatogram obtained with the test solution is not bigger than the spot in the chromatogram obtained with the reference solution, or there is no spot reveals in the chromotogram obtained with the test solution.

Assay

Carry out the method for high performance liquid chromatography. *Chromatographic system and system suitability* Use octadecylsilane bonded silica gel as the stationary phase, column temperature set at 30°C, and a mixture of acetonitrile and water (30:70) as the mobile phase. Use an evaporative light scattering detector. The number of theoretical plates of column is not less than 4000, calculated with the reference to the peak of astragaloside IV.

Reference solution Dissolve a quantity of astragaloside IV CRS, weighed accurately, in methanol to produce the reference solution containing 80 µg per ml.

Test solution Weigh accurately 2 g of the pulverized powder, obtained under the packing variation test, in a conical flask with stopper, add accurately 50 ml of methanol, stopper the flask, weigh the weight, ultrasonicate (power 250 W, frequency 40 kHz) for 30 minutes. Allow to cool, weigh again, and replenish the lost weight with methanol, mix well, filter, measure accurately 20 ml of the successive filtrate (keep the rest of methanol solution for other use), evaporate to dryness, and dissolve the residue with 20 ml of 3% sodium hydroxide solution, extract with three 20-ml quantities of *n*-butanol saturated with water, combine the extracts, wash with two 25-ml quantities of water saturated with *n*-butanol, combine the aqueous solution, extract with 20 ml of *n*-butanol saturated with water, combine the *n*-butanol extracts, evaporate the solvent to dryness, dissolve the residue with 70% ethanol, transfer to 5 ml volumetric flask, dilute with 70% ethanol to the volume, shake well, filter, and use the successive filtrate as the test solution.

Procedure Inject accurately 5 µl and 15 µl of the reference solution, 5-15 µl of the test

solution into the column, respectively, and calculate the content, using a calibration equation of logarithm conversion of external standard method. It contains not less than 0.12 mg of astragaloside ($C_{41}H_{68}O_{14}$) per capsule, referred to Astragals Radix.

2.7 Reference standards or materials

Reference drug/substance	Supplier	Batch No.
ginsenoside Rb_1	National institutes for food and drug control	110704-202129
ginsenoside Rb_2	National institutes for food and drug control	111715-201203
ginsenoside R_f	National institutes for food and drug control	111719-201806
Salviae Miltiorrhizae Rhizoma et Radix	National institutes for food and drug control	120923-201816
Periplocae Cortex	National institutes for food and drug control	121025-201404
cinnamaldehyde	National institutes for food and drug control	110710-202022
hesperidin	National institutes for food and drug control	110721-202019
aconitine	PCL	A1907010
astragaloside IV	National institutes for food and drug control	110781-202118

中国河北省石家庄市高新技术开发区天山大街 238 号 PC: 050035
No.238 Tianshan Street, High-Tech Area, Shijiazhuang Hebei Province, China.
TEL: 0086 311 85901719 FAX: 0086 311 85901719 http://www.yiling.cn

2.8 Stability summary and conclusion

Long term stability test under condition of 30℃±2℃/ 75%±5%RH for 36 months and accelerate stability test under condition of 40℃±2℃/75%±5%RH for 6 months were conducted as the following table:

Storage Condition → Testing frequency ↓	Long term stability test 30℃±2℃/ 75%±5%RH	Accelerate stability test 40℃±2℃ /75%±5%RH
Initial	√	√
1 month	×	√
2 months	×	√
3 months	√	√
6 months	√	√
9 months	√	×
12 months	√	×
18 months	√	×
24 months	√	×
36 months	√	×

Stability data of accelerate stability test (AST) and long term stability test (LST)

Items	Specification	LST	AST
Description	It should be hard capsules, containing dark brownish to black-brownish granules; taste, bitter.	+	+
Identification	(1) HPLC The peaks in the chromatogram obtained with the test solution should correspond in the retention time to the peaks in the chromatogram obtained with the reference solution.	+	+
	(2) (3) (4) (5) TLC TLC of Salviae Miltiorrhizae Rhizoma et Radix reference drug, Periplocae Cortex		

中国河北省石家庄市高新技术开发区天山大街 238 号 PC：050035
No.238 Tianshan Street, High-Tech Area, Shijiazhuang Hebei Province, China.
TEL: 0086 311 85901719 FAX: 0086 311 85901719 http://www.yiling.cn

	reference drug, cinnamaldehyde CRS, hesperidin CRS		
Filling variation	should be within ±10.0%	Qualified	Qualified
Water (per cent)	Not more than 9.0 %.	Qualified	Qualified
Disintegration (minute)	Should disintegrate within 30 minutes	Qualified	Qualified
Microbial limit	Should comply with the standard.<br>- Total Aerobic Microbial Count: NMT 10^3 CFU/g<br>- Total combined Yeast and Mold Count: NMT 10^2 CFU/g<br>- E.Coli: Absent/ g	Qualified	Qualified
Assay (mg)	It contains not less than 0.12 mg of astragaloside IV ($C_{41}H_{68}O_{14}$) per capsule, referred to Astragals Radix.	Qualified	Qualified
Limit of aconitine	The spot in the chromatogram obtained with the test solution is not bigger than the spot in the chromatogram obtained with the reference solution, or there is no spot reveals in the chromotogram obtained with the test solution.	Qualified	Qualified
Conclusion	——	Qualified	Qualified

Statement: Qili Qiangxin Capsules is found to be stable and two batches (batch No. 100101, 100102, 100103) remain within the specification after 36 months of the storage condition at 30℃±2℃/ 75%±5%RH. It is stable and one batch (batch No. 100101, 100102, 100103) remain within the specification after 6 months of the storage condition at 40℃±2℃/75%±5%RH.

Proposed storage condition:

Based on the stability test, following storage condition has been proposed:

“Preserve in tightly closed containers.”

中国河北省石家庄市高新技术开发区天山大街 238 号 PC：050035
NO. 238, Tianshan Avenue, Hi-Tech Development Zone, Shijiazhuang, Hebei, China
TEL: 0086 311 85901719 FAX: 0086 311 85901719 http://www.yiling.cn

Qili Qiangxin Capsules
(0.3 g/capsule)

ACCELERATED STABILITY TEST PROTOCOL

Shijiazhuang Yiling Pharmaceutical Co., Ltd.

石家庄以岭药业股份有限公司
SHIJIAZHUANGYILING PHARMACEUTICAL CO.,LTD

Qili Qiangxin Capsules Accelerated Stability Test Protocol

1. Objective

Through accelerated stability test, to monitor the product over its shelf life and to determine that the product remains, and can be expected to remain, within specification under the labeled storage conditions.

2. Scope

This protocol only applied to accelerated stability test of finished product of Qili Qiangxin Capsules.

3. Content

3.1. Information of test sample of Qili Qiangxin Capsules

Name	Strength	Batch No.	Packaging	Batch size	Date of production	Validity to
Qili Qiangxin Capsules	0.3g/capsule	100101	Aluminum panel and box	1.2 million capsules	January 3, 2010	*December 2011*
Qili Qiangxin Capsules	0.3g/capsule	100102	Aluminum panel and box	1.2 million capsules	January 4, 2010	*December 2011*
Qili Qiangxin Capsules	0.3g/capsule	100103	Aluminum panel and box	1.2 million capsules	January 5, 2010	*December 2011*

3.2. Amount of test sample: 4 times of amount for all items' inspection.

3.3. Test items and criteria:

3.3.1. Appearance: The product should be hard capsules, containing dark brownish to black-brownish granules; taste, bitter.

3.3.2. Identification:

(1) The peaks in the chromatogram obtained with the test solution should correspond in the retention time to the peaks in the chromatogram obtained with the reference solution.

(2) (3) (4) (5) TLC of Salviae Miltiorrhizae Rhizoma et Radix reference drug, Periplocae Cortex reference drug, cinnamaldehyde CRS, hesperidin CRS.

3.3.3. Filling variation: should be within ±10.0%

3.3.4. Determination of water: Not more than 9.0 %.

3.3.5. Disintegration: Should disintegrate within 30 minutes

3.3.6. Assay:

It contains not less than 0.12 mg of astragaloside IV ($C_{41}H_{68}O_{14}$) per capsule, referred to Astragals Radix.

3.3.7. Limit of Aconitine: The spot in the chromatogram obtained with the test solution is not bigger than the spot in the chromatogram obtained with the reference solution, or there is no spot reveals in the chromotogram obtained with the test solution.

3.4. Acceptance criteria: Chinese Pharmacopeia 2010, Volume I.

3.5. Storage condition

Temperature: 40℃ ± 2℃; RH = 75% ± 5%

3.6. Sampling time

Test samples should be stored under long-term test condition, and check if it reaches to the test condition during the test, sampling separately once at 1 months, 2 months, 3months, 6 months, inspect according to specified items in test method.

3.7. Manager of reserving samples will summary all the test data and write a valuation report after completing all the test of each batch.

Stability Data

TABLE 1: ACCELERATED STABILITY DATA (Log)

Drug Product: Qili Qiangxin Capsules — Batch No: 100101
Mfg Date: January 3, 2010 — Expiry date: December 2011
Temperature: 40°C ± 2°C; RH = 75% ± 5% — Package: blister and box

Tests	Specification	Periods (months)				
		0	1	2	3	6
**Description**	It should be hard capsules, containing dark brownish to black-brownish granules; taste, bitter.	C*	C*	C*	C*	C*
**Identification**	(1) HPLC The peaks in the chromatogram obtained with the test solution should correspond in the retention time to the peaks in the chromatogram obtained with the reference solution.	C*	C*	C*	C*	C*
	(2) (3) (4) (5) TLC TLC of Salviae Miltiorrhizae Rhizoma et Radix reference drug, Periplocae Cortex reference drug, cinnamaldehyde CRS, hesperidin CRS	C*	C*	C*	C*	C*
**Filling Variation**	should be within ±10.0%	6.1-3.5	5.6-4.4	5.9-1.3	3.5-2.1	5.3-1.2
**Water (per cent)**	Not more than 9.0 %.	4.5	5.9	5.8	5.7	5.9
**Disintegration (minute)**	Should disintegrate within 30 minutes	10	7	7	7	6
**Assay (mg)**	It contains not less than 0.12 mg of astragaloside IV ($C_{41}H_{68}O_{14}$) per capsule, referred to Astragals Radix.	0.33	0.22	0.23	0.23	0.32
**Limit of Aconitine**	The spot in the chromatogram obtained with the test solution is not bigger than the spot in the chromatogram obtained with the reference solution, or there is no spot reveals in the chromotogram obtained with the test solution.	C*	C*	C*	C*	C*
**Conclusion**	According to In-house standard of Qili Qiangxin Capsules, the product are stable when stored in tightly sealed package at 40°C ± 2°C; RH = 75% ± 5%					

C: Corresponded*

Stability Data

TABLE 2: ACCELERATED STABILITY DATA (Log)

Drug Product: Qili Qiangxin Capsules　　Batch No: 100102
Mfg Date: January 4, 2010　　Expiry date: December 2011
Temperature: 40°C ± 2°C; RH = 75% ± 5%　　Package: blister and box

Tests	Specification	Periods (months)				
		0	1	2	3	6
**Description**	It should be hard capsules, containing dark brownish to black-brownish granules; taste, bitter.	C*	C*	C*	C*	C*
**Identification**	(1) HPLC The peaks in the chromatogram obtained with the test solution should correspond in the retention time to the peaks in the chromatogram obtained with the reference solution.	C*	C*	C*	C*	C*
	(2) (3) (4) (5) TLC TLC of Salviae Miltiorrhizae Rhizoma et Radix reference drug, Periplocae Cortex reference drug, cinnamaldehyde CRS, hesperidin CRS	C*	C*	C*	C*	C*
**Filling Variation**	should be within ±10.0%	6.6-1.4	4.9-1.7	3.7-1.8	4.3-3.5	2.8-2.2
**Water (per cent)**	Not more than 9.0 %.	4.3	5.3	5.9	5.2	5.6
**Disintegration (minute)**	Should disintegrate within 30 minutes	11	7	7	8	6
**Assay (mg)**	It contains not less than 0.12 mg of astragaloside IV ($C_{41}H_{68}O_{14}$) per capsule, referred to Astragals Radix.	0.24	0.18	0.2	0.19	0.19
**Limit of Aconitine**	The spot in the chromatogram obtained with the test solution is not bigger than the spot in the chromatogram obtained with the reference solution, or there is no spot reveals in the chromotogram obtained with the test solution.	C*	C*	C*	C*	C*
**Conclusion**	According to In-house standard of Qili Qiangxin Capsules, the product are stable when stored in tightly sealed package at 40°C ± 2°C; RH = 75% ± 5%					

C: Corresponded*

石家庄以岭药业股份有限公司
YILING SHIJIAZHUANGYILING PHARMACEUTICAL CO.,LTD
中国河北省石家庄市高新技术开发区天山大街 238 号 PC：050035
NO. 238, Tianshan Avenue, Hi-Tech Development Zone, Shijiazhuang, Hebei, China
TEL: 0086 311 85901719 FAX: 0086 311 85901719 http://www.yiling.cn

Stability Data

TABLE 3: ACCELERATED STABILITY DATA (Log)

Drug Product: Qili Qiangxin Capsules Batch No: 100103
Mfg Date: January 5, 2010 Expiry date: December 2011
Temperature: 40°C ± 2°C; RH = 75% ± 5% Package: blister and box

Tests	Specification	Periods (months)				
		0	1	2	3	6
**Description**	It should be hard capsules, containing dark brownish to black-brownish granules; taste, bitter.	C*	C*	C*	C*	C*
**Identification**	(1) HPLC The peaks in the chromatogram obtained with the test solution should correspond in the retention time to the peaks in the chromatogram obtained with the reference solution.	C*	C*	C*	C*	C*
	(2) (3) (4) (5) TLC TLC of Salviae Miltiorrhizae Rhizoma et Radix reference drug, Periplocae Cortex reference drug, cinnamaldehyde CRS, hesperidin CRS	C*	C*	C*	C*	C*
**Filling Variation**	should be within ±10.0%	5.0-1.4	6.4-2.3	6.0-1.1	3.6-5.1	4.9-2.2
**Water (per cent)**	Not more than 9.0 %.	5.1	5.3	5.9	7.6	5.2
**Disintegration (minute)**	Should disintegrate within 30 minutes	9	7	7	7	7
**Assay (mg)**	It contains not less than 0.12 mg of astragaloside IV ($C_{41}H_{68}O_{14}$) per capsule, referred to Astragals Radix.	0.34	0.24	0.28	0.23	0.33
**Limit of Aconitine**	The spot in the chromatogram obtained with the test solution is not bigger than the spot in the chromatogram obtained with the reference solution, or there is no spot reveals in the chromotogram obtained with the test solution.	C*	C*	C*	C*	C*
**Conclusion**	According to In-house standard of Qili Qiangxin Capsules, the product are stable when stored in tightly sealed package at 40°C ± 2°C; RH = 75% ± 5%					

C: Corresponded*

Evaluation Report of Qili Qiangxin Capsules accelerated Stability Study

1. Objective

To investigate quality of Qili Qiangxin Capsules within the validity, by Comparing the test result of accelerated retention sample with the results of 0 month sample.

2. Evaluation items

2.1 Description

Description was consistent with the requirements, within the test period (6 months).

2.2 Identification

Identification items of the batch were consistent with the requirements within test period (6 months), seen from stability log results.

2.3 Other requirements

2.3.1 Filling variation

Filling variation of the batch was consistent with the requirements within test period (6 months), seen from the results of stability log.

2.3.2 Water

Water of the batch was consistent with the requirements within test period (6 months), seen from the results of stability log.

2.3.3 Disintegration

Disintegration of the batch was consistent with the requirements within test period (6 months), seen from the results of stability log.

2.4 Assay

Batch No.	100101	100102	100103
Range of content (mg/capsule)	0.22-0.33	0.18-0.24	0.23-0.34

Assay results of batch were consistent with the requirements within test period (6 months) seen from the above data. The difference of results belongs to random error.

3. Conclusion

All items comply with the requirements within 6 months, seen from the above results. Therefore, it can be concluded that the quality of Qili Qiangxin Capsules is stable within validity.

石家庄以岭药业股份有限公司
YILING SHIJIAZHUANGYILING PHARMACEUTICAL CO.,LTD
中国河北省石家庄市高新技术开发区天山大街 238 号 PC: 050035
NO. 238, Tianshan Avenue, Hi-Tech Development Zone, Shijiazhuang, Hebei, China
TEL: 0086 311 85901719 FAX: 0086 311 85901719 http://www.yiling.cn

Qili Qiangxin Capsules
(0.3 g/capsule)

LONG TERM STABILITY TEST PROTOCOL

Shijiazhuang Yiling Pharmaceutical Co., Ltd.

石家庄以岭药业股份有限公司
SHIJIAZHUANGYILING PHARMACEUTICAL CO.,LTD

中国河北省石家庄市高新技术开发区天山大街 238 号 PC：050035
NO. 238, Tianshan Avenue, Hi-Tech Development Zone, Shijiazhuang, Hebei, China
TEL: 0086 311 85901719 FAX: 0086 311 85901719 http://www.yiling.cn

Qili Qiangxin Capsules Long-term Stability Test Protocol

1. Objective

Through long term stability test, to monitor the product over its shelf life and to determine that the product remains, and can be expected to remain, within specification under the labeled storage conditions.

2. Scope

This protocol only applied to long-term stability test of finished product of Qili Qiangxin Capsules.

3. Content

3.1. Information of test sample of Qili Qiangxin Capsules

Name	Strength	Batch No.	Packaging	Batch size	Date of production	Validity to
Qili Qiangxin Capsules	0.3g/capsule	100101	Aluminum panel and box	1.2 million capsules	January 3, 2010	*December 2011*
Qili Qiangxin Capsules	0.3g/capsule	100102	Aluminum panel and box	1.2 million capsules	January 4, 2010	*December 2011*
Qili Qiangxin Capsules	0.3g/capsule	100103	Aluminum panel and box	1.2 million capsules	January 5, 2010	*December 2011*

3.2. Amount of test sample: 8 times of amount for all items' inspection.

3.3. Test items and criteria:

3.3.1. Appearance: The product should be hard capsules, containing dark brownish to black-brownish granules; taste, bitter.

3.3.2. Identification:

(1) The peaks in the chromatogram obtained with the test solution should correspond in the retention time to the peaks in the chromatogram obtained with the reference solution.

(2) (3) (4) (5) TLC of Salviae Miltiorrhizae Rhizoma et Radix reference drug, Periplocae Cortex reference drug, cinnamaldehyde CRS, hesperidin CRS.

3.3.3. Filling variation: should be within ±10.0%

3.3.4. Determination of water: Not more than 9.0 %.

3.3.5. Disintegration: Should disintegrate within 30 minutes

3.3.6. Microbial limits test:

-Should comply with the standard.

- Bacteria count: NMT 1000 cfu/g

-Total combined yeasts and molds count: NMT 100 cfu/g

- E.Coli: Absent/ g

3.3.7. Assay:

It contains not less than 0.12 mg of astragaloside IV ($C_{41}H_{68}O_{14}$) per capsule, referred to Astragals Radix.

3.3.8. Limit of Aconitine: The spot in the chromatogram obtained with the test solution is not bigger than the spot in the chromatogram obtained with the reference solution, or there is no spot reveals in the chromotogram obtained with the test solution.

3.4. Acceptance criteria: Chinese Pharmacopeia 2010, Volume I.

中国河北省石家庄市高新技术开发区天山大街 238 号 PC: 050035
NO. 238, Tianshan Avenue, Hi-Tech Development Zone, Shijiazhuang, Hebei, China
TEL: 0086 311 85901719 FAX: 0086 311 85901719 http://www.yiling.cn

3.5. Storage condition

Temperature: 30ºC ± 2ºC; RH = 75% ± 5%

3.6. Sampling time

Test samples should be stored under long-term test condition, and check if it reaches to the test condition during the test, sampling separately once at 3 months, 6 months, 9 months, 12 months, 18 months, 24 months and 36 months, inspect according to specified items in test method.

3.7. Manager of reserving samples will summary all the test data and write a valuation report after completing all the test of each batch.

石家庄以岭药业股份有限公司
YILING SHIJIAZHUANGYILING PHARMACEUTICAL CO.,LTD
中国河北省石家庄市高新技术开发区天山大街 238 号 PC：050035
NO. 238, Tianshan Avenue, Hi-Tech Development Zone, Shijiazhuang, Hebei, China
TEL: 0086 311 85901719 FAX: 0086 311 85901719 http://www.yiling.cn

Stability Data

Table 1 LONG-TERM STABILITY DATA

Drug Product: Qili Qiangxin Capsules Package: blister and box Batch No: 100101
Mfg Date: January 3, 2010 Expiry date: December 2011 Temperature: 30°C± 2°C; RH = 75% ± 5%

Tests	Specification	Periods (months)							
		0	3	6	9	12	18	24	36
Description	It should be hard capsules, containing dark brownish to black-brownish granules; taste, bitter.	C*	C*	C*	C*	C*	C*	C*	C*
Identification	(1) HPLC The peaks in the chromatogram obtained with the test solution should correspond in the retention time to the peaks in the chromatogram obtained with the reference solution.	C*	C*	C*	C*	C*	C*	C*	C*
	(2) (3) (4) (5) TLC TLC of Salviae Miltiorrhizae Rhizoma et Radix reference drug, Periplocae Cortex reference drug, cinnamaldehyde CRS, hesperidin CRS	C*	C*	C*	C*	C*	C*	C*	C*
Filling variation	should be within ±10.0%	6.1-3.5	5.1-3.4	4.3-7.7	4.5-1.5	3.9-4.1	3.7-7.8	6.8-5.9	7.4-6.6
Water (per cent)	Not more than 9.0 %.	4.5	5.1	5.3	4.3	5.1	4.9	5.1	4.7
Disintegration (minute)	Should disintegrate within 30 minutes	10	7	6	7	6	7	7	8
Microbial limit	Should comply with the standard.<br>- Total Aerobic Microbial Count: NMT 10^3 CFU/g<br>- Total combined Yeast and Mold Count: NMT 10^2 CFU/g<br>- E.Coli: Absent/ g	<10<br><10<br>Absent	<10<br><10<br>Absent	<10<br><10<br>Absent	10<br><10<br>Absent	<10<br><10<br>Absent	20<br><10<br>Absent	<10<br><10<br>Absent	<10<br><10<br>Absent
Assay (mg)	It contains not less than 0.12 mg of astragaloside IV ($C_{41}H_{68}O_{14}$) per capsule, referred to Astragals Radix.	0.33	0.27	0.26	0.33	0.25	0.25	0.32	0.35
Limit of aconitine	The spot in the chromatogram obtained with the test solution is not bigger than the spot in the chromatogram obtained with the reference solution, or there is no spot reveals in the chromotogram obtained with the test solution.	C*	C*	C*	C*	C*	C*	C*	C*
Conclusion	According to in-house standard of Qili Qiangxin Capsules, the product are stable within 36 months when stored in tightly sealed package at 30°C ± 2°C/RH:75% ± 5%								

C: Corresponded*

石家庄以岭药业股份有限公司
YILING SHIJIAZHUANGYILING PHARMACEUTICAL CO.,LTD
中国河北省石家庄市高新技术开发区天山大街 238 号 PC：050035
NO. 238, Tianshan Avenue, Hi-Tech Development Zone, Shijiazhuang, Hebei, China
TEL: 0086 311 85901719 FAX: 0086 311 85901719 http://www.yiling.cn

Stability Data

Table 1 LONG-TERM STABILITY DATA

Drug Product: Qili Qiangxin Capsules Package: blister and box Batch No: 100102
Mfg Date: January 4, 2010 Expiry date: December 2011 Temperature: 30°C± 2°C; RH = 75% ± 5%

Tests	Specification	Periods (months)							
		0	3	6	9	12	18	24	36
Description	It should be hard capsules, containing dark brownish to black-brownish granules; taste, bitter.	C*	C*	C*	C*	C*	C*	C*	C*
Identification	(1) HPLC The peaks in the chromatogram obtained with the test solution should correspond in the retention time to the peaks in the chromatogram obtained with the reference solution.	C*	C*	C*	C*	C*	C*	C*	C*
	(2) (3) (4) (5) TLC TLC of Salviae Miltiorrhizae Rhizoma et Radix reference drug, Periplocae Cortex reference drug, cinnamaldehyde CRS, hesperidin CRS	C*	C*	C*	C*	C*	C*	C*	C*
Filling variation	should be within ±10.0%	6.6-1.4	6.3-2.0	5.2-2.7	5.7-1.4	6.1-3.8	3.2-1.6	7.5-3.4	7.5-5.0
Water (per cent)	Not more than 9.0 %.	4.3	5.1	5.3	4.6	5	4.4	5.2	5.7
Disintegration (minute)	Should disintegrate within 30 minutes	11	8	6	6	6	7	7	7
Microbial limit	Should comply with the standard.<br>- Total Aerobic Microbial Count: NMT 10^3 CFU/g<br>- Total combined Yeast and Mold Count: NMT 10^2 CFU/g<br>- E.Coli: Absent/ g	<10<br><10<br>Absent	<10<br><10<br>Absent	<10<br><10<br>Absent	<10<br><10<br>Absent	<10<br><10<br>Absent	20<br><10<br>Absent	<10<br><10<br>Absent	10<br><10<br>Absent
Assay (mg)	It contains not less than 0.12 mg of astragaloside IV ($C_{41}H_{68}O_{14}$) per capsule, referred to Astragals Radix.	0.24	0.24	0.23	0.23	0.23	0.22	0.22	0.24
Limit of aconitine	The spot in the chromatogram obtained with the test solution is not bigger than the spot in the chromatogram obtained with the reference solution, or there is no spot reveals in the chromotogram obtained with the test solution.	C*	C*	C*	C*	C*	C*	C*	C*
Conclusion	According to in-house standard of Qili Qiangxin Capsules, the product are stable within 36 months when stored in tightly sealed package at 30°C ± 2°C/RH:75% ± 5%								

C: Corresponded*

石家庄以岭药业股份有限公司
YILING SHIJIAZHUANGYILING PHARMACEUTICAL CO.,LTD
中国河北省石家庄市高新技术开发区天山大街 238 号 PC：050035
NO. 238, Tianshan Avenue, Hi-Tech Development Zone, Shijiazhuang, Hebei, China
TEL: 0086 311 85901719 FAX: 0086 311 85901719 http://www.yiling.cn

Stability Data

Table 1 LONG-TERM STABILITY DATA

Drug Product: Qili Qiangxin Capsules Package: blister and box Batch No: 100103
Mfg Date: January 5, 2010 Expiry date: December 2011 Temperature: 30°C± 2°C; RH = 75% ± 5%

Tests	Specification	Periods (months)							
		0	3	6	9	12	18	24	36
Description	It should be hard capsules, containing dark brownish to black-brownish granules; taste, bitter.	C*	C*	C*	C*	C*	C*	C*	C*
Identification	(1) HPLC The peaks in the chromatogram obtained with the test solution should correspond in the retention time to the peaks in the chromatogram obtained with the reference solution.	C*	C*	C*	C*	C*	C*	C*	C*
	(2) (3) (4) (5) TLC TLC of Salviae Miltiorrhizae Rhizoma et Radix reference drug, Periplocae Cortex reference drug, cinnamaldehyde CRS, hesperidin CRS	C*	C*	C*	C*	C*	C*	C*	C*
Filling variation	should be within ±10.0%	5.0-1.4	8.8-5.0	8.7-3.7	6.2-3.5	5.1-3.5	5.2-5.8	5.7-3.2	7.9-2.3
Water (per cent)	Not more than 9.0 %.	5.1	5.3	5.5	4.7	5.2	5.1	5	5.7
Disintegration (minute)	Should disintegrate within 30 minutes	9	7	6	7	6	6	7	7
Microbial limit	Should comply with the standard.<br>- Total Aerobic Microbial Count: NMT 10^3 CFU/g<br>- Total combined Yeast and Mold Count: NMT 10^2 CFU/g<br>- E.Coli: Absent/ g	<10<br><10<br>Absent	<10<br><10<br>Absent	<10<br><10<br>Absent	10<br><10<br>Absent	<10<br><10<br>Absent	20<br><10<br>Absent	<10<br><10<br>Absent	<10<br><10<br>Absent
Assay (mg)	It contains not less than 0.12 mg of astragaloside IV ($C_{41}H_{68}O_{14}$) per capsule, referred to Astragals Radix.	0.34	0.33	0.36	0.36	0.32	0.34	0.34	0.32
Limit of aconitine	The spot in the chromatogram obtained with the test solution is not bigger than the spot in the chromatogram obtained with the reference solution, or there is no spot reveals in the chromotogram obtained with the test solution.	C*	C*	C*	C*	C*	C*	C*	C*
Conclusion	According to in-house standard of Qili Qiangxin Capsules, the product are stable within 36 months when stored in tightly sealed package at 30°C ± 2°C/RH:75% ± 5%								

C: Corresponded*

中国河北省石家庄市高新技术开发区天山大街 238 号 PC：050035
NO. 238, Tianshan Avenue, Hi-Tech Development Zone, Shijiazhuang, Hebei, China
TEL: 0086 311 85901719 FAX: 0086 311 85901719 http://www.yiling.cn

Evaluation Report of Qili Qiangxin Capsules Long-term Stability Study

1. Objective

To investigate quality of Qili Qiangxin Capsules within the validity, by Comparing the test result of long-term retention sample with the results of 0 month sample.

2. Evaluation items

2.1 Description

Description was consistent with the requirements, within the validity (36 months).

2.2 Identification

Identification items of the batch were consistent with the requirements within 36 months, seen from stability log results.

2.3 Other requirements

2.3.1 Filling variation

Filling variation of the batch was consistent with the requirements within 36 months, seen from the results of stability log.

2.3.2 Water

Water of the batch was consistent with the requirements within 36 months, seen from the results of stability log.

2.3.3 Disintegration

Disintegration of the batch was consistent with the requirements within 36 months, seen from the results of stability log.

2.4 Microbial limit

Microbial limit of the batch was consistent with the requirements within 36 months, seen from the results of stability log.

2.5 Assay

Batch No.	100101	100102	100103
Range of content (mg/capsule)	0.25-0.35	0.22-0.24	0.32-0.36

Assay results of batch were consistent with the requirements within 36 months seen from the above data. The difference of results belongs to random error.

3. Conclusion

All items comply with the requirements within 36 months, seen from the above results. Therefore, it can be concluded that the quality of Qili Qiangxin Capsules is stable within validity.

中国河北省石家庄市高新技术开发区天山大街 238 号 PC：050035
No.238 Tianshan Street, High-Tech Area, Shijiazhuang Hebei Province, China.
TEL: 0086 311 85901719 FAX: 0086 311 85901719 http://www.yiling.cn

2.9 Container closure system

Primary packaging materials:

2.9.1 PTP aluminium foil

Manufacturer: Jiangsu Zhongjin Matai Medicinal Packaging Co., Ltd

Texture: PTP/aluminum

Standard No.: YBB00152002-2015 Pharmaceutical Packaging PTP Aluminum Foil

2.9.2 Pharmaceutical polyvinyl chloride (PVC) rigid film

Manufacturer: Chengde Yage Packing Technology Co., Ltd

Texture: polyvinyl chloride

Standard No.: YBB00212005-2015 Polyvinyl Chloride Solid Pharmaceutical Rigid Film.

Secondary packaging materials:

2.9.3 Pharmaceutical Packaging Compound Membrane

Manufacturer: Jiangsu Zhongjin Matai Medicinal Packaging Co., Ltd

Texture: Polyester, aluminum, polyethylene

Standard No.: YBB00172002 Polyester, aluminum, polyethylene pharmaceutical packaging compound membrane or pouch

2.9.4 Leaflet

Manufacturer: Shandong Luxin Tianyi Printing Co., Ltd.

Texture: 80 g two-side Copper paper

Standard No.: GB/7705-2008 Standard for Planography Upholstery Printed Matters

2.9.5 Big carton

Manufacturer: Shijiazhuang Hengtai Paper Co., Ltd.

Texture: corrugated case

Standard No.: GB/T-6543-2008 corrugated case standard

Selection criteria: select the above packaging materials for the product due to stability test of on marketing capsule package of Lianhua Qingwen capsules. In the accelerated stability test condition of 40℃±2℃ and 75%±5% in relative humidity, characteristics of the product is stable, inspection of description, identification, water, filling variation, disintegration, content and microbial limit are consistent with the specification; in the long term stability test condition of room temperature, inspection of description, identification, water, filling variation, disintegration, content and microbial test are consistent with the specification. According to above results, in the case of sealing tightly and store in dark place, this selected package can assure its quality stability and conformance.

芪苈强心胶囊36粒说明书13X18.5cm
版号:103B-36-201012

核准日期:2007年04月27日
修改日期:2020年12月30日

芪苈强心胶囊说明书

请仔细阅读说明书并在医师指导下使用

本品含黑顺片

【药品名称】
通用名称:芪苈强心胶囊
汉语拼音:Qili Qiangxin Jiaonang
【成　　份】黄芪、人参、黑顺片、丹参、葶苈子、泽泻、玉竹、桂枝、红花、香加皮、陈皮
【性　　状】本品为硬胶囊,内容物为棕褐色至黑褐色的颗粒;味苦。
【功能主治】益气温阳,活血通络,利水消肿。用于冠心病、高血压病所致轻、中度充血性心力衰竭证属阳气虚乏,络瘀水停证,症见心慌气短,动则加剧,夜间不能平卧,下肢浮肿,倦怠乏力,小便短少,口唇青紫,畏寒肢冷,咳吐稀白痰。
【规　　格】每粒装0.3g
【用法用量】口服。一次4粒,一日3次。
【不良反应】上市后监测数据显示本品可见以下胃肠道不良反应如恶心、胃不适、腹痛、腹泻、呕吐,以及皮疹、瘙痒等过敏反应。
【禁　　忌】对本品及本品成分过敏者忌服。
【注意事项】临床应用时,如果正在服用其它治疗心衰的药物,不宜突然停用。打开防潮袋后,请注意防潮。
【药理毒理】药效学试验表明,在戊巴比妥钠致犬实验性心力衰竭和腹主动脉结扎致家兔实验性慢性心力衰竭试验中,本品可使模型动物的心肌收缩力、心输出量和肾血流量增加,可使心室壁厚度和心脏指数降低,血管紧张素II和醛固酮水平降低,减轻心室重构。本品可增加大鼠排尿量。本品还可延长常压下小鼠的存活时间,延长小鼠低温游泳时间。
【贮　　藏】密封。
【包　　装】铝塑板装。3X12粒/板/盒。
【有 效 期】30个月
【执行标准】《中国药典》2020年版一部
【批准文号】国药准字Z20040141
【上市许可持有人】
名称:石家庄以岭药业股份有限公司
地址:石家庄市高新技术开发区天山大街238号
【生产企业】
企业名称:石家庄以岭药业股份有限公司
生产地址:石家庄市高新技术开发区天山大街238号
邮政编码:050035
电话号码:800 8038581(座机拨打)　400 7898989(手机/座机均可拨打)
　　　　　(0311)85901719
传真号码:(0311)85901719
注册地址:石家庄市高新技术开发区天山大街238号
网　　址:http://www.yilingshop.com

石家庄以岭药业股份有限公司

芪苈强心胶囊36粒 小盒 12.3X7.7X3cm
版号:103C-36-200507

【上市许可持有人】
名称:石家庄以岭药业股份有限公司
地址:石家庄市高新技术开发区天山大街238号
【生产企业】
企业名称:石家庄以岭药业股份有限公司
生产地址:石家庄市高新技术开发区天山大街238号 邮政编码:050035
电话号码:800 8038581(座机拨打) 400 7898989(手机/座机均可拨打) (0311) 85901719
传真号码:(0311) 85901719
注册地址:石家庄市高新技术开发区天山大街238号
网 址:http://www.yilingshop.com

6 920855 011515

【批准文号】国药准字Z20040141

以岭

36粒

石家庄以岭药业股份有限公司

【功能主治】益气温阳，活血通络，利水消肿。用于冠心病、高血压病所致轻、中度充血性心力衰竭证属阳气虚乏，络瘀水停证，症见心慌气短，动则加剧，夜间不能平卧，下肢浮肿，倦怠乏力，小便短少，口唇青紫，畏寒肢冷，咳吐稀白痰。

芪苈强心胶囊

请仔细阅读说明书并在医师指导下使用
【规　　格】每粒装0.3g
【不良反应】、【禁忌】、【注意事项】详见说明书。
【贮　　藏】密封。
【包　　装】铝塑板装。3X12粒/板/盒。

版号:103C-36-200507

码上放心·追溯码
请使用手机淘宝扫码查询
电话查询：010-95001111

扫一扫 辨真伪

【批准文号】国药准字Z20040141

以岭

QILI QIANGXIN JIAONANG

【成　　份】黄芪、人参、黑顺片、丹参、葶苈子、泽泻、玉竹、桂枝、红花、香加皮、陈皮
【性　　状】本品为硬胶囊，内容物为棕褐色至黑褐色的颗粒；味苦。
【用法用量】口服。一次4粒，一日3次。

石家庄以岭药业股份有限公司

芪苈强心胶囊

【生产日期】
【产品批号】
【有效期至】

国药准字Z20040141

芪苈强心胶囊

Qili Qiangxin Jiaonang

【规　　格】每粒装0.3g

【用法用量】口服。一次4粒，一日3次。

【产品批号】见铝塑板边缘

【有 效 期】30个月

石家庄以岭药业股份有限公司

杞蒡36粒复合膜18.2X13.5cm
版号：103F-36-170305

QL36

闷透，切薄片，干燥；或蒸半小时，取出，切薄片，干燥（注意避免暴晒）。

【性状】 本品为类圆形或不规则形薄片。外表皮黄棕色或棕褐色。切面黄棕色或黄绿色，具放射状纹理。

【含量测定】 同药材，含黄芩苷（$C_{21}H_{18}O_{11}$）不得少于8.0%。

【鉴别】 同药材。

酒黄芩 取黄芩片，照酒炙法（通则0213）炒干。

【性状】 本品形如黄芩片。略带焦斑，微有酒香气。

【含量测定】 同药材，含黄芩苷（$C_{21}H_{18}O_{11}$）不得少于8.0%。

【鉴别】 同药材。

【性味与归经】 苦，寒。归肺、胆、脾、大肠、小肠经。

【功能与主治】 清热燥湿，泻火解毒，止血，安胎。用于湿温、暑湿，胸闷呕恶，湿热痞满，泻痢，黄疸，肺热咳嗽，高热烦渴，血热吐衄，痈肿疮毒，胎动不安。

【用法与用量】 3～10g。

【贮藏】 置通风干燥处，防潮。

黄 芪

Huangqi

ASTRAGALI RADIX

本品为豆科植物蒙古黄芪 *Astragalus membranaceus* (Fisch.) Bge. var. *mongholicus* (Bge.) Hsiao 或膜荚黄芪 *Astragalus membranaceus* (Fisch.) Bge. 的干燥根。春、秋二季采挖，除去须根和根头，晒干。

【性状】 本品呈圆柱形，有的有分枝，上端较粗，长30～90cm，直径1～3.5cm。表面淡棕黄色或淡棕褐色，有不整齐的纵皱纹或纵沟。质硬而韧，不易折断，断面纤维性强，并显粉性，皮部黄白色，木部淡黄色，有放射状纹理和裂隙，老根中心偶呈枯朽状，黑褐色或呈空洞。气微，味微甜，嚼之微有豆腥味。

【鉴别】 (1)本品横切面：木栓细胞多列；栓内层为3～5列厚角细胞。韧皮部射线外侧常弯曲，有裂隙；纤维成束，壁厚，木化或微木化，与筛管群交互排列；近栓内层处有时可见石细胞。形成层成环。木质部导管单个散在或2～3个相聚；导管间有木纤维；射线中有时可见单个或2～4个成群的石细胞。薄壁细胞含淀粉粒。

粉末黄白色。纤维成束或散离，直径8～30μm，壁厚，表面有纵裂纹，初生壁常与次生壁分离，两端常断裂成须状，或较平截。具缘纹孔导管无色或橙黄色，具缘纹孔排列紧密。石细胞少见，圆形、长圆形或形状不规则，壁较厚。

(2)照薄层色谱法（通则0502）试验，吸取〔含量测定〕项下的供试品溶液及对照品溶液各5～10μl，分别点于同一硅胶G薄层板上，以三氯甲烷-甲醇-水（13：7：2）的下层溶液为展开剂，展开，取出，晾干，喷以10%硫酸乙醇溶液，在105℃加热至斑点显色清晰，分别置日光和紫外光灯（365nm）下检视。供试品色谱中，在与对照品色谱相应的位置上，日光下显相同的棕褐色斑点；紫外光（365nm）下显相同的橙黄色荧光斑点。

(3)取本品粉末2g，加乙醇30ml，加热回流20分钟，滤过，滤液蒸干，残渣加0.3%氢氧化钠溶液15ml使溶解，滤过，滤液用稀盐酸调节pH值至5～6，用乙酸乙酯15ml振摇提取，分取乙酸乙酯液，用铺有适量无水硫酸钠的滤纸滤过，滤液蒸干。残渣加乙酸乙酯1ml使溶解，作为供试品溶液。另取黄芪对照药材2g，同法制成对照药材溶液。照薄层色谱法（通则0502）试验，吸取上述两种溶液各10μl，分别点于同一硅胶G薄层板上，以三氯甲烷-甲醇（10：1）为展开剂，展开，取出，晾干，置氨蒸气中熏后，置紫外光灯（365nm）下检视。供试品色谱中，在与对照药材色谱相应的位置上，显相同颜色的荧光主斑点。

【检查】 **水分** 不得过10.0%（通则0832第二法）。

总灰分 不得过5.0%（通则2302）。

重金属及有害元素 照铅、镉、砷、汞、铜测定法（通则2321原子吸收分光光度法或电感耦合等离子体质谱法）测定，铅不得过5mg/kg；镉不得过1mg/kg；砷不得过2mg/kg；汞不得过0.2mg/kg；铜不得过20mg/kg。

其他有机氯类农药残留量 照农药残留量测定法（通则2341有机氯类农药残留量测定法—第一法）测定。

五氯硝基苯不得过0.1mg/kg。

【浸出物】 照水溶性浸出物测定法（通则2201）项下的冷浸法测定，不得少于17.0%。

【含量测定】 **黄芪甲苷** 照高效液相色谱法（通则0512）测定。

色谱条件与系统适用性试验 以十八烷基硅烷键合硅胶为填充剂；以乙腈-水（32：68）为流动相；蒸发光散射检测器检测。理论板数按黄芪甲苷峰计算应不低于4000。

对照品溶液的制备 取黄芪甲苷对照品适量，精密称定，加80%甲醇制成每1ml含0.5mg的溶液，即得。

供试品溶液的制备 取本品粉末（过四号筛）约1g，精密称定，置具塞锥形瓶中，精密加入含4%浓氨试液的80%甲醇溶液（取浓氨试液4ml，加80%甲醇至100ml，摇匀）50ml，密塞，称定重量，加热回流1小时，放冷，再称定重量，用含4%浓氨试液的80%甲醇溶液补足减失的重量，摇匀，滤过，精密量取续滤液25ml，蒸干，残渣用80%甲醇溶解，转移至5ml量瓶中，加80%甲醇至刻度，摇匀，滤过，取续滤液，即得。

测定法 分别精密吸取对照品溶液2μl（或5μl）、10μl，供试品溶液10～20μl，注入液相色谱仪，测定，以外标两点法对数方程计算，即得。

本品按干燥品计算，含黄芪甲苷（$C_{41}H_{68}O_{14}$）不得少于0.080%。

毛蕊异黄酮葡萄糖苷　照高效液相色谱法(通则 0512)测定。

色谱条件与系统适用性试验　以十八烷基硅烷键合硅胶为填充剂;以乙腈为流动相 A,以 0.2%甲酸溶液为流动相 B,按下表中的规定进行梯度洗脱;检测波长为 260nm。理论板数按毛蕊异黄酮葡萄糖苷峰计算应不低于 3000。

时间(分钟)	流动相 A(%)	流动相 B(%)
0～20	20→40	80→60
20～30	40	60

对照品溶液的制备　取毛蕊异黄酮葡萄糖苷对照品适量,精密称定,加甲醇制成每 1ml 含 50μg 的溶液,即得。

供试品溶液的制备　取本品粉末(过四号筛)约 1g,精密称定,置圆底烧瓶中,精密加入甲醇 50ml,称定重量,加热回流 4 小时,放冷,再称定重量,用甲醇补足减失的重量,摇匀,滤过,精密量取续滤液 25ml,回收溶剂至干,残渣加甲醇溶解,转移至 5ml 量瓶中,加甲醇至刻度,摇匀,即得。

测定法　分别精密吸取对照品溶液与供试品溶液各 10μl,注入液相色谱仪,测定,即得。

本品按干燥品计算,含毛蕊异黄酮葡萄糖苷($C_{22}H_{22}O_{10}$)不得少于 0.020%。

饮片

【炮制】　除去杂质,大小分开,洗净,润透,切厚片,干燥。

【性状】　本品呈类圆形或椭圆形的厚片,外表皮黄白色至淡棕褐色,可见纵皱纹或纵沟。切面皮部黄白色,木部淡黄色,有放射状纹理及裂隙,有的中心偶有枯朽状,黑褐色或呈空洞。气微,味微甜,嚼之有豆腥味。

【鉴别】(除横切面外)　**【检查】**　**【浸出物】**　**【含量测定】**　同药材。

【性味与归经】　甘,微温。归肺、脾经。

【功能与主治】　补气升阳,固表止汗,利水消肿,生津养血,行滞通痹,托毒排脓,敛疮生肌。用于气虚乏力,食少便溏,中气下陷,久泻脱肛,便血崩漏,表虚自汗,气虚水肿,内热消渴,血虚萎黄,半身不遂,痹痛麻木,痈疽难溃,久溃不敛。

【用法与用量】　9～30g。

【贮藏】　置通风干燥处,防潮,防蛀。

炙黄芪

Zhihuangqi

ASTRAGALI RADIX PRAEPARATA CUM MELLE

本品为黄芪的炮制加工品。

【炮制】　取黄芪片,照蜜炙法(通则 0213)炒至不粘手。

【性状】　本品呈圆形或椭圆形的厚片,直径 0.8～3.5cm,厚 0.1～0.4cm。外表皮淡棕黄色或淡棕褐色,略有光泽,可见纵皱纹或纵沟。切面皮部黄白色,木部淡黄色,有放射状纹理和裂隙,有的中心偶有枯朽状,黑褐色或呈空洞。具蜜香气,味甜,略带黏性,嚼之微有豆腥味。

【鉴别】　照黄芪项下的〔鉴别〕(2)、(3)试验,显相同的结果。

【检查】　**水分**　不得过 10.0%(通则 0832 第二法)。

总灰分　不得过 4.0%(通则 2302)。

【含量测定】　**黄芪甲苷**　取本品粉末(过四号筛)约 1g,精密称定,照黄芪〔含量测定〕项下的方法测定。

本品按干燥品计算,含黄芪甲苷($C_{41}H_{68}O_{14}$)不得少于 0.060%。

毛蕊异黄酮葡萄糖苷　取本品粉末(过四号筛)约 2g,精密称定,照黄芪〔含量测定〕项下的方法测定。

本品按干燥品计算,含毛蕊异黄酮葡萄糖苷($C_{22}H_{22}O_{10}$)不得少于 0.020%。

【性味与归经】　甘,温。归肺、脾经。

【功能与主治】　益气补中。用于气虚乏力,食少便溏。

【用法与用量】　9～30g。

【贮藏】　置通风干燥处,防潮,防蛀。

黄　连

Huanglian

COPTIDIS RHIZOMA

本品为毛茛科植物黄连 *Coptis chinensis* Franch.、三角叶黄连 *Coptis deltoidea* C. Y. Cheng et Hsiao 或云连 *Coptis teeta* Wall. 的干燥根茎。以上三种分别习称“味连”、“雅连”、“云连”。秋季采挖,除去须根和泥沙,干燥,撞去残留须根。

【性状】　**味连**　多集聚成簇,常弯曲,形如鸡爪,单枝根茎长 3～6cm,直径 0.3～0.8cm。表面灰黄色或黄褐色,粗糙,有不规则结节状隆起、须根及须根残基,有的节间表面平滑如茎秆,习称“过桥”。上部多残留褐色鳞叶,顶端常留有残余的茎或叶柄。质硬,断面不整齐,皮部橙红色或暗棕色,木部鲜黄色或橙黄色,呈放射状排列,髓部有的中空。气微,味极苦。

雅连　多为单枝,略呈圆柱形,微弯曲,长 4～8cm,直径 0.5～1cm。“过桥”较长。顶端有少许残茎。

云连　弯曲呈钩状,多为单枝,较细小。

【鉴别】　(1)本品横切面:味连　木栓层为数列细胞,其外有表皮,常脱落。皮层较宽,石细胞单个或成群散在。中柱鞘纤维成束或伴有少数石细胞,均显黄色。维管束外韧型,环列。木质部黄色,均木化,木纤维较发达。髓部均为薄壁细胞,无石细胞。

雅连　髓部有石细胞。

滤液 5ml，置 50ml 量瓶中，并用 60%冰醋酸溶液稀释至刻度，摇匀，精密量取各 1ml，分别置甲、乙两个试管中。于甲管中加新制的糠醛溶液 1ml，乙管中加水 1ml 作空白，照标准曲线的制备项下的方法，自“在冰浴中放置 5 分钟”起，依法测定吸光度。从标准曲线上读出供试品溶液中含胆酸的重量，计算，即得。

本品按干燥品计算，含胆酸（$C_{24}H_{40}O_5$）不得少于 80.0%。

〔用途〕 人工牛黄的原料。

〔贮藏〕 密闭保存。

5. 胆固醇质量标准

胆　固　醇

本品由牛、羊、猪脑经提取、加工制成。

〔性状〕 本品为白色、类白色结晶或结晶性粉末。气微。

熔点 本品的熔点不得低于 140℃（通则 0612）。

〔鉴别〕 （1）取本品 10mg，加三氯甲烷 1ml 使溶解，加硫酸 1ml，三氯甲烷层显血红色，硫酸层显绿色荧光。

（2）取本品约 5mg，加三氯甲烷 2ml 使溶解，加醋酐 1ml 与硫酸 1 滴，即显粉红色，立即成红色后变蓝色直至亮绿色。

〔检查〕 **醇溶度** 取本品 0.4g，加乙醇 50ml，温热使充分溶解，静置 2 小时，溶液应澄清并不得有沉淀产生。

酸度 取本品约 1g，精密称定，置锥形瓶中，加乙醚 10ml 使溶解，精密加 0.1mol/L 氢氧化钠溶液 10ml，振摇 1 分钟，缓缓加热，将乙醚除去，煮沸 5 分钟，放冷，加水 10ml 与酚酞指示液 2 滴，用硫酸滴定液（0.1mol/L）滴定至终点，并进行空白试验校正。供试品消耗量与空白试验消耗量之差不得过 0.5ml。

干燥失重 取本品，在 105℃干燥 3 小时，减失重量不得过 1.0%（通则 0831）。

炽灼残渣 取本品 1.0g，依法检查（通则 0841），残渣不得过 0.2%。

〔用途〕 人工牛黄的原料。

〔贮藏〕 密闭，避光。

人　参

Renshen

GINSENG RADIX ET RHIZOMA

本品为五加科植物人参 *Panax ginseng* C. A. Mey. 的干燥根和根茎。多于秋季采挖，洗净经晒干或烘干。栽培的俗称“园参”；播种在山林野生状态下自然生长的称“林下山参”，习称“籽海”。

【性状】 主根呈纺锤形或圆柱形，长 3～15cm，直径 1～2cm。表面灰黄色，上部或全体有疏浅断续的粗横纹及明显的纵皱，下部有支根 2～3 条，并着生多数细长的须根，须根上常有不明显的细小疣状突出。根茎（芦头）长 1～4cm，直径 0.3～1.5cm，多拘挛而弯曲，具不定根（艼）和稀疏的凹窝状茎痕（芦碗）。质较硬，断面淡黄白色，显粉性，形成层环纹棕黄色，皮部有黄棕色的点状树脂道及放射状裂隙。香气特异，味微苦、甘。

或主根多与根茎近等长或较短，呈圆柱形、菱角形或人字形，长 1～6cm。表面灰黄色，具纵皱纹，上部或中下部有环纹。支根多为 2～3 条，须根少而细长，清晰不乱，有较明显的疣状突起。根茎细长，少数粗短，中上部具稀疏或密集而深陷的茎痕。不定根较细，多下垂。

【鉴别】 （1）本品横切面：木栓层为数列细胞。栓内层窄。韧皮部外侧有裂隙，内侧薄壁细胞排列较紧密，有树脂道散在，内含黄色分泌物。形成层成环。木质部射线宽广，导管单个散在或数个相聚，断续排列成放射状，导管旁偶有非木化的纤维。薄壁细胞含草酸钙簇晶。

粉末淡黄白色。树脂道碎片易见，含黄色块状分泌物。草酸钙簇晶直径 20～68μm，棱角锐尖。木栓细胞表面观类方形或多角形，壁细波状弯曲。网纹导管和梯纹导管直径 10～56μm。淀粉粒甚多，单粒类球形、半圆形或不规则多角形，直径 4～20μm，脐点点状或裂缝状；复粒由 2～6 分粒组成。

（2）取本品粉末 1g，加三氯甲烷 40ml，加热回流 1 小时，弃去三氯甲烷液，药渣挥干溶剂，加水 0.5ml 搅拌湿润，加水饱和正丁醇 10ml，超声处理 30 分钟，吸取上清液加 3 倍量氨试液，摇匀，放置分层，取上层液蒸干，残渣加甲醇 1ml 使溶解，作为供试品溶液。另取人参对照药材 1g，同法制成对照药材溶液。再取人参皂苷 Rb_1 对照品、人参皂苷 Re 对照品、人参皂苷 Rf 对照品及人参皂苷 Rg_1 对照品，加甲醇制成每 1ml 各含 2mg 的混合溶液，作为对照品溶液。照薄层色谱法（通则 0502）试验，吸取上述三种溶液各 1～2μl，分别点于同一硅胶 G 薄层板上，以三氯甲烷-乙酸乙酯-甲醇-水（15∶40∶22∶10）10℃以下放置的下层溶液为展开剂，展开，取出，晾干，喷以 10%硫酸乙醇溶液，在 105℃加热至斑点显色清晰，分别置日光和紫外光灯（365nm）下检视。供试品色谱中，在与对照药材色谱和对照品色谱相应位置上，分别显相同颜色的斑点或荧光斑点。

【检查】 **水分** 不得过 12.0%（通则 0832 第二法）。

总灰分 不得过 5.0%（通则 2302）。

重金属及有害元素 照铅、镉、砷、汞、铜测定法（通则 2321 原子吸收分光光度法或电感耦合等离子体质谱法）测定，铅不得过 5mg/kg；镉不得过 1mg/kg；砷不得过 2mg/kg；汞不得过 0.2mg/kg；铜不得过 20mg/kg。

其他有机氯类农药残留量 照气相色谱法（通则 0521）测定。

色谱条件与系统适用性试验 分析柱：以键合交联 14%氰丙基苯基二甲基硅氧烷为固定液（DM1701 或同类型）的毛细管柱（30m×0.32mm×0.25μm），验证柱：以键合交联 5%苯基甲基硅氧烷为固定液（DB5 或同类型）的毛细管柱

(30m×0.32mm×0.25μm)；^{63}Ni-ECD 电子捕获检测器；进样口温度 230℃，检测器温度 300℃，不分流进样。程序升温：初始温度 60℃，保持 0.3 分钟，以每分钟 60℃升至 170℃，再以每分钟 10℃升至 220℃，保持 10 分钟，再以每分钟 1℃升至 240℃，再以每分钟 15℃升至 280℃，保持 5 分钟。理论板数按α-BHC峰计算应不低于 1×10^5，两个相邻色谱峰的分离度应大于 1.5。

混合对照品储备液的制备　分别精密称取五氯硝基苯、六氯苯、七氯(七氯、环氧七氯)、氯丹(顺式氯丹、反式氯丹、氧化氯丹)农药对照品适量，用正己烷溶解分别制成每 1ml 约含 100μg 的溶液。精密量取上述对照品溶液各 1ml，置同一 100ml 量瓶中，加正己烷至刻度，摇匀；或精密量取有机氯农药混合对照品溶液 1ml，置 10ml 量瓶中，加正己烷至刻度，摇匀，即得(每 1ml 含各农药对照品 1μg)。

混合对照品溶液的制备　精密量取上述混合对照品储备液，用正己烷制成每 1ml 分别含 1ng、2ng、5ng、10ng、20ng、50ng、100ng 的溶液，即得。

供试品溶液的制备　取本品，粉碎成细粉(过二号筛)，取约 5g，精密称定，置具塞锥形瓶中，加水 30ml，振摇 10 分钟，精密加丙酮 50ml，称定重量，超声处理(功率 300W，频率 40kHz)30 分钟，放冷，再称定重量，用丙酮补足减失的重量，再加氯化钠约 8g，精密加二氯甲烷 25ml，称定重量，超声处理(功率 300W，频率 40kHz)15 分钟，再称定重量，用二氯甲烷补足减失的重量，振摇使氯化钠充分溶解，静置，转移至离心管中，离心(每分钟 3000 转)3 分钟，使完全分层，将有机相转移至装有适量无水硫酸钠的具塞锥形瓶中，放置 30 分钟。精密量取 15ml，置 40℃水浴中减压浓缩至约 1ml，加正己烷约 5ml，减压浓缩至近干，用正己烷溶解并转移至 5ml 量瓶中，并稀释至刻度，摇匀，转移至离心管中，缓缓加入硫酸溶液(9→10)1ml，振摇 1 分钟，离心(每分钟 3000 转)10 分钟，分取上清液，加水 1ml，振摇，取上清液，即得。

测定法　分别精密吸取供试品溶液和与之相应浓度的混合对照品溶液各 1μl，注入气相色谱仪，分别连续进样 3 次，取 3 次平均值，按外标法计算，即得。

本品中含五氯硝基苯不得过 0.1mg/kg；六氯苯不得过 0.1mg/kg；七氯(七氯、环氧七氯之和)不得过 0.05mg/kg；氯丹(顺式氯丹、反式氯丹、氧化氯丹之和)不得过 0.1mg/kg。

【含量测定】　照高效液相色谱法(通则 0512)测定。

色谱条件与系统适用性试验　以十八烷基硅烷键合硅胶为填充剂；以乙腈为流动相 A，以水为流动相 B，按下表中的规定进行梯度洗脱；检测波长为 203nm。理论板数按人参皂苷 Rg_1 峰计算应不低于 6000。

时间(分钟)	流动相 A(%)	流动相 B(%)
0～35	19	81
35～55	19→29	81→71
55～70	29	71
70～100	29→40	71→60

对照品溶液的制备　精密称取人参皂苷 Rg_1 对照品、人参皂苷 Re 对照品及人参皂苷 Rb_1 对照品，加甲醇制成每 1ml 各含 0.2mg 的混合溶液，摇匀，即得。

供试品溶液的制备　取本品粉末(过四号筛)约 1g，精密称定，置索氏提取器中，加三氯甲烷加热回流 3 小时，弃去三氯甲烷液，药渣挥干溶剂，连同滤纸筒移入 100ml 锥形瓶中，精密加水饱和正丁醇 50ml，密塞，放置过夜，超声处理(功率 250W，频率 50kHz)30 分钟，滤过，弃去初滤液，精密量取续滤液 25ml，置蒸发皿中蒸干，残渣加甲醇溶解并转移至 5ml 量瓶中，加甲醇稀释至刻度，摇匀，滤过，取续滤液，即得。

测定法　分别精密吸取对照品溶液 10μl 与供试品溶液 10～20μl，注入液相色谱仪，测定，即得。

本品按干燥品计算，含人参皂苷Rg_1($C_{42}H_{72}O_{14}$)和人参皂苷 Re($C_{48}H_{82}O_{18}$)的总量不得少于 0.30%，人参皂苷 Rb_1($C_{54}H_{92}O_{23}$)不得少于 0.20%。

饮片

【炮制】　润透，切薄片，干燥，或用时粉碎、捣碎。

人参片　本品呈圆形或类圆形薄片。外表皮灰黄色。切面淡黄白色或类白色，显粉性，形成层环纹棕黄色，皮部有黄棕色的点状树脂道及放射性裂隙。体轻，质脆。香气特异，味微苦、甘。

【含量测定】　同药材，含人参皂苷 Rg_1($C_{42}H_{72}O_{14}$)和人参皂苷 Re($C_{48}H_{82}O_{18}$)的总量不得少于 0.27%，人参皂苷 Rb_1($C_{54}H_{92}O_{23}$)不得少于 0.18%。

【鉴别】(除横切面外)　**【检查】**　同药材。

【性味与归经】　甘、微苦，微温。归脾、肺、心、肾经。

【功能与主治】　大补元气，复脉固脱，补脾益肺，生津养血，安神益智。用于体虚欲脱，肢冷脉微，脾虚食少，肺虚喘咳，津伤口渴，内热消渴，气血亏虚，久病虚羸，惊悸失眠，阳痿宫冷。

【用法与用量】　3～9g，另煎兑服；也可研粉吞服，一次 2g，一日 2 次。

【注意】　不宜与藜芦、五灵脂同用。

【贮藏】　置阴凉干燥处，密闭保存，防蛀。

人参叶

Renshenye

GINSENG FOLIUM

本品为五加科植物人参 *Panax ginseng* C. A. Mey. 的干燥叶。秋季采收，晾干或烘干。

【性状】　本品常扎成小把，呈束状或扇状，长 12～35cm。掌状复叶带有长柄，暗绿色，3～6 枚轮生。小叶通常 5 枚，偶有 7 或 9 枚，呈卵形或倒卵形。基部的小叶长2～8cm，宽 1～4cm；上部的小叶大小相近，长 4～16cm，宽 2～7cm。基部楔

饮片

【炮制】 除去杂质，喷淋水，润透，切丝，干燥。

【性状】 本品呈不规则的条状或丝状。外表面橙红色或红棕色，有细皱纹和凹下的点状油室。内表面浅黄白色，粗糙，附黄白色或黄棕色筋络状维管束。气香，味辛、苦。

【含量测定】 **陈皮** 同药材，含橙皮苷($C_{28}H_{34}O_{15}$)不得少于2.5%。

广陈皮 同药材，含橙皮苷($C_{28}H_{34}O_{15}$)不得少于1.75%；含川陈皮素($C_{21}H_{22}O_8$)和橘皮素($C_{20}H_{20}O_7$)的总量，不得少于0.40%。

【鉴别】【检查】 同药材。

【性味与归经】 苦、辛，温。归肺、脾经。

【功能与主治】 理气健脾，燥湿化痰。用于脘腹胀满，食少吐泻，咳嗽痰多。

【用法与用量】 3～10g。

【贮藏】 置阴凉干燥处，防霉，防蛀。

注： 栽培变种主要有茶枝柑 *Citrus reticulata* 'Chachi'(广陈皮)、大红袍 *Citrus reticulata* 'Dahongpao'、温州蜜柑 *Citrus reticulata* 'Unshiu'、福橘 *Citrus reticulata* 'Tangerina'。

附　子

Fuzi

ACONITI LATERALIS RADIX PRAEPARATA

本品为毛茛科植物乌头 *Aconitum carmichaelii* Debx. 的子根的加工品。6月下旬至8月上旬采挖，除去母根、须根及泥沙，习称"泥附子"，加工成下列规格。

(1)选择个大、均匀的泥附子，洗净，浸入胆巴的水溶液中过夜，再加食盐，继续浸泡，每日取出晒晾，并逐渐延长晒晾时间，直至附子表面出现大量结晶盐粒(盐霜)、体质变硬为止，习称"盐附子"。

(2)取泥附子，按大小分别洗净，浸入胆巴的水溶液中数日，连同浸液煮至透心，捞出，水漂，纵切成厚约0.5cm的片，再用水浸漂，用调色液使附片染成浓茶色，取出，蒸至出现油面、光泽后，烘至半干，再晒干或继续烘干，习称"黑顺片"。

(3)选择大小均匀的泥附子，洗净，浸入胆巴的水溶液中数日，连同浸液煮至透心，捞出，剥去外皮，纵切成厚约0.3cm的片，用水浸漂，取出，蒸透，晒干，习称"白附片"。

【性状】 **盐附子** 呈圆锥形，长4～7cm，直径3～5cm。表面灰黑色，被盐霜，顶端有凹陷的芽痕，周围有瘤状突起的支根或支根痕。体重，横切面灰褐色，可见充满盐霜的小空隙和多角形形成层环纹，环纹内侧导管束排列不整齐。气微，味咸而麻，刺舌。

黑顺片 为纵切片，上宽下窄，长1.7～5cm，宽0.9～3cm，厚0.2～0.5cm。外皮黑褐色，切面暗黄色，油润具光泽，半透明状，并有纵向导管束。质硬而脆，断面角质样。气微，味淡。

白附片 无外皮，黄白色，半透明，厚约0.3cm。

【鉴别】 取本品粉末2g，加氨试液3ml润湿，加乙醚25ml，超声处理30分钟，滤过，滤液挥干，残渣加二氯甲烷0.5ml使溶解，作为供试品溶液。另取苯甲酰新乌头原碱对照品、苯甲酰乌头原碱对照品、苯甲酰次乌头原碱对照品，加异丙醇-二氯甲烷(1∶1)混合溶液制成每1ml各含1mg的混合溶液，作为对照品溶液(单酯型生物碱)。再取新乌头碱对照品、次乌头碱对照品、乌头碱对照品，加异丙醇-二氯甲烷(1∶1)混合溶液制成每1ml各含1mg的混合溶液，作为对照品溶液(双酯型生物碱)。照薄层色谱法(通则0502)试验，吸取供试品溶液和对照品溶液各5～10μl，分别点于同一硅胶G薄层板上，以正己烷-乙酸乙酯-甲醇(6.4∶3.6∶1)为展开剂，置氨蒸气饱和20分钟的展开缸内，展开，取出，晾干，喷以稀碘化铋钾试液。供试品色谱中，盐附子在与新乌头碱对照品、次乌头碱对照品和乌头碱对照品色谱相应的位置上，显相同颜色的斑点；黑顺片或白附片在与苯甲酰新乌头原碱对照品、苯甲酰乌头原碱对照品、苯甲酰次乌头原碱对照品色谱相应的位置上，显相同颜色的斑点。

【检查】 **水分** 不得过15.0%(通则0832第二法)。

双酯型生物碱 照〔含量测定〕项下色谱条件、供试品溶液的制备方法试验。

对照品溶液的制备　取新乌头碱对照品、次乌头碱对照品、乌头碱对照品适量，精密称定，加异丙醇-二氯甲烷(1∶1)混合溶液制成每1ml各含5μg的混合溶液，即得。

测定法　分别精密吸取上述对照品溶液与〔含量测定〕项下供试品溶液各10μl，注入液相色谱仪，测定，即得。

本品含双酯型生物碱以新乌头碱($C_{33}H_{45}NO_{11}$)、次乌头碱($C_{33}H_{45}NO_{10}$)和乌头碱($C_{34}H_{47}NO_{11}$)的总量计，不得过0.020%。

【含量测定】 照高效液相色谱法(通则0512)测定。

色谱条件与系统适用性试验 以十八烷基硅烷键合硅胶为填充剂；以乙腈-四氢呋喃(25∶15)为流动相A，以0.1mol/L醋酸铵溶液(每1000ml加冰醋酸0.5ml)为流动相B，按下表中的规定进行梯度洗脱，检测波长为235nm。理论板数按苯甲酰新乌头原碱峰计算应不低于3000。

时间(分钟)	流动相A(%)	流动相B(%)
0～48	15→26	85→74
48～49	26→35	74→65
49～58	35	65
58～65	35→15	65→85

对照品溶液的制备 取苯甲酰新乌头原碱对照品、苯甲酰乌头原碱对照品、苯甲酰次乌头原碱对照品适量，精密称定，加异丙醇-二氯甲烷(1∶1)混合溶液制成每1ml各含

10μg 的混合溶液，即得。

供试品溶液的制备 取本品粉末（过三号筛）约 2g，精密称定，置具塞锥形瓶中，加氨试液 3ml，精密加入异丙醇-乙酸乙酯（1∶1）混合溶液 50ml，称定重量，超声处理（功率 300W，频率 40kHz，水温在 25℃以下）30 分钟，放冷，再称定重量，用异丙醇-乙酸乙酯（1∶1）混合溶液补足减失的重量，摇匀，滤过。精密量取续滤液 25ml，40℃以下减压回收溶剂至干，残渣精密加入异丙醇-二氯甲烷（1∶1）混合溶液 3ml 溶解，滤过，取续滤液，即得。

测定法 分别精密吸取对照品溶液与供试品溶液各 10μl，注入液相色谱仪，测定，即得。

本品按干燥品计算，含苯甲酰新乌头原碱（$C_{31}H_{43}NO_{10}$）、苯甲酰乌头原碱（$C_{32}H_{45}NO_{10}$）和苯甲酰次乌头原碱（$C_{31}H_{43}NO_{9}$）的总量，不得少于 0.010%。

饮片

【炮制】 附片（黑顺片、白附片） 直接入药。

【检查】 **总灰分** 不得过 6.0%（通则 2302）。

酸不溶性灰分 不得过 1.0%（通则 2302）。

【性状】【鉴别】【检查】（水分 双酯型生物碱） **【含量测定】** 同药材。

淡附片 取盐附子，用清水浸漂，每日换水 2～3 次，至盐分漂尽，与甘草、黑豆加水共煮透心，至切开后口尝无麻舌感时，取出，除去甘草，黑豆，切薄片，晒干。

每 100kg 盐附子，用甘草 5kg、黑豆 10kg。

【性状】 本品呈纵切片，上宽下窄，长 1.7～5cm，宽 0.9～3cm，厚 0.2～0.5cm。外皮褐色。切面褐色，半透明，有纵向导管束。质硬，断面角质样。气微，味淡，口尝无麻舌感。

【检查】 **双酯型生物碱** 同药材，含双酯型生物碱以新乌头碱（$C_{33}H_{45}NO_{11}$）、次乌头碱（$C_{33}H_{45}NO_{10}$）和乌头碱（$C_{34}H_{47}NO_{11}$）的总量计，不得过 0.010%。

总灰分 不得过 7.0%（通则 2302）。

酸不溶性灰分 不得过 1.0%（通则 2302）。

【鉴别】【检查】（水分） **【含量测定】** 同药材。

炮附片 取附片，照炒法（通则 0213）用砂烫至鼓起并微变色。

【性状】 本品形如黑顺片或白附片，表面鼓起黄棕色，质松脆。气微，味淡。

【鉴别】【检查】 同附片。

【性味与归经】 辛、甘，大热；有毒。归心、肾、脾经。

【功能与主治】 回阳救逆，补火助阳，散寒止痛。用于亡阳虚脱，肢冷脉微，心阳不足，胸痹心痛，虚寒吐泻，脘腹冷痛，肾阳虚衰，阳痿宫冷，阴寒水肿，阳虚外感，寒湿痹痛。

【用法与用量】 3～15g，先煎，久煎。

【注意】 孕妇慎用；不宜与半夏、瓜蒌、瓜蒌子、瓜蒌皮、天花粉、川贝母、浙贝母、平贝母、伊贝母、湖北贝母、白蔹、白及同用。

【贮藏】 盐附子密闭，置阴凉干燥处；黑顺片及白附片置干燥处，防潮。

注：盐附子仅做〔性状〕检测。

忍冬藤
Rendongteng
LONICERAE JAPONICAE CAULIS

本品为忍冬科植物忍冬 *Lonicera japonica* Thunb. 的干燥茎枝。秋、冬二季采割，晒干。

【性状】 本品呈长圆柱形，多分枝，常缠绕成束，直径1.5～6mm。表面棕红色至暗棕色，有的灰绿色，光滑或被茸毛；外皮易剥落。枝上多节，节间长 6～9cm，有残叶和叶痕。质脆，易折断，断面黄白色，中空。气微，老枝味微苦，嫩枝味淡。

【鉴别】 （1）本品粉末浅棕黄色至黄棕色。非腺毛较多，单细胞，多断碎，壁厚，表面有疣状突起。表皮细胞棕黄色至棕红色，表面观类多角形，常有非腺毛脱落后的痕迹，石细胞状。薄壁细胞内含草酸钙簇晶，常排列成行，也有的单个散在，棱角较钝，直径 5～15μm。

（2）取本品粉末 1g，加 50%甲醇 10ml，超声处理 30 分钟，滤过，取滤液作为供试品溶液。另取忍冬藤对照药材 1g，同法制成对照药材溶液。再取马钱苷对照品，加 50%甲醇制成每 1ml 含 1mg 的溶液，作为对照品溶液。照薄层色谱法（通则 0502）试验，吸取供试品溶液和对照药材溶液各 10μl、对照品溶液 5μl，分别点于同一硅胶 G 薄层板上，以三氯甲烷-甲醇-水（65∶35∶10）10℃以下放置的下层溶液为展开剂，展开，取出，晾干，喷以 10%硫酸乙醇溶液，在 105℃加热至斑点显色清晰。供试品色谱中，在与对照药材色谱和对照品色谱相应的位置上，显相同颜色的斑点。

【检查】 **水分** 不得过 12.0%（通则 0832 第二法）。

总灰分 不得过 4.0%（通则 2302）。

【浸出物】 照醇溶性浸出物测定法（通则 2201）项下的热浸法测定，用 50%乙醇作溶剂，不得少于 14.0%。

【含量测定】 **绿原酸** 照高效液相色谱法（通则 0512）测定。

色谱条件与系统适用性试验 以十八烷基硅烷键合硅胶为填充剂；以乙腈-0.4%磷酸溶液（10∶90）为流动相；检测波长为 327nm。理论板数按绿原酸峰计算应不低于 1000。

对照品溶液的制备 取绿原酸对照品适量，精密称定，加 50%甲醇制成每 1ml 含 40μg 的溶液，即得。

供试品溶液的制备 取本品粉末（过三号筛）约 1g，精密称定，置具塞锥形瓶中，精密加入 50%甲醇 25ml，称定重量，超声处理（功率 250W，频率 30kHz）30 分钟，放冷，再称定重量，用 50%甲醇补足减失的重量，摇匀，滤过，取续滤液，

成每 1ml 含 1mg 的溶液，作为对照品溶液。照薄层色谱法（通则 0502）试验，吸取上述两种溶液各 2μl，分别点于同一高效硅胶 G 薄层板上，以乙酸乙酯-丙酮-冰醋酸-水（8∶4∶0.3∶1）为展开剂，展开，取出，晾干，喷以 5% 三氯化铝乙醇溶液，在 105℃加热 1 分钟，置紫外光灯（365nm）下检视。供试品色谱中，在与对照品色谱相应的位置上，显相同颜色的荧光斑点。

【检查】 **水分**　不得过 11.0%（通则 0832 第四法）。

总灰分　不得过 5.0%（通则 2302）。

【含量测定】 照高效液相色谱法（通则 0512）测定。

色谱条件与系统适用性试验　以十八烷基硅烷键合硅胶为填充剂；以甲醇-醋酸-水（35∶4∶61）为流动相；检测波长为 283nm。理论板数按柚皮苷峰计算应不低于 1000。

对照品溶液的制备　取柚皮苷对照品适量，精密称定，加甲醇制成每 1ml 含 60μg 的溶液，即得。

供试品溶液的制备　取本品粉末（过二号筛）约 0.5g，精密称定，置具塞锥形瓶中，精密加入甲醇 50ml，称定重量，水浴加热回流 1 小时，放冷，再称定重量，用甲醇补足减失的重量，摇匀，滤过，精密量取续滤液 5ml，置 50ml 量瓶中，加 50% 甲醇至刻度，摇匀，即得。

测定法　分别精密吸取对照品溶液与供试品溶液各 10μl，注入液相色谱仪，测定，即得。

本品按干燥品计算，含柚皮苷（$C_{27}H_{32}O_{14}$）不得少于 3.5%。

饮片

【炮制】 除去杂质，洗净，闷润，切丝或块，晒干。

【性味与归经】 辛、苦，温。归肺、脾经。

【功能与主治】 理气宽中，燥湿化痰。用于咳嗽痰多，食积伤酒，呕恶痞闷。

【用法与用量】 3～6g。

【贮藏】 置阴凉干燥处，防蛀。

月　季　花

Yuejihua

ROSAE CHINENSIS FLOS

本品为蔷薇科植物月季 *Rosa chinensis* Jacq. 的干燥花。全年均可采收，花微开时采摘，阴干或低温干燥。

【性状】 本品呈类球形，直径 1.5～2.5cm。花托长圆形，萼片 5，暗绿色，先端尾尖；花瓣呈覆瓦状排列，有的散落，长圆形，紫红色或淡紫红色；雄蕊多数，黄色。体轻，质脆。气清香，味淡、微苦。

【鉴别】 （1）本品粉末淡棕色。单细胞非腺毛有两种：一种较细长，多弯曲，长 85～280μm，直径 13～23μm；另一种粗长，先端尖或钝圆，长约至 1200μm，直径 38～65μm。花粉粒类球形，直径 30～45μm，具 3 孔沟，表面有细密点状雕纹，有的中心有一圆形核状物。草酸钙簇晶直径 19～40μm，棱角较短尖。花瓣上表皮细胞外壁突起，有细密脑纹状纹理；下表皮细胞垂周壁波状弯曲。

（2）取本品粉末 1g，加 70% 甲醇 20ml，超声处理 40 分钟，滤过，取滤液作为供试品溶液。另取金丝桃苷对照品、异槲皮苷对照品，加甲醇制成每 1ml 各含 0.4mg 的混合溶液，作为对照品溶液。照薄层色谱法（通则 0502）试验，吸取上述两种溶液各 1μl，分别点于同一硅胶 G 薄层板上，以乙酸乙酯-甲酸-水（15∶1∶1）为展开剂，展开，取出，晾干，喷以 10% 硫酸乙醇溶液，在 105℃加热数分钟，立即置紫外光灯（365nm）下检视。供试品色谱中，在与对照品色谱相应的位置上，显相同颜色的荧光斑点。

【检查】 **水分**　不得过 12.0%（通则 0832 第二法）。

总灰分　不得过 5.0%（通则 2302）。

【含量测定】 照高效液相色谱法（通则 0512）测定。

色谱条件与系统适用性试验　以十八烷基硅烷键合硅胶为填充剂；以乙腈-0.1% 甲酸溶液（15∶85）为流动相；检测波长为 354nm。理论板数按金丝桃苷峰计算应不低于 3000。

对照品溶液的制备　取金丝桃苷对照品、异槲皮苷对照品适量，精密称定，加 50% 甲醇制成每 1ml 各含 20μg 的混合溶液，即得。

供试品溶液的制备　取本品粉末（过四号筛）约 0.2g，精密称定，置具塞锥形瓶中，精密加入 50% 甲醇 25ml，密塞，称定重量，加热回流 1 小时，放冷，再称定重量，用 50% 甲醇补足减失的重量，摇匀，滤过，取续滤液，即得。

测定法　分别精密吸取对照品溶液与供试品溶液各 20μl，注入液相色谱仪，测定，即得。

本品按干燥品计算，含金丝桃苷（$C_{21}H_{20}O_{12}$）和异槲皮苷（$C_{21}H_{20}O_{12}$）的总量不得少于 0.38%。

【性味与归经】 甘，温。归肝经。

【功能与主治】 活血调经，疏肝解郁。用于气滞血瘀，月经不调，痛经，闭经，胸胁胀痛。

【用法与用量】 3～6g。

【贮藏】 置阴凉干燥处，防压、防蛀。

丹　参

Danshen

SALVIAE MILTIORRHIZAE RADIX ET RHIZOMA

本品为唇形科植物丹参 *Salvia miltiorrhiza* Bge. 的干燥根和根茎。春、秋二季采挖，除去泥沙，干燥。

【性状】 本品根茎短粗，顶端有时残留茎基。根数条，长圆柱形，略弯曲，有的分枝并具须状细根，长 10～20cm，直径

0.3～1cm。表面棕红色或暗棕红色，粗糙，具纵皱纹。老根外皮疏松，多显紫棕色，常呈鳞片状剥落。质硬而脆，断面疏松，有裂隙或略平整而致密，皮部棕红色，木部灰黄色或紫褐色，导管束黄白色，呈放射状排列。气微，味微苦涩。

栽培品较粗壮，直径 0.5～1.5cm。表面红棕色，具纵皱纹，外皮紧贴不易剥落。质坚实，断面较平整，略呈角质样。

【鉴别】 (1)本品粉末红棕色。石细胞类圆形、类三角形、类长方形或不规则形，也有延长呈纤维状，边缘不平整，直径 14～70μm，长可达 257μm，孔沟明显，有的胞腔内含黄棕色物。木纤维多为纤维管胞，长梭形，末端斜尖或钝圆，直径 12～27μm，具缘纹孔点状，纹孔斜裂缝状或十字形，孔沟稀疏。网纹导管和具缘纹孔导管直径 11～60μm。

(2)取本品粉末 1g，加乙醇 5ml，超声处理 15 分钟，离心，取上清液作为供试品溶液。另取丹参对照药材 1g，同法制成对照药材溶液。再取丹参酮 $Ⅱ_A$ 对照品、丹酚酸 B 对照品，加乙醇制成每 1ml 分别含 0.5mg 和 1.5mg 的混合溶液，作为对照品溶液。照薄层色谱法(通则 0502)试验，吸取上述三种溶液各 5μl，分别点于同一硅胶 G 薄层板上，使成条状，以三氯甲烷-甲苯-乙酸乙酯-甲醇-甲酸(6：4：8：1：4)为展开剂，展开，展至约 4cm，取出，晾干，再以石油醚(60～90℃)-乙酸乙酯(4：1)为展开剂，展开，展至约 8cm，取出，晾干，分别置日光及紫外光灯(365nm)下检视。供试品色谱中，在与对照药材色谱和对照品色谱相应的位置上，显相同颜色的斑点或荧光斑点。

【检查】 **水分** 不得过 13.0%(通则 0832 第二法)。

总灰分 不得过 10.0%(通则 2302)。

酸不溶性灰分 不得过 3.0%(通则 2302)。

重金属及有害元素 照铅、镉、砷、汞、铜测定法(通则 2321 原子吸收分光光度法或电感耦合等离子体质谱法)测定，铅不得过 5mg/kg；镉不得过 1mg/kg；砷不得过 2mg/kg；汞不得过 0.2mg/kg；铜不得过 20mg/kg。

【浸出物】 **水溶性浸出物** 照水溶性浸出物测定法(通则 2201)项下的冷浸法测定，不得少于 35.0%。

醇溶性浸出物 照醇溶性浸出物测定法(通则 2201)项下的热浸法测定，用乙醇作溶剂，不得少于 15.0%。

【含量测定】 **丹参酮类** 照高效液相色谱法(通则 0512)测定。

色谱条件与系统适用性试验　以十八烷基硅烷键合硅胶为填充剂；以乙腈为流动相 A，以 0.02%磷酸溶液为流动相 B，按下表中的规定进行梯度洗脱；柱温为 20℃；检测波长为 270nm。理论板数按丹参酮 $Ⅱ_A$ 峰计算应不低于 60000。

时间(分钟)	流动相 A(%)	流动相 B(%)
0～6	61	39
6～20	61→90	39→10
20～20.5	90→61	10→39
20.5～25	61	39

对照品溶液的制备　取丹参酮 $Ⅱ_A$ 对照品适量，精密称定，置棕色量瓶中，加甲醇制成每 1ml 含 20μg 的溶液，即得。

供试品溶液的制备　取本品粉末(过三号筛)约 0.3g，精密称定，置具塞锥形瓶中，精密加入甲醇 50ml，密塞，称定重量，超声处理(功率 140W，频率 42kHz)30 分钟，放冷，再称定重量，用甲醇补足减失的重量，摇匀，滤过，取续滤液，即得。

测定法　分别精密吸取对照品溶液与供试品溶液各 10μl，注入液相色谱仪，测定。以丹参酮 $Ⅱ_A$ 对照品为参照，以其相应的峰为 S 峰，计算隐丹参酮、丹参酮Ⅰ的相对保留时间，其相对保留时间应在规定值的±5%范围之内。相对保留时间及校正因子见下表。

待测成分(峰)	相对保留时间	校正因子
隐丹参酮	0.75	1.18
丹参酮Ⅰ	0.79	1.31
丹参酮 $Ⅱ_A$	1.00	1.00

以丹参酮 $Ⅱ_A$ 的峰面积为对照，分别乘以校正因子，计算隐丹参酮、丹参酮Ⅰ、丹参酮 $Ⅱ_A$ 的含量。

本品按干燥品计算，含丹参酮 $Ⅱ_A$($C_{19}H_{18}O_3$)、隐丹参酮($C_{19}H_{20}O_3$)和丹参酮Ⅰ($C_{18}H_{12}O_3$)的总量不得少于 0.25%。

丹酚酸 B 照高效液相色谱法(通则 0512)测定。

色谱条件与系统适用性试验　以十八烷基硅烷键合硅胶为填充剂；以乙腈-0.1%磷酸溶液(22：78)为流动相；柱温为 20℃；流速为每分钟 1.2ml；检测波长为 286nm。理论板数按丹酚酸 B 峰计算应不低于 6000。

对照品溶液的制备　取丹酚酸 B 对照品适量，精密称定，加甲醇-水(8：2)混合溶液制成每 1ml 含 0.10mg 的溶液，即得。

供试品溶液的制备　取本品粉末(过三号筛)约 0.15g，精密称定，置具塞锥形瓶中，精密加入甲醇-水(8：2)混合溶液 50ml，密塞，称定重量，超声处理(功率 140W，频率 42kHz)30 分钟，放冷，再称定重量，用甲醇-水(8：2)混合溶液补足减失的重量，摇匀，滤过，精密量取续滤液 5ml，移至 10ml 量瓶中，加甲醇-水(8：2)混合溶液稀释至刻度，摇匀，滤过，取续滤液，即得。

测定法　分别精密吸取对照品溶液与供试品溶液各 10μl，注入液相色谱仪，测定，即得。

本品按干燥品计算，含丹酚酸 B($C_{36}H_{30}O_{16}$)不得少于 3.0%。

饮片

【炮制】 **丹参** 除去杂质和残茎，洗净，润透，切厚片，干燥。

【性状】 本品呈类圆形或椭圆形的厚片。外表皮棕红色或暗棕红色，粗糙，具纵皱纹。切面有裂隙或略平整而致密，有的呈角质样，皮部棕红色，木部灰黄色或紫褐色，有黄白色放射状纹理。气微，味微苦涩。

【检查】 **酸不溶性灰分** 同药材，不得过 2.0%(通

则 2302)。

【浸出物】 **醇溶性浸出物** 同药材,不得少于 11.0%。

【鉴别】【检查】(水分 总灰分) **【浸出物】**(水溶性浸出物) 同药材。

酒丹参 取丹参片,照酒炙法(通则 0213)炒干。

【性状】 本品形如丹参片,表面红褐色,略具酒香气。

【检查】 **水分** 同药材,不得过 10.0%(通则 0832 第二法)。

【浸出物】 **醇溶性浸出物** 同药材,不得少于 11.0%。

【鉴别】【检查】(总灰分) **【浸出物】**(水溶性浸出物) 同药材。

【性味与归经】 苦,微寒。归心、肝经。

【功能与主治】 活血祛瘀,通经止痛,清心除烦,凉血消痈。用于胸痹心痛,脘腹胁痛,癥瘕积聚,热痹疼痛,心烦不眠,月经不调,痛经经闭,疮疡肿痛。

【用法与用量】 10~15g。

【注意】 不宜与藜芦同用。

【贮藏】 置干燥处。

乌 药

Wuyao

LINDERAE RADIX

本品为樟科植物乌药 *Lindera aggregata* (Sims) Kosterm. 的干燥块根。全年均可采挖,除去细根,洗净,趁鲜切片,晒干,或直接晒干。

【性状】 本品多呈纺锤状,略弯曲,有的中部收缩成连珠状,长 6~15cm,直径 1~3cm。表面黄棕色或黄褐色,有纵皱纹及稀疏的细根痕。质坚硬。切片厚 0.2~2mm,切面黄白色或淡黄棕色,射线放射状,可见年轮环纹,中心颜色较深。气香,味微苦、辛,有清凉感。

质老、不呈纺锤状的直根,不可供药用。

【鉴别】 (1)本品粉末黄白色。淀粉粒甚多,单粒类球形、长圆形或卵圆形,直径 4~39μm,脐点叉状、人字状或裂缝状;复粒由 2~4 分粒组成。木纤维淡黄色,多成束,直径 20~30μm,壁厚约 5μm,有单纹孔,胞腔含淀粉粒。韧皮纤维近无色,长梭形,多单个散在,直径 15~17μm,壁极厚,孔沟不明显。具缘纹孔导管直径约至 68μm,具缘纹孔排列紧密。木射线细胞壁稍增厚,纹孔较密。油细胞长圆形,含棕色分泌物。

(2)取本品粉末 1g,加石油醚(30~60℃)30ml,放置 30 分钟,超声处理(保持水温低于 30℃)10 分钟,滤过,滤液挥干,残渣加乙酸乙酯 1ml 使溶解,作为供试品溶液。另取乌药对照药材 1g,同法制成对照药材溶液。再取乌药醚内酯对照品,用乙酸乙酯溶解,制成每 1ml 含 0.75mg 的溶液,作为对照品溶液。照薄层色谱法(通则 0502)试验,吸取供试品溶液 4μl、对照药材溶液 4μl、对照品溶液 3μl,分别点于同一硅胶 H 薄层板上,以甲苯-乙酸乙酯(15∶1)为展开剂,展开,取出,晾干,喷以 1% 香草醛硫酸溶液。供试品色谱中,在与对照药材色谱和对照品色谱相应的位置上,显相同颜色的斑点。

【检查】 **水分** 不得过 11.0%(通则 0832 第四法)。

总灰分 不得过 4.0%(通则 2302)。

酸不溶性灰分 不得过 2.0%(通则 2302)。

【浸出物】 照醇溶性浸出物测定法(通则 2201)项下的热浸法测定,用 70% 乙醇作溶剂,不得少于 12.0%。

【含量测定】 **乌药醚内酯** 照高效液相色谱法(通则 0512)测定。

色谱条件与系统适用性试验 以十八烷基硅烷键合硅胶为填充剂;以乙腈-水(56∶44)为流动相;检测波长为 235nm。理论板数按乌药醚内酯峰计算应不低于 2000。

对照品溶液的制备 取乌药醚内酯对照品 10mg,精密称定,置 100ml 量瓶中,用甲醇溶解并稀释至刻度,摇匀,精密量取 10ml,置 25ml 量瓶中,加甲醇至刻度,摇匀,即得(每 1ml 中含乌药醚内酯 40μg)。

供试品溶液的制备 取本品粗粉约 1g,精密称定,置索氏提取器中,加乙醚 50ml,提取 4 小时,提取液挥干,残渣用甲醇分次溶解,转移至 50ml 量瓶中,加甲醇至刻度,摇匀,滤过,取续滤液,即得。

测定法 分别精密吸取对照品溶液与供试品溶液各 10μl,注入液相色谱仪,测定,即得。

本品按干燥品计算,含乌药醚内酯($C_{15}H_{16}O_4$)不得少于 0.030%。

去甲异波尔定 照高效液相色谱法(通则 0512)测定。

色谱条件与系统适用性试验 以十八烷基硅烷键合硅胶为填充剂;以乙腈为流动相 A,以含 0.5% 甲酸和 0.1% 三乙胺溶液为流动相 B,按下表中的规定进行梯度洗脱;检测波长为 280nm。理论板数按去甲异波尔定峰计算应不低于 5000。

时间(分钟)	流动相 A(%)	流动相 B(%)
0~13	10→22	90→78
13~22	22	78

对照品溶液的制备 取去甲异波尔定对照品适量,精密称定,加甲醇-盐酸溶液(0.5→100)(2∶1)的混合溶液制成每 1ml 含 0.2mg 的溶液,即得。

供试品溶液的制备 取本品粉末(过三号筛)约 0.5g,精密称定,置圆底烧瓶中,精密加入甲醇-盐酸溶液(0.5→100)(2∶1)的混合溶液 25ml,密塞,称定重量,加热回流并保持微沸 1 小时,放冷,再称定重量,用甲醇-盐酸溶液(0.5→100)(2∶1)的混合溶液补足减失的重量,摇匀,滤过,取续滤液,即得。

葶　苈　子

Tinglizi

DESCURAINIAE SEMEN
LEPIDII SEMEN

本品为十字花科植物播娘蒿 *Descurainia sophia* (L.) Webb. ex Prantl. 或独行菜 *Lepidium apetalum* Willd. 的干燥成熟种子。前者习称“南葶苈子”，后者习称“北葶苈子”。夏季果实成熟时采割植株，晒干，搓出种子，除去杂质。

【性状】 **南葶苈子** 呈长圆形略扁，长约 0.8～1.2mm，宽约 0.5mm。表面棕色或红棕色，微有光泽，具纵沟 2 条，其中 1 条较明显。一端钝圆，另端微凹或较平截，种脐类白色，位于凹入端或平截处。气微，味微辛、苦，略带黏性。

北葶苈子 呈扁卵形，长 1～1.5mm，宽0.5～1mm。一端钝圆，另端尖而微凹，种脐位于凹入端。味微辛辣，黏性较强。

【鉴别】 (1)取本品少量，加水浸泡后，用放大镜观察，南葶苈子透明状黏液层薄，厚度约为种子宽度的 1/5 以下。北葶苈子透明状黏液层较厚，厚度可超过种子宽度的 1/2 以上。

(2)南葶苈子　粉末黄棕色。种皮外表皮细胞为黏液细胞，断面观类方形，内壁增厚向外延伸成纤维素柱，纤维素柱长 8～18μm，顶端钝圆、偏斜或平截，周围可见黏液质纹理。种皮内表皮细胞为黄色，表面观呈长方多角形，直径15～42μm，壁厚 5～8μm。

北葶苈子　种皮外表皮细胞断面观略呈类长方形，纤维素柱较长，长 24～34μm，种皮内表皮细胞表面观长方多角形或类方形。

(3)南葶苈子　取本品粉末 1g，加 70%甲醇 20ml，加热回流 1 小时，滤过，取滤液作为供试品溶液。另取槲皮素-3-*O*-*β*-D-葡萄糖-7-*O*-*β*-D-龙胆双糖苷对照品，加 30%甲醇制成每 1ml 含 90μg 的溶液，作为对照品溶液。照薄层色谱法(通则 0502)试验，吸取上述两种溶液各 1μl，分别点于同一聚酰胺薄膜上，以乙酸乙酯-甲醇-水(7∶2∶1)为展开剂，展开，取出，晾干，喷以 2%三氯化铝乙醇溶液，热风吹干，置紫外光灯(365nm)下检视。供试品色谱中，在与对照品色谱相应的位置上，显相同的黄色荧光斑点。

【检查】 **水分** 不得过 9.0%(通则 0832 第二法)。

总灰分 不得过 8.0%(通则 2302)。

酸不溶性灰分 不得过 3.0%(通则 2302)。

膨胀度 取本品 0.6g，称定重量，照膨胀度测定法(通则 2101)测定。南葶苈子不得低于 3，北葶苈子不得低于 12。

【含量测定】 **南葶苈子** 照高效液相色谱法(通则 0512)测定。

色谱条件与系统适用性试验　以十八烷基硅烷键合硅胶为填充剂；以乙腈-0.1%醋酸溶液(11∶89)为流动相；检测波长为 254nm。理论板数按槲皮素-3-*O*-*β*-D-葡萄糖-7-*O*-*β*-D-龙胆双糖苷峰计算应不低于 5800。

对照品溶液的制备　取槲皮素-3-*O*-*β*-D-葡萄糖-7-*O*-*β*-D-龙胆双糖苷对照品适量，精密称定，加 30%甲醇制成每 1ml 含 20μg 的溶液，即得。

供试品溶液的制备　取本品粉末(过四号筛)约 1g，精密称定，置具塞锥形瓶中，精密加入 70%甲醇 50ml，密塞，称定重量，加热回流 1 小时，放冷，再称定重量，用 70%甲醇补足减失的重量，摇匀，滤过，取续滤液，即得。

测定法　分别精密吸取对照品溶液与供试品溶液各 25μl，注入液相色谱仪，测定，即得。

本品按干燥品计算，含槲皮素-3-*O*-*β*-D-葡萄糖-7-*O*-*β*-D-龙胆双糖苷($C_{33}H_{40}O_{22}$)不得少于 0.075%。

饮片

【炮制】 **葶苈子** 除去杂质和灰屑。

【性状】【鉴别】【检查】【含量测定】 同药材。

炒葶苈子 取净葶苈子，照清炒法(通则 0213)炒至有爆声。

【性状】 本品形如葶苈子，微鼓起，表面棕黄色。有油香气，不带黏性。

【检查】 **水分** 同药材，不得过 5.0%。

【含量测定】 **南葶苈子** 同药材，含槲皮素-3-*O*-*β*-D-葡萄糖-7-*O*-*β*-D-龙胆双糖苷($C_{33}H_{40}O_{22}$)不得少于 0.080%。

【鉴别】【检查】(总灰分　酸不溶性灰分)　同药材。

【性味与归经】 辛、苦，大寒。归肺、膀胱经。

【功能与主治】 泻肺平喘，行水消肿。用于痰涎壅肺，喘咳痰多，胸胁胀满，不得平卧，胸腹水肿，小便不利。

【用法与用量】 3～10g，包煎。

【贮藏】 置干燥处。

萹　蓄

Bianxu

POLYGONI AVICULARIS HERBA

本品为蓼科植物萹蓄 *Polygonum aviculare* L. 的干燥地上部分。夏季叶茂盛时采收，除去根和杂质，晒干。

【性状】 本品茎呈圆柱形而略扁，有分枝，长 15～40cm，直径 0.2～0.3cm。表面灰绿色或棕红色，有细密微突起的纵纹；节部稍膨大，有浅棕色膜质的托叶鞘，节间长约 3cm；质硬，易折断，断面髓部白色。叶互生，近无柄或具短柄，叶片多脱落或皱缩、破碎，完整者展平后呈披针形，全缘，两面均呈棕绿色或灰绿色。气微，味微苦。

【鉴别】 (1)本品茎横切面：表皮细胞 1 列，长方形，外

泽　兰

Zelan

LYCOPI HERBA

本品为唇形科植物毛叶地瓜儿苗 *Lycopus lucidus* Turcz. var. *hirtus* Regel 的干燥地上部分。夏、秋二季茎叶茂盛时采割，晒干。

【性状】 本品茎呈方柱形，少分枝，四面均有浅纵沟，长 50～100cm，直径 0.2～0.6cm；表面黄绿色或带紫色，节处紫色明显，有白色茸毛；质脆，断面黄白色，髓部中空。叶对生，有短柄或近无柄；叶片多皱缩，展平后呈披针形或长圆形，长 5～10cm；上表面黑绿色或暗绿色，下表面灰绿色，密具腺点，两面均有短毛；先端尖，基部渐狭，边缘有锯齿。轮伞花序腋生，花冠多脱落，苞片和花萼宿存，小包片披针形，有缘毛，花萼钟形，5 齿。气微，味淡。

【鉴别】 （1）叶表面观：上表皮细胞垂周壁近平直，非腺毛较多，由 1～5 细胞组成，表面有疣状突起。下表皮细胞垂周壁波状弯曲，角质线纹明显，气孔直轴式，主脉和侧脉上非腺毛较多，由 3～6 细胞组成，表面有疣状突起。腺鳞头部类圆形，8 细胞，直径 66～83μm。

（2）取本品粉末 1g，加丙酮 30ml，加热回流 30 分钟，滤过，滤液蒸干，残渣加石油醚（30～60℃）10ml，浸泡约 2 分钟，倾去石油醚液，蒸干，残渣加无水乙醇 2ml 使溶解，作为供试品溶液。另取熊果酸对照品，加无水乙醇制成每 1ml 含 0.5mg 的溶液，作为对照品溶液。照薄层色谱法（通则 0502）试验，吸取供试品溶液 2～4μl、对照品溶液 2μl，分别点于同一硅胶 G 薄层板上，以环己烷-三氯甲烷-乙酸乙酯-甲酸（20：5：8：0.1）为展开剂，展开，取出，晾干，喷以 10%硫酸乙醇溶液，在 105℃加热至斑点显色清晰。供试品色谱中，在与对照品色谱相应的位置上，显相同颜色的斑点。

【检查】 **水分** 不得过 13.0%（通则 0832 第二法）。

总灰分 不得过 10.0%（通则 2302）。

【浸出物】 照醇溶性浸出物测定法（通则 2201）项下的热浸法测定，用乙醇作溶剂，不得少于 7.0%。

饮片

【炮制】 除去杂质，略洗，润透，切段，干燥。

【性状】 本品呈不规则的段。茎方柱形，四面均有浅纵沟，表面黄绿色或带紫色，节处紫色明显，有白色茸毛。切面黄白色，中空。叶多破碎，展平后呈披针形或长圆形，边缘有锯齿。有时可见轮伞花序。气微，味淡。

【鉴别】【检查】【浸出物】 同药材。

【性味与归经】 苦、辛，微温。归肝、脾经。

【功能与主治】 活血调经，祛瘀消痈，利水消肿。用于月经不调，经闭，痛经，产后瘀血腹痛，疮痈肿毒，水肿腹水。

【用法与用量】 6～12g。

【贮藏】 置通风干燥处。

泽　泻

Zexie

ALISMATIS RHIZOMA

本品为泽泻科植物东方泽泻 *Alisma orientale*（Sam.）Juzep. 或泽泻 *Alisma plantago-aquatica* Linn. 的干燥块茎。冬季茎叶开始枯萎时采挖，洗净，干燥，除去须根和粗皮。

【性状】 本品呈类球形、椭圆形或卵圆形，长 2～7cm，直径 2～6cm。表面淡黄色至淡黄棕色，有不规则的横向环状浅沟纹和多数细小突起的须根痕，底部有的有瘤状芽痕。质坚实，断面黄白色，粉性，有多数细孔。气微，味微苦。

【鉴别】 （1）本品粉末淡黄棕色。淀粉粒甚多，单粒长卵形、类球形或椭圆形，直径 3～14μm，脐点人字状、短缝状或三叉状；复粒由 2～3 分粒组成。薄壁细胞类圆形，具多数椭圆形纹孔，集成纹孔群。内皮层细胞垂周壁波状弯曲，较厚，木化，有稀疏细孔沟。油室大多破碎，完整者类圆形，直径 54～110μm，分泌细胞中有时可见油滴。

（2）取本品粉末 2g，加 70%乙醇 20ml，超声处理 30 分钟，滤过，滤液蒸至无醇味，通过 HP20 型大孔吸附树脂柱（内径为 1cm，柱高为 5cm，30%乙醇湿法装柱），用 30%乙醇 15ml 洗脱，弃去洗脱液，再用 70%乙醇 15ml 洗脱，收集洗脱液，蒸干，残渣加甲醇 1ml 使溶解，作为供试品溶液。另取泽泻对照药材 2g，同法制成对照药材溶液。再取 23-乙酰泽泻醇 B 对照品和 23-乙酰泽泻醇 C 对照品，加甲醇制成每 1ml 含 1mg 的溶液，作为对照品溶液。照薄层色谱法（通则 0502）试验，吸取上述四种溶液各 10μl，分别点于同一硅胶 GF_{254} 薄层板上，以二氯甲烷-甲醇（15：1）为展开剂，展开，取出，晾干，喷以 2%香草醛硫酸溶液-乙醇（1：9）混合溶液，在 105℃加热至斑点显色清晰，分别置日光和紫外光灯（365nm）下检视。供试品色谱中，在与对照药材色谱和对照品色谱相应位置上，分别显相同颜色的斑点或荧光斑点。

【检查】 **水分** 不得过 14.0%（通则 0832 第二法）。

总灰分 不得过 5.0%（通则 2302）。

【浸出物】 照醇溶性浸出物测定法（通则 2201）项下的热浸法测定，用乙醇作溶剂，不得少于 10.0%。

【含量测定】 照高效液相色谱法（通则 0512）测定。

色谱条件与系统适用性试验 以十八烷基硅烷键合硅胶为填充剂；以乙腈为流动相 A，以水为流动相 B，按下表中的规定进行梯度洗脱，23-乙酰泽泻醇 B 检测波长为 208nm，23-乙酰泽泻醇 C 检测波长为 246nm。理论板数按 23-乙酰泽泻醇 B 峰计算应不低于 3000。

时间(分钟)	流动相 A(%)	流动相 B(%)
0～5	45	55
5～30	45→84	55→16
30～40	84	16

对照品溶液的制备　取 23-乙酰泽泻醇 B 对照品和 23-乙酰泽泻醇 C 对照品适量，精密称定，加乙腈制成每 1ml 含 23-乙酰泽泻醇 B 35μg 和 23-乙酰泽泻醇 C 5μg 的混合溶液，即得。

供试品溶液的制备　取本品粉末（过五号筛）约 0.5g，精密称定，置具塞锥形瓶中，精密加入乙腈 25ml，密塞，称定重量，超声处理（功率 250W，频率 50kHz）30 分钟，放冷，再称定重量，用乙腈补足减失的重量，摇匀，滤过，取续滤液，即得。

测定法　分别精密吸取对照品溶液与供试品溶液各 20μl，注入液相色谱仪，测定，即得。

本品按干燥品计算，含 23-乙酰泽泻醇 B($C_{32}H_{50}O_5$)和 23-乙酰泽泻醇 C($C_{32}H_{48}O_6$)的总量不得少于 0.10%。

饮片

【炮制】 **泽泻**　除去杂质，稍浸，润透，切厚片，干燥。

【性状】 本品呈圆形或椭圆形厚片。外表皮淡黄色至淡黄棕色，可见细小突起的须根痕。切面黄白色至淡黄色，粉性，有多数细孔。气微，味微苦。

【检查】 **水分**　同药材，不得过 12.0%。

【鉴别】 **【检查】**（总灰分） **【浸出物】** **【含量测定】** 同药材。

盐泽泻　取泽泻片，照盐水炙法（通则 0213）炒干。

【性状】 本品形如泽泻片，表面淡黄棕色或黄褐色，偶见焦斑。味微咸。

【检查】 **水分**　同药材，不得过 13.0%。

总灰分　同药材，不得过 6.0%。

【浸出物】 同药材，不得少于 9.0%。

【鉴别】（除显微粉末外） **【含量测定】** 同药材。

【性味与归经】 甘、淡，寒。归肾、膀胱经。

【功能与主治】 利水渗湿，泄热，化浊降脂。用于小便不利，水肿胀满，泄泻尿少，痰饮眩晕，热淋涩痛，高脂血症。

【用法与用量】 6～10g。

【贮藏】 置干燥处，防蛀。

降　香

Jiangxiang

DALBERGIAE ODORIFERAE LIGNUM

本品为豆科植物降香檀 *Dalbergia odorifera* T. Chen 树干和根的干燥心材。全年均可采收，除去边材，阴干。

【性状】 本品呈类圆柱形或不规则块状。表面紫红色或红褐色，切面有致密的纹理。质硬，有油性。气微香，味微苦。

【鉴别】 (1)本品粉末棕紫色或黄棕色。具缘纹孔导管巨大，完整者直径约至 300μm，多破碎，具缘纹孔大而清晰，管腔内含红棕色或黄棕色物。纤维成束，棕红色，直径 8～26μm，壁甚厚，有的纤维束周围细胞含草酸钙方晶，形成晶纤维，含晶细胞的壁不均匀木化增厚。草酸钙方晶直径 6～22μm。木射线宽 1～2 列细胞，高至 15 细胞，壁稍厚，纹孔较密。色素块红棕色、黄棕色或淡黄色。

(2)取本品粉末 1g，加甲醇 10ml，超声处理 30 分钟，放置，取上清液作为供试品溶液。另取降香对照药材 1g，同法制成对照药材溶液。照薄层色谱法（通则 0502）试验，吸取上述两种溶液各 2μl，分别点于同一硅胶 G 薄层板上，以甲苯-乙醚-三氯甲烷（7∶2∶1）为展开剂，展开，取出，晾干，喷以 1%香草醛硫酸溶液与无水乙醇（1∶9）的混合溶液，在 105℃加热至斑点显色清晰。供试品色谱中，在与对照药材色谱相应的位置上，显相同颜色的斑点。

(3)取〔鉴别〕(2)项下供试品溶液和对照药材溶液，照薄层色谱法（通则 0502）试验，吸取上述两种溶液各 2μl，分别点于同一硅胶 G 薄层板上，以甲苯-乙酸乙酯（2∶1）为展开剂，展开，取出，晾干，置紫外光灯（365nm）下检视。供试品色谱中，在与对照药材色谱相应的位置上，显相同颜色的荧光斑点。

【浸出物】 照醇溶性浸出物测定法（通则 2201）项下的热浸法测定，用乙醇作溶剂，不得少于 8.0%。

【含量测定】 **挥发油**　照挥发油测定法（通则 2204 甲法）测定。

本品含挥发油不得少于 1.0%(ml/g)。

饮片

【炮制】 除去杂质，劈成小块，碾成细粉或镑片。

【性味与归经】 辛，温。归肝、脾经。

【功能与主治】 化瘀止血，理气止痛。用于吐血，衄血，外伤出血，肝郁胁痛，胸痹刺痛，跌扑伤痛，呕吐腹痛。

【用法与用量】 9～15g，后下。外用适量，研细末敷患处。

【贮藏】 置阴凉干燥处。

细　辛

Xixin

ASARI RADIX ET RHIZOMA

本品为马兜铃科植物北细辛 *Asarum heterotropoides* Fr. Schmidt var. *mandshuricum* (Maxim.) Kitag.、汉城细辛 *Asarum sieboldii* Miq. var. *seoulense* Nakai 或华细辛 *Asarum*

重金属及有害元素　照铅、镉、砷、汞、铜测定法（通则 2321 原子吸收分光光度法或电感耦合等离子体质谱法）测定，铅不得过 10mg/kg、镉不得过 1mg/kg、砷不得过5mg/kg、汞不得过 1mg/kg。

黄曲霉毒素　照真菌毒素测定法（通则 2351）测定。

本品每 1000g 含黄曲霉毒素 B_1 不得过 5μg，黄曲霉毒素 G_2、黄曲霉毒素 G_1、黄曲霉毒素 B_2 和黄曲霉毒素 B_1 的总量不得过 10μg。

【含量测定】　取本品粉末（过三号筛）约 1g，精密称定，精密加入 0.9%氯化钠溶液 5ml，充分搅拌，浸提 30 分钟，并时时振摇，离心，精密量取上清液 100μl，置试管（8mm×38mm）中，加入含 0.5%（牛）纤维蛋白原（以凝固物计）的三羟甲基氨基甲烷盐酸缓冲液[注1]（临用配制）200μl，摇匀，置水浴中（37℃±0.5℃）温浸 5 分钟，滴加每 1ml 中含 40 单位的凝血酶溶液[注2]（每 1 分钟滴加 1 次，每次 5μl，边滴加边轻轻摇匀）至凝固（水蛭）或滴加每 1ml 中含 10 单位的凝血酶溶液[注2]（每 4 分钟滴加 1 次，每次 2μl，边滴加边轻轻摇匀）至凝固（蚂蟥、柳叶蚂蟥），记录消耗凝血酶溶液的体积，按下式计算：

$$U=\frac{C_1V_1}{C_2V_2}$$

式中　U 为每 1g 含凝血酶活性单位，U/g；

C_1 为凝血酶溶液的浓度，μ/ml；

C_2 为供试品溶液的浓度，g/ml；

V_1 为消耗凝血酶溶液的体积，μl；

V_2 为供试品溶液的加入量，μl。

中和一个单位的凝血酶的量，为一个抗凝血酶活性单位。

本品每 1g 含抗凝血酶活性水蛭应不低于 16.0U；蚂蟥、柳叶蚂蟥应不低于 3.0U。

饮片

【炮制】　**水蛭**　洗净，切段，干燥。

【性状】　本品呈不规则的段状、扁块状或扁圆柱状。背部表面黑褐色，稍隆起，腹面棕褐色，均可见细密横环纹。切面灰白色至棕黄色，胶质状。质脆，气微腥。

烫水蛭　取净水蛭段，照炒法（通则 0213）用滑石粉烫至微鼓起。

【性状】　本品呈不规则段状、扁块状或扁圆柱状，略鼓起，背部黑褐色，腹面棕黄色至棕褐色，附有少量白色滑石粉。断面松泡，灰白色至焦黄色。气微腥。

【检查】　**水分**　同药材，不得过 14.0%。

总灰分　同药材，不得过 10.0%。

酸不溶性灰分　同药材，不得过 3.0%。

【鉴别】【检查】（酸碱度　重金属及有害元素　黄曲霉毒素）同药材。

【性味与归经】　咸、苦，平；有小毒。归肝经。

【功能与主治】　破血通经，逐瘀消癥。用于血瘀经闭，癥瘕痞块，中风偏瘫，跌扑损伤。

【用法与用量】　1～3g。

【注意】　孕妇禁用。

【贮藏】　置干燥处，防蛀。

注：[1]三羟甲基氨基甲烷盐酸缓冲液的配制　取 0.2mol/L 三羟甲基氨基甲烷溶液 25ml 与 0.1mol/L 盐酸溶液约 40ml，加水至 100ml，调节 pH 值至 7.4。

[2]凝血酶溶液的配制　取凝血酶试剂适量，加生理盐水配制成每 1ml 含凝血酶 40 个单位或 10 个单位的溶液（临用配制）。

玉　竹

Yuzhu

POLYGONATI ODORATI RHIZOMA

本品为百合科植物玉竹 *Polygonatum odoratum* (Mill.) Druce 的干燥根茎。秋季采挖，除去须根，洗净，晒至柔软后，反复揉搓、晾晒至无硬心，晒干；或蒸透后，揉至半透明，晒干。

【性状】　本品呈长圆柱形，略扁，少有分枝，长 4～18cm，直径 0.3～1.6cm。表面黄白色或淡黄棕色，半透明，具纵皱纹和微隆起的环节，有白色圆点状的须根痕和圆盘状茎痕。质硬而脆或稍软，易折断，断面角质样或显颗粒性。气微，味甘，嚼之发黏。

【鉴别】　本品横切面：表皮细胞扁圆形或扁长方形，外壁稍厚，角质化。薄壁组织中散有多数黏液细胞，直径 80～140μm，内含草酸钙针晶束。维管束外韧型，稀有周木型，散列。

【检查】　**水分**　不得过 16.0%（通则 0832 第二法）。

总灰分　不得过 3.0%（通则 2302）。

【浸出物】　照醇溶性浸出物测定法（通则 2201）项下的冷浸法测定，用 70%乙醇作溶剂，不得少于 50.0%。

【含量测定】　**对照品溶液的制备**　取无水葡萄糖对照品适量，精密称定，加水制成每 1ml 含无水葡萄糖 0.6mg 的溶液，即得。

标准曲线的制备　精密量取对照品溶液 1.0ml、1.5ml、2.0ml、2.5ml、3.0ml，分别置 50ml 量瓶中，加水至刻度，摇匀。精密量取上述各溶液 2ml，置具塞试管中，分别加 4%苯酚溶液 1ml，混匀，迅速加入硫酸 7.0ml，摇匀，于 40℃水浴中保温 30 分钟，取出，置冰水浴中 5 分钟，取出，以相应试剂为空白，照紫外-可见分光光度法（通则 0401），在 490nm 的波长处测定吸光度，以吸光度为纵坐标，浓度为横坐标，绘制标准曲线。

测定法　取本品粗粉约 1g，精密称定，置圆底烧瓶中，加水 100ml，加热回流 1 小时，用脱脂棉滤过，如上重复提取 1 次，两次滤液合并，浓缩至适量，转移至 100ml 量瓶中，加水至刻度，摇匀，精密量取 2ml，加乙醇 10ml，搅拌，离心，取沉

淀加水溶解，置 50ml 量瓶中，并稀释至刻度，摇匀，精密量取 2ml，照标准曲线的制备项下的方法，自“加 4%苯酚溶液 1ml”起，依法测定吸光度，从标准曲线上读出供试品溶液中无水葡萄糖的重量(mg)，计算，即得。

本品按干燥品计算，含玉竹多糖以葡萄糖($C_6H_{12}O_6$)计，不得少于 6.0%。

饮片

【炮制】 除去杂质，洗净，润透，切厚片或段，干燥。

【性状】 本品呈不规则厚片或段。外表皮黄白色至淡黄棕色，半透明，有时可见环节。切面角质样或显颗粒性。气微，味甘，嚼之发黏。

【检查】【浸出物】【含量测定】 同药材。

【性味与归经】 甘，微寒。归肺、胃经。

【功能与主治】 养阴润燥，生津止渴。用于肺胃阴伤，燥热咳嗽，咽干口渴，内热消渴。

【用法与用量】 6～12g。

【贮藏】 置通风干燥处，防霉，防蛀。

功 劳 木

Gonglaomu

MAHONIAE CAULIS

本品为小檗科植物阔叶十大功劳 *Mahonia bealei* (Fort.) Carr. 或细叶十大功劳 *Mahonia fortunei* (Lindl.) Fedde 的干燥茎。全年均可采收，切块片，干燥。

【性状】 本品为不规则的块片，大小不等。外表面灰黄色至棕褐色，有明显的纵沟纹和横向细裂纹，有的外皮较光滑，有光泽，或有叶柄残基。质硬，切面皮部薄，棕褐色，木部黄色，可见数个同心性环纹及排列紧密的放射状纹理，髓部色较深。气微，味苦。

【鉴别】 (1)本品粉末黄色。韧皮纤维淡黄色，直径20～27μm，木化纹孔明显，常 2～3 个成束。石细胞淡黄色，类方形或圆形，直径 20～30μm，壁厚，孔沟明显。网纹导管和具缘纹孔导管，直径 15～27μm。

(2)取本品粉末 0.3g，加甲醇 5ml，超声处理 15 分钟，滤过，滤液补加甲醇至 5ml，作为供试品溶液。另取盐酸小檗碱对照品、盐酸巴马汀对照品、盐酸药根碱对照品，加甲醇制成每 1ml 各含 0.5mg 的混合溶液，作为对照品溶液。照薄层色谱法(通则 0502)试验，吸取上述两种溶液各 1μl，分别点于同一硅胶 G 薄层板上，以甲苯-乙酸乙酯-甲醇-异丙醇-浓氨试液(6∶3∶1.5∶1.5∶0.5)为展开剂，置氨蒸气饱和的展开缸内，展开，取出，晾干，置紫外光灯(365nm)下检视。供试品色谱中，在与对照品色谱相应的位置上，显三个相同的黄色荧光斑点。

【检查】 **水分** 不得过 9.0%(通则 0832 第二法)。

总灰分 不得过 2.0%(通则 2302)。

【浸出物】 照醇溶性浸出物测定法(通则 2201)项下的热浸法测定，用乙醇作溶剂，不得少于 3.0%。

【含量测定】 照高效液相色谱法(通则 0512)测定。

色谱条件与系统适用性试验 以十八烷基硅烷键合硅胶为填充剂；以乙腈为流动相 A，以 0.05mol/L 磷酸二氢钾缓冲液(磷酸调节 pH 值至 3.0)为流动相 B，按下表中的规定进行梯度洗脱；检测波长为 345nm。理论板数按小檗碱峰计算应不低于 5000。

时间(分钟)	流动相 A(%)	流动相 B(%)
0～10	25→28	75→72
10～18	28→50	72→50
18～22	50	50

对照提取物溶液的制备 取功劳木对照提取物(已标示非洲防己碱、药根碱、巴马汀、小檗碱的含量)适量，精密称定，加乙腈-水(25∶75)混合溶液制成每 1ml 含 0.4mg 的溶液，即得。

供试品溶液的制备 取本品粉末(过三号筛)约 0.25g，精密称定，置具塞锥形瓶中，精密加入盐酸-甲醇(1∶100)混合溶液 50ml，密塞，称定重量，超声处理(功率 500W，频率 40kHz)45 分钟，取出，放冷，再称定重量，用盐酸-甲醇(1∶100)混合溶液补足减失的重量，摇匀，滤过，取续滤液，即得。

测定法 分别精密吸取对照提取物溶液与供试品溶液各 10～20μl，注入液相色谱仪，测定。计算非洲防己碱、药根碱、巴马汀和小檗碱的含量。

本品按干燥品计算，含非洲防己碱($C_{20}H_{20}NO_4$)、药根碱($C_{20}H_{20}NO_4$)、巴马汀($C_{21}H_{21}NO_4$)、小檗碱($C_{20}H_{17}NO_4$)的总量，不得少于 1.5%。

【性味与归经】 苦，寒。归肝、胃、大肠经。

【功能与主治】 清热燥湿，泻火解毒。用于湿热泻痢，黄疸尿赤，目赤肿痛，胃火牙痛，疮疖痈肿。

【用法与用量】 9～15g。外用适量。

【贮藏】 置干燥处。

甘 松

Gansong

NARDOSTACHYOS RADIX ET RHIZOMA

本品为败酱科植物甘松 *Nardostachys jatamansi* DC. 的干燥根及根茎。春、秋二季采挖，除去泥沙和杂质，晒干或阴干。

【性状】 本品略呈圆锥形，多弯曲，长 5～18cm。根茎短小，上端有茎、叶残基，呈狭长的膜质片状或纤维状。外层黑棕色，内层棕色或黄色。根单一或数条交结、分枝或并列，直

色谱条件与系统适用性试验 以十八烷基硅烷键合硅胶为填充剂；以乙腈-水-三乙胺-冰醋酸（27∶70.6∶1.6∶0.78）为流动相；检测波长为 270nm。理论板数按荷叶碱峰计算应不低于 2000。

对照品溶液的制备 取荷叶碱对照品适量，精密称定，加甲醇制成每 1ml 含 16μg 的溶液，即得。

供试品溶液的制备 取本品粗粉约 0.5g，精密称定，置具塞锥形瓶中，精密加入甲醇 50ml，称定重量，加热回流 2.5 小时，放冷，再称定重量，用甲醇补足减失的重量，摇匀，滤过，精密量取续滤液 5ml，置 10ml 量瓶中，加水至刻度，摇匀，即得。

测定法 分别精密吸取对照品溶液与供试品溶液各 20μl，注入液相色谱仪，测定，即得。

本品按干燥品计算，含荷叶碱（$C_{19}H_{21}NO_2$）不得少于 0.10%。

饮片

【炮制】 **荷叶** 喷水，稍润，切丝，干燥。

【性状】 本品呈不规则的丝状。上表面深绿色或黄绿色，较粗糙；下表面淡灰棕色，较光滑，叶脉明显突起。质脆，易破碎。稍有清香气，味微苦。

【含量测定】 同药材，含荷叶碱（$C_{19}H_{21}NO_2$）不得少于 0.070%。

【鉴别】【检查】【浸出物】 同药材。

荷叶炭 取净荷叶，照煅炭法（通则 0213）煅成炭。

【性状】 本品呈不规则的片状，表面棕褐色或黑褐色。气焦香，味涩。

【性味与归经】 苦，平。归肝、脾、胃经。

【功能与主治】 清暑化湿，升发清阳，凉血止血。用于暑热烦渴，暑湿泄泻，脾虚泄泻，血热吐衄，便血崩漏。荷叶炭收涩化瘀止血。用于出血症和产后血晕。

【用法与用量】 3～10g；荷叶炭 3～6g。

【贮藏】 置通风干燥处，防蛀。

桂　　枝

Guizhi

CINNAMOMI RAMULUS

本品为樟科植物肉桂 *Cinnamomum cassia* Presl 的干燥嫩枝。春、夏二季采收，除去叶，晒干，或切片晒干。

【性状】 本品呈长圆柱形，多分枝，长 30～75cm，粗端直径 0.3～1cm。表面红棕色至棕色，有纵棱线、细皱纹及小疙瘩状的叶痕、枝痕和芽痕，皮孔点状。质硬而脆，易折断。切片厚 2～4mm，切面皮部红棕色，木部黄白色至浅黄棕色，髓部略呈方形。有特异香气，味甜、微辛，皮部味较浓。

【鉴别】 （1）本品横切面：表皮细胞 1 列，嫩枝有时可见单细胞非腺毛。木栓细胞 3～5 列，最内 1 列细胞外壁增厚。皮层有油细胞及石细胞散在。中柱鞘石细胞群断续排列成环，并伴有纤维束。韧皮部有分泌细胞和纤维散在。形成层明显。木质部射线宽 1～2 列细胞，含棕色物；导管单个散列或 2 至数个相聚；木纤维壁较薄，与木薄壁细胞不易区别。髓部细胞壁略厚，木化。射线细胞偶见细小草酸钙针晶。

粉末红棕色。石细胞类方形或类圆形，直径 30～64μm，壁厚，有的一面菲薄。韧皮纤维大多成束或单个散离，无色或棕色，梭状，有的边缘齿状突出，直径 12～40μm，壁甚厚，木化，孔沟不明显。油细胞类圆形或椭圆形，直径 41～104μm。木纤维众多，常成束，具斜纹孔或相交成十字形。木栓细胞黄棕色，表面观多角形，含红棕色物。导管主为具缘纹孔，直径约至 76μm。

（2）取本品粉末 0.5g，加乙醇 10ml，密塞，浸泡 20 分钟，时时振摇，滤过，取滤液作为供试品溶液。另取桂皮醛对照品，加乙醇制成每 1ml 含 1μl 的溶液，作为对照品溶液。照薄层色谱法（通则 0502）试验，吸取供试品溶液 10～15μl、对照品溶液 2μl，分别点于同一硅胶 G 薄层板上，以石油醚（60～90℃）-乙酸乙酯（17∶3）为展开剂，展开，取出，晾干，喷以二硝基苯肼乙醇试液。供试品色谱中，在与对照品色谱相应的位置上，显相同的橙红色斑点。

（3）取本品粉末 2g，加乙醚 10ml，浸泡 30 分钟，时时振摇，滤过，滤液挥干，残渣加三氯甲烷 1ml 使溶解，作为供试品溶液。另取桂枝对照药材 2g，同法制成对照药材溶液。照薄层色谱法（通则 0502）试验，吸取上述两种溶液各 15μl，分别点于同一硅胶 G 薄层板上，使成条状，以石油醚（60～90℃）-乙酸乙酯（17∶3）为展开剂，展开，取出，晾干，喷以香草醛硫酸试液，在 105℃加热至斑点显色清晰。供试品色谱中，在与对照药材色谱相应的位置上，显相同颜色的斑点。

【检查】 **水分** 不得过 12.0%（通则 0832 第四法）。

总灰分 不得过 3.0%（通则 2302）。

【浸出物】 照醇溶性浸出物测定法（通则 2201）项下的热浸法测定，用乙醇作溶剂，不得少于 6.0%。

【含量测定】 照高效液相色谱法（通则 0512）测定。

色谱条件与系统适用性试验 以十八烷基硅烷键合硅胶为填充剂；以乙腈-水（32∶68）为流动相；检测波长为 290nm。理论板数按桂皮醛峰计算应不低于 3000。

对照品溶液的制备 取桂皮醛对照品适量，精密称定，加甲醇制成每 1ml 含 10μg 的溶液，即得。

供试品溶液的制备 取本品粉末（过四号筛）约 0.5g，精密称定，置具塞锥形瓶中，精密加入甲醇 25ml，称定重量，超声处理（功率 250W，频率 40kHz）30 分钟，放冷，再称定重量，用甲醇补足减失的重量，摇匀，滤过，精密量取续滤液 1ml，置 25ml 量瓶中，加甲醇至刻度，摇匀，即得。

测定法 分别精密吸取对照品溶液与供试品溶液各 10μl，注入液相色谱仪，测定，即得。

本品按干燥品计算，含桂皮醛（C_9H_8O）不得少于1.0%。

饮片

【炮制】 除去杂质，洗净，润透，切厚片，干燥。

【性状】 本品呈类圆形或椭圆形的厚片。表面红棕色至棕色，有时可见点状皮孔或纵棱线。切面皮部红棕色，木部黄白色或浅黄棕色，髓部类圆形或略呈方形，有特异香气，味甜、微辛。

【鉴别】（除横切面外） **【检查】** **【浸出物】** **【含量测定】** 同药材。

【性味与归经】 辛、甘，温。归心、肺、膀胱经。

【功能与主治】 发汗解肌，温通经脉，助阳化气，平冲降气。用于风寒感冒，脘腹冷痛，血寒经闭，关节痹痛，痰饮，水肿，心悸，奔豚。

【用法与用量】 3～10g。

【注意】 孕妇慎用。

【贮藏】 置阴凉干燥处。

桔　梗

Jiegeng

PLATYCODONIS RADIX

本品为桔梗科植物桔梗 *Platycodon grandiflorum* (Jacq.) A. DC. 的干燥根。春、秋二季采挖，洗净，除去须根，趁鲜剥去外皮或不去外皮，干燥。

【性状】 本品呈圆柱形或略呈纺锤形，下部渐细，有的有分枝，略扭曲，长7～20cm，直径0.7～2cm。表面淡黄白色至黄色，不去外皮者表面黄棕色至灰棕色，具纵扭皱沟，并有横长的皮孔样斑痕及支根痕，上部有横纹。有的顶端有较短的根茎或不明显，其上有数个半月形茎痕。质脆，断面不平坦，形成层环棕色，皮部黄白色，有裂隙，木部淡黄色。气微，味微甜后苦。

【鉴别】 （1）本品横切面：木栓细胞有时残存，不去外皮者有木栓层，细胞中含草酸钙小棱晶。栓内层窄。韧皮部乳管群散在，乳管壁略厚，内含微细颗粒状黄棕色物。形成层成环。木质部导管单个散在或数个相聚，呈放射状排列。薄壁细胞含菊糖。

（2）取本品，切片，用稀甘油装片，置显微镜下观察，可见扇形或类圆形的菊糖结晶。

（3）取本品粉末1g，加7%硫酸乙醇-水（1：3）混合溶液20ml，加热回流3小时，放冷，用三氯甲烷振摇提取2次，每次20ml，合并三氯甲烷液，加水洗涤2次，每次30ml，弃去洗液，三氯甲烷液用无水硫酸钠脱水，滤过，滤液回收溶剂至干，残渣加甲醇1ml使溶解，作为供试品溶液。另取桔梗对照药材1g，同法制成对照药材溶液。照薄层色谱法（通则0502）试验，吸取上述两种溶液各10μl，分别点于同一硅胶G薄层板上，以三氯甲烷-乙醚（2：1）为展开剂，展开，取出，晾干，喷以10%硫酸乙醇溶液，在105℃加热至斑点显色清晰。供试品色谱中，在与对照药材色谱相应的位置上，显相同颜色的斑点。

【检查】 **水分** 不得过15.0%（通则0832第二法）。

总灰分 不得过6.0%（通则2302）。

【浸出物】 照醇溶性浸出物测定法（通则2201）项下的热浸法测定，用乙醇作溶剂，不得少于17.0%。

【含量测定】 照高效液相色谱法（通则0512）测定。

色谱条件与系统适用性试验 以十八烷基硅烷键合硅胶为填充剂，YMC-Pack ODS-A色谱柱（柱长为25cm，内径为4.6mm，粒径为5μm）；以乙腈-水（25：75）为流动相；蒸发光散射检测器检测；理论板数按桔梗皂苷D峰计算应不低于3000。

对照品溶液的制备 取桔梗皂苷D对照品适量，精密称定，加甲醇制成每1ml含0.5mg的溶液，即得。

供试品溶液的制备 取本品粉末（过二号筛）约2g，精密称定，置具塞锥形瓶中，精密加入50%甲醇50ml，称定重量，超声处理（功率250W，频率40kHz）30分钟，放冷，再称定重量，用50%甲醇补足减失的重量，摇匀，滤过；精密量取续滤液25ml，蒸干，残渣加水20ml，微热使溶解，用水饱和的正丁醇振摇提取3次，每次20ml，合并正丁醇液，用氨试液50ml洗涤，弃去氨液，再用正丁醇饱和的水50ml洗涤，弃去水液，正丁醇液回收溶剂至干，残渣加甲醇适量使溶解，转移至5ml量瓶中，加甲醇至刻度，摇匀，滤过，取续滤液，即得。

测定法 分别精密吸取对照品溶液10μl、20μl，供试品溶液10～15μl，注入液相色谱仪，测定，以外标两点法对数方程计算，即得。

本品按干燥品计算，含桔梗皂苷D（$C_{57}H_{92}O_{28}$）不得少于0.10%。

饮片

【炮制】 除去杂质，洗净，润透，切厚片，干燥。

【性状】 本品呈椭圆形或不规则厚片。外皮多已除去或偶有残留。切面皮部黄白色，较窄；形成层环纹明显，棕色；木部宽，有较多裂隙。气微，味微甜后苦。

【检查】 **水分** 不得过12.0%（通则0832第二法）。

总灰分 不得过5.0%（通则2302）。

【鉴别】（除横切面外） **【浸出物】** **【含量测定】** 同药材。

【性味与归经】 苦、辛，平。归肺经。

【功能与主治】 宣肺，利咽，祛痰，排脓。用于咳嗽痰多，胸闷不畅，咽痛音哑，肺痈吐脓。

【用法与用量】 3～10g。

【贮藏】 置通风干燥处，防蛀。

中散在含草酸钙针晶束的黏液细胞和含红棕色物的分泌细胞。

粉末红棕色。草酸钙针晶散在或成束存在于黏液细胞中，长50～153μm。导管主为具缘纹孔，直径12～74μm。木纤维多成束，长梭形，直径16～24μm，纹孔口斜裂缝状或人字状。木栓细胞表面观呈类长方形或类多角形，微木化，有的细胞中充满红棕色或棕色物。色素块散在，淡黄色、棕黄色或红棕色。

(2)取本品粉末1g，置试管中，加水10ml，煮沸10分钟，滤过，滤液加氢氧化钠试液1滴，显樱红色，再滴加盐酸酸化后，变为橙黄色。

(3)取本品粉末0.1g，加甲醇1ml，超声处理30分钟，静置或离心，取上清液作为供试品溶液。另取红大戟对照药材0.1g，同法制成对照药材溶液。再取3-羟基巴戟醌对照品、芦西定对照品，加甲醇分别制成每1ml各含0.1mg的溶液，作为对照品溶液。照薄层色谱法(通则0502)试验，吸取上述四种溶液各5μl，分别点于同一硅胶G薄层板上，以三氯甲烷-丙酮-甲酸(8∶1∶0.1)为展开剂，展开，取出，晾干，置紫外光灯(365nm)下检视。供试品色谱中，在与对照药材色谱和对照品色谱相应的位置上，显相同颜色的荧光斑点；在氢氧化钠试液中快速浸渍后，置日光下检视，显相同颜色的斑点。

【检查】 **水分**　不得过11.0%(通则0832第二法)。

总灰分　不得过15.0%(通则2302)。

酸不溶性灰分　不得过4.0%(通则2302)。

【浸出物】 照醇溶性浸出物测定法(通则2201)项下的冷浸法测定，用乙醇作溶剂，不得少于7.0%。

【含量测定】 **3-羟基巴戟醌**　照高效液相色谱法(通则0512)测定。

色谱条件与系统适用性试验　以十八烷基硅烷键合硅胶为填充剂；以甲醇-1%冰醋酸溶液(75∶25)为流动相；检测波长为276nm。理论板数按3-羟基巴戟醌峰计算应不低于3000。

对照品溶液的制备　取3-羟基巴戟醌对照品适量，精密称定，加甲醇制成每1ml含30μg的溶液，即得。

供试品溶液的制备　取本品粉末(过四号筛)约1g，精密称定，置具塞锥形瓶中，精密加入甲醇20ml，称定重量，超声处理(功率300W，频率40kHz)30分钟，放冷，再称定重量，用甲醇补足减失的重量，摇匀，滤过，取续滤液，即得。

测定法　分别精密吸取对照品溶液与供试品溶液各20μl，注入液相色谱仪，测定，即得。

本品按干燥品计算，含3-羟基巴戟醌($C_{15}H_9O_6$)不得少于0.030%。

芦西定　照高效液相色谱法(通则0512)测定。

色谱条件与系统适用性试验　以十八烷基硅烷键合硅胶为填充剂；以甲醇-1%冰醋酸溶液(60∶40)为流动相；检测波长为280nm。理论板数按芦西定峰计算应不低于3000。

对照品溶液的制备　取芦西定对照品适量，精密称定，加甲醇超声处理使溶解制成每1ml含50μg的溶液，即得。

供试品溶液的制备　取本品粉末(过四号筛)约1g，精密称定，置具塞锥形瓶中，精密加入甲醇20ml，称定重量，超声处理(功率300W，频率40kHz)1小时，放冷，再称定重量，用甲醇补足减失的重量，摇匀，滤过，取续滤液，即得。

测定法　分别精密吸取对照品溶液与供试品溶液各20μl，注入液相色谱仪，测定，即得。

本品按干燥品计算，含芦西定($C_{15}H_{10}O_5$)应为0.040%～0.15%。

饮片

【炮制】 除去杂质，洗净，润透，切厚片，干燥。

【性状】 本品呈不规则长圆形或圆形厚片。外表皮红褐色或棕黄色，切面棕黄色。气微，味甘、微辛。

【鉴别】【检查】【浸出物】【含量测定】 同药材。

【性味与归经】 苦，寒；有小毒。归肺、脾、肾经。

【功能与主治】 泻水逐饮，消肿散结。用于水肿胀满，胸腹积水，痰饮积聚，气逆咳喘，二便不利，痈肿疮毒，瘰疬痰核。

【用法与用量】 1.5～3g，入丸散服，每次1g；内服醋制用。外用适量，生用。

【贮藏】 置阴凉干燥处。

红　花

Honghua

CARTHAMI FLOS

本品为菊科植物红花 *Carthamus tinctorius* L. 的干燥花。夏季花由黄变红时采摘，阴干或晒干。

【性状】 本品为不带子房的管状花，长1～2cm。表面红黄色或红色。花冠筒细长，先端5裂，裂片呈狭条形，长5～8mm；雄蕊5，花药聚合成筒状，黄白色；柱头长圆柱形，顶端微分叉。质柔软。气微香，味微苦。

【鉴别】 (1)本品粉末橙黄色。花冠、花丝、柱头碎片多见，有长管状分泌细胞常位于导管旁，直径约至66μm，含黄棕色至红棕色分泌物。花冠裂片顶端表皮细胞外壁突起呈短绒毛状。柱头和花柱上部表皮细胞分化成圆锥形单细胞毛，先端尖或稍钝。花粉粒类圆形、椭圆形或橄榄形，直径约至60μm，具3个萌发孔，外壁有齿状突起。草酸钙方晶存在于薄壁细胞中，直径2～6μm。

(2)取本品粉末0.5g，加80%丙酮溶液5ml，密塞，振摇15分钟，静置，取上清液作为供试品溶液。另取红花对照药材0.5g，同法制成对照药材溶液。照薄层色谱法(通则0502)试验，吸取上述两种溶液各5μl，分别点于同一硅胶H薄层板上，以乙酸乙酯-甲酸-水-甲醇(7∶2∶3∶0.4)为展开剂，展开，取出，晾干。供试品色谱中，在与对照药材色谱相应的位置上，显相同颜色的斑点。

【检查】 **杂质**　不得过2%(通则2301)。

水分　不得过13.0%(通则0832第二法)。

总灰分　不得过15.0%(通则2302)。

酸不溶性灰分　不得过 5.0%（通则 2302）。

吸光度　红色素　取本品，置硅胶干燥器中干燥 24 小时，研成细粉，取约 0.25g，精密称定，置锥形瓶中，加 80%丙酮溶液 50ml，连接冷凝器，置 50℃水浴上温浸 90 分钟，放冷，用 3 号垂熔玻璃漏斗滤过，收集滤液于 100ml 量瓶中，用 80%丙酮溶液 25ml 分次洗涤，洗液并入量瓶中，加 80%丙酮溶液至刻度，摇匀，照紫外-可见分光光度法（通则 0401），在 518nm 的波长处测定吸光度，不得低于 0.20。

【浸出物】　照水溶性浸出物测定法（通则 2201）项下的冷浸法测定，不得少于 30.0%。

【含量测定】　**羟基红花黄色素 A**　照高效液相色谱法（通则 0512）测定。

色谱条件与系统适用性试验　以十八烷基硅烷键合硅胶为填充剂；以甲醇-乙腈-0.7%磷酸溶液（26∶2∶72）为流动相；检测波长为 403nm。理论板数按羟基红花黄色素 A 峰计算应不低于 3000。

对照品溶液的制备　取羟基红花黄色素 A 对照品适量，精密称定，加 25%甲醇制成每 1ml 含 0.13mg 的溶液，即得。

供试品溶液的制备　取本品粉末（过三号筛）约 0.4g，精密称定，置具塞锥形瓶中，精密加入 25%甲醇 50ml，称定重量，超声处理（功率 300W，频率 50kHz）40 分钟，放冷，再称定重量，用 25%甲醇补足减失的重量，摇匀，滤过，取续滤液，即得。

测定法　分别精密吸取对照品溶液与供试品溶液各 10μl，注入液相色谱仪，测定，即得。

本品按干燥品计算，含羟基红花黄色素 A（$C_{27}H_{32}O_{16}$）不得少于 1.0%。

山柰酚　照高效液相色谱法（通则 0512）测定。

色谱条件与系统适用性试验　以十八烷基硅烷键合硅胶为填充剂；以甲醇-0.4%磷酸溶液（52∶48）为流动相；检测波长为 367nm。理论板数按山柰酚峰计算应不低于 3000。

对照品溶液的制备　取山柰酚对照品适量，精密称定，加甲醇制成每 1ml 含 9μg 的溶液，即得。

供试品溶液的制备　取本品粉末（过三号筛）约 0.5g，精密称定，置具塞锥形瓶中，精密加入甲醇 25ml，称定重量，加热回流 30 分钟，放冷，再称定重量，用甲醇补足减失的重量，摇匀，滤过，精密量取续滤液 15ml，置平底烧瓶中，加盐酸溶液（15→37）5ml，摇匀，置水浴中加热水解 30 分钟，立即冷却，转移至 25ml 量瓶中，用甲醇稀释至刻度，摇匀，滤过，取续滤液，即得。

测定法　分别精密吸取对照品溶液与供试品溶液各 10μl，注入液相色谱仪，测定，即得。

本品按干燥品计算，含山柰酚（$C_{15}H_{10}O_6$）不得少于 0.050%。

饮片

【炮制】　除去杂质。

【性状】【鉴别】【检查】【浸出物】【含量测定】　同药材。

【性味与归经】　辛，温。归心、肝经。

【功能与主治】　活血通经，散瘀止痛。用于经闭，痛经，恶露不行，癥瘕痞块，胸痹心痛，瘀滞腹痛，胸胁刺痛，跌扑损伤，疮疡肿痛。

【用法与用量】　3～10g。

【注意】　孕妇慎用。

【贮藏】　置阴凉干燥处，防潮，防蛀。

红花龙胆

Honghualongdan

GENTIANAE RHODANTHAE HERBA

本品为龙胆科植物红花龙胆 *Gentiana rhodantha* Franch. 的干燥全草。秋、冬二季采挖，除去泥沙，晒干。

【性状】　本品长 30～60cm。根茎短，具数条细根；根直径 1～2mm，表面浅棕色或黄白色。茎具棱，直径 1～2mm，黄绿色或带紫色，质脆，断面中空。花单生于枝顶及上部叶腋，花萼筒状，5 裂；花冠喇叭状，长 2～3.5cm，淡紫色或淡黄棕色，先端 5 裂，裂片间褶流苏状。蒴果狭长，2 瓣裂。种子扁卵形，长约 1mm，具狭翅。气微清香，茎叶味微苦，根味极苦。

【鉴别】　（1）本品粉末绿色或黄绿色。下表皮细胞有明显的角质纹理，中央有小且短的乳突，气孔不定式。上表皮细胞稍小，隐现角质纹理。非腺毛 1～9 个细胞，表面具明显的纵向角质纹理，有的细胞含红色色素，基部常膨大或突起呈分支状。木纤维单个或成束散在，细长条形，尖端倾斜或平截，直径 8～18μm，具斜纹孔，直径小者纹孔不明显。花粉粒直径约 35μm，具三个萌发孔。

（2）取本品粉末 0.5g，加甲醇 10ml，超声处理 15 分钟，滤过，滤液作为供试品溶液。另取红花龙胆对照药材 0.5g，同法制成对照药材溶液。再取芒果苷对照品，加甲醇制成每 1ml 含 1mg 的溶液，作为对照品溶液。照薄层色谱法（通则 0502）试验，吸取上述三种溶液各 5μl，分别点于同一硅胶 GF_{254} 薄层板上，以乙酸乙酯-甲醇-水（10∶2∶1）为展开剂，展开，取出，晾干，置紫外光灯（254nm）下检视。供试品色谱中，在与对照药材色谱和对照品色谱相应的位置上，显相同颜色的斑点。

【检查】　**水分**　不得过 9.0%（通则 0832 第二法）。

总灰分　不得过 8.0%（通则 2302）。

酸不溶性灰分　不得过 3.0 %（通则 2302）。

【含量测定】　照高效液相色谱法（通则 0512）测定。

色谱条件与系统适用性试验　以十八烷基硅烷键合硅胶为填充剂；以乙腈-0.02%磷酸溶液（13∶87）为流动相；检测波长为 254nm。理论板数按芒果苷峰计算应不低于 3000。

对照品溶液的制备　取芒果苷对照品适量，精密称定，加甲醇制成每 1ml 含 40μg 的溶液，即得。

供试品溶液的制备　取本品粉末（过三号筛）约 0.3g，精密称定，置具塞锥形瓶中，精密加入 60%甲醇 50ml，密塞，称定重量，超声处理（功率 250W，频率 40kHz）30 分钟，放冷，再称定重量，用 60%甲醇补足减失的重量，摇匀，滤过，精密量取续滤

细胞中，密集，有的含砂晶细胞连接成行。

华钩藤　与钩藤相似。

大叶钩藤　单细胞非腺毛多见，多细胞非腺毛2～15细胞。

毛钩藤　非腺毛1～5细胞。

无柄果钩藤　少见非腺毛，1～7细胞。可见厚壁细胞，类长方形，长41～121μm，直径17～32μm。

（2）取本品粉末2g，加入浓氨试液2ml，浸泡30分钟，加入三氯甲烷50ml，加热回流2小时，放冷，滤过，取滤液10ml，挥干，残渣加甲醇1ml使溶解，作为供试品溶液。另取异钩藤碱对照品，加甲醇制成每1ml含0.5mg的溶液，作为对照品溶液。照薄层色谱法（通则0502）试验，吸取供试品溶液10～20μl、对照品溶液5μl，分别点于同一硅胶G薄层板上，以石油醚（60～90℃）-丙酮（6：4）为展开剂，展开，取出，晾干，喷以改良碘化铋钾试液。供试品色谱中，在与对照品色谱相应的位置上，显相同颜色的斑点。

【检查】 **水分**　不得过10.0%（通则0832第二法）测定。

总灰分　不得过3.0%（通则2302）。

【浸出物】 照醇溶性浸出物测定法（通则2201）项下的热浸法测定，用乙醇作溶剂，不得少于6.0%。

【性味与归经】 甘，凉。归肝、心包经。

【功能与主治】 息风定惊，清热平肝。用于肝风内动，惊痫抽搐，高热惊厥，感冒夹惊，小儿惊啼，妊娠子痫，头痛眩晕。

【用法与用量】 3～12g，后下。

【贮藏】 置干燥处。

香加皮

Xiangjiapi

PERIPLOCAE CORTEX

本品为萝藦科植物杠柳 *Periploca sepium* Bge. 的干燥根皮。春、秋二季采挖，剥取根皮，晒干。

【性状】 本品呈卷筒状或槽状，少数呈不规则的块片状，长3～10cm，直径1～2cm，厚0.2～0.4cm。外表面灰棕色或黄棕色，栓皮松软常呈鳞片状，易剥落。内表面淡黄色或淡黄棕色，较平滑，有细纵纹。体轻，质脆，易折断，断面不整齐，黄白色。有特异香气，味苦。

【鉴别】 （1）本品粉末淡棕色。草酸钙方晶直径9～20μm。石细胞长方形或类多角形，直径24～70μm。乳管含无色油滴状颗粒。木栓细胞棕黄色，多角形。淀粉粒甚多，单粒类圆形或长圆形，直径3～11μm；复粒由2～6分粒组成。

（2）取本品粉末10g，置250ml烧瓶中，加水150ml，加热蒸馏，馏出液具特异香气，收集馏出液10ml，分置二支试管中，一管中加1%三氯化铁溶液1滴，即显红棕色；另一管中加硫酸肼饱和溶液5ml与醋酸钠结晶少量，稍加热，放冷，生成淡黄绿色沉淀，置紫外光灯（365nm）下观察，显强烈的黄色荧光。

（3）取本品粉末1g，加乙醇10ml，加热回流1小时，滤过，滤液置25ml量瓶中，加乙醇至刻度，摇匀，精密量取1ml，置20ml量瓶中，加乙醇至刻度，摇匀，照紫外-可见分光光度法（通则0401）测定，在278nm的波长处有最大吸收。

（4）取本品粉末2g，加甲醇30ml，加热回流1小时，滤过，滤液蒸干，残渣加甲醇2ml使溶解，作为供试品溶液。另取4-甲氧基水杨醛对照品，加甲醇制成每1ml含1mg的溶液，作为对照品溶液。照薄层色谱法（通则0502）试验，吸取上述两种溶液各2μl，分别点于同一硅胶G薄层板上，以石油醚（60～90℃）-乙酸乙酯-冰醋酸（20：3：0.5）为展开剂，展开，取出，晾干，喷以二硝基苯肼试液。供试品色谱中，在与对照品色谱相应的位置上，显相同颜色的斑点。

【检查】 **水分**　不得过13.0%（通则0832第二法）。

总灰分　不得过10.0%（通则2302）。

酸不溶性灰分　不得过4.0%（通则2302）。

【浸出物】 照醇溶性浸出物测定法（通则2201）项下的热浸法测定，用稀乙醇作溶剂，不得少于20.0%。

【含量测定】 照高效液相色谱法（通则0512）测定。

色谱条件与系统适用性试验　以十八烷基硅烷键合硅胶为填充剂；以甲醇-水-醋酸（70：30：2）为流动相；检测波长为278nm。理论板数按4-甲氧基水杨醛峰计算应不低于1000。

内标溶液的制备　取对羟基苯甲酸丁酯适量，精密称定，加60%甲醇制成每1ml含6mg的溶液，即得。

测定法　取4-甲氧基水杨醛对照品适量，精密称定，置棕色量瓶中，加60%甲醇制成每1ml含1mg的溶液。精密量取该溶液4ml、内标溶液2ml，置25ml量瓶中，加60%甲醇至刻度，摇匀，吸取20μl，注入液相色谱仪，记录色谱图；另取本品粗粉约0.25～0.5g，60℃干燥4小时，精密称定，置50ml烧瓶中，加60%甲醇15ml，加热回流1.5小时，滤过，滤液置25ml量瓶中，用少量60%甲醇洗涤容器，洗液滤入同一量瓶中，精密加入内标溶液2ml，加60%甲醇至刻度，摇匀，滤过，取续滤液作为供试品溶液。吸取20μl注入液相色谱仪，按内标法以峰面积计算，即得。

本品于60℃干燥4小时，含4-甲氧基水杨醛（$C_8H_8O_3$）不得少于0.20%。

饮片

【炮制】 除去杂质，洗净，润透，切厚片，干燥。

【性状】 本品呈不规则的厚片。外表面灰棕色或黄棕色，栓皮常呈鳞片状。内表面淡黄色或淡黄棕色，有细纵纹。切面黄白色。有特异香气，味苦。

【鉴别】【检查】【含量测定】 同药材。

【性味与归经】 辛、苦，温；有毒。归肝、肾、心经。

【功能与主治】 利水消肿，祛风湿，强筋骨。用于下肢浮肿，心悸气短，风寒湿痹，腰膝酸软。

【用法与用量】 3～6g。

【注意】 不宜过量服用。

【贮藏】 置阴凉干燥处。

陈　皮

Chenpi

CITRI RETICULATAE PERICARPIUM

本品为芸香科植物橘 *Citrus reticulata* Blanco 及其栽培变种的干燥成熟果皮。药材分为“陈皮”和“广陈皮”。采摘成熟果实，剥取果皮，晒干或低温干燥。

【性状】 **陈皮** 常剥成数瓣，基部相连，有的呈不规则的片状，厚 1～4mm。外表面橙红色或红棕色，有细皱纹和凹下的点状油室；内表面浅黄白色，粗糙，附黄白色或黄棕色筋络状维管束。质稍硬而脆。气香，味辛、苦。

广陈皮 常 3 瓣相连，形状整齐，厚度均匀，约 1mm。外表面橙黄色至棕褐色，点状油室较大，对光照视，透明清晰。质较柔软。

【鉴别】 (1)本品粉末黄白色至黄棕色。中果皮薄壁组织众多，细胞形状不规则，壁不均匀增厚，有的成连珠状。果皮表皮细胞表面观多角形、类方形或长方形，垂周壁稍厚，气孔类圆形，直径 18～26μm，副卫细胞不清晰；侧面观外被角质层，靠外方的径向壁增厚。草酸钙方晶成片存在于中果皮薄壁细胞中，呈多面体形、菱形或双锥形，直径 3～34μm，长 5～53μm，有的一个细胞内含有由两个多面体构成的平行双晶或 3～5 个方晶。橙皮苷结晶大多存在于薄壁细胞中，黄色或无色，呈圆形或无定形团块，有的可见放射状条纹。可见螺纹导管、孔纹导管和网纹导管及较小的管胞。

(2)取本品粉末 0.3g，加甲醇 10ml，超声处理 20 分钟，滤过，取滤液 5ml，浓缩至 1ml，作为供试品溶液。另取橙皮苷对照品，加甲醇制成饱和溶液，作为对照品溶液。照薄层色谱法(通则 0502)试验，吸取上述两种溶液各 2μl，分别点于同一用 0.5%氢氧化钠溶液制备的硅胶 G 薄层板上，以乙酸乙酯-甲醇-水(100∶17∶13)为展开剂，展至约 3cm，取出，晾干，再以甲苯-乙酸乙酯-甲酸-水(20∶10∶1∶1)的上层溶液为展开剂，展至约 8cm，取出，晾干，喷以三氯化铝试液，置紫外光灯(365nm)下检视。供试品色谱中，在与对照品色谱相应的位置上，显相同颜色的荧光斑点。

(3)另取 2-甲氨基苯甲酸甲酯对照品，加甲醇制成每 1ml 含 0.1mg 的溶液，作为对照品溶液，再取广陈皮对照提取物，加甲醇超声处理 20 分钟，制成每 1ml 含 15mg 的溶液，作为对照提取物溶液。照薄层色谱法(通则 0502)试验，吸取上述两种溶液及〔鉴别〕(2)项下的供试品溶液各 2μl，分别点于同一硅胶 G 薄层板上，以甲苯-乙酸乙酯-甲醇-水(10∶4∶2∶0.5)10℃以下放置的上层溶液为展开剂，展至约 5cm，取出，晾干，再以环己烷为展开剂，展至约 8cm，取出，晾干，置紫外光灯(365nm)下检视。供试品色谱中，在与对照提取物色谱和对照品色谱相应的位置上，显相同颜色的荧光斑点(广陈皮)。

【检查】 **水分** 不得过 13.0%(通则 0832 第四法)。

黄曲霉毒素 照真菌毒素测定法(通则 2351)测定。

取本品粉末(过二号筛)约 5g，精密称定，加入氯化钠 3g，照黄曲霉毒素测定法项下供试品的制备方法测定，计算，即得。

本品每 1000g 含黄曲霉毒素 B_1 不得过 5μg，黄曲霉毒素 G_2、黄曲霉毒素 G_1、黄曲霉毒素 B_2 和黄曲霉毒素 B_1 的总量不得过 10μg。

【含量测定】 **陈皮** 照高效液相色谱法(通则 0512)测定。

色谱条件与系统适用性试验　以十八烷基硅烷键合硅胶为填充剂；以乙腈-水(22∶78)为流动相；检测波长为 283nm。理论板数按橙皮苷峰计算应不低于 2000。

对照品溶液的制备　取橙皮苷对照品适量，精密称定，加甲醇制成每 1ml 含 0.4mg 的溶液，即得。

供试品溶液的制备　取本品粗粉(过二号筛)约 0.2g，精密称定，置具塞锥形瓶中，精密加入甲醇 25ml，密塞，称定重量，超声处理(功率 300W；频率 40kHz)45 分钟，放冷，再称定重量，用甲醇补足减失的重量，摇匀，滤过，取续滤液，即得。

测定法　分别精密吸取对照品溶液与供试品溶液各 5μl，注入液相色谱仪，测定，即得。

本品按干燥品计算，含橙皮苷($C_{28}H_{34}O_{15}$)不得少于 3.5%。

广陈皮 照高效液相色谱法(通则 0512)测定。

色谱条件与系统适用性试验　以十八烷基硅烷键合硅胶为填充剂；以乙腈为流动相 A，以水为流动相 B，按下表中的规定进行梯度洗脱；橙皮苷检测波长为 283nm，川陈皮素和橘皮素检测波长为 330nm。理论板数按橙皮苷峰和川陈皮素峰计算均应不低于 2000。

时间(分钟)	流动相 A(%)	流动相 B(%)	检测波长(nm)
0～10	22	78	283
10～20	22→48	78→52	283
20～35	48	52	330

对照品溶液的制备　取橙皮苷对照品、川陈皮素对照品、橘皮素对照品适量，精密称定，加甲醇制成每 1ml 含橙皮苷 0.2mg、川陈皮素 25μg、橘皮素 15μg 的混合溶液，即得。

供试品溶液的制备　取本品粗粉(过二号筛)约 0.2g，精密称定，置具塞锥形瓶中，精密加入甲醇 25ml，密塞，称定重量，超声处理(功率 300W，频率 40kHz)45 分钟，放冷，再称定重量，用甲醇补足减失的重量，摇匀，滤过，取续滤液，即得。

测定法　分别精密吸取对照品溶液与供试品溶液各 5μl，注入液相色谱仪，测定，即得。

本品按干燥品计算，含橙皮苷($C_{28}H_{34}O_{15}$)不得少于 2.0%；含川陈皮素($C_{21}H_{22}O_8$)和橘皮素($C_{20}H_{20}O_7$)的总量，不得少于 0.42%。

饮片

【炮制】 除去杂质，喷淋水，润透，切丝，干燥。

【性状】 本品呈不规则的条状或丝状。外表面橙红色或红棕色，有细皱纹和凹下的点状油室。内表面浅黄白色，粗糙，附黄白色或黄棕色筋络状维管束。气香，味辛、苦。

【含量测定】 陈皮 同药材，含橙皮苷（$C_{28}H_{34}O_{15}$）不得少于 2.5%。

广陈皮 同药材，含橙皮苷（$C_{28}H_{34}O_{15}$）不得少于 1.75%；含川陈皮素（$C_{21}H_{22}O_8$）和橘皮素（$C_{20}H_{20}O_7$）的总量，不得少于 0.40%。

【鉴别】【检查】 同药材。

【性味与归经】 苦、辛，温。归肺、脾经。

【功能与主治】 理气健脾，燥湿化痰。用于脘腹胀满，食少吐泻，咳嗽痰多。

【用法与用量】 3～10g。

【贮藏】 置阴凉干燥处，防霉，防蛀。

注：栽培变种主要有茶枝柑 *Citrus reticulata* 'Chachi'（广陈皮）、大红袍 *Citrus reticulata* 'Dahongpao'、温州蜜柑 *Citrus reticulata* 'Unshiu'、福橘 *Citrus reticulata* 'Tangerina'。

附　子

Fuzi

ACONITI LATERALIS RADIX PRAEPARATA

本品为毛茛科植物乌头 *Aconitum carmichaelii* Debx. 的子根的加工品。6 月下旬至 8 月上旬采挖，除去母根、须根及泥沙，习称"泥附子"，加工成下列规格。

（1）选择个大、均匀的泥附子，洗净，浸入胆巴的水溶液中过夜，再加食盐，继续浸泡，每日取出晒晾，并逐渐延长晒晾时间，直至附子表面出现大量结晶盐粒（盐霜）、体质变硬为止，习称"盐附子"。

（2）取泥附子，按大小分别洗净，浸入胆巴的水溶液中数日，连同浸液煮至透心，捞出，水漂，纵切成厚约 0.5cm 的片，再用水浸漂，用调色液使附片染成浓茶色，取出，蒸至出现油面、光泽后，烘至半干，再晒干或继续烘干，习称"黑顺片"。

（3）选择大小均匀的泥附子，洗净，浸入胆巴的水溶液中数日，连同浸液煮至透心，捞出，剥去外皮，纵切成厚约 0.3cm 的片，用水浸漂，取出，蒸透，晒干，习称"白附片"。

【性状】 盐附子 呈圆锥形，长 4～7cm，直径 3～5cm。表面灰黑色，被盐霜，顶端有凹陷的芽痕，周围有瘤状突起的支根或支根痕。体重，横切面灰褐色，可见充满盐霜的小空隙和多角形形成层环纹，环纹内侧导管束排列不整齐。气微，味咸而麻，刺舌。

黑顺片 为纵切片，上宽下窄，长 1.7～5cm，宽 0.9～3cm，厚 0.2～0.5cm。外皮黑褐色，切面暗黄色，油润具光泽，半透明状，并有纵向导管束。质硬而脆，断面角质样。气微，味淡。

白附片 无外皮，黄白色，半透明，厚约 0.3cm。

【鉴别】 取本品粉末 2g，加氨试液 3ml 润湿，加乙醚 25ml，超声处理 30 分钟，滤过，滤液挥干，残渣加二氯甲烷 0.5ml 使溶解，作为供试品溶液。另取苯甲酰新乌头原碱对照品、苯甲酰乌头原碱对照品、苯甲酰次乌头原碱对照品，加异丙醇-二氯甲烷（1∶1）混合溶液制成每 1ml 各含 1mg 的混合溶液，作为对照品溶液（单酯型生物碱）。再取新乌头碱对照品、次乌头碱对照品、乌头碱对照品，加异丙醇-二氯甲烷（1∶1）混合溶液制成每 1ml 各含 1mg 的混合溶液，作为对照品溶液（双酯型生物碱）。照薄层色谱法（通则 0502）试验，吸取供试品溶液和对照品溶液各 5～10μl，分别点于同一硅胶 G 薄层板上，以正己烷-乙酸乙酯-甲醇（6.4∶3.6∶1）为展开剂，置氨蒸气饱和 20 分钟的展开缸内，展开，取出，晾干，喷以稀碘化铋钾试液。供试品色谱中，盐附子在与新乌头碱对照品、次乌头碱对照品和乌头碱对照品色谱相应的位置上，显相同颜色的斑点；黑顺片或白附片在与苯甲酰新乌头原碱对照品、苯甲酰乌头原碱对照品、苯甲酰次乌头原碱对照品色谱相应的位置上，显相同颜色的斑点。

【检查】 水分 不得过 15.0%（通则 0832 第二法）。

双酯型生物碱 照〔含量测定〕项下色谱条件、供试品溶液的制备方法试验。

对照品溶液的制备　取新乌头碱对照品、次乌头碱对照品、乌头碱对照品适量，精密称定，加异丙醇-二氯甲烷（1∶1）混合溶液制成每 1ml 各含 5μg 的混合溶液，即得。

测定法　分别精密吸取上述对照品溶液与〔含量测定〕项下供试品溶液各 10μl，注入液相色谱仪，测定，即得。

本品含双酯型生物碱以新乌头碱（$C_{33}H_{45}NO_{11}$）、次乌头碱（$C_{33}H_{45}NO_{10}$）和乌头碱（$C_{34}H_{47}NO_{11}$）的总量计，不得过 0.020%。

【含量测定】 照高效液相色谱法（通则 0512）测定。

色谱条件与系统适用性试验 以十八烷基硅烷键合硅胶为填充剂；以乙腈-四氢呋喃（25∶15）为流动相 A，以 0.1mol/L 醋酸铵溶液（每 1000ml 加冰醋酸 0.5ml）为流动相 B，按下表中的规定进行梯度洗脱，检测波长为 235nm。理论板数按苯甲酰新乌头原碱峰计算应不低于 3000。

时间（分钟）	流动相 A（%）	流动相 B（%）
0～48	15→26	85→74
48～49	26→35	74→65
49～58	35	65
58～65	35→15	65→85

对照品溶液的制备 取苯甲酰新乌头原碱对照品、苯甲酰乌头原碱对照品、苯甲酰次乌头原碱对照品适量，精密称定，加异丙醇-二氯甲烷（1∶1）混合溶液制成每 1ml 各含

试品色谱中，在与对照药材色谱和对照品色谱相应的位置上，显相同颜色的斑点。

(2)取淫羊藿苷对照品，加甲醇制成每 1ml 含 1mg 的溶液，作为对照品溶液。照薄层色谱法（通则 0502）试验，吸取〔鉴别〕(1)项下供试品溶液和上述对照品溶液各 5μl，分别点于同一硅胶 G 薄层板上，以三氯甲烷-甲醇-水（13：7：2）10℃以下放置的下层溶液为展开剂，展开，取出，晾干，喷以三氯化铝试液，置紫外光灯（365nm）下检视。供试品色谱中，在与对照品色谱相应的位置上，显相同颜色的荧光斑点。

(3)取本品 4g，研细，加甲醇 40ml，超声处理 30 分钟，滤过，滤液回收溶剂至干，残渣加水 20ml 使溶解，加稀盐酸调节 pH 值至 1，用乙酸乙酯 50ml 振摇提取，提取液回收溶剂至干，残渣加甲醇 1ml 使溶解，作为供试品溶液。另取丹参素钠对照品，加甲醇制成每 1ml 含 1mg 的溶液，作为对照品溶液。照薄层色谱法（通则 0502）试验，吸取上述两种溶液各 5μl，分别点于同一硅胶 G 薄层板上，以三氯甲烷-丙酮-甲酸（25：10：4）为展开剂，展开，取出，晾干，置氨蒸气中熏 15 分钟后，放置 10 分钟，置紫外光灯（365nm）下检视。供试品色谱中，在与对照品色谱相应的位置上，显相同颜色的荧光斑点。

(4)取枳壳对照药材 0.5g，加甲醇 30ml，超声处理 30 分钟，滤过，滤液回收溶剂至干，残渣加甲醇 1ml 溶解，作为对照药材溶液。照薄层色谱法（通则 0502）试验，吸取〔鉴别〕(3)项下供试品溶液 5μl 和上述对照药材溶液 3μl，分别点于同一硅胶 G 薄层板上，以乙酸乙酯-丙酮-甲酸-水（4：2：0.15：5）的上层溶液为展开剂，展开，取出，晾干，喷以三氯化铝试液，置紫外光灯（365nm）下检视。供试品色谱中，在与对照药材色谱相应的位置上，显相同颜色的荧光斑点。

(5)取本品 4g，研细，加甲醇 40ml，超声处理 30 分钟，滤过，滤液回收溶剂至干，残渣加水 20ml 使溶解，用乙醚振摇提取三次（25ml，20ml，15ml），合并提取液，回收溶剂至干，残渣加乙酸乙酯 2ml 使溶解，作为供试品溶液。另取肉桂酸对照品，加乙酸乙酯制成每 1ml 含 1mg 的溶液，作为对照品溶液。照薄层色谱法（通则 0502）试验，取上述两种溶液各 5μl，分别点于同一硅胶 GF_{254} 薄层板上，以石油醚（30～60℃）-乙酸乙酯-甲酸（12：5：1）的上层溶液为展开剂，展开，取出，晾干，置紫外光灯（254nm）下检视。供试品色谱中，在与对照品色谱相应的位置上，显相同颜色的斑点。

(6)取本品 1g，研细，加 50%甲醇 50ml，超声处理 30 分钟，放冷，摇匀，滤过，取续滤液作为供试品溶液。另取绿原酸对照品，加 50%甲醇制成每 1ml 含 50μg 的溶液，作为对照品溶液。照高效液相色谱法（通则 0512）试验，以十八烷基硅烷键合硅胶为填充剂；以乙腈-0.4%磷酸溶液（10：90）为流动相；检测波长为 327nm。理论板数按绿原酸峰计算应不得低于 1500。分别吸取上述两种溶液各 10μl，注入液相色谱仪。供试品色谱中，应呈现与对照品色谱峰保留时间相对应的色谱峰。

【检查】 应符合颗粒剂项下有关的各项规定（通则 0104）。

【含量测定】 照高效液相色谱法（通则 0512）测定。

色谱条件与系统适用性试验 以十八烷基硅烷键合硅胶为填充剂；以乙腈-水（35：65）为流动相；蒸发光散射检测器检测。理论板数按黄芪甲苷峰计算应不低于 4000。

对照品溶液的制备 取黄芪甲苷对照品适量，精密称定，加甲醇制成每 1ml 含 0.25mg 的溶液，即得。

供试品溶液的制备 取装量差异项下的本品适量，混匀，研细，取约 5g，精密称定，置具塞锥形瓶中，精密加水 50ml，密塞，称定重量，超声处理（功率 300W，频率 33kHz）30 分钟，放冷，再称定重量，用水补足减失的重量，摇匀，离心，精密量取上清液 25ml，用水饱和的正丁醇振摇提取 6 次（30ml，30ml，30ml，20ml，20ml，20ml），合并正丁醇提取液，用氨试液充分洗涤 2 次，每次 20ml，弃去氨洗液，用正丁醇饱和的水洗涤 2 次，每次 20ml，弃去水液，正丁醇液回收溶剂至干，残渣加甲醇适量使溶解，转移至 10ml 量瓶中，加甲醇稀释至刻度，摇匀，即得。

测定法 分别精密吸取对照品溶液 5μl、15μl，供试品溶液 15μl，注入液相色谱仪，测定，用外标两点法对数方程计算，即得。

本品每袋含黄芪以黄芪甲苷（$C_{41}H_{68}O_{14}$）计，不得少于 1.2mg。

【功能与主治】 益气养心，安神止悸。用于气阴两虚所致的心悸、胸闷、胸痛、气短乏力、失眠多梦、自汗、盗汗、心烦；病毒性心肌炎、冠心病心绞痛见上述症候者。

【用法与用量】 口服。一次 1 袋，一日 3 次，饭后服用或遵医嘱。28 天为一疗程。

【注意】 孕妇忌服。偶见服药后胃部不适，宜饭后服用。

【规格】 每袋装 5g

【贮藏】 密封，置阴凉处。

芪苈强心胶囊

Qili Qiangxin Jiaonang

【处方】

黄芪 450g	人参 225g
黑顺片 112.5g	丹参 225g
葶苈子 150g	泽泻 225g
玉竹 75g	桂枝 90g
红花 90g	香加皮 180g
陈皮 75g	

【制法】 以上十一味，黄芪、葶苈子、泽泻、人参、香加皮加 70%乙醇加热回流提取二次，第一次 3 小时，第二次 2 小时，提取液滤过，滤液减压回收乙醇，浓缩至相对密度为 1.25～1.30(60℃)的稠膏，备用；桂枝、陈皮水蒸气蒸馏提取

挥发油，收集挥发油，备用；提油后的水溶液滤过，备用；药渣再加水煎煮1小时，滤过，与备用滤液合并，备用；黑顺片、丹参、玉竹、红花加水煎煮二次，每次2小时，合并煎液，滤过，滤液与桂枝和陈皮的水煎液合并，浓缩至相对密度为1.25～1.30(60℃)，加乙醇，使含醇量达70%，在4℃以下静置24小时，滤过，滤液减压回收乙醇，浓缩至相对密度为1.25～1.30(60℃)，与上述备用稠膏合并，在65～70℃干燥。干膏粉碎成细粉，加入适量糊精，制颗粒，喷入挥发油，混匀，装入胶囊，制成1000粒，即得。

【性状】 本品为硬胶囊，内容物为棕褐色至黑褐色的颗粒；味苦。

【鉴别】 (1)取〔含量测定〕项下的供试品溶液作为供试品溶液。另取人参皂苷 Rb_1 对照品、人参皂苷 Rb_2 对照品和人参皂苷Rf对照品，分别加甲醇制成每1ml含0.2mg的溶液，作为对照品溶液。照〔含量测定〕项下的方法试验，吸取对照品溶液与供试品溶液各5～15μl，注入液相色谱仪，记录色谱图。供试品色谱中应呈现与对照品色谱峰保留时间相对应的色谱峰。

(2)取本品内容物2g，加甲醇25ml，超声处理30分钟，滤过，滤液蒸干。残渣用水25ml溶解，滤过，滤液用盐酸调节pH值至1～2，用乙酸乙酯振摇提取2次，每次15ml，合并乙酸乙酯提取液，蒸干，残渣加无水乙醇1ml使溶解，作为供试品溶液。另取丹参对照药材0.5g，加水30ml，加热回流30分钟，放冷，滤过，取滤液，自“用盐酸调节pH值至1～2”起，同法制成对照药材溶液。照薄层色谱法(通则0502)试验，吸取上述两种溶液各3～7μl，分别点于同一硅胶G薄层板上，以甲苯-二氯甲烷-乙酸乙酯-甲酸(5：5：5：0.8)为展开剂，展开，取出，晾干，喷以2%三氯化铁乙醇溶液，加热至斑点显色清晰，置日光下检视。供试品色谱中，在与对照药材色谱相应的位置上，显相同颜色的主斑点。

(3)取〔含量测定〕项下供试品溶液制备项的备用甲醇溶液，浓缩至约2ml，作为供试品溶液。另取香加皮对照药材0.5g，加甲醇10ml，超声处理30分钟，滤过，滤液浓缩至约2ml，作为对照药材溶液。照薄层色谱法(通则0502)试验，吸取供试品溶液5～10μl，对照药材溶液2～4μl，分别点于同一硅胶G薄层板上，以石油醚(60～90℃)-乙酸乙酯-冰醋酸(20：3：0.5)为展开剂，展开，取出，晾干，置紫外光灯(254nm)下检视。供试品色谱中，在与对照药材色谱相应的位置上，显相同颜色的荧光斑点。

(4)取本品内容物2g，置具塞锥形瓶中，加乙醇20ml，密塞，浸泡20分钟，振摇10分钟，滤过，滤液作为供试品溶液。另取桂皮醛对照品，加乙醇制成每1ml含1μl的溶液，作为对照品溶液。照薄层色谱法(通则0502)试验，吸取供试品溶液10～15μl、对照品溶液2μl，分别点于同一硅胶G薄层板上，以石油醚(60～90℃)-乙酸乙酯(17：3)为展开剂，展开，取出，晾干，喷以二硝基苯肼乙醇试液，放置约5分钟，置日光下检视。供试品色谱中，在与对照品色谱相应的位置上，显相同颜色的斑点。

(5)取〔鉴别〕(3)项下的供试品溶液，蒸干，残渣加水10ml使溶解，用三氯甲烷振摇提取2次，每次15ml，再用乙酸乙酯15ml振摇提取，乙酸乙酯液蒸干，残渣加甲醇1ml使溶解，作为供试品溶液。另取橙皮苷对照品，加甲醇制成饱和溶液，作为对照品溶液。照薄层色谱法(通则0502)试验，吸取供试品溶液5～10μl、对照品溶液2μl，分别点于同一硅胶G薄层板上，以三氯甲烷-乙酸乙酯-甲醇-水(15：40：22：10)10℃以下放置的下层溶液为展开剂，置4℃以下展开，取出，晾干，喷以三氯化铝试液，置紫外光灯(365nm)下检视。供试品色谱中，在与对照品色谱相应的位置上，显相同颜色的荧光斑点。

【检查】 **乌头碱限量** 取本品内容物18g，置具塞锥形瓶中，加氨试液10ml，振摇30分钟，加乙醚100ml，密塞，振摇15分钟，放置24小时，分取乙醚液，滤过，用乙醚10ml洗涤滤渣及滤纸，合并乙醚，低温蒸干，残渣用无水乙醇溶解并转移至2ml量瓶中，加乙醇至刻度，摇匀，作为供试品溶液。另取乌头碱对照品，加无水乙醇制成每1ml含1.0mg的溶液，作为对照品溶液。照薄层色谱法(通则0502)试验，吸取供试品溶液12μl、对照品溶液5μl，分别点于同一硅胶G薄层板上，以正己烷-乙酸乙酯-乙醇(6.4：3.6：1)为展开剂，置氨蒸气饱和的展开缸内，展开，取出，晾干，喷以稀碘化铋钾试液，置日光下检视。供试品色谱中，在与对照品色谱相应的位置上出现的斑点应小于对照品的斑点，或不出现斑点。

其他 应符合胶囊剂项下有关的各项规定(通则0103)。

【含量测定】 照高效液相色谱法(通则0512)测定。

色谱条件与系统适用性试验 以十八烷基硅烷键合硅胶为填充剂；以乙腈-水(30：70)为流动相；柱温为30℃；用蒸发光散射检测器检测。理论板数按黄芪甲苷峰计算应不低于4000。

对照品溶液的制备 取黄芪甲苷对照品适量，精密称定，加70%甲醇制成每1ml含80μg的溶液，即得。

供试品溶液的制备 取装量差异项下的本品内容物，混匀，研细，取约2g，精密称定，置具塞锥形瓶中，精密加甲醇50ml，密塞，称定重量，超声处理(功率250W，频率40kHz)30分钟，放冷，再称定重量，用甲醇补足减失的重量，摇匀，滤过，精密量取续滤液20ml(剩余甲醇溶液备用)，蒸干，残渣用3%氢氧化钠溶液20ml溶解，用水饱和的正丁醇振摇提取3次，每次20ml，合并正丁醇提取液，用正丁醇饱和的水洗涤2次，每次25ml，合并水洗液，用水饱和正丁醇20ml振摇提取，合并正丁醇液，蒸干，残渣用70%甲醇溶解并转移至5ml量瓶中，加70%甲醇至刻度，摇匀，滤过，取续滤液，即得。

测定法 精密吸取对照品溶液5μl与15μl、供试品溶液5～15μl，注入高效液相色谱仪，测定，用外标两点法对数方程计算，即得。

本品每粒含黄芪以黄芪甲苷($C_{41}H_{68}O_{14}$)计，不得少

于 0.12mg。

【功能与主治】 益气温阳，活血通络，利水消肿。用于冠心病、高血压病所致轻、中度充血性心力衰竭证属阳气虚乏，络瘀水停证，症见心慌气短，动则加剧，夜间不能平卧，下肢浮肿，倦怠乏力，小便短少，口唇青紫，畏寒肢冷，咳吐稀白痰。

【用法与用量】 口服。一次 4 粒，一日 3 次。

【规格】 每粒装 0.3g

【贮藏】 密封。

芪明颗粒

Qiming Keli

【处方】 黄芪 592g　　葛根 592g

地黄 556g　　枸杞子 556g

决明子 370g　　茺蔚子 222g

蒲黄 370g　　水蛭 74g

【制法】 以上八味，决明子破碎后，与黄芪等七味加65%乙醇回流提取 2 小时，滤过，滤液回收乙醇，并浓缩至相对密度为 1.10～1.12(60℃)的清膏，备用。药渣用水煎煮二次，每次 2 小时，合并煎液，滤过，取上清液，减压浓缩至相对密度为 1.10～1.12(60℃)的清膏，与醇提清膏混合，用聚维酮浆制粒，干燥，制成 1000g，即得。

【性状】 本品为棕黄色至棕褐色的颗粒；气微，味甘、微苦。

【鉴别】 (1)取本品 0.5g，加水 35ml，加热煮沸 15 分钟，放冷，滤过，滤液用乙酸乙酯 15ml 振摇提取，提取液回收溶剂至约 1ml，作为供试品溶液。另取枸杞子对照药材 0.5g，同法制成对照药材溶液。再取东莨菪内酯对照品，加乙酸乙酯制成每 1ml 含 0.5mg 的溶液，作为对照品溶液。照薄层色谱法(通则 0502)试验，吸取上述三种溶液各 10μl，分别点于同一硅胶 G 薄层板上，以甲苯-乙酸乙酯-甲酸(15：8：1.5)为展开剂，展开，取出，晾干，置紫外光灯(365nm)下检视。供试品色谱中，在与对照药材色谱和对照品色谱相应位置上，显相同颜色的荧光主斑点。

(2)取本品 2g，加甲醇 20ml，超声处理 30 分钟，滤过，滤液回收溶剂至干，残渣加水 10ml 及盐酸 1ml，置水浴上加热回流 30 分钟，立即冷却，用乙醚振摇提取 2 次，每次 20ml，合并乙醚液，挥干，残渣加甲醇 1ml 使溶解，作为供试品溶液。另取决明子对照药材 1g，同法制成对照药材溶液。再取大黄素对照品，加甲醇制成每 1ml 含 1mg 的溶液，作为对照品溶液。照薄层色谱法(通则 0502)试验，吸取上述三种溶液各 2μl，分别点于同一硅胶 H 薄层板上，以石油醚(30～60℃)-甲酸乙酯-甲酸(15：5：1)的上层溶液为展开剂，展开，取出，晾干，置紫外光灯(365nm)下检视。供试品色谱中，在与对照药材色谱和对照品色谱相应位置上，显相同颜色的荧光斑点；置氨蒸气中熏后，置日光下检视，斑点变为红色。

(3)取本品，照〔含量测定〕项下的方法试验。供试品色谱中应呈现与黄芪甲苷对照品色谱峰保留时间相对应的色谱峰。

【检查】 应符合颗粒剂项下有关的各项规定(通则 0104)。

【含量测定】 **黄芪** 照高效液相色谱法(通则 0512)测定。

色谱条件与系统适用性试验　以十八烷基硅烷键合硅胶为填充剂；以甲醇-水(75：25)为流动相；用蒸发光散射检测器检测。理论板数按黄芪甲苷峰计算应不低于 5000。

对照品溶液的制备　取黄芪甲苷对照品适量，精密称定，加甲醇制成每 1ml 含 0.5mg 的溶液，即得。

供试品溶液的制备　取装量差异项下的本品内容物，研细，取约 5g，精密称定，置具塞锥形瓶中，精密加甲醇 50ml，密塞，称定重量，超声处理(功率 300W，频率 50kHz)45 分钟，放冷，再称定重量，用甲醇补足减失的重量，摇匀，滤过，精密量取续滤液 25ml，回收溶剂至干，残渣加水 25ml，微热使溶解，用水饱和的正丁醇振摇提取 4 次，每次 25ml，合并正丁醇提取液，用氨试液充分洗涤 3 次，每次 30ml，弃去氨洗液，取正丁醇液回收溶剂至干，残渣用甲醇溶解并转移至 5ml 量瓶中，用甲醇稀释至刻度，摇匀，滤过，取续滤液，即得。

测定法　精密吸取对照品溶液 5μl、10μl 与供试品溶液 5～10μl，注入液相色谱仪，测定，以外标两点法对数方程计算，即得。

本品每袋含黄芪以黄芪甲苷($C_{41}H_{68}O_{14}$)计，不得少于 1.1mg。

葛根 照高效液相色谱法(通则 0512)测定。

色谱条件与系统适用性试验　以十八烷基硅烷键合硅胶为填充剂；以甲醇-水(25：75)为流动相；检测波长为 250nm。理论板数按葛根素峰计算应不低于 4000。

对照品溶液的制备　取葛根素对照品适量，精密称定，加30%乙醇制成每 1ml 含 80μg 的溶液，即得。

供试品溶液的制备　取装量差异项下的本品内容物，研细，取约 0.2g，精密称定，置具塞锥形瓶中，精密加入 30%乙醇 50ml，密塞，称定重量，超声处理(功率 300W，频率 50kHz)30 分钟，放冷，再称定重量，用 30%乙醇补足减失的重量，摇匀，滤过，取续滤液，即得。

测定法　分别精密吸取对照品溶液与供试品溶液各 20μl，注入液相色谱仪，测定，即得。

本品每袋含葛根以葛根素($C_{21}H_{20}O_9$)计，不得少于 32.0mg。

【功能与主治】 益气生津、滋养肝肾、通络明目。用于 2 型糖尿病视网膜病变单纯型，中医辨证属气阴亏虚、肝肾不足、目络瘀滞证，症见视物昏花、目睛干涩、神疲乏力、五心烦热、自汗盗汗、口渴喜饮、便秘、腰膝酸软、头晕、耳鸣。
