# Supplementary material for: The traditional Chinese medicine Qiliqiangxin in heart failure with reduced ejection fraction: a randomized, double-blind, placebo-controlled trial
Source: Nat Med. 2024 Aug 2;30(8):2295–302. doi: 10.1038/s41591-024-03169-2 (PMC11333273; doi:10.1038/s41591-024-03169-2)
Supplement: Supplementary file 2 — Reporting Summary [file 41591_2024_3169_MOESM2_ESM.pdf]

nature portfolio

Corresponding author(s): Haifeng Zhang, Anthony Rosenzweig, Zhenhua Jia, Xinli Li; NMED-A129749

Last updated by author(s): Jun 17, 2024

Reporting Summary

Nature Portfolio wishes to improve the reproducibility of the work that we publish. This form provides structure for consistency and transparency in reporting. For further information on Nature Portfolio policies, see our Editorial Policies and the Editorial Policy Checklist.

Statistics

For all statistical analyses, confirm that the following items are present in the figure legend, table legend, main text, or Methods section.

n/a	Confirmed	
☐	☒	The exact sample size (*n*) for each experimental group/condition, given as a discrete number and unit of measurement
☐	☒	A statement on whether measurements were taken from distinct samples or whether the same sample was measured repeatedly
☐	☒	The statistical test(s) used AND whether they are one- or two-sided<br>*Only common tests should be described solely by name; describe more complex techniques in the Methods section.*
☐	☒	A description of all covariates tested
☐	☒	A description of any assumptions or corrections, such as tests of normality and adjustment for multiple comparisons
☐	☒	A full description of the statistical parameters including central tendency (e.g. means) or other basic estimates (e.g. regression coefficient) AND variation (e.g. standard deviation) or associated estimates of uncertainty (e.g. confidence intervals)
☐	☒	For null hypothesis testing, the test statistic (e.g. *F*, *t*, *r*) with confidence intervals, effect sizes, degrees of freedom and *P* value noted<br>*Give P values as exact values whenever suitable.*
☒	☐	For Bayesian analysis, information on the choice of priors and Markov chain Monte Carlo settings
☒	☐	For hierarchical and complex designs, identification of the appropriate level for tests and full reporting of outcomes
☐	☒	Estimates of effect sizes (e.g. Cohen's *d*, Pearson's *r*), indicating how they were calculated

Our web collection on statistics for biologists contains articles on many of the points above.

Software and code

Policy information about availability of computer code

Data collection	Electronic data capture (EDC) application and Epidata software v3.1
Data analysis	SAS software v9.4

For manuscripts utilizing custom algorithms or software that are central to the research but not yet described in published literature, software must be made available to editors and reviewers. We strongly encourage code deposition in a community repository (e.g. GitHub). See the Nature Portfolio guidelines for submitting code & software for further information.

Data

Policy information about availability of data

All manuscripts must include a data availability statement. This statement should provide the following information, where applicable:

- Accession codes, unique identifiers, or web links for publicly available datasets
- A description of any restrictions on data availability
- For clinical datasets or third party data, please ensure that the statement adheres to our policy

The data that support the findings of this study are not publicly available due to restrictions on patient privacy, but are available on reasonable request for access to the patient-level data from this study can be submitted via email to the corresponding authors (X.Li: xinli3267@yeah.net and Z.J.: jzhjiazhenhua@163.com) with detailed proposals for use of information, and responses to such requests can be expected within 1month. Depending on the data requested, the data request will be reviewed and, if agreed, a signed agreement with the sponsor is required before accessing the data.

Research involving human participants, their data, or biological material

Policy information about studies with human participants or human data. See also policy information about sex, gender (identity/presentation), and sexual orientation and race, ethnicity and racism.

Reporting on sex and gender	Sex - male or female.<br>In the Qiliqiangxin group, 1,095 participants (70.42%) were male.<br>In the placebo group, 1,148 participants (73.83%) were male.
Reporting on race, ethnicity, or other socially relevant groupings	Ethnicity - Han or other (non-specify).<br>In the Qiliqiangxin group, 1,467 participants (94.34%) were of Han ethnicity.<br>In the placebo group, 1,465 participants (94.21%) were of Han ethnicity.
Population characteristics	Age, sex, ethnicity, body mass index, smoking history, principal cause of heart failure, time from initial diagnosis of heart failure, comorbidity and medical history, etc. Table 1 of the manuscript.
Recruitment	Patients with heart failure and reduced ejection fraction (with LVEF ≤ 40% and NT-proBNP ≥ 450pg/ml) were recruited between May 2019 and May 2021. Recruitment involved obtaining written informed consent from all patients or their legal representatives before inclusion in the trial. To minimize self-selection bias, patients were randomized to receive either QLQX capsules or a matching placebo (4 capsules thrice daily) in addition to standard heart failure therapy. The study design allowed participants to withdraw at any point without needing to provide a reason, enhancing participant autonomy and minimizing withdrawal bias. Despite these efforts, there remains a potential for self-selection bias, as patients who agreed to participate may differ systematically from those who declined, affecting the generalizability of the findings. Additionally, randomization helps mitigate allocation bias, though other unmeasured confounders could still influence the study outcomes. These factors should be carefully considered in interpreting the results to ensure a comprehensive understanding of the study's implications.
Ethics oversight	The protocol was reviewed and approved by the independent ethics committee of the First Affiliated Hospital of Nanjing Medical University (Approved No. of ethic committee: 2018-SR-275) and each ethics committee of the participating study center.

Note that full information on the approval of the study protocol must also be provided in the manuscript.

Field-specific reporting

Please select the one below that is the best fit for your research. If you are not sure, read the appropriate sections before making your selection.

☒ Life sciences ☐ Behavioural & social sciences ☐ Ecological, evolutionary & environmental sciences

For a reference copy of the document with all sections, see nature.com/documents/nr-reporting-summary-flat.pdf

Life sciences study design

All studies must disclose on these points even when the disclosure is negative.

Sample size	According to the previous heart failure RCT, the incidence of cardiovascular death and hospitalization for heart failure was anticipated of 25% in patients with standardized treatment + placebo group and 20% in standardized treatment + Qiliqiangxin capsule group. A sample size of 1540 patients per treatment arm was calculated in order to be able to reject the null hypothesis of equal effect with a power of 80% and a significance level of 5% for a 20% MACE risk reduction among patients treated with QLQX.
Data exclusions	No data were excluded from the primary analyses based on the ITT principle.
Replication	To ensure the reproducibility of our experimental findings, several rigorous measures were implemented throughout the randomized controlled trial. Firstly, the study design incorporated randomization to evenly distribute participants into treatment and placebo groups, thus minimizing selection biases and ensuring comparable baseline characteristics. Both the primary and secondary endpoints were meticulously defined prior to the commencement of the trial, providing clear criteria for assessing outcomes. An independent Clinical Event Adjudication Committee, blinded to treatment allocation, was established to objectively evaluate the predefined endpoints. In addition, the study utilized Full Analysis Set (FAS), Per Protocol Set (PPS), and sensitivity analyses to verify the results. All replication attempts were successful, confirming the reliability and robustness of the study outcomes across different analytical approaches.
Randomization	Statistical experts at Peking University Clinical Research Institute adopts SAS 9.4 statistic software package to generate random numbers using the block randomization method according to the ratio of 1:1 between study group and control group. The study drug (Qiliqiangxin or placebo capsules) was packaged according to this random number by the person unrelated to the study. A randomization and trail supply management system (RTSM) is used in the study, and statistical professionals will provide a random numbered list to the RTSM. At each participating hospital, patients who provided written informed consent and met the study criteria were randomized by investigators, will then assigned a random number by the RTSM.
Blinding	All personnel involved with the analysis of the study will remain blinded until database lock and protocol violations have been identified and documented the study adopts two-step unblinding provision.

Reporting for specific materials, systems and methods

We require information from authors about some types of materials, experimental systems and methods used in many studies. Here, indicate whether each material, system or method listed is relevant to your study. If you are not sure if a list item applies to your research, read the appropriate section before selecting a response.

Materials & experimental systems

n/a	Involved in the study
☒	☐ Antibodies
☒	☐ Eukaryotic cell lines
☒	☐ Palaeontology and archaeology
☒	☐ Animals and other organisms
☐	☒ Clinical data
☒	☐ Dual use research of concern
☒	☐ Plants

Methods

n/a	Involved in the study
☒	☐ ChIP-seq
☒	☐ Flow cytometry
☒	☐ MRI-based neuroimaging

Clinical data

Policy information about clinical studies

All manuscripts should comply with the ICMJE guidelines for publication of clinical research and a completed CONSORT checklist must be included with all submissions.

Clinical trial registration	The trial was registered at http://www.chictr.org.cn, Registration number: ChiCTR1900021929.
Study protocol	The study protocol was published in BMC Complement Med Ther. 2020 Feb 5;20(1):38. and all the corresponding versions are provided in the supplementary material.
Data collection	Participants were recruited from May 2019 to May 2021 in 133 Chinese (mainland and Hong Kong) hospitals. Patients were enrolled and followed up for at least 12 months. All scheduled followed-up were finished in July 2022.
Outcomes	The primary and secondary endoints were predefined in the protocol and SAP based on clinically important endpoints for heart failure. The primary outcome was major adverse composite endpoint of cardiovascular (CV) death and/or hospitalisation for heart failure (HHF). Over a median of 18.3 months follow-up, a primary outcome event occurred in 389 of 1555 patients (25.02%) in the QLQX group and in 467 of 1555 patients (30.03%) in the placebo group (hazard ratio [HR], 0.78; 95% confidence interval [CI], 0.68 to 0.90; p<0.001). This effect was related to both lower risks of HHF and CV death in the QLQX group. HHF occurred in 243 patients (15.63%) in QLQX group and 298 patients (19.16%) in placebo group (HR, 0.76; 95% CI, 0.64 to 0.90; p=0.002). Death from cardiovascular causes occurred in 207 patients (13.31%) in the QLQX group and in 248 patients (15.95%) in the placebo group (HR, 0.83; 95% CI, 0.68 to 0.996; p=0.045). The effect of QLQX on the primary outcome was generally consistent across prespecified subgroups including in patients with or without ARNi and in patients with ischemic etiology. Also, results of the safety endpoint regarding all-cause death, AE, and SEA demonstrated that QLQX were well-tolerated.

Plants

Seed stocks	*Report on the source of all seed stocks or other plant material used. If applicable, state the seed stock centre and catalogue number. If plant specimens were collected from the field, describe the collection location, date and sampling procedures.*
Novel plant genotypes	*Describe the methods by which all novel plant genotypes were produced. This includes those generated by transgenic approaches, gene editing, chemical/radiation-based mutagenesis and hybridization. For transgenic lines, describe the transformation method, the number of independent lines analyzed and the generation upon which experiments were performed. For gene-edited lines, describe the editor used, the endogenous sequence targeted for editing, the targeting guide RNA sequence (if applicable) and how the editor was applied.*
Authentication	*Describe any authentication procedures for each seed stock used or novel genotype generated. Describe any experiments used to assess the effect of a mutation and, where applicable, how potential secondary effects (e.g. second site T-DNA insertions, mosiacism, off-target gene editing) were examined.*
